# Targeting Aquaporin Function: Potent Inhibition of Aquaglyceroporin-3 by a Gold-Based Compound

**DOI:** 10.1371/journal.pone.0037435

**Published:** 2012-05-18

**Authors:** Ana Paula Martins, Alessandro Marrone, Antonella Ciancetta, Ana Galán Cobo, Miriam Echevarría, Teresa F. Moura, Nazzareno Re, Angela Casini, Graça Soveral

**Affiliations:** 1 REQUIMTE, Departamento de Química, Faculdade de Ciências e Tecnologia, Universidade Nova de Lisboa, Caparica, Portugal; 2 Dipartimento di Scienze del Farmaco, Università G. d'Annunzio, Chieti, Italy; 3 Instituto de Biomedicina de Sevilla (IBiS), Hospital Universitario Virgen del Rocío/CSIC/Universidad de Sevilla, Seville, Spain; 4 Pharmacokinetics, Toxicology and Targeting, Research Institute of Pharmacy, University of Groningen, Groningen, The Netherlands; 5 Departamento de Bioquímica e Biologia Humana, Faculdade de Farmácia, Universidade de Lisboa, Lisbon, Portugal; New Mexico State University, United States of America

## Abstract

Aquaporins (AQPs) are membrane channels that conduct water and small solutes such as glycerol and are involved in many physiological functions. Aquaporin-based modulator drugs are predicted to be of broad potential utility in the treatment of several diseases. Until today few AQP inhibitors have been described as suitable candidates for clinical development. Here we report on the potent inhibition of AQP3 channels by gold(III) complexes screened on human red blood cells (hRBC) and AQP3-transfected PC12 cells by a stopped-flow method. Among the various metal compounds tested, Auphen is the most active on AQP3 (IC_50_ = 0.8±0.08 µM in hRBC). Interestingly, the compound poorly affects the water permeability of AQP1. The mechanism of gold inhibition is related to the ability of Au(III) to interact with sulphydryls groups of proteins such as the thiolates of cysteine residues. Additional DFT and modeling studies on possible gold compound/AQP adducts provide a tentative description of the system at a molecular level. The mapping of the periplasmic surface of an homology model of human AQP3 evidenced the thiol group of Cys40 as a likely candidate for binding to gold(III) complexes. Moreover, the investigation of non-covalent binding of Au complexes by docking approaches revealed their preferential binding to AQP3 with respect to AQP1. The high selectivity and low concentration dependent inhibitory effect of Auphen (in the nanomolar range) together with its high water solubility makes the compound a suitable drug lead for future *in vivo* studies. These results may present novel metal-based scaffolds for AQP drug development.

## Introduction

AQPs belong to a highly conserved group of membrane proteins called the major intrinsic proteins (MIPs) present in all type of organisms and involved in the transport of water and small solutes such as glycerol, nitrate and urea [1]. The 13 human AQP isoforms (AQP0-12) are differentially expressed in many types of cells and tissues in the body and can be divided into two major groups: those strictly selective for water (called orthodox aquaporins) and those that besides water are also permeable to small solutes including glycerol (called “aquaglyceroporins”) [2]. Both groups of channels are involved in many pathophysiological conditions [3], [4]. There is considerable potential for transferring knowledge of AQP structure, function and physiology to the clinic, and certainly there is great translational potential in aquaporin-based therapeutics. AQP-based modulator drugs are predicted to be of broad potential utility in the treatment of several diseases such as kidney diseases, cancer, obesity, glaucoma, brain edema and epilepsy [5]. In particular, recent studies have correlated AQP3 glycerol permeation with skin tumorigenesis [6] and identified it as being aberrantly expressed in melanoma [7], suggesting that AQP3 might be a novel target for skin tumor prevention and therapy.

There are at present very few reported AQP inhibitors that are suitable candidates for clinical trials and none of them showed specificity for AQP3 so far. Though various AQPs are inhibited by mercurial compounds, such as HgCl_2_ [8], these substances are non-selective in their action and extremely toxic. Other inorganic salts such as AgNO_3_ and HAuCl_4_, that are prone to interact with sulfhydryl groups of proteins as mercurials, have been also shown to inhibit water permeability in plasma membrane from roots, and in particular AgNO_3_ has been reported to efficiently inhibit water permeability in human red blood cells (EC_50_ = 3.9 µM) [9]. Various other candidate blockers of AQP1 have been also reported, including tetraethyl-ammonium [10], acetazolamide [11] and DMSO [12]; however, other studies indicated little or no AQP1 inhibition by tetraethylammonium salts or acetazolamide [13] and apparently inhibition by DMSO results from an osmotic clamp effect rather than true inhibition [14]. Several papers reported AQP4 inhibition by a series of arylsulfonamides, antiepileptic drugs and related molecules, with strong inhibition at low micromolar concentrations [15], [16]; yet, these results could not be confirmed, with no inhibition activity found even at high concentrations of any of the putative AQP4 inhibitors [17]. An AQP4 inhibitor (2-nicotinamido-1,3,4-thiadiazole) was also shown to reduce cerebral edema in rodent models [18], a radio-labeled version of which has been developed to study AQP distribution *in vivo* using PET [19]. Migliati et al. reported on AQP1 and AQP4 inhibition by an analogue of the sulfonamide Bumetamide [20], that was also recently found to reduce cerebral edema in rodent models [21] via AQP4 inhibition. Recently, Jelen et al. identified novel small molecule inhibitors of AQP9 glycerol permeability; however, since their solubility in aqueous solution is very limited, these compounds are currently not suitable for *in vivo* experiments [22].

**Figure 1:**
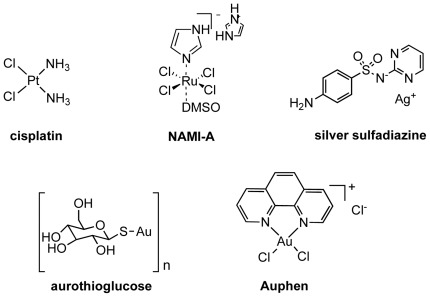
Metal compounds used in this study.

Within this frame, we decided to reconsider metal-based compounds as possible AQP inhibitors, and we report here the inhibitory effect on the water and glycerol permeability mediated by AQP1 and AQP3 of a series of metal complexes based on different transition metals. The selected compounds are metal-based drugs already known to possess different therapeutic properties as anticancer, antirheumatic and antibacterial agents. Among them were the anticancer drug *cis*-[PtCl_2_(NH_3_)_2_] (cisplatin) [23], the antimetastatic trans-[Ru(dmso)(Him)Cl4] (dmso = dimethylsulfoxide, Him = imidazole, NAMI-A) [24], the antibacterial Ag(I) sulfadiazine (AgSDZ) [25], and the antirheumatic agent aurothioglucose (AuTG) [26] (Figure 1). In addition, the gold(III) compound [Au(phen)Cl_2_]Cl (phen = 1,10-phenatroline, Auphen) [27], [28], [29] showing antiproliferative properties on cancer cells *in vitro*, was also selected. For most of these compounds, the mechanisms of pharmacological actions are still poorly understood [30]. Therefore, AQPs also appeared to be interesting to investigate as putative targets for metal-based drugs.

The effect of the compounds was tested by a stopped-flow method on human red blood cells (hRBC) that specifically express large amount of AQP1 and AQP3 [31], [32], and confirmed on transfected PC12 cell lines with overexpression of either AQP1 or AQP3. In all cases, the gold(III) complex Auphen was the most potent inhibitor and showed selectivity towards AQP3. Further studies on a selected series of gold(III) complexes supported the idea of the necessity of direct binding of gold ions to the protein to achieve glycerol permeability inhibition. Additional DFT calculations on the interactions of gold(III) complexes with model amino acids side chains, as well as docking studies investigating the non-covalent binding of gold(III) complexes with both the known structure of AQP1 and a homology model of AQP3, allowed to provide a tentative description of the putative mechanisms of inhibition and to explain the compounds' selectivity for AQP3.

## Results

### Inhibition of water and glycerol permeability by metal compounds

The effect of different metallo-drugs based on Pt(II), Ru(III), Ag(I), Au(I,III) (Figure 1) was tested on water and glycerol permeability of hRBCs. For this purpose hRBCs incubated in isotonic phosphate buffered saline (PBS) solution were challenged with hypertonic sucrose solution (impermeant solute, inducing cell shrinkage) or hypertonic glycerol solution (permeant solute, cells shrink due to the hyper-osmotic gradient and re-swell due to glycerol entrance). Since hRBCs were shown to express large amount of AQP1 and AQP3 accountable for membrane permeability to water and glycerol [32], [33], [34], this assay allows the direct evaluation of these aquaporins activity and is thus a promising screening assay for modulators of aquaporin function. From the rate of cell volume changes (shrinkage and re-swelling) after imposed osmotic shocks, the membrane permeability for water and for glycerol can be calculated [34].

**Figure 2:**
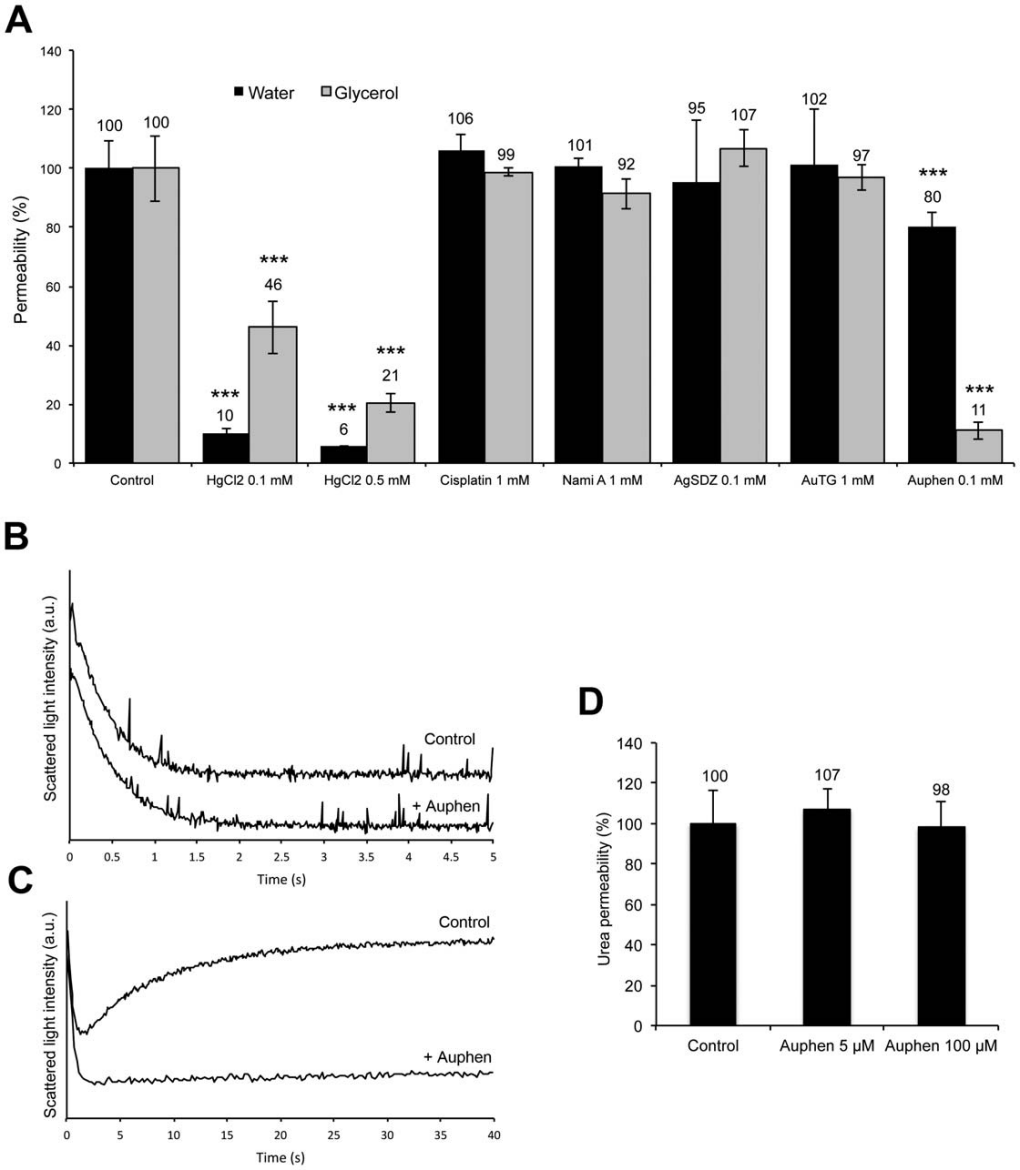
Effect of compounds on hRBC permeability. (A) Water and glycerol permeabilities (% of control) after treatment with the compounds under study and with HgCl_2_ (10 min at r.t.) A marked effect of Auphen (100 µM) is depicted (***P<0.001). (B) Stopped flow representative traces of water and (C) of glycerol permeability (control and after incubation with 5 µM Auphen, 30 min at r.t.). (D) Urea permeability showing no significant effect of Auphen treatment (5 and 100 µM, 30 min at r.t.; P>0.05).

Figure 2A shows the effect induced by the metal complexes (0.1 mM concentration or higher) on AQP1 and AQP3 (10 min incubation at room temperature) in comparison to HgCl_2_, a well-known inhibitor of aquaporin activity, tested in the same conditions. The obtained results demonstrated that the Au(III) complex Auphen is the most effective of the series on glycerol permeability, and far more effective than the mercurial compound (with a statistical significance of P<0.001). For control hRBCs, the osmotic water (*P_f_*) and glycerol (*P_gly_*) permeability values were respectively (4.2±0.4)×10^−2^ cm s^−1^ (n = 5) at 10°C and (1.8±0.2)×10^−5^ cm s^−1^ (n = 5) at 23°C. As shown in Figure 2A, Auphen showed a modest effect on water permeability (ca. 20% inhibition), while being able to drastically reduce glycerol transport with a residual permeability of ca. 11% (90% inhibition). The smaller effect obtained for water permeability points to a more potent effect on AQP3, which itself is also a water-transporting channel [35], [36] but with a smaller contribution to the total bulk of water flow through hRBC membranes where AQP1 is the main water channel [32].

Representative traces of stopped-flow experiments with control and hRBCs treated with even lower concentration of Auphen (5 µM, incubated 30 min at room temperature) showing the inhibition of the osmotic water flux or glycerol flux are reported in Figure 2B and 2C, respectively.

To assure the compound selectivity, the effect of Auphen was also tested on urea transport. Notably, no significant effect could be observed for both concentrations tested in comparison to the controls (P>0.05, n = 5) (Figure 2D). The ability of AQP3 to transport urea has been debated [37], [38]. In the erythrocyte, the urea transporter UT-B [39] accounts for a very high urea permeability reducing osmotic shrinkage of RBCs while passing through the kidney. Therefore, compared to urea transporters, urea permeability through AQP3 may have only a negligible contribution and thus its inhibition would not be enough to observe decreased urea permeability. Since Auphen did not affect urea permeability, this result indicates that this metallodrug is specific for AQP3 not having any effect on UT-B.

**Figure 3:**
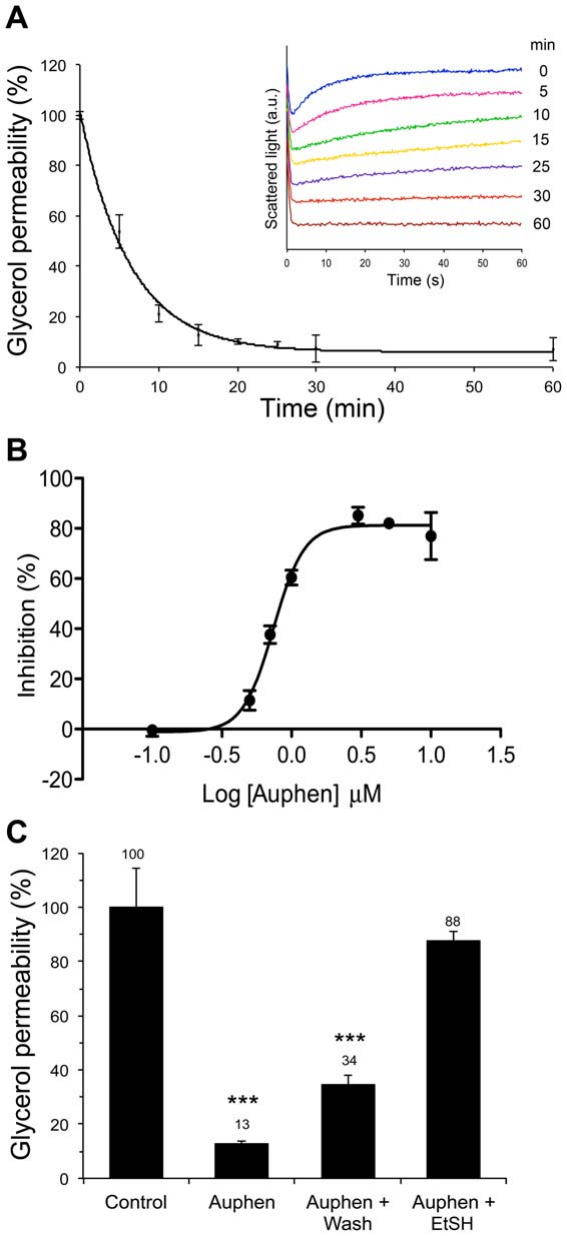
Effect of Auphen on hRBC glycerol permeability. (A) Time dependent inhibition of glycerol permeability by 5 µM Auphen. The inset shows the progressive decrease of glycerol permeability observed in the assays where the increase in cell volume due to glycerol entrance decreases drastically with the incubation time. (B) Concentration dependent inhibition of glycerol permeability in hRBC by Auphen (compound concentrations in the range 0.1–10 µM; IC_50_ = 0.8±0.08 µM). (C) Inhibition of glycerol permeability (% of control) of hRBCs after Auphen treatment (2 µM, 30 min at r.t.), and reversibility by washing with PBS or incubation with 2-mercaptoethanol (1 mM for 30 min) (***P<0.001).

Following these promising results we further investigated the inhibition of glycerol transport through AQP3 evaluating the effect of the incubation time of hRBCs with Auphen. Figure 3A shows the inhibitory effect of a fixed concentration of Auphen (5 µM) where a maximum inhibition, after an exponential decay of activity, could be observed after 30 min incubation of the samples at r.t. It is worth mentioning that no cell hemolysis was detected even after 4 hours incubation with the compound, pointing to a non-toxic inhibitory effect. Subsequently, the concentration dependent inhibition of glycerol transport in hRBC by Auphen incubated 30 min at r.t. was assessed (Figure 3B). According to the obtained results the IC_50_ value for Auphen was calculated as 0.8±0.08 µM.

The activation energy (*E_a_*) for water and glycerol transport, a valuable parameter indicating the contribution of protein channels to permeation, was also estimated from an Arrhenius plot (Figure S1). Upon treatment of hRBC with 5 µM Auphen, similar *E_a_* values for water transport were obtained for control (3.9±0.4 kcal mol^−1^) and Auphen treated hRBCs (4.1±1.0 kcal mol^−1^). However, the *E_a_* for glycerol permeation increased ca. 54% when Auphen was present (8.5±0.8 to 13.2±1.1 kcal mol^−1^). Since the Auphen concentration used in the assay was higher than the IC_50_ (corresponding approximately to 80% inhibition), the increase in *E_a_* is in accordance with a blockage of the AQP3 channel. Regarding water transport, the observed variation on the *E_a_* was not significant (P>0.05); indeed, in hRBC the contribution of the channel pathway to the total water permeability is usually considered to be 90% while the bilayer adds the remaining 10% [40]. The total 20% inhibition observed with Auphen (Figure 2) would exclusively reduce the contribution of the aquaporin (AQP1+AQP3) pathway maintaining the lipid pathway intact, thus reflecting a decrease of the total permeability only to ca. 87.5% of its initial value. Since the channel contribution for the total permeability still remains very large, the *E_a_* for water transport is predicted not to be significantly affected.

### Recovery of aquaporin activity by mercaptoethanol

In order to assess the reversibility of inhibition by Auphen, hRBCs pre-treated with 2 µM Auphen for 30 min r.t. were subsequently washed with PBS or with the reducing agent 2-mercaptoethanol (EtSH, 1 mM in PBS). As seen in Figure 3C, washing the sample twice with PBS had a limited effect in the recovery of Auphen inhibition of glycerol permeability. Conversely, incubation of the Auphen-treated hRBC sample with EtSH for 30 min produced an almost complete recovery of glycerol permeability (ca. 90%), suggesting that EtSH effect on cysteine residues is competing with Auphen binding to the pore [9].

The results obtained with EtSH, as well as the known affinity of gold ions for binding to sulfhydryl groups of proteins, suggest that AQP3 inhibition by Auphen might involve direct protein binding of the Au centre to Cys residues as it has already been reported for HgCl_2_. Indeed, mercury inhibition is likely to occur both via covalent modification to Cys189 located immediately after the extracellular entrance of the water pore of hAQP1 [41] and also to other regions of the protein, causing either blockage or conformational changes with a resultant inhibition of water transport [42].

### Selectivity of AQP3 inhibition in transfected cell lines

**Figure 4:**
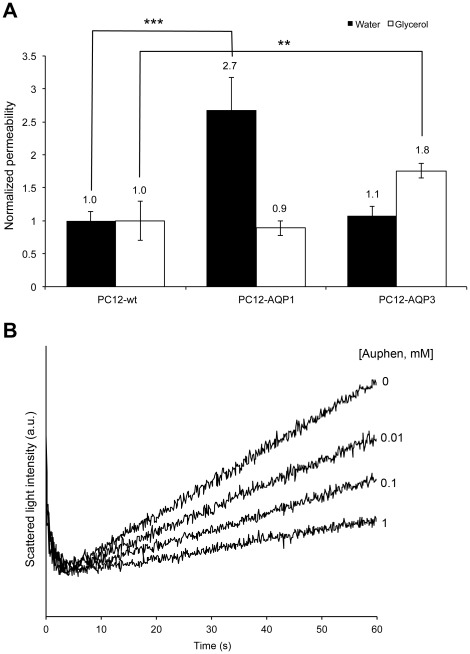
PC12 cell lines assays. (A) Permeability to water and glycerol of wild type PC12 cells and PC12 transfected with either AQP1 or AQP3. Significant differences were found for the PC12-AQP3 clones (**P<0.01) for glycerol permeability, and for the PC12-AQP1 for water permeability (***P<0.001) when compared to PC12-wt. (B) Effect of Auphen on glycerol permeability of PC12-AQP3 transfected cells (30 min incubation at r.t.).

To further confirm the specific effect of Auphen on AQP3 glycerol transport, the inhibition of glycerol permeation by Auphen was also assessed on PC12 cells stably transfected with rat AQP1 or AQP3 (Table 1) [43]. Cell permeability for water and glycerol was analyzed by stopped-flow experiments and results are depicted in Figure 4A. The enhancement of water permeability for AQP1 overexpressing cells (2.7 -folds) and that of glycerol permeability for AQP3 overexpressing cells (1.8 -folds) correlates well with their respective protein level of expression (Table 1). When treated with Auphen (10 to 1000 µM, 30 min incubation at r.t.) a decrease in glycerol permeability was only observed for the PC12-AQP3 cell line, reaching 72% inhibition (Figure 4B); none of the other two cell lines (wild type and PC12-AQP1) were affected regarding glycerol. As for water permeability, only the PC12-AQP1 showed an inhibition of ca. 25% for 100 µM Auphen. It must be noted that higher concentrations of Auphen were needed to produce the same inhibitory effect observed in hRBCs. Besides the fact that Auphen might have a lower affinity for rat AQP3 than for human AQP3, the possibility of Auphen binding to other reactive groups within the whole cell membrane decreasing its effective concentration in the media and therefore leading to an underestimation of the IC_50_ should not be disregarded. The PC12 cells are much larger than RBCs; hence, the presence of a larger number of these bias reactive groups may eventually contribute to a higher decrease in the effective Auphen concentration available for AQP3 blockage.

**Table 1:** Folds of expression and permeabilities of PC12 cells.

Clone	Expression level	*P_Gly_*×10^−7^ (cm s^−1^)	*P_f_*×10^−2^ (cm s^−1^)
PC12wt	1.0	2.16±0.64	3.76±0.50
AQP1	4.0	1.91±0.25	10.11±1.84
AQP3	2.0	3.78±0.24	4.00±0.52

Folds of mRNA expression were determined by Northern blot analysis and are normalized relative to the clone PC12wt.

### Analysis of the mechanisms of AQP3 inhibition by gold(III) compounds

**Figure 5:**
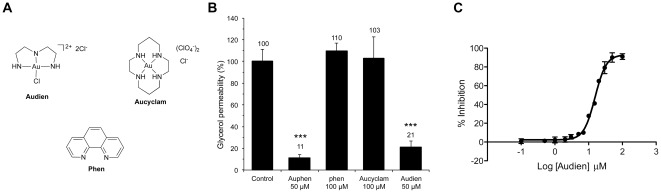
AQP3 inhibition by Au(III) compounds in hRBC. (A) Structures of the Au(III) complexes Audien and Aucyclam, and of the ligand phen. (B) Effect of Auphen, phen, Aucyclam and Audien on glycerol permeability (% of control) after 30 min incubation at r.t. (***P<0.001). (C) Dose response curve of Audien (IC_50_ = 16.62±1.61 µM).

In order to evaluate our mechanistic hypothesis that sees the gold centre as responsible for AQP3 inhibition, the ligand Phen, as well as the Au(III) complexes [Au(dien)Cl]Cl_2_ [28], (dien = diethylentriamine, Audien) and [Au(cyclam)](ClO_4_)_2_Cl [28] (cyclam = 1,4,8,11-tetraazacyclotetradecane, Aucyclam) (Figure 5A) were tested for glycerol transport inhibition in hRBCs. It must be noted that Auphen and Audien, with AuN_2_Cl_2_ and AuN_3_Cl cores, can undergo substitution of the chlorides with other nucleophiles present in physiological environment. Instead, Aucyclam is a gold(III) complex with a AuN_4_ chromophore which lacks of chemical activation resulting into poor reactivity and scarce biological (e.g. anticancer) effects [28]. The obtained results, plotted in Figure 5B, confirm that while Audien at a concentration of 50 µM reaches an inhibition of 80% (lower than the 90% inhibition observed for Auphen at the same concentration), its IC_50_ is 20-fold higher than observed for Auphen (IC_50_ = 16.6±1.6 µM, Figure 5C). Notably, similarly to Auphen, the inhibition of AQP3 by Audien reached its maximum at 30 min incubation. On the other hand, Aucyclam and Phen are completely inactive at any tested concentrations and incubation times.

### DFT calculations

The inhibition of AQP3 by Auphen and Audien is believed to be related to the coordination of the Au(III) centre to a protein residue in the pore, presumably cysteine, methionine or histidine side-chains [44]. In order to establish the possibility of such a mechanism and to elucidate possible structure-activity relationships, DFT studies were performed to determine the molecular geometry and the binding energy of these two selected Au(III) compounds with several models of soft metal ion-binding protein sites (Figure S2). A large body of knowledge on Au(III) chemistry in water solutions [27] indicates that Auphen, and presumably also Audien, is predominantly in its intact or hydroxo forms at neutral pH, the diaquo form of Auphen being essentially negligible (Table S1). Thus, both chloro and monohydroxo species, namely [Au(phen)Cl_2_]^+^ or [Au(phen)Cl(OH)]^+^, and [Au(dien)Cl]^2+^ or [Au(dien)(OH)]^2+^), were taken into account for the DFT evaluation of the Auphen and Audien binding at putative protein target residues through the calculation of thermodynamics of the ligand exchange process (Figure S3, Table S1) between Cl/OH and suitable models of the side chains of Cys (L = CH_3_SH, CH_3_S^−^), Met (L = CH_3_SCH_3_), and His residues (L = CH_3_-Im), in different polarity media.

The trend in Au–L binding strength estimated at this level of theory (see Table S1) was CH_3_S>CH_3_Im>CH_3_SCH_3_>CH_3_SH indicating the thiolate form of Cys as the most stable binding site for Au(III) complexes, and the only one showing a favorable reaction free energy with both Cl^−^ and OH^−^ ligands, together with the CH_3_Im which, however, show a much smaller reaction free energy. It must be noted that, while at physiological pH cysteine residues are predominantly in their neutral form, therefore appearing the least favorable target of Au(III) binding, calculations on the acidic dissociation of thioalcohol complexes gave negative free energy values and very low pK_a_'s (below zero), indicating the facile deprotonation at neutral and even acidic pH with formation of a metal-thiolate complex (Figure S3 and Figure S4).

It is worth noting that the reaction free energy for Cl^−^ substitution by thiolate is much more favorable than for OH^−^, thus suggesting that the ligand reactivity for Au(III) complexes is different from that for cisplatin and other Pt(II) complexes where the aqua-species are well known to be more reactive. This could be due to the differences in redox properties of the Au(III) compounds with respect to the Pt(II) complexes. Finally, the free energy for Cl^−^ substitution by thiolate is slightly but significantly, 10–20 kJ mol^−1^, more favorable for Auphen than for Audien, suggesting a stronger binding of Aupen to Cys, in agreement with its experimentally observed higher AQP3 inhibition.

### Construction of the homology model of AQP3 and comparison with AQP1 structure

Three homology models of human AQP3, namely hmod1, hmod2 and hmod3, were obtained by the use of freely available web-servers SWISS-MODEL, Phyre, and ESyPred, respectively, based on the structurally characterized *Escherichia coli* glycerol facilitator (GlpF) [45], the bacterial homolog of human AQP3, or on its W48F, F200T mutant [46] as templates. The obtained models (Dataset S1) were characterized by a high degree of structure similarity, as evidenced by the overall good superimposition of helix domains and by the small values of RMSD of Cα backbones. In addition, a moderate degree of structure similarity was detected by the comparison of either side chain or loop conformations, thus suggesting that the three homology models may actually be characterized by similar binding properties. We thus selected the hmod1 structure because it was characterized by a slightly more favored ratio of model completeness: 250/292 residues for hmo1 with respect to 249/292 for both hmod2 and hmod3.

The family of the aquaporins and the aquaglyceroporin subfamily are known to share a common protein fold. It comprises six membrane-spanning helices plus two half-helices with their positive, N-terminal ends located at the centre of the protein and their C-terminal ends pointing towards either side of the membrane. The helices surround the 20-Å-long and 3–4-Å-wide amphipathic AQP channel. AQPs are identified by two asparagine-proline-alanine (NPA) sequence motifs located at the ends of the two quasi 2-fold related half-spanning helices. The selectivity filter, a constricted region formed by four residues near the periplasmic/extracellular entrance, provides distinguishing features that identify the subfamilies. In water selective AQPs this region is smaller and more polar and contains a conserved histidine residue, while in aquaglyceroporins it is larger and more hydrophobic with two conserved aromatic residues [47]. Two conserved constriction sites are present in the channel. An aromatic/Arg (ar/R) constriction is located at the extracellular pore mouth. Its diameter determines whether or not solutes, such as glycerol and methylamine, can pass the AQP in addition to water [48], [49], [50]. Furthermore, the positively charged residues in this region form an energy barrier for protons. The second constriction resides in the centre of the channel, where the positive ends of the two half-helices meet. The helix dipole moments add up to a full positive charge, and the resulting electrostatic field poses another energy barrier for cations [51].

**Figure 6:**
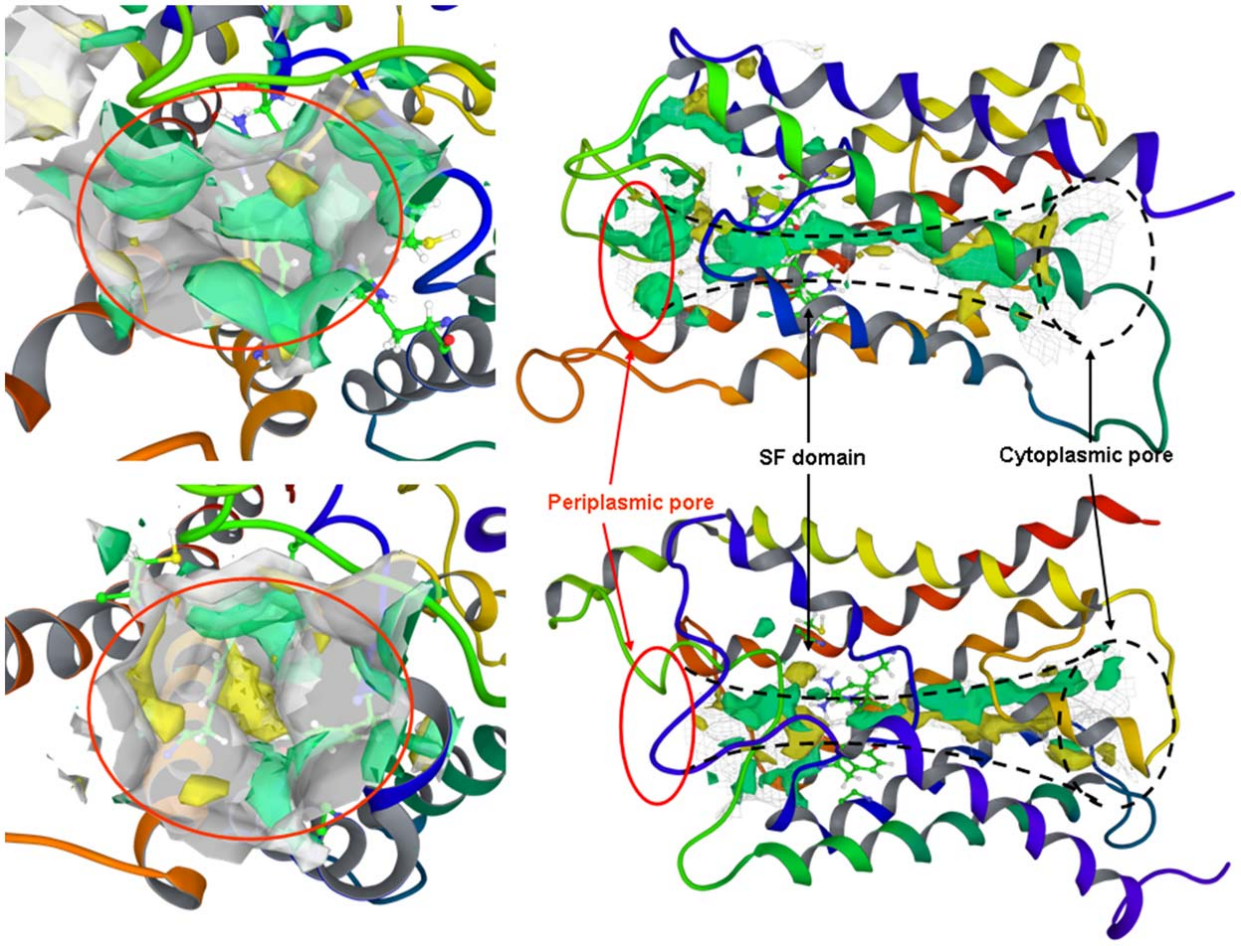
Identification of protein binding pockets. Ribbon view of the whole protein channel (left) and periplasmic pocket (right) with molecular surfaces displayed for AQP1 (top) and AQP3 (bottom). Hydrophilic and hydrophobic isosurfaces are also shown in light green and yellow, respectively.

The structure of our homology model of AQP3 was analyzed in detail and compared with the crystal structure of human AQP1 to characterize the different propensity of both proteins for the binding of the considered Au(III) complexes. Thus, the two AQP1 and AQP3 structures were analyzed by the SiteMap module of Maestro for the identification and characterization of protein binding pockets. Two major binding pockets were observed in both AQP1 and AQP3, and, as expected, the mapping retraced the cavities corresponding to the water channels identifying a binding pocket for each side of the protein, namely a periplasmic and a cytoplasmic pocket separated by the selectivity filter (SF) domain (Figure 6) [47].

The SiteMap comparison of periplasmic pockets of AQP1 and AQP3 evidenced the main distinctive features of these protein surfaces. In particular, an extended hydrophobic region was detected in proximity of the SF of AQP3, but not in AQP1, the latter showing a markedly higher hydrophilic character. This result is in agreement with several literature evidences suggesting the different hydrophilic/hydrophobic balance on the periplasmic pocket as the major difference between orthodox aquaporin and aquaglycerolporin subfamilies, including AQP1 and AQP3, respectively [52].

Afterwards, the position and the chemical environment of the most suitable soft-metal ion protein binding sites, such as cysteine, methionine and histidine side chain residues, was analyzed in detail. First of all, it must be noted that AQP3 is characterized by a higher content of these residues (5 Cys, 5 Met and 6 His) in comparison to AQP1 (4 Cys, 1 Met and 5 His). The mapping of the periplasmic surface provided a valuable assessment of these possible gold-binding sites location and exposure on both aquaporin isoforms. In AQP1, Cys189 and His180 resulted to be the only residues proximal to the periplasmic pore, therefore, directly accessible to gold complexes. However, the exposure of these residues is quite scarce and, being part of the SF domain, they are located in the narrowest section of the membrane filter, which can be easily approached only by small molecules. Moreover, being the size filter function a conserved property of SF [47], the transition to a “more open” conformation with enhanced exposure of Cys189 and His180 is very unlikely.

**Figure 7:**
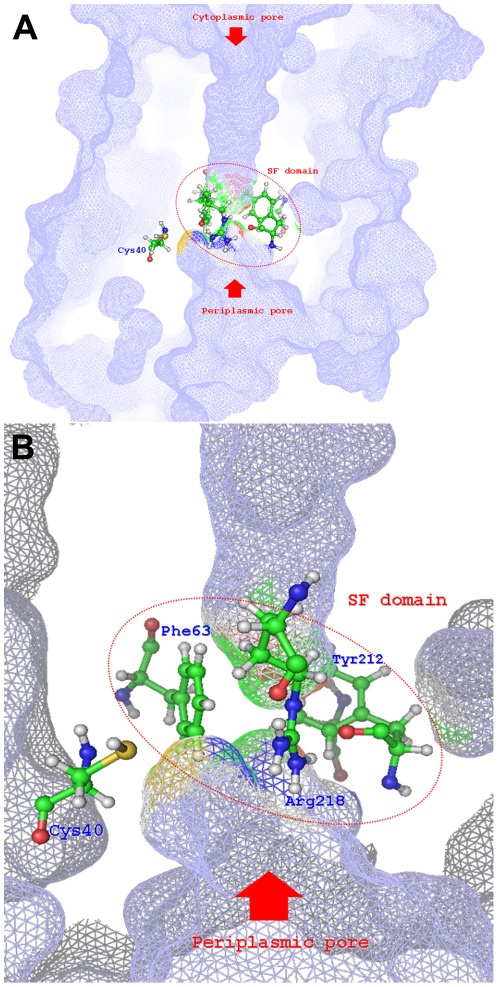
Molecular surface of AQP3 periplasmic pocket. (A) SF domain residues together with Cys40 side chain are displayed. (B) Colour scheme representing the contributor atoms to the molecular surface is also shown.

On the other hand, AQP3 is characterized by the presence of a MxxC motif located on the H1 domain, close enough to the periplasmic pocket to be potentially targeted by metal complexes. In this homology model, while Met37 is projected toward the outer space of the protein (quite far from the pore surface and probably in close contact with the membrane phospholipidic layer), the thiol group of Cys40 is projected toward the periplasmic space approaching the channel pore (Figure 7). The proximity of Cys40 to the SF domain is further indicated by the partial burying of this residue's side chain by the guanidine moiety of Arg218. Thus, among the possible gold binding sites available on AQP3, the side chain of Cys40 is the most favorable, being very close to the SF domain and in the right orientation.

### Docking study of the non-covalent adducts of Au complexes with AQP1 and AQP3

**Figure 8:**
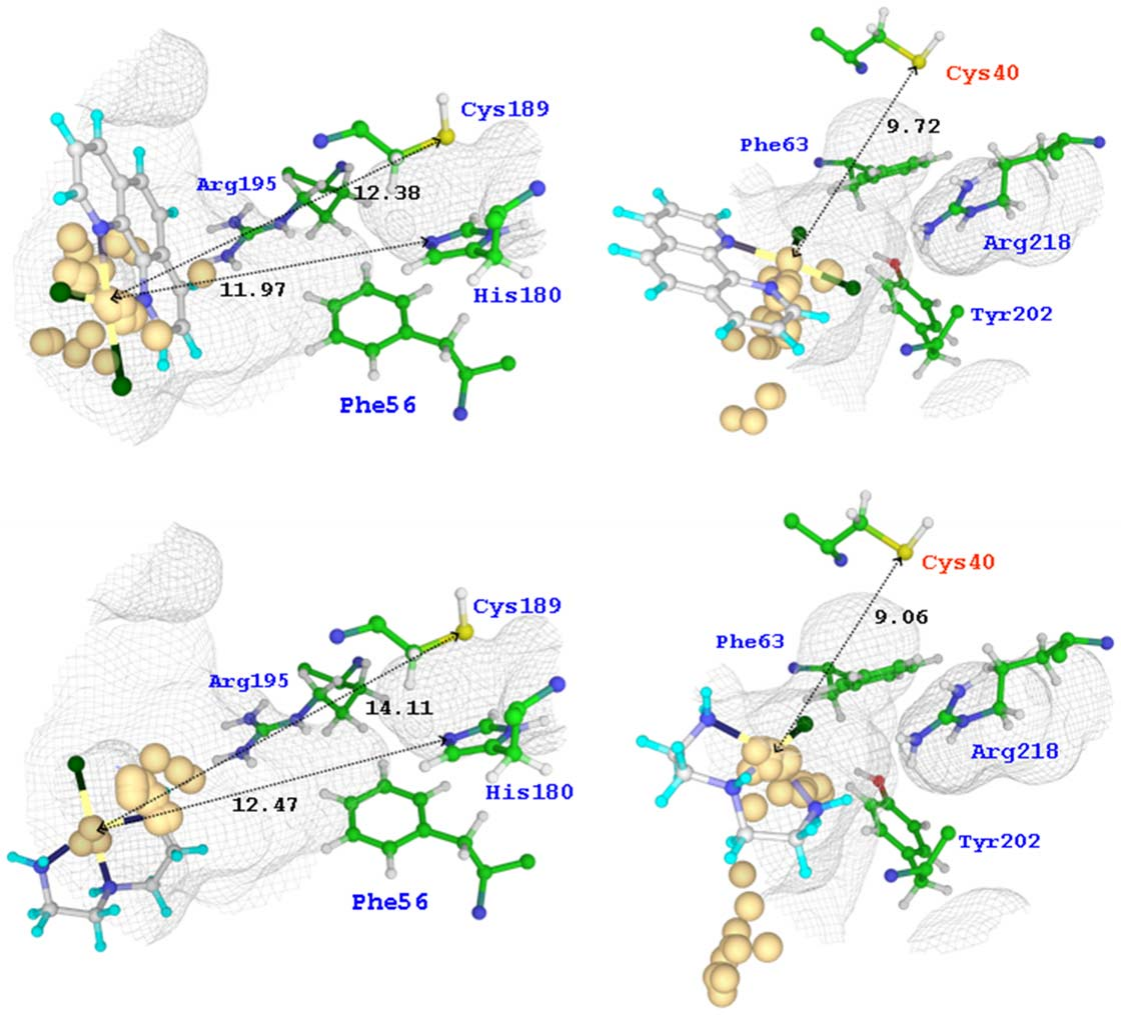
Docking of Auphen and Audien on AQP1 and AQP3. Top ranked poses of Auphen (top) and Audien (bottom) at periplasmic pocket of AQP1 (left) and AQP3 (right). Positions of metal centre for the other calculated poses are also displayed in golden yellow. SF domain residues are labelled in blue. Au–S and Au–N distances are reported in Å.

Although the Auphen and Audien inhibition is essentially irreversible, a preliminary docking calculation has been carried out to evaluate the accessibility of the proposed cysteine binding side of AQP1 and AQP3, trying to identify non-covalent binding poses and possible steric crashes. Thus, we considered the same species assumed in the DFT calculations, *i.e.* Auphen and Audien and their monohydroxo derivatives, and through the calculation of the docking poses and their corresponding scoring we made a preliminary estimation of both the affinity and accessibility of these metal complexes to the targeted pores. In particular, the molecular geometries of the Au(III) complexes obtained at DFT level of theory were rigidly docked into the periplasmic pocket of both AQP1 and AQP3 by using the Glide algorithm. The results of docking studies are summarized in Table S2, and the most representative calculated poses are depicted in Figure 8. In general, docking calculations showed that the non-covalent interaction of both Au(III) complexes is favored in the periplasmic pocket of both AQP1 and AQP3. On the other hand, the interaction of both Au(III) complexes with AQP3 is slightly stronger as indicated by both the scoring of top ranked poses and average score values (Table S2). Additionally, the analysis of the docking poses indicated the AQP3 periplasmic pocket as the most sterically accessible to the interaction of the considered Au(III) complexes. Indeed, as shown in Figure 8, the top ranked poses of both Au(III) complexes approached closer to the SF domain when docked at AQP3, thus favoring the binding of these species at a protein site closer to the constriction pore.

It is also worth noting that the Cys40 thiol group in AQP3 is in proximity (9–10 Å) and well-oriented with respect to the gold centre of top ranked poses (Figure 8), thus confirming to be a suitable metal binding site upon minimal adaptation of the protein structure. On the other hand, the side chains of Cys189 and His180, the only two possible metal sites at AQP1, are more distant from the gold ion (12–14 Å) and not favorably oriented to be easily bound by Auphen and Audien.

## Discussion

While AQP1 is a selective water channel, AQP3 permeates both water and glycerol on human erythrocytes. In spite of being also a water-transporting channel [35], [36] its contribution to the total bulk of water flow through hRBC membranes is minimal compared to AQP1 [32]. Conversely, AQP3 has been shown to mediate most of the glycerol movements across RBCs membranes [32], [34]. In addition to its expression in hRBCs, AQP3 has a wide tissue distribution in the epithelial cells of kidney, airways and skin, suggesting a role in water reabsorption, mucosal secretions, skin hydration and cell volume regulation [53]. Moreover, a recent study reported an aberrant AQP3 expression in melanoma and proved that it significantly increases chemoresistance to arsenite treatment [7].

In the present study we report on the screening of different known metallodrugs, with various therapeutic applications, for the inhibition of AQP1 and AQP3. Thus, the potent and selective inhibition of the glycerol permeability through AQP3 in hRBC by water soluble gold(III) complexes, namely Auphen and Audien, was observed, while the other metal complexes were completely ineffective as AQPs inhibitors. Both the effective compounds are tetracoordinated gold(III) complexes with square planar geometry in which the Au(III) oxidation state, at variance with the case of NaAuCl_4_, is stabilized by the presence of nitrogens on the phenantroline and diethylentriamine ligands. Auphen and Audien resulted to inhibit glycerol transport in hRBC with an IC_50_ = 0.8±0.08 µM and IC_50_ = 16.6±1.6 µM, respectively, while having only a modest inhibitory effect on water permeability. Moreover, the gold(III) compounds also resulted to be ineffective as inhibitor of urea transport on hRBC.

The observed increase in the *E_a_* for glycerol transport in hRBC with no concomitant *E_a_* change for water transport upon treatment with Auphen, points to a potent blockage of the AQP3 channel with a much lower effect on AQP1. A similar effect has already been reported in the case of Cu^2+^ and Ni^2+^ ions, which were shown to inhibit selectively AQP3 in transfected human cell lines, although at higher concentrations in the mM range [54], [55]. Notably, the Au(III) compounds are also much more effective on AQP3 than the non-specific inhibitor of aquaporins HgCl_2_, and most importantly they are not toxic for the cells in the entire range of investigated concentrations as suggested by the absence of hemolysis and by the recovery of activity after treatment with EtSH.

The specificity of Auphen towards AQP3 was further confirmed by assessing glycerol transport on PC12 cell lines transfected with either AQP1 or AQP3 from rat. The marked inhibitory effect of the compound for the PC12-AQP3 cell clone denotes its higher affinity for AQP3 residues.

The time-dependent inhibition of AQP3 by Auphen is in accordance with the typical reactivity pattern of this gold(III) complex in aqueous solution. It is worth mentioning that, as commonly found for several other metallodrugs, gold(III) compounds behave as *“prodrugs”* [56]. In other words, their adducts with biomolecular targets are often the result of a specific chemical transformation (e.g. ligand substitution, redox processes, hydrolysis) of the compounds. In the case of Auphen, AQP3 binding is most likely achieved through substitution of at least one halide ligand from the tetracoordinated gold(III) chromophore AuN_2_Cl_2_ [28]. Similarly, Audien can bind AQP3 after replacement of the unique chloride ligand, but slightly less efficiently than Auphen as demonstrated by its higher IC_50_ value. Conversely, the chemically stable Au(III) complex Aucyclam shows lack of inhibition properties supporting the hypothesis that the gold centre is essential for inhibition and most likely is involved in protein binding.

The mechanism of gold inhibition is probably due to the ability of Au(III) to interact with sulphydryl groups of proteins such as the thiolate of cysteine. This hypothesis is partly confirmed by the almost complete recovery of AQP3 activity upon treatment of hRBC with EtSH. However, other modes of Auphen binding might also occur (e.g. with histidine groups). In order to shed light on the putative binding modes of gold complexes to AQPs we used DFT analysis. Thus, DFT calculations on the reactions of Auphen and Audien with model side chain residues indicated the thiolate form of Cys as the most favorable binding site for Au(III) complexes both in the intact or mono-hydroxo species.

To further investigate the mechanisms of AQP inhibition by gold compounds at a molecular level, we undertook molecular modeling studies. Initially, a homology model of human AQP3 was built and compared to the structure of human AQP1 via molecular modeling SiteMap analysis, allowing the identification and characterization of protein binding pockets. Our model confirmed the well-established presence of an extended hydrophobic area in the periplasmic region of AQP3, while a marked hydrophilic character was evidenced in the same region of AQP1. This important difference might account for a higher binding affinity of the Auphen complex for AQP3 with respect to Audien, resulting in higher inhibition potency, being the phenantroline ligand likely to establish hydrophobic interactions at the entrance of the glyceroporin channel. Most importantly, the mapping of the periplasmic surface allowed establishing the possible gold binding sites and their exposure on both aquaporin isoforms. In AQP1 none of the cysteine, methionine or histidine residues appear to be accessible for gold binding, including Cys189 previously reported to be the binding site of mercurial AQP inhibitors [42]. Conversely, in AQP3 the thiol group of Cys40 is projected towards the periplasmic space approaching the channel pore and, therefore, it is proposed here as a likely candidate for binding to gold(III) complexes.

The investigation of non-covalent binding of Auphen and Audien and their monohydroxo species at AQP1 and AQP3 by docking approaches allowed to ascertain the possibility for the gold complexes to reach the SF domain of AQP3 in closer proximity with respect to AQP1, therefore allowing the compounds to bind at protein sites closer to the constriction pore of the aquaglyceroporin such as the side chain of Cys40. Indeed, it is known that the AQP1 channel cross-section size is the major determinant of selectivity for larger amphipathic molecules such as glycerol [47]. Thus, the same steric restrictions which account for the poor glycerol permeation by pure water channels with respect to aquaglyceroporins, might also apply to gold(III) complexes. In addition, the analysis of docking scores evidenced a slightly higher affinity of the two compounds for the AQP3 periplasmatic pore, characterized by a higher extent of hydrophobic sites with respect to AQP1. The highest docking scores for the approach of Auphen at the targeted periplasmic pore, together with the strongest binding of Auphen to Cys shown by our DFT calculations, can explain the slightly higher inhibitory potency demonstrated by Auphen.

Interestingly, both Auphen and Audien have been previously reported to possess anticancer properties *in vitro* [28], [29]. Indeed, in recent years several gold(III) compounds have shown promising anticancer effects related to the inhibition of different protein targets, such as the proteasome and specific zinc finger proteins [57], [58], [59], [60]. In this context, we cannot exclude that inhibition of AQP3 might influence the biological effects of the compounds towards cancer cells, although other studies need to be performed to validate such a hypothesis.

In conclusion, metal compounds, in particular mercurial salts, have already been reported as inhibitors of AQPs, but have major drawbacks such as poor selectivity and extreme toxicity, which hamper their development as therapeutic agents. We described here the selective and potent inhibitory effect (in the nanomolar range) of two Au(III) complexes bearing nitrogen donor ligands on AQP3, which together with their limited toxicity and high water solubility makes them suitable candidates for future *in vivo* studies. These results may present novel metal-based scaffolds for AQP drug development.

## Methods

### Chemistry

Gold compounds and NAMI-A were prepared according to literature procedures (see references throughout the text). The purity of the compounds was confirmed by elemental analysis, and all of them showed purity greater than 98%. Cisplatin, silver sulfadiazine, aurothioglucose and 2-Mercaptoethanol were from Sigma.

### Ethics Statement

Venous blood samples were obtained from healthy human volunteers following a protocol approved by the Ethics Committee of the Faculty of Pharmacy of the University of Lisbon. Informed written consent was obtained from all participants. Rat adrenal medulla pheochromocytoma cells (PC12) were kindly provided by Dr. M. Eschevarria, Virgen del Rocio University Hospital, Seville [33].

### Erythrocyte sampling and preparation

Venous blood samples, collected in citrate anticoagulant (2.7% citric acid, 4.5% trisodium citrate and 2% glucose). Fresh blood was centrifuged at 750×*g* for 5 min at 4°C and plasma and buffy coat were discarded. Packed erythrocytes were washed three times in PBS (KCl 2.7 mM, KH_2_PO_4_ 1.76 mM, Na_2_HPO_4_ 10.1 mM, NaCl 137 mM, pH 7.4), diluted to 0.5% haematocrit and immediately used for experiments [34].

### Cell Culture and Transfections

To obtain stable clones of PC12 (cell line derived from a pheochromocytoma of rat adrenal medulla) that overexpress either rat AQP1 or rat AQP3, twenty micrograms of pcDNA3-AQP1 or pcDNA3-AQP3 were transfected into wild type PC12 by electroporation. After selection with geneticin sulphate (GIBCO) 40 clones were analyzed for levels of expression of either AQP1 or AQP3. Out of 20 positive clones with variable levels of AQPs expression, for each AQP those with higher expression were selected. PC12 cells were cultured in Dulbecco's modified Eagle's medium (Invitrogen) supplemented with 5% foetal bovine serum, 10% horse serum, and 1% penicillin/streptomycin (Invitrogen) in a CO_2_ (10%) incubator at 37°C. Geneticin at 0.2 mg/ml was added to culture AQP-overexpressing clones. Levels of AQP overexpression were determined by Northern blot analysis and expressed relative to wild type PC12. RNA analysis and functional characterization of these clones have been described in detail previously [43].

### Cell volume measurements

hRBC mean volume in isotonic solution was determined using a CASY-1 Cell Counter (Schärfe System GmbH, Reutlingen, Germany) and was calculated as 82 fL. Equilibrium volumes of PC12 cells were obtained by phase contrast microscopy on an inverted microscope (Axiovert Zeiss 100M) equipped with a digital camera.

Plated cells were dislodged by mechanical aspiration with pipette, washed and re-suspended in PBS. For each measured set of data, an aliquot of cell suspension was placed on a microscope slide and an average of 6 pictures with 4–6 cells each were taken and analyzed using NIH ImageJ software. Cells were assumed to have a spherical shape with a diameter calculated as the average of the maximum and minimum dimensions of each cell. Calculated volumes were 1076±123 µm^3^ for all the clones measured.

### Stopped-flow light scattering experiments

Stopped-flow experiments were performed on a HI-TECH Scientific PQ/SF-53 apparatus, with 2 ms dead time, temperature controlled and interfaced with a microcomputer. Experiments were performed at 23°C for glycerol permeability and at 10°C for water and urea permeability; for activation energy measurements temperatures were ranged from 10°C to 37°C. For each experimental condition, 5–7 replicates were analyzed. For measuring the osmotic water permeability (*P_f_*), 100 µL of a suspension of fresh erythrocytes (0.5%) or PC12 cells (1.5×10^3^ to 3.5×10^3^ cells/mm^3^) was mixed with an equal volume of PBS containing 200 mM sucrose as a non-permeable osmolyte to produce a 100 mM inwardly directed sucrose gradient [34]. The kinetics of cell shrinkage was measured from the time course of 90° scattered light intensity at 400 nm until a stable light scatter signal was attained.

*Pf* was estimated by *Pf = k (V_o_/A)(1/V_w_(osm_out_)_∞_)*, where *V_w_* is the molar volume of water, *V_o_/A* is the initial cell volume to area ratio and *(osm_out_)_∞_* is the final medium osmolarity after the applied osmotic gradient and *k* is the single exponential time constant fitted to the light scattering signal of erythrocyte shrinkage. For PC12 cells, a double exponential function was used instead and the weighted averaged rate constant k_de_ = (ΔI_1_k_1_+ΔI_2_k_2_)/(ΔI_1_+ΔI_2_), where ΔI_1_ and ΔI_2_ correspond to the signal changes with either a slow rate constant k_1_ or a fast one k_2_, was alternatively used to calculate *P_f_* [61].

For glycerol permeability (*P_gly_*), 100 µL of erythrocyte or PC12 cell suspension was mixed with an equal volume of hyperosmotic PBS containing 200 mM glycerol creating a 100 mM inwardly directed glycerol gradient. After the first fast cell shrinkage due to water outflow, glycerol influx in response to its chemical gradient was followed by water influx with subsequent cell reswelling. *P_gly_* was calculated as *Pgly = k (V_o_/A)*, where *k* is the single exponential time constant fitted to the light scattering signal of glycerol influx in erythrocytes [34]. The same protocol was performed for urea permeability, but using instead a hyperosmotic PBS solution containing 200 mM urea. For PC12 cells, the rate of re-swelling due to glycerol influx was measured as the slope of a linear regression fit.

For inhibition experiments cells were incubated with different concentrations of complexes, from freshly prepared stock aqueous solutions, for various times at room temperature before stopped-flow experiments. A time dependent inhibition assay for Auphen over several hours incubation with hRBC and PC12 cells showed no further increase of inhibition after 30 min at r.t. A similar time dependent inhibition assay was performed for all the compounds up to 4 hours incubation at r.t. except for the highly toxic HgCl_2_ (10 min, r.t.). The inhibitor concentration necessary to achieve 50% inhibition (IC_50_) was calculated by nonlinear regression of dose-response curves (Graph Pad Prism, Inc) to the equation: y = y_min_+(y_max_−y_min_)/(1+10^((LogIC50-[Inh]) H))^, where y is the percentage inhibition obtained for each concentration of inhibitor [Inh] and H is the Hill slope. The activation energy (*E_a_*) of water and glycerol transport was calculated from the slope of the Arrhenius plot (ln*P_f_* or ln*P_gly_* as a function of 1/*T*) multiplied by the gas constant *R*. All solution osmolarities were determined from freezing point depression on a semi-micro osmometer (Knauer GmbH, Berlin, Germany) using standards of 100 and 400 mOsM.

### Statistic analysis

Data were presented as mean ± standard error of the mean (SEM) of at least four independent experiments, and were analyzed with either the paired Student's t-test or one-way analysis of variance (ANOVA) followed by Tukey's test. A value of P≤0.01 was considered to be statistically significant.

### QM calculations

The structures of Au(III) complexes, i.e. Auphen and Audien, and their respective hydrolyzed forms were investigated at DFT level of theory with the B3LYP hybrid functional [62], [63], which is known to give good descriptions of transition metal-containing compounds [64], [65]. The core electrons of the chloride and metal atoms were described with the Hay and Wadt core-valence relativistic effective core-potential (ECP) leaving the outer electrons to be treated explicitly through the basis set denoted as LACV3P* in Jaguar [66], while for the remaining atoms the 6-311G** basis set was used [67]. Each structure was optimized in the gas phase (ε = 0) and frequency calculations were performed to verify the correct nature of the stationary points and to estimate zero point energy (ZPE) and vibrational entropy corrections at room temperature. Single point energies of all stationary points were calculated by using the larger 6-311++G** set for the main group elements, and the LACV3P++** set for the metal and the chloride atoms. The Poisson-Boltzmann (PB) continuum solvent method was employed to simulate ε>0 environments, namely a low-polarity medium resembling the inner protein with ε = 4 and an aqueous medium with ε = 80 [68].

The thermodynamics of Auphen and Audien aquation (I) and acidity of the corresponding aquo (II) and methanethiol (III) complexes was investigated by means of the thermodynamic cycles depicted in Figure S2. The reaction free energy for the exchange processes involving metallodrug species was first calculated at DFT level of theory while the free energy values for the processes of interest, i.e. Δ*G°*_I_, Δ*G°*_II_ and Δ*G°*_III_, were obtained as reported in Figure S2. The Δ*G°* values obtained by this indirect procedure are expectedly subjected to less approximations than those calculated for the respective direct processes because the inclusion of empirical parameters such as the solvation energy of proton and chloride ion, characterized by large and negative values (and thus affected by larger approximation), is avoided. The corresponding values of *pK_a_* for the acidic dissociation reaction II and III were calculated by the equation *pK_a_* = Δ*G°_rea_*/2.303 *RT*, where *R* is the ideal gas constant and *T* is 298.15 K.

The Jaguar 7.7 quantum chemistry package was used for all calculations [69].

### Molecular modeling

The crystal structure of AQP1 was downloaded from the PDB archive (entry code 1H6I) [70] and processed in the Maestro environment [71]. Thus, the pdb structure of AQP1 underwent the Protein Preparation workflow to remove water and other co-crystallized molecules, to assign the most plausible formal charge to the ionisable protein residues and to eventually add hydrogen atoms. After these steps, the protein structure was relaxed in a OPLS2001 [72], [73] force field through a conjugate-gradient minimization algorithm and a gradient-based convergence threshold of 0.3 kJ mol^−1^ Å^−1^, allowing to preserve the main features of the parent crystal conformation in the final structure of AQP1.

The 3D structure of AQP3, not yet resolved, was obtained by homology modeling on accessible web-servers as follow. Initially, possible AQP3 homologues were searched using the HHmod web-server [74], [75]. Among the top ranked homologues 1LDA and 1LDF [46] were selected as templates for subsequent homology modeling on the above cited web-servers. However, the homology models obtained by 1LDF as template were either incomplete or presenting structural misfolding, so that only the three models generated by 1LDA were considered afterwards, namely hmod1, hmod2 and hmod3 from Swiss-Model, Phyre, and ESyPred, respectively.

The three homology models were subjected to a Protein Preparation procedure by the same treatment employed for AQP1 (see above). After this step, the structures of AQP1 and hmod1-3 were superimposed in the Maestro workspace by using the protein alignment tool and then processed by the SiteMap module [76]. This tool adopts a grid-based procedure to map the space surrounding the protein structure by employing volumetric, electrostatic and hydrophobic probes. By the identification and characterization of protein cavities, SiteMap is able to retrace the possible binding pockets from a protein structure. The molecular surface, the hydrophobic and hydrophilic isosurfaces of each identified site are measured and used to calculate the corresponding site score.

The SiteMap calculation on AQP1 was performed by essentially using the default parameters with the exception of the mapping resolution set to 0.3 to gain the maximum mapping accuracy.

Docking calculations were carried out with Glide 3.5 that makes use of a series of hierarchical filters to calculate the ligand binding poses in the active-site region of the target [77]. The periplasmic pore of AQP1 and AQP3 was selected as docking active region through a 30×30×30 grid centered on to the center of mass of SF domain residues for each protein structure, namely residues 56, 180 189 and 195 for AQP1 and residues 63, 203 212 and 218 for AQP3. The positional space of Au(III) complexes was instead limited to a 15×15×15 box centered as already mentioned; these settings are default for grid calculation with Glide3.5 [77]. Docking was performed using the standard parameters set-up for rigid docking in which only the roto-translational degrees of freedom were explored and metal complexes kept the corresponding DFT geometry. The evaluation of binding pose quality was performed in terms of GlideScore as function of ligand-target molecular mechanics interaction energy and ligand strain energy. A maximum of 30 docking poses per metal complex were searched in each docking run.

## Supporting Information

Figure S1 Arrhenius plots showing the water and glycerol permeability of hRBS, in the absence (control) or presence of 5 µM Auphen (30 min incubation at r.t. previous to permeability measurements). Activation energy (*E_a_*) values for water transport were not affected by Auphen treatment (3.9±0.4 kcal mol^−1^ for control and 4.1±1.0 kcal mol^−1^ for Auphen treated hRBCs). The *E_a_* for glycerol permeation increased ca. 54% when Auphen was present (8.5±0.8 to 13.2±1.1 kcal mol^−1^).
(TIF)

Figure S2 Reaction of Au(III) complexes with soft-metal protein sites investigated at DFT level of theory.
(TIF)

Figure S3 Two-step formation of the Au–thiolate adduct between Auphen and Cys side chain.
(TIF)

Figure S4 Calculation of reaction free energies in water for the I) aquation of Audien (X = NH_2_) and Auphen (X = Cl), II) acidic dissociation of their respective aquo forms and III) acidic dissociation of corresponding thiol adducts, and the corresponding *pKa* values of the two latter processes. All free energy values were reported in kJ mol^−1^. Values of *K_hyd_* ^Pt^ and *K_a1_* ^Pt^, referred to the cisplatin first hydrolysis and to the acidity of the corresponding monaquo form, respectively, were taken from B. Lippert, “Cisplatin” 1999, Wiley-VCH, Weinheim, Germany, pp 184, 186.
(TIF)

Table S1 Calculated enthalpies and free energies for the reaction of Auphen and Audien with soft-metal protein sites in different polarity media. All values are in kJ mol^−1^.
(DOC)

Table S2 Docking score of Auphen and Audien at periplasmic pocket of AQP1 and AQP3.
(DOC)

Dataset S1 Calculated homology modelling structures (pdb format) of AQP3. Available at http://www.ff.ul.pt/fct/dataset_s1.pdf.
(PDF)
