# Supplementary figures and images for: Targeting Aquaporin Function: Potent Inhibition of Aquaglyceroporin-3 by a Gold-Based Compound
Source: PLoS One. 2012 May 18;7(5):e37435. doi: 10.1371/journal.pone.0037435 (PMC3356263; doi:10.1371/journal.pone.0037435)

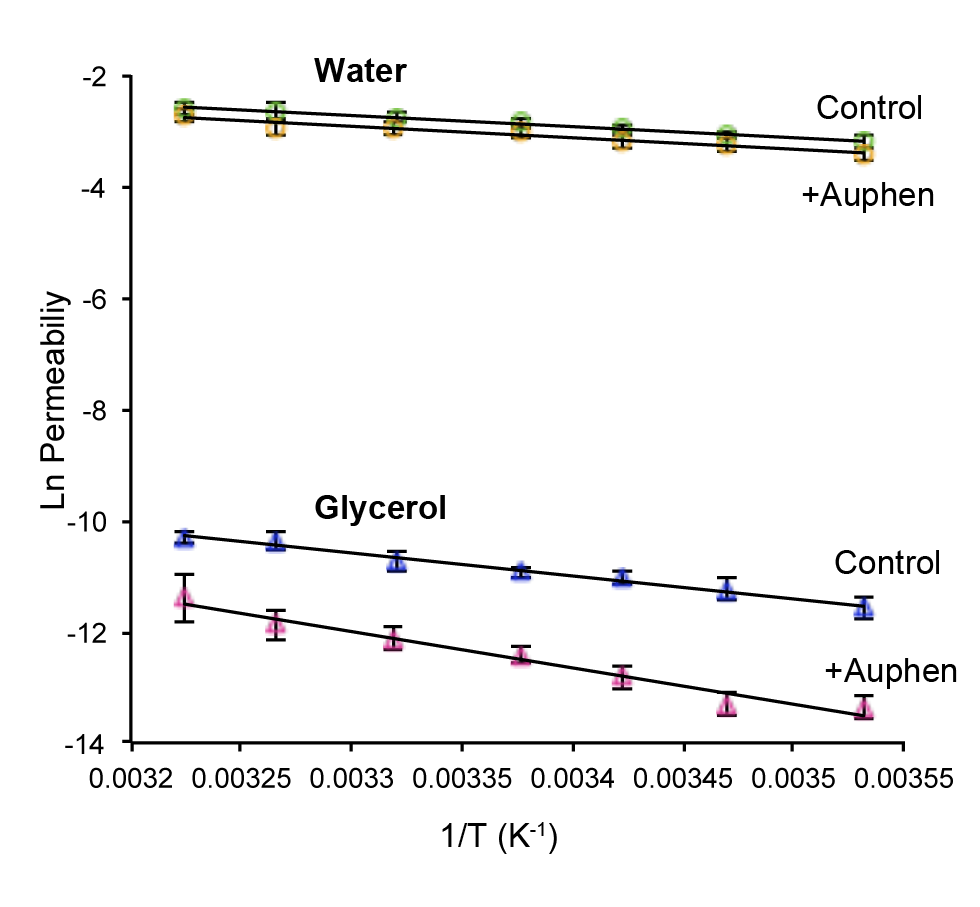

Supplement: Figure S1 — Arrhenius plots showing the water and glycerol permeability of hRBS, in the absence (control) or presence of 5 µM Auphen (30 min incubation at r.t. previous to permeability measurements). Activation energy (Ea) values for water transport were not affected by Auphen treatment (3.9±0.4 kcal mol−1 for control and 4.1±1.0 kcal mol−1 for Auphen treated hRBCs). The Ea for glycerol permeation increased ca. 54% when Auphen was present (8.5±0.8 to 13.2±1.1 kcal mol−1). (TIF) [file pone.0037435.s001.tif]

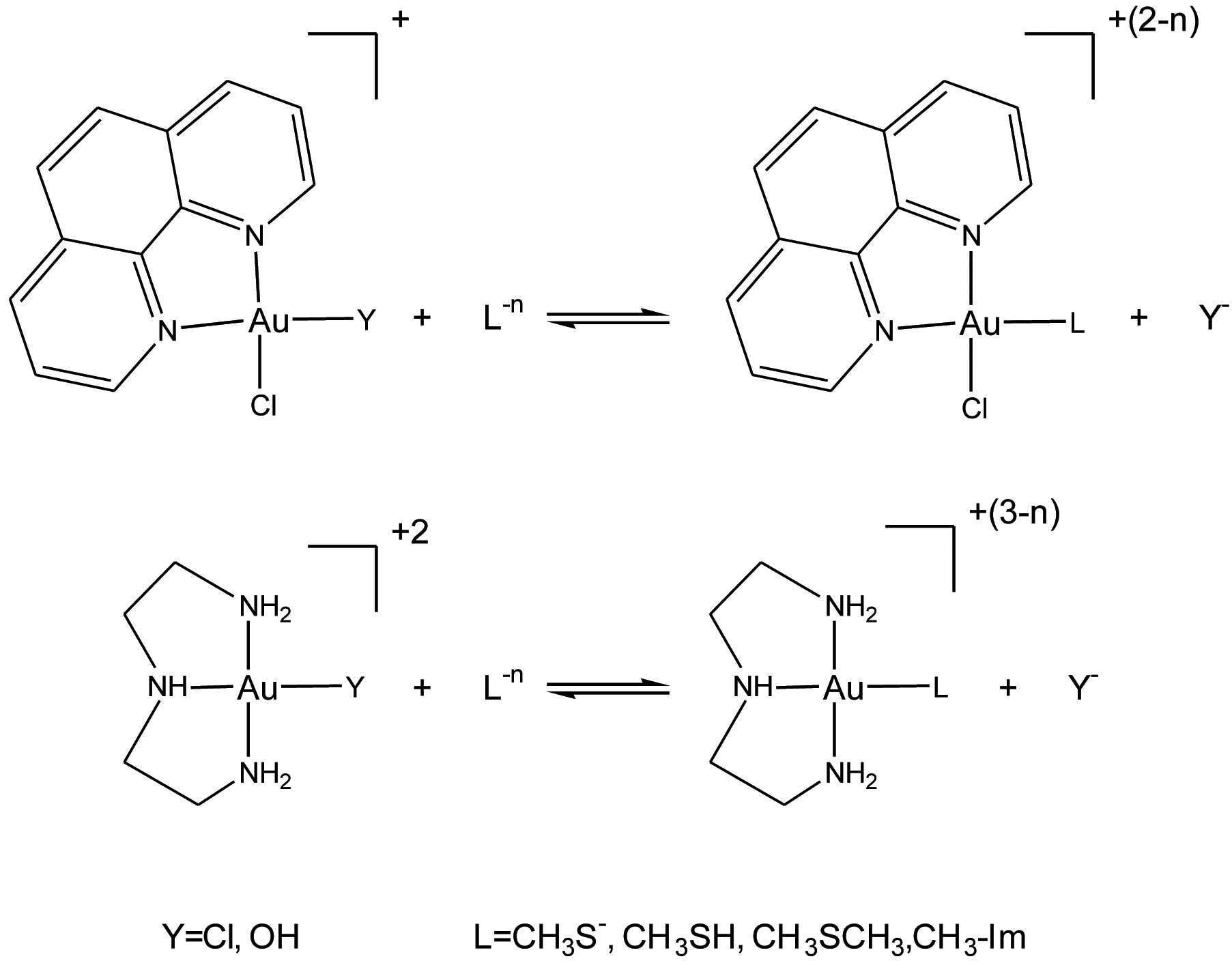

Supplement: Figure S2 — Reaction of Au(III) complexes with soft-metal protein sites investigated at DFT level of theory. (TIF) [file pone.0037435.s002.tif]

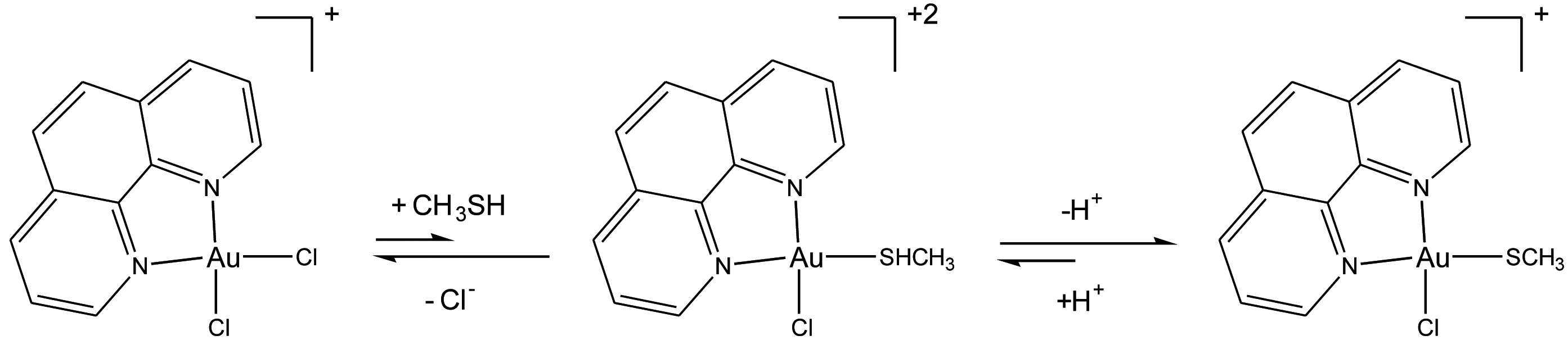

Supplement: Figure S3 — Two-step formation of the Au–thiolate adduct between Auphen and Cys side chain. (TIF) [file pone.0037435.s003.tif]

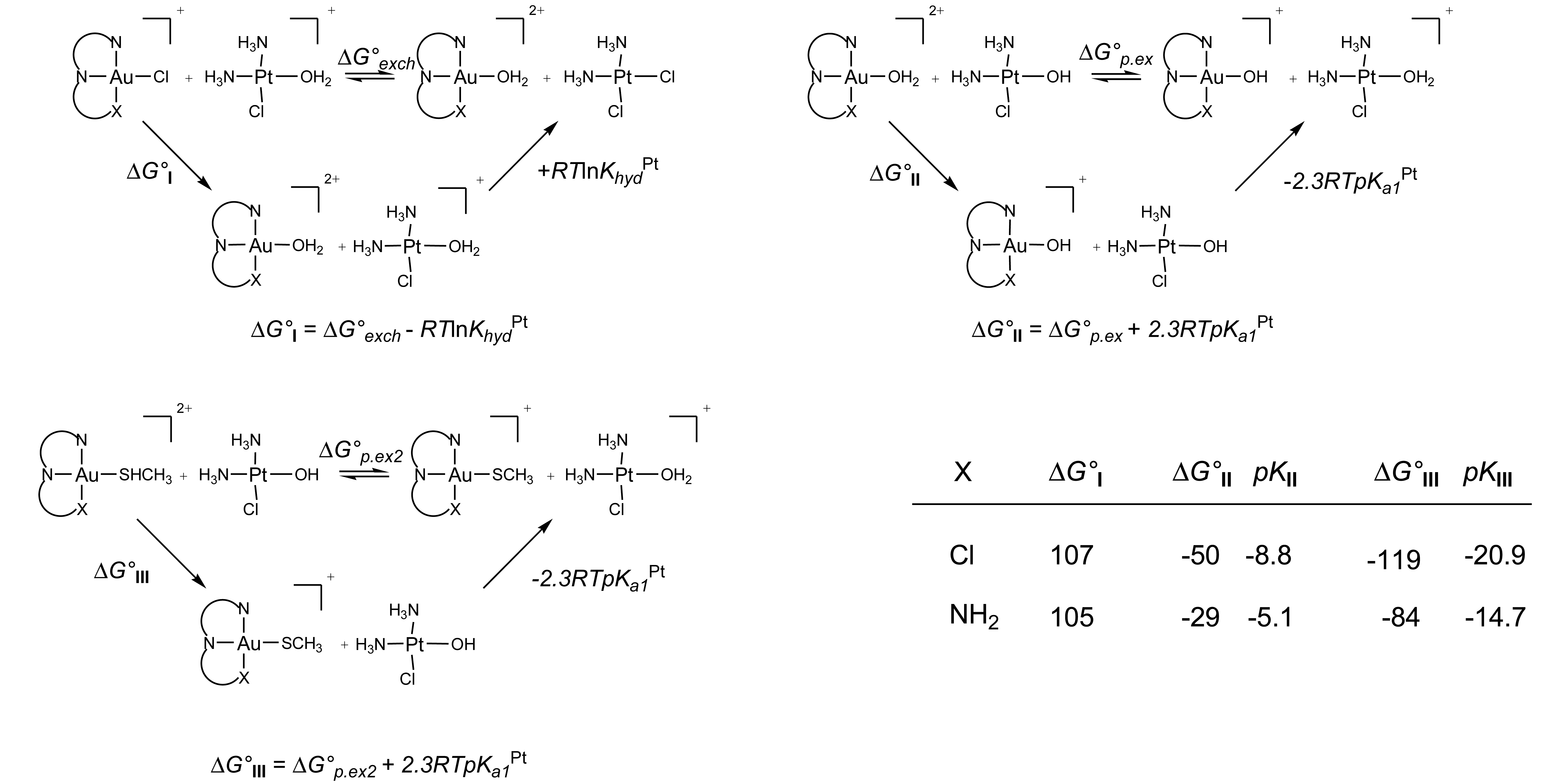

Supplement: Figure S4 — Calculation of reaction free energies in water for the I) aquation of Audien (X = NH2) and Auphen (X = Cl), II) acidic dissociation of their respective aquo forms and III) acidic dissociation of corresponding thiol adducts, and the corresponding pKa values of the two latter processes. All free energy values were reported in kJ mol−1. Values of Khyd Pt and Ka1 Pt, referred to the cisplatin first hydrolysis and to the acidity of the corresponding monaquo form, respectively, were taken from B. Lippert, “Cisplatin” 1999, Wiley-VCH, Weinheim, Germany, pp 184, 186. (TIF) [file pone.0037435.s004.tif]
