# Supplementary material for: Targeting Aquaporin Function: Potent Inhibition of Aquaglyceroporin-3 by a Gold-Based Compound
Source: PLoS One. 2012 May 18;7(5):e37435. doi: 10.1371/journal.pone.0037435 (PMC3356263; doi:10.1371/journal.pone.0037435)
Supplement: Table S1 — Calculated enthalpies and free energies for the reaction of Auphen and Audien with soft-metal protein sites in different polarity media. All values are in kJ mol−1. (DOC) [file pone.0037435.s005.doc]

**Table S1.** Calculated enthalpies and free energies for the reaction of Auphen and Audien with soft-metal protein sites in different polarity media. All values are in kJ mol^-1^.

| Complex | L | Y | ΔH^298^ | | | ΔG^298^ | | |
| --- | --- | --- | --- | --- | --- | --- | --- | --- |
|  |  |  | ε=0 | ε=4 | ε=80 | ε=0 | ε=4 | ε=80 |
| Auphen | CH_3_S^–^ | Cl | -153.0 | -144.8 | -128.6 | -145.2 | -137.0 | -120.8 |
|  |  | OH | 45.5 | -14.9 | -59.7 | 47.6 | -12.9 | -57.7 |
|  | CH_3_SH | Cl | 756.3 | 181.1 | -2.3 | 769.1 | 193.9 | 10.5 |
|  |  | OH | 954.9 | 310.9 | 66.6 | 961.9 | 318.0 | 73.6 |
|  | CH_3_SCH_3_ | Cl | 710.2 | 153.7 | -20.3 | 725.1 | 168.6 | -5.4 |
|  |  | OH | 908.8 | 283.5 | 48.6 | 917.9 | 292.7 | 57.8 |
|  | CH_3_–Im | Cl | 647.7 | 121.0 | -38.9 | 661.6 | 134.8 | -25.0 |
|  |  | OH | 846.3 | 250.8 | 30.0 | 854.4 | 258.9 | 38.1 |
| Audien | CH_3_S^–^ | Cl | -177.0 | -137.6 | -113.2 | -167.2 | -127.9 | -103.5 |
|  |  | OH | 3.5 | -23.8 | -59.0 | 7.9 | -19.4 | -54.7 |
|  | CH_3_SH | Cl | 1136.8 | 278.6 | 4.5 | 1148.3 | 290.2 | 16.0 |
|  |  | OH | 1317.3 | 392.5 | 58.6 | 1323.4 | 398.6 | 64.8 |
|  | CH_3_SCH_3_ | Cl | 1073.0 | 257.7 | 0.7 | 1085.3 | 270.1 | 13.0 |
|  |  | OH | 1253.5 | 371.6 | 54.8 | 1260.5 | 378.5 | 61.8 |
|  | CH_3_–Im | Cl | 963.2 | 202.3 | -33.0 | 978.7 | 217.8 | -17.5 |
|  |  | OH | 1143.7 | 316.2 | 21.2 | 1153.8 | 326.2 | 31.2 |
