# Supplementary material for: Targeting Aquaporin Function: Potent Inhibition of Aquaglyceroporin-3 by a Gold-Based Compound
Source: PLoS One. 2012 May 18;7(5):e37435. doi: 10.1371/journal.pone.0037435 (PMC3356263; doi:10.1371/journal.pone.0037435)
Supplement: Table S2 — Docking score of Auphen and Audien at periplasmic pocket of AQP1 and AQP3. (DOC) [file pone.0037435.s006.doc]

**Table S2.** Docking score of Auphen and Audien at periplasmic pocket of AQP1 and AQP3.

| Complex | Y | AQP1 | | AQP3 | |
| --- | --- | --- | --- | --- | --- |
|  |  | top | ave | top | ave |
| Auphen | Cl | -6.13 | -4.45 | -6.47 | -4.93 |
|  | OH | -6.64 | -5.25 | -7.77 | -6.24 |
| Audien | Cl | -7.40 | -5.68 | -7.42 | -5.37 |
|  | OH | -7.37 | -5.64 | -7.69 | -6.56 |
