# Supplementary material for: Targeting Aquaporin Function: Potent Inhibition of Aquaglyceroporin-3 by a Gold-Based Compound
Source: PLoS One. 2012 May 18;7(5):e37435. doi: 10.1371/journal.pone.0037435 (PMC3356263; doi:10.1371/journal.pone.0037435)
Supplement: Dataset S1 — Calculated homology modelling structures (pdb format) of AQP3. Available at http://www.ff.ul.pt/fct/dataset_s1.pdf. (PDF) [file pone.0037435.s007.pdf]

Dataset S1 – Calculated homology modelling structures (pdb format) of AQP3

```
REMARK 888
REMARK 888 WRITTEN BY MAESTRO (A PRODUCT OF SCHRODINGER, LLC)
TITLE     hmod1
HELIX    1   1 LEU     21  LEU     48  1
HELIX    2   2 PHE     56  SER     78  1
HELIX    3   3 VAL     86  LEU     93  1
HELIX    4   4 LEU    101  LEU    122  1
HELIX    5   5 TYR    124  ASN    133  1
HELIX    6   6 MET    157  VAL    179  1
HELIX    7   7 LEU    190  SER    210  1
HELIX    8   8 PHE    220  LEU    228  1
HELIX    9   9 VAL    245  MET    264  1
TURN     1   1 ARG    20  ARG    20
TURN     2   2 SER    49  GLY    55
TURN     3   3 GLY    79  ALA    85
TURN     4   4 ALA    94  LYS   100
TURN     5   5 TYR   123  TYR   123
TURN     6   6 GLN   134  ASP   156
TURN     7   7 ASP   180  GLY   189
TURN     8   8 GLY   211  ASP   219
TURN     9   9 ALA   229  TRP   244
TURN    10  10 ILE   265  LEU   269
SEQRES   1     250  ARG LEU LEU ARG GLN ALA LEU ALA GLU CYS LEU GLY THR
SEQRES   2     250  LEU ILE LEU VAL MET PHE GLY CYS GLY SER VAL ALA GLN
SEQRES   3     250  VAL VAL LEU SER ARG GLY THR HIS GLY GLY PHE LEU THR
SEQRES   4     250  ILE ASN LEU ALA PHE GLY PHE ALA VAL THR LEU GLY ILE
SEQRES   5     250  LEU ILE ALA GLY GLN VAL SER GLY ALA HIS LEU ASN PRO
SEQRES   6     250  ALA VAL THR PHE ALA MET CYS PHE LEU ALA ARG GLU PRO
SEQRES   7     250  TRP ILE LYS LEU PRO ILE TYR THR LEU ALA GLN THR LEU
SEQRES   8     250  GLY ALA PHE LEU GLY ALA GLY ILE VAL PHE GLY LEU TYR
SEQRES   9     250  TYR ASP ALA ILE TRP HIS PHE ALA ASP ASN GLN LEU PHE
SEQRES  10     250  VAL SER GLY PRO ASN GLY THR ALA GLY ILE PHE ALA THR
SEQRES  11     250  TYR PRO SER GLY HIS LEU ASP MET ILE ASN GLY PHE PHE
SEQRES  12     250  ASP GLN PHE ILE GLY THR ALA SER LEU ILE VAL CYS VAL
SEQRES  13     250  LEU ALA ILE VAL ASP PRO TYR ASN ASN PRO VAL PRO ARG
SEQRES  14     250  GLY LEU GLU ALA PHE THR VAL GLY LEU VAL VAL LEU VAL
SEQRES  15     250  ILE GLY THR SER MET GLY PHE ASN SER GLY TYR ALA VAL
SEQRES  16     250  ASN PRO ALA ARG ASP PHE GLY PRO ARG LEU PHE THR ALA
SEQRES  17     250  LEU ALA GLY TRP GLY SER ALA VAL PHE THR THR GLY GLN
SEQRES  18     250  HIS TRP TRP TRP VAL PRO ILE VAL SER PRO LEU LEU GLY
SEQRES  19     250  SER ILE ALA GLY VAL PHE VAL TYR GLN LEU MET ILE GLY
SEQRES  20     250  CYS HIS LEU
MODEL        1
ATOM      1  N   ARG    20      -5.968  78.245 -15.851  1.00  4.39           N1+
ATOM      2  CA  ARG    20      -6.071  79.699 -15.611  1.00  4.39           C
ATOM      3  C   ARG    20      -6.594  79.913 -14.183  1.00  4.39           C
ATOM      4  O   ARG    20      -7.372  79.091 -13.702  1.00  4.39           O
ATOM      5  CB  ARG    20      -6.858  80.460 -16.716  1.00  4.39           C
ATOM      6  CG  ARG    20      -8.362  80.149 -16.916  1.00  4.39           C
ATOM      7  CD  ARG    20      -8.966  80.986 -18.071  1.00  4.39           C
ATOM      8  NE  ARG    20     -10.391  80.685 -18.328  1.00  4.39           N
ATOM      9  CZ  ARG    20     -11.069  80.925 -19.467  1.00  4.39           C
ATOM     10  NH1 ARG    20     -10.469  81.472 -20.529  1.00  4.39           N
ATOM     11  NH2 ARG    20     -12.366  80.615 -19.545  1.00  4.39           N1+
ATOM     12  H1  ARG    20      -5.595  78.003 -16.755  1.00  0.00           H
ATOM     13  H2  ARG    20      -5.322  77.883 -15.134  1.00  0.00           H
ATOM     14  H3  ARG    20      -6.853  77.802 -15.660  1.00  0.00           H
ATOM     15  HA  ARG    20      -5.042  80.066 -15.632  1.00  0.00           H
```

ATOM	16	HB2	ARG	20	-6.762	81.529	-16.514	1.00	0.00	H
ATOM	17	HB3	ARG	20	-6.341	80.312	-17.666	1.00	0.00	H
ATOM	18	HG2	ARG	20	-8.498	79.088	-17.129	1.00	0.00	H
ATOM	19	HG3	ARG	20	-8.907	80.354	-15.993	1.00	0.00	H
ATOM	20	HD2	ARG	20	-8.871	82.054	-17.861	1.00	0.00	H
ATOM	21	HD3	ARG	20	-8.399	80.793	-18.982	1.00	0.00	H
ATOM	22	HE	ARG	20	-10.912	80.377	-17.516	1.00	0.00	H
ATOM	23	HH12	ARG	20	-10.958	81.663	-21.397	1.00	0.00	H
ATOM	24	HH11	ARG	20	-9.501	81.758	-20.510	1.00	0.00	H
ATOM	25	HH22	ARG	20	-12.913	80.796	-20.380	1.00	0.00	H
ATOM	26	HH21	ARG	20	-12.889	80.205	-18.781	1.00	0.00	H
ATOM	27	N	LEU	21	-6.093	80.947	-13.497	1.00	3.68	N
ATOM	28	CA	LEU	21	-5.580	80.808	-12.128	1.00	3.68	C
ATOM	29	C	LEU	21	-6.594	80.784	-11.010	1.00	3.68	C
ATOM	30	O	LEU	21	-6.351	80.105	-10.020	1.00	3.68	O
ATOM	31	CB	LEU	21	-4.586	81.977	-11.914	1.00	3.68	C
ATOM	32	CG	LEU	21	-3.536	81.782	-10.799	1.00	3.68	C
ATOM	33	CD1	LEU	21	-2.623	80.570	-11.040	1.00	3.68	C
ATOM	34	CD2	LEU	21	-2.673	83.044	-10.677	1.00	3.68	C
ATOM	35	H	LEU	21	-5.608	81.666	-14.014	1.00	0.00	H
ATOM	36	HA	LEU	21	-5.040	79.862	-12.091	1.00	0.00	H
ATOM	37	HB2	LEU	21	-4.031	82.162	-12.835	1.00	0.00	H
ATOM	38	HB3	LEU	21	-5.141	82.898	-11.722	1.00	0.00	H
ATOM	39	HG	LEU	21	-4.046	81.640	-9.844	1.00	0.00	H
ATOM	40	HD11	LEU	21	-1.857	80.509	-10.266	1.00	0.00	H
ATOM	41	HD12	LEU	21	-3.164	79.629	-11.012	1.00	0.00	H
ATOM	42	HD13	LEU	21	-2.114	80.639	-12.001	1.00	0.00	H
ATOM	43	HD21	LEU	21	-1.975	82.963	-9.844	1.00	0.00	H
ATOM	44	HD22	LEU	21	-2.080	83.226	-11.574	1.00	0.00	H
ATOM	45	HD23	LEU	21	-3.287	83.927	-10.502	1.00	0.00	H
ATOM	46	N	LEU	22	-7.740	81.442	-11.184	1.00	3.40	N
ATOM	47	CA	LEU	22	-8.771	81.493	-10.155	1.00	3.40	C
ATOM	48	C	LEU	22	-9.294	80.101	-9.777	1.00	3.40	C
ATOM	49	O	LEU	22	-9.557	79.839	-8.609	1.00	3.40	O
ATOM	50	CB	LEU	22	-9.912	82.422	-10.636	1.00	3.40	C
ATOM	51	CG	LEU	22	-11.071	82.659	-9.636	1.00	3.40	C
ATOM	52	CD1	LEU	22	-10.598	83.304	-8.323	1.00	3.40	C
ATOM	53	CD2	LEU	22	-12.174	83.513	-10.280	1.00	3.40	C
ATOM	54	H	LEU	22	-7.850	82.019	-12.000	1.00	0.00	H
ATOM	55	HA	LEU	22	-8.316	81.925	-9.260	1.00	0.00	H
ATOM	56	HB2	LEU	22	-9.489	83.393	-10.896	1.00	0.00	H
ATOM	57	HB3	LEU	22	-10.331	82.020	-11.559	1.00	0.00	H
ATOM	58	HG	LEU	22	-11.529	81.701	-9.382	1.00	0.00	H
ATOM	59	HD11	LEU	22	-11.441	83.503	-7.659	1.00	0.00	H
ATOM	60	HD12	LEU	22	-9.919	82.654	-7.769	1.00	0.00	H
ATOM	61	HD13	LEU	22	-10.089	84.253	-8.496	1.00	0.00	H
ATOM	62	HD21	LEU	22	-13.013	83.649	-9.596	1.00	0.00	H
ATOM	63	HD22	LEU	22	-11.808	84.506	-10.546	1.00	0.00	H
ATOM	64	HD23	LEU	22	-12.568	83.047	-11.183	1.00	0.00	H
ATOM	65	N	ARG	23	-9.343	79.172	-10.745	1.00	2.83	N
ATOM	66	CA	ARG	23	-9.713	77.799	-10.437	1.00	2.83	C
ATOM	67	C	ARG	23	-8.604	77.040	-9.694	1.00	2.83	C
ATOM	68	O	ARG	23	-8.883	76.121	-8.931	1.00	2.83	O
ATOM	69	CB	ARG	23	-10.078	76.987	-11.724	1.00	2.83	C
ATOM	70	CG	ARG	23	-10.773	75.617	-11.434	1.00	2.83	C
ATOM	71	CD	ARG	23	-10.910	74.635	-12.623	1.00	2.83	C
ATOM	72	NE	ARG	23	-11.439	73.322	-12.178	1.00	2.83	N
ATOM	73	CZ	ARG	23	-11.073	72.076	-12.543	1.00	2.83	C
ATOM	74	NH1	ARG	23	-10.117	71.842	-13.444	1.00	2.83	N
ATOM	75	NH2	ARG	23	-11.669	71.014	-12.001	1.00	2.83	N1+
ATOM	76	H	ARG	23	-9.028	79.417	-11.669	1.00	0.00	H
ATOM	77	HA	ARG	23	-10.593	77.816	-9.789	1.00	0.00	H
ATOM	78	HB2	ARG	23	-10.743	77.582	-12.351	1.00	0.00	H

ATOM	79	HB3	ARG	23	-9.174	76.819	-12.311	1.00	0.00	H
ATOM	80	HG2	ARG	23	-10.244	75.078	-10.652	1.00	0.00	H
ATOM	81	HG3	ARG	23	-11.760	75.817	-11.014	1.00	0.00	H
ATOM	82	HD2	ARG	23	-11.574	75.047	-13.384	1.00	0.00	H
ATOM	83	HD3	ARG	23	-9.937	74.498	-13.094	1.00	0.00	H
ATOM	84	HE	ARG	23	-12.189	73.404	-11.500	1.00	0.00	H
ATOM	85	HH12	ARG	23	-9.573	72.565	-13.881	1.00	0.00	H
ATOM	86	HH11	ARG	23	-9.836	70.875	-13.624	1.00	0.00	H
ATOM	87	HH22	ARG	23	-11.339	70.073	-12.253	1.00	0.00	H
ATOM	88	HH21	ARG	23	-12.374	71.050	-11.277	1.00	0.00	H
ATOM	89	N	GLN	24	-7.350	77.404	-9.949	1.00	2.43	N
ATOM	90	CA	GLN	24	-6.190	76.704	-9.425	1.00	2.43	C
ATOM	91	C	GLN	24	-5.934	77.153	-7.989	1.00	2.43	C
ATOM	92	O	GLN	24	-5.632	76.318	-7.150	1.00	2.43	O
ATOM	93	CB	GLN	24	-4.968	77.011	-10.314	1.00	2.43	C
ATOM	94	CG	GLN	24	-5.255	76.730	-11.808	1.00	2.43	C
ATOM	95	CD	GLN	24	-4.105	77.020	-12.765	1.00	2.43	C
ATOM	96	OE1	GLN	24	-4.357	77.433	-13.901	1.00	2.43	O
ATOM	97	NE2	GLN	24	-2.858	76.878	-12.342	1.00	2.43	N
ATOM	98	H	GLN	24	-7.194	78.302	-10.383	1.00	0.00	H
ATOM	99	HA	GLN	24	-6.370	75.627	-9.423	1.00	0.00	H
ATOM	100	HB2	GLN	24	-4.654	78.046	-10.182	1.00	0.00	H
ATOM	101	HB3	GLN	24	-4.136	76.398	-9.967	1.00	0.00	H
ATOM	102	HG2	GLN	24	-5.534	75.683	-11.928	1.00	0.00	H
ATOM	103	HG3	GLN	24	-6.109	77.311	-12.150	1.00	0.00	H
ATOM	104	HE22	GLN	24	-2.096	76.994	-12.988	1.00	0.00	H
ATOM	105	HE21	GLN	24	-2.644	76.509	-11.411	1.00	0.00	H
ATOM	106	N	ALA	25	-6.175	78.438	-7.697	1.00	2.11	N
ATOM	107	CA	ALA	25	-6.202	78.984	-6.349	1.00	2.11	C
ATOM	108	C	ALA	25	-7.248	78.292	-5.475	1.00	2.11	C
ATOM	109	O	ALA	25	-6.928	77.833	-4.387	1.00	2.11	O
ATOM	110	CB	ALA	25	-6.472	80.495	-6.422	1.00	2.11	C
ATOM	111	H	ALA	25	-6.390	79.071	-8.457	1.00	0.00	H
ATOM	112	HA	ALA	25	-5.221	78.824	-5.893	1.00	0.00	H
ATOM	113	HB1	ALA	25	-6.450	80.938	-5.424	1.00	0.00	H
ATOM	114	HB2	ALA	25	-5.714	81.002	-7.016	1.00	0.00	H
ATOM	115	HB3	ALA	25	-7.447	80.716	-6.858	1.00	0.00	H
ATOM	116	N	LEU	26	-8.475	78.139	-5.987	1.00	2.17	N
ATOM	117	CA	LEU	26	-9.547	77.423	-5.299	1.00	2.17	C
ATOM	118	C	LEU	26	-9.226	75.952	-5.016	1.00	2.17	C
ATOM	119	O	LEU	26	-9.557	75.448	-3.946	1.00	2.17	O
ATOM	120	CB	LEU	26	-10.869	77.565	-6.095	1.00	2.17	C
ATOM	121	CG	LEU	26	-11.813	78.667	-5.562	1.00	2.17	C
ATOM	122	CD1	LEU	26	-11.209	80.081	-5.617	1.00	2.17	C
ATOM	123	CD2	LEU	26	-13.161	78.635	-6.298	1.00	2.17	C
ATOM	124	H	LEU	26	-8.689	78.578	-6.870	1.00	0.00	H
ATOM	125	HA	LEU	26	-9.675	77.886	-4.318	1.00	0.00	H
ATOM	126	HB2	LEU	26	-10.666	77.716	-7.156	1.00	0.00	H
ATOM	127	HB3	LEU	26	-11.421	76.624	-6.045	1.00	0.00	H
ATOM	128	HG	LEU	26	-12.015	78.444	-4.512	1.00	0.00	H
ATOM	129	HD11	LEU	26	-11.881	80.807	-5.158	1.00	0.00	H
ATOM	130	HD12	LEU	26	-10.265	80.143	-5.075	1.00	0.00	H
ATOM	131	HD13	LEU	26	-11.033	80.407	-6.640	1.00	0.00	H
ATOM	132	HD21	LEU	26	-13.850	79.372	-5.883	1.00	0.00	H
ATOM	133	HD22	LEU	26	-13.041	78.853	-7.359	1.00	0.00	H
ATOM	134	HD23	LEU	26	-13.639	77.659	-6.205	1.00	0.00	H
ATOM	135	N	ALA	27	-8.539	75.283	-5.946	1.00	2.10	N
ATOM	136	CA	ALA	27	-8.079	73.917	-5.751	1.00	2.10	C
ATOM	137	C	ALA	27	-6.982	73.814	-4.689	1.00	2.10	C
ATOM	138	O	ALA	27	-7.032	72.914	-3.854	1.00	2.10	O
ATOM	139	CB	ALA	27	-7.564	73.353	-7.081	1.00	2.10	C
ATOM	140	H	ALA	27	-8.285	75.762	-6.797	1.00	0.00	H
ATOM	141	HA	ALA	27	-8.921	73.305	-5.418	1.00	0.00	H

ATOM	142	HB1	ALA	27	-7.167	72.344	-6.954	1.00	0.00	H
ATOM	143	HB2	ALA	27	-8.370	73.297	-7.809	1.00	0.00	H
ATOM	144	HB3	ALA	27	-6.770	73.967	-7.498	1.00	0.00	H
ATOM	145	N	GLU	28	-6.040	74.763	-4.683	1.00	2.37	N
ATOM	146	CA	GLU	28	-4.983	74.832	-3.685	1.00	2.37	C
ATOM	147	C	GLU	28	-5.507	75.189	-2.295	1.00	2.37	C
ATOM	148	O	GLU	28	-5.024	74.629	-1.314	1.00	2.37	O
ATOM	149	CB	GLU	28	-3.891	75.825	-4.142	1.00	2.37	C
ATOM	150	CG	GLU	28	-3.081	75.361	-5.380	1.00	2.37	C
ATOM	151	CD	GLU	28	-2.096	74.222	-5.122	1.00	2.37	C
ATOM	152	OE1	GLU	28	-1.333	74.313	-4.139	1.00	2.37	O
ATOM	153	OE2	GLU	28	-2.074	73.271	-5.937	1.00	2.37	O1-
ATOM	154	H	GLU	28	-6.025	75.450	-5.427	1.00	0.00	H
ATOM	155	HA	GLU	28	-4.528	73.842	-3.600	1.00	0.00	H
ATOM	156	HB2	GLU	28	-4.337	76.798	-4.346	1.00	0.00	H
ATOM	157	HB3	GLU	28	-3.194	75.992	-3.321	1.00	0.00	H
ATOM	158	HG2	GLU	28	-3.749	75.055	-6.179	1.00	0.00	H
ATOM	159	HG3	GLU	28	-2.504	76.202	-5.761	1.00	0.00	H
ATOM	160	N	CYS	29	-6.560	76.015	-2.211	1.00	2.57	N
ATOM	161	CA	CYS	29	-7.273	76.262	-0.963	1.00	2.57	C
ATOM	162	C	CYS	29	-7.878	74.966	-0.415	1.00	2.57	C
ATOM	163	O	CYS	29	-7.590	74.573	0.712	1.00	2.57	O
ATOM	164	CB	CYS	29	-8.433	77.278	-1.134	1.00	2.57	C
ATOM	165	SG	CYS	29	-7.921	78.924	-1.691	1.00	2.57	S
ATOM	166	H	CYS	29	-6.870	76.480	-3.056	1.00	0.00	H
ATOM	167	HA	CYS	29	-6.568	76.638	-0.222	1.00	0.00	H
ATOM	168	HB2	CYS	29	-9.181	76.908	-1.834	1.00	0.00	H
ATOM	169	HB3	CYS	29	-8.946	77.407	-0.179	1.00	0.00	H
ATOM	170	HG	CYS	29	-7.324	78.498	-2.815	1.00	0.00	H
ATOM	171	N	LEU	30	-8.680	74.281	-1.238	1.00	2.68	N
ATOM	172	CA	LEU	30	-9.423	73.095	-0.835	1.00	2.68	C
ATOM	173	C	LEU	30	-8.531	71.873	-0.590	1.00	2.68	C
ATOM	174	O	LEU	30	-8.855	71.045	0.257	1.00	2.68	O
ATOM	175	CB	LEU	30	-10.455	72.745	-1.932	1.00	2.68	C
ATOM	176	CG	LEU	30	-11.632	73.740	-2.050	1.00	2.68	C
ATOM	177	CD1	LEU	30	-12.379	73.541	-3.378	1.00	2.68	C
ATOM	178	CD2	LEU	30	-12.609	73.628	-0.867	1.00	2.68	C
ATOM	179	H	LEU	30	-8.834	74.632	-2.176	1.00	0.00	H
ATOM	180	HA	LEU	30	-9.941	73.303	0.102	1.00	0.00	H
ATOM	181	HB2	LEU	30	-9.932	72.675	-2.888	1.00	0.00	H
ATOM	182	HB3	LEU	30	-10.861	71.747	-1.753	1.00	0.00	H
ATOM	183	HG	LEU	30	-11.235	74.755	-2.054	1.00	0.00	H
ATOM	184	HD11	LEU	30	-13.189	74.263	-3.485	1.00	0.00	H
ATOM	185	HD12	LEU	30	-11.710	73.671	-4.228	1.00	0.00	H
ATOM	186	HD13	LEU	30	-12.812	72.542	-3.443	1.00	0.00	H
ATOM	187	HD21	LEU	30	-13.442	74.323	-0.977	1.00	0.00	H
ATOM	188	HD22	LEU	30	-13.026	72.624	-0.786	1.00	0.00	H
ATOM	189	HD23	LEU	30	-12.128	73.861	0.083	1.00	0.00	H
ATOM	190	N	GLY	31	-7.380	71.790	-1.260	1.00	2.47	N
ATOM	191	CA	GLY	31	-6.483	70.655	-1.131	1.00	2.47	C
ATOM	192	C	GLY	31	-5.545	70.820	0.061	1.00	2.47	C
ATOM	193	O	GLY	31	-5.216	69.833	0.714	1.00	2.47	O
ATOM	194	H	GLY	31	-7.191	72.460	-2.000	1.00	0.00	H
ATOM	195	HA2	GLY	31	-7.036	69.719	-1.061	1.00	0.00	H
ATOM	196	HA3	GLY	31	-5.886	70.578	-2.039	1.00	0.00	H
ATOM	197	N	THR	32	-5.190	72.062	0.415	1.00	2.29	N
ATOM	198	CA	THR	32	-4.418	72.315	1.627	1.00	2.29	C
ATOM	199	C	THR	32	-5.329	72.308	2.861	1.00	2.29	C
ATOM	200	O	THR	32	-4.901	71.918	3.945	1.00	2.29	O
ATOM	201	CB	THR	32	-3.694	73.683	1.541	1.00	2.29	C
ATOM	202	OG1	THR	32	-3.047	73.815	0.291	1.00	2.29	O
ATOM	203	CG2	THR	32	-2.583	73.828	2.594	1.00	2.29	C
ATOM	204	H	THR	32	-5.477	72.851	-0.146	1.00	0.00	H

ATOM	205	HA	THR	32	-3.661	71.539	1.741	1.00	0.00	H
ATOM	206	HB	THR	32	-4.405	74.506	1.644	1.00	0.00	H
ATOM	207	HG1	THR	32	-3.714	74.016	-0.366	1.00	0.00	H
ATOM	208	HG21	THR	32	-2.041	74.765	2.477	1.00	0.00	H
ATOM	209	HG22	THR	32	-2.987	73.805	3.604	1.00	0.00	H
ATOM	210	HG23	THR	32	-1.856	73.019	2.516	1.00	0.00	H
ATOM	211	N	LEU	33	-6.604	72.673	2.691	1.00	1.96	N
ATOM	212	CA	LEU	33	-7.621	72.542	3.718	1.00	1.96	C
ATOM	213	C	LEU	33	-7.881	71.065	4.038	1.00	1.96	C
ATOM	214	O	LEU	33	-8.000	70.712	5.211	1.00	1.96	O
ATOM	215	CB	LEU	33	-8.923	73.219	3.218	1.00	1.96	C
ATOM	216	CG	LEU	33	-10.131	73.194	4.189	1.00	1.96	C
ATOM	217	CD1	LEU	33	-10.859	74.547	4.215	1.00	1.96	C
ATOM	218	CD2	LEU	33	-11.131	72.070	3.861	1.00	1.96	C
ATOM	219	H	LEU	33	-6.891	73.041	1.792	1.00	0.00	H
ATOM	220	HA	LEU	33	-7.306	73.076	4.607	1.00	0.00	H
ATOM	221	HB2	LEU	33	-8.677	74.260	3.012	1.00	0.00	H
ATOM	222	HB3	LEU	33	-9.222	72.799	2.259	1.00	0.00	H
ATOM	223	HG	LEU	33	-9.757	73.023	5.198	1.00	0.00	H
ATOM	224	HD11	LEU	33	-11.674	74.542	4.940	1.00	0.00	H
ATOM	225	HD12	LEU	33	-10.182	75.350	4.504	1.00	0.00	H
ATOM	226	HD13	LEU	33	-11.277	74.796	3.240	1.00	0.00	H
ATOM	227	HD21	LEU	33	-11.970	72.080	4.557	1.00	0.00	H
ATOM	228	HD22	LEU	33	-11.536	72.177	2.854	1.00	0.00	H
ATOM	229	HD23	LEU	33	-10.676	71.082	3.929	1.00	0.00	H
ATOM	230	N	ILE	34	-7.980	70.197	3.016	1.00	1.68	N
ATOM	231	CA	ILE	34	-8.377	68.814	3.247	1.00	1.68	C
ATOM	232	C	ILE	34	-7.314	67.986	3.974	1.00	1.68	C
ATOM	233	O	ILE	34	-7.659	67.168	4.829	1.00	1.68	O
ATOM	234	CB	ILE	34	-8.944	68.125	1.970	1.00	1.68	C
ATOM	235	CG1	ILE	34	-9.904	66.953	2.289	1.00	1.68	C
ATOM	236	CG2	ILE	34	-7.871	67.614	1.002	1.00	1.68	C
ATOM	237	CD1	ILE	34	-11.212	67.355	2.988	1.00	1.68	C
ATOM	238	H	ILE	34	-7.937	70.536	2.062	1.00	0.00	H
ATOM	239	HA	ILE	34	-9.208	68.879	3.948	1.00	0.00	H
ATOM	240	HB	ILE	34	-9.537	68.864	1.431	1.00	0.00	H
ATOM	241	HG12	ILE	34	-10.174	66.455	1.356	1.00	0.00	H
ATOM	242	HG13	ILE	34	-9.387	66.199	2.883	1.00	0.00	H
ATOM	243	HG21	ILE	34	-8.331	67.237	0.091	1.00	0.00	H
ATOM	244	HG22	ILE	34	-7.188	68.406	0.726	1.00	0.00	H
ATOM	245	HG23	ILE	34	-7.283	66.797	1.425	1.00	0.00	H
ATOM	246	HD11	ILE	34	-11.895	66.507	3.036	1.00	0.00	H
ATOM	247	HD12	ILE	34	-11.047	67.682	4.015	1.00	0.00	H
ATOM	248	HD13	ILE	34	-11.720	68.157	2.452	1.00	0.00	H
ATOM	249	N	LEU	35	-6.029	68.246	3.708	1.00	1.50	N
ATOM	250	CA	LEU	35	-4.969	67.612	4.474	1.00	1.50	C
ATOM	251	C	LEU	35	-4.892	68.153	5.905	1.00	1.50	C
ATOM	252	O	LEU	35	-4.552	67.379	6.801	1.00	1.50	O
ATOM	253	CB	LEU	35	-3.606	67.696	3.735	1.00	1.50	C
ATOM	254	CG	LEU	35	-2.992	69.090	3.456	1.00	1.50	C
ATOM	255	CD1	LEU	35	-2.077	69.589	4.590	1.00	1.50	C
ATOM	256	CD2	LEU	35	-2.205	69.103	2.134	1.00	1.50	C
ATOM	257	H	LEU	35	-5.794	68.920	2.993	1.00	0.00	H
ATOM	258	HA	LEU	35	-5.203	66.549	4.552	1.00	0.00	H
ATOM	259	HB2	LEU	35	-2.881	67.088	4.276	1.00	0.00	H
ATOM	260	HB3	LEU	35	-3.744	67.179	2.784	1.00	0.00	H
ATOM	261	HG	LEU	35	-3.811	69.792	3.336	1.00	0.00	H
ATOM	262	HD11	LEU	35	-1.693	70.585	4.368	1.00	0.00	H
ATOM	263	HD12	LEU	35	-2.594	69.658	5.545	1.00	0.00	H
ATOM	264	HD13	LEU	35	-1.221	68.928	4.729	1.00	0.00	H
ATOM	265	HD21	LEU	35	-1.810	70.097	1.921	1.00	0.00	H
ATOM	266	HD22	LEU	35	-1.356	68.422	2.156	1.00	0.00	H
ATOM	267	HD23	LEU	35	-2.834	68.816	1.291	1.00	0.00	H

ATOM	268	N	VAL	36	-5.251	69.430	6.153	1.00	1.54	N
ATOM	269	CA	VAL	36	-5.310	69.916	7.527	1.00	1.54	C
ATOM	270	C	VAL	36	-6.495	69.385	8.325	1.00	1.54	C
ATOM	271	O	VAL	36	-6.328	69.076	9.501	1.00	1.54	O
ATOM	272	CB	VAL	36	-5.121	71.438	7.735	1.00	1.54	C
ATOM	273	CG1	VAL	36	-3.748	71.934	7.253	1.00	1.54	C
ATOM	274	CG2	VAL	36	-6.252	72.315	7.216	1.00	1.54	C
ATOM	275	H	VAL	36	-5.532	70.042	5.399	1.00	0.00	H
ATOM	276	HA	VAL	36	-4.443	69.490	8.038	1.00	0.00	H
ATOM	277	HB	VAL	36	-5.122	71.586	8.817	1.00	0.00	H
ATOM	278	HG11	VAL	36	-3.645	71.862	6.171	1.00	0.00	H
ATOM	279	HG12	VAL	36	-3.595	72.974	7.538	1.00	0.00	H
ATOM	280	HG13	VAL	36	-2.942	71.352	7.702	1.00	0.00	H
ATOM	281	HG21	VAL	36	-6.233	73.293	7.692	1.00	0.00	H
ATOM	282	HG22	VAL	36	-6.135	72.443	6.150	1.00	0.00	H
ATOM	283	HG23	VAL	36	-7.233	71.890	7.418	1.00	0.00	H
ATOM	284	N	MET	37	-7.646	69.199	7.680	1.00	1.69	N
ATOM	285	CA	MET	37	-8.841	68.626	8.285	1.00	1.69	C
ATOM	286	C	MET	37	-8.556	67.250	8.890	1.00	1.69	C
ATOM	287	O	MET	37	-8.858	67.003	10.054	1.00	1.69	O
ATOM	288	CB	MET	37	-9.932	68.525	7.187	1.00	1.69	C
ATOM	289	CG	MET	37	-11.286	67.932	7.625	1.00	1.69	C
ATOM	290	SD	MET	37	-12.397	69.083	8.472	1.00	1.69	S
ATOM	291	CE	MET	37	-13.111	69.944	7.046	1.00	1.69	C
ATOM	292	H	MET	37	-7.705	69.530	6.722	1.00	0.00	H
ATOM	293	HA	MET	37	-9.186	69.297	9.070	1.00	0.00	H
ATOM	294	HB2	MET	37	-10.097	69.505	6.739	1.00	0.00	H
ATOM	295	HB3	MET	37	-9.556	67.898	6.379	1.00	0.00	H
ATOM	296	HG2	MET	37	-11.812	67.556	6.747	1.00	0.00	H
ATOM	297	HG3	MET	37	-11.154	67.058	8.263	1.00	0.00	H
ATOM	298	HE1	MET	37	-13.874	70.648	7.378	1.00	0.00	H
ATOM	299	HE2	MET	37	-12.342	70.495	6.505	1.00	0.00	H
ATOM	300	HE3	MET	37	-13.580	69.234	6.365	1.00	0.00	H
ATOM	301	N	PHE	38	-7.925	66.377	8.104	1.00	1.85	N
ATOM	302	CA	PHE	38	-7.643	65.012	8.518	1.00	1.85	C
ATOM	303	C	PHE	38	-6.344	64.852	9.308	1.00	1.85	C
ATOM	304	O	PHE	38	-6.114	63.810	9.926	1.00	1.85	O
ATOM	305	CB	PHE	38	-7.634	64.113	7.273	1.00	1.85	C
ATOM	306	CG	PHE	38	-8.991	63.902	6.618	1.00	1.85	C
ATOM	307	CD1	PHE	38	-10.059	63.344	7.355	1.00	1.85	C
ATOM	308	CD2	PHE	38	-9.186	64.240	5.262	1.00	1.85	C
ATOM	309	CE1	PHE	38	-11.313	63.144	6.746	1.00	1.85	C
ATOM	310	CE2	PHE	38	-10.438	64.031	4.652	1.00	1.85	C
ATOM	311	CZ	PHE	38	-11.504	63.488	5.395	1.00	1.85	C
ATOM	312	H	PHE	38	-7.717	66.645	7.150	1.00	0.00	H
ATOM	313	HA	PHE	38	-8.432	64.660	9.185	1.00	0.00	H
ATOM	314	HB2	PHE	38	-6.966	64.559	6.545	1.00	0.00	H
ATOM	315	HB3	PHE	38	-7.237	63.126	7.518	1.00	0.00	H
ATOM	316	HD1	PHE	38	-9.925	63.063	8.389	1.00	0.00	H
ATOM	317	HD2	PHE	38	-8.376	64.656	4.679	1.00	0.00	H
ATOM	318	HE1	PHE	38	-12.128	62.719	7.315	1.00	0.00	H
ATOM	319	HE2	PHE	38	-10.581	64.282	3.612	1.00	0.00	H
ATOM	320	HZ	PHE	38	-12.464	63.327	4.926	1.00	0.00	H
ATOM	321	N	GLY	39	-5.547	65.913	9.383	1.00	1.97	N
ATOM	322	CA	GLY	39	-4.289	65.906	10.093	1.00	1.97	C
ATOM	323	C	GLY	39	-4.460	66.504	11.478	1.00	1.97	C
ATOM	324	O	GLY	39	-4.109	65.879	12.474	1.00	1.97	O
ATOM	325	H	GLY	39	-5.786	66.735	8.845	1.00	0.00	H
ATOM	326	HA2	GLY	39	-3.850	64.908	10.162	1.00	0.00	H
ATOM	327	HA3	GLY	39	-3.582	66.515	9.531	1.00	0.00	H
ATOM	328	N	CYS	40	-5.116	67.661	11.566	1.00	1.93	N
ATOM	329	CA	CYS	40	-5.480	68.278	12.834	1.00	1.93	C
ATOM	330	C	CYS	40	-6.672	67.551	13.481	1.00	1.93	C

ATOM	331	O	CYS	40	-6.867	67.638	14.691	1.00	1.93	O
ATOM	332	CB	CYS	40	-5.843	69.754	12.594	1.00	1.93	C
ATOM	333	SG	CYS	40	-4.481	70.624	11.737	1.00	1.93	S
ATOM	334	H	CYS	40	-5.429	68.137	10.722	1.00	0.00	H
ATOM	335	HA	CYS	40	-4.633	68.231	13.523	1.00	0.00	H
ATOM	336	HB2	CYS	40	-6.755	69.847	12.003	1.00	0.00	H
ATOM	337	HB3	CYS	40	-6.033	70.241	13.551	1.00	0.00	H
ATOM	338	HG	CYS	40	-5.142	71.775	11.592	1.00	0.00	H
ATOM	339	N	GLY	41	-7.425	66.764	12.703	1.00	1.89	N
ATOM	340	CA	GLY	41	-8.652	66.122	13.147	1.00	1.89	C
ATOM	341	C	GLY	41	-8.434	64.734	13.740	1.00	1.89	C
ATOM	342	O	GLY	41	-9.346	64.199	14.370	1.00	1.89	O
ATOM	343	H	GLY	41	-7.238	66.758	11.709	1.00	0.00	H
ATOM	344	HA2	GLY	41	-9.169	66.743	13.881	1.00	0.00	H
ATOM	345	HA3	GLY	41	-9.336	66.020	12.306	1.00	0.00	H
ATOM	346	N	SER	42	-7.226	64.182	13.601	1.00	1.80	N
ATOM	347	CA	SER	42	-6.774	63.005	14.330	1.00	1.80	C
ATOM	348	C	SER	42	-6.029	63.419	15.588	1.00	1.80	C
ATOM	349	O	SER	42	-6.280	62.870	16.658	1.00	1.80	O
ATOM	350	CB	SER	42	-5.902	62.153	13.379	1.00	1.80	C
ATOM	351	OG	SER	42	-4.918	62.912	12.691	1.00	1.80	O
ATOM	352	H	SER	42	-6.523	64.684	13.079	1.00	0.00	H
ATOM	353	HA	SER	42	-7.622	62.389	14.639	1.00	0.00	H
ATOM	354	HB2	SER	42	-5.417	61.352	13.938	1.00	0.00	H
ATOM	355	HB3	SER	42	-6.532	61.660	12.638	1.00	0.00	H
ATOM	356	HG	SER	42	-5.270	63.191	11.850	1.00	0.00	H
ATOM	357	N	VAL	43	-5.202	64.462	15.503	1.00	1.72	N
ATOM	358	CA	VAL	43	-4.526	65.040	16.651	1.00	1.72	C
ATOM	359	C	VAL	43	-5.491	65.540	17.734	1.00	1.72	C
ATOM	360	O	VAL	43	-5.275	65.300	18.921	1.00	1.72	O
ATOM	361	CB	VAL	43	-3.528	66.130	16.164	1.00	1.72	C
ATOM	362	CG1	VAL	43	-2.838	66.894	17.310	1.00	1.72	C
ATOM	363	CG2	VAL	43	-2.435	65.535	15.252	1.00	1.72	C
ATOM	364	H	VAL	43	-4.964	64.815	14.584	1.00	0.00	H
ATOM	365	HA	VAL	43	-3.939	64.242	17.112	1.00	0.00	H
ATOM	366	HB	VAL	43	-4.088	66.864	15.582	1.00	0.00	H
ATOM	367	HG11	VAL	43	-2.125	67.624	16.925	1.00	0.00	H
ATOM	368	HG12	VAL	43	-3.558	67.443	17.914	1.00	0.00	H
ATOM	369	HG13	VAL	43	-2.296	66.219	17.974	1.00	0.00	H
ATOM	370	HG21	VAL	43	-1.839	66.324	14.792	1.00	0.00	H
ATOM	371	HG22	VAL	43	-1.755	64.896	15.816	1.00	0.00	H
ATOM	372	HG23	VAL	43	-2.842	64.931	14.442	1.00	0.00	H
ATOM	373	N	ALA	44	-6.602	66.151	17.316	1.00	1.59	N
ATOM	374	CA	ALA	44	-7.650	66.598	18.216	1.00	1.59	C
ATOM	375	C	ALA	44	-8.456	65.465	18.855	1.00	1.59	C
ATOM	376	O	ALA	44	-9.049	65.646	19.918	1.00	1.59	O
ATOM	377	CB	ALA	44	-8.620	67.481	17.416	1.00	1.59	C
ATOM	378	H	ALA	44	-6.681	66.393	16.335	1.00	0.00	H
ATOM	379	HA	ALA	44	-7.213	67.217	18.997	1.00	0.00	H
ATOM	380	HB1	ALA	44	-9.432	67.844	18.048	1.00	0.00	H
ATOM	381	HB2	ALA	44	-8.113	68.357	17.011	1.00	0.00	H
ATOM	382	HB3	ALA	44	-9.068	66.937	16.583	1.00	0.00	H
ATOM	383	N	GLN	45	-8.464	64.292	18.215	1.00	1.69	N
ATOM	384	CA	GLN	45	-9.055	63.089	18.771	1.00	1.69	C
ATOM	385	C	GLN	45	-8.114	62.533	19.845	1.00	1.69	C
ATOM	386	O	GLN	45	-8.587	62.199	20.927	1.00	1.69	O
ATOM	387	CB	GLN	45	-9.370	62.085	17.621	1.00	1.69	C
ATOM	388	CG	GLN	45	-8.552	60.772	17.504	1.00	1.69	C
ATOM	389	CD	GLN	45	-8.792	59.984	16.215	1.00	1.69	C
ATOM	390	OE1	GLN	45	-8.016	59.097	15.886	1.00	1.69	O
ATOM	391	NE2	GLN	45	-9.818	60.309	15.436	1.00	1.69	N
ATOM	392	H	GLN	45	-7.881	64.200	17.396	1.00	0.00	H
ATOM	393	HA	GLN	45	-9.995	63.356	19.248	1.00	0.00	H

ATOM	394	HB2	GLN	45	-10.421	61.807	17.717	1.00	0.00	H
ATOM	395	HB3	GLN	45	-9.305	62.615	16.670	1.00	0.00	H
ATOM	396	HG2	GLN	45	-7.483	60.954	17.546	1.00	0.00	H
ATOM	397	HG3	GLN	45	-8.771	60.119	18.346	1.00	0.00	H
ATOM	398	HE22	GLN	45	-9.972	59.751	14.611	1.00	0.00	H
ATOM	399	HE21	GLN	45	-10.459	61.039	15.705	1.00	0.00	H
ATOM	400	N	VAL	46	-6.802	62.498	19.554	1.00	1.91	N
ATOM	401	CA	VAL	46	-5.736	62.106	20.468	1.00	1.91	C
ATOM	402	C	VAL	46	-5.730	62.938	21.749	1.00	1.91	C
ATOM	403	O	VAL	46	-5.755	62.378	22.844	1.00	1.91	O
ATOM	404	CB	VAL	46	-4.368	62.010	19.736	1.00	1.91	C
ATOM	405	CG1	VAL	46	-3.153	61.867	20.676	1.00	1.91	C
ATOM	406	CG2	VAL	46	-4.375	60.816	18.767	1.00	1.91	C
ATOM	407	H	VAL	46	-6.524	62.783	18.621	1.00	0.00	H
ATOM	408	HA	VAL	46	-5.984	61.095	20.792	1.00	0.00	H
ATOM	409	HB	VAL	46	-4.213	62.920	19.156	1.00	0.00	H
ATOM	410	HG11	VAL	46	-2.230	61.731	20.112	1.00	0.00	H
ATOM	411	HG12	VAL	46	-3.010	62.748	21.302	1.00	0.00	H
ATOM	412	HG13	VAL	46	-3.262	61.003	21.335	1.00	0.00	H
ATOM	413	HG21	VAL	46	-3.400	60.683	18.300	1.00	0.00	H
ATOM	414	HG22	VAL	46	-4.598	59.888	19.295	1.00	0.00	H
ATOM	415	HG23	VAL	46	-5.115	60.930	17.976	1.00	0.00	H
ATOM	416	N	VAL	47	-5.702	64.264	21.600	1.00	2.18	N
ATOM	417	CA	VAL	47	-5.408	65.144	22.717	1.00	2.18	C
ATOM	418	C	VAL	47	-6.617	65.399	23.641	1.00	2.18	C
ATOM	419	O	VAL	47	-6.392	65.752	24.792	1.00	2.18	O
ATOM	420	CB	VAL	47	-4.717	66.413	22.172	1.00	2.18	C
ATOM	421	CG1	VAL	47	-5.657	67.307	21.358	1.00	2.18	C
ATOM	422	CG2	VAL	47	-3.931	67.169	23.262	1.00	2.18	C
ATOM	423	H	VAL	47	-5.653	64.645	20.660	1.00	0.00	H
ATOM	424	HA	VAL	47	-4.670	64.637	23.344	1.00	0.00	H
ATOM	425	HB	VAL	47	-3.957	66.059	21.473	1.00	0.00	H
ATOM	426	HG11	VAL	47	-5.125	67.772	20.532	1.00	0.00	H
ATOM	427	HG12	VAL	47	-6.471	66.726	20.935	1.00	0.00	H
ATOM	428	HG13	VAL	47	-6.105	68.085	21.967	1.00	0.00	H
ATOM	429	HG21	VAL	47	-3.305	67.955	22.850	1.00	0.00	H
ATOM	430	HG22	VAL	47	-4.583	67.618	24.009	1.00	0.00	H
ATOM	431	HG23	VAL	47	-3.263	66.491	23.795	1.00	0.00	H
ATOM	432	N	LEU	48	-7.866	65.232	23.169	1.00	2.50	N
ATOM	433	CA	LEU	48	-9.009	65.960	23.737	1.00	2.50	C
ATOM	434	C	LEU	48	-10.348	65.233	23.786	1.00	2.50	C
ATOM	435	O	LEU	48	-11.280	65.775	24.375	1.00	2.50	O
ATOM	436	CB	LEU	48	-9.107	67.311	22.976	1.00	2.50	C
ATOM	437	CG	LEU	48	-8.771	68.608	23.741	1.00	2.50	C
ATOM	438	CD1	LEU	48	-7.515	68.611	24.618	1.00	2.50	C
ATOM	439	CD2	LEU	48	-8.618	69.731	22.723	1.00	2.50	C
ATOM	440	H	LEU	48	-7.952	64.875	22.230	1.00	0.00	H
ATOM	441	HA	LEU	48	-8.821	66.158	24.793	1.00	0.00	H
ATOM	442	HB2	LEU	48	-8.495	67.265	22.076	1.00	0.00	H
ATOM	443	HB3	LEU	48	-10.117	67.434	22.579	1.00	0.00	H
ATOM	444	HG	LEU	48	-9.617	68.820	24.396	1.00	0.00	H
ATOM	445	HD11	LEU	48	-7.415	69.554	25.155	1.00	0.00	H
ATOM	446	HD12	LEU	48	-7.549	67.833	25.370	1.00	0.00	H
ATOM	447	HD13	LEU	48	-6.609	68.469	24.041	1.00	0.00	H
ATOM	448	HD21	LEU	48	-8.430	70.678	23.229	1.00	0.00	H
ATOM	449	HD22	LEU	48	-7.786	69.561	22.043	1.00	0.00	H
ATOM	450	HD23	LEU	48	-9.519	69.803	22.119	1.00	0.00	H
ATOM	451	N	SER	49	-10.511	64.076	23.137	1.00	3.16	N
ATOM	452	CA	SER	49	-11.842	63.561	22.841	1.00	3.16	C
ATOM	453	C	SER	49	-11.952	62.059	23.090	1.00	3.16	C
ATOM	454	O	SER	49	-10.986	61.303	23.001	1.00	3.16	O
ATOM	455	CB	SER	49	-12.267	64.033	21.430	1.00	3.16	C
ATOM	456	OG	SER	49	-13.624	63.761	21.078	1.00	3.16	O

ATOM	457	H	SER	49	-9.709	63.577	22.773	1.00	0.00	H
ATOM	458	HA	SER	49	-12.562	63.997	23.537	1.00	0.00	H
ATOM	459	HB2	SER	49	-12.096	65.106	21.329	1.00	0.00	H
ATOM	460	HB3	SER	49	-11.623	63.546	20.701	1.00	0.00	H
ATOM	461	HG	SER	49	-13.677	63.822	20.130	1.00	0.00	H
ATOM	462	N	ARG	50	-13.200	61.643	23.318	1.00	3.91	N
ATOM	463	CA	ARG	50	-13.781	60.307	23.455	1.00	3.91	C
ATOM	464	C	ARG	50	-13.520	59.328	22.278	1.00	3.91	C
ATOM	465	O	ARG	50	-14.257	58.361	22.112	1.00	3.91	O
ATOM	466	CB	ARG	50	-15.295	60.500	23.762	1.00	3.91	C
ATOM	467	CG	ARG	50	-16.036	61.533	22.869	1.00	3.91	C
ATOM	468	CD	ARG	50	-16.180	62.938	23.509	1.00	3.91	C
ATOM	469	NE	ARG	50	-16.060	64.030	22.521	1.00	3.91	N
ATOM	470	CZ	ARG	50	-16.696	65.219	22.555	1.00	3.91	C
ATOM	471	NH1	ARG	50	-17.500	65.554	23.559	1.00	3.91	N
ATOM	472	NH2	ARG	50	-16.524	66.103	21.572	1.00	3.91	N1+
ATOM	473	H	ARG	50	-13.872	62.392	23.307	1.00	0.00	H
ATOM	474	HA	ARG	50	-13.317	59.838	24.324	1.00	0.00	H
ATOM	475	HB2	ARG	50	-15.806	59.538	23.682	1.00	0.00	H
ATOM	476	HB3	ARG	50	-15.418	60.772	24.811	1.00	0.00	H
ATOM	477	HG2	ARG	50	-15.576	61.580	21.881	1.00	0.00	H
ATOM	478	HG3	ARG	50	-17.044	61.154	22.687	1.00	0.00	H
ATOM	479	HD2	ARG	50	-17.139	62.995	24.025	1.00	0.00	H
ATOM	480	HD3	ARG	50	-15.418	63.107	24.271	1.00	0.00	H
ATOM	481	HE	ARG	50	-15.277	63.925	21.868	1.00	0.00	H
ATOM	482	HH12	ARG	50	-17.964	66.514	23.539	1.00	0.00	H
ATOM	483	HH11	ARG	50	-17.691	65.000	24.373	1.00	0.00	H
ATOM	484	HH22	ARG	50	-16.894	67.085	21.687	1.00	0.00	H
ATOM	485	HH21	ARG	50	-16.035	65.927	20.715	1.00	0.00	H
ATOM	486	N	GLY	51	-12.503	59.582	21.449	1.00	4.28	N
ATOM	487	CA	GLY	51	-12.037	58.732	20.360	1.00	4.28	C
ATOM	488	C	GLY	51	-10.514	58.558	20.343	1.00	4.28	C
ATOM	489	O	GLY	51	-9.989	58.112	19.327	1.00	4.28	O
ATOM	490	H	GLY	51	-11.897	60.349	21.723	1.00	0.00	H
ATOM	491	HA2	GLY	51	-12.483	57.737	20.413	1.00	0.00	H
ATOM	492	HA3	GLY	51	-12.354	59.166	19.412	1.00	0.00	H
ATOM	493	N	THR	52	-9.792	58.963	21.401	1.00	4.60	N
ATOM	494	CA	THR	52	-8.333	58.872	21.523	1.00	4.60	C
ATOM	495	C	THR	52	-7.842	57.409	21.379	1.00	4.60	C
ATOM	496	O	THR	52	-8.149	56.557	22.216	1.00	4.60	O
ATOM	497	CB	THR	52	-7.882	59.584	22.835	1.00	4.60	C
ATOM	498	OG1	THR	52	-6.471	59.659	22.900	1.00	4.60	O
ATOM	499	CG2	THR	52	-8.395	58.979	24.153	1.00	4.60	C
ATOM	500	H	THR	52	-10.273	59.351	22.200	1.00	0.00	H
ATOM	501	HA	THR	52	-7.919	59.444	20.692	1.00	0.00	H
ATOM	502	HB	THR	52	-8.238	60.612	22.801	1.00	0.00	H
ATOM	503	HG1	THR	52	-6.228	60.549	23.156	1.00	0.00	H
ATOM	504	HG21	THR	52	-8.116	59.607	24.999	1.00	0.00	H
ATOM	505	HG22	THR	52	-9.481	58.892	24.158	1.00	0.00	H
ATOM	506	HG23	THR	52	-7.978	57.989	24.337	1.00	0.00	H
ATOM	507	N	HIS	53	-7.151	57.107	20.269	1.00	5.03	N
ATOM	508	CA	HIS	53	-6.681	55.771	19.882	1.00	5.03	C
ATOM	509	C	HIS	53	-5.474	55.837	18.926	1.00	5.03	C
ATOM	510	O	HIS	53	-5.233	54.899	18.169	1.00	5.03	O
ATOM	511	CB	HIS	53	-7.860	54.893	19.369	1.00	5.03	C
ATOM	512	CG	HIS	53	-8.232	53.742	20.274	1.00	5.03	C
ATOM	513	ND1	HIS	53	-8.557	53.921	21.605	1.00	5.03	N
ATOM	514	CD2	HIS	53	-8.341	52.387	20.050	1.00	5.03	C
ATOM	515	CE1	HIS	53	-8.834	52.724	22.122	1.00	5.03	C
ATOM	516	NE2	HIS	53	-8.727	51.746	21.231	1.00	5.03	N
ATOM	517	H	HIS	53	-7.056	57.830	19.572	1.00	0.00	H
ATOM	518	HA	HIS	53	-6.273	55.301	20.779	1.00	0.00	H
ATOM	519	HB2	HIS	53	-8.754	55.500	19.220	1.00	0.00	H

ATOM	520	HB3	HIS	53	-7.650	54.469	18.387	1.00	0.00	H
ATOM	521	HD1	HIS	53	-8.537	54.825	22.091	1.00	0.00	H
ATOM	522	HD2	HIS	53	-8.172	51.829	19.140	1.00	0.00	H
ATOM	523	HE1	HIS	53	-9.110	52.563	23.155	1.00	0.00	H
ATOM	524	N	GLY	54	-4.725	56.949	18.917	1.00	4.76	N
ATOM	525	CA	GLY	54	-3.655	57.203	17.956	1.00	4.76	C
ATOM	526	C	GLY	54	-2.313	57.441	18.646	1.00	4.76	C
ATOM	527	O	GLY	54	-2.218	58.282	19.536	1.00	4.76	O
ATOM	528	H	GLY	54	-4.853	57.626	19.655	1.00	0.00	H
ATOM	529	HA2	GLY	54	-3.561	56.407	17.215	1.00	0.00	H
ATOM	530	HA3	GLY	54	-3.910	58.103	17.395	1.00	0.00	H
ATOM	531	N	GLY	55	-1.269	56.744	18.186	1.00	4.21	N
ATOM	532	CA	GLY	55	0.135	56.969	18.519	1.00	4.21	C
ATOM	533	C	GLY	55	0.937	57.307	17.252	1.00	4.21	C
ATOM	534	O	GLY	55	0.345	57.605	16.212	1.00	4.21	O
ATOM	535	H	GLY	55	-1.450	56.066	17.450	1.00	0.00	H
ATOM	536	HA2	GLY	55	0.253	57.778	19.242	1.00	0.00	H
ATOM	537	HA3	GLY	55	0.532	56.063	18.978	1.00	0.00	H
ATOM	538	N	PHE	56	2.276	57.270	17.345	1.00	3.96	N
ATOM	539	CA	PHE	56	3.284	57.628	16.330	1.00	3.96	C
ATOM	540	C	PHE	56	2.951	57.313	14.877	1.00	3.96	C
ATOM	541	O	PHE	56	2.539	58.197	14.128	1.00	3.96	O
ATOM	542	CB	PHE	56	4.716	57.248	16.790	1.00	3.96	C
ATOM	543	CG	PHE	56	5.119	55.783	16.678	1.00	3.96	C
ATOM	544	CD1	PHE	56	4.406	54.774	17.362	1.00	3.96	C
ATOM	545	CD2	PHE	56	6.211	55.425	15.857	1.00	3.96	C
ATOM	546	CE1	PHE	56	4.764	53.421	17.203	1.00	3.96	C
ATOM	547	CE2	PHE	56	6.568	54.073	15.700	1.00	3.96	C
ATOM	548	CZ	PHE	56	5.842	53.070	16.368	1.00	3.96	C
ATOM	549	H	PHE	56	2.648	57.031	18.252	1.00	0.00	H
ATOM	550	HA	PHE	56	3.273	58.719	16.365	1.00	0.00	H
ATOM	551	HB2	PHE	56	5.424	57.838	16.204	1.00	0.00	H
ATOM	552	HB3	PHE	56	4.877	57.570	17.819	1.00	0.00	H
ATOM	553	HD1	PHE	56	3.581	55.020	18.012	1.00	0.00	H
ATOM	554	HD2	PHE	56	6.785	56.184	15.341	1.00	0.00	H
ATOM	555	HE1	PHE	56	4.218	52.650	17.727	1.00	0.00	H
ATOM	556	HE2	PHE	56	7.406	53.809	15.070	1.00	0.00	H
ATOM	557	HZ	PHE	56	6.122	52.033	16.250	1.00	0.00	H
ATOM	558	N	LEU	57	3.091	56.045	14.498	1.00	3.72	N
ATOM	559	CA	LEU	57	2.843	55.549	13.156	1.00	3.72	C
ATOM	560	C	LEU	57	1.413	55.839	12.683	1.00	3.72	C
ATOM	561	O	LEU	57	1.210	56.247	11.546	1.00	3.72	O
ATOM	562	CB	LEU	57	3.169	54.033	13.138	1.00	3.72	C
ATOM	563	CG	LEU	57	2.847	53.257	11.836	1.00	3.72	C
ATOM	564	CD1	LEU	57	3.616	53.789	10.618	1.00	3.72	C
ATOM	565	CD2	LEU	57	3.119	51.757	12.023	1.00	3.72	C
ATOM	566	H	LEU	57	3.505	55.406	15.165	1.00	0.00	H
ATOM	567	HA	LEU	57	3.521	56.066	12.477	1.00	0.00	H
ATOM	568	HB2	LEU	57	4.228	53.903	13.368	1.00	0.00	H
ATOM	569	HB3	LEU	57	2.630	53.552	13.956	1.00	0.00	H
ATOM	570	HG	LEU	57	1.782	53.353	11.619	1.00	0.00	H
ATOM	571	HD11	LEU	57	3.337	53.244	9.716	1.00	0.00	H
ATOM	572	HD12	LEU	57	3.408	54.843	10.430	1.00	0.00	H
ATOM	573	HD13	LEU	57	4.693	53.681	10.743	1.00	0.00	H
ATOM	574	HD21	LEU	57	2.858	51.195	11.125	1.00	0.00	H
ATOM	575	HD22	LEU	57	4.172	51.567	12.237	1.00	0.00	H
ATOM	576	HD23	LEU	57	2.533	51.345	12.845	1.00	0.00	H
ATOM	577	N	THR	58	0.426	55.668	13.564	1.00	3.35	N
ATOM	578	CA	THR	58	-0.980	55.825	13.235	1.00	3.35	C
ATOM	579	C	THR	58	-1.420	57.272	12.981	1.00	3.35	C
ATOM	580	O	THR	58	-2.196	57.507	12.054	1.00	3.35	O
ATOM	581	CB	THR	58	-1.818	55.157	14.354	1.00	3.35	C
ATOM	582	OG1	THR	58	-1.331	55.515	15.636	1.00	3.35	O

ATOM	583	CG2	THR	58	-1.795	53.624	14.282	1.00	3.35	C
ATOM	584	H	THR	58	0.640	55.366	14.502	1.00	0.00	H
ATOM	585	HA	THR	58	-1.175	55.284	12.306	1.00	0.00	H
ATOM	586	HB	THR	58	-2.857	55.487	14.276	1.00	0.00	H
ATOM	587	HG1	THR	58	-1.067	56.433	15.615	1.00	0.00	H
ATOM	588	HG21	THR	58	-2.409	53.186	15.070	1.00	0.00	H
ATOM	589	HG22	THR	58	-2.191	53.272	13.328	1.00	0.00	H
ATOM	590	HG23	THR	58	-0.785	53.228	14.388	1.00	0.00	H
ATOM	591	N	ILE	59	-0.890	58.243	13.740	1.00	3.07	N
ATOM	592	CA	ILE	59	-1.062	59.667	13.457	1.00	3.07	C
ATOM	593	C	ILE	59	-0.421	60.000	12.105	1.00	3.07	C
ATOM	594	O	ILE	59	-1.032	60.647	11.254	1.00	3.07	O
ATOM	595	CB	ILE	59	-0.520	60.520	14.647	1.00	3.07	C
ATOM	596	CG1	ILE	59	-1.564	60.575	15.787	1.00	3.07	C
ATOM	597	CG2	ILE	59	-0.034	61.945	14.290	1.00	3.07	C
ATOM	598	CD1	ILE	59	-2.732	61.542	15.510	1.00	3.07	C
ATOM	599	H	ILE	59	-0.228	57.999	14.472	1.00	0.00	H
ATOM	600	HA	ILE	59	-2.129	59.857	13.329	1.00	0.00	H
ATOM	601	HB	ILE	59	0.361	60.009	15.040	1.00	0.00	H
ATOM	602	HG12	ILE	59	-1.956	59.574	15.972	1.00	0.00	H
ATOM	603	HG13	ILE	59	-1.077	60.871	16.718	1.00	0.00	H
ATOM	604	HG21	ILE	59	0.227	62.507	15.188	1.00	0.00	H
ATOM	605	HG22	ILE	59	0.859	61.925	13.664	1.00	0.00	H
ATOM	606	HG23	ILE	59	-0.797	62.513	13.755	1.00	0.00	H
ATOM	607	HD11	ILE	59	-3.553	61.398	16.206	1.00	0.00	H
ATOM	608	HD12	ILE	59	-2.416	62.580	15.600	1.00	0.00	H
ATOM	609	HD13	ILE	59	-3.142	61.418	14.510	1.00	0.00	H
ATOM	610	N	ASN	60	0.799	59.498	11.902	1.00	3.02	N
ATOM	611	CA	ASN	60	1.608	59.815	10.735	1.00	3.02	C
ATOM	612	C	ASN	60	1.101	59.189	9.437	1.00	3.02	C
ATOM	613	O	ASN	60	1.146	59.815	8.379	1.00	3.02	O
ATOM	614	CB	ASN	60	3.085	59.618	11.055	1.00	3.02	C
ATOM	615	CG	ASN	60	3.516	60.738	12.009	1.00	3.02	C
ATOM	616	OD1	ASN	60	3.529	61.902	11.635	1.00	3.02	O
ATOM	617	ND2	ASN	60	3.830	60.434	13.257	1.00	3.02	N
ATOM	618	H	ASN	60	1.221	58.951	12.642	1.00	0.00	H
ATOM	619	HA	ASN	60	1.464	60.882	10.542	1.00	0.00	H
ATOM	620	HB2	ASN	60	3.265	58.634	11.485	1.00	0.00	H
ATOM	621	HB3	ASN	60	3.684	59.695	10.148	1.00	0.00	H
ATOM	622	HD22	ASN	60	4.197	61.164	13.848	1.00	0.00	H
ATOM	623	HD21	ASN	60	3.646	59.499	13.605	1.00	0.00	H
ATOM	624	N	LEU	61	0.475	58.021	9.546	1.00	2.88	N
ATOM	625	CA	LEU	61	-0.270	57.350	8.497	1.00	2.88	C
ATOM	626	C	LEU	61	-1.508	58.104	8.062	1.00	2.88	C
ATOM	627	O	LEU	61	-1.728	58.311	6.866	1.00	2.88	O
ATOM	628	CB	LEU	61	-0.598	55.927	9.028	1.00	2.88	C
ATOM	629	CG	LEU	61	-1.450	54.990	8.141	1.00	2.88	C
ATOM	630	CD1	LEU	61	-1.001	53.527	8.302	1.00	2.88	C
ATOM	631	CD2	LEU	61	-2.955	55.087	8.463	1.00	2.88	C
ATOM	632	H	LEU	61	0.561	57.536	10.436	1.00	0.00	H
ATOM	633	HA	LEU	61	0.360	57.284	7.617	1.00	0.00	H
ATOM	634	HB2	LEU	61	0.364	55.442	9.200	1.00	0.00	H
ATOM	635	HB3	LEU	61	-1.059	55.995	10.013	1.00	0.00	H
ATOM	636	HG	LEU	61	-1.284	55.266	7.100	1.00	0.00	H
ATOM	637	HD11	LEU	61	-1.576	52.863	7.656	1.00	0.00	H
ATOM	638	HD12	LEU	61	0.049	53.397	8.041	1.00	0.00	H
ATOM	639	HD13	LEU	61	-1.128	53.183	9.330	1.00	0.00	H
ATOM	640	HD21	LEU	61	-3.533	54.377	7.870	1.00	0.00	H
ATOM	641	HD22	LEU	61	-3.153	54.868	9.513	1.00	0.00	H
ATOM	642	HD23	LEU	61	-3.362	56.073	8.251	1.00	0.00	H
ATOM	643	N	ALA	62	-2.300	58.540	9.041	1.00	2.58	N
ATOM	644	CA	ALA	62	-3.547	59.227	8.787	1.00	2.58	C
ATOM	645	C	ALA	62	-3.332	60.569	8.087	1.00	2.58	C

ATOM	646	O	ALA	62	-4.041	60.897	7.137	1.00	2.58	O
ATOM	647	CB	ALA	62	-4.274	59.451	10.123	1.00	2.58	C
ATOM	648	H	ALA	62	-2.043	58.346	10.002	1.00	0.00	H
ATOM	649	HA	ALA	62	-4.170	58.602	8.144	1.00	0.00	H
ATOM	650	HB1	ALA	62	-5.226	59.962	9.969	1.00	0.00	H
ATOM	651	HB2	ALA	62	-4.488	58.503	10.618	1.00	0.00	H
ATOM	652	HB3	ALA	62	-3.685	60.058	10.812	1.00	0.00	H
ATOM	653	N	PHE	63	-2.289	61.289	8.504	1.00	2.25	N
ATOM	654	CA	PHE	63	-1.867	62.555	7.922	1.00	2.25	C
ATOM	655	C	PHE	63	-1.315	62.410	6.516	1.00	2.25	C
ATOM	656	O	PHE	63	-1.455	63.310	5.692	1.00	2.25	O
ATOM	657	CB	PHE	63	-0.773	63.171	8.843	1.00	2.25	C
ATOM	658	CG	PHE	63	-0.843	64.672	9.110	1.00	2.25	C
ATOM	659	CD1	PHE	63	-0.831	65.608	8.052	1.00	2.25	C
ATOM	660	CD2	PHE	63	-0.885	65.140	10.442	1.00	2.25	C
ATOM	661	CE1	PHE	63	-0.877	66.990	8.323	1.00	2.25	C
ATOM	662	CE2	PHE	63	-0.917	66.522	10.713	1.00	2.25	C
ATOM	663	CZ	PHE	63	-0.917	67.449	9.654	1.00	2.25	C
ATOM	664	H	PHE	63	-1.777	60.942	9.310	1.00	0.00	H
ATOM	665	HA	PHE	63	-2.735	63.218	7.900	1.00	0.00	H
ATOM	666	HB2	PHE	63	-0.803	62.688	9.817	1.00	0.00	H
ATOM	667	HB3	PHE	63	0.227	62.946	8.466	1.00	0.00	H
ATOM	668	HD1	PHE	63	-0.780	65.281	7.025	1.00	0.00	H
ATOM	669	HD2	PHE	63	-0.893	64.444	11.270	1.00	0.00	H
ATOM	670	HE1	PHE	63	-0.881	67.694	7.505	1.00	0.00	H
ATOM	671	HE2	PHE	63	-0.956	66.868	11.737	1.00	0.00	H
ATOM	672	HZ	PHE	63	-0.953	68.507	9.867	1.00	0.00	H
ATOM	673	N	GLY	64	-0.741	61.253	6.208	1.00	2.03	N
ATOM	674	CA	GLY	64	-0.067	61.083	4.949	1.00	2.03	C
ATOM	675	C	GLY	64	-1.015	60.593	3.886	1.00	2.03	C
ATOM	676	O	GLY	64	-1.030	61.136	2.783	1.00	2.03	O
ATOM	677	H	GLY	64	-0.643	60.537	6.916	1.00	0.00	H
ATOM	678	HA2	GLY	64	0.390	62.017	4.630	1.00	0.00	H
ATOM	679	HA3	GLY	64	0.734	60.358	5.086	1.00	0.00	H
ATOM	680	N	PHE	65	-1.914	59.679	4.248	1.00	1.70	N
ATOM	681	CA	PHE	65	-3.043	59.337	3.408	1.00	1.70	C
ATOM	682	C	PHE	65	-4.018	60.483	3.205	1.00	1.70	C
ATOM	683	O	PHE	65	-4.591	60.609	2.123	1.00	1.70	O
ATOM	684	CB	PHE	65	-3.803	58.129	4.004	1.00	1.70	C
ATOM	685	CG	PHE	65	-3.128	56.764	3.899	1.00	1.70	C
ATOM	686	CD1	PHE	65	-2.413	56.378	2.741	1.00	1.70	C
ATOM	687	CD2	PHE	65	-3.268	55.838	4.954	1.00	1.70	C
ATOM	688	CE1	PHE	65	-1.827	55.103	2.654	1.00	1.70	C
ATOM	689	CE2	PHE	65	-2.702	54.551	4.855	1.00	1.70	C
ATOM	690	CZ	PHE	65	-1.972	54.186	3.710	1.00	1.70	C
ATOM	691	H	PHE	65	-1.847	59.257	5.172	1.00	0.00	H
ATOM	692	HA	PHE	65	-2.695	59.041	2.432	1.00	0.00	H
ATOM	693	HB2	PHE	65	-4.045	58.333	5.048	1.00	0.00	H
ATOM	694	HB3	PHE	65	-4.764	58.019	3.497	1.00	0.00	H
ATOM	695	HD1	PHE	65	-2.308	57.036	1.895	1.00	0.00	H
ATOM	696	HD2	PHE	65	-3.821	56.109	5.841	1.00	0.00	H
ATOM	697	HE1	PHE	65	-1.277	54.829	1.765	1.00	0.00	H
ATOM	698	HE2	PHE	65	-2.820	53.844	5.662	1.00	0.00	H
ATOM	699	HZ	PHE	65	-1.529	53.203	3.639	1.00	0.00	H
ATOM	700	N	ALA	66	-4.137	61.366	4.196	1.00	1.45	N
ATOM	701	CA	ALA	66	-4.880	62.596	4.058	1.00	1.45	C
ATOM	702	C	ALA	66	-4.353	63.506	2.936	1.00	1.45	C
ATOM	703	O	ALA	66	-5.131	64.079	2.171	1.00	1.45	O
ATOM	704	CB	ALA	66	-4.871	63.379	5.370	1.00	1.45	C
ATOM	705	H	ALA	66	-3.688	61.179	5.086	1.00	0.00	H
ATOM	706	HA	ALA	66	-5.915	62.346	3.817	1.00	0.00	H
ATOM	707	HB1	ALA	66	-5.350	64.345	5.229	1.00	0.00	H
ATOM	708	HB2	ALA	66	-5.396	62.837	6.153	1.00	0.00	H

ATOM	709	HB3	ALA	66	-3.869	63.589	5.726	1.00	0.00	H
ATOM	710	N	VAL	67	-3.026	63.591	2.819	1.00	1.37	N
ATOM	711	CA	VAL	67	-2.341	64.314	1.761	1.00	1.37	C
ATOM	712	C	VAL	67	-2.508	63.647	0.397	1.00	1.37	C
ATOM	713	O	VAL	67	-2.674	64.369	-0.585	1.00	1.37	O
ATOM	714	CB	VAL	67	-0.836	64.482	2.150	1.00	1.37	C
ATOM	715	CG1	VAL	67	0.089	64.968	1.014	1.00	1.37	C
ATOM	716	CG2	VAL	67	-0.665	65.427	3.354	1.00	1.37	C
ATOM	717	H	VAL	67	-2.452	63.114	3.503	1.00	0.00	H
ATOM	718	HA	VAL	67	-2.784	65.309	1.679	1.00	0.00	H
ATOM	719	HB	VAL	67	-0.452	63.511	2.454	1.00	0.00	H
ATOM	720	HG11	VAL	67	1.102	65.144	1.378	1.00	0.00	H
ATOM	721	HG12	VAL	67	0.174	64.232	0.214	1.00	0.00	H
ATOM	722	HG13	VAL	67	-0.272	65.899	0.574	1.00	0.00	H
ATOM	723	HG21	VAL	67	0.338	65.350	3.775	1.00	0.00	H
ATOM	724	HG22	VAL	67	-0.822	66.463	3.067	1.00	0.00	H
ATOM	725	HG23	VAL	67	-1.369	65.212	4.155	1.00	0.00	H
ATOM	726	N	THR	68	-2.548	62.305	0.325	1.00	1.50	N
ATOM	727	CA	THR	68	-2.787	61.614	-0.943	1.00	1.50	C
ATOM	728	C	THR	68	-4.139	62.038	-1.540	1.00	1.50	C
ATOM	729	O	THR	68	-4.248	62.343	-2.727	1.00	1.50	O
ATOM	730	CB	THR	68	-2.864	60.042	-0.822	1.00	1.50	C
ATOM	731	OG1	THR	68	-4.037	59.487	-0.240	1.00	1.50	O
ATOM	732	CG2	THR	68	-1.752	59.366	-0.063	1.00	1.50	C
ATOM	733	H	THR	68	-2.354	61.767	1.160	1.00	0.00	H
ATOM	734	HA	THR	68	-1.992	61.895	-1.636	1.00	0.00	H
ATOM	735	HB	THR	68	-2.860	59.645	-1.838	1.00	0.00	H
ATOM	736	HG1	THR	68	-4.190	59.877	0.620	1.00	0.00	H
ATOM	737	HG21	THR	68	-1.923	58.298	0.072	1.00	0.00	H
ATOM	738	HG22	THR	68	-0.803	59.465	-0.564	1.00	0.00	H
ATOM	739	HG23	THR	68	-1.663	59.836	0.898	1.00	0.00	H
ATOM	740	N	LEU	69	-5.160	62.079	-0.669	1.00	1.70	N
ATOM	741	CA	LEU	69	-6.538	62.340	-1.014	1.00	1.70	C
ATOM	742	C	LEU	69	-6.674	63.776	-1.525	1.00	1.70	C
ATOM	743	O	LEU	69	-7.244	63.999	-2.591	1.00	1.70	O
ATOM	744	CB	LEU	69	-7.456	62.109	0.207	1.00	1.70	C
ATOM	745	CG	LEU	69	-7.635	60.626	0.603	1.00	1.70	C
ATOM	746	CD1	LEU	69	-8.209	60.505	2.023	1.00	1.70	C
ATOM	747	CD2	LEU	69	-8.516	59.858	-0.397	1.00	1.70	C
ATOM	748	H	LEU	69	-4.960	61.821	0.288	1.00	0.00	H
ATOM	749	HA	LEU	69	-6.825	61.676	-1.830	1.00	0.00	H
ATOM	750	HB2	LEU	69	-7.061	62.666	1.057	1.00	0.00	H
ATOM	751	HB3	LEU	69	-8.440	62.539	0.014	1.00	0.00	H
ATOM	752	HG	LEU	69	-6.657	60.145	0.611	1.00	0.00	H
ATOM	753	HD11	LEU	69	-8.312	59.461	2.318	1.00	0.00	H
ATOM	754	HD12	LEU	69	-7.556	60.983	2.753	1.00	0.00	H
ATOM	755	HD13	LEU	69	-9.191	60.972	2.100	1.00	0.00	H
ATOM	756	HD21	LEU	69	-8.641	58.819	-0.089	1.00	0.00	H
ATOM	757	HD22	LEU	69	-9.509	60.300	-0.478	1.00	0.00	H
ATOM	758	HD23	LEU	69	-8.076	59.840	-1.394	1.00	0.00	H
ATOM	759	N	GLY	70	-6.054	64.718	-0.805	1.00	1.92	N
ATOM	760	CA	GLY	70	-5.696	66.060	-1.247	1.00	1.92	C
ATOM	761	C	GLY	70	-5.177	66.179	-2.679	1.00	1.92	C
ATOM	762	O	GLY	70	-5.634	67.022	-3.453	1.00	1.92	O
ATOM	763	H	GLY	70	-5.619	64.404	0.056	1.00	0.00	H
ATOM	764	HA2	GLY	70	-6.554	66.717	-1.145	1.00	0.00	H
ATOM	765	HA3	GLY	70	-4.930	66.451	-0.576	1.00	0.00	H
ATOM	766	N	ILE	71	-4.219	65.324	-3.030	1.00	2.13	N
ATOM	767	CA	ILE	71	-3.549	65.357	-4.315	1.00	2.13	C
ATOM	768	C	ILE	71	-4.383	64.733	-5.431	1.00	2.13	C
ATOM	769	O	ILE	71	-4.436	65.304	-6.523	1.00	2.13	O
ATOM	770	CB	ILE	71	-2.126	64.738	-4.172	1.00	2.13	C
ATOM	771	CG1	ILE	71	-1.258	65.636	-3.253	1.00	2.13	C

ATOM	772	CG2	ILE	71	-1.390	64.525	-5.515	1.00	2.13	C
ATOM	773	CD1	ILE	71	0.032	64.978	-2.762	1.00	2.13	C
ATOM	774	H	ILE	71	-3.867	64.675	-2.333	1.00	0.00	H
ATOM	775	HA	ILE	71	-3.414	66.402	-4.596	1.00	0.00	H
ATOM	776	HB	ILE	71	-2.231	63.762	-3.697	1.00	0.00	H
ATOM	777	HG12	ILE	71	-1.008	66.567	-3.764	1.00	0.00	H
ATOM	778	HG13	ILE	71	-1.818	65.938	-2.371	1.00	0.00	H
ATOM	779	HG21	ILE	71	-0.426	64.037	-5.371	1.00	0.00	H
ATOM	780	HG22	ILE	71	-1.949	63.896	-6.205	1.00	0.00	H
ATOM	781	HG23	ILE	71	-1.201	65.471	-6.020	1.00	0.00	H
ATOM	782	HD11	ILE	71	0.528	65.606	-2.022	1.00	0.00	H
ATOM	783	HD12	ILE	71	-0.172	64.012	-2.299	1.00	0.00	H
ATOM	784	HD13	ILE	71	0.736	64.835	-3.581	1.00	0.00	H
ATOM	785	N	LEU	72	-5.106	63.643	-5.153	1.00	2.56	N
ATOM	786	CA	LEU	72	-6.087	63.088	-6.082	1.00	2.56	C
ATOM	787	C	LEU	72	-7.184	64.109	-6.403	1.00	2.56	C
ATOM	788	O	LEU	72	-7.595	64.208	-7.561	1.00	2.56	O
ATOM	789	CB	LEU	72	-6.757	61.810	-5.502	1.00	2.56	C
ATOM	790	CG	LEU	72	-6.054	60.471	-5.821	1.00	2.56	C
ATOM	791	CD1	LEU	72	-4.883	60.192	-4.878	1.00	2.56	C
ATOM	792	CD2	LEU	72	-7.047	59.298	-5.771	1.00	2.56	C
ATOM	793	H	LEU	72	-4.997	63.220	-4.234	1.00	0.00	H
ATOM	794	HA	LEU	72	-5.584	62.847	-7.021	1.00	0.00	H
ATOM	795	HB2	LEU	72	-6.917	61.913	-4.428	1.00	0.00	H
ATOM	796	HB3	LEU	72	-7.762	61.736	-5.922	1.00	0.00	H
ATOM	797	HG	LEU	72	-5.667	60.518	-6.840	1.00	0.00	H
ATOM	798	HD11	LEU	72	-4.355	59.279	-5.154	1.00	0.00	H
ATOM	799	HD12	LEU	72	-4.170	61.011	-4.909	1.00	0.00	H
ATOM	800	HD13	LEU	72	-5.217	60.081	-3.845	1.00	0.00	H
ATOM	801	HD21	LEU	72	-6.554	58.356	-6.013	1.00	0.00	H
ATOM	802	HD22	LEU	72	-7.491	59.195	-4.780	1.00	0.00	H
ATOM	803	HD23	LEU	72	-7.858	59.432	-6.489	1.00	0.00	H
ATOM	804	N	ILE	73	-7.647	64.871	-5.397	1.00	2.78	N
ATOM	805	CA	ILE	73	-8.701	65.842	-5.629	1.00	2.78	C
ATOM	806	C	ILE	73	-8.275	67.085	-6.439	1.00	2.78	C
ATOM	807	O	ILE	73	-9.117	67.680	-7.117	1.00	2.78	O
ATOM	808	CB	ILE	73	-9.514	66.244	-4.362	1.00	2.78	C
ATOM	809	CG1	ILE	73	-8.757	67.063	-3.297	1.00	2.78	C
ATOM	810	CG2	ILE	73	-10.256	65.035	-3.757	1.00	2.78	C
ATOM	811	CD1	ILE	73	-9.677	67.875	-2.372	1.00	2.78	C
ATOM	812	H	ILE	73	-7.310	64.716	-4.450	1.00	0.00	H
ATOM	813	HA	ILE	73	-9.431	65.349	-6.276	1.00	0.00	H
ATOM	814	HB	ILE	73	-10.299	66.905	-4.734	1.00	0.00	H
ATOM	815	HG12	ILE	73	-8.140	66.396	-2.703	1.00	0.00	H
ATOM	816	HG13	ILE	73	-8.078	67.768	-3.773	1.00	0.00	H
ATOM	817	HG21	ILE	73	-10.916	65.333	-2.944	1.00	0.00	H
ATOM	818	HG22	ILE	73	-10.873	64.541	-4.507	1.00	0.00	H
ATOM	819	HG23	ILE	73	-9.571	64.288	-3.364	1.00	0.00	H
ATOM	820	HD11	ILE	73	-9.093	68.516	-1.711	1.00	0.00	H
ATOM	821	HD12	ILE	73	-10.343	68.523	-2.942	1.00	0.00	H
ATOM	822	HD13	ILE	73	-10.289	67.229	-1.743	1.00	0.00	H
ATOM	823	N	ALA	74	-7.001	67.486	-6.378	1.00	2.77	N
ATOM	824	CA	ALA	74	-6.546	68.760	-6.927	1.00	2.77	C
ATOM	825	C	ALA	74	-5.599	68.652	-8.113	1.00	2.77	C
ATOM	826	O	ALA	74	-5.541	69.584	-8.921	1.00	2.77	O
ATOM	827	CB	ALA	74	-5.844	69.537	-5.799	1.00	2.77	C
ATOM	828	H	ALA	74	-6.378	66.963	-5.772	1.00	0.00	H
ATOM	829	HA	ALA	74	-7.388	69.371	-7.258	1.00	0.00	H
ATOM	830	HB1	ALA	74	-5.444	70.490	-6.153	1.00	0.00	H
ATOM	831	HB2	ALA	74	-6.534	69.759	-4.985	1.00	0.00	H
ATOM	832	HB3	ALA	74	-5.014	68.973	-5.372	1.00	0.00	H
ATOM	833	N	GLY	75	-4.859	67.542	-8.209	1.00	2.84	N
ATOM	834	CA	GLY	75	-3.844	67.217	-9.206	1.00	2.84	C

ATOM	835	C	GLY	75	-4.161	67.682	-10.626	1.00	2.84	C
ATOM	836	O	GLY	75	-3.389	68.409	-11.249	1.00	2.84	O
ATOM	837	H	GLY	75	-4.966	66.840	-7.479	1.00	0.00	H
ATOM	838	HA2	GLY	75	-2.894	67.650	-8.894	1.00	0.00	H
ATOM	839	HA3	GLY	75	-3.694	66.137	-9.213	1.00	0.00	H
ATOM	840	N	GLN	76	-5.367	67.332	-11.090	1.00	2.85	N
ATOM	841	CA	GLN	76	-5.865	67.615	-12.432	1.00	2.85	C
ATOM	842	C	GLN	76	-6.137	69.108	-12.709	1.00	2.85	C
ATOM	843	O	GLN	76	-6.429	69.475	-13.848	1.00	2.85	O
ATOM	844	CB	GLN	76	-7.134	66.739	-12.682	1.00	2.85	C
ATOM	845	CG	GLN	76	-8.524	67.324	-12.281	1.00	2.85	C
ATOM	846	CD	GLN	76	-9.302	68.079	-13.377	1.00	2.85	C
ATOM	847	OE1	GLN	76	-10.154	68.923	-13.089	1.00	2.85	O
ATOM	848	NE2	GLN	76	-9.012	67.838	-14.650	1.00	2.85	N
ATOM	849	H	GLN	76	-5.942	66.784	-10.467	1.00	0.00	H
ATOM	850	HA	GLN	76	-5.087	67.298	-13.130	1.00	0.00	H
ATOM	851	HB2	GLN	76	-7.128	66.432	-13.726	1.00	0.00	H
ATOM	852	HB3	GLN	76	-7.007	65.792	-12.153	1.00	0.00	H
ATOM	853	HG2	GLN	76	-9.166	66.492	-11.989	1.00	0.00	H
ATOM	854	HG3	GLN	76	-8.438	67.935	-11.385	1.00	0.00	H
ATOM	855	HE22	GLN	76	-9.523	68.287	-15.390	1.00	0.00	H
ATOM	856	HE21	GLN	76	-8.266	67.196	-14.874	1.00	0.00	H
ATOM	857	N	VAL	77	-6.130	69.952	-11.675	1.00	2.43	N
ATOM	858	CA	VAL	77	-6.511	71.349	-11.753	1.00	2.43	C
ATOM	859	C	VAL	77	-5.297	72.260	-11.598	1.00	2.43	C
ATOM	860	O	VAL	77	-5.110	73.168	-12.412	1.00	2.43	O
ATOM	861	CB	VAL	77	-7.507	71.684	-10.588	1.00	2.43	C
ATOM	862	CG1	VAL	77	-8.133	73.082	-10.747	1.00	2.43	C
ATOM	863	CG2	VAL	77	-8.601	70.626	-10.373	1.00	2.43	C
ATOM	864	H	VAL	77	-5.831	69.595	-10.769	1.00	0.00	H
ATOM	865	HA	VAL	77	-6.991	71.568	-12.708	1.00	0.00	H
ATOM	866	HB	VAL	77	-6.965	71.702	-9.640	1.00	0.00	H
ATOM	867	HG11	VAL	77	-8.914	73.254	-10.008	1.00	0.00	H
ATOM	868	HG12	VAL	77	-7.390	73.868	-10.614	1.00	0.00	H
ATOM	869	HG13	VAL	77	-8.568	73.205	-11.733	1.00	0.00	H
ATOM	870	HG21	VAL	77	-9.341	70.952	-9.641	1.00	0.00	H
ATOM	871	HG22	VAL	77	-9.114	70.394	-11.299	1.00	0.00	H
ATOM	872	HG23	VAL	77	-8.178	69.700	-9.980	1.00	0.00	H
ATOM	873	N	SER	78	-4.563	72.093	-10.495	1.00	2.20	N
ATOM	874	CA	SER	78	-3.541	73.026	-10.065	1.00	2.20	C
ATOM	875	C	SER	78	-2.125	72.461	-10.218	1.00	2.20	C
ATOM	876	O	SER	78	-1.171	73.241	-10.271	1.00	2.20	O
ATOM	877	CB	SER	78	-3.814	73.427	-8.601	1.00	2.20	C
ATOM	878	OG	SER	78	-3.770	72.327	-7.714	1.00	2.20	O
ATOM	879	H	SER	78	-4.793	71.353	-9.835	1.00	0.00	H
ATOM	880	HA	SER	78	-3.575	73.940	-10.661	1.00	0.00	H
ATOM	881	HB2	SER	78	-3.064	74.150	-8.274	1.00	0.00	H
ATOM	882	HB3	SER	78	-4.774	73.929	-8.506	1.00	0.00	H
ATOM	883	HG	SER	78	-3.207	72.600	-6.963	1.00	0.00	H
ATOM	884	N	GLY	79	-2.002	71.133	-10.251	1.00	2.00	N
ATOM	885	CA	GLY	79	-0.761	70.387	-10.101	1.00	2.00	C
ATOM	886	C	GLY	79	-0.754	69.678	-8.743	1.00	2.00	C
ATOM	887	O	GLY	79	-0.121	68.638	-8.615	1.00	2.00	O
ATOM	888	H	GLY	79	-2.841	70.568	-10.239	1.00	0.00	H
ATOM	889	HA2	GLY	79	-0.694	69.641	-10.893	1.00	0.00	H
ATOM	890	HA3	GLY	79	0.123	71.022	-10.177	1.00	0.00	H
ATOM	891	N	ALA	80	-1.471	70.242	-7.754	1.00	1.62	N
ATOM	892	CA	ALA	80	-1.536	69.860	-6.345	1.00	1.62	C
ATOM	893	C	ALA	80	-0.184	70.101	-5.671	1.00	1.62	C
ATOM	894	O	ALA	80	0.517	69.151	-5.330	1.00	1.62	O
ATOM	895	CB	ALA	80	-2.056	68.430	-6.133	1.00	1.62	C
ATOM	896	H	ALA	80	-1.964	71.096	-7.976	1.00	0.00	H
ATOM	897	HA	ALA	80	-2.246	70.536	-5.867	1.00	0.00	H

ATOM	898	HB1	ALA	80	-2.082	68.201	-5.069	1.00	0.00	H
ATOM	899	HB2	ALA	80	-3.064	68.315	-6.513	1.00	0.00	H
ATOM	900	HB3	ALA	80	-1.419	67.686	-6.610	1.00	0.00	H
ATOM	901	N	HIE	81	0.186	71.375	-5.490	1.00	1.13	N
ATOM	902	CA	HIE	81	1.403	71.716	-4.755	1.00	1.13	C
ATOM	903	C	HIE	81	1.102	71.635	-3.252	1.00	1.13	C
ATOM	904	O	HIE	81	1.824	70.968	-2.514	1.00	1.13	O
ATOM	905	CB	HIE	81	1.871	73.143	-5.100	1.00	1.13	C
ATOM	906	CG	HIE	81	2.443	73.348	-6.476	1.00	1.13	C
ATOM	907	ND1	HIE	81	2.958	74.594	-6.858	1.00	1.13	N
ATOM	908	CD2	HIE	81	2.578	72.475	-7.536	1.00	1.13	C
ATOM	909	CE1	HIE	81	3.408	74.420	-8.093	1.00	1.13	C
ATOM	910	NE2	HIE	81	3.188	73.193	-8.556	1.00	1.13	N
ATOM	911	H	HIE	81	-0.506	72.106	-5.672	1.00	0.00	H
ATOM	912	HA	HIE	81	2.206	71.011	-4.982	1.00	0.00	H
ATOM	913	HB2	HIE	81	1.060	73.857	-4.966	1.00	0.00	H
ATOM	914	HB3	HIE	81	2.652	73.426	-4.393	1.00	0.00	H
ATOM	915	HD2	HIE	81	2.299	71.433	-7.633	1.00	0.00	H
ATOM	916	HE1	HIE	81	3.894	75.196	-8.668	1.00	0.00	H
ATOM	917	HE2	HIE	81	3.428	72.878	-9.492	1.00	0.00	H
ATOM	918	N	LEU	82	0.028	72.311	-2.812	1.00	0.99	N
ATOM	919	CA	LEU	82	-0.611	72.246	-1.490	1.00	0.99	C
ATOM	920	C	LEU	82	0.321	72.680	-0.339	1.00	0.99	C
ATOM	921	O	LEU	82	0.096	72.347	0.826	1.00	0.99	O
ATOM	922	CB	LEU	82	-1.243	70.854	-1.219	1.00	0.99	C
ATOM	923	CG	LEU	82	-2.092	70.246	-2.360	1.00	0.99	C
ATOM	924	CD1	LEU	82	-2.707	68.906	-1.932	1.00	0.99	C
ATOM	925	CD2	LEU	82	-3.171	71.205	-2.886	1.00	0.99	C
ATOM	926	H	LEU	82	-0.457	72.915	-3.484	1.00	0.00	H
ATOM	927	HA	LEU	82	-1.415	72.978	-1.527	1.00	0.00	H
ATOM	928	HB2	LEU	82	-0.450	70.148	-0.964	1.00	0.00	H
ATOM	929	HB3	LEU	82	-1.866	70.934	-0.327	1.00	0.00	H
ATOM	930	HG	LEU	82	-1.420	70.021	-3.186	1.00	0.00	H
ATOM	931	HD11	LEU	82	-3.223	68.430	-2.765	1.00	0.00	H
ATOM	932	HD12	LEU	82	-1.936	68.216	-1.589	1.00	0.00	H
ATOM	933	HD13	LEU	82	-3.422	69.031	-1.122	1.00	0.00	H
ATOM	934	HD21	LEU	82	-3.839	70.711	-3.591	1.00	0.00	H
ATOM	935	HD22	LEU	82	-3.766	71.619	-2.075	1.00	0.00	H
ATOM	936	HD23	LEU	82	-2.720	72.044	-3.411	1.00	0.00	H
ATOM	937	N	ASN	83	1.417	73.365	-0.675	1.00	0.59	N
ATOM	938	CA	ASN	83	2.600	73.567	0.147	1.00	0.59	C
ATOM	939	C	ASN	83	3.393	74.721	-0.489	1.00	0.59	C
ATOM	940	O	ASN	83	3.897	74.533	-1.602	1.00	0.59	O
ATOM	941	CB	ASN	83	3.420	72.251	0.159	1.00	0.59	C
ATOM	942	CG	ASN	83	4.626	72.247	1.103	1.00	0.59	C
ATOM	943	OD1	ASN	83	5.178	73.290	1.443	1.00	0.59	O
ATOM	944	ND2	ASN	83	5.026	71.068	1.568	1.00	0.59	N
ATOM	945	H	ASN	83	1.507	73.604	-1.654	1.00	0.00	H
ATOM	946	HA	ASN	83	2.289	73.793	1.165	1.00	0.00	H
ATOM	947	HB2	ASN	83	2.771	71.421	0.436	1.00	0.00	H
ATOM	948	HB3	ASN	83	3.758	72.029	-0.848	1.00	0.00	H
ATOM	949	HD22	ASN	83	5.787	70.997	2.242	1.00	0.00	H
ATOM	950	HD21	ASN	83	4.605	70.219	1.222	1.00	0.00	H
ATOM	951	N	PRO	84	3.559	75.882	0.188	1.00	0.48	N
ATOM	952	CA	PRO	84	4.385	77.006	-0.288	1.00	0.48	C
ATOM	953	C	PRO	84	5.821	76.622	-0.686	1.00	0.48	C
ATOM	954	O	PRO	84	6.361	77.142	-1.662	1.00	0.48	O
ATOM	955	CB	PRO	84	4.422	77.989	0.894	1.00	0.48	C
ATOM	956	CG	PRO	84	3.134	77.721	1.655	1.00	0.48	C
ATOM	957	CD	PRO	84	2.852	76.238	1.421	1.00	0.48	C
ATOM	958	HA	PRO	84	3.880	77.462	-1.141	1.00	0.00	H
ATOM	959	HB2	PRO	84	5.270	77.786	1.551	1.00	0.00	H
ATOM	960	HB3	PRO	84	4.519	79.021	0.558	1.00	0.00	H

ATOM	961	HG2	PRO	84	3.186	77.990	2.710	1.00	0.00	H
ATOM	962	HG3	PRO	84	2.327	78.304	1.208	1.00	0.00	H
ATOM	963	HD3	PRO	84	1.779	76.054	1.336	1.00	0.00	H
ATOM	964	HD2	PRO	84	3.240	75.639	2.246	1.00	0.00	H
ATOM	965	N	ALA	85	6.428	75.686	0.054	1.00	0.55	N
ATOM	966	CA	ALA	85	7.811	75.262	-0.115	1.00	0.55	C
ATOM	967	C	ALA	85	8.003	74.459	-1.413	1.00	0.55	C
ATOM	968	O	ALA	85	9.001	74.655	-2.109	1.00	0.55	O
ATOM	969	CB	ALA	85	8.184	74.421	1.115	1.00	0.55	C
ATOM	970	H	ALA	85	5.886	75.203	0.760	1.00	0.00	H
ATOM	971	HA	ALA	85	8.445	76.147	-0.167	1.00	0.00	H
ATOM	972	HB1	ALA	85	9.259	74.436	1.259	1.00	0.00	H
ATOM	973	HB2	ALA	85	7.748	74.817	2.030	1.00	0.00	H
ATOM	974	HB3	ALA	85	7.868	73.380	1.021	1.00	0.00	H
ATOM	975	N	VAL	86	7.000	73.651	-1.793	1.00	0.83	N
ATOM	976	CA	VAL	86	6.939	72.957	-3.078	1.00	0.83	C
ATOM	977	C	VAL	86	6.781	73.977	-4.210	1.00	0.83	C
ATOM	978	O	VAL	86	7.507	73.911	-5.201	1.00	0.83	O
ATOM	979	CB	VAL	86	5.717	71.973	-3.116	1.00	0.83	C
ATOM	980	CG1	VAL	86	5.443	71.338	-4.500	1.00	0.83	C
ATOM	981	CG2	VAL	86	5.800	70.835	-2.087	1.00	0.83	C
ATOM	982	H	VAL	86	6.185	73.612	-1.200	1.00	0.00	H
ATOM	983	HA	VAL	86	7.865	72.399	-3.232	1.00	0.00	H
ATOM	984	HB	VAL	86	4.823	72.547	-2.874	1.00	0.00	H
ATOM	985	HG11	VAL	86	4.639	70.602	-4.441	1.00	0.00	H
ATOM	986	HG12	VAL	86	5.130	72.073	-5.241	1.00	0.00	H
ATOM	987	HG13	VAL	86	6.326	70.827	-4.886	1.00	0.00	H
ATOM	988	HG21	VAL	86	4.821	70.379	-1.936	1.00	0.00	H
ATOM	989	HG22	VAL	86	6.439	70.034	-2.446	1.00	0.00	H
ATOM	990	HG23	VAL	86	6.171	71.169	-1.119	1.00	0.00	H
ATOM	991	N	THR	87	5.840	74.918	-4.057	1.00	0.88	N
ATOM	992	CA	THR	87	5.538	75.949	-5.037	1.00	0.88	C
ATOM	993	C	THR	87	6.765	76.800	-5.396	1.00	0.88	C
ATOM	994	O	THR	87	7.016	77.063	-6.573	1.00	0.88	O
ATOM	995	CB	THR	87	4.350	76.810	-4.518	1.00	0.88	C
ATOM	996	OG1	THR	87	3.193	76.003	-4.379	1.00	0.88	O
ATOM	997	CG2	THR	87	3.938	77.966	-5.442	1.00	0.88	C
ATOM	998	H	THR	87	5.245	74.888	-3.234	1.00	0.00	H
ATOM	999	HA	THR	87	5.246	75.447	-5.958	1.00	0.00	H
ATOM	1000	HB	THR	87	4.592	77.227	-3.540	1.00	0.00	H
ATOM	1001	HG1	THR	87	3.095	75.497	-5.187	1.00	0.00	H
ATOM	1002	HG21	THR	87	3.117	78.526	-4.999	1.00	0.00	H
ATOM	1003	HG22	THR	87	4.756	78.670	-5.591	1.00	0.00	H
ATOM	1004	HG23	THR	87	3.625	77.606	-6.422	1.00	0.00	H
ATOM	1005	N	PHE	88	7.570	77.160	-4.392	1.00	1.26	N
ATOM	1006	CA	PHE	88	8.824	77.871	-4.584	1.00	1.26	C
ATOM	1007	C	PHE	88	9.844	77.010	-5.328	1.00	1.26	C
ATOM	1008	O	PHE	88	10.468	77.467	-6.284	1.00	1.26	O
ATOM	1009	CB	PHE	88	9.415	78.279	-3.210	1.00	1.26	C
ATOM	1010	CG	PHE	88	8.806	79.474	-2.477	1.00	1.26	C
ATOM	1011	CD1	PHE	88	7.455	79.858	-2.637	1.00	1.26	C
ATOM	1012	CD2	PHE	88	9.618	80.196	-1.574	1.00	1.26	C
ATOM	1013	CE1	PHE	88	6.923	80.932	-1.899	1.00	1.26	C
ATOM	1014	CE2	PHE	88	9.090	81.278	-0.843	1.00	1.26	C
ATOM	1015	CZ	PHE	88	7.740	81.645	-1.003	1.00	1.26	C
ATOM	1016	H	PHE	88	7.283	76.941	-3.440	1.00	0.00	H
ATOM	1017	HA	PHE	88	8.653	78.765	-5.181	1.00	0.00	H
ATOM	1018	HB2	PHE	88	9.398	77.427	-2.529	1.00	0.00	H
ATOM	1019	HB3	PHE	88	10.473	78.514	-3.348	1.00	0.00	H
ATOM	1020	HD1	PHE	88	6.794	79.324	-3.302	1.00	0.00	H
ATOM	1021	HD2	PHE	88	10.651	79.914	-1.428	1.00	0.00	H
ATOM	1022	HE1	PHE	88	5.881	81.197	-2.012	1.00	0.00	H
ATOM	1023	HE2	PHE	88	9.718	81.819	-0.151	1.00	0.00	H

ATOM	1024	HZ	PHE	88	7.330	82.465	-0.432	1.00	0.00	H
ATOM	1025	N	ALA	89	9.977	75.748	-4.916	1.00	1.54	N
ATOM	1026	CA	ALA	89	10.946	74.829	-5.479	1.00	1.54	C
ATOM	1027	C	ALA	89	10.674	74.450	-6.940	1.00	1.54	C
ATOM	1028	O	ALA	89	11.598	74.380	-7.754	1.00	1.54	O
ATOM	1029	CB	ALA	89	10.994	73.558	-4.622	1.00	1.54	C
ATOM	1030	H	ALA	89	9.401	75.417	-4.150	1.00	0.00	H
ATOM	1031	HA	ALA	89	11.929	75.303	-5.442	1.00	0.00	H
ATOM	1032	HB1	ALA	89	11.689	72.826	-5.036	1.00	0.00	H
ATOM	1033	HB2	ALA	89	11.341	73.796	-3.618	1.00	0.00	H
ATOM	1034	HB3	ALA	89	10.020	73.076	-4.540	1.00	0.00	H
ATOM	1035	N	MET	90	9.394	74.290	-7.284	1.00	2.07	N
ATOM	1036	CA	MET	90	8.897	74.092	-8.641	1.00	2.07	C
ATOM	1037	C	MET	90	9.263	75.284	-9.527	1.00	2.07	C
ATOM	1038	O	MET	90	9.761	75.112	-10.640	1.00	2.07	O
ATOM	1039	CB	MET	90	7.359	73.944	-8.572	1.00	2.07	C
ATOM	1040	CG	MET	90	6.865	72.601	-8.013	1.00	2.07	C
ATOM	1041	SD	MET	90	6.558	71.348	-9.282	1.00	2.07	S
ATOM	1042	CE	MET	90	6.018	69.963	-8.241	1.00	2.07	C
ATOM	1043	H	MET	90	8.710	74.324	-6.530	1.00	0.00	H
ATOM	1044	HA	MET	90	9.349	73.189	-9.046	1.00	0.00	H
ATOM	1045	HB2	MET	90	6.951	74.748	-7.958	1.00	0.00	H
ATOM	1046	HB3	MET	90	6.914	74.093	-9.554	1.00	0.00	H
ATOM	1047	HG2	MET	90	7.561	72.203	-7.274	1.00	0.00	H
ATOM	1048	HG3	MET	90	5.924	72.762	-7.487	1.00	0.00	H
ATOM	1049	HE1	MET	90	6.805	69.676	-7.542	1.00	0.00	H
ATOM	1050	HE2	MET	90	5.128	70.231	-7.671	1.00	0.00	H
ATOM	1051	HE3	MET	90	5.782	69.096	-8.859	1.00	0.00	H
ATOM	1052	N	CYS	91	9.071	76.495	-9.003	1.00	2.76	N
ATOM	1053	CA	CYS	91	9.387	77.729	-9.696	1.00	2.76	C
ATOM	1054	C	CYS	91	10.875	77.961	-9.953	1.00	2.76	C
ATOM	1055	O	CYS	91	11.244	78.539	-10.976	1.00	2.76	O
ATOM	1056	CB	CYS	91	8.742	78.913	-8.951	1.00	2.76	C
ATOM	1057	SG	CYS	91	6.985	79.012	-9.413	1.00	2.76	S
ATOM	1058	H	CYS	91	8.637	76.569	-8.089	1.00	0.00	H
ATOM	1059	HA	CYS	91	8.947	77.665	-10.689	1.00	0.00	H
ATOM	1060	HB2	CYS	91	8.849	78.825	-7.870	1.00	0.00	H
ATOM	1061	HB3	CYS	91	9.204	79.855	-9.233	1.00	0.00	H
ATOM	1062	HG	CYS	91	7.195	78.923	-10.735	1.00	0.00	H
ATOM	1063	N	PHE	92	11.739	77.453	-9.074	1.00	3.48	N
ATOM	1064	CA	PHE	92	13.176	77.487	-9.293	1.00	3.48	C
ATOM	1065	C	PHE	92	13.588	76.418	-10.318	1.00	3.48	C
ATOM	1066	O	PHE	92	14.425	76.709	-11.168	1.00	3.48	O
ATOM	1067	CB	PHE	92	13.914	77.167	-7.966	1.00	3.48	C
ATOM	1068	CG	PHE	92	14.075	78.288	-6.942	1.00	3.48	C
ATOM	1069	CD1	PHE	92	13.045	79.217	-6.678	1.00	3.48	C
ATOM	1070	CD2	PHE	92	15.280	78.377	-6.210	1.00	3.48	C
ATOM	1071	CE1	PHE	92	13.211	80.208	-5.692	1.00	3.48	C
ATOM	1072	CE2	PHE	92	15.453	79.377	-5.234	1.00	3.48	C
ATOM	1073	CZ	PHE	92	14.416	80.291	-4.971	1.00	3.48	C
ATOM	1074	H	PHE	92	11.378	77.037	-8.224	1.00	0.00	H
ATOM	1075	HA	PHE	92	13.494	78.465	-9.660	1.00	0.00	H
ATOM	1076	HB2	PHE	92	13.445	76.320	-7.465	1.00	0.00	H
ATOM	1077	HB3	PHE	92	14.924	76.827	-8.208	1.00	0.00	H
ATOM	1078	HD1	PHE	92	12.109	79.181	-7.214	1.00	0.00	H
ATOM	1079	HD2	PHE	92	16.082	77.675	-6.389	1.00	0.00	H
ATOM	1080	HE1	PHE	92	12.409	80.902	-5.489	1.00	0.00	H
ATOM	1081	HE2	PHE	92	16.380	79.435	-4.682	1.00	0.00	H
ATOM	1082	HZ	PHE	92	14.545	81.052	-4.215	1.00	0.00	H
ATOM	1083	N	LEU	93	13.044	75.188	-10.229	1.00	3.92	N
ATOM	1084	CA	LEU	93	13.681	74.025	-10.849	1.00	3.92	C
ATOM	1085	C	LEU	93	12.773	73.019	-11.570	1.00	3.92	C
ATOM	1086	O	LEU	93	13.289	72.231	-12.360	1.00	3.92	O

ATOM	1087	CB	LEU	93	14.692	73.381	-9.869	1.00	3.92	C
ATOM	1088	CG	LEU	93	16.080	73.073	-10.471	1.00	3.92	C
ATOM	1089	CD1	LEU	93	16.905	74.344	-10.738	1.00	3.92	C
ATOM	1090	CD2	LEU	93	16.869	72.129	-9.555	1.00	3.92	C
ATOM	1091	H	LEU	93	12.378	75.019	-9.481	1.00	0.00	H
ATOM	1092	HA	LEU	93	14.263	74.393	-11.695	1.00	0.00	H
ATOM	1093	HB2	LEU	93	14.823	73.997	-8.979	1.00	0.00	H
ATOM	1094	HB3	LEU	93	14.259	72.451	-9.503	1.00	0.00	H
ATOM	1095	HG	LEU	93	15.939	72.557	-11.421	1.00	0.00	H
ATOM	1096	HD11	LEU	93	17.894	74.093	-11.121	1.00	0.00	H
ATOM	1097	HD12	LEU	93	16.436	74.989	-11.480	1.00	0.00	H
ATOM	1098	HD13	LEU	93	17.042	74.931	-9.830	1.00	0.00	H
ATOM	1099	HD21	LEU	93	17.822	71.846	-10.006	1.00	0.00	H
ATOM	1100	HD22	LEU	93	17.080	72.597	-8.594	1.00	0.00	H
ATOM	1101	HD23	LEU	93	16.322	71.205	-9.366	1.00	0.00	H
ATOM	1102	N	ALA	94	11.446	73.051	-11.408	1.00	4.49	N
ATOM	1103	CA	ALA	94	10.525	72.261	-12.245	1.00	4.49	C
ATOM	1104	C	ALA	94	10.113	73.056	-13.499	1.00	4.49	C
ATOM	1105	O	ALA	94	9.321	72.576	-14.302	1.00	4.49	O
ATOM	1106	CB	ALA	94	9.254	71.912	-11.458	1.00	4.49	C
ATOM	1107	H	ALA	94	11.035	73.781	-10.833	1.00	0.00	H
ATOM	1108	HA	ALA	94	10.988	71.326	-12.569	1.00	0.00	H
ATOM	1109	HB1	ALA	94	8.621	71.233	-12.033	1.00	0.00	H
ATOM	1110	HB2	ALA	94	9.491	71.400	-10.526	1.00	0.00	H
ATOM	1111	HB3	ALA	94	8.646	72.787	-11.236	1.00	0.00	H
ATOM	1112	N	ARG	95	10.666	74.267	-13.676	1.00	5.03	N
ATOM	1113	CA	ARG	95	10.496	75.176	-14.815	1.00	5.03	C
ATOM	1114	C	ARG	95	9.144	75.930	-14.746	1.00	5.03	C
ATOM	1115	O	ARG	95	8.661	76.443	-15.755	1.00	5.03	O
ATOM	1116	CB	ARG	95	10.762	74.506	-16.198	1.00	5.03	C
ATOM	1117	CG	ARG	95	11.851	73.405	-16.320	1.00	5.03	C
ATOM	1118	CD	ARG	95	13.263	73.739	-15.803	1.00	5.03	C
ATOM	1119	NE	ARG	95	14.261	72.810	-16.380	1.00	5.03	N
ATOM	1120	CZ	ARG	95	15.009	71.879	-15.758	1.00	5.03	C
ATOM	1121	NH1	ARG	95	14.926	71.672	-14.446	1.00	5.03	N
ATOM	1122	NH2	ARG	95	15.853	71.132	-16.468	1.00	5.03	N1+
ATOM	1123	H	ARG	95	11.226	74.601	-12.906	1.00	0.00	H
ATOM	1124	HA	ARG	95	11.260	75.943	-14.679	1.00	0.00	H
ATOM	1125	HB2	ARG	95	9.828	74.062	-16.547	1.00	0.00	H
ATOM	1126	HB3	ARG	95	10.978	75.296	-16.919	1.00	0.00	H
ATOM	1127	HG2	ARG	95	11.509	72.497	-15.823	1.00	0.00	H
ATOM	1128	HG3	ARG	95	11.910	73.125	-17.373	1.00	0.00	H
ATOM	1129	HD2	ARG	95	13.537	74.749	-16.110	1.00	0.00	H
ATOM	1130	HD3	ARG	95	13.293	73.730	-14.715	1.00	0.00	H
ATOM	1131	HE	ARG	95	14.370	72.915	-17.379	1.00	0.00	H
ATOM	1132	HH12	ARG	95	15.515	71.001	-13.929	1.00	0.00	H
ATOM	1133	HH11	ARG	95	14.222	72.100	-13.845	1.00	0.00	H
ATOM	1134	HH22	ARG	95	16.347	70.350	-16.030	1.00	0.00	H
ATOM	1135	HH21	ARG	95	15.956	71.183	-17.471	1.00	0.00	H
ATOM	1136	N	GLU	96	8.508	75.939	-13.568	1.00	4.85	N
ATOM	1137	CA	GLU	96	7.099	76.266	-13.347	1.00	4.85	C
ATOM	1138	C	GLU	96	6.915	77.808	-13.283	1.00	4.85	C
ATOM	1139	O	GLU	96	7.748	78.496	-12.685	1.00	4.85	O
ATOM	1140	CB	GLU	96	6.724	75.582	-12.006	1.00	4.85	C
ATOM	1141	CG	GLU	96	5.434	74.741	-12.036	1.00	4.85	C
ATOM	1142	CD	GLU	96	4.205	75.479	-11.549	1.00	4.85	C
ATOM	1143	OE1	GLU	96	4.190	76.726	-11.495	1.00	4.85	O
ATOM	1144	OE2	GLU	96	3.201	74.820	-11.202	1.00	4.85	O1-
ATOM	1145	H	GLU	96	9.007	75.557	-12.777	1.00	0.00	H
ATOM	1146	HA	GLU	96	6.539	75.825	-14.169	1.00	0.00	H
ATOM	1147	HB2	GLU	96	7.496	74.865	-11.740	1.00	0.00	H
ATOM	1148	HB3	GLU	96	6.714	76.296	-11.178	1.00	0.00	H
ATOM	1149	HG2	GLU	96	5.243	74.337	-13.030	1.00	0.00	H

ATOM	1150	HG3	GLU	96	5.552	73.872	-11.388	1.00	0.00	H
ATOM	1151	N	PRO	97	5.875	78.387	-13.929	1.00	4.69	N
ATOM	1152	CA	PRO	97	5.751	79.842	-14.100	1.00	4.69	C
ATOM	1153	C	PRO	97	5.371	80.611	-12.816	1.00	4.69	C
ATOM	1154	O	PRO	97	4.246	80.525	-12.316	1.00	4.69	O
ATOM	1155	CB	PRO	97	4.703	80.010	-15.214	1.00	4.69	C
ATOM	1156	CG	PRO	97	3.849	78.756	-15.144	1.00	4.69	C
ATOM	1157	CD	PRO	97	4.810	77.679	-14.644	1.00	4.69	C
ATOM	1158	HA	PRO	97	6.704	80.227	-14.469	1.00	0.00	H
ATOM	1159	HB2	PRO	97	4.109	80.917	-15.126	1.00	0.00	H
ATOM	1160	HB3	PRO	97	5.210	80.048	-16.180	1.00	0.00	H
ATOM	1161	HG2	PRO	97	3.057	78.898	-14.408	1.00	0.00	H
ATOM	1162	HG3	PRO	97	3.382	78.499	-16.096	1.00	0.00	H
ATOM	1163	HD3	PRO	97	5.246	77.151	-15.494	1.00	0.00	H
ATOM	1164	HD2	PRO	97	4.300	76.946	-14.020	1.00	0.00	H
ATOM	1165	N	TRP	98	6.307	81.458	-12.361	1.00	4.88	N
ATOM	1166	CA	TRP	98	6.295	82.335	-11.180	1.00	4.88	C
ATOM	1167	C	TRP	98	5.033	83.185	-10.957	1.00	4.88	C
ATOM	1168	O	TRP	98	4.736	83.552	-9.824	1.00	4.88	O
ATOM	1169	CB	TRP	98	7.547	83.237	-11.186	1.00	4.88	C
ATOM	1170	CG	TRP	98	8.857	82.582	-10.865	1.00	4.88	C
ATOM	1171	CD1	TRP	98	9.458	81.614	-11.594	1.00	4.88	C
ATOM	1172	CD2	TRP	98	9.738	82.828	-9.724	1.00	4.88	C
ATOM	1173	NE1	TRP	98	10.635	81.245	-10.982	1.00	4.88	N
ATOM	1174	CE2	TRP	98	10.870	81.965	-9.833	1.00	4.88	C
ATOM	1175	CE3	TRP	98	9.694	83.685	-8.598	1.00	4.88	C
ATOM	1176	CZ2	TRP	98	11.902	81.949	-8.880	1.00	4.88	C
ATOM	1177	CZ3	TRP	98	10.726	83.683	-7.636	1.00	4.88	C
ATOM	1178	CH2	TRP	98	11.830	82.820	-7.778	1.00	4.88	C
ATOM	1179	H	TRP	98	7.205	81.377	-12.813	1.00	0.00	H
ATOM	1180	HA	TRP	98	6.360	81.687	-10.305	1.00	0.00	H
ATOM	1181	HB2	TRP	98	7.644	83.744	-12.147	1.00	0.00	H
ATOM	1182	HB3	TRP	98	7.424	84.037	-10.454	1.00	0.00	H
ATOM	1183	HD1	TRP	98	9.067	81.174	-12.499	1.00	0.00	H
ATOM	1184	HE1	TRP	98	11.164	80.419	-11.275	1.00	0.00	H
ATOM	1185	HE3	TRP	98	8.854	84.350	-8.470	1.00	0.00	H
ATOM	1186	HZ2	TRP	98	12.741	81.277	-8.990	1.00	0.00	H
ATOM	1187	HZ3	TRP	98	10.670	84.347	-6.785	1.00	0.00	H
ATOM	1188	HH2	TRP	98	12.621	82.822	-7.042	1.00	0.00	H
ATOM	1189	N	ILE	99	4.262	83.475	-12.011	1.00	4.60	N
ATOM	1190	CA	ILE	99	2.994	84.208	-11.953	1.00	4.60	C
ATOM	1191	C	ILE	99	1.885	83.463	-11.170	1.00	4.60	C
ATOM	1192	O	ILE	99	0.867	84.067	-10.835	1.00	4.60	O
ATOM	1193	CB	ILE	99	2.531	84.548	-13.397	1.00	4.60	C
ATOM	1194	CG1	ILE	99	2.421	83.309	-14.320	1.00	4.60	C
ATOM	1195	CG2	ILE	99	3.447	85.622	-14.020	1.00	4.60	C
ATOM	1196	CD1	ILE	99	1.607	83.557	-15.598	1.00	4.60	C
ATOM	1197	H	ILE	99	4.544	83.090	-12.898	1.00	0.00	H
ATOM	1198	HA	ILE	99	3.167	85.139	-11.409	1.00	0.00	H
ATOM	1199	HB	ILE	99	1.537	84.994	-13.314	1.00	0.00	H
ATOM	1200	HG12	ILE	99	3.418	82.970	-14.593	1.00	0.00	H
ATOM	1201	HG13	ILE	99	1.958	82.480	-13.784	1.00	0.00	H
ATOM	1202	HG21	ILE	99	3.066	85.973	-14.978	1.00	0.00	H
ATOM	1203	HG22	ILE	99	3.523	86.495	-13.371	1.00	0.00	H
ATOM	1204	HG23	ILE	99	4.458	85.248	-14.183	1.00	0.00	H
ATOM	1205	HD11	ILE	99	1.521	82.641	-16.183	1.00	0.00	H
ATOM	1206	HD12	ILE	99	0.599	83.898	-15.363	1.00	0.00	H
ATOM	1207	HD13	ILE	99	2.076	84.306	-16.236	1.00	0.00	H
ATOM	1208	N	LYS	100	2.071	82.169	-10.874	1.00	3.74	N
ATOM	1209	CA	LYS	100	1.180	81.403	-10.008	1.00	3.74	C
ATOM	1210	C	LYS	100	1.583	81.529	-8.529	1.00	3.74	C
ATOM	1211	O	LYS	100	0.709	81.532	-7.664	1.00	3.74	O
ATOM	1212	CB	LYS	100	1.291	79.910	-10.380	1.00	3.74	C

ATOM	1213	CG	LYS	100	0.880	79.602	-11.833	1.00	3.74	C
ATOM	1214	CD	LYS	100	0.153	78.258	-12.007	1.00	3.74	C
ATOM	1215	CE	LYS	100	1.044	77.004	-11.951	1.00	3.74	C
ATOM	1216	NZ	LYS	100	1.660	76.773	-10.639	1.00	3.74	N1+
ATOM	1217	H	LYS	100	2.910	81.717	-11.225	1.00	0.00	H
ATOM	1218	HA	LYS	100	0.149	81.740	-10.119	1.00	0.00	H
ATOM	1219	HB2	LYS	100	2.307	79.547	-10.207	1.00	0.00	H
ATOM	1220	HB3	LYS	100	0.654	79.351	-9.693	1.00	0.00	H
ATOM	1221	HG2	LYS	100	0.212	80.390	-12.180	1.00	0.00	H
ATOM	1222	HG3	LYS	100	1.751	79.656	-12.487	1.00	0.00	H
ATOM	1223	HD2	LYS	100	-0.659	78.181	-11.287	1.00	0.00	H
ATOM	1224	HD3	LYS	100	-0.323	78.271	-12.987	1.00	0.00	H
ATOM	1225	HE2	LYS	100	0.460	76.116	-12.196	1.00	0.00	H
ATOM	1226	HE3	LYS	100	1.837	77.066	-12.698	1.00	0.00	H
ATOM	1227	HZ1	LYS	100	2.218	75.901	-10.770	1.00	0.00	H
ATOM	1228	HZ2	LYS	100	2.430	77.416	-10.494	1.00	0.00	H
ATOM	1229	HZ3	LYS	100	1.049	76.669	-9.847	1.00	0.00	H
ATOM	1230	N	LEU	101	2.893	81.599	-8.252	1.00	3.28	N
ATOM	1231	CA	LEU	101	3.554	81.504	-6.946	1.00	3.28	C
ATOM	1232	C	LEU	101	2.863	82.178	-5.736	1.00	3.28	C
ATOM	1233	O	LEU	101	2.487	81.464	-4.799	1.00	3.28	O
ATOM	1234	CB	LEU	101	5.073	81.780	-7.085	1.00	3.28	C
ATOM	1235	CG	LEU	101	5.941	81.635	-5.808	1.00	3.28	C
ATOM	1236	CD1	LEU	101	7.327	81.080	-6.167	1.00	3.28	C
ATOM	1237	CD2	LEU	101	6.110	82.960	-5.042	1.00	3.28	C
ATOM	1238	H	LEU	101	3.513	81.719	-9.040	1.00	0.00	H
ATOM	1239	HA	LEU	101	3.487	80.440	-6.718	1.00	0.00	H
ATOM	1240	HB2	LEU	101	5.446	81.076	-7.830	1.00	0.00	H
ATOM	1241	HB3	LEU	101	5.255	82.760	-7.519	1.00	0.00	H
ATOM	1242	HG	LEU	101	5.478	80.910	-5.137	1.00	0.00	H
ATOM	1243	HD11	LEU	101	7.970	81.019	-5.288	1.00	0.00	H
ATOM	1244	HD12	LEU	101	7.245	80.075	-6.580	1.00	0.00	H
ATOM	1245	HD13	LEU	101	7.836	81.704	-6.904	1.00	0.00	H
ATOM	1246	HD21	LEU	101	6.771	82.829	-4.184	1.00	0.00	H
ATOM	1247	HD22	LEU	101	6.550	83.733	-5.673	1.00	0.00	H
ATOM	1248	HD23	LEU	101	5.170	83.341	-4.651	1.00	0.00	H
ATOM	1249	N	PRO	102	2.653	83.517	-5.727	1.00	3.08	N
ATOM	1250	CA	PRO	102	2.080	84.206	-4.561	1.00	3.08	C
ATOM	1251	C	PRO	102	0.597	83.880	-4.343	1.00	3.08	C
ATOM	1252	O	PRO	102	0.143	83.763	-3.206	1.00	3.08	O
ATOM	1253	CB	PRO	102	2.319	85.699	-4.821	1.00	3.08	C
ATOM	1254	CG	PRO	102	2.429	85.823	-6.335	1.00	3.08	C
ATOM	1255	CD	PRO	102	2.971	84.469	-6.798	1.00	3.08	C
ATOM	1256	HA	PRO	102	2.620	83.909	-3.659	1.00	0.00	H
ATOM	1257	HB2	PRO	102	1.545	86.344	-4.401	1.00	0.00	H
ATOM	1258	HB3	PRO	102	3.264	85.995	-4.363	1.00	0.00	H
ATOM	1259	HG2	PRO	102	1.437	85.985	-6.759	1.00	0.00	H
ATOM	1260	HG3	PRO	102	3.058	86.658	-6.647	1.00	0.00	H
ATOM	1261	HD3	PRO	102	4.052	84.533	-6.916	1.00	0.00	H
ATOM	1262	HD2	PRO	102	2.542	84.189	-7.760	1.00	0.00	H
ATOM	1263	N	ILE	103	-0.140	83.676	-5.437	1.00	2.66	N
ATOM	1264	CA	ILE	103	-1.561	83.358	-5.438	1.00	2.66	C
ATOM	1265	C	ILE	103	-1.811	81.906	-5.032	1.00	2.66	C
ATOM	1266	O	ILE	103	-2.860	81.600	-4.470	1.00	2.66	O
ATOM	1267	CB	ILE	103	-2.127	83.732	-6.843	1.00	2.66	C
ATOM	1268	CG1	ILE	103	-2.033	85.270	-7.051	1.00	2.66	C
ATOM	1269	CG2	ILE	103	-3.575	83.251	-7.080	1.00	2.66	C
ATOM	1270	CD1	ILE	103	-2.207	85.748	-8.498	1.00	2.66	C
ATOM	1271	H	ILE	103	0.323	83.720	-6.332	1.00	0.00	H
ATOM	1272	HA	ILE	103	-2.059	83.978	-4.688	1.00	0.00	H
ATOM	1273	HB	ILE	103	-1.497	83.245	-7.589	1.00	0.00	H
ATOM	1274	HG12	ILE	103	-2.764	85.769	-6.413	1.00	0.00	H
ATOM	1275	HG13	ILE	103	-1.066	85.644	-6.718	1.00	0.00	H

ATOM	1276	HG21	ILE	103	-3.969	83.586	-8.037	1.00	0.00	H
ATOM	1277	HG22	ILE	103	-3.638	82.163	-7.083	1.00	0.00	H
ATOM	1278	HG23	ILE	103	-4.244	83.618	-6.299	1.00	0.00	H
ATOM	1279	HD11	ILE	103	-2.038	86.823	-8.565	1.00	0.00	H
ATOM	1280	HD12	ILE	103	-1.490	85.266	-9.164	1.00	0.00	H
ATOM	1281	HD13	ILE	103	-3.213	85.561	-8.869	1.00	0.00	H
ATOM	1282	N	TYR	104	-0.828	81.033	-5.255	1.00	2.40	N
ATOM	1283	CA	TYR	104	-0.822	79.698	-4.696	1.00	2.40	C
ATOM	1284	C	TYR	104	-0.580	79.711	-3.193	1.00	2.40	C
ATOM	1285	O	TYR	104	-1.342	79.110	-2.445	1.00	2.40	O
ATOM	1286	CB	TYR	104	0.232	78.809	-5.405	1.00	2.40	C
ATOM	1287	CG	TYR	104	-0.254	78.033	-6.628	1.00	2.40	C
ATOM	1288	CD1	TYR	104	-1.134	78.613	-7.567	1.00	2.40	C
ATOM	1289	CD2	TYR	104	0.168	76.698	-6.817	1.00	2.40	C
ATOM	1290	CE1	TYR	104	-1.581	77.866	-8.673	1.00	2.40	C
ATOM	1291	CE2	TYR	104	-0.271	75.954	-7.929	1.00	2.40	C
ATOM	1292	CZ	TYR	104	-1.139	76.544	-8.867	1.00	2.40	C
ATOM	1293	OH	TYR	104	-1.524	75.862	-9.986	1.00	2.40	O
ATOM	1294	H	TYR	104	-0.017	81.339	-5.779	1.00	0.00	H
ATOM	1295	HA	TYR	104	-1.802	79.238	-4.841	1.00	0.00	H
ATOM	1296	HB2	TYR	104	1.097	79.402	-5.698	1.00	0.00	H
ATOM	1297	HB3	TYR	104	0.617	78.070	-4.696	1.00	0.00	H
ATOM	1298	HD1	TYR	104	-1.475	79.629	-7.438	1.00	0.00	H
ATOM	1299	HD2	TYR	104	0.818	76.225	-6.092	1.00	0.00	H
ATOM	1300	HE1	TYR	104	-2.264	78.317	-9.372	1.00	0.00	H
ATOM	1301	HE2	TYR	104	0.062	74.928	-8.032	1.00	0.00	H
ATOM	1302	HH	TYR	104	-1.256	74.932	-9.947	1.00	0.00	H
ATOM	1303	N	THR	105	0.424	80.458	-2.746	1.00	2.51	N
ATOM	1304	CA	THR	105	0.824	80.489	-1.347	1.00	2.51	C
ATOM	1305	C	THR	105	-0.258	81.082	-0.437	1.00	2.51	C
ATOM	1306	O	THR	105	-0.527	80.570	0.652	1.00	2.51	O
ATOM	1307	CB	THR	105	2.122	81.347	-1.274	1.00	2.51	C
ATOM	1308	OG1	THR	105	3.146	80.768	-2.069	1.00	2.51	O
ATOM	1309	CG2	THR	105	2.710	81.508	0.138	1.00	2.51	C
ATOM	1310	H	THR	105	0.998	80.950	-3.414	1.00	0.00	H
ATOM	1311	HA	THR	105	1.043	79.483	-0.997	1.00	0.00	H
ATOM	1312	HB	THR	105	1.926	82.346	-1.668	1.00	0.00	H
ATOM	1313	HG1	THR	105	2.879	80.755	-2.981	1.00	0.00	H
ATOM	1314	HG21	THR	105	3.677	82.011	0.109	1.00	0.00	H
ATOM	1315	HG22	THR	105	2.060	82.099	0.783	1.00	0.00	H
ATOM	1316	HG23	THR	105	2.851	80.543	0.619	1.00	0.00	H
ATOM	1317	N	LEU	106	-0.918	82.137	-0.920	1.00	2.62	N
ATOM	1318	CA	LEU	106	-2.029	82.774	-0.239	1.00	2.62	C
ATOM	1319	C	LEU	106	-3.251	81.857	-0.183	1.00	2.62	C
ATOM	1320	O	LEU	106	-3.944	81.818	0.830	1.00	2.62	O
ATOM	1321	CB	LEU	106	-2.357	84.084	-0.999	1.00	2.62	C
ATOM	1322	CG	LEU	106	-3.408	85.009	-0.335	1.00	2.62	C
ATOM	1323	CD1	LEU	106	-3.027	86.489	-0.508	1.00	2.62	C
ATOM	1324	CD2	LEU	106	-4.833	84.777	-0.872	1.00	2.62	C
ATOM	1325	H	LEU	106	-0.620	82.530	-1.806	1.00	0.00	H
ATOM	1326	HA	LEU	106	-1.727	83.026	0.779	1.00	0.00	H
ATOM	1327	HB2	LEU	106	-1.421	84.639	-1.082	1.00	0.00	H
ATOM	1328	HB3	LEU	106	-2.647	83.866	-2.028	1.00	0.00	H
ATOM	1329	HG	LEU	106	-3.415	84.809	0.739	1.00	0.00	H
ATOM	1330	HD11	LEU	106	-3.751	87.141	-0.018	1.00	0.00	H
ATOM	1331	HD12	LEU	106	-2.053	86.700	-0.065	1.00	0.00	H
ATOM	1332	HD13	LEU	106	-2.984	86.770	-1.560	1.00	0.00	H
ATOM	1333	HD21	LEU	106	-5.545	85.447	-0.389	1.00	0.00	H
ATOM	1334	HD22	LEU	106	-4.890	84.952	-1.946	1.00	0.00	H
ATOM	1335	HD23	LEU	106	-5.185	83.763	-0.683	1.00	0.00	H
ATOM	1336	N	ALA	107	-3.483	81.084	-1.252	1.00	2.47	N
ATOM	1337	CA	ALA	107	-4.522	80.070	-1.295	1.00	2.47	C
ATOM	1338	C	ALA	107	-4.276	78.954	-0.274	1.00	2.47	C

ATOM	1339	O	ALA	107	-5.178	78.581	0.473	1.00	2.47	O
ATOM	1340	CB	ALA	107	-4.628	79.487	-2.713	1.00	2.47	C
ATOM	1341	H	ALA	107	-2.842	81.134	-2.029	1.00	0.00	H
ATOM	1342	HA	ALA	107	-5.472	80.541	-1.037	1.00	0.00	H
ATOM	1343	HB1	ALA	107	-5.252	78.598	-2.709	1.00	0.00	H
ATOM	1344	HB2	ALA	107	-5.069	80.208	-3.399	1.00	0.00	H
ATOM	1345	HB3	ALA	107	-3.670	79.182	-3.122	1.00	0.00	H
ATOM	1346	N	GLN	108	-3.036	78.463	-0.222	1.00	2.46	N
ATOM	1347	CA	GLN	108	-2.601	77.400	0.669	1.00	2.46	C
ATOM	1348	C	GLN	108	-2.721	77.778	2.150	1.00	2.46	C
ATOM	1349	O	GLN	108	-3.320	77.042	2.934	1.00	2.46	O
ATOM	1350	CB	GLN	108	-1.154	77.000	0.296	1.00	2.46	C
ATOM	1351	CG	GLN	108	-1.045	76.290	-1.081	1.00	2.46	C
ATOM	1352	CD	GLN	108	0.328	76.401	-1.748	1.00	2.46	C
ATOM	1353	OE1	GLN	108	1.216	77.086	-1.259	1.00	2.46	O
ATOM	1354	NE2	GLN	108	0.541	75.742	-2.879	1.00	2.46	N
ATOM	1355	H	GLN	108	-2.362	78.806	-0.900	1.00	0.00	H
ATOM	1356	HA	GLN	108	-3.251	76.539	0.506	1.00	0.00	H
ATOM	1357	HB2	GLN	108	-0.524	77.889	0.329	1.00	0.00	H
ATOM	1358	HB3	GLN	108	-0.750	76.325	1.052	1.00	0.00	H
ATOM	1359	HG2	GLN	108	-1.301	75.239	-0.974	1.00	0.00	H
ATOM	1360	HG3	GLN	108	-1.770	76.703	-1.776	1.00	0.00	H
ATOM	1361	HE22	GLN	108	1.462	75.801	-3.307	1.00	0.00	H
ATOM	1362	HE21	GLN	108	-0.212	75.184	-3.327	1.00	0.00	H
ATOM	1363	N	THR	109	-2.193	78.947	2.523	1.00	2.39	N
ATOM	1364	CA	THR	109	-2.255	79.468	3.886	1.00	2.39	C
ATOM	1365	C	THR	109	-3.678	79.756	4.377	1.00	2.39	C
ATOM	1366	O	THR	109	-4.019	79.422	5.514	1.00	2.39	O
ATOM	1367	CB	THR	109	-1.314	80.695	4.019	1.00	2.39	C
ATOM	1368	OG1	THR	109	-1.532	81.647	2.996	1.00	2.39	O
ATOM	1369	CG2	THR	109	0.176	80.321	4.001	1.00	2.39	C
ATOM	1370	H	THR	109	-1.741	79.513	1.816	1.00	0.00	H
ATOM	1371	HA	THR	109	-1.873	78.692	4.553	1.00	0.00	H
ATOM	1372	HB	THR	109	-1.521	81.191	4.970	1.00	0.00	H
ATOM	1373	HG1	THR	109	-1.179	81.300	2.179	1.00	0.00	H
ATOM	1374	HG21	THR	109	0.804	81.210	4.075	1.00	0.00	H
ATOM	1375	HG22	THR	109	0.424	79.685	4.848	1.00	0.00	H
ATOM	1376	HG23	THR	109	0.456	79.789	3.091	1.00	0.00	H
ATOM	1377	N	LEU	110	-4.524	80.291	3.495	1.00	2.26	N
ATOM	1378	CA	LEU	110	-5.932	80.523	3.761	1.00	2.26	C
ATOM	1379	C	LEU	110	-6.710	79.211	3.932	1.00	2.26	C
ATOM	1380	O	LEU	110	-7.549	79.103	4.825	1.00	2.26	O
ATOM	1381	CB	LEU	110	-6.503	81.364	2.592	1.00	2.26	C
ATOM	1382	CG	LEU	110	-8.010	81.718	2.660	1.00	2.26	C
ATOM	1383	CD1	LEU	110	-8.251	83.195	2.309	1.00	2.26	C
ATOM	1384	CD2	LEU	110	-8.845	80.817	1.733	1.00	2.26	C
ATOM	1385	H	LEU	110	-4.158	80.586	2.596	1.00	0.00	H
ATOM	1386	HA	LEU	110	-6.024	81.096	4.686	1.00	0.00	H
ATOM	1387	HB2	LEU	110	-5.927	82.290	2.560	1.00	0.00	H
ATOM	1388	HB3	LEU	110	-6.291	80.869	1.643	1.00	0.00	H
ATOM	1389	HG	LEU	110	-8.361	81.577	3.685	1.00	0.00	H
ATOM	1390	HD11	LEU	110	-9.309	83.448	2.373	1.00	0.00	H
ATOM	1391	HD12	LEU	110	-7.718	83.854	2.995	1.00	0.00	H
ATOM	1392	HD13	LEU	110	-7.912	83.423	1.298	1.00	0.00	H
ATOM	1393	HD21	LEU	110	-9.907	81.048	1.810	1.00	0.00	H
ATOM	1394	HD22	LEU	110	-8.554	80.943	0.690	1.00	0.00	H
ATOM	1395	HD23	LEU	110	-8.724	79.762	1.979	1.00	0.00	H
ATOM	1396	N	GLY	111	-6.387	78.196	3.124	1.00	2.10	N
ATOM	1397	CA	GLY	111	-6.994	76.875	3.197	1.00	2.10	C
ATOM	1398	C	GLY	111	-6.689	76.168	4.517	1.00	2.10	C
ATOM	1399	O	GLY	111	-7.584	75.611	5.151	1.00	2.10	O
ATOM	1400	H	GLY	111	-5.700	78.362	2.395	1.00	0.00	H
ATOM	1401	HA2	GLY	111	-8.074	76.947	3.056	1.00	0.00	H

ATOM	1402	HA3	GLY	111	-6.599	76.267	2.385	1.00	0.00	H
ATOM	1403	N	ALA	112	-5.435	76.262	4.965	1.00	1.90	N
ATOM	1404	CA	ALA	112	-4.963	75.661	6.205	1.00	1.90	C
ATOM	1405	C	ALA	112	-5.485	76.369	7.456	1.00	1.90	C
ATOM	1406	O	ALA	112	-5.695	75.728	8.480	1.00	1.90	O
ATOM	1407	CB	ALA	112	-3.434	75.735	6.184	1.00	1.90	C
ATOM	1408	H	ALA	112	-4.758	76.741	4.381	1.00	0.00	H
ATOM	1409	HA	ALA	112	-5.265	74.617	6.216	1.00	0.00	H
ATOM	1410	HB1	ALA	112	-3.011	75.155	7.002	1.00	0.00	H
ATOM	1411	HB2	ALA	112	-3.025	75.321	5.265	1.00	0.00	H
ATOM	1412	HB3	ALA	112	-3.076	76.762	6.269	1.00	0.00	H
ATOM	1413	N	PHE	113	-5.722	77.680	7.365	1.00	1.80	N
ATOM	1414	CA	PHE	113	-6.350	78.471	8.416	1.00	1.80	C
ATOM	1415	C	PHE	113	-7.817	78.062	8.599	1.00	1.80	C
ATOM	1416	O	PHE	113	-8.249	77.736	9.704	1.00	1.80	O
ATOM	1417	CB	PHE	113	-6.234	79.967	8.033	1.00	1.80	C
ATOM	1418	CG	PHE	113	-6.927	80.958	8.956	1.00	1.80	C
ATOM	1419	CD1	PHE	113	-6.290	81.398	10.133	1.00	1.80	C
ATOM	1420	CD2	PHE	113	-8.214	81.446	8.640	1.00	1.80	C
ATOM	1421	CE1	PHE	113	-6.935	82.306	10.991	1.00	1.80	C
ATOM	1422	CE2	PHE	113	-8.861	82.353	9.500	1.00	1.80	C
ATOM	1423	CZ	PHE	113	-8.222	82.784	10.678	1.00	1.80	C
ATOM	1424	H	PHE	113	-5.484	78.144	6.498	1.00	0.00	H
ATOM	1425	HA	PHE	113	-5.830	78.301	9.361	1.00	0.00	H
ATOM	1426	HB2	PHE	113	-5.179	80.242	7.987	1.00	0.00	H
ATOM	1427	HB3	PHE	113	-6.617	80.129	7.026	1.00	0.00	H
ATOM	1428	HD1	PHE	113	-5.306	81.043	10.390	1.00	0.00	H
ATOM	1429	HD2	PHE	113	-8.716	81.120	7.741	1.00	0.00	H
ATOM	1430	HE1	PHE	113	-6.438	82.633	11.892	1.00	0.00	H
ATOM	1431	HE2	PHE	113	-9.849	82.717	9.260	1.00	0.00	H
ATOM	1432	HZ	PHE	113	-8.717	83.479	11.341	1.00	0.00	H
ATOM	1433	N	LEU	114	-8.576	78.014	7.499	1.00	1.87	N
ATOM	1434	CA	LEU	114	-10.003	77.723	7.545	1.00	1.87	C
ATOM	1435	C	LEU	114	-10.322	76.256	7.863	1.00	1.87	C
ATOM	1436	O	LEU	114	-11.393	75.956	8.386	1.00	1.87	O
ATOM	1437	CB	LEU	114	-10.640	78.079	6.183	1.00	1.87	C
ATOM	1438	CG	LEU	114	-10.674	79.588	5.847	1.00	1.87	C
ATOM	1439	CD1	LEU	114	-11.080	79.799	4.382	1.00	1.87	C
ATOM	1440	CD2	LEU	114	-11.603	80.384	6.779	1.00	1.87	C
ATOM	1441	H	LEU	114	-8.167	78.271	6.607	1.00	0.00	H
ATOM	1442	HA	LEU	114	-10.464	78.318	8.335	1.00	0.00	H
ATOM	1443	HB2	LEU	114	-10.096	77.549	5.399	1.00	0.00	H
ATOM	1444	HB3	LEU	114	-11.661	77.694	6.143	1.00	0.00	H
ATOM	1445	HG	LEU	114	-9.669	79.993	5.958	1.00	0.00	H
ATOM	1446	HD11	LEU	114	-11.047	80.855	4.113	1.00	0.00	H
ATOM	1447	HD12	LEU	114	-10.403	79.269	3.712	1.00	0.00	H
ATOM	1448	HD13	LEU	114	-12.090	79.435	4.192	1.00	0.00	H
ATOM	1449	HD21	LEU	114	-11.642	81.435	6.490	1.00	0.00	H
ATOM	1450	HD22	LEU	114	-12.622	79.997	6.753	1.00	0.00	H
ATOM	1451	HD23	LEU	114	-11.263	80.356	7.814	1.00	0.00	H
ATOM	1452	N	GLY	115	-9.365	75.357	7.635	1.00	1.84	N
ATOM	1453	CA	GLY	115	-9.498	73.950	7.971	1.00	1.84	C
ATOM	1454	C	GLY	115	-8.935	73.639	9.354	1.00	1.84	C
ATOM	1455	O	GLY	115	-9.089	72.518	9.826	1.00	1.84	O
ATOM	1456	H	GLY	115	-8.522	75.658	7.161	1.00	0.00	H
ATOM	1457	HA2	GLY	115	-10.517	73.598	7.842	1.00	0.00	H
ATOM	1458	HA3	GLY	115	-8.908	73.390	7.251	1.00	0.00	H
ATOM	1459	N	ALA	116	-8.339	74.631	10.028	1.00	1.72	N
ATOM	1460	CA	ALA	116	-8.076	74.576	11.455	1.00	1.72	C
ATOM	1461	C	ALA	116	-9.313	75.050	12.221	1.00	1.72	C
ATOM	1462	O	ALA	116	-9.735	74.396	13.174	1.00	1.72	O
ATOM	1463	CB	ALA	116	-6.898	75.500	11.808	1.00	1.72	C
ATOM	1464	H	ALA	116	-8.228	75.525	9.569	1.00	0.00	H

ATOM	1465	HA	ALA	116	-7.831	73.556	11.751	1.00	0.00	H
ATOM	1466	HB1	ALA	116	-6.648	75.418	12.868	1.00	0.00	H
ATOM	1467	HB2	ALA	116	-6.008	75.227	11.244	1.00	0.00	H
ATOM	1468	HB3	ALA	116	-7.108	76.551	11.613	1.00	0.00	H
ATOM	1469	N	GLY	117	-9.942	76.134	11.743	1.00	1.93	N
ATOM	1470	CA	GLY	117	-11.114	76.733	12.372	1.00	1.93	C
ATOM	1471	C	GLY	117	-12.337	75.824	12.339	1.00	1.93	C
ATOM	1472	O	GLY	117	-13.104	75.801	13.298	1.00	1.93	O
ATOM	1473	H	GLY	117	-9.497	76.648	10.993	1.00	0.00	H
ATOM	1474	HA2	GLY	117	-10.880	76.936	13.414	1.00	0.00	H
ATOM	1475	HA3	GLY	117	-11.350	77.684	11.895	1.00	0.00	H
ATOM	1476	N	ILE	118	-12.486	75.016	11.280	1.00	2.15	N
ATOM	1477	CA	ILE	118	-13.576	74.056	11.188	1.00	2.15	C
ATOM	1478	C	ILE	118	-13.373	72.842	12.104	1.00	2.15	C
ATOM	1479	O	ILE	118	-14.331	72.391	12.725	1.00	2.15	O
ATOM	1480	CB	ILE	118	-13.794	73.640	9.703	1.00	2.15	C
ATOM	1481	CG1	ILE	118	-15.165	72.972	9.437	1.00	2.15	C
ATOM	1482	CG2	ILE	118	-12.682	72.730	9.166	1.00	2.15	C
ATOM	1483	CD1	ILE	118	-16.375	73.895	9.641	1.00	2.15	C
ATOM	1484	H	ILE	118	-11.855	75.116	10.498	1.00	0.00	H
ATOM	1485	HA	ILE	118	-14.477	74.566	11.532	1.00	0.00	H
ATOM	1486	HB	ILE	118	-13.769	74.555	9.108	1.00	0.00	H
ATOM	1487	HG12	ILE	118	-15.190	72.619	8.405	1.00	0.00	H
ATOM	1488	HG13	ILE	118	-15.282	72.080	10.055	1.00	0.00	H
ATOM	1489	HG21	ILE	118	-12.674	72.710	8.077	1.00	0.00	H
ATOM	1490	HG22	ILE	118	-11.712	73.066	9.517	1.00	0.00	H
ATOM	1491	HG23	ILE	118	-12.803	71.707	9.520	1.00	0.00	H
ATOM	1492	HD11	ILE	118	-17.292	73.398	9.323	1.00	0.00	H
ATOM	1493	HD12	ILE	118	-16.509	74.164	10.688	1.00	0.00	H
ATOM	1494	HD13	ILE	118	-16.280	74.814	9.063	1.00	0.00	H
ATOM	1495	N	VAL	119	-12.135	72.350	12.240	1.00	2.16	N
ATOM	1496	CA	VAL	119	-11.795	71.248	13.136	1.00	2.16	C
ATOM	1497	C	VAL	119	-12.046	71.652	14.593	1.00	2.16	C
ATOM	1498	O	VAL	119	-12.660	70.911	15.357	1.00	2.16	O
ATOM	1499	CB	VAL	119	-10.326	70.800	12.882	1.00	2.16	C
ATOM	1500	CG1	VAL	119	-9.724	69.894	13.977	1.00	2.16	C
ATOM	1501	CG2	VAL	119	-10.205	70.081	11.529	1.00	2.16	C
ATOM	1502	H	VAL	119	-11.382	72.795	11.738	1.00	0.00	H
ATOM	1503	HA	VAL	119	-12.459	70.407	12.923	1.00	0.00	H
ATOM	1504	HB	VAL	119	-9.702	71.694	12.839	1.00	0.00	H
ATOM	1505	HG11	VAL	119	-8.717	69.579	13.713	1.00	0.00	H
ATOM	1506	HG12	VAL	119	-9.642	70.399	14.940	1.00	0.00	H
ATOM	1507	HG13	VAL	119	-10.323	68.994	14.124	1.00	0.00	H
ATOM	1508	HG21	VAL	119	-9.161	69.885	11.286	1.00	0.00	H
ATOM	1509	HG22	VAL	119	-10.727	69.123	11.537	1.00	0.00	H
ATOM	1510	HG23	VAL	119	-10.628	70.668	10.714	1.00	0.00	H
ATOM	1511	N	PHE	120	-11.653	72.876	14.939	1.00	2.39	N
ATOM	1512	CA	PHE	120	-11.895	73.468	16.243	1.00	2.39	C
ATOM	1513	C	PHE	120	-13.374	73.757	16.507	1.00	2.39	C
ATOM	1514	O	PHE	120	-13.781	73.805	17.660	1.00	2.39	O
ATOM	1515	CB	PHE	120	-11.107	74.799	16.289	1.00	2.39	C
ATOM	1516	CG	PHE	120	-10.932	75.451	17.651	1.00	2.39	C
ATOM	1517	CD1	PHE	120	-9.966	74.955	18.552	1.00	2.39	C
ATOM	1518	CD2	PHE	120	-11.698	76.583	18.005	1.00	2.39	C
ATOM	1519	CE1	PHE	120	-9.740	75.607	19.780	1.00	2.39	C
ATOM	1520	CE2	PHE	120	-11.472	77.233	19.233	1.00	2.39	C
ATOM	1521	CZ	PHE	120	-10.490	76.749	20.118	1.00	2.39	C
ATOM	1522	H	PHE	120	-11.160	73.436	14.252	1.00	0.00	H
ATOM	1523	HA	PHE	120	-11.523	72.792	17.016	1.00	0.00	H
ATOM	1524	HB2	PHE	120	-10.103	74.650	15.895	1.00	0.00	H
ATOM	1525	HB3	PHE	120	-11.565	75.523	15.613	1.00	0.00	H
ATOM	1526	HD1	PHE	120	-9.397	74.072	18.308	1.00	0.00	H
ATOM	1527	HD2	PHE	120	-12.454	76.964	17.334	1.00	0.00	H

ATOM	1528	HE1	PHE	120	-9.003	75.227	20.474	1.00	0.00	H
ATOM	1529	HE2	PHE	120	-12.052	78.104	19.503	1.00	0.00	H
ATOM	1530	HZ	PHE	120	-10.329	77.242	21.067	1.00	0.00	H
ATOM	1531	N	GLY	121	-14.189	73.865	15.454	1.00	2.89	N
ATOM	1532	CA	GLY	121	-15.617	74.112	15.559	1.00	2.89	C
ATOM	1533	C	GLY	121	-16.405	72.813	15.704	1.00	2.89	C
ATOM	1534	O	GLY	121	-17.525	72.830	16.207	1.00	2.89	O
ATOM	1535	H	GLY	121	-13.790	73.826	14.528	1.00	0.00	H
ATOM	1536	HA2	GLY	121	-15.844	74.780	16.391	1.00	0.00	H
ATOM	1537	HA3	GLY	121	-15.951	74.617	14.653	1.00	0.00	H
ATOM	1538	N	LEU	122	-15.799	71.684	15.324	1.00	3.13	N
ATOM	1539	CA	LEU	122	-16.353	70.354	15.509	1.00	3.13	C
ATOM	1540	C	LEU	122	-15.914	69.738	16.853	1.00	3.13	C
ATOM	1541	O	LEU	122	-16.693	69.021	17.473	1.00	3.13	O
ATOM	1542	CB	LEU	122	-15.822	69.437	14.379	1.00	3.13	C
ATOM	1543	CG	LEU	122	-16.388	69.756	12.975	1.00	3.13	C
ATOM	1544	CD1	LEU	122	-15.507	69.147	11.873	1.00	3.13	C
ATOM	1545	CD2	LEU	122	-17.844	69.288	12.820	1.00	3.13	C
ATOM	1546	H	LEU	122	-14.892	71.766	14.887	1.00	0.00	H
ATOM	1547	HA	LEU	122	-17.444	70.379	15.492	1.00	0.00	H
ATOM	1548	HB2	LEU	122	-14.734	69.501	14.356	1.00	0.00	H
ATOM	1549	HB3	LEU	122	-16.042	68.393	14.615	1.00	0.00	H
ATOM	1550	HG	LEU	122	-16.377	70.837	12.831	1.00	0.00	H
ATOM	1551	HD11	LEU	122	-15.891	69.389	10.882	1.00	0.00	H
ATOM	1552	HD12	LEU	122	-14.489	69.532	11.926	1.00	0.00	H
ATOM	1553	HD13	LEU	122	-15.458	68.061	11.957	1.00	0.00	H
ATOM	1554	HD21	LEU	122	-18.228	69.521	11.827	1.00	0.00	H
ATOM	1555	HD22	LEU	122	-17.935	68.211	12.966	1.00	0.00	H
ATOM	1556	HD23	LEU	122	-18.502	69.776	13.539	1.00	0.00	H
ATOM	1557	N	TYR	123	-14.687	70.012	17.317	1.00	3.08	N
ATOM	1558	CA	TYR	123	-14.138	69.533	18.595	1.00	3.08	C
ATOM	1559	C	TYR	123	-14.256	70.617	19.693	1.00	3.08	C
ATOM	1560	O	TYR	123	-13.488	70.618	20.655	1.00	3.08	O
ATOM	1561	CB	TYR	123	-12.647	69.153	18.384	1.00	3.08	C
ATOM	1562	CG	TYR	123	-12.398	67.797	17.737	1.00	3.08	C
ATOM	1563	CD1	TYR	123	-12.156	67.681	16.351	1.00	3.08	C
ATOM	1564	CD2	TYR	123	-12.356	66.640	18.543	1.00	3.08	C
ATOM	1565	CE1	TYR	123	-11.847	66.429	15.782	1.00	3.08	C
ATOM	1566	CE2	TYR	123	-12.058	65.388	17.972	1.00	3.08	C
ATOM	1567	CZ	TYR	123	-11.788	65.282	16.597	1.00	3.08	C
ATOM	1568	OH	TYR	123	-11.452	64.070	16.070	1.00	3.08	O
ATOM	1569	H	TYR	123	-14.079	70.576	16.735	1.00	0.00	H
ATOM	1570	HA	TYR	123	-14.681	68.658	18.958	1.00	0.00	H
ATOM	1571	HB2	TYR	123	-12.132	69.931	17.819	1.00	0.00	H
ATOM	1572	HB3	TYR	123	-12.118	69.113	19.338	1.00	0.00	H
ATOM	1573	HD1	TYR	123	-12.188	68.553	15.720	1.00	0.00	H
ATOM	1574	HD2	TYR	123	-12.526	66.719	19.608	1.00	0.00	H
ATOM	1575	HE1	TYR	123	-11.653	66.358	14.722	1.00	0.00	H
ATOM	1576	HE2	TYR	123	-12.001	64.508	18.592	1.00	0.00	H
ATOM	1577	HH	TYR	123	-10.813	64.142	15.359	1.00	0.00	H
ATOM	1578	N	TYR	124	-15.203	71.553	19.553	1.00	3.37	N
ATOM	1579	CA	TYR	124	-15.274	72.793	20.334	1.00	3.37	C
ATOM	1580	C	TYR	124	-15.766	72.609	21.774	1.00	3.37	C
ATOM	1581	O	TYR	124	-15.556	73.479	22.614	1.00	3.37	O
ATOM	1582	CB	TYR	124	-16.278	73.751	19.633	1.00	3.37	C
ATOM	1583	CG	TYR	124	-16.266	75.179	20.163	1.00	3.37	C
ATOM	1584	CD1	TYR	124	-15.174	76.027	19.881	1.00	3.37	C
ATOM	1585	CD2	TYR	124	-17.301	75.635	21.007	1.00	3.37	C
ATOM	1586	CE1	TYR	124	-15.096	77.299	20.477	1.00	3.37	C
ATOM	1587	CE2	TYR	124	-17.220	76.905	21.608	1.00	3.37	C
ATOM	1588	CZ	TYR	124	-16.106	77.729	21.356	1.00	3.37	C
ATOM	1589	OH	TYR	124	-15.973	78.928	21.988	1.00	3.37	O
ATOM	1590	H	TYR	124	-15.826	71.463	18.765	1.00	0.00	H

ATOM	1591	HA	TYR	124	-14.289	73.258	20.338	1.00	0.00	H
ATOM	1592	HB2	TYR	124	-16.078	73.800	18.567	1.00	0.00	H
ATOM	1593	HB3	TYR	124	-17.291	73.351	19.708	1.00	0.00	H
ATOM	1594	HD1	TYR	124	-14.367	75.687	19.250	1.00	0.00	H
ATOM	1595	HD2	TYR	124	-18.134	74.989	21.245	1.00	0.00	H
ATOM	1596	HE1	TYR	124	-14.242	77.931	20.290	1.00	0.00	H
ATOM	1597	HE2	TYR	124	-18.000	77.218	22.286	1.00	0.00	H
ATOM	1598	HH	TYR	124	-16.658	79.107	22.617	1.00	0.00	H
ATOM	1599	N	ASP	125	-16.410	71.490	22.090	1.00	3.53	N
ATOM	1600	CA	ASP	125	-16.804	71.188	23.457	1.00	3.53	C
ATOM	1601	C	ASP	125	-15.644	70.480	24.155	1.00	3.53	C
ATOM	1602	O	ASP	125	-15.285	70.848	25.273	1.00	3.53	O
ATOM	1603	CB	ASP	125	-18.056	70.248	23.421	1.00	3.53	C
ATOM	1604	CG	ASP	125	-17.902	68.833	22.840	1.00	3.53	C
ATOM	1605	OD1	ASP	125	-18.474	67.882	23.407	1.00	3.53	O
ATOM	1606	OD2	ASP	125	-17.121	68.638	21.878	1.00	3.53	O1-
ATOM	1607	H	ASP	125	-16.536	70.761	21.398	1.00	0.00	H
ATOM	1608	HA	ASP	125	-17.064	72.094	24.009	1.00	0.00	H
ATOM	1609	HB2	ASP	125	-18.431	70.133	24.438	1.00	0.00	H
ATOM	1610	HB3	ASP	125	-18.861	70.737	22.872	1.00	0.00	H
ATOM	1611	N	ALA	126	-15.050	69.499	23.466	1.00	3.48	N
ATOM	1612	CA	ALA	126	-13.974	68.631	23.899	1.00	3.48	C
ATOM	1613	C	ALA	126	-12.773	69.427	24.424	1.00	3.48	C
ATOM	1614	O	ALA	126	-12.234	69.096	25.476	1.00	3.48	O
ATOM	1615	CB	ALA	126	-13.563	67.722	22.731	1.00	3.48	C
ATOM	1616	H	ALA	126	-15.570	69.223	22.632	1.00	0.00	H
ATOM	1617	HA	ALA	126	-14.342	68.013	24.720	1.00	0.00	H
ATOM	1618	HB1	ALA	126	-12.618	67.224	22.933	1.00	0.00	H
ATOM	1619	HB2	ALA	126	-14.304	66.946	22.566	1.00	0.00	H
ATOM	1620	HB3	ALA	126	-13.434	68.281	21.806	1.00	0.00	H
ATOM	1621	N	ILE	127	-12.417	70.520	23.733	1.00	3.51	N
ATOM	1622	CA	ILE	127	-11.403	71.479	24.161	1.00	3.51	C
ATOM	1623	C	ILE	127	-11.640	72.052	25.569	1.00	3.51	C
ATOM	1624	O	ILE	127	-10.767	71.959	26.432	1.00	3.51	O
ATOM	1625	CB	ILE	127	-11.226	72.650	23.116	1.00	3.51	C
ATOM	1626	CG1	ILE	127	-12.538	73.361	22.733	1.00	3.51	C
ATOM	1627	CG2	ILE	127	-10.514	72.229	21.815	1.00	3.51	C
ATOM	1628	CD1	ILE	127	-12.389	74.678	21.960	1.00	3.51	C
ATOM	1629	H	ILE	127	-12.932	70.722	22.885	1.00	0.00	H
ATOM	1630	HA	ILE	127	-10.461	70.943	24.234	1.00	0.00	H
ATOM	1631	HB	ILE	127	-10.588	73.403	23.585	1.00	0.00	H
ATOM	1632	HG12	ILE	127	-13.128	72.664	22.155	1.00	0.00	H
ATOM	1633	HG13	ILE	127	-13.133	73.614	23.601	1.00	0.00	H
ATOM	1634	HG21	ILE	127	-10.556	72.997	21.047	1.00	0.00	H
ATOM	1635	HG22	ILE	127	-9.453	72.075	21.978	1.00	0.00	H
ATOM	1636	HG23	ILE	127	-10.959	71.332	21.385	1.00	0.00	H
ATOM	1637	HD11	ILE	127	-13.347	75.195	21.923	1.00	0.00	H
ATOM	1638	HD12	ILE	127	-11.680	75.348	22.446	1.00	0.00	H
ATOM	1639	HD13	ILE	127	-12.086	74.523	20.927	1.00	0.00	H
ATOM	1640	N	TRP	128	-12.820	72.639	25.790	1.00	3.84	N
ATOM	1641	CA	TRP	128	-13.128	73.413	26.982	1.00	3.84	C
ATOM	1642	C	TRP	128	-13.483	72.506	28.152	1.00	3.84	C
ATOM	1643	O	TRP	128	-13.216	72.875	29.287	1.00	3.84	O
ATOM	1644	CB	TRP	128	-14.279	74.401	26.710	1.00	3.84	C
ATOM	1645	CG	TRP	128	-13.995	75.498	25.719	1.00	3.84	C
ATOM	1646	CD1	TRP	128	-14.792	75.825	24.676	1.00	3.84	C
ATOM	1647	CD2	TRP	128	-12.852	76.416	25.642	1.00	3.84	C
ATOM	1648	NE1	TRP	128	-14.232	76.866	23.964	1.00	3.84	N
ATOM	1649	CE2	TRP	128	-13.026	77.259	24.500	1.00	3.84	C
ATOM	1650	CE3	TRP	128	-11.680	76.628	26.412	1.00	3.84	C
ATOM	1651	CZ2	TRP	128	-12.084	78.235	24.129	1.00	3.84	C
ATOM	1652	CZ3	TRP	128	-10.727	77.604	26.051	1.00	3.84	C
ATOM	1653	CH2	TRP	128	-10.926	78.404	24.909	1.00	3.84	C

ATOM	1654	H	TRP	128	-13.508	72.624	25.052	1.00	0.00	H
ATOM	1655	HA	TRP	128	-12.243	73.973	27.281	1.00	0.00	H
ATOM	1656	HB2	TRP	128	-15.173	73.860	26.397	1.00	0.00	H
ATOM	1657	HB3	TRP	128	-14.545	74.898	27.645	1.00	0.00	H
ATOM	1658	HD1	TRP	128	-15.723	75.327	24.441	1.00	0.00	H
ATOM	1659	HE1	TRP	128	-14.662	77.258	23.134	1.00	0.00	H
ATOM	1660	HE3	TRP	128	-11.508	76.040	27.300	1.00	0.00	H
ATOM	1661	HZ2	TRP	128	-12.247	78.852	23.259	1.00	0.00	H
ATOM	1662	HZ3	TRP	128	-9.838	77.742	26.652	1.00	0.00	H
ATOM	1663	HH2	TRP	128	-10.192	79.150	24.638	1.00	0.00	H
ATOM	1664	N	HIS	129	-13.976	71.293	27.876	1.00	3.87	N
ATOM	1665	CA	HIS	129	-14.139	70.274	28.903	1.00	3.87	C
ATOM	1666	C	HIS	129	-12.790	69.699	29.329	1.00	3.87	C
ATOM	1667	O	HIS	129	-12.582	69.446	30.511	1.00	3.87	O
ATOM	1668	CB	HIS	129	-15.007	69.097	28.405	1.00	3.87	C
ATOM	1669	CG	HIS	129	-16.487	69.368	28.276	1.00	3.87	C
ATOM	1670	ND1	HIS	129	-17.026	70.156	27.277	1.00	3.87	N
ATOM	1671	CD2	HIS	129	-17.563	68.949	29.027	1.00	3.87	C
ATOM	1672	CE1	HIS	129	-18.349	70.177	27.444	1.00	3.87	C
ATOM	1673	NE2	HIS	129	-18.747	69.452	28.480	1.00	3.87	N
ATOM	1674	H	HIS	129	-14.186	71.063	26.913	1.00	0.00	H
ATOM	1675	HA	HIS	129	-14.616	70.710	29.786	1.00	0.00	H
ATOM	1676	HB2	HIS	129	-14.632	68.716	27.454	1.00	0.00	H
ATOM	1677	HB3	HIS	129	-14.920	68.269	29.112	1.00	0.00	H
ATOM	1678	HD1	HIS	129	-16.489	70.619	26.544	1.00	0.00	H
ATOM	1679	HD2	HIS	129	-17.577	68.325	29.911	1.00	0.00	H
ATOM	1680	HE1	HIS	129	-19.031	70.715	26.801	1.00	0.00	H
ATOM	1681	N	PHE	130	-11.855	69.506	28.388	1.00	3.78	N
ATOM	1682	CA	PHE	130	-10.542	68.975	28.724	1.00	3.78	C
ATOM	1683	C	PHE	130	-9.697	70.001	29.490	1.00	3.78	C
ATOM	1684	O	PHE	130	-8.960	69.653	30.407	1.00	3.78	O
ATOM	1685	CB	PHE	130	-9.791	68.504	27.466	1.00	3.78	C
ATOM	1686	CG	PHE	130	-8.607	67.594	27.757	1.00	3.78	C
ATOM	1687	CD1	PHE	130	-8.789	66.195	27.775	1.00	3.78	C
ATOM	1688	CD2	PHE	130	-7.323	68.132	27.991	1.00	3.78	C
ATOM	1689	CE1	PHE	130	-7.694	65.341	28.006	1.00	3.78	C
ATOM	1690	CE2	PHE	130	-6.226	67.276	28.210	1.00	3.78	C
ATOM	1691	CZ	PHE	130	-6.411	65.881	28.216	1.00	3.78	C
ATOM	1692	H	PHE	130	-12.065	69.696	27.415	1.00	0.00	H
ATOM	1693	HA	PHE	130	-10.682	68.108	29.373	1.00	0.00	H
ATOM	1694	HB2	PHE	130	-10.470	67.936	26.830	1.00	0.00	H
ATOM	1695	HB3	PHE	130	-9.465	69.356	26.868	1.00	0.00	H
ATOM	1696	HD1	PHE	130	-9.767	65.770	27.602	1.00	0.00	H
ATOM	1697	HD2	PHE	130	-7.173	69.202	27.986	1.00	0.00	H
ATOM	1698	HE1	PHE	130	-7.835	64.270	28.011	1.00	0.00	H
ATOM	1699	HE2	PHE	130	-5.240	67.686	28.368	1.00	0.00	H
ATOM	1700	HZ	PHE	130	-5.570	65.223	28.378	1.00	0.00	H
ATOM	1701	N	ALA	131	-9.825	71.279	29.142	1.00	4.10	N
ATOM	1702	CA	ALA	131	-9.107	72.365	29.785	1.00	4.10	C
ATOM	1703	C	ALA	131	-9.784	72.886	31.054	1.00	4.10	C
ATOM	1704	O	ALA	131	-9.190	73.708	31.747	1.00	4.10	O
ATOM	1705	CB	ALA	131	-9.086	73.519	28.776	1.00	4.10	C
ATOM	1706	H	ALA	131	-10.434	71.506	28.363	1.00	0.00	H
ATOM	1707	HA	ALA	131	-8.086	72.067	30.028	1.00	0.00	H
ATOM	1708	HB1	ALA	131	-8.595	74.381	29.208	1.00	0.00	H
ATOM	1709	HB2	ALA	131	-8.545	73.250	27.869	1.00	0.00	H
ATOM	1710	HB3	ALA	131	-10.093	73.829	28.492	1.00	0.00	H
ATOM	1711	N	ASP	132	-10.989	72.404	31.363	1.00	4.54	N
ATOM	1712	CA	ASP	132	-11.618	72.528	32.671	1.00	4.54	C
ATOM	1713	C	ASP	132	-11.026	71.414	33.533	1.00	4.54	C
ATOM	1714	O	ASP	132	-10.161	71.665	34.374	1.00	4.54	O
ATOM	1715	CB	ASP	132	-13.162	72.442	32.511	1.00	4.54	C
ATOM	1716	CG	ASP	132	-13.938	72.004	33.751	1.00	4.54	C

ATOM	1717	OD1	ASP	132	-14.358	70.828	33.754	1.00	4.54	O
ATOM	1718	OD2	ASP	132	-14.080	72.847	34.660	1.00	4.54	O1-
ATOM	1719	H	ASP	132	-11.435	71.776	30.713	1.00	0.00	H
ATOM	1720	HA	ASP	132	-11.376	73.482	33.143	1.00	0.00	H
ATOM	1721	HB2	ASP	132	-13.552	73.408	32.192	1.00	0.00	H
ATOM	1722	HB3	ASP	132	-13.423	71.742	31.719	1.00	0.00	H
ATOM	1723	N	ASN	133	-11.370	70.172	33.176	1.00	5.19	N
ATOM	1724	CA	ASN	133	-11.030	68.941	33.875	1.00	5.19	C
ATOM	1725	C	ASN	133	-9.514	68.735	34.099	1.00	5.19	C
ATOM	1726	O	ASN	133	-9.114	68.067	35.053	1.00	5.19	O
ATOM	1727	CB	ASN	133	-11.625	67.739	33.099	1.00	5.19	C
ATOM	1728	CG	ASN	133	-11.727	66.448	33.923	1.00	5.19	C
ATOM	1729	OD1	ASN	133	-12.665	66.255	34.686	1.00	5.19	O
ATOM	1730	ND2	ASN	133	-10.801	65.507	33.774	1.00	5.19	N
ATOM	1731	H	ASN	133	-12.158	70.117	32.539	1.00	0.00	H
ATOM	1732	HA	ASN	133	-11.509	69.001	34.855	1.00	0.00	H
ATOM	1733	HB2	ASN	133	-12.645	67.985	32.798	1.00	0.00	H
ATOM	1734	HB3	ASN	133	-11.072	67.560	32.177	1.00	0.00	H
ATOM	1735	HD22	ASN	133	-10.889	64.691	34.353	1.00	0.00	H
ATOM	1736	HD21	ASN	133	-9.971	65.626	33.203	1.00	0.00	H
ATOM	1737	N	GLN	134	-8.662	69.293	33.226	1.00	5.98	N
ATOM	1738	CA	GLN	134	-7.203	69.215	33.295	1.00	5.98	C
ATOM	1739	C	GLN	134	-6.535	70.604	33.329	1.00	5.98	C
ATOM	1740	O	GLN	134	-5.312	70.677	33.241	1.00	5.98	O
ATOM	1741	CB	GLN	134	-6.638	68.342	32.142	1.00	5.98	C
ATOM	1742	CG	GLN	134	-7.399	67.039	31.798	1.00	5.98	C
ATOM	1743	CD	GLN	134	-7.441	65.954	32.872	1.00	5.98	C
ATOM	1744	OE1	GLN	134	-8.160	64.974	32.716	1.00	5.98	O
ATOM	1745	NE2	GLN	134	-6.719	66.094	33.976	1.00	5.98	N
ATOM	1746	H	GLN	134	-9.068	69.765	32.425	1.00	0.00	H
ATOM	1747	HA	GLN	134	-6.913	68.764	34.244	1.00	0.00	H
ATOM	1748	HB2	GLN	134	-6.614	68.945	31.235	1.00	0.00	H
ATOM	1749	HB3	GLN	134	-5.594	68.109	32.351	1.00	0.00	H
ATOM	1750	HG2	GLN	134	-8.423	67.266	31.500	1.00	0.00	H
ATOM	1751	HG3	GLN	134	-6.940	66.591	30.918	1.00	0.00	H
ATOM	1752	HE22	GLN	134	-6.843	65.419	34.712	1.00	0.00	H
ATOM	1753	HE21	GLN	134	-6.209	66.946	34.137	1.00	0.00	H
ATOM	1754	N	LEU	135	-7.299	71.699	33.444	1.00	7.21	N
ATOM	1755	CA	LEU	135	-6.853	73.068	33.761	1.00	7.21	C
ATOM	1756	C	LEU	135	-6.120	73.830	32.630	1.00	7.21	C
ATOM	1757	O	LEU	135	-5.799	75.008	32.790	1.00	7.21	O
ATOM	1758	CB	LEU	135	-6.084	73.138	35.107	1.00	7.21	C
ATOM	1759	CG	LEU	135	-6.840	72.581	36.337	1.00	7.21	C
ATOM	1760	CD1	LEU	135	-5.893	72.448	37.540	1.00	7.21	C
ATOM	1761	CD2	LEU	135	-8.062	73.437	36.713	1.00	7.21	C
ATOM	1762	H	LEU	135	-8.297	71.545	33.575	1.00	0.00	H
ATOM	1763	HA	LEU	135	-7.775	73.633	33.898	1.00	0.00	H
ATOM	1764	HB2	LEU	135	-5.137	72.608	34.996	1.00	0.00	H
ATOM	1765	HB3	LEU	135	-5.805	74.173	35.310	1.00	0.00	H
ATOM	1766	HG	LEU	135	-7.201	71.578	36.107	1.00	0.00	H
ATOM	1767	HD11	LEU	135	-6.410	72.020	38.400	1.00	0.00	H
ATOM	1768	HD12	LEU	135	-5.052	71.795	37.307	1.00	0.00	H
ATOM	1769	HD13	LEU	135	-5.492	73.417	37.840	1.00	0.00	H
ATOM	1770	HD21	LEU	135	-8.564	73.038	37.594	1.00	0.00	H
ATOM	1771	HD22	LEU	135	-7.782	74.468	36.927	1.00	0.00	H
ATOM	1772	HD23	LEU	135	-8.805	73.452	35.915	1.00	0.00	H
ATOM	1773	N	PHE	136	-5.846	73.197	31.481	1.00	6.65	N
ATOM	1774	CA	PHE	136	-5.018	73.710	30.377	1.00	6.65	C
ATOM	1775	C	PHE	136	-5.712	74.792	29.503	1.00	6.65	C
ATOM	1776	O	PHE	136	-5.608	74.761	28.271	1.00	6.65	O
ATOM	1777	CB	PHE	136	-4.613	72.519	29.475	1.00	6.65	C
ATOM	1778	CG	PHE	136	-3.819	71.400	30.126	1.00	6.65	C
ATOM	1779	CD1	PHE	136	-2.638	71.681	30.847	1.00	6.65	C

ATOM	1780	CD2	PHE	136	-4.246	70.063	29.985	1.00	6.65	C
ATOM	1781	CE1	PHE	136	-1.899	70.633	31.430	1.00	6.65	C
ATOM	1782	CE2	PHE	136	-3.502	69.016	30.561	1.00	6.65	C
ATOM	1783	CZ	PHE	136	-2.330	69.300	31.287	1.00	6.65	C
ATOM	1784	H	PHE	136	-6.066	72.211	31.488	1.00	0.00	H
ATOM	1785	HA	PHE	136	-4.122	74.169	30.797	1.00	0.00	H
ATOM	1786	HB2	PHE	136	-5.506	72.099	29.009	1.00	0.00	H
ATOM	1787	HB3	PHE	136	-3.987	72.881	28.658	1.00	0.00	H
ATOM	1788	HD1	PHE	136	-2.296	72.698	30.967	1.00	0.00	H
ATOM	1789	HD2	PHE	136	-5.151	69.834	29.441	1.00	0.00	H
ATOM	1790	HE1	PHE	136	-1.005	70.853	31.994	1.00	0.00	H
ATOM	1791	HE2	PHE	136	-3.840	67.995	30.460	1.00	0.00	H
ATOM	1792	HZ	PHE	136	-1.767	68.499	31.741	1.00	0.00	H
ATOM	1793	N	VAL	137	-6.419	75.743	30.131	1.00	5.94	N
ATOM	1794	CA	VAL	137	-7.201	76.846	29.548	1.00	5.94	C
ATOM	1795	C	VAL	137	-6.427	77.717	28.533	1.00	5.94	C
ATOM	1796	O	VAL	137	-6.998	78.181	27.549	1.00	5.94	O
ATOM	1797	CB	VAL	137	-7.861	77.650	30.703	1.00	5.94	C
ATOM	1798	CG1	VAL	137	-6.851	78.335	31.646	1.00	5.94	C
ATOM	1799	CG2	VAL	137	-8.914	78.658	30.208	1.00	5.94	C
ATOM	1800	H	VAL	137	-6.426	75.668	31.147	1.00	0.00	H
ATOM	1801	HA	VAL	137	-8.004	76.399	28.965	1.00	0.00	H
ATOM	1802	HB	VAL	137	-8.409	76.924	31.308	1.00	0.00	H
ATOM	1803	HG11	VAL	137	-7.363	78.799	32.489	1.00	0.00	H
ATOM	1804	HG12	VAL	137	-6.137	77.624	32.063	1.00	0.00	H
ATOM	1805	HG13	VAL	137	-6.285	79.115	31.137	1.00	0.00	H
ATOM	1806	HG21	VAL	137	-9.449	79.108	31.045	1.00	0.00	H
ATOM	1807	HG22	VAL	137	-8.465	79.467	29.631	1.00	0.00	H
ATOM	1808	HG23	VAL	137	-9.656	78.171	29.574	1.00	0.00	H
ATOM	1809	N	SER	138	-5.125	77.899	28.754	1.00	4.61	N
ATOM	1810	CA	SER	138	-4.163	78.461	27.823	1.00	4.61	C
ATOM	1811	C	SER	138	-2.810	77.838	28.189	1.00	4.61	C
ATOM	1812	O	SER	138	-2.542	77.614	29.373	1.00	4.61	O
ATOM	1813	CB	SER	138	-4.235	80.003	27.820	1.00	4.61	C
ATOM	1814	OG	SER	138	-3.325	80.586	26.903	1.00	4.61	O
ATOM	1815	H	SER	138	-4.716	77.472	29.575	1.00	0.00	H
ATOM	1816	HA	SER	138	-4.430	78.113	26.826	1.00	0.00	H
ATOM	1817	HB2	SER	138	-5.242	80.324	27.548	1.00	0.00	H
ATOM	1818	HB3	SER	138	-4.035	80.395	28.818	1.00	0.00	H
ATOM	1819	HG	SER	138	-3.573	81.491	26.772	1.00	0.00	H
ATOM	1820	N	GLY	139	-1.989	77.488	27.196	1.00	3.69	N
ATOM	1821	CA	GLY	139	-0.668	76.898	27.384	1.00	3.69	C
ATOM	1822	C	GLY	139	0.380	77.541	26.468	1.00	3.69	C
ATOM	1823	O	GLY	139	0.015	77.994	25.383	1.00	3.69	O
ATOM	1824	H	GLY	139	-2.233	77.770	26.254	1.00	0.00	H
ATOM	1825	HA2	GLY	139	-0.362	76.976	28.427	1.00	0.00	H
ATOM	1826	HA3	GLY	139	-0.727	75.835	27.148	1.00	0.00	H
ATOM	1827	N	PRO	140	1.684	77.508	26.835	1.00	2.84	N
ATOM	1828	CA	PRO	140	2.796	77.849	25.928	1.00	2.84	C
ATOM	1829	C	PRO	140	3.081	76.725	24.912	1.00	2.84	C
ATOM	1830	O	PRO	140	3.742	76.941	23.902	1.00	2.84	O
ATOM	1831	CB	PRO	140	3.997	78.054	26.861	1.00	2.84	C
ATOM	1832	CG	PRO	140	3.721	77.141	28.048	1.00	2.84	C
ATOM	1833	CD	PRO	140	2.193	77.112	28.154	1.00	2.84	C
ATOM	1834	HA	PRO	140	2.598	78.763	25.370	1.00	0.00	H
ATOM	1835	HB2	PRO	140	4.953	77.838	26.382	1.00	0.00	H
ATOM	1836	HB3	PRO	140	4.027	79.094	27.188	1.00	0.00	H
ATOM	1837	HG2	PRO	140	4.096	76.140	27.828	1.00	0.00	H
ATOM	1838	HG3	PRO	140	4.205	77.479	28.964	1.00	0.00	H
ATOM	1839	HD3	PRO	140	1.854	77.836	28.898	1.00	0.00	H
ATOM	1840	HD2	PRO	140	1.843	76.124	28.455	1.00	0.00	H
ATOM	1841	N	ASN	141	2.571	75.522	25.183	1.00	2.48	N
ATOM	1842	CA	ASN	141	2.506	74.373	24.297	1.00	2.48	C

ATOM	1843	C	ASN	141	1.171	73.668	24.572	1.00	2.48	C
ATOM	1844	O	ASN	141	0.137	74.331	24.494	1.00	2.48	O
ATOM	1845	CB	ASN	141	3.820	73.552	24.231	1.00	2.48	C
ATOM	1846	CG	ASN	141	4.313	73.014	25.574	1.00	2.48	C
ATOM	1847	OD1	ASN	141	3.738	72.067	26.099	1.00	2.48	O
ATOM	1848	ND2	ASN	141	5.355	73.594	26.159	1.00	2.48	N
ATOM	1849	H	ASN	141	2.016	75.448	26.018	1.00	0.00	H
ATOM	1850	HA	ASN	141	2.364	74.767	23.291	1.00	0.00	H
ATOM	1851	HB2	ASN	141	3.696	72.712	23.547	1.00	0.00	H
ATOM	1852	HB3	ASN	141	4.604	74.169	23.790	1.00	0.00	H
ATOM	1853	HD22	ASN	141	5.689	73.211	27.027	1.00	0.00	H
ATOM	1854	HD21	ASN	141	5.844	74.392	25.744	1.00	0.00	H
ATOM	1855	N	GLY	142	1.164	72.390	24.972	1.00	2.16	N
ATOM	1856	CA	GLY	142	0.018	71.486	25.150	1.00	2.16	C
ATOM	1857	C	GLY	142	-1.199	71.760	24.252	1.00	2.16	C
ATOM	1858	O	GLY	142	-1.188	71.373	23.085	1.00	2.16	O
ATOM	1859	H	GLY	142	2.078	71.990	25.176	1.00	0.00	H
ATOM	1860	HA2	GLY	142	0.357	70.467	24.959	1.00	0.00	H
ATOM	1861	HA3	GLY	142	-0.288	71.513	26.196	1.00	0.00	H
ATOM	1862	N	THR	143	-2.222	72.456	24.765	1.00	2.00	N
ATOM	1863	CA	THR	143	-3.439	72.837	24.046	1.00	2.00	C
ATOM	1864	C	THR	143	-3.216	73.764	22.822	1.00	2.00	C
ATOM	1865	O	THR	143	-4.060	73.787	21.927	1.00	2.00	O
ATOM	1866	CB	THR	143	-4.458	73.396	25.072	1.00	2.00	C
ATOM	1867	OG1	THR	143	-3.873	74.366	25.938	1.00	2.00	O
ATOM	1868	CG2	THR	143	-5.072	72.283	25.935	1.00	2.00	C
ATOM	1869	H	THR	143	-2.144	72.855	25.689	1.00	0.00	H
ATOM	1870	HA	THR	143	-3.866	71.922	23.635	1.00	0.00	H
ATOM	1871	HB	THR	143	-5.273	73.872	24.525	1.00	0.00	H
ATOM	1872	HG1	THR	143	-4.496	74.548	26.647	1.00	0.00	H
ATOM	1873	HG21	THR	143	-5.836	72.675	26.607	1.00	0.00	H
ATOM	1874	HG22	THR	143	-5.557	71.530	25.313	1.00	0.00	H
ATOM	1875	HG23	THR	143	-4.320	71.772	26.537	1.00	0.00	H
ATOM	1876	N	ALA	144	-2.063	74.442	22.706	1.00	1.84	N
ATOM	1877	CA	ALA	144	-1.661	75.187	21.508	1.00	1.84	C
ATOM	1878	C	ALA	144	-1.021	74.267	20.446	1.00	1.84	C
ATOM	1879	O	ALA	144	-0.897	74.646	19.281	1.00	1.84	O
ATOM	1880	CB	ALA	144	-0.590	76.217	21.902	1.00	1.84	C
ATOM	1881	H	ALA	144	-1.392	74.390	23.470	1.00	0.00	H
ATOM	1882	HA	ALA	144	-2.520	75.701	21.072	1.00	0.00	H
ATOM	1883	HB1	ALA	144	-0.280	76.798	21.034	1.00	0.00	H
ATOM	1884	HB2	ALA	144	-0.959	76.917	22.650	1.00	0.00	H
ATOM	1885	HB3	ALA	144	0.308	75.748	22.303	1.00	0.00	H
ATOM	1886	N	GLY	145	-0.637	73.040	20.819	1.00	1.79	N
ATOM	1887	CA	GLY	145	0.147	72.116	20.001	1.00	1.79	C
ATOM	1888	C	GLY	145	-0.721	71.030	19.367	1.00	1.79	C
ATOM	1889	O	GLY	145	-0.210	70.086	18.775	1.00	1.79	O
ATOM	1890	H	GLY	145	-0.852	72.733	21.763	1.00	0.00	H
ATOM	1891	HA2	GLY	145	0.696	72.637	19.216	1.00	0.00	H
ATOM	1892	HA3	GLY	145	0.891	71.634	20.636	1.00	0.00	H
ATOM	1893	N	ILE	146	-2.049	71.190	19.443	1.00	1.55	N
ATOM	1894	CA	ILE	146	-3.048	70.317	18.823	1.00	1.55	C
ATOM	1895	C	ILE	146	-3.217	70.643	17.317	1.00	1.55	C
ATOM	1896	O	ILE	146	-3.930	69.950	16.594	1.00	1.55	O
ATOM	1897	CB	ILE	146	-4.380	70.457	19.621	1.00	1.55	C
ATOM	1898	CG1	ILE	146	-5.182	71.751	19.364	1.00	1.55	C
ATOM	1899	CG2	ILE	146	-4.110	70.259	21.125	1.00	1.55	C
ATOM	1900	CD1	ILE	146	-6.468	71.891	20.196	1.00	1.55	C
ATOM	1901	H	ILE	146	-2.387	71.963	19.997	1.00	0.00	H
ATOM	1902	HA	ILE	146	-2.697	69.288	18.905	1.00	0.00	H
ATOM	1903	HB	ILE	146	-5.018	69.636	19.290	1.00	0.00	H
ATOM	1904	HG12	ILE	146	-4.555	72.629	19.520	1.00	0.00	H
ATOM	1905	HG13	ILE	146	-5.485	71.755	18.320	1.00	0.00	H

ATOM	1906	HG21	ILE	146	-5.023	70.101	21.697	1.00	0.00	H
ATOM	1907	HG22	ILE	146	-3.464	69.399	21.279	1.00	0.00	H
ATOM	1908	HG23	ILE	146	-3.601	71.118	21.555	1.00	0.00	H
ATOM	1909	HD11	ILE	146	-6.251	72.019	21.257	1.00	0.00	H
ATOM	1910	HD12	ILE	146	-7.038	72.766	19.884	1.00	0.00	H
ATOM	1911	HD13	ILE	146	-7.112	71.018	20.083	1.00	0.00	H
ATOM	1912	N	PHE	147	-2.538	71.701	16.858	1.00	1.14	N
ATOM	1913	CA	PHE	147	-2.460	72.174	15.484	1.00	1.14	C
ATOM	1914	C	PHE	147	-0.990	72.382	15.089	1.00	1.14	C
ATOM	1915	O	PHE	147	-0.545	71.931	14.031	1.00	1.14	O
ATOM	1916	CB	PHE	147	-3.239	73.502	15.348	1.00	1.14	C
ATOM	1917	CG	PHE	147	-4.752	73.397	15.431	1.00	1.14	C
ATOM	1918	CD1	PHE	147	-5.498	72.959	14.320	1.00	1.14	C
ATOM	1919	CD2	PHE	147	-5.422	73.727	16.624	1.00	1.14	C
ATOM	1920	CE1	PHE	147	-6.893	72.793	14.421	1.00	1.14	C
ATOM	1921	CE2	PHE	147	-6.819	73.593	16.717	1.00	1.14	C
ATOM	1922	CZ	PHE	147	-7.555	73.108	15.621	1.00	1.14	C
ATOM	1923	H	PHE	147	-1.945	72.138	17.545	1.00	0.00	H
ATOM	1924	HA	PHE	147	-2.882	71.435	14.800	1.00	0.00	H
ATOM	1925	HB2	PHE	147	-2.894	74.216	16.096	1.00	0.00	H
ATOM	1926	HB3	PHE	147	-2.990	73.956	14.395	1.00	0.00	H
ATOM	1927	HD1	PHE	147	-4.992	72.726	13.399	1.00	0.00	H
ATOM	1928	HD2	PHE	147	-4.868	74.049	17.493	1.00	0.00	H
ATOM	1929	HE1	PHE	147	-7.462	72.408	13.590	1.00	0.00	H
ATOM	1930	HE2	PHE	147	-7.313	73.831	17.647	1.00	0.00	H
ATOM	1931	HZ	PHE	147	-8.622	72.965	15.702	1.00	0.00	H
ATOM	1932	N	ALA	148	-0.251	73.122	15.923	1.00	0.86	N
ATOM	1933	CA	ALA	148	1.072	73.653	15.630	1.00	0.86	C
ATOM	1934	C	ALA	148	2.185	72.697	16.088	1.00	0.86	C
ATOM	1935	O	ALA	148	2.034	71.989	17.082	1.00	0.86	O
ATOM	1936	CB	ALA	148	1.202	74.936	16.461	1.00	0.86	C
ATOM	1937	H	ALA	148	-0.648	73.411	16.804	1.00	0.00	H
ATOM	1938	HA	ALA	148	1.172	73.881	14.566	1.00	0.00	H
ATOM	1939	HB1	ALA	148	2.105	75.472	16.187	1.00	0.00	H
ATOM	1940	HB2	ALA	148	0.363	75.609	16.288	1.00	0.00	H
ATOM	1941	HB3	ALA	148	1.249	74.735	17.533	1.00	0.00	H
ATOM	1942	N	THR	149	3.323	72.731	15.390	1.00	1.00	N
ATOM	1943	CA	THR	149	4.582	72.074	15.757	1.00	1.00	C
ATOM	1944	C	THR	149	5.175	72.632	17.068	1.00	1.00	C
ATOM	1945	O	THR	149	4.955	73.799	17.404	1.00	1.00	O
ATOM	1946	CB	THR	149	5.594	72.312	14.612	1.00	1.00	C
ATOM	1947	OG1	THR	149	5.669	73.688	14.296	1.00	1.00	O
ATOM	1948	CG2	THR	149	5.310	71.514	13.340	1.00	1.00	C
ATOM	1949	H	THR	149	3.345	73.329	14.557	1.00	0.00	H
ATOM	1950	HA	THR	149	4.405	71.004	15.883	1.00	0.00	H
ATOM	1951	HB	THR	149	6.591	72.042	14.955	1.00	0.00	H
ATOM	1952	HG1	THR	149	4.880	73.903	13.761	1.00	0.00	H
ATOM	1953	HG21	THR	149	6.110	71.662	12.616	1.00	0.00	H
ATOM	1954	HG22	THR	149	5.273	70.451	13.560	1.00	0.00	H
ATOM	1955	HG23	THR	149	4.364	71.788	12.875	1.00	0.00	H
ATOM	1956	N	TYR	150	5.983	71.831	17.768	1.00	1.45	N
ATOM	1957	CA	TYR	150	6.765	72.214	18.942	1.00	1.45	C
ATOM	1958	C	TYR	150	8.027	71.341	19.013	1.00	1.45	C
ATOM	1959	O	TYR	150	7.982	70.217	18.508	1.00	1.45	O
ATOM	1960	CB	TYR	150	5.908	72.154	20.228	1.00	1.45	C
ATOM	1961	CG	TYR	150	5.067	73.392	20.491	1.00	1.45	C
ATOM	1962	CD1	TYR	150	5.676	74.551	21.014	1.00	1.45	C
ATOM	1963	CD2	TYR	150	3.687	73.401	20.198	1.00	1.45	C
ATOM	1964	CE1	TYR	150	4.910	75.701	21.271	1.00	1.45	C
ATOM	1965	CE2	TYR	150	2.924	74.560	20.436	1.00	1.45	C
ATOM	1966	CZ	TYR	150	3.533	75.698	20.994	1.00	1.45	C
ATOM	1967	OH	TYR	150	2.783	76.789	21.295	1.00	1.45	O
ATOM	1968	H	TYR	150	6.224	70.918	17.395	1.00	0.00	H

ATOM	1969	HA	TYR	150	7.102	73.239	18.783	1.00	0.00	H
ATOM	1970	HB2	TYR	150	5.269	71.270	20.208	1.00	0.00	H
ATOM	1971	HB3	TYR	150	6.551	72.013	21.099	1.00	0.00	H
ATOM	1972	HD1	TYR	150	6.732	74.565	21.223	1.00	0.00	H
ATOM	1973	HD2	TYR	150	3.216	72.529	19.764	1.00	0.00	H
ATOM	1974	HE1	TYR	150	5.377	76.579	21.694	1.00	0.00	H
ATOM	1975	HE2	TYR	150	1.869	74.572	20.205	1.00	0.00	H
ATOM	1976	HH	TYR	150	3.082	77.224	22.098	1.00	0.00	H
ATOM	1977	N	PRO	151	9.130	71.814	19.637	1.00	2.00	N
ATOM	1978	CA	PRO	151	10.358	71.029	19.776	1.00	2.00	C
ATOM	1979	C	PRO	151	10.236	69.989	20.902	1.00	2.00	C
ATOM	1980	O	PRO	151	9.519	70.198	21.884	1.00	2.00	O
ATOM	1981	CB	PRO	151	11.438	72.060	20.144	1.00	2.00	C
ATOM	1982	CG	PRO	151	10.687	73.174	20.856	1.00	2.00	C
ATOM	1983	CD	PRO	151	9.285	73.141	20.242	1.00	2.00	C
ATOM	1984	HA	PRO	151	10.611	70.545	18.833	1.00	0.00	H
ATOM	1985	HB2	PRO	151	12.246	71.652	20.755	1.00	0.00	H
ATOM	1986	HB3	PRO	151	11.902	72.441	19.238	1.00	0.00	H
ATOM	1987	HG2	PRO	151	10.628	72.942	21.921	1.00	0.00	H
ATOM	1988	HG3	PRO	151	11.176	74.144	20.755	1.00	0.00	H
ATOM	1989	HD3	PRO	151	9.184	73.912	19.480	1.00	0.00	H
ATOM	1990	HD2	PRO	151	8.537	73.314	21.013	1.00	0.00	H
ATOM	1991	N	SER	152	11.022	68.915	20.800	1.00	2.65	N
ATOM	1992	CA	SER	152	11.374	68.050	21.921	1.00	2.65	C
ATOM	1993	C	SER	152	12.347	68.812	22.847	1.00	2.65	C
ATOM	1994	O	SER	152	13.123	69.657	22.397	1.00	2.65	O
ATOM	1995	CB	SER	152	12.038	66.773	21.369	1.00	2.65	C
ATOM	1996	OG	SER	152	12.282	65.813	22.384	1.00	2.65	O
ATOM	1997	H	SER	152	11.509	68.784	19.924	1.00	0.00	H
ATOM	1998	HA	SER	152	10.466	67.789	22.468	1.00	0.00	H
ATOM	1999	HB2	SER	152	11.392	66.322	20.620	1.00	0.00	H
ATOM	2000	HB3	SER	152	12.978	67.008	20.869	1.00	0.00	H
ATOM	2001	HG	SER	152	12.601	65.024	21.966	1.00	0.00	H
ATOM	2002	N	GLY	153	12.367	68.487	24.143	1.00	3.35	N
ATOM	2003	CA	GLY	153	12.967	69.325	25.186	1.00	3.35	C
ATOM	2004	C	GLY	153	14.457	69.053	25.412	1.00	3.35	C
ATOM	2005	O	GLY	153	14.967	69.274	26.507	1.00	3.35	O
ATOM	2006	H	GLY	153	11.824	67.685	24.425	1.00	0.00	H
ATOM	2007	HA2	GLY	153	12.837	70.383	24.950	1.00	0.00	H
ATOM	2008	HA3	GLY	153	12.435	69.149	26.121	1.00	0.00	H
ATOM	2009	N	HIE	154	15.140	68.591	24.361	1.00	3.77	N
ATOM	2010	CA	HIE	154	16.587	68.434	24.230	1.00	3.77	C
ATOM	2011	C	HIE	154	17.101	69.130	22.955	1.00	3.77	C
ATOM	2012	O	HIE	154	18.290	69.039	22.654	1.00	3.77	O
ATOM	2013	CB	HIE	154	16.972	66.934	24.268	1.00	3.77	C
ATOM	2014	CG	HIE	154	16.989	66.270	25.620	1.00	3.77	C
ATOM	2015	ND1	HIE	154	17.069	64.879	25.758	1.00	3.77	N
ATOM	2016	CD2	HIE	154	16.972	66.827	26.882	1.00	3.77	C
ATOM	2017	CE1	HIE	154	17.077	64.654	27.064	1.00	3.77	C
ATOM	2018	NE2	HIE	154	17.019	65.775	27.782	1.00	3.77	N
ATOM	2019	H	HIE	154	14.597	68.481	23.515	1.00	0.00	H
ATOM	2020	HA	HIE	154	17.092	68.953	25.046	1.00	0.00	H
ATOM	2021	HB2	HIE	154	16.310	66.367	23.613	1.00	0.00	H
ATOM	2022	HB3	HIE	154	17.975	66.794	23.862	1.00	0.00	H
ATOM	2023	HD2	HIE	154	16.924	67.861	27.194	1.00	0.00	H
ATOM	2024	HE1	HIE	154	17.128	63.665	27.496	1.00	0.00	H
ATOM	2025	HE2	HIE	154	17.011	65.844	28.788	1.00	0.00	H
ATOM	2026	N	LEU	155	16.222	69.801	22.197	1.00	3.73	N
ATOM	2027	CA	LEU	155	16.530	70.496	20.952	1.00	3.73	C
ATOM	2028	C	LEU	155	16.385	72.004	21.195	1.00	3.73	C
ATOM	2029	O	LEU	155	15.401	72.437	21.798	1.00	3.73	O
ATOM	2030	CB	LEU	155	15.493	70.077	19.878	1.00	3.73	C
ATOM	2031	CG	LEU	155	15.378	68.559	19.609	1.00	3.73	C

ATOM	2032	CD1	LEU	155	14.261	68.279	18.594	1.00	3.73	C
ATOM	2033	CD2	LEU	155	16.694	67.924	19.134	1.00	3.73	C
ATOM	2034	H	LEU	155	15.281	69.948	22.540	1.00	0.00	H
ATOM	2035	HA	LEU	155	17.542	70.277	20.607	1.00	0.00	H
ATOM	2036	HB2	LEU	155	14.508	70.443	20.177	1.00	0.00	H
ATOM	2037	HB3	LEU	155	15.709	70.594	18.944	1.00	0.00	H
ATOM	2038	HG	LEU	155	15.096	68.071	20.542	1.00	0.00	H
ATOM	2039	HD11	LEU	155	14.127	67.207	18.448	1.00	0.00	H
ATOM	2040	HD12	LEU	155	13.310	68.691	18.929	1.00	0.00	H
ATOM	2041	HD13	LEU	155	14.484	68.720	17.621	1.00	0.00	H
ATOM	2042	HD21	LEU	155	16.578	66.848	18.999	1.00	0.00	H
ATOM	2043	HD22	LEU	155	17.013	68.337	18.179	1.00	0.00	H
ATOM	2044	HD23	LEU	155	17.499	68.069	19.856	1.00	0.00	H
ATOM	2045	N	ASP	156	17.331	72.801	20.685	1.00	3.58	N
ATOM	2046	CA	ASP	156	17.175	74.247	20.510	1.00	3.58	C
ATOM	2047	C	ASP	156	16.463	74.507	19.155	1.00	3.58	C
ATOM	2048	O	ASP	156	16.293	73.600	18.333	1.00	3.58	O
ATOM	2049	CB	ASP	156	18.585	74.919	20.552	1.00	3.58	C
ATOM	2050	CG	ASP	156	18.596	76.403	20.179	1.00	3.58	C
ATOM	2051	OD1	ASP	156	17.849	77.151	20.850	1.00	3.58	O
ATOM	2052	OD2	ASP	156	19.075	76.715	19.068	1.00	3.58	O1-
ATOM	2053	H	ASP	156	18.124	72.379	20.212	1.00	0.00	H
ATOM	2054	HA	ASP	156	16.561	74.667	21.308	1.00	0.00	H
ATOM	2055	HB2	ASP	156	19.011	74.823	21.550	1.00	0.00	H
ATOM	2056	HB3	ASP	156	19.268	74.407	19.876	1.00	0.00	H
ATOM	2057	N	MET	157	16.110	75.773	18.914	1.00	3.50	N
ATOM	2058	CA	MET	157	15.887	76.436	17.631	1.00	3.50	C
ATOM	2059	C	MET	157	16.699	75.879	16.463	1.00	3.50	C
ATOM	2060	O	MET	157	16.115	75.400	15.490	1.00	3.50	O
ATOM	2061	CB	MET	157	16.037	77.968	17.853	1.00	3.50	C
ATOM	2062	CG	MET	157	16.436	78.869	16.664	1.00	3.50	C
ATOM	2063	SD	MET	157	15.309	78.954	15.249	1.00	3.50	S
ATOM	2064	CE	MET	157	13.803	79.518	16.073	1.00	3.50	C
ATOM	2065	H	MET	157	16.447	76.397	19.646	1.00	0.00	H
ATOM	2066	HA	MET	157	14.844	76.252	17.370	1.00	0.00	H
ATOM	2067	HB2	MET	157	15.139	78.355	18.333	1.00	0.00	H
ATOM	2068	HB3	MET	157	16.832	78.119	18.580	1.00	0.00	H
ATOM	2069	HG2	MET	157	16.555	79.887	17.036	1.00	0.00	H
ATOM	2070	HG3	MET	157	17.422	78.589	16.293	1.00	0.00	H
ATOM	2071	HE1	MET	157	13.006	79.678	15.350	1.00	0.00	H
ATOM	2072	HE2	MET	157	13.452	78.771	16.784	1.00	0.00	H
ATOM	2073	HE3	MET	157	13.984	80.452	16.605	1.00	0.00	H
ATOM	2074	N	ILE	158	18.033	75.912	16.571	1.00	3.46	N
ATOM	2075	CA	ILE	158	18.918	75.523	15.477	1.00	3.46	C
ATOM	2076	C	ILE	158	18.811	74.029	15.164	1.00	3.46	C
ATOM	2077	O	ILE	158	18.850	73.631	14.001	1.00	3.46	O
ATOM	2078	CB	ILE	158	20.376	75.965	15.812	1.00	3.46	C
ATOM	2079	CG1	ILE	158	21.292	76.021	14.568	1.00	3.46	C
ATOM	2080	CG2	ILE	158	21.058	75.122	16.912	1.00	3.46	C
ATOM	2081	CD1	ILE	158	20.915	77.103	13.544	1.00	3.46	C
ATOM	2082	H	ILE	158	18.450	76.267	17.443	1.00	0.00	H
ATOM	2083	HA	ILE	158	18.573	76.064	14.596	1.00	0.00	H
ATOM	2084	HB	ILE	158	20.317	76.982	16.204	1.00	0.00	H
ATOM	2085	HG12	ILE	158	22.316	76.212	14.890	1.00	0.00	H
ATOM	2086	HG13	ILE	158	21.310	75.050	14.076	1.00	0.00	H
ATOM	2087	HG21	ILE	158	21.977	75.597	17.253	1.00	0.00	H
ATOM	2088	HG22	ILE	158	20.419	75.003	17.785	1.00	0.00	H
ATOM	2089	HG23	ILE	158	21.317	74.123	16.558	1.00	0.00	H
ATOM	2090	HD11	ILE	158	21.665	77.160	12.755	1.00	0.00	H
ATOM	2091	HD12	ILE	158	19.961	76.891	13.060	1.00	0.00	H
ATOM	2092	HD13	ILE	158	20.847	78.086	14.010	1.00	0.00	H
ATOM	2093	N	ASN	159	18.580	73.217	16.200	1.00	3.34	N
ATOM	2094	CA	ASN	159	18.405	71.783	16.057	1.00	3.34	C

ATOM	2095	C	ASN	159	17.074	71.441	15.391	1.00	3.34	C
ATOM	2096	O	ASN	159	17.004	70.507	14.593	1.00	3.34	O
ATOM	2097	CB	ASN	159	18.415	71.048	17.416	1.00	3.34	C
ATOM	2098	CG	ASN	159	19.690	71.201	18.230	1.00	3.34	C
ATOM	2099	OD1	ASN	159	19.712	71.940	19.205	1.00	3.34	O
ATOM	2100	ND2	ASN	159	20.746	70.467	17.899	1.00	3.34	N
ATOM	2101	H	ASN	159	18.465	73.643	17.111	1.00	0.00	H
ATOM	2102	HA	ASN	159	19.210	71.391	15.434	1.00	0.00	H
ATOM	2103	HB2	ASN	159	17.585	71.392	18.030	1.00	0.00	H
ATOM	2104	HB3	ASN	159	18.245	69.983	17.255	1.00	0.00	H
ATOM	2105	HD22	ASN	159	21.570	70.556	18.472	1.00	0.00	H
ATOM	2106	HD21	ASN	159	20.713	69.830	17.120	1.00	0.00	H
ATOM	2107	N	GLY	160	16.043	72.244	15.665	1.00	3.33	N
ATOM	2108	CA	GLY	160	14.726	72.088	15.075	1.00	3.33	C
ATOM	2109	C	GLY	160	14.696	72.579	13.629	1.00	3.33	C
ATOM	2110	O	GLY	160	13.986	72.027	12.790	1.00	3.33	O
ATOM	2111	H	GLY	160	16.181	72.989	16.342	1.00	0.00	H
ATOM	2112	HA2	GLY	160	14.396	71.049	15.124	1.00	0.00	H
ATOM	2113	HA3	GLY	160	14.021	72.682	15.646	1.00	0.00	H
ATOM	2114	N	PHE	161	15.534	73.571	13.319	1.00	3.33	N
ATOM	2115	CA	PHE	161	15.719	74.102	11.980	1.00	3.33	C
ATOM	2116	C	PHE	161	16.506	73.149	11.074	1.00	3.33	C
ATOM	2117	O	PHE	161	16.368	73.212	9.853	1.00	3.33	O
ATOM	2118	CB	PHE	161	16.509	75.442	12.077	1.00	3.33	C
ATOM	2119	CG	PHE	161	16.279	76.450	10.952	1.00	3.33	C
ATOM	2120	CD1	PHE	161	16.846	76.257	9.672	1.00	3.33	C
ATOM	2121	CD2	PHE	161	15.509	77.611	11.189	1.00	3.33	C
ATOM	2122	CE1	PHE	161	16.596	77.174	8.633	1.00	3.33	C
ATOM	2123	CE2	PHE	161	15.264	78.531	10.154	1.00	3.33	C
ATOM	2124	CZ	PHE	161	15.797	78.308	8.870	1.00	3.33	C
ATOM	2125	H	PHE	161	16.041	74.012	14.080	1.00	0.00	H
ATOM	2126	HA	PHE	161	14.740	74.287	11.539	1.00	0.00	H
ATOM	2127	HB2	PHE	161	16.262	75.950	13.008	1.00	0.00	H
ATOM	2128	HB3	PHE	161	17.581	75.251	12.153	1.00	0.00	H
ATOM	2129	HD1	PHE	161	17.477	75.403	9.474	1.00	0.00	H
ATOM	2130	HD2	PHE	161	15.112	77.818	12.171	1.00	0.00	H
ATOM	2131	HE1	PHE	161	17.025	77.008	7.655	1.00	0.00	H
ATOM	2132	HE2	PHE	161	14.674	79.414	10.354	1.00	0.00	H
ATOM	2133	HZ	PHE	161	15.610	79.014	8.074	1.00	0.00	H
ATOM	2134	N	PHE	162	17.326	72.260	11.637	1.00	3.37	N
ATOM	2135	CA	PHE	162	18.013	71.250	10.845	1.00	3.37	C
ATOM	2136	C	PHE	162	17.068	70.158	10.413	1.00	3.37	C
ATOM	2137	O	PHE	162	17.070	69.774	9.247	1.00	3.37	O
ATOM	2138	CB	PHE	162	19.146	70.594	11.673	1.00	3.37	C
ATOM	2139	CG	PHE	162	20.340	71.453	12.074	1.00	3.37	C
ATOM	2140	CD1	PHE	162	20.762	72.558	11.300	1.00	3.37	C
ATOM	2141	CD2	PHE	162	21.065	71.110	13.236	1.00	3.37	C
ATOM	2142	CE1	PHE	162	21.887	73.309	11.690	1.00	3.37	C
ATOM	2143	CE2	PHE	162	22.188	71.863	13.627	1.00	3.37	C
ATOM	2144	CZ	PHE	162	22.604	72.961	12.851	1.00	3.37	C
ATOM	2145	H	PHE	162	17.489	72.301	12.635	1.00	0.00	H
ATOM	2146	HA	PHE	162	18.433	71.696	9.943	1.00	0.00	H
ATOM	2147	HB2	PHE	162	18.726	70.153	12.578	1.00	0.00	H
ATOM	2148	HB3	PHE	162	19.558	69.756	11.107	1.00	0.00	H
ATOM	2149	HD1	PHE	162	20.233	72.848	10.405	1.00	0.00	H
ATOM	2150	HD2	PHE	162	20.758	70.264	13.832	1.00	0.00	H
ATOM	2151	HE1	PHE	162	22.198	74.157	11.098	1.00	0.00	H
ATOM	2152	HE2	PHE	162	22.732	71.599	14.522	1.00	0.00	H
ATOM	2153	HZ	PHE	162	23.462	73.543	13.150	1.00	0.00	H
ATOM	2154	N	ASP	163	16.243	69.709	11.354	1.00	3.20	N
ATOM	2155	CA	ASP	163	15.259	68.670	11.158	1.00	3.20	C
ATOM	2156	C	ASP	163	14.291	68.979	10.011	1.00	3.20	C
ATOM	2157	O	ASP	163	14.196	68.241	9.027	1.00	3.20	O

ATOM	2158	CB	ASP	163	14.529	68.417	12.505	1.00	3.20	C
ATOM	2159	CG	ASP	163	13.350	67.441	12.444	1.00	3.20	C
ATOM	2160	OD1	ASP	163	12.221	67.899	12.173	1.00	3.20	O
ATOM	2161	OD2	ASP	163	13.582	66.253	12.753	1.00	3.20	O1-
ATOM	2162	H	ASP	163	16.301	70.118	12.278	1.00	0.00	H
ATOM	2163	HA	ASP	163	15.776	67.743	10.912	1.00	0.00	H
ATOM	2164	HB2	ASP	163	15.237	68.045	13.245	1.00	0.00	H
ATOM	2165	HB3	ASP	163	14.141	69.353	12.904	1.00	0.00	H
ATOM	2166	N	GLN	164	13.666	70.158	10.095	1.00	3.14	N
ATOM	2167	CA	GLN	164	12.722	70.624	9.098	1.00	3.14	C
ATOM	2168	C	GLN	164	13.350	71.018	7.756	1.00	3.14	C
ATOM	2169	O	GLN	164	12.621	71.114	6.768	1.00	3.14	O
ATOM	2170	CB	GLN	164	11.856	71.759	9.692	1.00	3.14	C
ATOM	2171	CG	GLN	164	10.556	71.234	10.333	1.00	3.14	C
ATOM	2172	CD	GLN	164	9.567	70.628	9.332	1.00	3.14	C
ATOM	2173	OE1	GLN	164	8.524	70.112	9.715	1.00	3.14	O
ATOM	2174	NE2	GLN	164	9.850	70.674	8.034	1.00	3.14	N
ATOM	2175	H	GLN	164	13.769	70.682	10.957	1.00	0.00	H
ATOM	2176	HA	GLN	164	12.100	69.758	8.868	1.00	0.00	H
ATOM	2177	HB2	GLN	164	12.425	72.312	10.441	1.00	0.00	H
ATOM	2178	HB3	GLN	164	11.601	72.496	8.932	1.00	0.00	H
ATOM	2179	HG2	GLN	164	10.799	70.476	11.082	1.00	0.00	H
ATOM	2180	HG3	GLN	164	10.053	72.039	10.869	1.00	0.00	H
ATOM	2181	HE22	GLN	164	9.307	70.159	7.359	1.00	0.00	H
ATOM	2182	HE21	GLN	164	10.719	71.089	7.722	1.00	0.00	H
ATOM	2183	N	PHE	165	14.671	71.196	7.702	1.00	3.06	N
ATOM	2184	CA	PHE	165	15.426	71.401	6.478	1.00	3.06	C
ATOM	2185	C	PHE	165	15.658	70.028	5.854	1.00	3.06	C
ATOM	2186	O	PHE	165	15.228	69.803	4.720	1.00	3.06	O
ATOM	2187	CB	PHE	165	16.761	72.116	6.814	1.00	3.06	C
ATOM	2188	CG	PHE	165	17.824	72.126	5.727	1.00	3.06	C
ATOM	2189	CD1	PHE	165	17.721	73.008	4.634	1.00	3.06	C
ATOM	2190	CD2	PHE	165	18.914	71.232	5.800	1.00	3.06	C
ATOM	2191	CE1	PHE	165	18.679	72.970	3.602	1.00	3.06	C
ATOM	2192	CE2	PHE	165	19.877	71.201	4.774	1.00	3.06	C
ATOM	2193	CZ	PHE	165	19.756	72.066	3.670	1.00	3.06	C
ATOM	2194	H	PHE	165	15.185	71.074	8.565	1.00	0.00	H
ATOM	2195	HA	PHE	165	14.862	72.014	5.774	1.00	0.00	H
ATOM	2196	HB2	PHE	165	16.542	73.152	7.076	1.00	0.00	H
ATOM	2197	HB3	PHE	165	17.211	71.692	7.707	1.00	0.00	H
ATOM	2198	HD1	PHE	165	16.893	73.698	4.574	1.00	0.00	H
ATOM	2199	HD2	PHE	165	19.006	70.555	6.637	1.00	0.00	H
ATOM	2200	HE1	PHE	165	18.583	73.622	2.747	1.00	0.00	H
ATOM	2201	HE2	PHE	165	20.702	70.506	4.828	1.00	0.00	H
ATOM	2202	HZ	PHE	165	20.486	72.034	2.874	1.00	0.00	H
ATOM	2203	N	ILE	166	16.263	69.094	6.609	1.00	3.06	N
ATOM	2204	CA	ILE	166	16.584	67.768	6.105	1.00	3.06	C
ATOM	2205	C	ILE	166	15.346	66.950	5.683	1.00	3.06	C
ATOM	2206	O	ILE	166	15.386	66.226	4.685	1.00	3.06	O
ATOM	2207	CB	ILE	166	17.531	66.951	7.032	1.00	3.06	C
ATOM	2208	CG1	ILE	166	16.910	66.493	8.373	1.00	3.06	C
ATOM	2209	CG2	ILE	166	18.883	67.665	7.226	1.00	3.06	C
ATOM	2210	CD1	ILE	166	17.756	65.478	9.154	1.00	3.06	C
ATOM	2211	H	ILE	166	16.526	69.313	7.568	1.00	0.00	H
ATOM	2212	HA	ILE	166	17.137	67.932	5.177	1.00	0.00	H
ATOM	2213	HB	ILE	166	17.755	66.036	6.480	1.00	0.00	H
ATOM	2214	HG12	ILE	166	16.733	67.358	8.999	1.00	0.00	H
ATOM	2215	HG13	ILE	166	15.933	66.041	8.211	1.00	0.00	H
ATOM	2216	HG21	ILE	166	19.624	67.005	7.674	1.00	0.00	H
ATOM	2217	HG22	ILE	166	19.292	67.997	6.271	1.00	0.00	H
ATOM	2218	HG23	ILE	166	18.794	68.539	7.868	1.00	0.00	H
ATOM	2219	HD11	ILE	166	17.222	65.147	10.046	1.00	0.00	H
ATOM	2220	HD12	ILE	166	17.974	64.594	8.553	1.00	0.00	H

ATOM	2221	HD13	ILE	166	18.701	65.907	9.486	1.00	0.00	H
ATOM	2222	N	GLY	167	14.214	67.144	6.366	1.00	2.74	N
ATOM	2223	CA	GLY	167	13.001	66.385	6.105	1.00	2.74	C
ATOM	2224	C	GLY	167	12.195	66.952	4.951	1.00	2.74	C
ATOM	2225	O	GLY	167	11.459	66.202	4.312	1.00	2.74	O
ATOM	2226	H	GLY	167	14.262	67.686	7.229	1.00	0.00	H
ATOM	2227	HA2	GLY	167	13.231	65.335	5.916	1.00	0.00	H
ATOM	2228	HA3	GLY	167	12.381	66.408	7.002	1.00	0.00	H
ATOM	2229	N	THR	168	12.385	68.236	4.619	1.00	2.47	N
ATOM	2230	CA	THR	168	11.718	68.819	3.462	1.00	2.47	C
ATOM	2231	C	THR	168	12.582	68.713	2.208	1.00	2.47	C
ATOM	2232	O	THR	168	12.040	68.644	1.109	1.00	2.47	O
ATOM	2233	CB	THR	168	11.249	70.265	3.741	1.00	2.47	C
ATOM	2234	OG1	THR	168	10.721	70.375	5.054	1.00	2.47	O
ATOM	2235	CG2	THR	168	10.086	70.666	2.821	1.00	2.47	C
ATOM	2236	H	THR	168	13.065	68.780	5.130	1.00	0.00	H
ATOM	2237	HA	THR	168	10.806	68.256	3.265	1.00	0.00	H
ATOM	2238	HB	THR	168	12.079	70.964	3.626	1.00	0.00	H
ATOM	2239	HG1	THR	168	11.470	70.478	5.644	1.00	0.00	H
ATOM	2240	HG21	THR	168	9.814	71.712	2.936	1.00	0.00	H
ATOM	2241	HG22	THR	168	10.335	70.503	1.774	1.00	0.00	H
ATOM	2242	HG23	THR	168	9.193	70.075	3.021	1.00	0.00	H
ATOM	2243	N	ALA	169	13.904	68.568	2.356	1.00	2.19	N
ATOM	2244	CA	ALA	169	14.762	68.186	1.247	1.00	2.19	C
ATOM	2245	C	ALA	169	14.437	66.770	0.759	1.00	2.19	C
ATOM	2246	O	ALA	169	14.278	66.544	-0.437	1.00	2.19	O
ATOM	2247	CB	ALA	169	16.233	68.275	1.685	1.00	2.19	C
ATOM	2248	H	ALA	169	14.321	68.662	3.275	1.00	0.00	H
ATOM	2249	HA	ALA	169	14.580	68.862	0.417	1.00	0.00	H
ATOM	2250	HB1	ALA	169	16.900	68.024	0.859	1.00	0.00	H
ATOM	2251	HB2	ALA	169	16.486	69.285	2.010	1.00	0.00	H
ATOM	2252	HB3	ALA	169	16.456	67.597	2.509	1.00	0.00	H
ATOM	2253	N	SER	170	14.239	65.844	1.699	1.00	2.01	N
ATOM	2254	CA	SER	170	13.860	64.468	1.413	1.00	2.01	C
ATOM	2255	C	SER	170	12.408	64.341	0.955	1.00	2.01	C
ATOM	2256	O	SER	170	12.092	63.456	0.162	1.00	2.01	O
ATOM	2257	CB	SER	170	14.074	63.680	2.731	1.00	2.01	C
ATOM	2258	OG	SER	170	13.983	62.279	2.560	1.00	2.01	O
ATOM	2259	H	SER	170	14.420	66.094	2.663	1.00	0.00	H
ATOM	2260	HA	SER	170	14.539	64.067	0.665	1.00	0.00	H
ATOM	2261	HB2	SER	170	15.064	63.893	3.137	1.00	0.00	H
ATOM	2262	HB3	SER	170	13.352	63.993	3.489	1.00	0.00	H
ATOM	2263	HG	SER	170	14.110	61.852	3.394	1.00	0.00	H
ATOM	2264	N	LEU	171	11.532	65.267	1.378	1.00	1.74	N
ATOM	2265	CA	LEU	171	10.205	65.376	0.803	1.00	1.74	C
ATOM	2266	C	LEU	171	10.303	65.699	-0.694	1.00	1.74	C
ATOM	2267	O	LEU	171	9.865	64.904	-1.522	1.00	1.74	O
ATOM	2268	CB	LEU	171	9.318	66.407	1.541	1.00	1.74	C
ATOM	2269	CG	LEU	171	7.881	66.550	0.973	1.00	1.74	C
ATOM	2270	CD1	LEU	171	7.122	65.215	0.973	1.00	1.74	C
ATOM	2271	CD2	LEU	171	7.082	67.620	1.727	1.00	1.74	C
ATOM	2272	H	LEU	171	11.820	65.932	2.078	1.00	0.00	H
ATOM	2273	HA	LEU	171	9.741	64.393	0.901	1.00	0.00	H
ATOM	2274	HB2	LEU	171	9.265	66.140	2.597	1.00	0.00	H
ATOM	2275	HB3	LEU	171	9.792	67.385	1.496	1.00	0.00	H
ATOM	2276	HG	LEU	171	7.941	66.892	-0.061	1.00	0.00	H
ATOM	2277	HD11	LEU	171	6.077	65.350	0.693	1.00	0.00	H
ATOM	2278	HD12	LEU	171	7.548	64.507	0.263	1.00	0.00	H
ATOM	2279	HD13	LEU	171	7.158	64.749	1.957	1.00	0.00	H
ATOM	2280	HD21	LEU	171	6.121	67.802	1.246	1.00	0.00	H
ATOM	2281	HD22	LEU	171	6.882	67.304	2.749	1.00	0.00	H
ATOM	2282	HD23	LEU	171	7.616	68.570	1.761	1.00	0.00	H
ATOM	2283	N	ILE	172	10.900	66.849	-1.030	1.00	1.75	N

ATOM	2284	CA	ILE	172	10.949	67.347	-2.399	1.00	1.75	C
ATOM	2285	C	ILE	172	11.748	66.466	-3.349	1.00	1.75	C
ATOM	2286	O	ILE	172	11.351	66.340	-4.504	1.00	1.75	O
ATOM	2287	CB	ILE	172	11.379	68.833	-2.433	1.00	1.75	C
ATOM	2288	CG1	ILE	172	10.419	69.723	-1.618	1.00	1.75	C
ATOM	2289	CG2	ILE	172	11.503	69.407	-3.860	1.00	1.75	C
ATOM	2290	CD1	ILE	172	8.936	69.620	-1.955	1.00	1.75	C
ATOM	2291	H	ILE	172	11.294	67.432	-0.300	1.00	0.00	H
ATOM	2292	HA	ILE	172	9.943	67.315	-2.799	1.00	0.00	H
ATOM	2293	HB	ILE	172	12.368	68.912	-1.977	1.00	0.00	H
ATOM	2294	HG12	ILE	172	10.535	69.555	-0.552	1.00	0.00	H
ATOM	2295	HG13	ILE	172	10.695	70.753	-1.793	1.00	0.00	H
ATOM	2296	HG21	ILE	172	11.651	70.485	-3.845	1.00	0.00	H
ATOM	2297	HG22	ILE	172	12.352	68.974	-4.389	1.00	0.00	H
ATOM	2298	HG23	ILE	172	10.611	69.205	-4.454	1.00	0.00	H
ATOM	2299	HD11	ILE	172	8.404	70.380	-1.386	1.00	0.00	H
ATOM	2300	HD12	ILE	172	8.749	69.793	-3.015	1.00	0.00	H
ATOM	2301	HD13	ILE	172	8.515	68.653	-1.680	1.00	0.00	H
ATOM	2302	N	VAL	173	12.814	65.821	-2.871	1.00	1.88	N
ATOM	2303	CA	VAL	173	13.583	64.929	-3.715	1.00	1.88	C
ATOM	2304	C	VAL	173	12.771	63.693	-4.134	1.00	1.88	C
ATOM	2305	O	VAL	173	12.748	63.325	-5.309	1.00	1.88	O
ATOM	2306	CB	VAL	173	14.936	64.547	-3.016	1.00	1.88	C
ATOM	2307	CG1	VAL	173	14.852	63.421	-1.974	1.00	1.88	C
ATOM	2308	CG2	VAL	173	16.059	64.188	-4.000	1.00	1.88	C
ATOM	2309	H	VAL	173	13.121	65.976	-1.917	1.00	0.00	H
ATOM	2310	HA	VAL	173	13.834	65.481	-4.622	1.00	0.00	H
ATOM	2311	HB	VAL	173	15.291	65.435	-2.494	1.00	0.00	H
ATOM	2312	HG11	VAL	173	15.740	63.394	-1.343	1.00	0.00	H
ATOM	2313	HG12	VAL	173	13.990	63.558	-1.338	1.00	0.00	H
ATOM	2314	HG13	VAL	173	14.754	62.439	-2.440	1.00	0.00	H
ATOM	2315	HG21	VAL	173	16.928	63.779	-3.484	1.00	0.00	H
ATOM	2316	HG22	VAL	173	15.732	63.448	-4.730	1.00	0.00	H
ATOM	2317	HG23	VAL	173	16.411	65.070	-4.527	1.00	0.00	H
ATOM	2318	N	CYS	174	12.041	63.105	-3.179	1.00	2.12	N
ATOM	2319	CA	CYS	174	11.228	61.927	-3.416	1.00	2.12	C
ATOM	2320	C	CYS	174	10.003	62.276	-4.256	1.00	2.12	C
ATOM	2321	O	CYS	174	9.617	61.470	-5.093	1.00	2.12	O
ATOM	2322	CB	CYS	174	10.738	61.333	-2.080	1.00	2.12	C
ATOM	2323	SG	CYS	174	12.114	60.589	-1.159	1.00	2.12	S
ATOM	2324	H	CYS	174	12.067	63.480	-2.240	1.00	0.00	H
ATOM	2325	HA	CYS	174	11.814	61.177	-3.951	1.00	0.00	H
ATOM	2326	HB2	CYS	174	10.245	62.084	-1.460	1.00	0.00	H
ATOM	2327	HB3	CYS	174	10.007	60.544	-2.265	1.00	0.00	H
ATOM	2328	HG	CYS	174	12.509	61.733	-0.577	1.00	0.00	H
ATOM	2329	N	VAL	175	9.428	63.477	-4.093	1.00	2.36	N
ATOM	2330	CA	VAL	175	8.273	63.886	-4.881	1.00	2.36	C
ATOM	2331	C	VAL	175	8.616	64.514	-6.244	1.00	2.36	C
ATOM	2332	O	VAL	175	7.713	64.944	-6.958	1.00	2.36	O
ATOM	2333	CB	VAL	175	7.256	64.736	-4.061	1.00	2.36	C
ATOM	2334	CG1	VAL	175	6.803	64.013	-2.778	1.00	2.36	C
ATOM	2335	CG2	VAL	175	7.653	66.185	-3.739	1.00	2.36	C
ATOM	2336	H	VAL	175	9.776	64.108	-3.378	1.00	0.00	H
ATOM	2337	HA	VAL	175	7.709	62.994	-5.153	1.00	0.00	H
ATOM	2338	HB	VAL	175	6.371	64.810	-4.697	1.00	0.00	H
ATOM	2339	HG11	VAL	175	7.590	63.985	-2.029	1.00	0.00	H
ATOM	2340	HG12	VAL	175	5.937	64.501	-2.331	1.00	0.00	H
ATOM	2341	HG13	VAL	175	6.540	62.978	-2.980	1.00	0.00	H
ATOM	2342	HG21	VAL	175	8.154	66.236	-2.779	1.00	0.00	H
ATOM	2343	HG22	VAL	175	8.297	66.629	-4.499	1.00	0.00	H
ATOM	2344	HG23	VAL	175	6.767	66.813	-3.655	1.00	0.00	H
ATOM	2345	N	LEU	176	9.891	64.501	-6.644	1.00	2.52	N
ATOM	2346	CA	LEU	176	10.283	64.569	-8.050	1.00	2.52	C

ATOM	2347	C	LEU	176	10.427	63.141	-8.561	1.00	2.52	C
ATOM	2348	O	LEU	176	9.849	62.779	-9.580	1.00	2.52	O
ATOM	2349	CB	LEU	176	11.632	65.288	-8.189	1.00	2.52	C
ATOM	2350	CG	LEU	176	11.534	66.778	-7.816	1.00	2.52	C
ATOM	2351	CD1	LEU	176	12.848	67.279	-7.226	1.00	2.52	C
ATOM	2352	CD2	LEU	176	11.074	67.650	-8.994	1.00	2.52	C
ATOM	2353	H	LEU	176	10.589	64.208	-5.974	1.00	0.00	H
ATOM	2354	HA	LEU	176	9.532	65.078	-8.658	1.00	0.00	H
ATOM	2355	HB2	LEU	176	12.369	64.781	-7.564	1.00	0.00	H
ATOM	2356	HB3	LEU	176	12.003	65.189	-9.211	1.00	0.00	H
ATOM	2357	HG	LEU	176	10.792	66.885	-7.024	1.00	0.00	H
ATOM	2358	HD11	LEU	176	12.629	68.133	-6.597	1.00	0.00	H
ATOM	2359	HD12	LEU	176	13.306	66.540	-6.572	1.00	0.00	H
ATOM	2360	HD13	LEU	176	13.572	67.556	-7.992	1.00	0.00	H
ATOM	2361	HD21	LEU	176	10.980	68.695	-8.701	1.00	0.00	H
ATOM	2362	HD22	LEU	176	11.773	67.593	-9.829	1.00	0.00	H
ATOM	2363	HD23	LEU	176	10.099	67.327	-9.362	1.00	0.00	H
ATOM	2364	N	ALA	177	11.125	62.307	-7.791	1.00	2.64	N
ATOM	2365	CA	ALA	177	11.499	60.952	-8.149	1.00	2.64	C
ATOM	2366	C	ALA	177	10.372	59.922	-8.138	1.00	2.64	C
ATOM	2367	O	ALA	177	10.597	58.767	-8.501	1.00	2.64	O
ATOM	2368	CB	ALA	177	12.509	60.490	-7.080	1.00	2.64	C
ATOM	2369	H	ALA	177	11.568	62.686	-6.964	1.00	0.00	H
ATOM	2370	HA	ALA	177	11.968	60.953	-9.135	1.00	0.00	H
ATOM	2371	HB1	ALA	177	12.998	59.561	-7.366	1.00	0.00	H
ATOM	2372	HB2	ALA	177	13.291	61.227	-6.912	1.00	0.00	H
ATOM	2373	HB3	ALA	177	12.024	60.317	-6.119	1.00	0.00	H
ATOM	2374	N	ILE	178	9.181	60.346	-7.723	1.00	2.87	N
ATOM	2375	CA	ILE	178	7.957	59.580	-7.779	1.00	2.87	C
ATOM	2376	C	ILE	178	6.869	60.317	-8.575	1.00	2.87	C
ATOM	2377	O	ILE	178	5.674	60.094	-8.394	1.00	2.87	O
ATOM	2378	CB	ILE	178	7.597	58.912	-6.416	1.00	2.87	C
ATOM	2379	CG1	ILE	178	6.814	57.584	-6.562	1.00	2.87	C
ATOM	2380	CG2	ILE	178	6.813	59.838	-5.475	1.00	2.87	C
ATOM	2381	CD1	ILE	178	7.619	56.422	-7.162	1.00	2.87	C
ATOM	2382	H	ILE	178	9.139	61.272	-7.321	1.00	0.00	H
ATOM	2383	HA	ILE	178	8.154	58.754	-8.461	1.00	0.00	H
ATOM	2384	HB	ILE	178	8.536	58.673	-5.914	1.00	0.00	H
ATOM	2385	HG12	ILE	178	6.479	57.266	-5.575	1.00	0.00	H
ATOM	2386	HG13	ILE	178	5.905	57.738	-7.146	1.00	0.00	H
ATOM	2387	HG21	ILE	178	6.809	59.449	-4.456	1.00	0.00	H
ATOM	2388	HG22	ILE	178	7.241	60.835	-5.449	1.00	0.00	H
ATOM	2389	HG23	ILE	178	5.776	59.939	-5.794	1.00	0.00	H
ATOM	2390	HD11	ILE	178	7.049	55.494	-7.111	1.00	0.00	H
ATOM	2391	HD12	ILE	178	7.859	56.588	-8.212	1.00	0.00	H
ATOM	2392	HD13	ILE	178	8.554	56.264	-6.624	1.00	0.00	H
ATOM	2393	N	VAL	179	7.320	61.181	-9.490	1.00	2.97	N
ATOM	2394	CA	VAL	179	6.539	61.887	-10.497	1.00	2.97	C
ATOM	2395	C	VAL	179	7.248	61.848	-11.867	1.00	2.97	C
ATOM	2396	O	VAL	179	6.587	61.863	-12.905	1.00	2.97	O
ATOM	2397	CB	VAL	179	6.167	63.321	-9.999	1.00	2.97	C
ATOM	2398	CG1	VAL	179	5.343	64.128	-11.022	1.00	2.97	C
ATOM	2399	CG2	VAL	179	5.419	63.321	-8.650	1.00	2.97	C
ATOM	2400	H	VAL	179	8.323	61.290	-9.543	1.00	0.00	H
ATOM	2401	HA	VAL	179	5.607	61.337	-10.648	1.00	0.00	H
ATOM	2402	HB	VAL	179	7.100	63.865	-9.843	1.00	0.00	H
ATOM	2403	HG11	VAL	179	5.075	65.109	-10.629	1.00	0.00	H
ATOM	2404	HG12	VAL	179	5.896	64.300	-11.946	1.00	0.00	H
ATOM	2405	HG13	VAL	179	4.420	63.609	-11.280	1.00	0.00	H
ATOM	2406	HG21	VAL	179	5.142	64.332	-8.351	1.00	0.00	H
ATOM	2407	HG22	VAL	179	4.509	62.723	-8.701	1.00	0.00	H
ATOM	2408	HG23	VAL	179	6.036	62.922	-7.845	1.00	0.00	H
ATOM	2409	N	ASP	180	8.588	61.803	-11.859	1.00	3.06	N

ATOM	2410	CA	ASP	180	9.511	61.643	-12.987	1.00	3.06	C
ATOM	2411	C	ASP	180	9.075	60.543	-13.986	1.00	3.06	C
ATOM	2412	O	ASP	180	8.696	59.453	-13.545	1.00	3.06	O
ATOM	2413	CB	ASP	180	10.930	61.446	-12.404	1.00	3.06	C
ATOM	2414	CG	ASP	180	12.080	61.349	-13.403	1.00	3.06	C
ATOM	2415	OD1	ASP	180	11.998	60.540	-14.350	1.00	3.06	O
ATOM	2416	OD2	ASP	180	13.096	62.026	-13.138	1.00	3.06	O1-
ATOM	2417	H	ASP	180	9.043	61.945	-10.965	1.00	0.00	H
ATOM	2418	HA	ASP	180	9.505	62.607	-13.490	1.00	0.00	H
ATOM	2419	HB2	ASP	180	11.164	62.265	-11.726	1.00	0.00	H
ATOM	2420	HB3	ASP	180	10.945	60.537	-11.804	1.00	0.00	H
ATOM	2421	N	PRO	181	9.097	60.794	-15.315	1.00	3.46	N
ATOM	2422	CA	PRO	181	8.652	59.807	-16.298	1.00	3.46	C
ATOM	2423	C	PRO	181	9.666	58.677	-16.555	1.00	3.46	C
ATOM	2424	O	PRO	181	9.266	57.651	-17.109	1.00	3.46	O
ATOM	2425	CB	PRO	181	8.411	60.623	-17.577	1.00	3.46	C
ATOM	2426	CG	PRO	181	9.392	61.782	-17.474	1.00	3.46	C
ATOM	2427	CD	PRO	181	9.496	62.045	-15.970	1.00	3.46	C
ATOM	2428	HA	PRO	181	7.718	59.358	-15.964	1.00	0.00	H
ATOM	2429	HB2	PRO	181	8.543	60.047	-18.495	1.00	0.00	H
ATOM	2430	HB3	PRO	181	7.389	61.004	-17.574	1.00	0.00	H
ATOM	2431	HG2	PRO	181	10.365	61.468	-17.857	1.00	0.00	H
ATOM	2432	HG3	PRO	181	9.084	62.660	-18.041	1.00	0.00	H
ATOM	2433	HD3	PRO	181	8.806	62.838	-15.678	1.00	0.00	H
ATOM	2434	HD2	PRO	181	10.508	62.348	-15.696	1.00	0.00	H
ATOM	2435	N	TYR	182	10.942	58.851	-16.187	1.00	4.17	N
ATOM	2436	CA	TYR	182	12.037	57.920	-16.425	1.00	4.17	C
ATOM	2437	C	TYR	182	12.359	57.166	-15.124	1.00	4.17	C
ATOM	2438	O	TYR	182	12.459	55.937	-15.126	1.00	4.17	O
ATOM	2439	CB	TYR	182	13.303	58.723	-16.813	1.00	4.17	C
ATOM	2440	CG	TYR	182	13.165	59.693	-17.976	1.00	4.17	C
ATOM	2441	CD1	TYR	182	12.937	59.225	-19.287	1.00	4.17	C
ATOM	2442	CD2	TYR	182	13.309	61.079	-17.746	1.00	4.17	C
ATOM	2443	CE1	TYR	182	12.888	60.135	-20.363	1.00	4.17	C
ATOM	2444	CE2	TYR	182	13.275	61.984	-18.824	1.00	4.17	C
ATOM	2445	CZ	TYR	182	13.082	61.511	-20.136	1.00	4.17	C
ATOM	2446	OH	TYR	182	13.127	62.375	-21.190	1.00	4.17	O
ATOM	2447	H	TYR	182	11.200	59.676	-15.629	1.00	0.00	H
ATOM	2448	HA	TYR	182	11.787	57.210	-17.215	1.00	0.00	H
ATOM	2449	HB2	TYR	182	13.677	59.288	-15.957	1.00	0.00	H
ATOM	2450	HB3	TYR	182	14.104	58.025	-17.062	1.00	0.00	H
ATOM	2451	HD1	TYR	182	12.821	58.169	-19.476	1.00	0.00	H
ATOM	2452	HD2	TYR	182	13.471	61.452	-16.743	1.00	0.00	H
ATOM	2453	HE1	TYR	182	12.741	59.777	-21.370	1.00	0.00	H
ATOM	2454	HE2	TYR	182	13.430	63.039	-18.646	1.00	0.00	H
ATOM	2455	HH	TYR	182	13.801	63.071	-21.053	1.00	0.00	H
ATOM	2456	N	ASN	183	12.527	57.890	-14.009	1.00	4.32	N
ATOM	2457	CA	ASN	183	12.904	57.353	-12.702	1.00	4.32	C
ATOM	2458	C	ASN	183	11.620	56.850	-12.024	1.00	4.32	C
ATOM	2459	O	ASN	183	10.928	57.595	-11.332	1.00	4.32	O
ATOM	2460	CB	ASN	183	13.554	58.465	-11.847	1.00	4.32	C
ATOM	2461	CG	ASN	183	14.141	57.925	-10.542	1.00	4.32	C
ATOM	2462	OD1	ASN	183	15.264	57.435	-10.501	1.00	4.32	O
ATOM	2463	ND2	ASN	183	13.379	57.968	-9.456	1.00	4.32	N
ATOM	2464	H	ASN	183	12.356	58.904	-14.080	1.00	0.00	H
ATOM	2465	HA	ASN	183	13.618	56.536	-12.826	1.00	0.00	H
ATOM	2466	HB2	ASN	183	14.362	58.938	-12.406	1.00	0.00	H
ATOM	2467	HB3	ASN	183	12.845	59.259	-11.624	1.00	0.00	H
ATOM	2468	HD22	ASN	183	13.746	57.601	-8.598	1.00	0.00	H
ATOM	2469	HD21	ASN	183	12.422	58.305	-9.529	1.00	0.00	H
ATOM	2470	N	ASN	184	11.331	55.566	-12.254	1.00	4.76	N
ATOM	2471	CA	ASN	184	10.096	54.825	-11.959	1.00	4.76	C
ATOM	2472	C	ASN	184	9.169	54.926	-13.180	1.00	4.76	C

ATOM	2473	O	ASN	184	8.142	55.594	-13.063	1.00	4.76	O
ATOM	2474	CB	ASN	184	9.399	55.155	-10.608	1.00	4.76	C
ATOM	2475	CG	ASN	184	10.246	54.835	-9.381	1.00	4.76	C
ATOM	2476	OD1	ASN	184	10.477	53.671	-9.071	1.00	4.76	O
ATOM	2477	ND2	ASN	184	10.723	55.848	-8.668	1.00	4.76	N
ATOM	2478	H	ASN	184	11.949	55.141	-12.931	1.00	0.00	H
ATOM	2479	HA	ASN	184	10.390	53.779	-11.879	1.00	0.00	H
ATOM	2480	HB2	ASN	184	9.051	56.188	-10.577	1.00	0.00	H
ATOM	2481	HB3	ASN	184	8.491	54.557	-10.524	1.00	0.00	H
ATOM	2482	HD22	ASN	184	11.283	55.651	-7.858	1.00	0.00	H
ATOM	2483	HD21	ASN	184	10.548	56.812	-8.955	1.00	0.00	H
ATOM	2484	N	PRO	185	9.539	54.324	-14.346	1.00	5.05	N
ATOM	2485	CA	PRO	185	8.962	54.577	-15.683	1.00	5.05	C
ATOM	2486	C	PRO	185	7.446	54.818	-15.709	1.00	5.05	C
ATOM	2487	O	PRO	185	6.691	53.883	-15.428	1.00	5.05	O
ATOM	2488	CB	PRO	185	9.398	53.387	-16.546	1.00	5.05	C
ATOM	2489	CG	PRO	185	10.737	52.991	-15.951	1.00	5.05	C
ATOM	2490	CD	PRO	185	10.598	53.311	-14.461	1.00	5.05	C
ATOM	2491	HA	PRO	185	9.479	55.463	-16.048	1.00	0.00	H
ATOM	2492	HB2	PRO	185	8.699	52.555	-16.451	1.00	0.00	H
ATOM	2493	HB3	PRO	185	9.471	53.646	-17.602	1.00	0.00	H
ATOM	2494	HG2	PRO	185	11.003	51.951	-16.142	1.00	0.00	H
ATOM	2495	HG3	PRO	185	11.521	53.618	-16.382	1.00	0.00	H
ATOM	2496	HD3	PRO	185	11.555	53.641	-14.059	1.00	0.00	H
ATOM	2497	HD2	PRO	185	10.294	52.418	-13.912	1.00	0.00	H
ATOM	2498	N	VAL	186	7.055	56.067	-16.013	1.00	5.08	N
ATOM	2499	CA	VAL	186	5.942	56.883	-15.483	1.00	5.08	C
ATOM	2500	C	VAL	186	4.921	56.181	-14.538	1.00	5.08	C
ATOM	2501	O	VAL	186	4.250	55.261	-15.007	1.00	5.08	O
ATOM	2502	CB	VAL	186	5.331	57.725	-16.640	1.00	5.08	C
ATOM	2503	CG1	VAL	186	4.600	56.878	-17.700	1.00	5.08	C
ATOM	2504	CG2	VAL	186	4.436	58.873	-16.141	1.00	5.08	C
ATOM	2505	H	VAL	186	7.821	56.642	-16.357	1.00	0.00	H
ATOM	2506	HA	VAL	186	6.493	57.599	-14.878	1.00	0.00	H
ATOM	2507	HB	VAL	186	6.163	58.200	-17.161	1.00	0.00	H
ATOM	2508	HG11	VAL	186	4.305	57.491	-18.552	1.00	0.00	H
ATOM	2509	HG12	VAL	186	5.239	56.080	-18.077	1.00	0.00	H
ATOM	2510	HG13	VAL	186	3.697	56.421	-17.296	1.00	0.00	H
ATOM	2511	HG21	VAL	186	4.083	59.486	-16.971	1.00	0.00	H
ATOM	2512	HG22	VAL	186	3.558	58.506	-15.607	1.00	0.00	H
ATOM	2513	HG23	VAL	186	4.983	59.534	-15.468	1.00	0.00	H
ATOM	2514	N	PRO	187	4.784	56.570	-13.243	1.00	4.54	N
ATOM	2515	CA	PRO	187	4.070	55.792	-12.208	1.00	4.54	C
ATOM	2516	C	PRO	187	2.607	55.369	-12.488	1.00	4.54	C
ATOM	2517	O	PRO	187	2.313	54.179	-12.332	1.00	4.54	O
ATOM	2518	CB	PRO	187	4.314	56.509	-10.873	1.00	4.54	C
ATOM	2519	CG	PRO	187	4.813	57.900	-11.240	1.00	4.54	C
ATOM	2520	CD	PRO	187	5.341	57.799	-12.669	1.00	4.54	C
ATOM	2521	HA	PRO	187	4.623	54.856	-12.142	1.00	0.00	H
ATOM	2522	HB2	PRO	187	3.440	56.537	-10.223	1.00	0.00	H
ATOM	2523	HB3	PRO	187	5.096	55.988	-10.319	1.00	0.00	H
ATOM	2524	HG2	PRO	187	3.984	58.605	-11.232	1.00	0.00	H
ATOM	2525	HG3	PRO	187	5.561	58.279	-10.542	1.00	0.00	H
ATOM	2526	HD3	PRO	187	6.431	57.733	-12.645	1.00	0.00	H
ATOM	2527	HD2	PRO	187	5.071	58.686	-13.243	1.00	0.00	H
ATOM	2528	N	ARG	188	1.734	56.259	-12.980	1.00	4.46	N
ATOM	2529	CA	ARG	188	0.502	55.997	-13.741	1.00	4.46	C
ATOM	2530	C	ARG	188	-0.799	55.822	-12.927	1.00	4.46	C
ATOM	2531	O	ARG	188	-1.751	55.219	-13.423	1.00	4.46	O
ATOM	2532	CB	ARG	188	0.707	55.030	-14.946	1.00	4.46	C
ATOM	2533	CG	ARG	188	0.366	53.536	-14.722	1.00	4.46	C
ATOM	2534	CD	ARG	188	1.411	52.581	-15.319	1.00	4.46	C
ATOM	2535	NE	ARG	188	2.598	52.566	-14.457	1.00	4.46	N

ATOM	2536	CZ	ARG	188	3.891	52.577	-14.791	1.00	4.46	C
ATOM	2537	NH1	ARG	188	4.292	52.536	-16.060	1.00	4.46	N
ATOM	2538	NH2	ARG	188	4.806	52.637	-13.827	1.00	4.46	N1+
ATOM	2539	H	ARG	188	1.899	57.245	-12.754	1.00	0.00	H
ATOM	2540	HA	ARG	188	0.344	56.973	-14.203	1.00	0.00	H
ATOM	2541	HB2	ARG	188	0.078	55.383	-15.764	1.00	0.00	H
ATOM	2542	HB3	ARG	188	1.722	55.141	-15.321	1.00	0.00	H
ATOM	2543	HG2	ARG	188	0.256	53.322	-13.658	1.00	0.00	H
ATOM	2544	HG3	ARG	188	-0.615	53.323	-15.149	1.00	0.00	H
ATOM	2545	HD2	ARG	188	1.010	51.568	-15.365	1.00	0.00	H
ATOM	2546	HD3	ARG	188	1.658	52.878	-16.338	1.00	0.00	H
ATOM	2547	HE	ARG	188	2.380	52.921	-13.514	1.00	0.00	H
ATOM	2548	HH12	ARG	188	5.238	52.876	-16.235	1.00	0.00	H
ATOM	2549	HH11	ARG	188	3.625	52.577	-16.809	1.00	0.00	H
ATOM	2550	HH22	ARG	188	5.739	52.961	-14.092	1.00	0.00	H
ATOM	2551	HH21	ARG	188	4.541	52.713	-12.857	1.00	0.00	H
ATOM	2552	N	GLY	189	-0.865	56.361	-11.709	1.00	4.10	N
ATOM	2553	CA	GLY	189	-2.016	56.321	-10.805	1.00	4.10	C
ATOM	2554	C	GLY	189	-1.677	55.814	-9.397	1.00	4.10	C
ATOM	2555	O	GLY	189	-2.583	55.514	-8.622	1.00	4.10	O
ATOM	2556	H	GLY	189	-0.025	56.855	-11.369	1.00	0.00	H
ATOM	2557	HA2	GLY	189	-2.415	57.330	-10.717	1.00	0.00	H
ATOM	2558	HA3	GLY	189	-2.819	55.700	-11.204	1.00	0.00	H
ATOM	2559	N	LEU	190	-0.387	55.721	-9.060	1.00	3.70	N
ATOM	2560	CA	LEU	190	0.147	55.328	-7.756	1.00	3.70	C
ATOM	2561	C	LEU	190	0.751	56.527	-7.036	1.00	3.70	C
ATOM	2562	O	LEU	190	0.747	56.557	-5.807	1.00	3.70	O
ATOM	2563	CB	LEU	190	1.290	54.307	-7.985	1.00	3.70	C
ATOM	2564	CG	LEU	190	0.900	53.033	-8.769	1.00	3.70	C
ATOM	2565	CD1	LEU	190	2.147	52.200	-9.100	1.00	3.70	C
ATOM	2566	CD2	LEU	190	-0.139	52.181	-8.021	1.00	3.70	C
ATOM	2567	H	LEU	190	0.273	56.146	-9.719	1.00	0.00	H
ATOM	2568	HA	LEU	190	-0.629	54.895	-7.122	1.00	0.00	H
ATOM	2569	HB2	LEU	190	2.101	54.804	-8.522	1.00	0.00	H
ATOM	2570	HB3	LEU	190	1.713	54.020	-7.021	1.00	0.00	H
ATOM	2571	HG	LEU	190	0.463	53.330	-9.724	1.00	0.00	H
ATOM	2572	HD11	LEU	190	1.888	51.318	-9.686	1.00	0.00	H
ATOM	2573	HD12	LEU	190	2.861	52.782	-9.685	1.00	0.00	H
ATOM	2574	HD13	LEU	190	2.654	51.863	-8.195	1.00	0.00	H
ATOM	2575	HD21	LEU	190	-0.379	51.275	-8.578	1.00	0.00	H
ATOM	2576	HD22	LEU	190	0.223	51.881	-7.038	1.00	0.00	H
ATOM	2577	HD23	LEU	190	-1.074	52.725	-7.882	1.00	0.00	H
ATOM	2578	N	GLU	191	1.274	57.489	-7.804	1.00	3.15	N
ATOM	2579	CA	GLU	191	2.123	58.605	-7.441	1.00	3.15	C
ATOM	2580	C	GLU	191	1.699	59.306	-6.152	1.00	3.15	C
ATOM	2581	O	GLU	191	2.439	59.354	-5.171	1.00	3.15	O
ATOM	2582	CB	GLU	191	2.361	59.591	-8.625	1.00	3.15	C
ATOM	2583	CG	GLU	191	1.263	59.760	-9.712	1.00	3.15	C
ATOM	2584	CD	GLU	191	1.251	58.667	-10.777	1.00	3.15	C
ATOM	2585	OE1	GLU	191	1.129	57.489	-10.387	1.00	3.15	O
ATOM	2586	OE2	GLU	191	1.319	58.956	-11.985	1.00	3.15	O1-
ATOM	2587	H	GLU	191	1.189	57.372	-8.826	1.00	0.00	H
ATOM	2588	HA	GLU	191	3.100	58.169	-7.227	1.00	0.00	H
ATOM	2589	HB2	GLU	191	2.623	60.575	-8.234	1.00	0.00	H
ATOM	2590	HB3	GLU	191	3.270	59.270	-9.134	1.00	0.00	H
ATOM	2591	HG2	GLU	191	0.274	59.832	-9.262	1.00	0.00	H
ATOM	2592	HG3	GLU	191	1.432	60.703	-10.233	1.00	0.00	H
ATOM	2593	N	ALA	192	0.444	59.751	-6.123	1.00	2.75	N
ATOM	2594	CA	ALA	192	-0.152	60.465	-5.006	1.00	2.75	C
ATOM	2595	C	ALA	192	-0.423	59.596	-3.798	1.00	2.75	C
ATOM	2596	O	ALA	192	-0.498	60.144	-2.711	1.00	2.75	O
ATOM	2597	CB	ALA	192	-1.488	61.025	-5.502	1.00	2.75	C
ATOM	2598	H	ALA	192	-0.095	59.618	-6.965	1.00	0.00	H

ATOM	2599	HA	ALA	192	0.491	61.303	-4.751	1.00	0.00	H
ATOM	2600	HB1	ALA	192	-1.974	61.616	-4.725	1.00	0.00	H
ATOM	2601	HB2	ALA	192	-1.344	61.673	-6.366	1.00	0.00	H
ATOM	2602	HB3	ALA	192	-2.169	60.227	-5.801	1.00	0.00	H
ATOM	2603	N	PHE	193	-0.541	58.277	-3.963	1.00	2.45	N
ATOM	2604	CA	PHE	193	-0.656	57.345	-2.854	1.00	2.45	C
ATOM	2605	C	PHE	193	0.731	57.122	-2.252	1.00	2.45	C
ATOM	2606	O	PHE	193	0.908	57.236	-1.039	1.00	2.45	O
ATOM	2607	CB	PHE	193	-1.259	56.010	-3.347	1.00	2.45	C
ATOM	2608	CG	PHE	193	-1.740	55.084	-2.244	1.00	2.45	C
ATOM	2609	CD1	PHE	193	-2.876	55.429	-1.480	1.00	2.45	C
ATOM	2610	CD2	PHE	193	-1.065	53.872	-1.983	1.00	2.45	C
ATOM	2611	CE1	PHE	193	-3.338	54.565	-0.470	1.00	2.45	C
ATOM	2612	CE2	PHE	193	-1.531	53.007	-0.973	1.00	2.45	C
ATOM	2613	CZ	PHE	193	-2.669	53.352	-0.219	1.00	2.45	C
ATOM	2614	H	PHE	193	-0.324	57.890	-4.876	1.00	0.00	H
ATOM	2615	HA	PHE	193	-1.323	57.758	-2.103	1.00	0.00	H
ATOM	2616	HB2	PHE	193	-2.119	56.216	-3.986	1.00	0.00	H
ATOM	2617	HB3	PHE	193	-0.554	55.466	-3.975	1.00	0.00	H
ATOM	2618	HD1	PHE	193	-3.399	56.358	-1.665	1.00	0.00	H
ATOM	2619	HD2	PHE	193	-0.191	53.598	-2.556	1.00	0.00	H
ATOM	2620	HE1	PHE	193	-4.204	54.837	0.117	1.00	0.00	H
ATOM	2621	HE2	PHE	193	-1.013	52.080	-0.776	1.00	0.00	H
ATOM	2622	HZ	PHE	193	-3.023	52.693	0.561	1.00	0.00	H
ATOM	2623	N	THR	194	1.737	56.897	-3.107	1.00	2.11	N
ATOM	2624	CA	THR	194	3.123	56.774	-2.699	1.00	2.11	C
ATOM	2625	C	THR	194	3.694	58.044	-2.067	1.00	2.11	C
ATOM	2626	O	THR	194	4.406	57.918	-1.072	1.00	2.11	O
ATOM	2627	CB	THR	194	3.981	56.215	-3.861	1.00	2.11	C
ATOM	2628	OG1	THR	194	3.753	56.884	-5.082	1.00	2.11	O
ATOM	2629	CG2	THR	194	3.704	54.729	-4.130	1.00	2.11	C
ATOM	2630	H	THR	194	1.551	56.835	-4.107	1.00	0.00	H
ATOM	2631	HA	THR	194	3.151	56.028	-1.904	1.00	0.00	H
ATOM	2632	HB	THR	194	5.038	56.326	-3.607	1.00	0.00	H
ATOM	2633	HG1	THR	194	3.759	57.828	-4.954	1.00	0.00	H
ATOM	2634	HG21	THR	194	4.343	54.352	-4.929	1.00	0.00	H
ATOM	2635	HG22	THR	194	3.896	54.125	-3.243	1.00	0.00	H
ATOM	2636	HG23	THR	194	2.669	54.564	-4.430	1.00	0.00	H
ATOM	2637	N	VAL	195	3.328	59.252	-2.529	1.00	1.86	N
ATOM	2638	CA	VAL	195	3.750	60.463	-1.830	1.00	1.86	C
ATOM	2639	C	VAL	195	3.145	60.617	-0.432	1.00	1.86	C
ATOM	2640	O	VAL	195	3.768	61.219	0.441	1.00	1.86	O
ATOM	2641	CB	VAL	195	3.604	61.786	-2.633	1.00	1.86	C
ATOM	2642	CG1	VAL	195	4.327	61.784	-3.987	1.00	1.86	C
ATOM	2643	CG2	VAL	195	2.175	62.310	-2.750	1.00	1.86	C
ATOM	2644	H	VAL	195	2.791	59.337	-3.390	1.00	0.00	H
ATOM	2645	HA	VAL	195	4.825	60.356	-1.671	1.00	0.00	H
ATOM	2646	HB	VAL	195	4.135	62.541	-2.048	1.00	0.00	H
ATOM	2647	HG11	VAL	195	4.457	62.800	-4.362	1.00	0.00	H
ATOM	2648	HG12	VAL	195	5.316	61.344	-3.890	1.00	0.00	H
ATOM	2649	HG13	VAL	195	3.796	61.241	-4.758	1.00	0.00	H
ATOM	2650	HG21	VAL	195	2.076	63.024	-3.566	1.00	0.00	H
ATOM	2651	HG22	VAL	195	1.501	61.487	-2.922	1.00	0.00	H
ATOM	2652	HG23	VAL	195	1.855	62.801	-1.830	1.00	0.00	H
ATOM	2653	N	GLY	196	1.996	59.987	-0.189	1.00	1.82	N
ATOM	2654	CA	GLY	196	1.374	60.010	1.115	1.00	1.82	C
ATOM	2655	C	GLY	196	1.997	58.991	2.061	1.00	1.82	C
ATOM	2656	O	GLY	196	2.124	59.233	3.260	1.00	1.82	O
ATOM	2657	H	GLY	196	1.551	59.466	-0.934	1.00	0.00	H
ATOM	2658	HA2	GLY	196	1.362	61.010	1.541	1.00	0.00	H
ATOM	2659	HA3	GLY	196	0.360	59.692	1.086	1.00	0.00	H
ATOM	2660	N	LEU	197	2.454	57.867	1.509	1.00	1.96	N
ATOM	2661	CA	LEU	197	3.226	56.884	2.244	1.00	1.96	C

ATOM	2662	C	LEU	197	4.651	57.366	2.549	1.00	1.96	C
ATOM	2663	O	LEU	197	5.238	56.954	3.548	1.00	1.96	O
ATOM	2664	CB	LEU	197	3.324	55.582	1.415	1.00	1.96	C
ATOM	2665	CG	LEU	197	2.002	54.797	1.263	1.00	1.96	C
ATOM	2666	CD1	LEU	197	2.133	53.715	0.180	1.00	1.96	C
ATOM	2667	CD2	LEU	197	1.557	54.162	2.591	1.00	1.96	C
ATOM	2668	H	LEU	197	2.276	57.709	0.525	1.00	0.00	H
ATOM	2669	HA	LEU	197	2.738	56.675	3.197	1.00	0.00	H
ATOM	2670	HB2	LEU	197	3.721	55.822	0.429	1.00	0.00	H
ATOM	2671	HB3	LEU	197	4.066	54.918	1.865	1.00	0.00	H
ATOM	2672	HG	LEU	197	1.216	55.481	0.939	1.00	0.00	H
ATOM	2673	HD11	LEU	197	1.205	53.156	0.070	1.00	0.00	H
ATOM	2674	HD12	LEU	197	2.360	54.152	-0.792	1.00	0.00	H
ATOM	2675	HD13	LEU	197	2.923	53.003	0.421	1.00	0.00	H
ATOM	2676	HD21	LEU	197	0.693	53.514	2.447	1.00	0.00	H
ATOM	2677	HD22	LEU	197	2.349	53.556	3.032	1.00	0.00	H
ATOM	2678	HD23	LEU	197	1.268	54.919	3.319	1.00	0.00	H
ATOM	2679	N	VAL	198	5.193	58.265	1.724	1.00	1.92	N
ATOM	2680	CA	VAL	198	6.433	58.967	2.006	1.00	1.92	C
ATOM	2681	C	VAL	198	6.255	59.932	3.174	1.00	1.92	C
ATOM	2682	O	VAL	198	7.037	59.863	4.124	1.00	1.92	O
ATOM	2683	CB	VAL	198	6.948	59.674	0.712	1.00	1.92	C
ATOM	2684	CG1	VAL	198	8.022	60.761	0.936	1.00	1.92	C
ATOM	2685	CG2	VAL	198	7.501	58.645	-0.293	1.00	1.92	C
ATOM	2686	H	VAL	198	4.708	58.473	0.859	1.00	0.00	H
ATOM	2687	HA	VAL	198	7.188	58.235	2.287	1.00	0.00	H
ATOM	2688	HB	VAL	198	6.104	60.173	0.242	1.00	0.00	H
ATOM	2689	HG11	VAL	198	8.390	61.152	-0.013	1.00	0.00	H
ATOM	2690	HG12	VAL	198	7.633	61.616	1.489	1.00	0.00	H
ATOM	2691	HG13	VAL	198	8.881	60.369	1.483	1.00	0.00	H
ATOM	2692	HG21	VAL	198	7.691	59.104	-1.264	1.00	0.00	H
ATOM	2693	HG22	VAL	198	8.441	58.219	0.059	1.00	0.00	H
ATOM	2694	HG23	VAL	198	6.818	57.813	-0.453	1.00	0.00	H
ATOM	2695	N	VAL	199	5.231	60.802	3.138	1.00	2.01	N
ATOM	2696	CA	VAL	199	5.002	61.705	4.259	1.00	2.01	C
ATOM	2697	C	VAL	199	4.767	61.026	5.621	1.00	2.01	C
ATOM	2698	O	VAL	199	5.247	61.501	6.650	1.00	2.01	O
ATOM	2699	CB	VAL	199	4.049	62.901	3.989	1.00	2.01	C
ATOM	2700	CG1	VAL	199	4.728	63.991	3.148	1.00	2.01	C
ATOM	2701	CG2	VAL	199	2.708	62.542	3.369	1.00	2.01	C
ATOM	2702	H	VAL	199	4.633	60.859	2.320	1.00	0.00	H
ATOM	2703	HA	VAL	199	5.972	62.182	4.420	1.00	0.00	H
ATOM	2704	HB	VAL	199	3.831	63.356	4.959	1.00	0.00	H
ATOM	2705	HG11	VAL	199	4.065	64.842	2.990	1.00	0.00	H
ATOM	2706	HG12	VAL	199	5.623	64.367	3.643	1.00	0.00	H
ATOM	2707	HG13	VAL	199	5.014	63.605	2.169	1.00	0.00	H
ATOM	2708	HG21	VAL	199	1.972	63.324	3.551	1.00	0.00	H
ATOM	2709	HG22	VAL	199	2.796	62.437	2.294	1.00	0.00	H
ATOM	2710	HG23	VAL	199	2.341	61.613	3.786	1.00	0.00	H
ATOM	2711	N	LEU	200	4.157	59.843	5.595	1.00	2.20	N
ATOM	2712	CA	LEU	200	4.026	58.935	6.722	1.00	2.20	C
ATOM	2713	C	LEU	200	5.347	58.521	7.335	1.00	2.20	C
ATOM	2714	O	LEU	200	5.516	58.659	8.548	1.00	2.20	O
ATOM	2715	CB	LEU	200	3.200	57.716	6.211	1.00	2.20	C
ATOM	2716	CG	LEU	200	2.936	56.459	7.088	1.00	2.20	C
ATOM	2717	CD1	LEU	200	1.952	55.540	6.336	1.00	2.20	C
ATOM	2718	CD2	LEU	200	4.166	55.602	7.436	1.00	2.20	C
ATOM	2719	H	LEU	200	3.791	59.541	4.701	1.00	0.00	H
ATOM	2720	HA	LEU	200	3.461	59.452	7.496	1.00	0.00	H
ATOM	2721	HB2	LEU	200	2.228	58.115	5.916	1.00	0.00	H
ATOM	2722	HB3	LEU	200	3.641	57.362	5.288	1.00	0.00	H
ATOM	2723	HG	LEU	200	2.486	56.777	8.027	1.00	0.00	H
ATOM	2724	HD11	LEU	200	1.604	54.730	6.975	1.00	0.00	H

ATOM	2725	HD12	LEU	200	1.074	56.068	5.964	1.00	0.00	H
ATOM	2726	HD13	LEU	200	2.433	55.087	5.468	1.00	0.00	H
ATOM	2727	HD21	LEU	200	3.869	54.629	7.825	1.00	0.00	H
ATOM	2728	HD22	LEU	200	4.780	55.420	6.556	1.00	0.00	H
ATOM	2729	HD23	LEU	200	4.787	56.051	8.209	1.00	0.00	H
ATOM	2730	N	VAL	201	6.270	57.980	6.530	1.00	2.34	N
ATOM	2731	CA	VAL	201	7.528	57.491	7.071	1.00	2.34	C
ATOM	2732	C	VAL	201	8.405	58.632	7.598	1.00	2.34	C
ATOM	2733	O	VAL	201	9.077	58.453	8.613	1.00	2.34	O
ATOM	2734	CB	VAL	201	8.306	56.579	6.082	1.00	2.34	C
ATOM	2735	CG1	VAL	201	7.565	55.256	5.820	1.00	2.34	C
ATOM	2736	CG2	VAL	201	8.690	57.231	4.748	1.00	2.34	C
ATOM	2737	H	VAL	201	6.095	57.902	5.535	1.00	0.00	H
ATOM	2738	HA	VAL	201	7.292	56.873	7.940	1.00	0.00	H
ATOM	2739	HB	VAL	201	9.242	56.313	6.578	1.00	0.00	H
ATOM	2740	HG11	VAL	201	8.189	54.565	5.252	1.00	0.00	H
ATOM	2741	HG12	VAL	201	7.291	54.760	6.751	1.00	0.00	H
ATOM	2742	HG13	VAL	201	6.655	55.418	5.243	1.00	0.00	H
ATOM	2743	HG21	VAL	201	9.337	56.578	4.162	1.00	0.00	H
ATOM	2744	HG22	VAL	201	7.799	57.414	4.158	1.00	0.00	H
ATOM	2745	HG23	VAL	201	9.217	58.177	4.877	1.00	0.00	H
ATOM	2746	N	ILE	202	8.319	59.814	6.976	1.00	2.25	N
ATOM	2747	CA	ILE	202	8.935	61.043	7.456	1.00	2.25	C
ATOM	2748	C	ILE	202	8.386	61.462	8.813	1.00	2.25	C
ATOM	2749	O	ILE	202	9.148	61.678	9.756	1.00	2.25	O
ATOM	2750	CB	ILE	202	8.814	62.169	6.374	1.00	2.25	C
ATOM	2751	CG1	ILE	202	9.597	61.796	5.088	1.00	2.25	C
ATOM	2752	CG2	ILE	202	9.278	63.558	6.878	1.00	2.25	C
ATOM	2753	CD1	ILE	202	9.324	62.710	3.880	1.00	2.25	C
ATOM	2754	H	ILE	202	7.742	59.871	6.143	1.00	0.00	H
ATOM	2755	HA	ILE	202	9.999	60.843	7.603	1.00	0.00	H
ATOM	2756	HB	ILE	202	7.760	62.262	6.111	1.00	0.00	H
ATOM	2757	HG12	ILE	202	10.667	61.789	5.298	1.00	0.00	H
ATOM	2758	HG13	ILE	202	9.358	60.778	4.785	1.00	0.00	H
ATOM	2759	HG21	ILE	202	9.169	64.327	6.114	1.00	0.00	H
ATOM	2760	HG22	ILE	202	8.695	63.905	7.731	1.00	0.00	H
ATOM	2761	HG23	ILE	202	10.325	63.540	7.181	1.00	0.00	H
ATOM	2762	HD11	ILE	202	9.822	62.325	2.989	1.00	0.00	H
ATOM	2763	HD12	ILE	202	8.258	62.770	3.661	1.00	0.00	H
ATOM	2764	HD13	ILE	202	9.697	63.722	4.034	1.00	0.00	H
ATOM	2765	N	GLY	203	7.060	61.482	8.924	1.00	2.34	N
ATOM	2766	CA	GLY	203	6.383	61.820	10.154	1.00	2.34	C
ATOM	2767	C	GLY	203	6.657	60.809	11.264	1.00	2.34	C
ATOM	2768	O	GLY	203	6.781	61.174	12.426	1.00	2.34	O
ATOM	2769	H	GLY	203	6.487	61.344	8.094	1.00	0.00	H
ATOM	2770	HA2	GLY	203	6.677	62.817	10.483	1.00	0.00	H
ATOM	2771	HA3	GLY	203	5.319	61.858	9.935	1.00	0.00	H
ATOM	2772	N	THR	204	6.773	59.526	10.929	1.00	2.62	N
ATOM	2773	CA	THR	204	6.944	58.487	11.931	1.00	2.62	C
ATOM	2774	C	THR	204	8.383	58.396	12.431	1.00	2.62	C
ATOM	2775	O	THR	204	8.595	58.038	13.588	1.00	2.62	O
ATOM	2776	CB	THR	204	6.568	57.121	11.285	1.00	2.62	C
ATOM	2777	OG1	THR	204	5.248	57.165	10.778	1.00	2.62	O
ATOM	2778	CG2	THR	204	6.579	55.924	12.251	1.00	2.62	C
ATOM	2779	H	THR	204	6.628	59.273	9.958	1.00	0.00	H
ATOM	2780	HA	THR	204	6.287	58.675	12.784	1.00	0.00	H
ATOM	2781	HB	THR	204	7.242	56.907	10.453	1.00	0.00	H
ATOM	2782	HG1	THR	204	5.247	57.755	10.021	1.00	0.00	H
ATOM	2783	HG21	THR	204	6.270	55.010	11.747	1.00	0.00	H
ATOM	2784	HG22	THR	204	7.572	55.735	12.661	1.00	0.00	H
ATOM	2785	HG23	THR	204	5.901	56.090	13.087	1.00	0.00	H
ATOM	2786	N	SER	205	9.357	58.687	11.566	1.00	2.88	N
ATOM	2787	CA	SER	205	10.753	58.662	11.949	1.00	2.88	C

ATOM	2788	C	SER	205	11.128	59.939	12.715	1.00	2.88	C
ATOM	2789	O	SER	205	11.725	59.847	13.786	1.00	2.88	O
ATOM	2790	CB	SER	205	11.648	58.466	10.702	1.00	2.88	C
ATOM	2791	OG	SER	205	11.382	59.370	9.644	1.00	2.88	O
ATOM	2792	H	SER	205	9.124	59.014	10.637	1.00	0.00	H
ATOM	2793	HA	SER	205	10.939	57.819	12.619	1.00	0.00	H
ATOM	2794	HB2	SER	205	12.697	58.572	10.988	1.00	0.00	H
ATOM	2795	HB3	SER	205	11.537	57.450	10.322	1.00	0.00	H
ATOM	2796	HG	SER	205	10.566	59.100	9.224	1.00	0.00	H
ATOM	2797	N	MET	206	10.747	61.112	12.195	1.00	3.15	N
ATOM	2798	CA	MET	206	11.227	62.393	12.702	1.00	3.15	C
ATOM	2799	C	MET	206	10.173	63.163	13.517	1.00	3.15	C
ATOM	2800	O	MET	206	10.428	64.267	14.002	1.00	3.15	O
ATOM	2801	CB	MET	206	11.666	63.287	11.519	1.00	3.15	C
ATOM	2802	CG	MET	206	12.790	62.704	10.649	1.00	3.15	C
ATOM	2803	SD	MET	206	14.359	62.385	11.508	1.00	3.15	S
ATOM	2804	CE	MET	206	15.404	61.975	10.088	1.00	3.15	C
ATOM	2805	H	MET	206	10.267	61.121	11.301	1.00	0.00	H
ATOM	2806	HA	MET	206	12.088	62.260	13.360	1.00	0.00	H
ATOM	2807	HB2	MET	206	10.807	63.513	10.886	1.00	0.00	H
ATOM	2808	HB3	MET	206	12.008	64.253	11.900	1.00	0.00	H
ATOM	2809	HG2	MET	206	12.460	61.783	10.170	1.00	0.00	H
ATOM	2810	HG3	MET	206	12.994	63.410	9.843	1.00	0.00	H
ATOM	2811	HE1	MET	206	16.413	61.734	10.422	1.00	0.00	H
ATOM	2812	HE2	MET	206	14.999	61.115	9.553	1.00	0.00	H
ATOM	2813	HE3	MET	206	15.461	62.821	9.402	1.00	0.00	H
ATOM	2814	N	GLY	207	8.977	62.606	13.731	1.00	3.42	N
ATOM	2815	CA	GLY	207	7.842	63.390	14.218	1.00	3.42	C
ATOM	2816	C	GLY	207	7.825	63.629	15.715	1.00	3.42	C
ATOM	2817	O	GLY	207	6.996	64.389	16.206	1.00	3.42	O
ATOM	2818	H	GLY	207	8.784	61.705	13.313	1.00	0.00	H
ATOM	2819	HA2	GLY	207	7.810	64.354	13.708	1.00	0.00	H
ATOM	2820	HA3	GLY	207	6.897	62.909	13.987	1.00	0.00	H
ATOM	2821	N	PHE	208	8.783	63.039	16.421	1.00	3.59	N
ATOM	2822	CA	PHE	208	9.043	63.289	17.825	1.00	3.59	C
ATOM	2823	C	PHE	208	9.857	64.561	18.031	1.00	3.59	C
ATOM	2824	O	PHE	208	9.871	65.090	19.138	1.00	3.59	O
ATOM	2825	CB	PHE	208	9.892	62.113	18.360	1.00	3.59	C
ATOM	2826	CG	PHE	208	9.261	60.739	18.186	1.00	3.59	C
ATOM	2827	CD1	PHE	208	8.286	60.285	19.099	1.00	3.59	C
ATOM	2828	CD2	PHE	208	9.648	59.910	17.110	1.00	3.59	C
ATOM	2829	CE1	PHE	208	7.708	59.009	18.942	1.00	3.59	C
ATOM	2830	CE2	PHE	208	9.065	58.639	16.950	1.00	3.59	C
ATOM	2831	CZ	PHE	208	8.099	58.185	17.869	1.00	3.59	C
ATOM	2832	H	PHE	208	9.422	62.438	15.921	1.00	0.00	H
ATOM	2833	HA	PHE	208	8.107	63.362	18.383	1.00	0.00	H
ATOM	2834	HB2	PHE	208	10.876	62.108	17.884	1.00	0.00	H
ATOM	2835	HB3	PHE	208	10.089	62.261	19.423	1.00	0.00	H
ATOM	2836	HD1	PHE	208	7.986	60.913	19.926	1.00	0.00	H
ATOM	2837	HD2	PHE	208	10.398	60.238	16.401	1.00	0.00	H
ATOM	2838	HE1	PHE	208	6.973	58.662	19.653	1.00	0.00	H
ATOM	2839	HE2	PHE	208	9.371	58.013	16.124	1.00	0.00	H
ATOM	2840	HZ	PHE	208	7.669	57.202	17.752	1.00	0.00	H
ATOM	2841	N	ASN	209	10.547	65.027	16.985	1.00	3.26	N
ATOM	2842	CA	ASN	209	11.524	66.091	17.106	1.00	3.26	C
ATOM	2843	C	ASN	209	10.826	67.438	16.931	1.00	3.26	C
ATOM	2844	O	ASN	209	10.852	68.262	17.841	1.00	3.26	O
ATOM	2845	CB	ASN	209	12.594	66.010	15.973	1.00	3.26	C
ATOM	2846	CG	ASN	209	13.358	64.694	15.844	1.00	3.26	C
ATOM	2847	OD1	ASN	209	13.501	63.941	16.805	1.00	3.26	O
ATOM	2848	ND2	ASN	209	13.815	64.390	14.636	1.00	3.26	N
ATOM	2849	H	ASN	209	10.489	64.545	16.096	1.00	0.00	H
ATOM	2850	HA	ASN	209	12.023	66.050	18.076	1.00	0.00	H

ATOM	2851	HB2	ASN	209	12.145	66.194	14.996	1.00	0.00	H
ATOM	2852	HB3	ASN	209	13.333	66.801	16.105	1.00	0.00	H
ATOM	2853	HD22	ASN	209	14.300	63.528	14.458	1.00	0.00	H
ATOM	2854	HD21	ASN	209	13.695	65.075	13.855	1.00	0.00	H
ATOM	2855	N	SER	210	10.219	67.652	15.755	1.00	2.96	N
ATOM	2856	CA	SER	210	9.569	68.896	15.375	1.00	2.96	C
ATOM	2857	C	SER	210	8.031	68.792	15.505	1.00	2.96	C
ATOM	2858	O	SER	210	7.357	69.821	15.450	1.00	2.96	O
ATOM	2859	CB	SER	210	9.937	69.214	13.897	1.00	2.96	C
ATOM	2860	OG	SER	210	9.264	70.358	13.385	1.00	2.96	O
ATOM	2861	H	SER	210	10.366	66.960	15.031	1.00	0.00	H
ATOM	2862	HA	SER	210	9.904	69.721	16.009	1.00	0.00	H
ATOM	2863	HB2	SER	210	11.010	69.393	13.814	1.00	0.00	H
ATOM	2864	HB3	SER	210	9.716	68.364	13.249	1.00	0.00	H
ATOM	2865	HG	SER	210	8.378	70.352	13.727	1.00	0.00	H
ATOM	2866	N	GLY	211	7.434	67.591	15.563	1.00	2.52	N
ATOM	2867	CA	GLY	211	5.970	67.445	15.516	1.00	2.52	C
ATOM	2868	C	GLY	211	5.442	67.572	14.076	1.00	2.52	C
ATOM	2869	O	GLY	211	4.346	68.093	13.874	1.00	2.52	O
ATOM	2870	H	GLY	211	8.001	66.773	15.738	1.00	0.00	H
ATOM	2871	HA2	GLY	211	5.674	66.478	15.915	1.00	0.00	H
ATOM	2872	HA3	GLY	211	5.487	68.192	16.151	1.00	0.00	H
ATOM	2873	N	TYR	212	6.283	67.172	13.107	1.00	2.19	N
ATOM	2874	CA	TYR	212	6.478	67.659	11.733	1.00	2.19	C
ATOM	2875	C	TYR	212	5.439	68.598	11.080	1.00	2.19	C
ATOM	2876	O	TYR	212	4.231	68.331	11.055	1.00	2.19	O
ATOM	2877	CB	TYR	212	7.016	66.546	10.798	1.00	2.19	C
ATOM	2878	CG	TYR	212	5.987	65.701	10.052	1.00	2.19	C
ATOM	2879	CD1	TYR	212	4.821	65.233	10.696	1.00	2.19	C
ATOM	2880	CD2	TYR	212	6.187	65.396	8.687	1.00	2.19	C
ATOM	2881	CE1	TYR	212	3.852	64.509	9.976	1.00	2.19	C
ATOM	2882	CE2	TYR	212	5.224	64.659	7.970	1.00	2.19	C
ATOM	2883	CZ	TYR	212	4.052	64.220	8.613	1.00	2.19	C
ATOM	2884	OH	TYR	212	3.118	63.508	7.921	1.00	2.19	O
ATOM	2885	H	TYR	212	7.092	66.702	13.480	1.00	0.00	H
ATOM	2886	HA	TYR	212	7.347	68.302	11.890	1.00	0.00	H
ATOM	2887	HB2	TYR	212	7.649	67.025	10.049	1.00	0.00	H
ATOM	2888	HB3	TYR	212	7.682	65.876	11.343	1.00	0.00	H
ATOM	2889	HD1	TYR	212	4.644	65.448	11.740	1.00	0.00	H
ATOM	2890	HD2	TYR	212	7.078	65.729	8.176	1.00	0.00	H
ATOM	2891	HE1	TYR	212	2.959	64.177	10.486	1.00	0.00	H
ATOM	2892	HE2	TYR	212	5.385	64.425	6.928	1.00	0.00	H
ATOM	2893	HH	TYR	212	2.520	63.066	8.502	1.00	0.00	H
ATOM	2894	N	ALA	213	5.938	69.689	10.487	1.00	1.83	N
ATOM	2895	CA	ALA	213	5.192	70.548	9.586	1.00	1.83	C
ATOM	2896	C	ALA	213	5.372	70.019	8.157	1.00	1.83	C
ATOM	2897	O	ALA	213	4.397	69.629	7.526	1.00	1.83	O
ATOM	2898	CB	ALA	213	5.709	72.000	9.658	1.00	1.83	C
ATOM	2899	H	ALA	213	6.952	69.800	10.473	1.00	0.00	H
ATOM	2900	HA	ALA	213	4.140	70.553	9.852	1.00	0.00	H
ATOM	2901	HB1	ALA	213	5.237	72.624	8.898	1.00	0.00	H
ATOM	2902	HB2	ALA	213	5.465	72.452	10.617	1.00	0.00	H
ATOM	2903	HB3	ALA	213	6.788	72.079	9.525	1.00	0.00	H
ATOM	2904	N	VAL	214	6.599	70.101	7.617	1.00	1.72	N
ATOM	2905	CA	VAL	214	7.038	69.761	6.247	1.00	1.72	C
ATOM	2906	C	VAL	214	6.339	70.617	5.155	1.00	1.72	C
ATOM	2907	O	VAL	214	6.502	70.412	3.952	1.00	1.72	O
ATOM	2908	CB	VAL	214	7.065	68.212	6.031	1.00	1.72	C
ATOM	2909	CG1	VAL	214	5.783	67.570	5.458	1.00	1.72	C
ATOM	2910	CG2	VAL	214	8.300	67.783	5.218	1.00	1.72	C
ATOM	2911	H	VAL	214	7.332	70.352	8.275	1.00	0.00	H
ATOM	2912	HA	VAL	214	8.069	70.105	6.195	1.00	0.00	H
ATOM	2913	HB	VAL	214	7.210	67.757	7.011	1.00	0.00	H

ATOM	2914	HG11	VAL	214	5.916	66.497	5.320	1.00	0.00	H
ATOM	2915	HG12	VAL	214	4.933	67.686	6.127	1.00	0.00	H
ATOM	2916	HG13	VAL	214	5.504	67.988	4.492	1.00	0.00	H
ATOM	2917	HG21	VAL	214	8.327	66.703	5.061	1.00	0.00	H
ATOM	2918	HG22	VAL	214	8.324	68.262	4.241	1.00	0.00	H
ATOM	2919	HG23	VAL	214	9.224	68.052	5.729	1.00	0.00	H
ATOM	2920	N	ASN	215	5.552	71.599	5.599	1.00	1.47	N
ATOM	2921	CA	ASN	215	4.590	72.396	4.863	1.00	1.47	C
ATOM	2922	C	ASN	215	4.337	73.640	5.730	1.00	1.47	C
ATOM	2923	O	ASN	215	3.649	73.503	6.746	1.00	1.47	O
ATOM	2924	CB	ASN	215	3.316	71.542	4.637	1.00	1.47	C
ATOM	2925	CG	ASN	215	2.230	72.230	3.802	1.00	1.47	C
ATOM	2926	OD1	ASN	215	2.046	73.444	3.861	1.00	1.47	O
ATOM	2927	ND2	ASN	215	1.522	71.462	2.986	1.00	1.47	N
ATOM	2928	H	ASN	215	5.484	71.663	6.604	1.00	0.00	H
ATOM	2929	HA	ASN	215	5.012	72.663	3.900	1.00	0.00	H
ATOM	2930	HB2	ASN	215	3.600	70.620	4.129	1.00	0.00	H
ATOM	2931	HB3	ASN	215	2.893	71.227	5.590	1.00	0.00	H
ATOM	2932	HD22	ASN	215	0.857	71.885	2.329	1.00	0.00	H
ATOM	2933	HD21	ASN	215	1.666	70.465	2.951	1.00	0.00	H
ATOM	2934	N	PRO	216	4.869	74.833	5.372	1.00	1.47	N
ATOM	2935	CA	PRO	216	4.629	76.095	6.088	1.00	1.47	C
ATOM	2936	C	PRO	216	3.152	76.458	6.302	1.00	1.47	C
ATOM	2937	O	PRO	216	2.812	76.996	7.355	1.00	1.47	O
ATOM	2938	CB	PRO	216	5.307	77.181	5.236	1.00	1.47	C
ATOM	2939	CG	PRO	216	6.385	76.450	4.459	1.00	1.47	C
ATOM	2940	CD	PRO	216	5.852	75.026	4.304	1.00	1.47	C
ATOM	2941	HA	PRO	216	5.108	76.021	7.063	1.00	0.00	H
ATOM	2942	HB2	PRO	216	4.607	77.628	4.531	1.00	0.00	H
ATOM	2943	HB3	PRO	216	5.707	77.992	5.844	1.00	0.00	H
ATOM	2944	HG2	PRO	216	6.616	76.920	3.504	1.00	0.00	H
ATOM	2945	HG3	PRO	216	7.301	76.436	5.052	1.00	0.00	H
ATOM	2946	HD3	PRO	216	6.655	74.289	4.337	1.00	0.00	H
ATOM	2947	HD2	PRO	216	5.345	74.934	3.344	1.00	0.00	H
ATOM	2948	N	ALA	217	2.267	76.165	5.334	1.00	1.55	N
ATOM	2949	CA	ALA	217	0.852	76.522	5.401	1.00	1.55	C
ATOM	2950	C	ALA	217	0.125	75.739	6.500	1.00	1.55	C
ATOM	2951	O	ALA	217	-0.616	76.344	7.269	1.00	1.55	O
ATOM	2952	CB	ALA	217	0.172	76.274	4.046	1.00	1.55	C
ATOM	2953	H	ALA	217	2.575	75.614	4.544	1.00	0.00	H
ATOM	2954	HA	ALA	217	0.775	77.582	5.645	1.00	0.00	H
ATOM	2955	HB1	ALA	217	-0.894	76.490	4.108	1.00	0.00	H
ATOM	2956	HB2	ALA	217	0.588	76.918	3.272	1.00	0.00	H
ATOM	2957	HB3	ALA	217	0.265	75.240	3.714	1.00	0.00	H
ATOM	2958	N	ARG	218	0.405	74.432	6.618	1.00	1.74	N
ATOM	2959	CA	ARG	218	-0.091	73.517	7.656	1.00	1.74	C
ATOM	2960	C	ARG	218	0.400	73.853	9.083	1.00	1.74	C
ATOM	2961	O	ARG	218	-0.051	73.209	10.028	1.00	1.74	O
ATOM	2962	CB	ARG	218	0.234	72.054	7.218	1.00	1.74	C
ATOM	2963	CG	ARG	218	0.233	70.912	8.274	1.00	1.74	C
ATOM	2964	CD	ARG	218	1.608	70.788	8.961	1.00	1.74	C
ATOM	2965	NE	ARG	218	1.689	69.858	10.101	1.00	1.74	N
ATOM	2966	CZ	ARG	218	1.391	70.118	11.382	1.00	1.74	C
ATOM	2967	NH1	ARG	218	0.663	71.186	11.716	1.00	1.74	N
ATOM	2968	NH2	ARG	218	1.852	69.299	12.326	1.00	1.74	N1+
ATOM	2969	H	ARG	218	1.075	74.061	5.957	1.00	0.00	H
ATOM	2970	HA	ARG	218	-1.176	73.620	7.698	1.00	0.00	H
ATOM	2971	HB2	ARG	218	-0.483	71.787	6.440	1.00	0.00	H
ATOM	2972	HB3	ARG	218	1.202	72.045	6.723	1.00	0.00	H
ATOM	2973	HG2	ARG	218	-0.586	71.013	8.986	1.00	0.00	H
ATOM	2974	HG3	ARG	218	0.040	69.972	7.756	1.00	0.00	H
ATOM	2975	HD2	ARG	218	2.296	70.441	8.200	1.00	0.00	H
ATOM	2976	HD3	ARG	218	2.010	71.744	9.290	1.00	0.00	H

ATOM	2977	HE	ARG	218	2.221	69.010	9.908	1.00	0.00	H
ATOM	2978	HH12	ARG	218	0.326	71.380	12.669	1.00	0.00	H
ATOM	2979	HH11	ARG	218	0.372	71.889	11.029	1.00	0.00	H
ATOM	2980	HH22	ARG	218	1.680	69.445	13.311	1.00	0.00	H
ATOM	2981	HH21	ARG	218	2.628	68.659	12.116	1.00	0.00	H
ATOM	2982	N	ASP	219	1.299	74.825	9.270	1.00	1.84	N
ATOM	2983	CA	ASP	219	1.691	75.312	10.595	1.00	1.84	C
ATOM	2984	C	ASP	219	1.281	76.776	10.754	1.00	1.84	C
ATOM	2985	O	ASP	219	0.516	77.098	11.657	1.00	1.84	O
ATOM	2986	CB	ASP	219	3.190	75.076	10.863	1.00	1.84	C
ATOM	2987	CG	ASP	219	3.521	75.254	12.341	1.00	1.84	C
ATOM	2988	OD1	ASP	219	3.454	74.254	13.087	1.00	1.84	O
ATOM	2989	OD2	ASP	219	3.859	76.374	12.771	1.00	1.84	O1-
ATOM	2990	H	ASP	219	1.642	75.326	8.461	1.00	0.00	H
ATOM	2991	HA	ASP	219	1.149	74.771	11.374	1.00	0.00	H
ATOM	2992	HB2	ASP	219	3.465	74.060	10.581	1.00	0.00	H
ATOM	2993	HB3	ASP	219	3.814	75.745	10.269	1.00	0.00	H
ATOM	2994	N	PHE	220	1.706	77.655	9.837	1.00	1.98	N
ATOM	2995	CA	PHE	220	1.534	79.106	9.937	1.00	1.98	C
ATOM	2996	C	PHE	220	0.069	79.556	9.867	1.00	1.98	C
ATOM	2997	O	PHE	220	-0.329	80.453	10.608	1.00	1.98	O
ATOM	2998	CB	PHE	220	2.335	79.792	8.810	1.00	1.98	C
ATOM	2999	CG	PHE	220	2.432	81.308	8.913	1.00	1.98	C
ATOM	3000	CD1	PHE	220	3.164	81.900	9.965	1.00	1.98	C
ATOM	3001	CD2	PHE	220	1.780	82.130	7.969	1.00	1.98	C
ATOM	3002	CE1	PHE	220	3.253	83.301	10.066	1.00	1.98	C
ATOM	3003	CE2	PHE	220	1.864	83.532	8.076	1.00	1.98	C
ATOM	3004	CZ	PHE	220	2.602	84.118	9.123	1.00	1.98	C
ATOM	3005	H	PHE	220	2.283	77.314	9.072	1.00	0.00	H
ATOM	3006	HA	PHE	220	1.941	79.418	10.900	1.00	0.00	H
ATOM	3007	HB2	PHE	220	3.349	79.401	8.801	1.00	0.00	H
ATOM	3008	HB3	PHE	220	1.915	79.526	7.839	1.00	0.00	H
ATOM	3009	HD1	PHE	220	3.662	81.284	10.700	1.00	0.00	H
ATOM	3010	HD2	PHE	220	1.207	81.693	7.165	1.00	0.00	H
ATOM	3011	HE1	PHE	220	3.822	83.748	10.869	1.00	0.00	H
ATOM	3012	HE2	PHE	220	1.359	84.160	7.356	1.00	0.00	H
ATOM	3013	HZ	PHE	220	2.667	85.193	9.203	1.00	0.00	H
ATOM	3014	N	GLY	221	-0.751	78.896	9.037	1.00	1.93	N
ATOM	3015	CA	GLY	221	-2.183	79.166	8.949	1.00	1.93	C
ATOM	3016	C	GLY	221	-2.877	78.732	10.253	1.00	1.93	C
ATOM	3017	O	GLY	221	-3.427	79.592	10.939	1.00	1.93	O
ATOM	3018	H	GLY	221	-0.388	78.093	8.538	1.00	0.00	H
ATOM	3019	HA2	GLY	221	-2.357	80.230	8.776	1.00	0.00	H
ATOM	3020	HA3	GLY	221	-2.614	78.632	8.102	1.00	0.00	H
ATOM	3021	N	PRO	222	-2.821	77.439	10.643	1.00	1.81	N
ATOM	3022	CA	PRO	222	-3.249	76.944	11.954	1.00	1.81	C
ATOM	3023	C	PRO	222	-2.760	77.714	13.182	1.00	1.81	C
ATOM	3024	O	PRO	222	-3.574	78.000	14.056	1.00	1.81	O
ATOM	3025	CB	PRO	222	-2.856	75.467	11.994	1.00	1.81	C
ATOM	3026	CG	PRO	222	-2.868	75.057	10.538	1.00	1.81	C
ATOM	3027	CD	PRO	222	-2.445	76.318	9.789	1.00	1.81	C
ATOM	3028	HA	PRO	222	-4.339	77.008	11.951	1.00	0.00	H
ATOM	3029	HB2	PRO	222	-1.859	75.326	12.418	1.00	0.00	H
ATOM	3030	HB3	PRO	222	-3.571	74.883	12.570	1.00	0.00	H
ATOM	3031	HG2	PRO	222	-2.258	74.184	10.330	1.00	0.00	H
ATOM	3032	HG3	PRO	222	-3.888	74.797	10.250	1.00	0.00	H
ATOM	3033	HD3	PRO	222	-2.910	76.384	8.811	1.00	0.00	H
ATOM	3034	HD2	PRO	222	-1.364	76.318	9.668	1.00	0.00	H
ATOM	3035	N	ARG	223	-1.481	78.116	13.223	1.00	1.60	N
ATOM	3036	CA	ARG	223	-0.927	78.943	14.290	1.00	1.60	C
ATOM	3037	C	ARG	223	-1.630	80.286	14.455	1.00	1.60	C
ATOM	3038	O	ARG	223	-1.869	80.736	15.575	1.00	1.60	O
ATOM	3039	CB	ARG	223	0.586	79.208	14.103	1.00	1.60	C

ATOM	3040	CG	ARG	223	1.477	78.106	14.691	1.00	1.60	C
ATOM	3041	CD	ARG	223	2.915	78.572	14.981	1.00	1.60	C
ATOM	3042	NE	ARG	223	3.387	77.964	16.230	1.00	1.60	N
ATOM	3043	CZ	ARG	223	4.048	76.815	16.364	1.00	1.60	C
ATOM	3044	NH1	ARG	223	4.465	76.122	15.306	1.00	1.60	N
ATOM	3045	NH2	ARG	223	4.261	76.356	17.595	1.00	1.60	N1+
ATOM	3046	H	ARG	223	-0.832	77.782	12.510	1.00	0.00	H
ATOM	3047	HA	ARG	223	-1.066	78.395	15.218	1.00	0.00	H
ATOM	3048	HB2	ARG	223	0.830	79.389	13.057	1.00	0.00	H
ATOM	3049	HB3	ARG	223	0.840	80.133	14.622	1.00	0.00	H
ATOM	3050	HG2	ARG	223	1.027	77.761	15.622	1.00	0.00	H
ATOM	3051	HG3	ARG	223	1.494	77.237	14.033	1.00	0.00	H
ATOM	3052	HD2	ARG	223	3.590	78.386	14.147	1.00	0.00	H
ATOM	3053	HD3	ARG	223	2.942	79.648	15.143	1.00	0.00	H
ATOM	3054	HE	ARG	223	3.073	78.418	17.098	1.00	0.00	H
ATOM	3055	HH12	ARG	223	4.954	75.233	15.378	1.00	0.00	H
ATOM	3056	HH11	ARG	223	4.200	76.347	14.313	1.00	0.00	H
ATOM	3057	HH22	ARG	223	4.607	75.389	17.716	1.00	0.00	H
ATOM	3058	HH21	ARG	223	3.883	76.822	18.416	1.00	0.00	H
ATOM	3059	N	LEU	224	-1.974	80.913	13.331	1.00	1.68	N
ATOM	3060	CA	LEU	224	-2.685	82.175	13.317	1.00	1.68	C
ATOM	3061	C	LEU	224	-4.127	81.992	13.808	1.00	1.68	C
ATOM	3062	O	LEU	224	-4.651	82.862	14.500	1.00	1.68	O
ATOM	3063	CB	LEU	224	-2.640	82.742	11.874	1.00	1.68	C
ATOM	3064	CG	LEU	224	-2.811	84.275	11.739	1.00	1.68	C
ATOM	3065	CD1	LEU	224	-2.029	84.802	10.523	1.00	1.68	C
ATOM	3066	CD2	LEU	224	-4.280	84.714	11.632	1.00	1.68	C
ATOM	3067	H	LEU	224	-1.743	80.485	12.442	1.00	0.00	H
ATOM	3068	HA	LEU	224	-2.170	82.862	13.991	1.00	0.00	H
ATOM	3069	HB2	LEU	224	-1.658	82.497	11.468	1.00	0.00	H
ATOM	3070	HB3	LEU	224	-3.347	82.224	11.225	1.00	0.00	H
ATOM	3071	HG	LEU	224	-2.380	84.750	12.621	1.00	0.00	H
ATOM	3072	HD11	LEU	224	-2.118	85.885	10.435	1.00	0.00	H
ATOM	3073	HD12	LEU	224	-0.965	84.571	10.604	1.00	0.00	H
ATOM	3074	HD13	LEU	224	-2.393	84.360	9.595	1.00	0.00	H
ATOM	3075	HD21	LEU	224	-4.360	85.796	11.523	1.00	0.00	H
ATOM	3076	HD22	LEU	224	-4.772	84.260	10.771	1.00	0.00	H
ATOM	3077	HD23	LEU	224	-4.850	84.449	12.520	1.00	0.00	H
ATOM	3078	N	PHE	225	-4.742	80.832	13.536	1.00	1.86	N
ATOM	3079	CA	PHE	225	-6.042	80.494	14.099	1.00	1.86	C
ATOM	3080	C	PHE	225	-5.957	80.255	15.605	1.00	1.86	C
ATOM	3081	O	PHE	225	-6.758	80.819	16.344	1.00	1.86	O
ATOM	3082	CB	PHE	225	-6.683	79.276	13.391	1.00	1.86	C
ATOM	3083	CG	PHE	225	-8.137	79.107	13.804	1.00	1.86	C
ATOM	3084	CD1	PHE	225	-9.139	79.889	13.191	1.00	1.86	C
ATOM	3085	CD2	PHE	225	-8.467	78.286	14.903	1.00	1.86	C
ATOM	3086	CE1	PHE	225	-10.446	79.900	13.715	1.00	1.86	C
ATOM	3087	CE2	PHE	225	-9.767	78.313	15.437	1.00	1.86	C
ATOM	3088	CZ	PHE	225	-10.756	79.128	14.852	1.00	1.86	C
ATOM	3089	H	PHE	225	-4.236	80.134	13.007	1.00	0.00	H
ATOM	3090	HA	PHE	225	-6.702	81.350	13.942	1.00	0.00	H
ATOM	3091	HB2	PHE	225	-6.648	79.407	12.309	1.00	0.00	H
ATOM	3092	HB3	PHE	225	-6.136	78.358	13.606	1.00	0.00	H
ATOM	3093	HD1	PHE	225	-8.896	80.524	12.351	1.00	0.00	H
ATOM	3094	HD2	PHE	225	-7.710	77.690	15.394	1.00	0.00	H
ATOM	3095	HE1	PHE	225	-11.205	80.524	13.266	1.00	0.00	H
ATOM	3096	HE2	PHE	225	-9.995	77.726	16.313	1.00	0.00	H
ATOM	3097	HZ	PHE	225	-11.750	79.154	15.275	1.00	0.00	H
ATOM	3098	N	THR	226	-4.978	79.479	16.084	1.00	2.29	N
ATOM	3099	CA	THR	226	-4.826	79.236	17.513	1.00	2.29	C
ATOM	3100	C	THR	226	-4.469	80.499	18.309	1.00	2.29	C
ATOM	3101	O	THR	226	-4.833	80.615	19.480	1.00	2.29	O
ATOM	3102	CB	THR	226	-3.804	78.104	17.768	1.00	2.29	C

ATOM	3103	OG1	THR	226	-2.559	78.347	17.144	1.00	2.29	O
ATOM	3104	CG2	THR	226	-4.294	76.735	17.297	1.00	2.29	C
ATOM	3105	H	THR	226	-4.332	79.007	15.454	1.00	0.00	H
ATOM	3106	HA	THR	226	-5.789	78.895	17.895	1.00	0.00	H
ATOM	3107	HB	THR	226	-3.630	78.029	18.844	1.00	0.00	H
ATOM	3108	HG1	THR	226	-2.268	79.229	17.341	1.00	0.00	H
ATOM	3109	HG21	THR	226	-4.445	76.700	16.219	1.00	0.00	H
ATOM	3110	HG22	THR	226	-3.567	75.967	17.561	1.00	0.00	H
ATOM	3111	HG23	THR	226	-5.239	76.475	17.775	1.00	0.00	H
ATOM	3112	N	ALA	227	-3.804	81.462	17.658	1.00	2.69	N
ATOM	3113	CA	ALA	227	-3.566	82.784	18.205	1.00	2.69	C
ATOM	3114	C	ALA	227	-4.874	83.526	18.451	1.00	2.69	C
ATOM	3115	O	ALA	227	-5.117	84.021	19.552	1.00	2.69	O
ATOM	3116	CB	ALA	227	-2.659	83.598	17.266	1.00	2.69	C
ATOM	3117	H	ALA	227	-3.498	81.279	16.709	1.00	0.00	H
ATOM	3118	HA	ALA	227	-3.053	82.663	19.160	1.00	0.00	H
ATOM	3119	HB1	ALA	227	-2.421	84.566	17.709	1.00	0.00	H
ATOM	3120	HB2	ALA	227	-1.713	83.090	17.088	1.00	0.00	H
ATOM	3121	HB3	ALA	227	-3.122	83.788	16.300	1.00	0.00	H
ATOM	3122	N	LEU	228	-5.741	83.519	17.436	1.00	3.07	N
ATOM	3123	CA	LEU	228	-7.055	84.124	17.496	1.00	3.07	C
ATOM	3124	C	LEU	228	-8.056	83.365	18.390	1.00	3.07	C
ATOM	3125	O	LEU	228	-9.013	83.968	18.869	1.00	3.07	O
ATOM	3126	CB	LEU	228	-7.649	84.240	16.074	1.00	3.07	C
ATOM	3127	CG	LEU	228	-6.947	85.261	15.149	1.00	3.07	C
ATOM	3128	CD1	LEU	228	-7.464	85.112	13.712	1.00	3.07	C
ATOM	3129	CD2	LEU	228	-7.123	86.713	15.624	1.00	3.07	C
ATOM	3130	H	LEU	228	-5.473	83.059	16.574	1.00	0.00	H
ATOM	3131	HA	LEU	228	-6.961	85.119	17.933	1.00	0.00	H
ATOM	3132	HB2	LEU	228	-7.636	83.253	15.607	1.00	0.00	H
ATOM	3133	HB3	LEU	228	-8.705	84.511	16.141	1.00	0.00	H
ATOM	3134	HG	LEU	228	-5.879	85.044	15.136	1.00	0.00	H
ATOM	3135	HD11	LEU	228	-6.939	85.780	13.029	1.00	0.00	H
ATOM	3136	HD12	LEU	228	-7.316	84.094	13.357	1.00	0.00	H
ATOM	3137	HD13	LEU	228	-8.530	85.335	13.646	1.00	0.00	H
ATOM	3138	HD21	LEU	228	-6.668	87.413	14.922	1.00	0.00	H
ATOM	3139	HD22	LEU	228	-8.177	86.978	15.718	1.00	0.00	H
ATOM	3140	HD23	LEU	228	-6.650	86.885	16.591	1.00	0.00	H
ATOM	3141	N	ALA	229	-7.833	82.070	18.646	1.00	3.04	N
ATOM	3142	CA	ALA	229	-8.688	81.211	19.467	1.00	3.04	C
ATOM	3143	C	ALA	229	-8.537	81.462	20.982	1.00	3.04	C
ATOM	3144	O	ALA	229	-9.254	80.847	21.766	1.00	3.04	O
ATOM	3145	CB	ALA	229	-8.323	79.745	19.187	1.00	3.04	C
ATOM	3146	H	ALA	229	-7.100	81.626	18.106	1.00	0.00	H
ATOM	3147	HA	ALA	229	-9.733	81.380	19.197	1.00	0.00	H
ATOM	3148	HB1	ALA	229	-8.997	79.072	19.716	1.00	0.00	H
ATOM	3149	HB2	ALA	229	-8.403	79.509	18.125	1.00	0.00	H
ATOM	3150	HB3	ALA	229	-7.312	79.510	19.519	1.00	0.00	H
ATOM	3151	N	GLY	230	-7.621	82.348	21.395	1.00	2.87	N
ATOM	3152	CA	GLY	230	-7.493	82.833	22.768	1.00	2.87	C
ATOM	3153	C	GLY	230	-6.127	82.559	23.398	1.00	2.87	C
ATOM	3154	O	GLY	230	-5.794	83.181	24.404	1.00	2.87	O
ATOM	3155	H	GLY	230	-7.094	82.824	20.675	1.00	0.00	H
ATOM	3156	HA2	GLY	230	-7.659	83.911	22.763	1.00	0.00	H
ATOM	3157	HA3	GLY	230	-8.256	82.413	23.426	1.00	0.00	H
ATOM	3158	N	TRP	231	-5.311	81.672	22.809	1.00	2.50	N
ATOM	3159	CA	TRP	231	-3.972	81.349	23.315	1.00	2.50	C
ATOM	3160	C	TRP	231	-2.975	82.480	22.981	1.00	2.50	C
ATOM	3161	O	TRP	231	-1.989	82.683	23.691	1.00	2.50	O
ATOM	3162	CB	TRP	231	-3.485	80.008	22.723	1.00	2.50	C
ATOM	3163	CG	TRP	231	-4.049	78.759	23.345	1.00	2.50	C
ATOM	3164	CD1	TRP	231	-3.335	77.863	24.066	1.00	2.50	C
ATOM	3165	CD2	TRP	231	-5.420	78.246	23.327	1.00	2.50	C

ATOM	3166	NE1	TRP	231	-4.165	76.856	24.516	1.00	2.50	N
ATOM	3167	CE2	TRP	231	-5.474	77.072	24.140	1.00	2.50	C
ATOM	3168	CE3	TRP	231	-6.635	78.654	22.724	1.00	2.50	C
ATOM	3169	CZ2	TRP	231	-6.677	76.391	24.401	1.00	2.50	C
ATOM	3170	CZ3	TRP	231	-7.843	77.969	22.962	1.00	2.50	C
ATOM	3171	CH2	TRP	231	-7.869	76.846	23.810	1.00	2.50	C
ATOM	3172	H	TRP	231	-5.640	81.202	21.978	1.00	0.00	H
ATOM	3173	HA	TRP	231	-4.006	81.256	24.401	1.00	0.00	H
ATOM	3174	HB2	TRP	231	-3.670	79.969	21.652	1.00	0.00	H
ATOM	3175	HB3	TRP	231	-2.403	79.945	22.848	1.00	0.00	H
ATOM	3176	HD1	TRP	231	-2.275	77.951	24.256	1.00	0.00	H
ATOM	3177	HE1	TRP	231	-3.850	76.046	25.058	1.00	0.00	H
ATOM	3178	HE3	TRP	231	-6.648	79.518	22.082	1.00	0.00	H
ATOM	3179	HZ2	TRP	231	-6.698	75.540	25.063	1.00	0.00	H
ATOM	3180	HZ3	TRP	231	-8.759	78.326	22.513	1.00	0.00	H
ATOM	3181	HH2	TRP	231	-8.803	76.342	24.014	1.00	0.00	H
ATOM	3182	N	GLY	232	-3.241	83.268	21.931	1.00	2.22	N
ATOM	3183	CA	GLY	232	-2.670	84.597	21.772	1.00	2.22	C
ATOM	3184	C	GLY	232	-1.248	84.529	21.223	1.00	2.22	C
ATOM	3185	O	GLY	232	-1.015	84.035	20.123	1.00	2.22	O
ATOM	3186	H	GLY	232	-4.051	83.047	21.366	1.00	0.00	H
ATOM	3187	HA2	GLY	232	-3.283	85.160	21.069	1.00	0.00	H
ATOM	3188	HA3	GLY	232	-2.708	85.140	22.718	1.00	0.00	H
ATOM	3189	N	SER	233	-0.293	85.012	22.023	1.00	2.04	N
ATOM	3190	CA	SER	233	1.135	84.996	21.719	1.00	2.04	C
ATOM	3191	C	SER	233	1.761	83.630	22.097	1.00	2.04	C
ATOM	3192	O	SER	233	2.819	83.260	21.587	1.00	2.04	O
ATOM	3193	CB	SER	233	1.790	86.125	22.553	1.00	2.04	C
ATOM	3194	OG	SER	233	3.107	86.427	22.134	1.00	2.04	O
ATOM	3195	H	SER	233	-0.574	85.340	22.934	1.00	0.00	H
ATOM	3196	HA	SER	233	1.282	85.197	20.658	1.00	0.00	H
ATOM	3197	HB2	SER	233	1.206	87.043	22.465	1.00	0.00	H
ATOM	3198	HB3	SER	233	1.807	85.863	23.613	1.00	0.00	H
ATOM	3199	HG	SER	233	3.482	87.074	22.713	1.00	0.00	H
ATOM	3200	N	ALA	234	1.095	82.846	22.964	1.00	2.01	N
ATOM	3201	CA	ALA	234	1.630	81.622	23.568	1.00	2.01	C
ATOM	3202	C	ALA	234	1.785	80.467	22.564	1.00	2.01	C
ATOM	3203	O	ALA	234	2.497	79.507	22.833	1.00	2.01	O
ATOM	3204	CB	ALA	234	0.677	81.191	24.693	1.00	2.01	C
ATOM	3205	H	ALA	234	0.163	83.118	23.252	1.00	0.00	H
ATOM	3206	HA	ALA	234	2.614	81.839	23.990	1.00	0.00	H
ATOM	3207	HB1	ALA	234	-0.263	80.795	24.306	1.00	0.00	H
ATOM	3208	HB2	ALA	234	1.124	80.409	25.302	1.00	0.00	H
ATOM	3209	HB3	ALA	234	0.442	82.017	25.364	1.00	0.00	H
ATOM	3210	N	VAL	235	1.165	80.574	21.383	1.00	2.06	N
ATOM	3211	CA	VAL	235	1.226	79.566	20.328	1.00	2.06	C
ATOM	3212	C	VAL	235	2.549	79.632	19.533	1.00	2.06	C
ATOM	3213	O	VAL	235	2.831	78.738	18.733	1.00	2.06	O
ATOM	3214	CB	VAL	235	0.011	79.765	19.384	1.00	2.06	C
ATOM	3215	CG1	VAL	235	-1.304	79.743	20.175	1.00	2.06	C
ATOM	3216	CG2	VAL	235	0.062	81.035	18.516	1.00	2.06	C
ATOM	3217	H	VAL	235	0.614	81.401	21.215	1.00	0.00	H
ATOM	3218	HA	VAL	235	1.147	78.581	20.782	1.00	0.00	H
ATOM	3219	HB	VAL	235	-0.010	78.910	18.705	1.00	0.00	H
ATOM	3220	HG11	VAL	235	-2.156	79.697	19.509	1.00	0.00	H
ATOM	3221	HG12	VAL	235	-1.362	78.878	20.837	1.00	0.00	H
ATOM	3222	HG13	VAL	235	-1.423	80.642	20.780	1.00	0.00	H
ATOM	3223	HG21	VAL	235	-0.855	81.147	17.942	1.00	0.00	H
ATOM	3224	HG22	VAL	235	0.181	81.935	19.119	1.00	0.00	H
ATOM	3225	HG23	VAL	235	0.879	81.009	17.796	1.00	0.00	H
ATOM	3226	N	PHE	236	3.341	80.699	19.702	1.00	2.21	N
ATOM	3227	CA	PHE	236	4.536	80.980	18.908	1.00	2.21	C
ATOM	3228	C	PHE	236	5.833	80.684	19.685	1.00	2.21	C

ATOM	3229	O	PHE	236	6.901	80.578	19.075	1.00	2.21	O
ATOM	3230	CB	PHE	236	4.509	82.476	18.525	1.00	2.21	C
ATOM	3231	CG	PHE	236	3.322	82.887	17.662	1.00	2.21	C
ATOM	3232	CD1	PHE	236	3.163	82.374	16.358	1.00	2.21	C
ATOM	3233	CD2	PHE	236	2.365	83.789	18.168	1.00	2.21	C
ATOM	3234	CE1	PHE	236	2.053	82.746	15.575	1.00	2.21	C
ATOM	3235	CE2	PHE	236	1.248	84.155	17.393	1.00	2.21	C
ATOM	3236	CZ	PHE	236	1.090	83.631	16.095	1.00	2.21	C
ATOM	3237	H	PHE	236	3.033	81.410	20.355	1.00	0.00	H
ATOM	3238	HA	PHE	236	4.552	80.381	17.998	1.00	0.00	H
ATOM	3239	HB2	PHE	236	4.533	83.093	19.425	1.00	0.00	H
ATOM	3240	HB3	PHE	236	5.413	82.741	17.980	1.00	0.00	H
ATOM	3241	HD1	PHE	236	3.897	81.700	15.948	1.00	0.00	H
ATOM	3242	HD2	PHE	236	2.491	84.201	19.156	1.00	0.00	H
ATOM	3243	HE1	PHE	236	1.942	82.358	14.573	1.00	0.00	H
ATOM	3244	HE2	PHE	236	0.510	84.833	17.798	1.00	0.00	H
ATOM	3245	HZ	PHE	236	0.233	83.912	15.500	1.00	0.00	H
ATOM	3246	N	THR	237	5.771	80.546	21.016	1.00	2.35	N
ATOM	3247	CA	THR	237	6.931	80.488	21.896	1.00	2.35	C
ATOM	3248	C	THR	237	6.668	79.579	23.103	1.00	2.35	C
ATOM	3249	O	THR	237	5.556	79.537	23.621	1.00	2.35	O
ATOM	3250	CB	THR	237	7.424	81.929	22.216	1.00	2.35	C
ATOM	3251	OG1	THR	237	8.578	81.917	23.037	1.00	2.35	O
ATOM	3252	CG2	THR	237	6.382	82.806	22.929	1.00	2.35	C
ATOM	3253	H	THR	237	4.868	80.514	21.475	1.00	0.00	H
ATOM	3254	HA	THR	237	7.722	79.988	21.337	1.00	0.00	H
ATOM	3255	HB	THR	237	7.697	82.422	21.282	1.00	0.00	H
ATOM	3256	HG1	THR	237	9.261	81.405	22.607	1.00	0.00	H
ATOM	3257	HG21	THR	237	6.776	83.804	23.119	1.00	0.00	H
ATOM	3258	HG22	THR	237	5.480	82.924	22.327	1.00	0.00	H
ATOM	3259	HG23	THR	237	6.085	82.379	23.888	1.00	0.00	H
ATOM	3260	N	THR	238	7.713	78.884	23.566	1.00	2.48	N
ATOM	3261	CA	THR	238	7.714	78.027	24.747	1.00	2.48	C
ATOM	3262	C	THR	238	9.045	78.186	25.509	1.00	2.48	C
ATOM	3263	O	THR	238	9.567	77.219	26.062	1.00	2.48	O
ATOM	3264	CB	THR	238	7.247	76.585	24.385	1.00	2.48	C
ATOM	3265	OG1	THR	238	7.131	75.764	25.536	1.00	2.48	O
ATOM	3266	CG2	THR	238	8.175	75.854	23.399	1.00	2.48	C
ATOM	3267	H	THR	238	8.602	79.000	23.091	1.00	0.00	H
ATOM	3268	HA	THR	238	6.973	78.428	25.440	1.00	0.00	H
ATOM	3269	HB	THR	238	6.255	76.642	23.940	1.00	0.00	H
ATOM	3270	HG1	THR	238	7.976	75.805	25.980	1.00	0.00	H
ATOM	3271	HG21	THR	238	7.822	74.841	23.211	1.00	0.00	H
ATOM	3272	HG22	THR	238	8.218	76.370	22.440	1.00	0.00	H
ATOM	3273	HG23	THR	238	9.194	75.774	23.778	1.00	0.00	H
ATOM	3274	N	GLY	239	9.616	79.397	25.498	1.00	2.61	N
ATOM	3275	CA	GLY	239	10.961	79.708	25.965	1.00	2.61	C
ATOM	3276	C	GLY	239	11.686	80.334	24.783	1.00	2.61	C
ATOM	3277	O	GLY	239	11.732	81.556	24.669	1.00	2.61	O
ATOM	3278	H	GLY	239	9.135	80.148	25.018	1.00	0.00	H
ATOM	3279	HA2	GLY	239	10.910	80.421	26.788	1.00	0.00	H
ATOM	3280	HA3	GLY	239	11.506	78.832	26.324	1.00	0.00	H
ATOM	3281	N	GLN	240	12.134	79.495	23.840	1.00	2.81	N
ATOM	3282	CA	GLN	240	12.532	79.922	22.498	1.00	2.81	C
ATOM	3283	C	GLN	240	11.288	80.343	21.694	1.00	2.81	C
ATOM	3284	O	GLN	240	10.170	79.887	21.969	1.00	2.81	O
ATOM	3285	CB	GLN	240	13.236	78.751	21.760	1.00	2.81	C
ATOM	3286	CG	GLN	240	14.768	78.705	21.947	1.00	2.81	C
ATOM	3287	CD	GLN	240	15.502	79.794	21.156	1.00	2.81	C
ATOM	3288	OE1	GLN	240	14.933	80.838	20.839	1.00	2.81	O
ATOM	3289	NE2	GLN	240	16.756	79.560	20.798	1.00	2.81	N
ATOM	3290	H	GLN	240	12.027	78.506	23.998	1.00	0.00	H
ATOM	3291	HA	GLN	240	13.186	80.790	22.591	1.00	0.00	H

ATOM	3292	HB2	GLN	240	12.805	77.798	22.067	1.00	0.00	H
ATOM	3293	HB3	GLN	240	13.047	78.805	20.685	1.00	0.00	H
ATOM	3294	HG2	GLN	240	15.031	78.792	23.001	1.00	0.00	H
ATOM	3295	HG3	GLN	240	15.141	77.735	21.614	1.00	0.00	H
ATOM	3296	HE22	GLN	240	17.264	80.245	20.269	1.00	0.00	H
ATOM	3297	HE21	GLN	240	17.199	78.624	20.935	1.00	0.00	H
ATOM	3298	N	HIE	241	11.478	81.203	20.689	1.00	2.82	N
ATOM	3299	CA	HIE	241	10.476	81.499	19.667	1.00	2.82	C
ATOM	3300	C	HIE	241	10.644	80.418	18.600	1.00	2.82	C
ATOM	3301	O	HIE	241	11.625	80.429	17.867	1.00	2.82	O
ATOM	3302	CB	HIE	241	10.745	82.874	19.017	1.00	2.82	C
ATOM	3303	CG	HIE	241	10.061	84.029	19.686	1.00	2.82	C
ATOM	3304	ND1	HIE	241	9.415	85.030	18.951	1.00	2.82	N
ATOM	3305	CD2	HIE	241	9.945	84.318	21.028	1.00	2.82	C
ATOM	3306	CE1	HIE	241	8.931	85.866	19.861	1.00	2.82	C
ATOM	3307	NE2	HIE	241	9.211	85.490	21.110	1.00	2.82	N
ATOM	3308	H	HIE	241	12.442	81.437	20.472	1.00	0.00	H
ATOM	3309	HA	HIE	241	9.459	81.466	20.060	1.00	0.00	H
ATOM	3310	HB2	HIE	241	11.814	83.077	18.936	1.00	0.00	H
ATOM	3311	HB3	HIE	241	10.370	82.863	17.993	1.00	0.00	H
ATOM	3312	HD2	HIE	241	10.312	83.794	21.901	1.00	0.00	H
ATOM	3313	HE1	HIE	241	8.371	86.758	19.615	1.00	0.00	H
ATOM	3314	HE2	HIE	241	8.950	85.976	21.956	1.00	0.00	H
ATOM	3315	N	TRP	242	9.713	79.465	18.551	1.00	2.75	N
ATOM	3316	CA	TRP	242	9.805	78.301	17.674	1.00	2.75	C
ATOM	3317	C	TRP	242	9.358	78.656	16.248	1.00	2.75	C
ATOM	3318	O	TRP	242	9.953	78.188	15.281	1.00	2.75	O
ATOM	3319	CB	TRP	242	8.862	77.212	18.229	1.00	2.75	C
ATOM	3320	CG	TRP	242	8.698	76.011	17.350	1.00	2.75	C
ATOM	3321	CD1	TRP	242	7.591	75.718	16.636	1.00	2.75	C
ATOM	3322	CD2	TRP	242	9.676	74.986	17.013	1.00	2.75	C
ATOM	3323	NE1	TRP	242	7.810	74.582	15.888	1.00	2.75	N
ATOM	3324	CE2	TRP	242	9.077	74.081	16.085	1.00	2.75	C
ATOM	3325	CE3	TRP	242	11.015	74.733	17.386	1.00	2.75	C
ATOM	3326	CZ2	TRP	242	9.766	72.974	15.569	1.00	2.75	C
ATOM	3327	CZ3	TRP	242	11.715	73.624	16.875	1.00	2.75	C
ATOM	3328	CH2	TRP	242	11.092	72.739	15.972	1.00	2.75	C
ATOM	3329	H	TRP	242	8.901	79.557	19.144	1.00	0.00	H
ATOM	3330	HA	TRP	242	10.830	77.921	17.639	1.00	0.00	H
ATOM	3331	HB2	TRP	242	9.227	76.864	19.196	1.00	0.00	H
ATOM	3332	HB3	TRP	242	7.871	77.630	18.408	1.00	0.00	H
ATOM	3333	HD1	TRP	242	6.700	76.323	16.635	1.00	0.00	H
ATOM	3334	HE1	TRP	242	7.115	74.206	15.227	1.00	0.00	H
ATOM	3335	HE3	TRP	242	11.510	75.395	18.079	1.00	0.00	H
ATOM	3336	HZ2	TRP	242	9.282	72.317	14.867	1.00	0.00	H
ATOM	3337	HZ3	TRP	242	12.732	73.453	17.198	1.00	0.00	H
ATOM	3338	HH2	TRP	242	11.627	71.887	15.577	1.00	0.00	H
ATOM	3339	N	TRP	243	8.300	79.471	16.138	1.00	2.76	N
ATOM	3340	CA	TRP	243	7.508	79.765	14.940	1.00	2.76	C
ATOM	3341	C	TRP	243	8.256	80.160	13.658	1.00	2.76	C
ATOM	3342	O	TRP	243	7.678	80.055	12.580	1.00	2.76	O
ATOM	3343	CB	TRP	243	6.418	80.797	15.276	1.00	2.76	C
ATOM	3344	CG	TRP	243	6.835	82.196	15.648	1.00	2.76	C
ATOM	3345	CD1	TRP	243	7.731	82.542	16.602	1.00	2.76	C
ATOM	3346	CD2	TRP	243	6.343	83.457	15.097	1.00	2.76	C
ATOM	3347	NE1	TRP	243	7.794	83.915	16.709	1.00	2.76	N
ATOM	3348	CE2	TRP	243	6.959	84.534	15.805	1.00	2.76	C
ATOM	3349	CE3	TRP	243	5.431	83.804	14.071	1.00	2.76	C
ATOM	3350	CZ2	TRP	243	6.672	85.880	15.524	1.00	2.76	C
ATOM	3351	CZ3	TRP	243	5.138	85.151	13.776	1.00	2.76	C
ATOM	3352	CH2	TRP	243	5.755	86.189	14.502	1.00	2.76	C
ATOM	3353	H	TRP	243	7.941	79.836	17.008	1.00	0.00	H
ATOM	3354	HA	TRP	243	7.001	78.829	14.694	1.00	0.00	H

ATOM	3355	HB2	TRP	243	5.750	80.878	14.417	1.00	0.00	H
ATOM	3356	HB3	TRP	243	5.801	80.406	16.081	1.00	0.00	H
ATOM	3357	HD1	TRP	243	8.287	81.850	17.213	1.00	0.00	H
ATOM	3358	HE1	TRP	243	8.422	84.390	17.361	1.00	0.00	H
ATOM	3359	HE3	TRP	243	4.950	83.020	13.506	1.00	0.00	H
ATOM	3360	HZ2	TRP	243	7.159	86.667	16.078	1.00	0.00	H
ATOM	3361	HZ3	TRP	243	4.437	85.388	12.990	1.00	0.00	H
ATOM	3362	HH2	TRP	243	5.531	87.220	14.272	1.00	0.00	H
ATOM	3363	N	TRP	244	9.521	80.583	13.757	1.00	2.81	N
ATOM	3364	CA	TRP	244	10.380	80.901	12.622	1.00	2.81	C
ATOM	3365	C	TRP	244	10.845	79.630	11.886	1.00	2.81	C
ATOM	3366	O	TRP	244	11.032	79.668	10.674	1.00	2.81	O
ATOM	3367	CB	TRP	244	11.624	81.664	13.119	1.00	2.81	C
ATOM	3368	CG	TRP	244	11.364	82.848	14.004	1.00	2.81	C
ATOM	3369	CD1	TRP	244	11.731	82.956	15.301	1.00	2.81	C
ATOM	3370	CD2	TRP	244	10.676	84.095	13.678	1.00	2.81	C
ATOM	3371	NE1	TRP	244	11.331	84.182	15.793	1.00	2.81	N
ATOM	3372	CE2	TRP	244	10.679	84.929	14.836	1.00	2.81	C
ATOM	3373	CE3	TRP	244	10.048	84.610	12.519	1.00	2.81	C
ATOM	3374	CZ2	TRP	244	10.101	86.211	14.842	1.00	2.81	C
ATOM	3375	CZ3	TRP	244	9.459	85.891	12.513	1.00	2.81	C
ATOM	3376	CH2	TRP	244	9.489	86.693	13.671	1.00	2.81	C
ATOM	3377	H	TRP	244	9.924	80.623	14.681	1.00	0.00	H
ATOM	3378	HA	TRP	244	9.827	81.536	11.927	1.00	0.00	H
ATOM	3379	HB2	TRP	244	12.290	80.985	13.651	1.00	0.00	H
ATOM	3380	HB3	TRP	244	12.195	82.017	12.259	1.00	0.00	H
ATOM	3381	HD1	TRP	244	12.260	82.191	15.853	1.00	0.00	H
ATOM	3382	HE1	TRP	244	11.500	84.468	16.747	1.00	0.00	H
ATOM	3383	HE3	TRP	244	10.022	84.007	11.623	1.00	0.00	H
ATOM	3384	HZ2	TRP	244	10.122	86.814	15.737	1.00	0.00	H
ATOM	3385	HZ3	TRP	244	8.984	86.257	11.615	1.00	0.00	H
ATOM	3386	HH2	TRP	244	9.035	87.673	13.662	1.00	0.00	H
ATOM	3387	N	VAL	245	11.020	78.501	12.587	1.00	2.62	N
ATOM	3388	CA	VAL	245	11.456	77.226	12.009	1.00	2.62	C
ATOM	3389	C	VAL	245	10.496	76.675	10.958	1.00	2.62	C
ATOM	3390	O	VAL	245	10.900	76.639	9.791	1.00	2.62	O
ATOM	3391	CB	VAL	245	11.829	76.214	13.150	1.00	2.62	C
ATOM	3392	CG1	VAL	245	11.851	74.723	12.744	1.00	2.62	C
ATOM	3393	CG2	VAL	245	13.161	76.566	13.810	1.00	2.62	C
ATOM	3394	H	VAL	245	10.765	78.499	13.571	1.00	0.00	H
ATOM	3395	HA	VAL	245	12.383	77.434	11.470	1.00	0.00	H
ATOM	3396	HB	VAL	245	11.123	76.317	13.968	1.00	0.00	H
ATOM	3397	HG11	VAL	245	12.195	74.103	13.571	1.00	0.00	H
ATOM	3398	HG12	VAL	245	10.866	74.341	12.476	1.00	0.00	H
ATOM	3399	HG13	VAL	245	12.514	74.543	11.899	1.00	0.00	H
ATOM	3400	HG21	VAL	245	13.282	76.052	14.765	1.00	0.00	H
ATOM	3401	HG22	VAL	245	13.996	76.271	13.183	1.00	0.00	H
ATOM	3402	HG23	VAL	245	13.227	77.634	13.997	1.00	0.00	H
ATOM	3403	N	PRO	246	9.242	76.301	11.308	1.00	2.73	N
ATOM	3404	CA	PRO	246	8.290	75.730	10.353	1.00	2.73	C
ATOM	3405	C	PRO	246	7.975	76.580	9.117	1.00	2.73	C
ATOM	3406	O	PRO	246	7.264	76.100	8.240	1.00	2.73	O
ATOM	3407	CB	PRO	246	6.965	75.585	11.120	1.00	2.73	C
ATOM	3408	CG	PRO	246	7.351	75.561	12.574	1.00	2.73	C
ATOM	3409	CD	PRO	246	8.668	76.306	12.660	1.00	2.73	C
ATOM	3410	HA	PRO	246	8.657	74.752	10.037	1.00	0.00	H
ATOM	3411	HB2	PRO	246	6.303	76.440	10.964	1.00	0.00	H
ATOM	3412	HB3	PRO	246	6.409	74.695	10.824	1.00	0.00	H
ATOM	3413	HG2	PRO	246	6.586	75.975	13.231	1.00	0.00	H
ATOM	3414	HG3	PRO	246	7.532	74.525	12.861	1.00	0.00	H
ATOM	3415	HD3	PRO	246	9.258	75.790	13.407	1.00	0.00	H
ATOM	3416	HD2	PRO	246	8.507	77.331	12.989	1.00	0.00	H
ATOM	3417	N	ILE	247	8.451	77.827	9.058	1.00	2.75	N

ATOM	3418	CA	ILE	247	8.122	78.770	8.019	1.00	2.75	C
ATOM	3419	C	ILE	247	9.307	79.187	7.143	1.00	2.75	C
ATOM	3420	O	ILE	247	9.088	79.886	6.157	1.00	2.75	O
ATOM	3421	CB	ILE	247	7.312	79.967	8.593	1.00	2.75	C
ATOM	3422	CG1	ILE	247	8.162	80.966	9.412	1.00	2.75	C
ATOM	3423	CG2	ILE	247	6.089	79.471	9.388	1.00	2.75	C
ATOM	3424	CD1	ILE	247	7.394	82.201	9.908	1.00	2.75	C
ATOM	3425	H	ILE	247	9.006	78.177	9.825	1.00	0.00	H
ATOM	3426	HA	ILE	247	7.456	78.294	7.300	1.00	0.00	H
ATOM	3427	HB	ILE	247	6.927	80.518	7.733	1.00	0.00	H
ATOM	3428	HG12	ILE	247	8.590	80.446	10.263	1.00	0.00	H
ATOM	3429	HG13	ILE	247	9.008	81.317	8.820	1.00	0.00	H
ATOM	3430	HG21	ILE	247	5.395	80.284	9.580	1.00	0.00	H
ATOM	3431	HG22	ILE	247	5.552	78.698	8.838	1.00	0.00	H
ATOM	3432	HG23	ILE	247	6.377	79.051	10.353	1.00	0.00	H
ATOM	3433	HD11	ILE	247	8.070	82.903	10.395	1.00	0.00	H
ATOM	3434	HD12	ILE	247	6.905	82.724	9.085	1.00	0.00	H
ATOM	3435	HD13	ILE	247	6.633	81.933	10.641	1.00	0.00	H
ATOM	3436	N	VAL	248	10.534	78.738	7.442	1.00	2.67	N
ATOM	3437	CA	VAL	248	11.688	78.958	6.569	1.00	2.67	C
ATOM	3438	C	VAL	248	12.613	77.765	6.401	1.00	2.67	C
ATOM	3439	O	VAL	248	13.175	77.565	5.322	1.00	2.67	O
ATOM	3440	CB	VAL	248	12.325	80.374	6.719	1.00	2.67	C
ATOM	3441	CG1	VAL	248	12.659	80.744	8.169	1.00	2.67	C
ATOM	3442	CG2	VAL	248	13.541	80.616	5.803	1.00	2.67	C
ATOM	3443	H	VAL	248	10.647	78.201	8.291	1.00	0.00	H
ATOM	3444	HA	VAL	248	11.275	78.988	5.559	1.00	0.00	H
ATOM	3445	HB	VAL	248	11.570	81.090	6.390	1.00	0.00	H
ATOM	3446	HG11	VAL	248	13.271	81.644	8.227	1.00	0.00	H
ATOM	3447	HG12	VAL	248	11.744	80.955	8.720	1.00	0.00	H
ATOM	3448	HG13	VAL	248	13.187	79.940	8.677	1.00	0.00	H
ATOM	3449	HG21	VAL	248	13.856	81.659	5.835	1.00	0.00	H
ATOM	3450	HG22	VAL	248	14.397	80.006	6.091	1.00	0.00	H
ATOM	3451	HG23	VAL	248	13.302	80.383	4.764	1.00	0.00	H
ATOM	3452	N	SER	249	12.698	76.919	7.423	1.00	2.46	N
ATOM	3453	CA	SER	249	13.367	75.635	7.353	1.00	2.46	C
ATOM	3454	C	SER	249	12.840	74.733	6.211	1.00	2.46	C
ATOM	3455	O	SER	249	13.657	74.331	5.377	1.00	2.46	O
ATOM	3456	CB	SER	249	13.369	75.039	8.774	1.00	2.46	C
ATOM	3457	OG	SER	249	13.982	73.793	8.858	1.00	2.46	O
ATOM	3458	H	SER	249	12.191	77.119	8.276	1.00	0.00	H
ATOM	3459	HA	SER	249	14.410	75.845	7.106	1.00	0.00	H
ATOM	3460	HB2	SER	249	13.862	75.715	9.474	1.00	0.00	H
ATOM	3461	HB3	SER	249	12.369	74.884	9.156	1.00	0.00	H
ATOM	3462	HG	SER	249	14.926	73.873	9.001	1.00	0.00	H
ATOM	3463	N	PRO	250	11.508	74.510	6.059	1.00	2.42	N
ATOM	3464	CA	PRO	250	10.958	73.848	4.875	1.00	2.42	C
ATOM	3465	C	PRO	250	11.310	74.458	3.517	1.00	2.42	C
ATOM	3466	O	PRO	250	11.544	73.712	2.567	1.00	2.42	O
ATOM	3467	CB	PRO	250	9.432	73.873	5.030	1.00	2.42	C
ATOM	3468	CG	PRO	250	9.196	74.040	6.509	1.00	2.42	C
ATOM	3469	CD	PRO	250	10.442	74.754	7.039	1.00	2.42	C
ATOM	3470	HA	PRO	250	11.320	72.824	4.899	1.00	0.00	H
ATOM	3471	HB2	PRO	250	8.992	74.723	4.505	1.00	0.00	H
ATOM	3472	HB3	PRO	250	8.958	72.972	4.642	1.00	0.00	H
ATOM	3473	HG2	PRO	250	8.264	74.560	6.708	1.00	0.00	H
ATOM	3474	HG3	PRO	250	9.118	73.056	6.970	1.00	0.00	H
ATOM	3475	HD3	PRO	250	10.674	74.379	8.031	1.00	0.00	H
ATOM	3476	HD2	PRO	250	10.248	75.824	7.113	1.00	0.00	H
ATOM	3477	N	LEU	251	11.334	75.793	3.412	1.00	2.16	N
ATOM	3478	CA	LEU	251	11.578	76.500	2.157	1.00	2.16	C
ATOM	3479	C	LEU	251	12.999	76.267	1.668	1.00	2.16	C
ATOM	3480	O	LEU	251	13.218	75.892	0.519	1.00	2.16	O

ATOM	3481	CB	LEU	251	11.371	78.029	2.336	1.00	2.16	C
ATOM	3482	CG	LEU	251	9.995	78.503	2.854	1.00	2.16	C
ATOM	3483	CD1	LEU	251	9.990	80.031	3.017	1.00	2.16	C
ATOM	3484	CD2	LEU	251	8.840	78.076	1.941	1.00	2.16	C
ATOM	3485	H	LEU	251	11.186	76.349	4.239	1.00	0.00	H
ATOM	3486	HA	LEU	251	10.893	76.129	1.393	1.00	0.00	H
ATOM	3487	HB2	LEU	251	12.137	78.423	3.005	1.00	0.00	H
ATOM	3488	HB3	LEU	251	11.560	78.512	1.375	1.00	0.00	H
ATOM	3489	HG	LEU	251	9.819	78.071	3.840	1.00	0.00	H
ATOM	3490	HD11	LEU	251	9.045	80.379	3.437	1.00	0.00	H
ATOM	3491	HD12	LEU	251	10.780	80.367	3.689	1.00	0.00	H
ATOM	3492	HD13	LEU	251	10.132	80.535	2.060	1.00	0.00	H
ATOM	3493	HD21	LEU	251	7.897	78.522	2.260	1.00	0.00	H
ATOM	3494	HD22	LEU	251	9.007	78.376	0.905	1.00	0.00	H
ATOM	3495	HD23	LEU	251	8.709	76.998	1.970	1.00	0.00	H
ATOM	3496	N	LEU	252	13.948	76.422	2.592	1.00	2.06	N
ATOM	3497	CA	LEU	252	15.355	76.180	2.356	1.00	2.06	C
ATOM	3498	C	LEU	252	15.624	74.685	2.085	1.00	2.06	C
ATOM	3499	O	LEU	252	16.463	74.350	1.252	1.00	2.06	O
ATOM	3500	CB	LEU	252	16.121	76.659	3.615	1.00	2.06	C
ATOM	3501	CG	LEU	252	17.658	76.731	3.481	1.00	2.06	C
ATOM	3502	CD1	LEU	252	18.108	77.871	2.552	1.00	2.06	C
ATOM	3503	CD2	LEU	252	18.316	76.871	4.862	1.00	2.06	C
ATOM	3504	H	LEU	252	13.666	76.703	3.524	1.00	0.00	H
ATOM	3505	HA	LEU	252	15.661	76.756	1.481	1.00	0.00	H
ATOM	3506	HB2	LEU	252	15.757	77.644	3.910	1.00	0.00	H
ATOM	3507	HB3	LEU	252	15.865	75.999	4.446	1.00	0.00	H
ATOM	3508	HG	LEU	252	18.022	75.798	3.053	1.00	0.00	H
ATOM	3509	HD11	LEU	252	19.196	77.916	2.488	1.00	0.00	H
ATOM	3510	HD12	LEU	252	17.738	77.732	1.536	1.00	0.00	H
ATOM	3511	HD13	LEU	252	17.757	78.840	2.908	1.00	0.00	H
ATOM	3512	HD21	LEU	252	19.404	76.892	4.779	1.00	0.00	H
ATOM	3513	HD22	LEU	252	18.002	77.786	5.365	1.00	0.00	H
ATOM	3514	HD23	LEU	252	18.061	76.029	5.506	1.00	0.00	H
ATOM	3515	N	GLY	253	14.857	73.796	2.734	1.00	1.93	N
ATOM	3516	CA	GLY	253	14.934	72.350	2.566	1.00	1.93	C
ATOM	3517	C	GLY	253	14.466	71.917	1.192	1.00	1.93	C
ATOM	3518	O	GLY	253	15.178	71.198	0.497	1.00	1.93	O
ATOM	3519	H	GLY	253	14.217	74.149	3.436	1.00	0.00	H
ATOM	3520	HA2	GLY	253	15.954	71.999	2.728	1.00	0.00	H
ATOM	3521	HA3	GLY	253	14.310	71.871	3.319	1.00	0.00	H
ATOM	3522	N	SER	254	13.316	72.437	0.763	1.00	1.87	N
ATOM	3523	CA	SER	254	12.706	72.153	-0.529	1.00	1.87	C
ATOM	3524	C	SER	254	13.623	72.476	-1.709	1.00	1.87	C
ATOM	3525	O	SER	254	13.709	71.720	-2.674	1.00	1.87	O
ATOM	3526	CB	SER	254	11.413	72.979	-0.644	1.00	1.87	C
ATOM	3527	OG	SER	254	10.479	72.527	0.310	1.00	1.87	O
ATOM	3528	H	SER	254	12.814	73.061	1.386	1.00	0.00	H
ATOM	3529	HA	SER	254	12.496	71.085	-0.559	1.00	0.00	H
ATOM	3530	HB2	SER	254	11.597	74.045	-0.504	1.00	0.00	H
ATOM	3531	HB3	SER	254	10.968	72.878	-1.633	1.00	0.00	H
ATOM	3532	HG	SER	254	10.800	72.782	1.170	1.00	0.00	H
ATOM	3533	N	ILE	255	14.352	73.586	-1.592	1.00	1.87	N
ATOM	3534	CA	ILE	255	15.263	74.054	-2.613	1.00	1.87	C
ATOM	3535	C	ILE	255	16.569	73.248	-2.642	1.00	1.87	C
ATOM	3536	O	ILE	255	17.153	73.065	-3.709	1.00	1.87	O
ATOM	3537	CB	ILE	255	15.519	75.579	-2.373	1.00	1.87	C
ATOM	3538	CG1	ILE	255	14.216	76.396	-2.586	1.00	1.87	C
ATOM	3539	CG2	ILE	255	16.642	76.173	-3.256	1.00	1.87	C
ATOM	3540	CD1	ILE	255	14.252	77.815	-1.999	1.00	1.87	C
ATOM	3541	H	ILE	255	14.245	74.152	-0.760	1.00	0.00	H
ATOM	3542	HA	ILE	255	14.780	73.957	-3.585	1.00	0.00	H
ATOM	3543	HB	ILE	255	15.832	75.699	-1.333	1.00	0.00	H

ATOM	3544	HG12	ILE	255	13.984	76.450	-3.651	1.00	0.00	H
ATOM	3545	HG13	ILE	255	13.365	75.886	-2.135	1.00	0.00	H
ATOM	3546	HG21	ILE	255	16.769	77.242	-3.093	1.00	0.00	H
ATOM	3547	HG22	ILE	255	17.613	75.723	-3.048	1.00	0.00	H
ATOM	3548	HG23	ILE	255	16.430	76.024	-4.316	1.00	0.00	H
ATOM	3549	HD11	ILE	255	13.281	78.298	-2.108	1.00	0.00	H
ATOM	3550	HD12	ILE	255	14.495	77.798	-0.936	1.00	0.00	H
ATOM	3551	HD13	ILE	255	14.983	78.449	-2.499	1.00	0.00	H
ATOM	3552	N	ALA	256	17.007	72.712	-1.501	1.00	2.04	N
ATOM	3553	CA	ALA	256	18.140	71.802	-1.465	1.00	2.04	C
ATOM	3554	C	ALA	256	17.759	70.422	-2.016	1.00	2.04	C
ATOM	3555	O	ALA	256	18.549	69.800	-2.721	1.00	2.04	O
ATOM	3556	CB	ALA	256	18.600	71.645	-0.006	1.00	2.04	C
ATOM	3557	H	ALA	256	16.497	72.888	-0.645	1.00	0.00	H
ATOM	3558	HA	ALA	256	18.965	72.205	-2.058	1.00	0.00	H
ATOM	3559	HB1	ALA	256	19.466	70.985	0.060	1.00	0.00	H
ATOM	3560	HB2	ALA	256	18.890	72.607	0.416	1.00	0.00	H
ATOM	3561	HB3	ALA	256	17.820	71.227	0.631	1.00	0.00	H
ATOM	3562	N	GLY	257	16.518	69.991	-1.768	1.00	2.03	N
ATOM	3563	CA	GLY	257	16.003	68.695	-2.184	1.00	2.03	C
ATOM	3564	C	GLY	257	15.693	68.636	-3.669	1.00	2.03	C
ATOM	3565	O	GLY	257	15.904	67.604	-4.300	1.00	2.03	O
ATOM	3566	H	GLY	257	15.939	70.560	-1.158	1.00	0.00	H
ATOM	3567	HA2	GLY	257	16.653	67.881	-1.864	1.00	0.00	H
ATOM	3568	HA3	GLY	257	15.047	68.548	-1.692	1.00	0.00	H
ATOM	3569	N	VAL	258	15.246	69.754	-4.253	1.00	2.08	N
ATOM	3570	CA	VAL	258	15.005	69.802	-5.683	1.00	2.08	C
ATOM	3571	C	VAL	258	16.313	69.839	-6.486	1.00	2.08	C
ATOM	3572	O	VAL	258	16.437	69.173	-7.514	1.00	2.08	O
ATOM	3573	CB	VAL	258	14.067	71.004	-6.054	1.00	2.08	C
ATOM	3574	CG1	VAL	258	14.721	72.393	-6.016	1.00	2.08	C
ATOM	3575	CG2	VAL	258	13.352	70.841	-7.408	1.00	2.08	C
ATOM	3576	H	VAL	258	15.005	70.556	-3.682	1.00	0.00	H
ATOM	3577	HA	VAL	258	14.504	68.880	-5.957	1.00	0.00	H
ATOM	3578	HB	VAL	258	13.278	71.029	-5.306	1.00	0.00	H
ATOM	3579	HG11	VAL	258	13.977	73.188	-6.062	1.00	0.00	H
ATOM	3580	HG12	VAL	258	15.274	72.508	-5.097	1.00	0.00	H
ATOM	3581	HG13	VAL	258	15.413	72.545	-6.843	1.00	0.00	H
ATOM	3582	HG21	VAL	258	12.850	71.763	-7.705	1.00	0.00	H
ATOM	3583	HG22	VAL	258	14.046	70.565	-8.203	1.00	0.00	H
ATOM	3584	HG23	VAL	258	12.575	70.085	-7.370	1.00	0.00	H
ATOM	3585	N	PHE	259	17.294	70.591	-5.978	1.00	2.26	N
ATOM	3586	CA	PHE	259	18.613	70.751	-6.567	1.00	2.26	C
ATOM	3587	C	PHE	259	19.377	69.430	-6.528	1.00	2.26	C
ATOM	3588	O	PHE	259	19.943	69.024	-7.540	1.00	2.26	O
ATOM	3589	CB	PHE	259	19.343	71.855	-5.768	1.00	2.26	C
ATOM	3590	CG	PHE	259	20.707	72.289	-6.275	1.00	2.26	C
ATOM	3591	CD1	PHE	259	20.806	73.250	-7.303	1.00	2.26	C
ATOM	3592	CD2	PHE	259	21.880	71.763	-5.693	1.00	2.26	C
ATOM	3593	CE1	PHE	259	22.071	73.692	-7.737	1.00	2.26	C
ATOM	3594	CE2	PHE	259	23.144	72.202	-6.131	1.00	2.26	C
ATOM	3595	CZ	PHE	259	23.240	73.170	-7.150	1.00	2.26	C
ATOM	3596	H	PHE	259	17.107	71.082	-5.115	1.00	0.00	H
ATOM	3597	HA	PHE	259	18.516	71.057	-7.608	1.00	0.00	H
ATOM	3598	HB2	PHE	259	18.720	72.749	-5.757	1.00	0.00	H
ATOM	3599	HB3	PHE	259	19.441	71.559	-4.722	1.00	0.00	H
ATOM	3600	HD1	PHE	259	19.915	73.657	-7.758	1.00	0.00	H
ATOM	3601	HD2	PHE	259	21.817	71.022	-4.909	1.00	0.00	H
ATOM	3602	HE1	PHE	259	22.146	74.427	-8.525	1.00	0.00	H
ATOM	3603	HE2	PHE	259	24.041	71.794	-5.687	1.00	0.00	H
ATOM	3604	HZ	PHE	259	24.211	73.503	-7.487	1.00	0.00	H
ATOM	3605	N	VAL	260	19.329	68.725	-5.388	1.00	2.44	N
ATOM	3606	CA	VAL	260	20.033	67.464	-5.235	1.00	2.44	C

ATOM	3607	C	VAL	260	19.409	66.317	-6.033	1.00	2.44	C
ATOM	3608	O	VAL	260	20.122	65.380	-6.373	1.00	2.44	O
ATOM	3609	CB	VAL	260	20.220	67.121	-3.725	1.00	2.44	C
ATOM	3610	CG1	VAL	260	18.982	66.524	-3.041	1.00	2.44	C
ATOM	3611	CG2	VAL	260	21.435	66.212	-3.475	1.00	2.44	C
ATOM	3612	H	VAL	260	18.851	69.100	-4.577	1.00	0.00	H
ATOM	3613	HA	VAL	260	21.031	67.615	-5.653	1.00	0.00	H
ATOM	3614	HB	VAL	260	20.450	68.057	-3.215	1.00	0.00	H
ATOM	3615	HG11	VAL	260	19.089	66.521	-1.956	1.00	0.00	H
ATOM	3616	HG12	VAL	260	18.105	67.106	-3.289	1.00	0.00	H
ATOM	3617	HG13	VAL	260	18.795	65.498	-3.360	1.00	0.00	H
ATOM	3618	HG21	VAL	260	21.612	66.075	-2.408	1.00	0.00	H
ATOM	3619	HG22	VAL	260	21.288	65.223	-3.910	1.00	0.00	H
ATOM	3620	HG23	VAL	260	22.344	66.634	-3.905	1.00	0.00	H
ATOM	3621	N	TYR	261	18.123	66.398	-6.399	1.00	2.51	N
ATOM	3622	CA	TYR	261	17.533	65.402	-7.281	1.00	2.51	C
ATOM	3623	C	TYR	261	18.110	65.524	-8.692	1.00	2.51	C
ATOM	3624	O	TYR	261	18.641	64.562	-9.241	1.00	2.51	O
ATOM	3625	CB	TYR	261	15.993	65.560	-7.386	1.00	2.51	C
ATOM	3626	CG	TYR	261	15.356	64.497	-8.269	1.00	2.51	C
ATOM	3627	CD1	TYR	261	15.415	63.143	-7.879	1.00	2.51	C
ATOM	3628	CD2	TYR	261	14.806	64.833	-9.526	1.00	2.51	C
ATOM	3629	CE1	TYR	261	14.969	62.138	-8.753	1.00	2.51	C
ATOM	3630	CE2	TYR	261	14.339	63.822	-10.391	1.00	2.51	C
ATOM	3631	CZ	TYR	261	14.437	62.473	-10.007	1.00	2.51	C
ATOM	3632	OH	TYR	261	14.039	61.473	-10.835	1.00	2.51	O
ATOM	3633	H	TYR	261	17.568	67.183	-6.088	1.00	0.00	H
ATOM	3634	HA	TYR	261	17.762	64.407	-6.895	1.00	0.00	H
ATOM	3635	HB2	TYR	261	15.518	65.503	-6.412	1.00	0.00	H
ATOM	3636	HB3	TYR	261	15.738	66.546	-7.772	1.00	0.00	H
ATOM	3637	HD1	TYR	261	15.844	62.856	-6.932	1.00	0.00	H
ATOM	3638	HD2	TYR	261	14.769	65.858	-9.859	1.00	0.00	H
ATOM	3639	HE1	TYR	261	15.048	61.098	-8.474	1.00	0.00	H
ATOM	3640	HE2	TYR	261	13.942	64.074	-11.366	1.00	0.00	H
ATOM	3641	HH	TYR	261	13.733	61.749	-11.724	1.00	0.00	H
ATOM	3642	N	GLN	262	18.043	66.731	-9.254	1.00	2.82	N
ATOM	3643	CA	GLN	262	18.369	67.000	-10.650	1.00	2.82	C
ATOM	3644	C	GLN	262	19.867	67.034	-10.932	1.00	2.82	C
ATOM	3645	O	GLN	262	20.275	67.247	-12.072	1.00	2.82	O
ATOM	3646	CB	GLN	262	17.724	68.355	-11.014	1.00	2.82	C
ATOM	3647	CG	GLN	262	16.180	68.267	-11.007	1.00	2.82	C
ATOM	3648	CD	GLN	262	15.484	69.448	-11.677	1.00	2.82	C
ATOM	3649	OE1	GLN	262	16.049	70.157	-12.507	1.00	2.82	O
ATOM	3650	NE2	GLN	262	14.230	69.692	-11.322	1.00	2.82	N
ATOM	3651	H	GLN	262	17.678	67.497	-8.705	1.00	0.00	H
ATOM	3652	HA	GLN	262	17.952	66.211	-11.275	1.00	0.00	H
ATOM	3653	HB2	GLN	262	18.058	69.134	-10.327	1.00	0.00	H
ATOM	3654	HB3	GLN	262	18.071	68.670	-11.998	1.00	0.00	H
ATOM	3655	HG2	GLN	262	15.857	67.366	-11.529	1.00	0.00	H
ATOM	3656	HG3	GLN	262	15.823	68.171	-9.982	1.00	0.00	H
ATOM	3657	HE22	GLN	262	13.747	70.477	-11.746	1.00	0.00	H
ATOM	3658	HE21	GLN	262	13.755	69.062	-10.696	1.00	0.00	H
ATOM	3659	N	LEU	263	20.668	66.824	-9.889	1.00	3.34	N
ATOM	3660	CA	LEU	263	22.107	66.825	-9.942	1.00	3.34	C
ATOM	3661	C	LEU	263	22.775	65.643	-9.265	1.00	3.34	C
ATOM	3662	O	LEU	263	24.004	65.576	-9.252	1.00	3.34	O
ATOM	3663	CB	LEU	263	22.673	68.182	-9.447	1.00	3.34	C
ATOM	3664	CG	LEU	263	22.280	69.430	-10.271	1.00	3.34	C
ATOM	3665	CD1	LEU	263	22.637	70.710	-9.507	1.00	3.34	C
ATOM	3666	CD2	LEU	263	22.930	69.442	-11.665	1.00	3.34	C
ATOM	3667	H	LEU	263	20.230	66.731	-8.985	1.00	0.00	H
ATOM	3668	HA	LEU	263	22.423	66.700	-10.979	1.00	0.00	H
ATOM	3669	HB2	LEU	263	22.356	68.319	-8.413	1.00	0.00	H

ATOM	3670	HB3	LEU	263	23.763	68.133	-9.404	1.00	0.00	H
ATOM	3671	HG	LEU	263	21.198	69.443	-10.400	1.00	0.00	H
ATOM	3672	HD11	LEU	263	22.334	71.600	-10.058	1.00	0.00	H
ATOM	3673	HD12	LEU	263	22.125	70.735	-8.544	1.00	0.00	H
ATOM	3674	HD13	LEU	263	23.708	70.779	-9.316	1.00	0.00	H
ATOM	3675	HD21	LEU	263	22.654	70.339	-12.219	1.00	0.00	H
ATOM	3676	HD22	LEU	263	24.018	69.413	-11.597	1.00	0.00	H
ATOM	3677	HD23	LEU	263	22.613	68.589	-12.264	1.00	0.00	H
ATOM	3678	N	MET	264	21.992	64.711	-8.724	1.00	3.81	N
ATOM	3679	CA	MET	264	22.507	63.437	-8.259	1.00	3.81	C
ATOM	3680	C	MET	264	21.710	62.243	-8.824	1.00	3.81	C
ATOM	3681	O	MET	264	22.174	61.116	-8.672	1.00	3.81	O
ATOM	3682	CB	MET	264	22.647	63.356	-6.719	1.00	3.81	C
ATOM	3683	CG	MET	264	23.655	64.324	-6.072	1.00	3.81	C
ATOM	3684	SD	MET	264	25.292	63.641	-5.670	1.00	3.81	S
ATOM	3685	CE	MET	264	25.992	63.387	-7.323	1.00	3.81	C
ATOM	3686	H	MET	264	20.992	64.838	-8.738	1.00	0.00	H
ATOM	3687	HA	MET	264	23.498	63.273	-8.682	1.00	0.00	H
ATOM	3688	HB2	MET	264	21.667	63.502	-6.272	1.00	0.00	H
ATOM	3689	HB3	MET	264	22.920	62.340	-6.430	1.00	0.00	H
ATOM	3690	HG2	MET	264	23.773	65.240	-6.649	1.00	0.00	H
ATOM	3691	HG3	MET	264	23.239	64.641	-5.118	1.00	0.00	H
ATOM	3692	HE1	MET	264	27.042	63.106	-7.245	1.00	0.00	H
ATOM	3693	HE2	MET	264	25.469	62.586	-7.845	1.00	0.00	H
ATOM	3694	HE3	MET	264	25.925	64.298	-7.917	1.00	0.00	H
ATOM	3695	N	ILE	265	20.538	62.455	-9.452	1.00	4.27	N
ATOM	3696	CA	ILE	265	19.654	61.421	-9.999	1.00	4.27	C
ATOM	3697	C	ILE	265	19.146	61.827	-11.400	1.00	4.27	C
ATOM	3698	O	ILE	265	19.669	61.354	-12.406	1.00	4.27	O
ATOM	3699	CB	ILE	265	18.525	61.004	-8.992	1.00	4.27	C
ATOM	3700	CG1	ILE	265	19.014	60.564	-7.589	1.00	4.27	C
ATOM	3701	CG2	ILE	265	17.559	59.943	-9.570	1.00	4.27	C
ATOM	3702	CD1	ILE	265	19.715	59.196	-7.545	1.00	4.27	C
ATOM	3703	H	ILE	265	20.203	63.400	-9.574	1.00	0.00	H
ATOM	3704	HA	ILE	265	20.255	60.528	-10.182	1.00	0.00	H
ATOM	3705	HB	ILE	265	17.926	61.898	-8.817	1.00	0.00	H
ATOM	3706	HG12	ILE	265	19.666	61.323	-7.160	1.00	0.00	H
ATOM	3707	HG13	ILE	265	18.155	60.525	-6.919	1.00	0.00	H
ATOM	3708	HG21	ILE	265	16.849	59.594	-8.820	1.00	0.00	H
ATOM	3709	HG22	ILE	265	16.965	60.329	-10.399	1.00	0.00	H
ATOM	3710	HG23	ILE	265	18.096	59.069	-9.940	1.00	0.00	H
ATOM	3711	HD11	ILE	265	20.097	58.991	-6.545	1.00	0.00	H
ATOM	3712	HD12	ILE	265	19.035	58.386	-7.807	1.00	0.00	H
ATOM	3713	HD13	ILE	265	20.560	59.152	-8.231	1.00	0.00	H
ATOM	3714	N	GLY	266	18.085	62.644	-11.467	1.00	4.98	N
ATOM	3715	CA	GLY	266	17.233	62.778	-12.647	1.00	4.98	C
ATOM	3716	C	GLY	266	17.092	64.215	-13.142	1.00	4.98	C
ATOM	3717	O	GLY	266	16.183	64.931	-12.725	1.00	4.98	O
ATOM	3718	H	GLY	266	17.750	63.062	-10.610	1.00	0.00	H
ATOM	3719	HA2	GLY	266	17.565	62.144	-13.471	1.00	0.00	H
ATOM	3720	HA3	GLY	266	16.239	62.410	-12.388	1.00	0.00	H
ATOM	3721	N	CYS	267	17.964	64.637	-14.063	1.00	5.92	N
ATOM	3722	CA	CYS	267	17.735	65.731	-15.015	1.00	5.92	C
ATOM	3723	C	CYS	267	18.768	65.602	-16.138	1.00	5.92	C
ATOM	3724	O	CYS	267	19.527	66.531	-16.424	1.00	5.92	O
ATOM	3725	CB	CYS	267	17.692	67.123	-14.345	1.00	5.92	C
ATOM	3726	SG	CYS	267	16.794	68.321	-15.379	1.00	5.92	S
ATOM	3727	H	CYS	267	18.771	64.051	-14.240	1.00	0.00	H
ATOM	3728	HA	CYS	267	16.760	65.540	-15.468	1.00	0.00	H
ATOM	3729	HB2	CYS	267	17.155	67.077	-13.406	1.00	0.00	H
ATOM	3730	HB3	CYS	267	18.692	67.494	-14.124	1.00	0.00	H
ATOM	3731	HG	CYS	267	15.786	67.485	-15.665	1.00	0.00	H
ATOM	3732	N	HIS	268	18.883	64.390	-16.689	1.00	6.43	N

```
ATOM   3733  CA  HIS   268      19.935  63.971 -17.617  1.00  6.43           C
ATOM   3734  C   HIS   268      19.334  63.274 -18.849  1.00  6.43           C
ATOM   3735  O   HIS   268      20.061  62.660 -19.627  1.00  6.43           O
ATOM   3736  CB  HIS   268      21.028  63.150 -16.883  1.00  6.43           C
ATOM   3737  CG  HIS   268      21.488  63.674 -15.531  1.00  6.43           C
ATOM   3738  ND1 HIS   268      21.745  65.013 -15.275  1.00  6.43           N
ATOM   3739  CD2 HIS   268      21.727  63.032 -14.335  1.00  6.43           C
ATOM   3740  CE1 HIS   268      22.059  65.131 -13.984  1.00  6.43           C
ATOM   3741  NE2 HIS   268      22.087  63.965 -13.357  1.00  6.43           N
ATOM   3742  H   HIS   268      18.183  63.696 -16.466  1.00  0.00           H
ATOM   3743  HA  HIS   268      20.423  64.858 -18.023  1.00  0.00           H
ATOM   3744  HB2 HIS   268      20.672  62.130 -16.731  1.00  0.00           H
ATOM   3745  HB3 HIS   268      21.903  63.062 -17.529  1.00  0.00           H
ATOM   3746  HD1 HIS   268      21.515  65.789 -15.888  1.00  0.00           H
ATOM   3747  HD2 HIS   268      21.642  61.981 -14.092  1.00  0.00           H
ATOM   3748  HE1 HIS   268      22.238  66.077 -13.490  1.00  0.00           H
ATOM   3749  N   LEU   269      18.014  63.398 -19.004  1.00  7.20           N
ATOM   3750  CA  LEU   269      17.118  63.040 -20.085  1.00  7.20           C
ATOM   3751  C   LEU   269      15.995  64.094 -19.990  1.00  7.20           C
ATOM   3752  O   LEU   269      15.065  64.043 -20.824  1.00  7.20           O
ATOM   3753  CB  LEU   269      16.588  61.592 -19.952  1.00  7.20           C
ATOM   3754  CG  LEU   269      17.611  60.441 -20.060  1.00  7.20           C
ATOM   3755  CD1 LEU   269      16.939  59.103 -19.715  1.00  7.20           C
ATOM   3756  CD2 LEU   269      18.265  60.372 -21.450  1.00  7.20           C
ATOM   3757  OXT LEU   269      16.053  64.911 -19.033  1.00  7.20           O1-
ATOM   3758  H   LEU   269      17.511  64.098 -18.455  1.00  0.00           H
ATOM   3759  HA  LEU   269      17.618  63.181 -21.043  1.00  0.00           H
ATOM   3760  HB2 LEU   269      16.067  61.512 -18.998  1.00  0.00           H
ATOM   3761  HB3 LEU   269      15.822  61.440 -20.714  1.00  0.00           H
ATOM   3762  HG  LEU   269      18.396  60.601 -19.322  1.00  0.00           H
ATOM   3763 HD11 LEU   269      17.653  58.280 -19.748  1.00  0.00           H
ATOM   3764 HD12 LEU   269      16.511  59.128 -18.712  1.00  0.00           H
ATOM   3765 HD13 LEU   269      16.130  58.876 -20.410  1.00  0.00           H
ATOM   3766 HD21 LEU   269      18.968  59.542 -21.513  1.00  0.00           H
ATOM   3767 HD22 LEU   269      17.518  60.241 -22.233  1.00  0.00           H
ATOM   3768 HD23 LEU   269      18.823  61.282 -21.671  1.00  0.00           H
ENDMDL
END
```

```
REMARK 888
REMARK 888 WRITTEN BY MAESTRO (A PRODUCT OF SCHRODINGER, LLC)
TITLE     hmod2
HELIX    1   1 LEU     21  VAL     47  1
HELIX    2   2 PHE     56  ALA     74  1
HELIX    3   3 VAL     86  LEU     93  1
HELIX    4   4 LEU    101  ASN    133  1
HELIX    5   5 MET    157  VAL    179  1
HELIX    6   6 GLU    191  SER    205  1
HELIX    7   7 GLY    207  SER    210  1
HELIX    8   8 PHE    220  LEU    228  1
HELIX    9   9 VAL    245  MET    264  1
TURN     1   1 ARG    20  ARG    20
TURN     2   2 LEU    48  GLY    55
TURN     3   3 GLY    75  ALA    85
TURN     4   4 ALA    94  LYS   100
TURN     5   5 GLN   134  ASP   156
TURN     6   6 ASP   180  LEU   190
TURN     7   7 MET   206  MET   206
TURN     8   8 GLY   211  ASP   219
TURN     9   9 ALA   229  TRP   244
```

```
TURN     10  10 ILE   265  LEU   269
SEQRES   1     250  ARG LEU LEU ARG GLN ALA LEU ALA GLU CYS LEU GLY THR
SEQRES   2     250  LEU ILE LEU VAL MET PHE GLY CYS GLY SER VAL ALA GLN
SEQRES   3     250  VAL VAL LEU SER ARG GLY THR HIS GLY GLY PHE LEU THR
SEQRES   4     250  ILE ASN LEU ALA PHE GLY PHE ALA VAL THR LEU GLY ILE
SEQRES   5     250  LEU ILE ALA GLY GLN VAL SER GLY ALA HIS LEU ASN PRO
SEQRES   6     250  ALA VAL THR PHE ALA MET CYS PHE LEU ALA ARG GLU PRO
SEQRES   7     250  TRP ILE LYS LEU PRO ILE TYR THR LEU ALA GLN THR LEU
SEQRES   8     250  GLY ALA PHE LEU GLY ALA GLY ILE VAL PHE GLY LEU TYR
SEQRES   9     250  TYR ASP ALA ILE TRP HIS PHE ALA ASP ASN GLN LEU PHE
SEQRES  10     250  VAL SER GLY PRO ASN GLY THR ALA GLY ILE PHE ALA THR
SEQRES  11     250  TYR PRO SER GLY HIS LEU ASP MET ILE ASN GLY PHE PHE
SEQRES  12     250  ASP GLN PHE ILE GLY THR ALA SER LEU ILE VAL CYS VAL
SEQRES  13     250  LEU ALA ILE VAL ASP PRO TYR ASN ASN PRO VAL PRO ARG
SEQRES  14     250  GLY LEU GLU ALA PHE THR VAL GLY LEU VAL VAL LEU VAL
SEQRES  15     250  ILE GLY THR SER MET GLY PHE ASN SER GLY TYR ALA VAL
SEQRES  16     250  ASN PRO ALA ARG ASP PHE GLY PRO ARG LEU PHE THR ALA
SEQRES  17     250  LEU ALA GLY TRP GLY SER ALA VAL PHE THR THR GLY GLN
SEQRES  18     250  HIS TRP TRP TRP VAL PRO ILE VAL SER PRO LEU LEU GLY
SEQRES  19     250  SER ILE ALA GLY VAL PHE VAL TYR GLN LEU MET ILE GLY
SEQRES  20     250  CYS HIS LEU
MODEL        1
ATOM      1  N   ARG    20      -6.792  77.233 -14.743  1.00  0.00           N1+
ATOM      2  CA  ARG    20      -6.753  78.642 -15.185  1.00  0.00           C
ATOM      3  C   ARG    20      -6.898  79.492 -13.915  1.00  0.00           C
ATOM      4  O   ARG    20      -7.948  79.382 -13.292  1.00  0.00           O
ATOM      5  CB  ARG    20      -7.856  78.935 -16.242  1.00  0.00           C
ATOM      6  CG  ARG    20      -7.818  80.352 -16.865  1.00  0.00           C
ATOM      7  CD  ARG    20      -8.840  80.541 -18.010  1.00  0.00           C
ATOM      8  NE  ARG    20      -8.704  81.855 -18.672  1.00  0.00           N
ATOM      9  CZ  ARG    20      -9.125  82.191 -19.906  1.00  0.00           C
ATOM     10  NH1 ARG    20      -9.748  81.306 -20.690  1.00  0.00           N
ATOM     11  NH2 ARG    20      -8.920  83.431 -20.359  1.00  0.00           N1+
ATOM     12  H1  ARG    20      -6.918  76.569 -15.489  1.00  0.00           H
ATOM     13  H2  ARG    20      -5.885  77.057 -14.309  1.00  0.00           H
ATOM     14  H3  ARG    20      -7.491  77.117 -14.013  1.00  0.00           H
ATOM     15  HA  ARG    20      -5.777  78.819 -15.642  1.00  0.00           H
ATOM     16  HB2 ARG    20      -7.763  78.208 -17.050  1.00  0.00           H
ATOM     17  HB3 ARG    20      -8.842  78.768 -15.803  1.00  0.00           H
ATOM     18  HG2 ARG    20      -8.004  81.098 -16.090  1.00  0.00           H
ATOM     19  HG3 ARG    20      -6.814  80.550 -17.247  1.00  0.00           H
ATOM     20  HD2 ARG    20      -8.689  79.758 -18.755  1.00  0.00           H
ATOM     21  HD3 ARG    20      -9.861  80.435 -17.637  1.00  0.00           H
ATOM     22  HE  ARG    20      -8.315  82.582 -18.084  1.00  0.00           H
ATOM     23 HH12 ARG    20      -9.950  80.367 -20.380  1.00  0.00           H
ATOM     24 HH11 ARG    20     -10.068  81.537 -21.624  1.00  0.00           H
ATOM     25 HH22 ARG    20      -9.226  83.730 -21.278  1.00  0.00           H
ATOM     26 HH21 ARG    20      -8.449  84.147 -19.820  1.00  0.00           H
ATOM     27  N   LEU    21      -5.861  80.266 -13.545  1.00  0.00           N
ATOM     28  CA  LEU    21      -5.475  80.841 -12.234  1.00  0.00           C
ATOM     29  C   LEU    21      -6.464  80.787 -11.074  1.00  0.00           C
ATOM     30  O   LEU    21      -6.174  80.181 -10.047  1.00  0.00           O
ATOM     31  CB  LEU    21      -4.792  82.208 -12.463  1.00  0.00           C
ATOM     32  CG  LEU    21      -4.050  82.820 -11.255  1.00  0.00           C
ATOM     33  CD1 LEU    21      -2.859  81.959 -10.794  1.00  0.00           C
ATOM     34  CD2 LEU    21      -3.575  84.240 -11.596  1.00  0.00           C
ATOM     35  H   LEU    21      -5.071  80.254 -14.174  1.00  0.00           H
ATOM     36  HA  LEU    21      -4.677  80.170 -11.916  1.00  0.00           H
ATOM     37  HB2 LEU    21      -4.075  82.123 -13.282  1.00  0.00           H
ATOM     38  HB3 LEU    21      -5.547  82.917 -12.804  1.00  0.00           H
ATOM     39  HG  LEU    21      -4.743  82.910 -10.417  1.00  0.00           H
ATOM     40 HD11 LEU    21      -2.301  82.461 -10.002  1.00  0.00           H
ATOM     41 HD12 LEU    21      -3.177  80.999 -10.389  1.00  0.00           H
```

ATOM	42	HD13	LEU	21	-2.153	81.774	-11.604	1.00	0.00	H
ATOM	43	HD21	LEU	21	-3.101	84.712	-10.737	1.00	0.00	H
ATOM	44	HD22	LEU	21	-2.844	84.238	-12.406	1.00	0.00	H
ATOM	45	HD23	LEU	21	-4.405	84.883	-11.893	1.00	0.00	H
ATOM	46	N	LEU	22	-7.634	81.396	-11.259	1.00	0.00	N
ATOM	47	CA	LEU	22	-8.665	81.472	-10.242	1.00	0.00	C
ATOM	48	C	LEU	22	-9.216	80.105	-9.836	1.00	0.00	C
ATOM	49	O	LEU	22	-9.215	79.776	-8.650	1.00	0.00	O
ATOM	50	CB	LEU	22	-9.816	82.380	-10.736	1.00	0.00	C
ATOM	51	CG	LEU	22	-9.410	83.824	-11.115	1.00	0.00	C
ATOM	52	CD1	LEU	22	-10.607	84.578	-11.717	1.00	0.00	C
ATOM	53	CD2	LEU	22	-8.836	84.608	-9.923	1.00	0.00	C
ATOM	54	H	LEU	22	-7.793	81.880	-12.125	1.00	0.00	H
ATOM	55	HA	LEU	22	-8.226	81.914	-9.344	1.00	0.00	H
ATOM	56	HB2	LEU	22	-10.301	81.912	-11.594	1.00	0.00	H
ATOM	57	HB3	LEU	22	-10.583	82.427	-9.960	1.00	0.00	H
ATOM	58	HG	LEU	22	-8.640	83.790	-11.886	1.00	0.00	H
ATOM	59	HD11	LEU	22	-10.326	85.586	-12.023	1.00	0.00	H
ATOM	60	HD12	LEU	22	-11.003	84.069	-12.595	1.00	0.00	H
ATOM	61	HD13	LEU	22	-11.420	84.673	-10.995	1.00	0.00	H
ATOM	62	HD21	LEU	22	-8.609	85.639	-10.199	1.00	0.00	H
ATOM	63	HD22	LEU	22	-9.541	84.644	-9.090	1.00	0.00	H
ATOM	64	HD23	LEU	22	-7.908	84.172	-9.552	1.00	0.00	H
ATOM	65	N	ARG	23	-9.550	79.255	-10.821	1.00	0.00	N
ATOM	66	CA	ARG	23	-9.677	77.835	-10.545	1.00	0.00	C
ATOM	67	C	ARG	23	-8.333	77.177	-10.886	1.00	0.00	C
ATOM	68	O	ARG	23	-8.153	76.541	-11.932	1.00	0.00	O
ATOM	69	CB	ARG	23	-10.864	77.123	-11.246	1.00	0.00	C
ATOM	70	CG	ARG	23	-11.083	75.735	-10.580	1.00	0.00	C
ATOM	71	CD	ARG	23	-12.211	74.841	-11.119	1.00	0.00	C
ATOM	72	NE	ARG	23	-12.361	73.659	-10.238	1.00	0.00	N
ATOM	73	CZ	ARG	23	-12.350	72.350	-10.548	1.00	0.00	C
ATOM	74	NH1	ARG	23	-12.241	71.936	-11.812	1.00	0.00	N
ATOM	75	NH2	ARG	23	-12.441	71.445	-9.570	1.00	0.00	N1+
ATOM	76	H	ARG	23	-9.333	79.539	-11.767	1.00	0.00	H
ATOM	77	HA	ARG	23	-9.849	77.669	-9.478	1.00	0.00	H
ATOM	78	HB2	ARG	23	-11.769	77.721	-11.126	1.00	0.00	H
ATOM	79	HB3	ARG	23	-10.693	77.023	-12.319	1.00	0.00	H
ATOM	80	HG2	ARG	23	-10.160	75.160	-10.635	1.00	0.00	H
ATOM	81	HG3	ARG	23	-11.256	75.893	-9.513	1.00	0.00	H
ATOM	82	HD2	ARG	23	-13.156	75.387	-11.129	1.00	0.00	H
ATOM	83	HD3	ARG	23	-11.999	74.548	-12.146	1.00	0.00	H
ATOM	84	HE	ARG	23	-12.493	73.922	-9.265	1.00	0.00	H
ATOM	85	HH12	ARG	23	-12.216	70.954	-12.062	1.00	0.00	H
ATOM	86	HH11	ARG	23	-12.205	72.593	-12.575	1.00	0.00	H
ATOM	87	HH22	ARG	23	-12.420	70.445	-9.742	1.00	0.00	H
ATOM	88	HH21	ARG	23	-12.506	71.702	-8.592	1.00	0.00	H
ATOM	89	N	GLN	24	-7.412	77.414	-9.957	1.00	0.00	N
ATOM	90	CA	GLN	24	-6.195	76.714	-9.579	1.00	0.00	C
ATOM	91	C	GLN	24	-5.986	77.053	-8.117	1.00	0.00	C
ATOM	92	O	GLN	24	-5.785	76.171	-7.292	1.00	0.00	O
ATOM	93	CB	GLN	24	-4.943	77.090	-10.403	1.00	0.00	C
ATOM	94	CG	GLN	24	-5.099	76.899	-11.911	1.00	0.00	C
ATOM	95	CD	GLN	24	-3.933	77.354	-12.776	1.00	0.00	C
ATOM	96	OE1	GLN	24	-4.162	77.607	-13.959	1.00	0.00	O
ATOM	97	NE2	GLN	24	-2.739	77.550	-12.242	1.00	0.00	N
ATOM	98	H	GLN	24	-7.721	78.051	-9.230	1.00	0.00	H
ATOM	99	HA	GLN	24	-6.372	75.640	-9.662	1.00	0.00	H
ATOM	100	HB2	GLN	24	-4.644	78.118	-10.194	1.00	0.00	H
ATOM	101	HB3	GLN	24	-4.126	76.471	-10.041	1.00	0.00	H
ATOM	102	HG2	GLN	24	-5.342	75.862	-12.139	1.00	0.00	H
ATOM	103	HG3	GLN	24	-5.934	77.512	-12.218	1.00	0.00	H
ATOM	104	HE22	GLN	24	-1.988	77.912	-12.805	1.00	0.00	H

ATOM	105	HE21	GLN	24	-2.524	77.190	-11.304	1.00	0.00	H
ATOM	106	N	ALA	25	-6.128	78.351	-7.818	1.00	0.00	N
ATOM	107	CA	ALA	25	-6.165	78.902	-6.482	1.00	0.00	C
ATOM	108	C	ALA	25	-7.218	78.244	-5.606	1.00	0.00	C
ATOM	109	O	ALA	25	-6.908	77.627	-4.592	1.00	0.00	O
ATOM	110	CB	ALA	25	-6.350	80.426	-6.551	1.00	0.00	C
ATOM	111	H	ALA	25	-6.183	79.019	-8.583	1.00	0.00	H
ATOM	112	HA	ALA	25	-5.194	78.705	-6.021	1.00	0.00	H
ATOM	113	HB1	ALA	25	-6.336	80.863	-5.551	1.00	0.00	H
ATOM	114	HB2	ALA	25	-5.542	80.894	-7.114	1.00	0.00	H
ATOM	115	HB3	ALA	25	-7.293	80.709	-7.019	1.00	0.00	H
ATOM	116	N	LEU	26	-8.455	78.234	-6.090	1.00	0.00	N
ATOM	117	CA	LEU	26	-9.519	77.444	-5.505	1.00	0.00	C
ATOM	118	C	LEU	26	-9.502	76.021	-6.106	1.00	0.00	C
ATOM	119	O	LEU	26	-10.420	75.559	-6.788	1.00	0.00	O
ATOM	120	CB	LEU	26	-10.866	78.134	-5.835	1.00	0.00	C
ATOM	121	CG	LEU	26	-11.017	79.577	-5.297	1.00	0.00	C
ATOM	122	CD1	LEU	26	-12.322	80.208	-5.805	1.00	0.00	C
ATOM	123	CD2	LEU	26	-10.953	79.643	-3.761	1.00	0.00	C
ATOM	124	H	LEU	26	-8.665	78.780	-6.916	1.00	0.00	H
ATOM	125	HA	LEU	26	-9.415	77.360	-4.422	1.00	0.00	H
ATOM	126	HB2	LEU	26	-11.008	78.149	-6.917	1.00	0.00	H
ATOM	127	HB3	LEU	26	-11.683	77.528	-5.438	1.00	0.00	H
ATOM	128	HG	LEU	26	-10.201	80.187	-5.687	1.00	0.00	H
ATOM	129	HD11	LEU	26	-12.420	81.239	-5.461	1.00	0.00	H
ATOM	130	HD12	LEU	26	-12.359	80.223	-6.894	1.00	0.00	H
ATOM	131	HD13	LEU	26	-13.194	79.660	-5.447	1.00	0.00	H
ATOM	132	HD21	LEU	26	-11.122	80.659	-3.402	1.00	0.00	H
ATOM	133	HD22	LEU	26	-11.707	79.005	-3.298	1.00	0.00	H
ATOM	134	HD23	LEU	26	-9.978	79.338	-3.380	1.00	0.00	H
ATOM	135	N	ALA	27	-8.387	75.352	-5.844	1.00	0.00	N
ATOM	136	CA	ALA	27	-8.109	73.930	-5.891	1.00	0.00	C
ATOM	137	C	ALA	27	-7.006	73.719	-4.867	1.00	0.00	C
ATOM	138	O	ALA	27	-7.162	72.887	-3.980	1.00	0.00	O
ATOM	139	CB	ALA	27	-7.822	73.423	-7.313	1.00	0.00	C
ATOM	140	H	ALA	27	-7.688	75.888	-5.338	1.00	0.00	H
ATOM	141	HA	ALA	27	-8.991	73.404	-5.519	1.00	0.00	H
ATOM	142	HB1	ALA	27	-7.523	72.373	-7.296	1.00	0.00	H
ATOM	143	HB2	ALA	27	-8.708	73.505	-7.942	1.00	0.00	H
ATOM	144	HB3	ALA	27	-7.025	73.983	-7.791	1.00	0.00	H
ATOM	145	N	GLU	28	-5.995	74.603	-4.886	1.00	0.00	N
ATOM	146	CA	GLU	28	-5.030	74.784	-3.809	1.00	0.00	C
ATOM	147	C	GLU	28	-5.658	75.028	-2.440	1.00	0.00	C
ATOM	148	O	GLU	28	-5.343	74.342	-1.469	1.00	0.00	O
ATOM	149	CB	GLU	28	-3.964	75.852	-4.143	1.00	0.00	C
ATOM	150	CG	GLU	28	-3.017	75.488	-5.306	1.00	0.00	C
ATOM	151	CD	GLU	28	-2.082	74.312	-5.013	1.00	0.00	C
ATOM	152	OE1	GLU	28	-1.291	74.415	-4.056	1.00	0.00	O
ATOM	153	OE2	GLU	28	-2.141	73.309	-5.758	1.00	0.00	O1-
ATOM	154	H	GLU	28	-5.883	75.203	-5.700	1.00	0.00	H
ATOM	155	HA	GLU	28	-4.511	73.828	-3.711	1.00	0.00	H
ATOM	156	HB2	GLU	28	-4.439	76.808	-4.355	1.00	0.00	H
ATOM	157	HB3	GLU	28	-3.351	76.027	-3.257	1.00	0.00	H
ATOM	158	HG2	GLU	28	-3.593	75.291	-6.203	1.00	0.00	H
ATOM	159	HG3	GLU	28	-2.386	76.347	-5.529	1.00	0.00	H
ATOM	160	N	CYS	29	-6.610	75.957	-2.392	1.00	0.00	N
ATOM	161	CA	CYS	29	-7.273	76.374	-1.169	1.00	0.00	C
ATOM	162	C	CYS	29	-8.192	75.315	-0.562	1.00	0.00	C
ATOM	163	O	CYS	29	-8.481	75.361	0.626	1.00	0.00	O
ATOM	164	CB	CYS	29	-8.081	77.659	-1.478	1.00	0.00	C
ATOM	165	SG	CYS	29	-8.383	78.679	-0.009	1.00	0.00	S
ATOM	166	H	CYS	29	-6.738	76.532	-3.220	1.00	0.00	H
ATOM	167	HA	CYS	29	-6.500	76.593	-0.432	1.00	0.00	H

ATOM	168	HB2	CYS	29	-7.539	78.299	-2.169	1.00	0.00	H
ATOM	169	HB3	CYS	29	-9.036	77.424	-1.950	1.00	0.00	H
ATOM	170	HG	CYS	29	-7.104	78.719	0.396	1.00	0.00	H
ATOM	171	N	LEU	30	-8.623	74.343	-1.365	1.00	0.00	N
ATOM	172	CA	LEU	30	-9.399	73.210	-0.890	1.00	0.00	C
ATOM	173	C	LEU	30	-8.478	72.057	-0.587	1.00	0.00	C
ATOM	174	O	LEU	30	-8.683	71.363	0.396	1.00	0.00	O
ATOM	175	CB	LEU	30	-10.348	72.761	-2.031	1.00	0.00	C
ATOM	176	CG	LEU	30	-11.296	73.858	-2.573	1.00	0.00	C
ATOM	177	CD1	LEU	30	-12.058	73.358	-3.809	1.00	0.00	C
ATOM	178	CD2	LEU	30	-12.276	74.362	-1.500	1.00	0.00	C
ATOM	179	H	LEU	30	-8.257	74.313	-2.302	1.00	0.00	H
ATOM	180	HA	LEU	30	-9.979	73.461	-0.007	1.00	0.00	H
ATOM	181	HB2	LEU	30	-9.760	72.370	-2.863	1.00	0.00	H
ATOM	182	HB3	LEU	30	-10.946	71.917	-1.679	1.00	0.00	H
ATOM	183	HG	LEU	30	-10.700	74.711	-2.899	1.00	0.00	H
ATOM	184	HD11	LEU	30	-12.698	74.139	-4.218	1.00	0.00	H
ATOM	185	HD12	LEU	30	-11.372	73.049	-4.598	1.00	0.00	H
ATOM	186	HD13	LEU	30	-12.691	72.504	-3.563	1.00	0.00	H
ATOM	187	HD21	LEU	30	-12.958	75.110	-1.904	1.00	0.00	H
ATOM	188	HD22	LEU	30	-12.880	73.548	-1.096	1.00	0.00	H
ATOM	189	HD23	LEU	30	-11.754	74.830	-0.665	1.00	0.00	H
ATOM	190	N	GLY	31	-7.428	71.891	-1.387	1.00	0.00	N
ATOM	191	CA	GLY	31	-6.489	70.817	-1.222	1.00	0.00	C
ATOM	192	C	GLY	31	-5.692	70.926	0.061	1.00	0.00	C
ATOM	193	O	GLY	31	-5.545	69.945	0.786	1.00	0.00	O
ATOM	194	H	GLY	31	-7.320	72.465	-2.217	1.00	0.00	H
ATOM	195	HA2	GLY	31	-7.014	69.860	-1.239	1.00	0.00	H
ATOM	196	HA3	GLY	31	-5.808	70.804	-2.071	1.00	0.00	H
ATOM	197	N	THR	32	-5.278	72.150	0.384	1.00	0.00	N
ATOM	198	CA	THR	32	-4.514	72.415	1.585	1.00	0.00	C
ATOM	199	C	THR	32	-5.387	72.457	2.837	1.00	0.00	C
ATOM	200	O	THR	32	-4.938	72.149	3.937	1.00	0.00	O
ATOM	201	CB	THR	32	-3.754	73.764	1.440	1.00	0.00	C
ATOM	202	OG1	THR	32	-3.190	73.897	0.145	1.00	0.00	O
ATOM	203	CG2	THR	32	-2.570	73.869	2.413	1.00	0.00	C
ATOM	204	H	THR	32	-5.434	72.910	-0.268	1.00	0.00	H
ATOM	205	HA	THR	32	-3.775	71.618	1.706	1.00	0.00	H
ATOM	206	HB	THR	32	-4.431	74.607	1.599	1.00	0.00	H
ATOM	207	HG1	THR	32	-3.896	74.015	-0.486	1.00	0.00	H
ATOM	208	HG21	THR	32	-2.011	74.793	2.271	1.00	0.00	H
ATOM	209	HG22	THR	32	-2.900	73.837	3.449	1.00	0.00	H
ATOM	210	HG23	THR	32	-1.873	73.043	2.275	1.00	0.00	H
ATOM	211	N	LEU	33	-6.661	72.772	2.644	1.00	0.00	N
ATOM	212	CA	LEU	33	-7.681	72.680	3.657	1.00	0.00	C
ATOM	213	C	LEU	33	-7.997	71.231	3.971	1.00	0.00	C
ATOM	214	O	LEU	33	-7.973	70.850	5.134	1.00	0.00	O
ATOM	215	CB	LEU	33	-8.953	73.395	3.071	1.00	0.00	C
ATOM	216	CG	LEU	33	-10.396	72.952	3.456	1.00	0.00	C
ATOM	217	CD1	LEU	33	-10.702	73.167	4.932	1.00	0.00	C
ATOM	218	CD2	LEU	33	-11.450	73.701	2.624	1.00	0.00	C
ATOM	219	H	LEU	33	-6.961	72.938	1.697	1.00	0.00	H
ATOM	220	HA	LEU	33	-7.363	73.174	4.576	1.00	0.00	H
ATOM	221	HB2	LEU	33	-8.865	74.465	3.246	1.00	0.00	H
ATOM	222	HB3	LEU	33	-8.918	73.301	1.993	1.00	0.00	H
ATOM	223	HG	LEU	33	-10.535	71.894	3.236	1.00	0.00	H
ATOM	224	HD11	LEU	33	-11.742	72.938	5.168	1.00	0.00	H
ATOM	225	HD12	LEU	33	-10.087	72.521	5.555	1.00	0.00	H
ATOM	226	HD13	LEU	33	-10.529	74.209	5.201	1.00	0.00	H
ATOM	227	HD21	LEU	33	-12.460	73.476	2.968	1.00	0.00	H
ATOM	228	HD22	LEU	33	-11.315	74.782	2.686	1.00	0.00	H
ATOM	229	HD23	LEU	33	-11.413	73.414	1.577	1.00	0.00	H
ATOM	230	N	ILE	34	-8.274	70.434	2.934	1.00	0.00	N

ATOM	231	CA	ILE	34	-8.584	69.021	3.031	1.00	0.00	C
ATOM	232	C	ILE	34	-7.493	68.238	3.767	1.00	0.00	C
ATOM	233	O	ILE	34	-7.760	67.432	4.658	1.00	0.00	O
ATOM	234	CB	ILE	34	-8.949	68.432	1.629	1.00	0.00	C
ATOM	235	CG1	ILE	34	-10.365	68.899	1.206	1.00	0.00	C
ATOM	236	CG2	ILE	34	-8.864	66.888	1.533	1.00	0.00	C
ATOM	237	CD1	ILE	34	-10.655	68.752	-0.295	1.00	0.00	C
ATOM	238	H	ILE	34	-8.319	70.844	2.003	1.00	0.00	H
ATOM	239	HA	ILE	34	-9.470	68.933	3.665	1.00	0.00	H
ATOM	240	HB	ILE	34	-8.224	68.821	0.913	1.00	0.00	H
ATOM	241	HG12	ILE	34	-11.118	68.357	1.780	1.00	0.00	H
ATOM	242	HG13	ILE	34	-10.507	69.946	1.474	1.00	0.00	H
ATOM	243	HG21	ILE	34	-9.155	66.523	0.548	1.00	0.00	H
ATOM	244	HG22	ILE	34	-7.851	66.520	1.695	1.00	0.00	H
ATOM	245	HG23	ILE	34	-9.513	66.409	2.267	1.00	0.00	H
ATOM	246	HD11	ILE	34	-11.621	69.190	-0.544	1.00	0.00	H
ATOM	247	HD12	ILE	34	-9.897	69.258	-0.893	1.00	0.00	H
ATOM	248	HD13	ILE	34	-10.686	67.707	-0.604	1.00	0.00	H
ATOM	249	N	LEU	35	-6.253	68.568	3.425	1.00	0.00	N
ATOM	250	CA	LEU	35	-5.044	68.116	4.066	1.00	0.00	C
ATOM	251	C	LEU	35	-5.021	68.381	5.578	1.00	0.00	C
ATOM	252	O	LEU	35	-4.831	67.469	6.386	1.00	0.00	O
ATOM	253	CB	LEU	35	-3.889	68.811	3.297	1.00	0.00	C
ATOM	254	CG	LEU	35	-2.423	68.466	3.621	1.00	0.00	C
ATOM	255	CD1	LEU	35	-1.545	68.841	2.410	1.00	0.00	C
ATOM	256	CD2	LEU	35	-1.867	69.166	4.873	1.00	0.00	C
ATOM	257	H	LEU	35	-6.151	69.214	2.650	1.00	0.00	H
ATOM	258	HA	LEU	35	-4.968	67.038	3.915	1.00	0.00	H
ATOM	259	HB2	LEU	35	-4.035	68.552	2.248	1.00	0.00	H
ATOM	260	HB3	LEU	35	-4.005	69.890	3.347	1.00	0.00	H
ATOM	261	HG	LEU	35	-2.369	67.390	3.772	1.00	0.00	H
ATOM	262	HD11	LEU	35	-0.486	68.671	2.600	1.00	0.00	H
ATOM	263	HD12	LEU	35	-1.812	68.260	1.526	1.00	0.00	H
ATOM	264	HD13	LEU	35	-1.655	69.896	2.153	1.00	0.00	H
ATOM	265	HD21	LEU	35	-0.808	68.942	4.999	1.00	0.00	H
ATOM	266	HD22	LEU	35	-1.964	70.249	4.799	1.00	0.00	H
ATOM	267	HD23	LEU	35	-2.358	68.841	5.789	1.00	0.00	H
ATOM	268	N	VAL	36	-5.242	69.642	5.952	1.00	0.00	N
ATOM	269	CA	VAL	36	-5.268	70.113	7.329	1.00	0.00	C
ATOM	270	C	VAL	36	-6.476	69.619	8.105	1.00	0.00	C
ATOM	271	O	VAL	36	-6.373	69.378	9.305	1.00	0.00	O
ATOM	272	CB	VAL	36	-5.159	71.667	7.297	1.00	0.00	C
ATOM	273	CG1	VAL	36	-5.496	72.390	8.614	1.00	0.00	C
ATOM	274	CG2	VAL	36	-3.738	72.078	6.869	1.00	0.00	C
ATOM	275	H	VAL	36	-5.412	70.330	5.229	1.00	0.00	H
ATOM	276	HA	VAL	36	-4.391	69.714	7.845	1.00	0.00	H
ATOM	277	HB	VAL	36	-5.863	72.044	6.553	1.00	0.00	H
ATOM	278	HG11	VAL	36	-5.369	73.466	8.516	1.00	0.00	H
ATOM	279	HG12	VAL	36	-6.525	72.225	8.935	1.00	0.00	H
ATOM	280	HG13	VAL	36	-4.836	72.062	9.412	1.00	0.00	H
ATOM	281	HG21	VAL	36	-3.660	73.154	6.750	1.00	0.00	H
ATOM	282	HG22	VAL	36	-3.006	71.775	7.615	1.00	0.00	H
ATOM	283	HG23	VAL	36	-3.445	71.629	5.922	1.00	0.00	H
ATOM	284	N	MET	37	-7.593	69.405	7.418	1.00	0.00	N
ATOM	285	CA	MET	37	-8.798	68.863	7.995	1.00	0.00	C
ATOM	286	C	MET	37	-8.549	67.486	8.617	1.00	0.00	C
ATOM	287	O	MET	37	-8.857	67.266	9.787	1.00	0.00	O
ATOM	288	CB	MET	37	-9.914	68.808	6.930	1.00	0.00	C
ATOM	289	CG	MET	37	-11.320	68.570	7.505	1.00	0.00	C
ATOM	290	SD	MET	37	-12.624	68.488	6.245	1.00	0.00	S
ATOM	291	CE	MET	37	-12.684	70.214	5.698	1.00	0.00	C
ATOM	292	H	MET	37	-7.619	69.704	6.445	1.00	0.00	H
ATOM	293	HA	MET	37	-9.110	69.547	8.784	1.00	0.00	H

ATOM	294	HB2	MET	37	-9.930	69.752	6.389	1.00	0.00	H
ATOM	295	HB3	MET	37	-9.702	68.036	6.192	1.00	0.00	H
ATOM	296	HG2	MET	37	-11.343	67.625	8.050	1.00	0.00	H
ATOM	297	HG3	MET	37	-11.575	69.347	8.228	1.00	0.00	H
ATOM	298	HE1	MET	37	-13.530	70.365	5.028	1.00	0.00	H
ATOM	299	HE2	MET	37	-12.795	70.884	6.551	1.00	0.00	H
ATOM	300	HE3	MET	37	-11.773	70.473	5.161	1.00	0.00	H
ATOM	301	N	PHE	38	-7.900	66.597	7.861	1.00	0.00	N
ATOM	302	CA	PHE	38	-7.598	65.250	8.324	1.00	0.00	C
ATOM	303	C	PHE	38	-6.396	65.155	9.257	1.00	0.00	C
ATOM	304	O	PHE	38	-6.277	64.203	10.030	1.00	0.00	O
ATOM	305	CB	PHE	38	-7.473	64.317	7.116	1.00	0.00	C
ATOM	306	CG	PHE	38	-8.794	63.982	6.441	1.00	0.00	C
ATOM	307	CD1	PHE	38	-9.058	64.427	5.128	1.00	0.00	C
ATOM	308	CD2	PHE	38	-9.766	63.222	7.125	1.00	0.00	C
ATOM	309	CE1	PHE	38	-10.292	64.137	4.516	1.00	0.00	C
ATOM	310	CE2	PHE	38	-10.998	62.928	6.510	1.00	0.00	C
ATOM	311	CZ	PHE	38	-11.264	63.388	5.207	1.00	0.00	C
ATOM	312	H	PHE	38	-7.691	66.841	6.899	1.00	0.00	H
ATOM	313	HA	PHE	38	-8.441	64.894	8.920	1.00	0.00	H
ATOM	314	HB2	PHE	38	-6.831	64.797	6.389	1.00	0.00	H
ATOM	315	HB3	PHE	38	-7.009	63.373	7.408	1.00	0.00	H
ATOM	316	HD1	PHE	38	-8.320	64.996	4.581	1.00	0.00	H
ATOM	317	HD2	PHE	38	-9.576	62.864	8.127	1.00	0.00	H
ATOM	318	HE1	PHE	38	-10.494	64.489	3.515	1.00	0.00	H
ATOM	319	HE2	PHE	38	-11.742	62.350	7.040	1.00	0.00	H
ATOM	320	HZ	PHE	38	-12.211	63.166	4.737	1.00	0.00	H
ATOM	321	N	GLY	39	-5.542	66.172	9.247	1.00	0.00	N
ATOM	322	CA	GLY	39	-4.276	66.130	9.939	1.00	0.00	C
ATOM	323	C	GLY	39	-4.069	67.182	11.031	1.00	0.00	C
ATOM	324	O	GLY	39	-2.995	67.237	11.632	1.00	0.00	O
ATOM	325	H	GLY	39	-5.690	66.899	8.556	1.00	0.00	H
ATOM	326	HA2	GLY	39	-4.028	65.148	10.345	1.00	0.00	H
ATOM	327	HA3	GLY	39	-3.535	66.325	9.167	1.00	0.00	H
ATOM	328	N	CYS	40	-5.076	68.012	11.303	1.00	0.00	N
ATOM	329	CA	CYS	40	-5.336	68.588	12.622	1.00	0.00	C
ATOM	330	C	CYS	40	-6.442	67.800	13.308	1.00	0.00	C
ATOM	331	O	CYS	40	-6.585	67.862	14.524	1.00	0.00	O
ATOM	332	CB	CYS	40	-5.803	70.052	12.493	1.00	0.00	C
ATOM	333	SG	CYS	40	-4.419	71.105	11.969	1.00	0.00	S
ATOM	334	H	CYS	40	-5.790	68.156	10.595	1.00	0.00	H
ATOM	335	HA	CYS	40	-4.452	68.545	13.263	1.00	0.00	H
ATOM	336	HB2	CYS	40	-6.636	70.154	11.796	1.00	0.00	H
ATOM	337	HB3	CYS	40	-6.151	70.427	13.458	1.00	0.00	H
ATOM	338	HG	CYS	40	-5.121	72.241	11.958	1.00	0.00	H
ATOM	339	N	GLY	41	-7.204	67.015	12.546	1.00	0.00	N
ATOM	340	CA	GLY	41	-8.445	66.469	13.036	1.00	0.00	C
ATOM	341	C	GLY	41	-8.290	65.109	13.697	1.00	0.00	C
ATOM	342	O	GLY	41	-9.044	64.773	14.607	1.00	0.00	O
ATOM	343	H	GLY	41	-7.060	66.999	11.547	1.00	0.00	H
ATOM	344	HA2	GLY	41	-8.936	67.150	13.734	1.00	0.00	H
ATOM	345	HA3	GLY	41	-9.135	66.354	12.200	1.00	0.00	H
ATOM	346	N	SER	42	-7.245	64.382	13.313	1.00	0.00	N
ATOM	347	CA	SER	42	-6.733	63.232	14.035	1.00	0.00	C
ATOM	348	C	SER	42	-6.002	63.651	15.304	1.00	0.00	C
ATOM	349	O	SER	42	-6.130	63.010	16.342	1.00	0.00	O
ATOM	350	CB	SER	42	-5.783	62.483	13.070	1.00	0.00	C
ATOM	351	OG	SER	42	-4.956	63.377	12.328	1.00	0.00	O
ATOM	352	H	SER	42	-6.669	64.714	12.555	1.00	0.00	H
ATOM	353	HA	SER	42	-7.553	62.568	14.322	1.00	0.00	H
ATOM	354	HB2	SER	42	-5.155	61.779	13.620	1.00	0.00	H
ATOM	355	HB3	SER	42	-6.363	61.884	12.366	1.00	0.00	H
ATOM	356	HG	SER	42	-5.364	63.511	11.470	1.00	0.00	H

ATOM	357	N	VAL	43	-5.277	64.767	15.236	1.00	0.00	N
ATOM	358	CA	VAL	43	-4.504	65.259	16.356	1.00	0.00	C
ATOM	359	C	VAL	43	-5.408	65.817	17.456	1.00	0.00	C
ATOM	360	O	VAL	43	-5.150	65.600	18.635	1.00	0.00	O
ATOM	361	CB	VAL	43	-3.450	66.300	15.885	1.00	0.00	C
ATOM	362	CG1	VAL	43	-2.355	66.497	16.953	1.00	0.00	C
ATOM	363	CG2	VAL	43	-2.765	65.902	14.561	1.00	0.00	C
ATOM	364	H	VAL	43	-5.175	65.214	14.340	1.00	0.00	H
ATOM	365	HA	VAL	43	-3.962	64.408	16.776	1.00	0.00	H
ATOM	366	HB	VAL	43	-3.945	67.260	15.721	1.00	0.00	H
ATOM	367	HG11	VAL	43	-1.619	67.238	16.638	1.00	0.00	H
ATOM	368	HG12	VAL	43	-2.765	66.828	17.908	1.00	0.00	H
ATOM	369	HG13	VAL	43	-1.815	65.568	17.141	1.00	0.00	H
ATOM	370	HG21	VAL	43	-2.016	66.639	14.270	1.00	0.00	H
ATOM	371	HG22	VAL	43	-2.262	64.939	14.648	1.00	0.00	H
ATOM	372	HG23	VAL	43	-3.471	65.828	13.736	1.00	0.00	H
ATOM	373	N	ALA	44	-6.524	66.438	17.073	1.00	0.00	N
ATOM	374	CA	ALA	44	-7.546	66.874	18.004	1.00	0.00	C
ATOM	375	C	ALA	44	-8.220	65.715	18.731	1.00	0.00	C
ATOM	376	O	ALA	44	-8.472	65.793	19.930	1.00	0.00	O
ATOM	377	CB	ALA	44	-8.612	67.667	17.232	1.00	0.00	C
ATOM	378	H	ALA	44	-6.650	66.645	16.089	1.00	0.00	H
ATOM	379	HA	ALA	44	-7.089	67.521	18.754	1.00	0.00	H
ATOM	380	HB1	ALA	44	-9.388	68.035	17.905	1.00	0.00	H
ATOM	381	HB2	ALA	44	-8.187	68.532	16.726	1.00	0.00	H
ATOM	382	HB3	ALA	44	-9.103	67.051	16.477	1.00	0.00	H
ATOM	383	N	GLN	45	-8.434	64.613	18.014	1.00	0.00	N
ATOM	384	CA	GLN	45	-8.995	63.398	18.569	1.00	0.00	C
ATOM	385	C	GLN	45	-8.058	62.739	19.582	1.00	0.00	C
ATOM	386	O	GLN	45	-8.510	62.162	20.569	1.00	0.00	O
ATOM	387	CB	GLN	45	-9.263	62.414	17.410	1.00	0.00	C
ATOM	388	CG	GLN	45	-10.176	61.227	17.767	1.00	0.00	C
ATOM	389	CD	GLN	45	-10.490	60.362	16.546	1.00	0.00	C
ATOM	390	OE1	GLN	45	-9.727	60.306	15.587	1.00	0.00	O
ATOM	391	NE2	GLN	45	-11.628	59.680	16.555	1.00	0.00	N
ATOM	392	H	GLN	45	-8.185	64.617	17.036	1.00	0.00	H
ATOM	393	HA	GLN	45	-9.936	63.647	19.065	1.00	0.00	H
ATOM	394	HB2	GLN	45	-9.706	62.946	16.573	1.00	0.00	H
ATOM	395	HB3	GLN	45	-8.321	62.019	17.029	1.00	0.00	H
ATOM	396	HG2	GLN	45	-9.709	60.595	18.524	1.00	0.00	H
ATOM	397	HG3	GLN	45	-11.107	61.601	18.193	1.00	0.00	H
ATOM	398	HE22	GLN	45	-11.849	59.119	15.748	1.00	0.00	H
ATOM	399	HE21	GLN	45	-12.256	59.738	17.341	1.00	0.00	H
ATOM	400	N	VAL	46	-6.753	62.838	19.327	1.00	0.00	N
ATOM	401	CA	VAL	46	-5.715	62.344	20.207	1.00	0.00	C
ATOM	402	C	VAL	46	-5.558	63.214	21.455	1.00	0.00	C
ATOM	403	O	VAL	46	-5.372	62.674	22.543	1.00	0.00	O
ATOM	404	CB	VAL	46	-4.410	62.157	19.388	1.00	0.00	C
ATOM	405	CG1	VAL	46	-3.141	61.916	20.230	1.00	0.00	C
ATOM	406	CG2	VAL	46	-4.593	60.978	18.413	1.00	0.00	C
ATOM	407	H	VAL	46	-6.473	63.268	18.455	1.00	0.00	H
ATOM	408	HA	VAL	46	-6.010	61.363	20.576	1.00	0.00	H
ATOM	409	HB	VAL	46	-4.239	63.061	18.803	1.00	0.00	H
ATOM	410	HG11	VAL	46	-2.281	61.705	19.594	1.00	0.00	H
ATOM	411	HG12	VAL	46	-2.883	62.787	20.834	1.00	0.00	H
ATOM	412	HG13	VAL	46	-3.273	61.071	20.907	1.00	0.00	H
ATOM	413	HG21	VAL	46	-3.793	60.950	17.683	1.00	0.00	H
ATOM	414	HG22	VAL	46	-4.597	60.023	18.941	1.00	0.00	H
ATOM	415	HG23	VAL	46	-5.525	61.035	17.853	1.00	0.00	H
ATOM	416	N	VAL	47	-5.642	64.539	21.309	1.00	0.00	N
ATOM	417	CA	VAL	47	-5.393	65.443	22.417	1.00	0.00	C
ATOM	418	C	VAL	47	-6.613	65.599	23.337	1.00	0.00	C
ATOM	419	O	VAL	47	-6.454	65.590	24.555	1.00	0.00	O

ATOM	420	CB	VAL	47	-4.855	66.802	21.875	1.00	0.00	C
ATOM	421	CG1	VAL	47	-4.658	67.887	22.957	1.00	0.00	C
ATOM	422	CG2	VAL	47	-3.489	66.612	21.177	1.00	0.00	C
ATOM	423	H	VAL	47	-5.735	64.922	20.375	1.00	0.00	H
ATOM	424	HA	VAL	47	-4.602	65.023	23.043	1.00	0.00	H
ATOM	425	HB	VAL	47	-5.570	67.181	21.144	1.00	0.00	H
ATOM	426	HG11	VAL	47	-4.283	68.814	22.523	1.00	0.00	H
ATOM	427	HG12	VAL	47	-5.578	68.130	23.482	1.00	0.00	H
ATOM	428	HG13	VAL	47	-3.942	67.561	23.713	1.00	0.00	H
ATOM	429	HG21	VAL	47	-3.164	67.527	20.681	1.00	0.00	H
ATOM	430	HG22	VAL	47	-2.718	66.341	21.898	1.00	0.00	H
ATOM	431	HG23	VAL	47	-3.498	65.824	20.428	1.00	0.00	H
ATOM	432	N	LEU	48	-7.811	65.773	22.776	1.00	0.00	N
ATOM	433	CA	LEU	48	-8.946	66.294	23.533	1.00	0.00	C
ATOM	434	C	LEU	48	-9.812	65.184	24.141	1.00	0.00	C
ATOM	435	O	LEU	48	-10.374	65.353	25.223	1.00	0.00	O
ATOM	436	CB	LEU	48	-9.817	67.095	22.553	1.00	0.00	C
ATOM	437	CG	LEU	48	-9.078	68.274	21.886	1.00	0.00	C
ATOM	438	CD1	LEU	48	-10.010	68.973	20.894	1.00	0.00	C
ATOM	439	CD2	LEU	48	-8.493	69.228	22.938	1.00	0.00	C
ATOM	440	H	LEU	48	-7.891	65.738	21.765	1.00	0.00	H
ATOM	441	HA	LEU	48	-8.617	66.935	24.355	1.00	0.00	H
ATOM	442	HB2	LEU	48	-10.224	66.434	21.786	1.00	0.00	H
ATOM	443	HB3	LEU	48	-10.679	67.478	23.092	1.00	0.00	H
ATOM	444	HG	LEU	48	-8.238	67.899	21.304	1.00	0.00	H
ATOM	445	HD11	LEU	48	-9.485	69.742	20.331	1.00	0.00	H
ATOM	446	HD12	LEU	48	-10.406	68.259	20.171	1.00	0.00	H
ATOM	447	HD13	LEU	48	-10.857	69.430	21.402	1.00	0.00	H
ATOM	448	HD21	LEU	48	-8.176	70.178	22.517	1.00	0.00	H
ATOM	449	HD22	LEU	48	-9.206	69.409	23.739	1.00	0.00	H
ATOM	450	HD23	LEU	48	-7.623	68.793	23.420	1.00	0.00	H
ATOM	451	N	SER	49	-9.907	64.028	23.475	1.00	0.00	N
ATOM	452	CA	SER	49	-10.599	62.856	23.996	1.00	0.00	C
ATOM	453	C	SER	49	-9.591	62.122	24.896	1.00	0.00	C
ATOM	454	O	SER	49	-8.873	61.220	24.466	1.00	0.00	O
ATOM	455	CB	SER	49	-11.108	62.014	22.803	1.00	0.00	C
ATOM	456	OG	SER	49	-11.639	60.751	23.153	1.00	0.00	O
ATOM	457	H	SER	49	-9.320	63.910	22.662	1.00	0.00	H
ATOM	458	HA	SER	49	-11.465	63.145	24.595	1.00	0.00	H
ATOM	459	HB2	SER	49	-11.887	62.571	22.282	1.00	0.00	H
ATOM	460	HB3	SER	49	-10.318	61.851	22.082	1.00	0.00	H
ATOM	461	HG	SER	49	-10.907	60.145	23.234	1.00	0.00	H
ATOM	462	N	ARG	50	-9.511	62.574	26.154	1.00	0.00	N
ATOM	463	CA	ARG	50	-8.672	62.046	27.225	1.00	0.00	C
ATOM	464	C	ARG	50	-9.008	60.565	27.481	1.00	0.00	C
ATOM	465	O	ARG	50	-9.980	60.264	28.171	1.00	0.00	O
ATOM	466	CB	ARG	50	-8.888	62.931	28.478	1.00	0.00	C
ATOM	467	CG	ARG	50	-8.081	62.512	29.724	1.00	0.00	C
ATOM	468	CD	ARG	50	-8.176	63.528	30.874	1.00	0.00	C
ATOM	469	NE	ARG	50	-9.534	63.601	31.444	1.00	0.00	N
ATOM	470	CZ	ARG	50	-9.921	64.421	32.436	1.00	0.00	C
ATOM	471	NH1	ARG	50	-9.036	65.221	33.037	1.00	0.00	N
ATOM	472	NH2	ARG	50	-11.204	64.435	32.807	1.00	0.00	N1+
ATOM	473	H	ARG	50	-10.068	63.395	26.352	1.00	0.00	H
ATOM	474	HA	ARG	50	-7.630	62.129	26.909	1.00	0.00	H
ATOM	475	HB2	ARG	50	-8.628	63.957	28.221	1.00	0.00	H
ATOM	476	HB3	ARG	50	-9.949	62.947	28.728	1.00	0.00	H
ATOM	477	HG2	ARG	50	-8.397	61.529	30.077	1.00	0.00	H
ATOM	478	HG3	ARG	50	-7.031	62.404	29.448	1.00	0.00	H
ATOM	479	HD2	ARG	50	-7.481	63.245	31.666	1.00	0.00	H
ATOM	480	HD3	ARG	50	-7.878	64.517	30.522	1.00	0.00	H
ATOM	481	HE	ARG	50	-10.198	62.975	31.007	1.00	0.00	H
ATOM	482	HH12	ARG	50	-9.263	65.930	33.737	1.00	0.00	H

ATOM	483	HH11	ARG	50	-8.042	65.222	32.771	1.00	0.00	H
ATOM	484	HH22	ARG	50	-11.590	65.127	33.459	1.00	0.00	H
ATOM	485	HH21	ARG	50	-11.901	63.841	32.385	1.00	0.00	H
ATOM	486	N	GLY	51	-8.199	59.662	26.922	1.00	0.00	N
ATOM	487	CA	GLY	51	-8.469	58.231	26.842	1.00	0.00	C
ATOM	488	C	GLY	51	-8.144	57.655	25.459	1.00	0.00	C
ATOM	489	O	GLY	51	-7.904	56.452	25.363	1.00	0.00	O
ATOM	490	H	GLY	51	-7.458	60.004	26.324	1.00	0.00	H
ATOM	491	HA2	GLY	51	-7.872	57.714	27.594	1.00	0.00	H
ATOM	492	HA3	GLY	51	-9.515	58.007	27.058	1.00	0.00	H
ATOM	493	N	THR	52	-8.044	58.486	24.409	1.00	0.00	N
ATOM	494	CA	THR	52	-7.471	58.103	23.117	1.00	0.00	C
ATOM	495	C	THR	52	-5.956	57.870	23.302	1.00	0.00	C
ATOM	496	O	THR	52	-5.295	58.592	24.052	1.00	0.00	O
ATOM	497	CB	THR	52	-7.706	59.240	22.088	1.00	0.00	C
ATOM	498	OG1	THR	52	-9.087	59.529	21.977	1.00	0.00	O
ATOM	499	CG2	THR	52	-7.249	58.887	20.661	1.00	0.00	C
ATOM	500	H	THR	52	-8.315	59.462	24.495	1.00	0.00	H
ATOM	501	HA	THR	52	-7.953	57.185	22.774	1.00	0.00	H
ATOM	502	HB	THR	52	-7.197	60.149	22.420	1.00	0.00	H
ATOM	503	HG1	THR	52	-9.140	60.372	21.535	1.00	0.00	H
ATOM	504	HG21	THR	52	-6.167	58.774	20.602	1.00	0.00	H
ATOM	505	HG22	THR	52	-7.523	59.663	19.946	1.00	0.00	H
ATOM	506	HG23	THR	52	-7.700	57.956	20.318	1.00	0.00	H
ATOM	507	N	HIE	53	-5.409	56.849	22.633	1.00	0.00	N
ATOM	508	CA	HIE	53	-3.983	56.540	22.635	1.00	0.00	C
ATOM	509	C	HIE	53	-3.352	57.263	21.438	1.00	0.00	C
ATOM	510	O	HIE	53	-3.924	57.272	20.347	1.00	0.00	O
ATOM	511	CB	HIE	53	-3.776	55.023	22.431	1.00	0.00	C
ATOM	512	CG	HIE	53	-4.251	54.124	23.545	1.00	0.00	C
ATOM	513	ND1	HIE	53	-3.936	52.763	23.573	1.00	0.00	N
ATOM	514	CD2	HIE	53	-5.036	54.410	24.644	1.00	0.00	C
ATOM	515	CE1	HIE	53	-4.531	52.285	24.657	1.00	0.00	C
ATOM	516	NE2	HIE	53	-5.199	53.219	25.332	1.00	0.00	N
ATOM	517	H	HIE	53	-5.980	56.354	21.966	1.00	0.00	H
ATOM	518	HA	HIE	53	-3.509	56.857	23.567	1.00	0.00	H
ATOM	519	HB2	HIE	53	-4.252	54.690	21.507	1.00	0.00	H
ATOM	520	HB3	HIE	53	-2.711	54.821	22.305	1.00	0.00	H
ATOM	521	HD2	HIE	53	-5.494	55.335	24.970	1.00	0.00	H
ATOM	522	HE1	HIE	53	-4.481	51.247	24.954	1.00	0.00	H
ATOM	523	HE2	HIE	53	-5.745	53.078	26.170	1.00	0.00	H
ATOM	524	N	GLY	54	-2.169	57.844	21.636	1.00	0.00	N
ATOM	525	CA	GLY	54	-1.393	58.515	20.606	1.00	0.00	C
ATOM	526	C	GLY	54	-0.062	57.788	20.465	1.00	0.00	C
ATOM	527	O	GLY	54	0.812	57.943	21.313	1.00	0.00	O
ATOM	528	H	GLY	54	-1.737	57.770	22.545	1.00	0.00	H
ATOM	529	HA2	GLY	54	-1.911	58.570	19.646	1.00	0.00	H
ATOM	530	HA3	GLY	54	-1.203	59.542	20.919	1.00	0.00	H
ATOM	531	N	GLY	55	0.085	56.987	19.408	1.00	0.00	N
ATOM	532	CA	GLY	55	1.314	56.300	19.042	1.00	0.00	C
ATOM	533	C	GLY	55	1.676	56.739	17.628	1.00	0.00	C
ATOM	534	O	GLY	55	0.824	56.638	16.744	1.00	0.00	O
ATOM	535	H	GLY	55	-0.673	56.918	18.743	1.00	0.00	H
ATOM	536	HA2	GLY	55	2.131	56.502	19.738	1.00	0.00	H
ATOM	537	HA3	GLY	55	1.139	55.224	19.052	1.00	0.00	H
ATOM	538	N	PHE	56	2.913	57.231	17.441	1.00	0.00	N
ATOM	539	CA	PHE	56	3.476	57.910	16.264	1.00	0.00	C
ATOM	540	C	PHE	56	2.854	57.546	14.924	1.00	0.00	C
ATOM	541	O	PHE	56	2.067	58.308	14.375	1.00	0.00	O
ATOM	542	CB	PHE	56	5.019	57.875	16.289	1.00	0.00	C
ATOM	543	CG	PHE	56	5.641	58.586	17.476	1.00	0.00	C
ATOM	544	CD1	PHE	56	6.200	57.845	18.539	1.00	0.00	C
ATOM	545	CD2	PHE	56	5.668	59.997	17.517	1.00	0.00	C

ATOM	546	CE1	PHE	56	6.778	58.512	19.636	1.00	0.00	C
ATOM	547	CE2	PHE	56	6.245	60.661	18.616	1.00	0.00	C
ATOM	548	CZ	PHE	56	6.800	59.919	19.676	1.00	0.00	C
ATOM	549	H	PHE	56	3.488	57.299	18.269	1.00	0.00	H
ATOM	550	HA	PHE	56	3.199	58.957	16.402	1.00	0.00	H
ATOM	551	HB2	PHE	56	5.373	56.843	16.263	1.00	0.00	H
ATOM	552	HB3	PHE	56	5.419	58.340	15.388	1.00	0.00	H
ATOM	553	HD1	PHE	56	6.201	56.765	18.519	1.00	0.00	H
ATOM	554	HD2	PHE	56	5.251	60.582	16.708	1.00	0.00	H
ATOM	555	HE1	PHE	56	7.214	57.947	20.448	1.00	0.00	H
ATOM	556	HE2	PHE	56	6.265	61.742	18.644	1.00	0.00	H
ATOM	557	HZ	PHE	56	7.249	60.427	20.517	1.00	0.00	H
ATOM	558	N	LEU	57	3.209	56.367	14.418	1.00	0.00	N
ATOM	559	CA	LEU	57	2.921	55.844	13.089	1.00	0.00	C
ATOM	560	C	LEU	57	1.531	56.127	12.519	1.00	0.00	C
ATOM	561	O	LEU	57	1.389	56.479	11.351	1.00	0.00	O
ATOM	562	CB	LEU	57	3.226	54.320	13.144	1.00	0.00	C
ATOM	563	CG	LEU	57	3.023	53.488	11.853	1.00	0.00	C
ATOM	564	CD1	LEU	57	4.176	53.679	10.862	1.00	0.00	C
ATOM	565	CD2	LEU	57	2.863	51.996	12.185	1.00	0.00	C
ATOM	566	H	LEU	57	3.791	55.792	15.005	1.00	0.00	H
ATOM	567	HA	LEU	57	3.631	56.310	12.409	1.00	0.00	H
ATOM	568	HB2	LEU	57	4.246	54.164	13.498	1.00	0.00	H
ATOM	569	HB3	LEU	57	2.584	53.893	13.918	1.00	0.00	H
ATOM	570	HG	LEU	57	2.102	53.790	11.354	1.00	0.00	H
ATOM	571	HD11	LEU	57	3.972	53.171	9.919	1.00	0.00	H
ATOM	572	HD12	LEU	57	4.334	54.733	10.640	1.00	0.00	H
ATOM	573	HD13	LEU	57	5.109	53.275	11.253	1.00	0.00	H
ATOM	574	HD21	LEU	57	2.707	51.406	11.280	1.00	0.00	H
ATOM	575	HD22	LEU	57	3.745	51.601	12.689	1.00	0.00	H
ATOM	576	HD23	LEU	57	2.004	51.824	12.834	1.00	0.00	H
ATOM	577	N	THR	58	0.507	55.975	13.352	1.00	0.00	N
ATOM	578	CA	THR	58	-0.882	56.103	12.961	1.00	0.00	C
ATOM	579	C	THR	58	-1.312	57.559	12.827	1.00	0.00	C
ATOM	580	O	THR	58	-2.169	57.866	11.998	1.00	0.00	O
ATOM	581	CB	THR	58	-1.710	55.411	14.079	1.00	0.00	C
ATOM	582	OG1	THR	58	-1.191	54.107	14.308	1.00	0.00	O
ATOM	583	CG2	THR	58	-3.205	55.247	13.761	1.00	0.00	C
ATOM	584	H	THR	58	0.678	55.684	14.306	1.00	0.00	H
ATOM	585	HA	THR	58	-1.045	55.595	12.008	1.00	0.00	H
ATOM	586	HB	THR	58	-1.610	55.971	15.012	1.00	0.00	H
ATOM	587	HG1	THR	58	-1.332	53.582	13.534	1.00	0.00	H
ATOM	588	HG21	THR	58	-3.719	54.709	14.559	1.00	0.00	H
ATOM	589	HG22	THR	58	-3.701	56.213	13.660	1.00	0.00	H
ATOM	590	HG23	THR	58	-3.364	54.700	12.831	1.00	0.00	H
ATOM	591	N	ILE	59	-0.651	58.459	13.563	1.00	0.00	N
ATOM	592	CA	ILE	59	-0.740	59.890	13.335	1.00	0.00	C
ATOM	593	C	ILE	59	-0.182	60.213	11.940	1.00	0.00	C
ATOM	594	O	ILE	59	-0.745	60.981	11.160	1.00	0.00	O
ATOM	595	CB	ILE	59	0.011	60.700	14.438	1.00	0.00	C
ATOM	596	CG1	ILE	59	-0.180	60.165	15.878	1.00	0.00	C
ATOM	597	CG2	ILE	59	-0.314	62.208	14.373	1.00	0.00	C
ATOM	598	CD1	ILE	59	-1.627	60.181	16.376	1.00	0.00	C
ATOM	599	H	ILE	59	0.110	58.141	14.155	1.00	0.00	H
ATOM	600	HA	ILE	59	-1.796	60.169	13.349	1.00	0.00	H
ATOM	601	HB	ILE	59	1.081	60.626	14.239	1.00	0.00	H
ATOM	602	HG12	ILE	59	0.205	59.150	15.963	1.00	0.00	H
ATOM	603	HG13	ILE	59	0.433	60.754	16.561	1.00	0.00	H
ATOM	604	HG21	ILE	59	0.224	62.763	15.142	1.00	0.00	H
ATOM	605	HG22	ILE	59	-0.036	62.643	13.414	1.00	0.00	H
ATOM	606	HG23	ILE	59	-1.379	62.398	14.508	1.00	0.00	H
ATOM	607	HD11	ILE	59	-1.686	59.799	17.395	1.00	0.00	H
ATOM	608	HD12	ILE	59	-2.024	61.196	16.379	1.00	0.00	H

ATOM	609	HD13	ILE	59	-2.277	59.564	15.755	1.00	0.00	H
ATOM	610	N	ASN	60	0.932	59.549	11.642	1.00	0.00	N
ATOM	611	CA	ASN	60	1.835	59.890	10.557	1.00	0.00	C
ATOM	612	C	ASN	60	1.309	59.395	9.223	1.00	0.00	C
ATOM	613	O	ASN	60	1.281	60.126	8.233	1.00	0.00	O
ATOM	614	CB	ASN	60	3.238	59.433	10.886	1.00	0.00	C
ATOM	615	CG	ASN	60	3.660	60.020	12.232	1.00	0.00	C
ATOM	616	OD1	ASN	60	3.262	61.103	12.640	1.00	0.00	O
ATOM	617	ND2	ASN	60	4.418	59.270	12.992	1.00	0.00	N
ATOM	618	H	ASN	60	1.288	58.948	12.374	1.00	0.00	H
ATOM	619	HA	ASN	60	1.860	60.976	10.459	1.00	0.00	H
ATOM	620	HB2	ASN	60	3.273	58.346	10.904	1.00	0.00	H
ATOM	621	HB3	ASN	60	3.926	59.785	10.121	1.00	0.00	H
ATOM	622	HD22	ASN	60	4.840	59.737	13.780	1.00	0.00	H
ATOM	623	HD21	ASN	60	4.779	58.408	12.605	1.00	0.00	H
ATOM	624	N	LEU	61	0.732	58.200	9.252	1.00	0.00	N
ATOM	625	CA	LEU	61	-0.082	57.624	8.208	1.00	0.00	C
ATOM	626	C	LEU	61	-1.275	58.481	7.827	1.00	0.00	C
ATOM	627	O	LEU	61	-1.553	58.672	6.644	1.00	0.00	O
ATOM	628	CB	LEU	61	-0.618	56.260	8.752	1.00	0.00	C
ATOM	629	CG	LEU	61	-1.598	55.455	7.860	1.00	0.00	C
ATOM	630	CD1	LEU	61	-0.967	55.014	6.532	1.00	0.00	C
ATOM	631	CD2	LEU	61	-2.137	54.237	8.625	1.00	0.00	C
ATOM	632	H	LEU	61	0.842	57.665	10.111	1.00	0.00	H
ATOM	633	HA	LEU	61	0.499	57.430	7.320	1.00	0.00	H
ATOM	634	HB2	LEU	61	0.203	55.603	9.028	1.00	0.00	H
ATOM	635	HB3	LEU	61	-1.129	56.456	9.697	1.00	0.00	H
ATOM	636	HG	LEU	61	-2.464	56.074	7.623	1.00	0.00	H
ATOM	637	HD11	LEU	61	-1.684	54.466	5.920	1.00	0.00	H
ATOM	638	HD12	LEU	61	-0.635	55.868	5.941	1.00	0.00	H
ATOM	639	HD13	LEU	61	-0.112	54.359	6.700	1.00	0.00	H
ATOM	640	HD21	LEU	61	-2.856	53.681	8.022	1.00	0.00	H
ATOM	641	HD22	LEU	61	-1.335	53.551	8.900	1.00	0.00	H
ATOM	642	HD23	LEU	61	-2.646	54.539	9.540	1.00	0.00	H
ATOM	643	N	ALA	62	-1.997	58.959	8.841	1.00	0.00	N
ATOM	644	CA	ALA	62	-3.233	59.681	8.647	1.00	0.00	C
ATOM	645	C	ALA	62	-2.986	61.052	8.037	1.00	0.00	C
ATOM	646	O	ALA	62	-3.739	61.467	7.163	1.00	0.00	O
ATOM	647	CB	ALA	62	-3.938	59.853	10.002	1.00	0.00	C
ATOM	648	H	ALA	62	-1.699	58.772	9.790	1.00	0.00	H
ATOM	649	HA	ALA	62	-3.882	59.112	7.977	1.00	0.00	H
ATOM	650	HB1	ALA	62	-4.875	60.400	9.889	1.00	0.00	H
ATOM	651	HB2	ALA	62	-4.180	58.886	10.444	1.00	0.00	H
ATOM	652	HB3	ALA	62	-3.324	60.400	10.719	1.00	0.00	H
ATOM	653	N	PHE	63	-1.889	61.710	8.428	1.00	0.00	N
ATOM	654	CA	PHE	63	-1.414	62.913	7.763	1.00	0.00	C
ATOM	655	C	PHE	63	-0.960	62.629	6.343	1.00	0.00	C
ATOM	656	O	PHE	63	-1.345	63.323	5.406	1.00	0.00	O
ATOM	657	CB	PHE	63	-0.277	63.560	8.584	1.00	0.00	C
ATOM	658	CG	PHE	63	0.086	64.958	8.105	1.00	0.00	C
ATOM	659	CD1	PHE	63	-0.670	66.065	8.532	1.00	0.00	C
ATOM	660	CD2	PHE	63	1.146	65.152	7.198	1.00	0.00	C
ATOM	661	CE1	PHE	63	-0.403	67.348	8.019	1.00	0.00	C
ATOM	662	CE2	PHE	63	1.446	66.438	6.714	1.00	0.00	C
ATOM	663	CZ	PHE	63	0.663	67.536	7.115	1.00	0.00	C
ATOM	664	H	PHE	63	-1.343	61.330	9.196	1.00	0.00	H
ATOM	665	HA	PHE	63	-2.246	63.619	7.719	1.00	0.00	H
ATOM	666	HB2	PHE	63	-0.571	63.632	9.633	1.00	0.00	H
ATOM	667	HB3	PHE	63	0.611	62.925	8.579	1.00	0.00	H
ATOM	668	HD1	PHE	63	-1.459	65.919	9.250	1.00	0.00	H
ATOM	669	HD2	PHE	63	1.738	64.310	6.880	1.00	0.00	H
ATOM	670	HE1	PHE	63	-1.020	68.181	8.317	1.00	0.00	H
ATOM	671	HE2	PHE	63	2.273	66.580	6.033	1.00	0.00	H

ATOM	672	HZ	PHE	63	0.894	68.516	6.728	1.00	0.00	H
ATOM	673	N	GLY	64	-0.208	61.549	6.172	1.00	0.00	N
ATOM	674	CA	GLY	64	0.285	61.138	4.886	1.00	0.00	C
ATOM	675	C	GLY	64	-0.808	60.825	3.862	1.00	0.00	C
ATOM	676	O	GLY	64	-0.812	61.322	2.733	1.00	0.00	O
ATOM	677	H	GLY	64	0.100	61.025	6.988	1.00	0.00	H
ATOM	678	HA2	GLY	64	0.946	61.899	4.476	1.00	0.00	H
ATOM	679	HA3	GLY	64	0.880	60.243	5.055	1.00	0.00	H
ATOM	680	N	PHE	65	-1.800	60.047	4.275	1.00	0.00	N
ATOM	681	CA	PHE	65	-2.911	59.670	3.422	1.00	0.00	C
ATOM	682	C	PHE	65	-3.979	60.753	3.305	1.00	0.00	C
ATOM	683	O	PHE	65	-4.772	60.766	2.363	1.00	0.00	O
ATOM	684	CB	PHE	65	-3.548	58.396	4.023	1.00	0.00	C
ATOM	685	CG	PHE	65	-4.532	57.677	3.113	1.00	0.00	C
ATOM	686	CD1	PHE	65	-4.071	57.032	1.946	1.00	0.00	C
ATOM	687	CD2	PHE	65	-5.910	57.667	3.419	1.00	0.00	C
ATOM	688	CE1	PHE	65	-4.982	56.382	1.090	1.00	0.00	C
ATOM	689	CE2	PHE	65	-6.819	57.016	2.563	1.00	0.00	C
ATOM	690	CZ	PHE	65	-6.355	56.373	1.399	1.00	0.00	C
ATOM	691	H	PHE	65	-1.749	59.650	5.214	1.00	0.00	H
ATOM	692	HA	PHE	65	-2.531	59.425	2.432	1.00	0.00	H
ATOM	693	HB2	PHE	65	-2.763	57.676	4.262	1.00	0.00	H
ATOM	694	HB3	PHE	65	-4.029	58.628	4.975	1.00	0.00	H
ATOM	695	HD1	PHE	65	-3.019	57.041	1.698	1.00	0.00	H
ATOM	696	HD2	PHE	65	-6.277	58.168	4.303	1.00	0.00	H
ATOM	697	HE1	PHE	65	-4.628	55.891	0.195	1.00	0.00	H
ATOM	698	HE2	PHE	65	-7.874	57.016	2.796	1.00	0.00	H
ATOM	699	HZ	PHE	65	-7.053	55.876	0.740	1.00	0.00	H
ATOM	700	N	ALA	66	-3.954	61.726	4.209	1.00	0.00	N
ATOM	701	CA	ALA	66	-4.758	62.909	4.058	1.00	0.00	C
ATOM	702	C	ALA	66	-4.266	63.794	2.925	1.00	0.00	C
ATOM	703	O	ALA	66	-5.043	64.272	2.096	1.00	0.00	O
ATOM	704	CB	ALA	66	-4.675	63.757	5.332	1.00	0.00	C
ATOM	705	H	ALA	66	-3.327	61.661	5.002	1.00	0.00	H
ATOM	706	HA	ALA	66	-5.800	62.640	3.873	1.00	0.00	H
ATOM	707	HB1	ALA	66	-5.140	64.730	5.177	1.00	0.00	H
ATOM	708	HB2	ALA	66	-5.165	63.255	6.160	1.00	0.00	H
ATOM	709	HB3	ALA	66	-3.657	63.968	5.642	1.00	0.00	H
ATOM	710	N	VAL	67	-2.945	63.959	2.885	1.00	0.00	N
ATOM	711	CA	VAL	67	-2.250	64.669	1.841	1.00	0.00	C
ATOM	712	C	VAL	67	-2.433	64.002	0.486	1.00	0.00	C
ATOM	713	O	VAL	67	-2.668	64.690	-0.506	1.00	0.00	O
ATOM	714	CB	VAL	67	-0.728	64.754	2.208	1.00	0.00	C
ATOM	715	CG1	VAL	67	0.179	65.320	1.094	1.00	0.00	C
ATOM	716	CG2	VAL	67	-0.439	65.524	3.510	1.00	0.00	C
ATOM	717	H	VAL	67	-2.389	63.570	3.642	1.00	0.00	H
ATOM	718	HA	VAL	67	-2.657	65.680	1.776	1.00	0.00	H
ATOM	719	HB	VAL	67	-0.382	63.736	2.382	1.00	0.00	H
ATOM	720	HG11	VAL	67	1.208	65.421	1.438	1.00	0.00	H
ATOM	721	HG12	VAL	67	0.210	64.669	0.220	1.00	0.00	H
ATOM	722	HG13	VAL	67	-0.159	66.304	0.764	1.00	0.00	H
ATOM	723	HG21	VAL	67	0.453	65.133	3.998	1.00	0.00	H
ATOM	724	HG22	VAL	67	-0.252	66.577	3.324	1.00	0.00	H
ATOM	725	HG23	VAL	67	-1.249	65.466	4.233	1.00	0.00	H
ATOM	726	N	THR	68	-2.405	62.664	0.477	1.00	0.00	N
ATOM	727	CA	THR	68	-2.686	61.859	-0.695	1.00	0.00	C
ATOM	728	C	THR	68	-4.012	62.249	-1.363	1.00	0.00	C
ATOM	729	O	THR	68	-4.093	62.470	-2.572	1.00	0.00	O
ATOM	730	CB	THR	68	-2.803	60.353	-0.332	1.00	0.00	C
ATOM	731	OG1	THR	68	-1.551	59.865	0.032	1.00	0.00	O
ATOM	732	CG2	THR	68	-3.358	59.411	-1.420	1.00	0.00	C
ATOM	733	H	THR	68	-2.185	62.201	1.350	1.00	0.00	H
ATOM	734	HA	THR	68	-1.888	62.004	-1.426	1.00	0.00	H

ATOM	735	HB	THR	68	-3.456	60.229	0.517	1.00	0.00	H
ATOM	736	HG1	THR	68	-1.166	60.466	0.660	1.00	0.00	H
ATOM	737	HG21	THR	68	-3.288	58.369	-1.110	1.00	0.00	H
ATOM	738	HG22	THR	68	-4.416	59.589	-1.613	1.00	0.00	H
ATOM	739	HG23	THR	68	-2.831	59.532	-2.365	1.00	0.00	H
ATOM	740	N	LEU	69	-5.061	62.307	-0.540	1.00	0.00	N
ATOM	741	CA	LEU	69	-6.410	62.564	-0.985	1.00	0.00	C
ATOM	742	C	LEU	69	-6.568	63.993	-1.480	1.00	0.00	C
ATOM	743	O	LEU	69	-7.173	64.227	-2.525	1.00	0.00	O
ATOM	744	CB	LEU	69	-7.369	62.276	0.198	1.00	0.00	C
ATOM	745	CG	LEU	69	-8.879	62.478	-0.071	1.00	0.00	C
ATOM	746	CD1	LEU	69	-9.402	61.585	-1.209	1.00	0.00	C
ATOM	747	CD2	LEU	69	-9.684	62.237	1.215	1.00	0.00	C
ATOM	748	H	LEU	69	-4.916	62.125	0.446	1.00	0.00	H
ATOM	749	HA	LEU	69	-6.637	61.887	-1.808	1.00	0.00	H
ATOM	750	HB2	LEU	69	-7.209	61.250	0.534	1.00	0.00	H
ATOM	751	HB3	LEU	69	-7.085	62.902	1.045	1.00	0.00	H
ATOM	752	HG	LEU	69	-9.052	63.516	-0.358	1.00	0.00	H
ATOM	753	HD11	LEU	69	-10.477	61.710	-1.343	1.00	0.00	H
ATOM	754	HD12	LEU	69	-8.935	61.833	-2.163	1.00	0.00	H
ATOM	755	HD13	LEU	69	-9.214	60.530	-1.008	1.00	0.00	H
ATOM	756	HD21	LEU	69	-10.745	62.439	1.065	1.00	0.00	H
ATOM	757	HD22	LEU	69	-9.587	61.206	1.559	1.00	0.00	H
ATOM	758	HD23	LEU	69	-9.339	62.885	2.021	1.00	0.00	H
ATOM	759	N	GLY	70	-5.943	64.925	-0.761	1.00	0.00	N
ATOM	760	CA	GLY	70	-5.722	66.287	-1.191	1.00	0.00	C
ATOM	761	C	GLY	70	-5.147	66.434	-2.596	1.00	0.00	C
ATOM	762	O	GLY	70	-5.620	67.252	-3.389	1.00	0.00	O
ATOM	763	H	GLY	70	-5.477	64.623	0.090	1.00	0.00	H
ATOM	764	HA2	GLY	70	-6.665	66.832	-1.132	1.00	0.00	H
ATOM	765	HA3	GLY	70	-5.040	66.767	-0.488	1.00	0.00	H
ATOM	766	N	ILE	71	-4.151	65.601	-2.907	1.00	0.00	N
ATOM	767	CA	ILE	71	-3.525	65.548	-4.212	1.00	0.00	C
ATOM	768	C	ILE	71	-4.449	65.020	-5.296	1.00	0.00	C
ATOM	769	O	ILE	71	-4.578	65.644	-6.353	1.00	0.00	O
ATOM	770	CB	ILE	71	-2.160	64.786	-4.149	1.00	0.00	C
ATOM	771	CG1	ILE	71	-1.132	65.538	-3.268	1.00	0.00	C
ATOM	772	CG2	ILE	71	-1.528	64.523	-5.537	1.00	0.00	C
ATOM	773	CD1	ILE	71	0.017	64.652	-2.763	1.00	0.00	C
ATOM	774	H	ILE	71	-3.776	65.002	-2.177	1.00	0.00	H
ATOM	775	HA	ILE	71	-3.302	66.576	-4.491	1.00	0.00	H
ATOM	776	HB	ILE	71	-2.340	63.816	-3.691	1.00	0.00	H
ATOM	777	HG12	ILE	71	-0.719	66.387	-3.817	1.00	0.00	H
ATOM	778	HG13	ILE	71	-1.617	65.978	-2.399	1.00	0.00	H
ATOM	779	HG21	ILE	71	-0.579	63.995	-5.456	1.00	0.00	H
ATOM	780	HG22	ILE	71	-2.162	63.910	-6.176	1.00	0.00	H
ATOM	781	HG23	ILE	71	-1.334	65.457	-6.066	1.00	0.00	H
ATOM	782	HD11	ILE	71	0.685	65.226	-2.121	1.00	0.00	H
ATOM	783	HD12	ILE	71	-0.357	63.809	-2.181	1.00	0.00	H
ATOM	784	HD13	ILE	71	0.621	64.256	-3.578	1.00	0.00	H
ATOM	785	N	LEU	72	-5.124	63.906	-5.015	1.00	0.00	N
ATOM	786	CA	LEU	72	-6.076	63.296	-5.929	1.00	0.00	C
ATOM	787	C	LEU	72	-7.223	64.222	-6.317	1.00	0.00	C
ATOM	788	O	LEU	72	-7.687	64.216	-7.460	1.00	0.00	O
ATOM	789	CB	LEU	72	-6.611	61.977	-5.326	1.00	0.00	C
ATOM	790	CG	LEU	72	-5.565	60.843	-5.212	1.00	0.00	C
ATOM	791	CD1	LEU	72	-6.068	59.724	-4.287	1.00	0.00	C
ATOM	792	CD2	LEU	72	-5.179	60.261	-6.583	1.00	0.00	C
ATOM	793	H	LEU	72	-4.944	63.458	-4.120	1.00	0.00	H
ATOM	794	HA	LEU	72	-5.545	63.073	-6.855	1.00	0.00	H
ATOM	795	HB2	LEU	72	-7.025	62.191	-4.339	1.00	0.00	H
ATOM	796	HB3	LEU	72	-7.454	61.616	-5.919	1.00	0.00	H
ATOM	797	HG	LEU	72	-4.658	61.244	-4.759	1.00	0.00	H

ATOM	798	HD11	LEU	72	-5.317	58.941	-4.173	1.00	0.00	H
ATOM	799	HD12	LEU	72	-6.290	60.103	-3.290	1.00	0.00	H
ATOM	800	HD13	LEU	72	-6.975	59.261	-4.677	1.00	0.00	H
ATOM	801	HD21	LEU	72	-4.477	59.433	-6.477	1.00	0.00	H
ATOM	802	HD22	LEU	72	-6.052	59.880	-7.116	1.00	0.00	H
ATOM	803	HD23	LEU	72	-4.697	61.003	-7.220	1.00	0.00	H
ATOM	804	N	ILE	73	-7.621	65.056	-5.359	1.00	0.00	N
ATOM	805	CA	ILE	73	-8.588	66.106	-5.537	1.00	0.00	C
ATOM	806	C	ILE	73	-8.164	67.206	-6.524	1.00	0.00	C
ATOM	807	O	ILE	73	-8.977	67.648	-7.341	1.00	0.00	O
ATOM	808	CB	ILE	73	-9.013	66.659	-4.136	1.00	0.00	C
ATOM	809	CG1	ILE	73	-9.892	65.646	-3.356	1.00	0.00	C
ATOM	810	CG2	ILE	73	-9.669	68.059	-4.130	1.00	0.00	C
ATOM	811	CD1	ILE	73	-11.362	65.567	-3.800	1.00	0.00	C
ATOM	812	H	ILE	73	-7.209	64.937	-4.437	1.00	0.00	H
ATOM	813	HA	ILE	73	-9.467	65.648	-5.992	1.00	0.00	H
ATOM	814	HB	ILE	73	-8.100	66.770	-3.551	1.00	0.00	H
ATOM	815	HG12	ILE	73	-9.465	64.647	-3.429	1.00	0.00	H
ATOM	816	HG13	ILE	73	-9.861	65.894	-2.294	1.00	0.00	H
ATOM	817	HG21	ILE	73	-10.024	68.318	-3.134	1.00	0.00	H
ATOM	818	HG22	ILE	73	-8.960	68.835	-4.421	1.00	0.00	H
ATOM	819	HG23	ILE	73	-10.518	68.107	-4.811	1.00	0.00	H
ATOM	820	HD11	ILE	73	-11.886	64.795	-3.236	1.00	0.00	H
ATOM	821	HD12	ILE	73	-11.890	66.505	-3.627	1.00	0.00	H
ATOM	822	HD13	ILE	73	-11.453	65.315	-4.857	1.00	0.00	H
ATOM	823	N	ALA	74	-6.929	67.696	-6.412	1.00	0.00	N
ATOM	824	CA	ALA	74	-6.541	68.967	-7.008	1.00	0.00	C
ATOM	825	C	ALA	74	-5.512	68.873	-8.119	1.00	0.00	C
ATOM	826	O	ALA	74	-5.342	69.837	-8.873	1.00	0.00	O
ATOM	827	CB	ALA	74	-5.962	69.842	-5.880	1.00	0.00	C
ATOM	828	H	ALA	74	-6.315	67.272	-5.724	1.00	0.00	H
ATOM	829	HA	ALA	74	-7.401	69.496	-7.422	1.00	0.00	H
ATOM	830	HB1	ALA	74	-5.663	70.825	-6.246	1.00	0.00	H
ATOM	831	HB2	ALA	74	-6.693	70.003	-5.088	1.00	0.00	H
ATOM	832	HB3	ALA	74	-5.083	69.384	-5.424	1.00	0.00	H
ATOM	833	N	GLY	75	-4.851	67.723	-8.254	1.00	0.00	N
ATOM	834	CA	GLY	75	-3.690	67.554	-9.115	1.00	0.00	C
ATOM	835	C	GLY	75	-3.976	67.726	-10.601	1.00	0.00	C
ATOM	836	O	GLY	75	-3.116	68.121	-11.381	1.00	0.00	O
ATOM	837	H	GLY	75	-5.016	66.986	-7.569	1.00	0.00	H
ATOM	838	HA2	GLY	75	-2.914	68.256	-8.822	1.00	0.00	H
ATOM	839	HA3	GLY	75	-3.276	66.558	-8.953	1.00	0.00	H
ATOM	840	N	GLN	76	-5.246	67.541	-10.949	1.00	0.00	N
ATOM	841	CA	GLN	76	-5.803	67.688	-12.276	1.00	0.00	C
ATOM	842	C	GLN	76	-6.036	69.142	-12.678	1.00	0.00	C
ATOM	843	O	GLN	76	-6.411	69.421	-13.816	1.00	0.00	O
ATOM	844	CB	GLN	76	-7.202	67.015	-12.247	1.00	0.00	C
ATOM	845	CG	GLN	76	-7.289	65.585	-11.653	1.00	0.00	C
ATOM	846	CD	GLN	76	-8.727	65.254	-11.240	1.00	0.00	C
ATOM	847	OE1	GLN	76	-9.672	65.590	-11.946	1.00	0.00	O
ATOM	848	NE2	GLN	76	-8.932	64.646	-10.078	1.00	0.00	N
ATOM	849	H	GLN	76	-5.864	67.252	-10.207	1.00	0.00	H
ATOM	850	HA	GLN	76	-5.157	67.203	-13.011	1.00	0.00	H
ATOM	851	HB2	GLN	76	-7.887	67.649	-11.685	1.00	0.00	H
ATOM	852	HB3	GLN	76	-7.608	66.991	-13.260	1.00	0.00	H
ATOM	853	HG2	GLN	76	-6.949	64.853	-12.387	1.00	0.00	H
ATOM	854	HG3	GLN	76	-6.652	65.460	-10.779	1.00	0.00	H
ATOM	855	HE22	GLN	76	-9.880	64.435	-9.817	1.00	0.00	H
ATOM	856	HE21	GLN	76	-8.187	64.427	-9.421	1.00	0.00	H
ATOM	857	N	VAL	77	-5.925	70.049	-11.709	1.00	0.00	N
ATOM	858	CA	VAL	77	-6.431	71.390	-11.815	1.00	0.00	C
ATOM	859	C	VAL	77	-5.361	72.415	-11.459	1.00	0.00	C
ATOM	860	O	VAL	77	-5.111	73.337	-12.240	1.00	0.00	O

ATOM	861	CB	VAL	77	-7.710	71.567	-10.929	1.00	0.00	C
ATOM	862	CG1	VAL	77	-8.417	72.916	-11.165	1.00	0.00	C
ATOM	863	CG2	VAL	77	-8.732	70.424	-11.065	1.00	0.00	C
ATOM	864	H	VAL	77	-5.578	69.734	-10.808	1.00	0.00	H
ATOM	865	HA	VAL	77	-6.715	71.605	-12.847	1.00	0.00	H
ATOM	866	HB	VAL	77	-7.396	71.558	-9.883	1.00	0.00	H
ATOM	867	HG11	VAL	77	-9.287	73.011	-10.516	1.00	0.00	H
ATOM	868	HG12	VAL	77	-7.763	73.761	-10.948	1.00	0.00	H
ATOM	869	HG13	VAL	77	-8.753	73.011	-12.198	1.00	0.00	H
ATOM	870	HG21	VAL	77	-9.659	70.648	-10.539	1.00	0.00	H
ATOM	871	HG22	VAL	77	-8.970	70.216	-12.109	1.00	0.00	H
ATOM	872	HG23	VAL	77	-8.346	69.509	-10.615	1.00	0.00	H
ATOM	873	N	SER	78	-4.783	72.301	-10.259	1.00	0.00	N
ATOM	874	CA	SER	78	-3.750	73.209	-9.806	1.00	0.00	C
ATOM	875	C	SER	78	-2.348	72.663	-10.126	1.00	0.00	C
ATOM	876	O	SER	78	-1.438	73.437	-10.445	1.00	0.00	O
ATOM	877	CB	SER	78	-3.891	73.448	-8.297	1.00	0.00	C
ATOM	878	OG	SER	78	-3.747	72.283	-7.515	1.00	0.00	O
ATOM	879	H	SER	78	-5.013	71.523	-9.641	1.00	0.00	H
ATOM	880	HA	SER	78	-3.846	74.177	-10.299	1.00	0.00	H
ATOM	881	HB2	SER	78	-3.113	74.152	-8.000	1.00	0.00	H
ATOM	882	HB3	SER	78	-4.838	73.921	-8.058	1.00	0.00	H
ATOM	883	HG	SER	78	-3.210	72.559	-6.743	1.00	0.00	H
ATOM	884	N	GLY	79	-2.193	71.343	-10.030	1.00	0.00	N
ATOM	885	CA	GLY	79	-0.927	70.623	-10.006	1.00	0.00	C
ATOM	886	C	GLY	79	-0.734	69.950	-8.644	1.00	0.00	C
ATOM	887	O	GLY	79	0.065	69.028	-8.548	1.00	0.00	O
ATOM	888	H	GLY	79	-3.000	70.806	-9.745	1.00	0.00	H
ATOM	889	HA2	GLY	79	-0.935	69.857	-10.782	1.00	0.00	H
ATOM	890	HA3	GLY	79	-0.072	71.273	-10.200	1.00	0.00	H
ATOM	891	N	ALA	80	-1.486	70.390	-7.618	1.00	0.00	N
ATOM	892	CA	ALA	80	-1.486	69.928	-6.230	1.00	0.00	C
ATOM	893	C	ALA	80	-0.129	70.177	-5.570	1.00	0.00	C
ATOM	894	O	ALA	80	0.579	69.230	-5.230	1.00	0.00	O
ATOM	895	CB	ALA	80	-1.944	68.465	-6.099	1.00	0.00	C
ATOM	896	H	ALA	80	-2.104	71.172	-7.793	1.00	0.00	H
ATOM	897	HA	ALA	80	-2.205	70.544	-5.689	1.00	0.00	H
ATOM	898	HB1	ALA	80	-1.857	68.148	-5.060	1.00	0.00	H
ATOM	899	HB2	ALA	80	-2.983	68.342	-6.388	1.00	0.00	H
ATOM	900	HB3	ALA	80	-1.330	67.787	-6.693	1.00	0.00	H
ATOM	901	N	HIE	81	0.235	71.453	-5.395	1.00	0.00	N
ATOM	902	CA	HIE	81	1.458	71.799	-4.673	1.00	0.00	C
ATOM	903	C	HIE	81	1.169	71.716	-3.167	1.00	0.00	C
ATOM	904	O	HIE	81	1.900	71.053	-2.433	1.00	0.00	O
ATOM	905	CB	HIE	81	1.904	73.236	-5.000	1.00	0.00	C
ATOM	906	CG	HIE	81	2.387	73.527	-6.392	1.00	0.00	C
ATOM	907	ND1	HIE	81	2.821	74.816	-6.722	1.00	0.00	N
ATOM	908	CD2	HIE	81	2.490	72.729	-7.514	1.00	0.00	C
ATOM	909	CE1	HIE	81	3.156	74.759	-7.998	1.00	0.00	C
ATOM	910	NE2	HIE	81	2.979	73.552	-8.522	1.00	0.00	N
ATOM	911	H	HIE	81	-0.464	72.177	-5.564	1.00	0.00	H
ATOM	912	HA	HIE	81	2.264	71.104	-4.918	1.00	0.00	H
ATOM	913	HB2	HIE	81	1.091	73.930	-4.805	1.00	0.00	H
ATOM	914	HB3	HIE	81	2.714	73.508	-4.322	1.00	0.00	H
ATOM	915	HD2	HIE	81	2.253	71.683	-7.661	1.00	0.00	H
ATOM	916	HE1	HIE	81	3.520	75.607	-8.560	1.00	0.00	H
ATOM	917	HE2	HIE	81	3.166	73.358	-9.514	1.00	0.00	H
ATOM	918	N	LEU	82	0.098	72.391	-2.717	1.00	0.00	N
ATOM	919	CA	LEU	82	-0.512	72.340	-1.381	1.00	0.00	C
ATOM	920	C	LEU	82	0.440	72.810	-0.261	1.00	0.00	C
ATOM	921	O	LEU	82	0.261	72.474	0.911	1.00	0.00	O
ATOM	922	CB	LEU	82	-1.112	70.943	-1.067	1.00	0.00	C
ATOM	923	CG	LEU	82	-1.977	70.300	-2.175	1.00	0.00	C

ATOM	924	CD1	LEU	82	-2.593	68.982	-1.686	1.00	0.00	C
ATOM	925	CD2	LEU	82	-3.061	71.245	-2.719	1.00	0.00	C
ATOM	926	H	LEU	82	-0.399	72.986	-3.386	1.00	0.00	H
ATOM	927	HA	LEU	82	-1.326	73.060	-1.410	1.00	0.00	H
ATOM	928	HB2	LEU	82	-0.301	70.256	-0.820	1.00	0.00	H
ATOM	929	HB3	LEU	82	-1.711	71.029	-0.159	1.00	0.00	H
ATOM	930	HG	LEU	82	-1.318	70.043	-3.003	1.00	0.00	H
ATOM	931	HD11	LEU	82	-3.128	68.484	-2.494	1.00	0.00	H
ATOM	932	HD12	LEU	82	-1.821	68.293	-1.339	1.00	0.00	H
ATOM	933	HD13	LEU	82	-3.290	69.141	-0.863	1.00	0.00	H
ATOM	934	HD21	LEU	82	-3.733	70.730	-3.406	1.00	0.00	H
ATOM	935	HD22	LEU	82	-3.648	71.679	-1.913	1.00	0.00	H
ATOM	936	HD23	LEU	82	-2.616	72.070	-3.273	1.00	0.00	H
ATOM	937	N	ASN	83	1.508	73.520	-0.632	1.00	0.00	N
ATOM	938	CA	ASN	83	2.704	73.731	0.169	1.00	0.00	C
ATOM	939	C	ASN	83	3.510	74.868	-0.485	1.00	0.00	C
ATOM	940	O	ASN	83	4.045	74.648	-1.575	1.00	0.00	O
ATOM	941	CB	ASN	83	3.514	72.407	0.202	1.00	0.00	C
ATOM	942	CG	ASN	83	4.728	72.426	1.132	1.00	0.00	C
ATOM	943	OD1	ASN	83	5.251	73.484	1.467	1.00	0.00	O
ATOM	944	ND2	ASN	83	5.167	71.263	1.599	1.00	0.00	N
ATOM	945	H	ASN	83	1.572	73.760	-1.613	1.00	0.00	H
ATOM	946	HA	ASN	83	2.406	73.980	1.184	1.00	0.00	H
ATOM	947	HB2	ASN	83	2.868	71.586	0.510	1.00	0.00	H
ATOM	948	HB3	ASN	83	3.836	72.159	-0.803	1.00	0.00	H
ATOM	949	HD22	ASN	83	5.918	71.238	2.293	1.00	0.00	H
ATOM	950	HD21	ASN	83	4.768	70.397	1.272	1.00	0.00	H
ATOM	951	N	PRO	84	3.669	76.043	0.166	1.00	0.00	N
ATOM	952	CA	PRO	84	4.537	77.137	-0.304	1.00	0.00	C
ATOM	953	C	PRO	84	5.989	76.721	-0.614	1.00	0.00	C
ATOM	954	O	PRO	84	6.596	77.245	-1.552	1.00	0.00	O
ATOM	955	CB	PRO	84	4.521	78.164	0.839	1.00	0.00	C
ATOM	956	CG	PRO	84	3.190	77.937	1.538	1.00	0.00	C
ATOM	957	CD	PRO	84	2.908	76.448	1.350	1.00	0.00	C
ATOM	958	HA	PRO	84	4.081	77.565	-1.199	1.00	0.00	H
ATOM	959	HB2	PRO	84	5.330	77.979	1.545	1.00	0.00	H
ATOM	960	HB3	PRO	84	4.645	79.182	0.470	1.00	0.00	H
ATOM	961	HG2	PRO	84	3.179	78.250	2.581	1.00	0.00	H
ATOM	962	HG3	PRO	84	2.413	78.502	1.021	1.00	0.00	H
ATOM	963	HD3	PRO	84	1.841	76.262	1.225	1.00	0.00	H
ATOM	964	HD2	PRO	84	3.259	75.882	2.214	1.00	0.00	H
ATOM	965	N	ALA	85	6.538	75.758	0.147	1.00	0.00	N
ATOM	966	CA	ALA	85	7.893	75.248	-0.023	1.00	0.00	C
ATOM	967	C	ALA	85	8.025	74.419	-1.308	1.00	0.00	C
ATOM	968	O	ALA	85	9.061	74.507	-1.964	1.00	0.00	O
ATOM	969	CB	ALA	85	8.291	74.397	1.193	1.00	0.00	C
ATOM	970	H	ALA	85	5.958	75.289	0.833	1.00	0.00	H
ATOM	971	HA	ALA	85	8.574	76.092	-0.092	1.00	0.00	H
ATOM	972	HB1	ALA	85	9.355	74.164	1.167	1.00	0.00	H
ATOM	973	HB2	ALA	85	8.113	74.931	2.125	1.00	0.00	H
ATOM	974	HB3	ALA	85	7.761	73.446	1.235	1.00	0.00	H
ATOM	975	N	VAL	86	6.973	73.689	-1.716	1.00	0.00	N
ATOM	976	CA	VAL	86	6.917	73.014	-3.015	1.00	0.00	C
ATOM	977	C	VAL	86	6.887	74.050	-4.134	1.00	0.00	C
ATOM	978	O	VAL	86	7.661	73.968	-5.085	1.00	0.00	O
ATOM	979	CB	VAL	86	5.650	72.094	-3.144	1.00	0.00	C
ATOM	980	CG1	VAL	86	5.396	71.545	-4.568	1.00	0.00	C
ATOM	981	CG2	VAL	86	5.657	70.894	-2.190	1.00	0.00	C
ATOM	982	H	VAL	86	6.120	73.745	-1.178	1.00	0.00	H
ATOM	983	HA	VAL	86	7.816	72.407	-3.145	1.00	0.00	H
ATOM	984	HB	VAL	86	4.773	72.688	-2.896	1.00	0.00	H
ATOM	985	HG11	VAL	86	4.585	70.817	-4.569	1.00	0.00	H
ATOM	986	HG12	VAL	86	5.106	72.328	-5.269	1.00	0.00	H

ATOM	987	HG13	VAL	86	6.279	71.045	-4.968	1.00	0.00	H
ATOM	988	HG21	VAL	86	4.659	70.463	-2.102	1.00	0.00	H
ATOM	989	HG22	VAL	86	6.290	70.099	-2.580	1.00	0.00	H
ATOM	990	HG23	VAL	86	5.997	71.159	-1.191	1.00	0.00	H
ATOM	991	N	THR	87	5.987	75.023	-4.016	1.00	0.00	N
ATOM	992	CA	THR	87	5.701	75.985	-5.057	1.00	0.00	C
ATOM	993	C	THR	87	6.888	76.849	-5.437	1.00	0.00	C
ATOM	994	O	THR	87	7.145	77.073	-6.622	1.00	0.00	O
ATOM	995	CB	THR	87	4.521	76.874	-4.552	1.00	0.00	C
ATOM	996	OG1	THR	87	3.381	76.072	-4.309	1.00	0.00	O
ATOM	997	CG2	THR	87	4.058	77.977	-5.520	1.00	0.00	C
ATOM	998	H	THR	87	5.344	75.007	-3.229	1.00	0.00	H
ATOM	999	HA	THR	87	5.393	75.439	-5.950	1.00	0.00	H
ATOM	1000	HB	THR	87	4.797	77.348	-3.608	1.00	0.00	H
ATOM	1001	HG1	THR	87	3.174	75.607	-5.123	1.00	0.00	H
ATOM	1002	HG21	THR	87	3.217	78.526	-5.097	1.00	0.00	H
ATOM	1003	HG22	THR	87	4.847	78.706	-5.706	1.00	0.00	H
ATOM	1004	HG23	THR	87	3.746	77.567	-6.481	1.00	0.00	H
ATOM	1005	N	PHE	88	7.634	77.281	-4.422	1.00	0.00	N
ATOM	1006	CA	PHE	88	8.857	78.019	-4.621	1.00	0.00	C
ATOM	1007	C	PHE	88	9.910	77.166	-5.335	1.00	0.00	C
ATOM	1008	O	PHE	88	10.582	77.622	-6.263	1.00	0.00	O
ATOM	1009	CB	PHE	88	9.375	78.515	-3.254	1.00	0.00	C
ATOM	1010	CG	PHE	88	10.345	79.681	-3.334	1.00	0.00	C
ATOM	1011	CD1	PHE	88	9.877	81.000	-3.151	1.00	0.00	C
ATOM	1012	CD2	PHE	88	11.713	79.457	-3.596	1.00	0.00	C
ATOM	1013	CE1	PHE	88	10.770	82.086	-3.234	1.00	0.00	C
ATOM	1014	CE2	PHE	88	12.605	80.543	-3.681	1.00	0.00	C
ATOM	1015	CZ	PHE	88	12.134	81.858	-3.500	1.00	0.00	C
ATOM	1016	H	PHE	88	7.341	77.070	-3.471	1.00	0.00	H
ATOM	1017	HA	PHE	88	8.633	78.886	-5.245	1.00	0.00	H
ATOM	1018	HB2	PHE	88	8.534	78.835	-2.638	1.00	0.00	H
ATOM	1019	HB3	PHE	88	9.844	77.699	-2.702	1.00	0.00	H
ATOM	1020	HD1	PHE	88	8.832	81.186	-2.946	1.00	0.00	H
ATOM	1021	HD2	PHE	88	12.086	78.453	-3.738	1.00	0.00	H
ATOM	1022	HE1	PHE	88	10.410	83.094	-3.092	1.00	0.00	H
ATOM	1023	HE2	PHE	88	13.651	80.365	-3.884	1.00	0.00	H
ATOM	1024	HZ	PHE	88	12.821	82.689	-3.563	1.00	0.00	H
ATOM	1025	N	ALA	89	9.986	75.892	-4.943	1.00	0.00	N
ATOM	1026	CA	ALA	89	10.938	74.948	-5.474	1.00	0.00	C
ATOM	1027	C	ALA	89	10.667	74.537	-6.919	1.00	0.00	C
ATOM	1028	O	ALA	89	11.583	74.481	-7.740	1.00	0.00	O
ATOM	1029	CB	ALA	89	11.005	73.724	-4.554	1.00	0.00	C
ATOM	1030	H	ALA	89	9.350	75.562	-4.229	1.00	0.00	H
ATOM	1031	HA	ALA	89	11.921	75.426	-5.451	1.00	0.00	H
ATOM	1032	HB1	ALA	89	11.900	73.147	-4.777	1.00	0.00	H
ATOM	1033	HB2	ALA	89	11.102	74.022	-3.511	1.00	0.00	H
ATOM	1034	HB3	ALA	89	10.137	73.072	-4.646	1.00	0.00	H
ATOM	1035	N	MET	90	9.389	74.358	-7.253	1.00	0.00	N
ATOM	1036	CA	MET	90	8.910	74.126	-8.608	1.00	0.00	C
ATOM	1037	C	MET	90	9.215	75.302	-9.518	1.00	0.00	C
ATOM	1038	O	MET	90	9.569	75.123	-10.683	1.00	0.00	O
ATOM	1039	CB	MET	90	7.381	73.893	-8.560	1.00	0.00	C
ATOM	1040	CG	MET	90	6.941	72.565	-7.920	1.00	0.00	C
ATOM	1041	SD	MET	90	7.050	71.111	-8.992	1.00	0.00	S
ATOM	1042	CE	MET	90	5.617	71.363	-10.076	1.00	0.00	C
ATOM	1043	H	MET	90	8.711	74.364	-6.492	1.00	0.00	H
ATOM	1044	HA	MET	90	9.409	73.239	-8.998	1.00	0.00	H
ATOM	1045	HB2	MET	90	6.913	74.713	-8.014	1.00	0.00	H
ATOM	1046	HB3	MET	90	6.963	73.939	-9.562	1.00	0.00	H
ATOM	1047	HG2	MET	90	7.533	72.356	-7.030	1.00	0.00	H
ATOM	1048	HG3	MET	90	5.908	72.648	-7.581	1.00	0.00	H
ATOM	1049	HE1	MET	90	5.544	70.544	-10.791	1.00	0.00	H

ATOM	1050	HE2	MET	90	4.697	71.387	-9.493	1.00	0.00	H
ATOM	1051	HE3	MET	90	5.700	72.295	-10.636	1.00	0.00	H
ATOM	1052	N	CYS	91	9.088	76.508	-8.970	1.00	0.00	N
ATOM	1053	CA	CYS	91	9.319	77.721	-9.712	1.00	0.00	C
ATOM	1054	C	CYS	91	10.786	77.935	-10.058	1.00	0.00	C
ATOM	1055	O	CYS	91	11.116	78.270	-11.194	1.00	0.00	O
ATOM	1056	CB	CYS	91	8.742	78.917	-8.927	1.00	0.00	C
ATOM	1057	SG	CYS	91	8.493	80.349	-10.016	1.00	0.00	S
ATOM	1058	H	CYS	91	8.783	76.579	-8.006	1.00	0.00	H
ATOM	1059	HA	CYS	91	8.778	77.641	-10.653	1.00	0.00	H
ATOM	1060	HB2	CYS	91	7.761	78.662	-8.529	1.00	0.00	H
ATOM	1061	HB3	CYS	91	9.371	79.198	-8.081	1.00	0.00	H
ATOM	1062	HG	CYS	91	7.934	79.648	-11.017	1.00	0.00	H
ATOM	1063	N	PHE	92	11.657	77.653	-9.094	1.00	0.00	N
ATOM	1064	CA	PHE	92	13.093	77.706	-9.272	1.00	0.00	C
ATOM	1065	C	PHE	92	13.592	76.590	-10.196	1.00	0.00	C
ATOM	1066	O	PHE	92	14.491	76.840	-10.996	1.00	0.00	O
ATOM	1067	CB	PHE	92	13.716	77.565	-7.861	1.00	0.00	C
ATOM	1068	CG	PHE	92	15.232	77.606	-7.763	1.00	0.00	C
ATOM	1069	CD1	PHE	92	15.946	78.736	-8.214	1.00	0.00	C
ATOM	1070	CD2	PHE	92	15.934	76.515	-7.205	1.00	0.00	C
ATOM	1071	CE1	PHE	92	17.350	78.772	-8.115	1.00	0.00	C
ATOM	1072	CE2	PHE	92	17.338	76.553	-7.106	1.00	0.00	C
ATOM	1073	CZ	PHE	92	18.047	77.681	-7.561	1.00	0.00	C
ATOM	1074	H	PHE	92	11.299	77.414	-8.175	1.00	0.00	H
ATOM	1075	HA	PHE	92	13.369	78.670	-9.704	1.00	0.00	H
ATOM	1076	HB2	PHE	92	13.339	78.366	-7.223	1.00	0.00	H
ATOM	1077	HB3	PHE	92	13.365	76.640	-7.400	1.00	0.00	H
ATOM	1078	HD1	PHE	92	15.422	79.578	-8.643	1.00	0.00	H
ATOM	1079	HD2	PHE	92	15.403	75.642	-6.855	1.00	0.00	H
ATOM	1080	HE1	PHE	92	17.894	79.636	-8.468	1.00	0.00	H
ATOM	1081	HE2	PHE	92	17.874	75.715	-6.683	1.00	0.00	H
ATOM	1082	HZ	PHE	92	19.125	77.708	-7.488	1.00	0.00	H
ATOM	1083	N	LEU	93	13.018	75.376	-10.102	1.00	0.00	N
ATOM	1084	CA	LEU	93	13.637	74.198	-10.692	1.00	0.00	C
ATOM	1085	C	LEU	93	12.781	73.189	-11.462	1.00	0.00	C
ATOM	1086	O	LEU	93	13.348	72.454	-12.271	1.00	0.00	O
ATOM	1087	CB	LEU	93	14.662	73.547	-9.735	1.00	0.00	C
ATOM	1088	CG	LEU	93	16.144	73.932	-9.947	1.00	0.00	C
ATOM	1089	CD1	LEU	93	17.008	73.381	-8.801	1.00	0.00	C
ATOM	1090	CD2	LEU	93	16.703	73.426	-11.290	1.00	0.00	C
ATOM	1091	H	LEU	93	12.327	75.234	-9.372	1.00	0.00	H
ATOM	1092	HA	LEU	93	14.219	74.562	-11.537	1.00	0.00	H
ATOM	1093	HB2	LEU	93	14.377	73.806	-8.715	1.00	0.00	H
ATOM	1094	HB3	LEU	93	14.590	72.460	-9.800	1.00	0.00	H
ATOM	1095	HG	LEU	93	16.227	75.021	-9.927	1.00	0.00	H
ATOM	1096	HD11	LEU	93	18.042	73.713	-8.894	1.00	0.00	H
ATOM	1097	HD12	LEU	93	16.647	73.712	-7.828	1.00	0.00	H
ATOM	1098	HD13	LEU	93	17.007	72.291	-8.799	1.00	0.00	H
ATOM	1099	HD21	LEU	93	17.765	73.657	-11.381	1.00	0.00	H
ATOM	1100	HD22	LEU	93	16.593	72.346	-11.385	1.00	0.00	H
ATOM	1101	HD23	LEU	93	16.211	73.889	-12.145	1.00	0.00	H
ATOM	1102	N	ALA	94	11.453	73.155	-11.320	1.00	0.00	N
ATOM	1103	CA	ALA	94	10.595	72.313	-12.172	1.00	0.00	C
ATOM	1104	C	ALA	94	10.174	73.069	-13.445	1.00	0.00	C
ATOM	1105	O	ALA	94	9.577	72.474	-14.336	1.00	0.00	O
ATOM	1106	CB	ALA	94	9.323	71.912	-11.417	1.00	0.00	C
ATOM	1107	H	ALA	94	11.000	73.838	-10.723	1.00	0.00	H
ATOM	1108	HA	ALA	94	11.110	71.397	-12.468	1.00	0.00	H
ATOM	1109	HB1	ALA	94	8.736	71.201	-12.001	1.00	0.00	H
ATOM	1110	HB2	ALA	94	9.569	71.416	-10.479	1.00	0.00	H
ATOM	1111	HB3	ALA	94	8.670	72.758	-11.209	1.00	0.00	H
ATOM	1112	N	ARG	95	10.512	74.364	-13.543	1.00	0.00	N

ATOM	1113	CA	ARG	95	10.265	75.270	-14.669	1.00	0.00	C
ATOM	1114	C	ARG	95	8.822	75.823	-14.650	1.00	0.00	C
ATOM	1115	O	ARG	95	8.288	76.225	-15.682	1.00	0.00	O
ATOM	1116	CB	ARG	95	10.738	74.701	-16.038	1.00	0.00	C
ATOM	1117	CG	ARG	95	12.196	74.170	-16.066	1.00	0.00	C
ATOM	1118	CD	ARG	95	12.338	72.630	-16.175	1.00	0.00	C
ATOM	1119	NE	ARG	95	13.151	72.068	-15.080	1.00	0.00	N
ATOM	1120	CZ	ARG	95	13.930	70.977	-15.074	1.00	0.00	C
ATOM	1121	NH1	ARG	95	14.103	70.217	-16.156	1.00	0.00	N
ATOM	1122	NH2	ARG	95	14.544	70.656	-13.941	1.00	0.00	N1+
ATOM	1123	H	ARG	95	10.921	74.775	-12.714	1.00	0.00	H
ATOM	1124	HA	ARG	95	10.888	76.143	-14.465	1.00	0.00	H
ATOM	1125	HB2	ARG	95	10.038	73.947	-16.398	1.00	0.00	H
ATOM	1126	HB3	ARG	95	10.659	75.508	-16.768	1.00	0.00	H
ATOM	1127	HG2	ARG	95	12.689	74.607	-16.936	1.00	0.00	H
ATOM	1128	HG3	ARG	95	12.754	74.562	-15.214	1.00	0.00	H
ATOM	1129	HD2	ARG	95	11.370	72.128	-16.182	1.00	0.00	H
ATOM	1130	HD3	ARG	95	12.809	72.388	-17.128	1.00	0.00	H
ATOM	1131	HE	ARG	95	13.026	72.524	-14.177	1.00	0.00	H
ATOM	1132	HH12	ARG	95	14.677	69.382	-16.118	1.00	0.00	H
ATOM	1133	HH11	ARG	95	13.610	70.401	-17.016	1.00	0.00	H
ATOM	1134	HH22	ARG	95	15.214	69.871	-13.851	1.00	0.00	H
ATOM	1135	HH21	ARG	95	14.339	71.185	-13.092	1.00	0.00	H
ATOM	1136	N	GLU	96	8.191	75.814	-13.469	1.00	0.00	N
ATOM	1137	CA	GLU	96	6.790	76.135	-13.215	1.00	0.00	C
ATOM	1138	C	GLU	96	6.576	77.672	-13.255	1.00	0.00	C
ATOM	1139	O	GLU	96	7.212	78.368	-12.458	1.00	0.00	O
ATOM	1140	CB	GLU	96	6.520	75.576	-11.799	1.00	0.00	C
ATOM	1141	CG	GLU	96	5.138	75.769	-11.171	1.00	0.00	C
ATOM	1142	CD	GLU	96	4.071	74.918	-11.824	1.00	0.00	C
ATOM	1143	OE1	GLU	96	3.407	74.130	-11.119	1.00	0.00	O
ATOM	1144	OE2	GLU	96	3.701	75.208	-12.980	1.00	0.00	O1-
ATOM	1145	H	GLU	96	8.734	75.517	-12.670	1.00	0.00	H
ATOM	1146	HA	GLU	96	6.169	75.600	-13.932	1.00	0.00	H
ATOM	1147	HB2	GLU	96	6.763	74.512	-11.787	1.00	0.00	H
ATOM	1148	HB3	GLU	96	7.208	76.057	-11.114	1.00	0.00	H
ATOM	1149	HG2	GLU	96	5.193	75.499	-10.118	1.00	0.00	H
ATOM	1150	HG3	GLU	96	4.836	76.816	-11.199	1.00	0.00	H
ATOM	1151	N	PRO	97	5.732	78.224	-14.163	1.00	0.00	N
ATOM	1152	CA	PRO	97	5.467	79.668	-14.306	1.00	0.00	C
ATOM	1153	C	PRO	97	5.178	80.447	-13.005	1.00	0.00	C
ATOM	1154	O	PRO	97	4.222	80.153	-12.283	1.00	0.00	O
ATOM	1155	CB	PRO	97	4.270	79.754	-15.267	1.00	0.00	C
ATOM	1156	CG	PRO	97	4.396	78.518	-16.136	1.00	0.00	C
ATOM	1157	CD	PRO	97	5.040	77.478	-15.220	1.00	0.00	C
ATOM	1158	HA	PRO	97	6.348	80.091	-14.794	1.00	0.00	H
ATOM	1159	HB2	PRO	97	3.328	79.700	-14.718	1.00	0.00	H
ATOM	1160	HB3	PRO	97	4.268	80.675	-15.850	1.00	0.00	H
ATOM	1161	HG2	PRO	97	3.445	78.184	-16.552	1.00	0.00	H
ATOM	1162	HG3	PRO	97	5.068	78.727	-16.970	1.00	0.00	H
ATOM	1163	HD3	PRO	97	5.718	76.836	-15.786	1.00	0.00	H
ATOM	1164	HD2	PRO	97	4.273	76.849	-14.772	1.00	0.00	H
ATOM	1165	N	TRP	98	5.965	81.505	-12.766	1.00	0.00	N
ATOM	1166	CA	TRP	98	5.944	82.410	-11.607	1.00	0.00	C
ATOM	1167	C	TRP	98	4.598	83.108	-11.348	1.00	0.00	C
ATOM	1168	O	TRP	98	4.340	83.548	-10.231	1.00	0.00	O
ATOM	1169	CB	TRP	98	7.069	83.457	-11.759	1.00	0.00	C
ATOM	1170	CG	TRP	98	7.152	84.158	-13.086	1.00	0.00	C
ATOM	1171	CD1	TRP	98	8.022	83.847	-14.074	1.00	0.00	C
ATOM	1172	CD2	TRP	98	6.348	85.268	-13.597	1.00	0.00	C
ATOM	1173	NE1	TRP	98	7.795	84.662	-15.164	1.00	0.00	N
ATOM	1174	CE2	TRP	98	6.774	85.558	-14.930	1.00	0.00	C
ATOM	1175	CE3	TRP	98	5.306	86.069	-13.072	1.00	0.00	C

ATOM	1176	CZ2	TRP	98	6.185	86.573	-15.704	1.00	0.00	C
ATOM	1177	CZ3	TRP	98	4.704	87.088	-13.839	1.00	0.00	C
ATOM	1178	CH2	TRP	98	5.141	87.339	-15.154	1.00	0.00	C
ATOM	1179	H	TRP	98	6.724	81.659	-13.414	1.00	0.00	H
ATOM	1180	HA	TRP	98	6.141	81.813	-10.717	1.00	0.00	H
ATOM	1181	HB2	TRP	98	6.998	84.213	-10.975	1.00	0.00	H
ATOM	1182	HB3	TRP	98	8.026	82.962	-11.587	1.00	0.00	H
ATOM	1183	HD1	TRP	98	8.773	83.070	-14.005	1.00	0.00	H
ATOM	1184	HE1	TRP	98	8.363	84.628	-16.000	1.00	0.00	H
ATOM	1185	HE3	TRP	98	4.968	85.902	-12.059	1.00	0.00	H
ATOM	1186	HZ2	TRP	98	6.535	86.772	-16.706	1.00	0.00	H
ATOM	1187	HZ3	TRP	98	3.914	87.686	-13.410	1.00	0.00	H
ATOM	1188	HH2	TRP	98	4.686	88.129	-15.734	1.00	0.00	H
ATOM	1189	N	ILE	99	3.722	83.177	-12.357	1.00	0.00	N
ATOM	1190	CA	ILE	99	2.360	83.710	-12.279	1.00	0.00	C
ATOM	1191	C	ILE	99	1.438	82.853	-11.374	1.00	0.00	C
ATOM	1192	O	ILE	99	0.372	83.316	-10.972	1.00	0.00	O
ATOM	1193	CB	ILE	99	1.816	83.869	-13.730	1.00	0.00	C
ATOM	1194	CG1	ILE	99	0.489	84.661	-13.788	1.00	0.00	C
ATOM	1195	CG2	ILE	99	1.709	82.529	-14.492	1.00	0.00	C
ATOM	1196	CD1	ILE	99	0.151	85.212	-15.181	1.00	0.00	C
ATOM	1197	H	ILE	99	4.027	82.791	-13.236	1.00	0.00	H
ATOM	1198	HA	ILE	99	2.424	84.699	-11.818	1.00	0.00	H
ATOM	1199	HB	ILE	99	2.554	84.477	-14.256	1.00	0.00	H
ATOM	1200	HG12	ILE	99	-0.338	84.047	-13.429	1.00	0.00	H
ATOM	1201	HG13	ILE	99	0.550	85.506	-13.100	1.00	0.00	H
ATOM	1202	HG21	ILE	99	1.461	82.685	-15.541	1.00	0.00	H
ATOM	1203	HG22	ILE	99	2.650	81.983	-14.472	1.00	0.00	H
ATOM	1204	HG23	ILE	99	0.944	81.882	-14.063	1.00	0.00	H
ATOM	1205	HD11	ILE	99	-0.752	85.823	-15.143	1.00	0.00	H
ATOM	1206	HD12	ILE	99	0.956	85.843	-15.561	1.00	0.00	H
ATOM	1207	HD13	ILE	99	-0.023	84.416	-15.904	1.00	0.00	H
ATOM	1208	N	LYS	100	1.848	81.621	-11.030	1.00	0.00	N
ATOM	1209	CA	LYS	100	1.224	80.824	-9.980	1.00	0.00	C
ATOM	1210	C	LYS	100	1.672	81.328	-8.590	1.00	0.00	C
ATOM	1211	O	LYS	100	0.823	81.564	-7.736	1.00	0.00	O
ATOM	1212	CB	LYS	100	1.650	79.343	-10.143	1.00	0.00	C
ATOM	1213	CG	LYS	100	0.990	78.613	-11.334	1.00	0.00	C
ATOM	1214	CD	LYS	100	1.435	77.140	-11.417	1.00	0.00	C
ATOM	1215	CE	LYS	100	0.722	76.297	-12.498	1.00	0.00	C
ATOM	1216	NZ	LYS	100	1.198	74.902	-12.528	1.00	0.00	N1+
ATOM	1217	H	LYS	100	2.725	81.290	-11.418	1.00	0.00	H
ATOM	1218	HA	LYS	100	0.136	80.900	-10.037	1.00	0.00	H
ATOM	1219	HB2	LYS	100	2.736	79.270	-10.223	1.00	0.00	H
ATOM	1220	HB3	LYS	100	1.397	78.799	-9.230	1.00	0.00	H
ATOM	1221	HG2	LYS	100	-0.093	78.670	-11.234	1.00	0.00	H
ATOM	1222	HG3	LYS	100	1.247	79.125	-12.262	1.00	0.00	H
ATOM	1223	HD2	LYS	100	2.506	77.147	-11.609	1.00	0.00	H
ATOM	1224	HD3	LYS	100	1.307	76.664	-10.444	1.00	0.00	H
ATOM	1225	HE2	LYS	100	-0.351	76.285	-12.328	1.00	0.00	H
ATOM	1226	HE3	LYS	100	0.892	76.736	-13.481	1.00	0.00	H
ATOM	1227	HZ1	LYS	100	0.748	74.299	-13.192	1.00	0.00	H
ATOM	1228	HZ2	LYS	100	2.215	74.934	-12.764	1.00	0.00	H
ATOM	1229	HZ3	LYS	100	1.234	74.470	-11.614	1.00	0.00	H
ATOM	1230	N	LEU	101	2.990	81.485	-8.380	1.00	0.00	N
ATOM	1231	CA	LEU	101	3.715	81.681	-7.112	1.00	0.00	C
ATOM	1232	C	LEU	101	2.990	82.286	-5.916	1.00	0.00	C
ATOM	1233	O	LEU	101	2.637	81.552	-4.984	1.00	0.00	O
ATOM	1234	CB	LEU	101	5.207	82.040	-7.338	1.00	0.00	C
ATOM	1235	CG	LEU	101	6.116	81.977	-6.082	1.00	0.00	C
ATOM	1236	CD1	LEU	101	7.507	81.431	-6.438	1.00	0.00	C
ATOM	1237	CD2	LEU	101	6.258	83.339	-5.379	1.00	0.00	C
ATOM	1238	H	LEU	101	3.573	81.396	-9.197	1.00	0.00	H

ATOM	1239	HA	LEU	101	3.790	80.640	-6.794	1.00	0.00	H
ATOM	1240	HB2	LEU	101	5.591	81.319	-8.061	1.00	0.00	H
ATOM	1241	HB3	LEU	101	5.319	83.000	-7.838	1.00	0.00	H
ATOM	1242	HG	LEU	101	5.687	81.272	-5.367	1.00	0.00	H
ATOM	1243	HD11	LEU	101	8.156	81.391	-5.562	1.00	0.00	H
ATOM	1244	HD12	LEU	101	7.440	80.418	-6.832	1.00	0.00	H
ATOM	1245	HD13	LEU	101	8.004	82.049	-7.187	1.00	0.00	H
ATOM	1246	HD21	LEU	101	6.924	83.264	-4.518	1.00	0.00	H
ATOM	1247	HD22	LEU	101	6.676	84.092	-6.049	1.00	0.00	H
ATOM	1248	HD23	LEU	101	5.308	83.715	-5.006	1.00	0.00	H
ATOM	1249	N	PRO	102	2.733	83.603	-5.929	1.00	0.00	N
ATOM	1250	CA	PRO	102	2.142	84.241	-4.769	1.00	0.00	C
ATOM	1251	C	PRO	102	0.667	83.943	-4.589	1.00	0.00	C
ATOM	1252	O	PRO	102	0.196	83.758	-3.470	1.00	0.00	O
ATOM	1253	CB	PRO	102	2.381	85.744	-4.995	1.00	0.00	C
ATOM	1254	CG	PRO	102	2.466	85.906	-6.508	1.00	0.00	C
ATOM	1255	CD	PRO	102	3.026	84.567	-6.995	1.00	0.00	C
ATOM	1256	HA	PRO	102	2.663	83.934	-3.858	1.00	0.00	H
ATOM	1257	HB2	PRO	102	1.617	86.381	-4.546	1.00	0.00	H
ATOM	1258	HB3	PRO	102	3.334	86.026	-4.546	1.00	0.00	H
ATOM	1259	HG2	PRO	102	1.467	86.066	-6.916	1.00	0.00	H
ATOM	1260	HG3	PRO	102	3.081	86.755	-6.808	1.00	0.00	H
ATOM	1261	HD3	PRO	102	4.104	84.662	-7.119	1.00	0.00	H
ATOM	1262	HD2	PRO	102	2.597	84.272	-7.954	1.00	0.00	H
ATOM	1263	N	ILE	103	-0.038	83.840	-5.711	1.00	0.00	N
ATOM	1264	CA	ILE	103	-1.452	83.556	-5.762	1.00	0.00	C
ATOM	1265	C	ILE	103	-1.740	82.128	-5.260	1.00	0.00	C
ATOM	1266	O	ILE	103	-2.790	81.872	-4.673	1.00	0.00	O
ATOM	1267	CB	ILE	103	-1.952	83.749	-7.227	1.00	0.00	C
ATOM	1268	CG1	ILE	103	-1.431	85.041	-7.908	1.00	0.00	C
ATOM	1269	CG2	ILE	103	-3.487	83.661	-7.347	1.00	0.00	C
ATOM	1270	CD1	ILE	103	-1.786	86.347	-7.177	1.00	0.00	C
ATOM	1271	H	ILE	103	0.448	83.933	-6.589	1.00	0.00	H
ATOM	1272	HA	ILE	103	-1.970	84.254	-5.101	1.00	0.00	H
ATOM	1273	HB	ILE	103	-1.549	82.923	-7.815	1.00	0.00	H
ATOM	1274	HG12	ILE	103	-0.350	84.985	-8.039	1.00	0.00	H
ATOM	1275	HG13	ILE	103	-1.819	85.093	-8.924	1.00	0.00	H
ATOM	1276	HG21	ILE	103	-3.820	83.856	-8.366	1.00	0.00	H
ATOM	1277	HG22	ILE	103	-3.855	82.673	-7.071	1.00	0.00	H
ATOM	1278	HG23	ILE	103	-3.978	84.383	-6.694	1.00	0.00	H
ATOM	1279	HD11	ILE	103	-1.402	87.210	-7.722	1.00	0.00	H
ATOM	1280	HD12	ILE	103	-2.864	86.475	-7.080	1.00	0.00	H
ATOM	1281	HD13	ILE	103	-1.354	86.379	-6.176	1.00	0.00	H
ATOM	1282	N	TYR	104	-0.783	81.216	-5.465	1.00	0.00	N
ATOM	1283	CA	TYR	104	-0.787	79.878	-4.909	1.00	0.00	C
ATOM	1284	C	TYR	104	-0.548	79.878	-3.410	1.00	0.00	C
ATOM	1285	O	TYR	104	-1.343	79.314	-2.664	1.00	0.00	O
ATOM	1286	CB	TYR	104	0.254	78.976	-5.611	1.00	0.00	C
ATOM	1287	CG	TYR	104	-0.273	78.211	-6.817	1.00	0.00	C
ATOM	1288	CD1	TYR	104	-1.079	78.844	-7.788	1.00	0.00	C
ATOM	1289	CD2	TYR	104	0.030	76.841	-6.955	1.00	0.00	C
ATOM	1290	CE1	TYR	104	-1.567	78.115	-8.887	1.00	0.00	C
ATOM	1291	CE2	TYR	104	-0.452	76.109	-8.055	1.00	0.00	C
ATOM	1292	CZ	TYR	104	-1.243	76.751	-9.026	1.00	0.00	C
ATOM	1293	OH	TYR	104	-1.684	76.064	-10.116	1.00	0.00	O
ATOM	1294	H	TYR	104	0.012	81.487	-6.034	1.00	0.00	H
ATOM	1295	HA	TYR	104	-1.776	79.437	-5.056	1.00	0.00	H
ATOM	1296	HB2	TYR	104	1.131	79.549	-5.911	1.00	0.00	H
ATOM	1297	HB3	TYR	104	0.631	78.232	-4.904	1.00	0.00	H
ATOM	1298	HD1	TYR	104	-1.324	79.891	-7.696	1.00	0.00	H
ATOM	1299	HD2	TYR	104	0.626	76.334	-6.207	1.00	0.00	H
ATOM	1300	HE1	TYR	104	-2.177	78.618	-9.619	1.00	0.00	H
ATOM	1301	HE2	TYR	104	-0.203	75.059	-8.128	1.00	0.00	H

ATOM	1302	HH	TYR	104	-1.461	75.128	-10.057	1.00	0.00	H
ATOM	1303	N	THR	105	0.505	80.561	-2.972	1.00	0.00	N
ATOM	1304	CA	THR	105	0.926	80.594	-1.580	1.00	0.00	C
ATOM	1305	C	THR	105	-0.147	81.177	-0.652	1.00	0.00	C
ATOM	1306	O	THR	105	-0.408	80.650	0.433	1.00	0.00	O
ATOM	1307	CB	THR	105	2.227	81.447	-1.519	1.00	0.00	C
ATOM	1308	OG1	THR	105	3.247	80.847	-2.302	1.00	0.00	O
ATOM	1309	CG2	THR	105	2.814	81.634	-0.111	1.00	0.00	C
ATOM	1310	H	THR	105	1.094	81.024	-3.648	1.00	0.00	H
ATOM	1311	HA	THR	105	1.147	79.587	-1.238	1.00	0.00	H
ATOM	1312	HB	THR	105	2.035	82.439	-1.931	1.00	0.00	H
ATOM	1313	HG1	THR	105	3.010	80.888	-3.223	1.00	0.00	H
ATOM	1314	HG21	THR	105	3.780	82.138	-0.149	1.00	0.00	H
ATOM	1315	HG22	THR	105	2.164	82.238	0.523	1.00	0.00	H
ATOM	1316	HG23	THR	105	2.956	80.679	0.388	1.00	0.00	H
ATOM	1317	N	LEU	106	-0.818	82.231	-1.122	1.00	0.00	N
ATOM	1318	CA	LEU	106	-1.919	82.861	-0.419	1.00	0.00	C
ATOM	1319	C	LEU	106	-3.156	81.977	-0.336	1.00	0.00	C
ATOM	1320	O	LEU	106	-3.900	82.046	0.636	1.00	0.00	O
ATOM	1321	CB	LEU	106	-2.294	84.176	-1.142	1.00	0.00	C
ATOM	1322	CG	LEU	106	-1.253	85.311	-1.003	1.00	0.00	C
ATOM	1323	CD1	LEU	106	-1.494	86.403	-2.058	1.00	0.00	C
ATOM	1324	CD2	LEU	106	-1.248	85.925	0.407	1.00	0.00	C
ATOM	1325	H	LEU	106	-0.538	82.631	-2.012	1.00	0.00	H
ATOM	1326	HA	LEU	106	-1.612	83.090	0.601	1.00	0.00	H
ATOM	1327	HB2	LEU	106	-2.461	83.956	-2.198	1.00	0.00	H
ATOM	1328	HB3	LEU	106	-3.253	84.543	-0.768	1.00	0.00	H
ATOM	1329	HG	LEU	106	-0.259	84.901	-1.185	1.00	0.00	H
ATOM	1330	HD11	LEU	106	-0.740	87.188	-1.989	1.00	0.00	H
ATOM	1331	HD12	LEU	106	-1.446	85.994	-3.067	1.00	0.00	H
ATOM	1332	HD13	LEU	106	-2.472	86.868	-1.931	1.00	0.00	H
ATOM	1333	HD21	LEU	106	-0.517	86.730	0.484	1.00	0.00	H
ATOM	1334	HD22	LEU	106	-2.223	86.339	0.667	1.00	0.00	H
ATOM	1335	HD23	LEU	106	-0.989	85.190	1.170	1.00	0.00	H
ATOM	1336	N	ALA	107	-3.356	81.119	-1.335	1.00	0.00	N
ATOM	1337	CA	ALA	107	-4.453	80.179	-1.330	1.00	0.00	C
ATOM	1338	C	ALA	107	-4.198	79.000	-0.411	1.00	0.00	C
ATOM	1339	O	ALA	107	-5.113	78.535	0.267	1.00	0.00	O
ATOM	1340	CB	ALA	107	-4.661	79.653	-2.762	1.00	0.00	C
ATOM	1341	H	ALA	107	-2.669	81.061	-2.073	1.00	0.00	H
ATOM	1342	HA	ALA	107	-5.368	80.680	-1.005	1.00	0.00	H
ATOM	1343	HB1	ALA	107	-5.498	78.960	-2.798	1.00	0.00	H
ATOM	1344	HB2	ALA	107	-4.880	80.470	-3.449	1.00	0.00	H
ATOM	1345	HB3	ALA	107	-3.792	79.120	-3.143	1.00	0.00	H
ATOM	1346	N	GLN	108	-2.946	78.545	-0.382	1.00	0.00	N
ATOM	1347	CA	GLN	108	-2.492	77.468	0.468	1.00	0.00	C
ATOM	1348	C	GLN	108	-2.620	77.817	1.946	1.00	0.00	C
ATOM	1349	O	GLN	108	-3.259	77.092	2.711	1.00	0.00	O
ATOM	1350	CB	GLN	108	-1.040	77.104	0.101	1.00	0.00	C
ATOM	1351	CG	GLN	108	-0.923	76.424	-1.284	1.00	0.00	C
ATOM	1352	CD	GLN	108	0.473	76.513	-1.897	1.00	0.00	C
ATOM	1353	OE1	GLN	108	1.353	77.195	-1.390	1.00	0.00	O
ATOM	1354	NE2	GLN	108	0.709	75.828	-3.003	1.00	0.00	N
ATOM	1355	H	GLN	108	-2.271	78.962	-1.015	1.00	0.00	H
ATOM	1356	HA	GLN	108	-3.124	76.598	0.286	1.00	0.00	H
ATOM	1357	HB2	GLN	108	-0.424	78.002	0.150	1.00	0.00	H
ATOM	1358	HB3	GLN	108	-0.629	76.421	0.845	1.00	0.00	H
ATOM	1359	HG2	GLN	108	-1.220	75.379	-1.206	1.00	0.00	H
ATOM	1360	HG3	GLN	108	-1.610	76.877	-1.995	1.00	0.00	H
ATOM	1361	HE22	GLN	108	1.640	75.871	-3.410	1.00	0.00	H
ATOM	1362	HE21	GLN	108	-0.052	75.264	-3.431	1.00	0.00	H
ATOM	1363	N	THR	109	-2.068	78.971	2.321	1.00	0.00	N
ATOM	1364	CA	THR	109	-2.168	79.511	3.665	1.00	0.00	C

ATOM	1365	C	THR	109	-3.603	79.791	4.117	1.00	0.00	C
ATOM	1366	O	THR	109	-3.967	79.454	5.245	1.00	0.00	O
ATOM	1367	CB	THR	109	-1.237	80.744	3.803	1.00	0.00	C
ATOM	1368	OG1	THR	109	-1.448	81.685	2.768	1.00	0.00	O
ATOM	1369	CG2	THR	109	0.257	80.386	3.808	1.00	0.00	C
ATOM	1370	H	THR	109	-1.589	79.524	1.622	1.00	0.00	H
ATOM	1371	HA	THR	109	-1.796	78.746	4.350	1.00	0.00	H
ATOM	1372	HB	THR	109	-1.463	81.246	4.747	1.00	0.00	H
ATOM	1373	HG1	THR	109	-1.057	81.347	1.965	1.00	0.00	H
ATOM	1374	HG21	THR	109	0.875	81.281	3.882	1.00	0.00	H
ATOM	1375	HG22	THR	109	0.501	79.760	4.665	1.00	0.00	H
ATOM	1376	HG23	THR	109	0.555	79.848	2.907	1.00	0.00	H
ATOM	1377	N	LEU	110	-4.433	80.324	3.216	1.00	0.00	N
ATOM	1378	CA	LEU	110	-5.828	80.610	3.495	1.00	0.00	C
ATOM	1379	C	LEU	110	-6.677	79.364	3.732	1.00	0.00	C
ATOM	1380	O	LEU	110	-7.566	79.370	4.582	1.00	0.00	O
ATOM	1381	CB	LEU	110	-6.417	81.450	2.334	1.00	0.00	C
ATOM	1382	CG	LEU	110	-7.906	81.862	2.440	1.00	0.00	C
ATOM	1383	CD1	LEU	110	-8.196	82.714	3.687	1.00	0.00	C
ATOM	1384	CD2	LEU	110	-8.348	82.603	1.169	1.00	0.00	C
ATOM	1385	H	LEU	110	-4.048	80.650	2.338	1.00	0.00	H
ATOM	1386	HA	LEU	110	-5.864	81.210	4.407	1.00	0.00	H
ATOM	1387	HB2	LEU	110	-5.827	82.363	2.243	1.00	0.00	H
ATOM	1388	HB3	LEU	110	-6.274	80.903	1.401	1.00	0.00	H
ATOM	1389	HG	LEU	110	-8.522	80.963	2.500	1.00	0.00	H
ATOM	1390	HD11	LEU	110	-9.241	83.024	3.717	1.00	0.00	H
ATOM	1391	HD12	LEU	110	-8.009	82.159	4.607	1.00	0.00	H
ATOM	1392	HD13	LEU	110	-7.582	83.614	3.710	1.00	0.00	H
ATOM	1393	HD21	LEU	110	-9.406	82.861	1.213	1.00	0.00	H
ATOM	1394	HD22	LEU	110	-7.783	83.526	1.031	1.00	0.00	H
ATOM	1395	HD23	LEU	110	-8.199	81.985	0.283	1.00	0.00	H
ATOM	1396	N	GLY	111	-6.368	78.272	3.038	1.00	0.00	N
ATOM	1397	CA	GLY	111	-7.166	77.069	3.151	1.00	0.00	C
ATOM	1398	C	GLY	111	-6.800	76.276	4.393	1.00	0.00	C
ATOM	1399	O	GLY	111	-7.673	75.737	5.070	1.00	0.00	O
ATOM	1400	H	GLY	111	-5.618	78.312	2.359	1.00	0.00	H
ATOM	1401	HA2	GLY	111	-8.237	77.285	3.134	1.00	0.00	H
ATOM	1402	HA3	GLY	111	-6.954	76.443	2.287	1.00	0.00	H
ATOM	1403	N	ALA	112	-5.516	76.302	4.753	1.00	0.00	N
ATOM	1404	CA	ALA	112	-5.023	75.760	6.006	1.00	0.00	C
ATOM	1405	C	ALA	112	-5.516	76.532	7.217	1.00	0.00	C
ATOM	1406	O	ALA	112	-5.770	75.947	8.265	1.00	0.00	O
ATOM	1407	CB	ALA	112	-3.492	75.816	5.957	1.00	0.00	C
ATOM	1408	H	ALA	112	-4.857	76.785	4.155	1.00	0.00	H
ATOM	1409	HA	ALA	112	-5.350	74.722	6.094	1.00	0.00	H
ATOM	1410	HB1	ALA	112	-3.061	75.307	6.817	1.00	0.00	H
ATOM	1411	HB2	ALA	112	-3.100	75.323	5.072	1.00	0.00	H
ATOM	1412	HB3	ALA	112	-3.119	76.841	5.954	1.00	0.00	H
ATOM	1413	N	PHE	113	-5.689	77.842	7.048	1.00	0.00	N
ATOM	1414	CA	PHE	113	-6.297	78.688	8.045	1.00	0.00	C
ATOM	1415	C	PHE	113	-7.751	78.317	8.317	1.00	0.00	C
ATOM	1416	O	PHE	113	-8.120	78.000	9.448	1.00	0.00	O
ATOM	1417	CB	PHE	113	-6.156	80.178	7.639	1.00	0.00	C
ATOM	1418	CG	PHE	113	-6.731	81.191	8.620	1.00	0.00	C
ATOM	1419	CD1	PHE	113	-5.947	81.670	9.690	1.00	0.00	C
ATOM	1420	CD2	PHE	113	-8.057	81.653	8.471	1.00	0.00	C
ATOM	1421	CE1	PHE	113	-6.488	82.583	10.615	1.00	0.00	C
ATOM	1422	CE2	PHE	113	-8.598	82.567	9.396	1.00	0.00	C
ATOM	1423	CZ	PHE	113	-7.814	83.030	10.470	1.00	0.00	C
ATOM	1424	H	PHE	113	-5.404	78.260	6.171	1.00	0.00	H
ATOM	1425	HA	PHE	113	-5.752	78.550	8.982	1.00	0.00	H
ATOM	1426	HB2	PHE	113	-5.100	80.414	7.505	1.00	0.00	H
ATOM	1427	HB3	PHE	113	-6.618	80.357	6.671	1.00	0.00	H

ATOM	1428	HD1	PHE	113	-4.928	81.341	9.812	1.00	0.00	H
ATOM	1429	HD2	PHE	113	-8.672	81.298	7.658	1.00	0.00	H
ATOM	1430	HE1	PHE	113	-5.886	82.938	11.439	1.00	0.00	H
ATOM	1431	HE2	PHE	113	-9.617	82.908	9.288	1.00	0.00	H
ATOM	1432	HZ	PHE	113	-8.231	83.726	11.184	1.00	0.00	H
ATOM	1433	N	LEU	114	-8.564	78.298	7.260	1.00	0.00	N
ATOM	1434	CA	LEU	114	-9.985	78.030	7.382	1.00	0.00	C
ATOM	1435	C	LEU	114	-10.287	76.585	7.759	1.00	0.00	C
ATOM	1436	O	LEU	114	-11.260	76.323	8.457	1.00	0.00	O
ATOM	1437	CB	LEU	114	-10.671	78.343	6.031	1.00	0.00	C
ATOM	1438	CG	LEU	114	-10.669	79.835	5.626	1.00	0.00	C
ATOM	1439	CD1	LEU	114	-11.023	79.994	4.140	1.00	0.00	C
ATOM	1440	CD2	LEU	114	-11.613	80.679	6.498	1.00	0.00	C
ATOM	1441	H	LEU	114	-8.199	78.561	6.351	1.00	0.00	H
ATOM	1442	HA	LEU	114	-10.405	78.664	8.166	1.00	0.00	H
ATOM	1443	HB2	LEU	114	-10.182	77.755	5.252	1.00	0.00	H
ATOM	1444	HB3	LEU	114	-11.705	77.990	6.054	1.00	0.00	H
ATOM	1445	HG	LEU	114	-9.664	80.236	5.754	1.00	0.00	H
ATOM	1446	HD11	LEU	114	-10.987	81.040	3.835	1.00	0.00	H
ATOM	1447	HD12	LEU	114	-10.319	79.449	3.509	1.00	0.00	H
ATOM	1448	HD13	LEU	114	-12.024	79.617	3.926	1.00	0.00	H
ATOM	1449	HD21	LEU	114	-11.609	81.724	6.184	1.00	0.00	H
ATOM	1450	HD22	LEU	114	-12.641	80.321	6.438	1.00	0.00	H
ATOM	1451	HD23	LEU	114	-11.318	80.663	7.548	1.00	0.00	H
ATOM	1452	N	GLY	115	-9.411	75.655	7.382	1.00	0.00	N
ATOM	1453	CA	GLY	115	-9.589	74.253	7.692	1.00	0.00	C
ATOM	1454	C	GLY	115	-9.176	73.913	9.115	1.00	0.00	C
ATOM	1455	O	GLY	115	-9.700	72.960	9.685	1.00	0.00	O
ATOM	1456	H	GLY	115	-8.640	75.919	6.776	1.00	0.00	H
ATOM	1457	HA2	GLY	115	-10.617	73.931	7.529	1.00	0.00	H
ATOM	1458	HA3	GLY	115	-8.940	73.677	7.034	1.00	0.00	H
ATOM	1459	N	ALA	116	-8.313	74.735	9.720	1.00	0.00	N
ATOM	1460	CA	ALA	116	-8.042	74.675	11.143	1.00	0.00	C
ATOM	1461	C	ALA	116	-9.199	75.213	11.965	1.00	0.00	C
ATOM	1462	O	ALA	116	-9.584	74.605	12.963	1.00	0.00	O
ATOM	1463	CB	ALA	116	-6.782	75.498	11.460	1.00	0.00	C
ATOM	1464	H	ALA	116	-7.930	75.512	9.199	1.00	0.00	H
ATOM	1465	HA	ALA	116	-7.864	73.638	11.436	1.00	0.00	H
ATOM	1466	HB1	ALA	116	-6.561	75.479	12.529	1.00	0.00	H
ATOM	1467	HB2	ALA	116	-5.914	75.096	10.940	1.00	0.00	H
ATOM	1468	HB3	ALA	116	-6.887	76.545	11.175	1.00	0.00	H
ATOM	1469	N	GLY	117	-9.806	76.295	11.474	1.00	0.00	N
ATOM	1470	CA	GLY	117	-10.964	76.894	12.101	1.00	0.00	C
ATOM	1471	C	GLY	117	-12.196	76.007	12.034	1.00	0.00	C
ATOM	1472	O	GLY	117	-12.975	75.957	12.980	1.00	0.00	O
ATOM	1473	H	GLY	117	-9.396	76.765	10.676	1.00	0.00	H
ATOM	1474	HA2	GLY	117	-10.744	77.075	13.150	1.00	0.00	H
ATOM	1475	HA3	GLY	117	-11.182	77.856	11.639	1.00	0.00	H
ATOM	1476	N	ILE	118	-12.317	75.236	10.951	1.00	0.00	N
ATOM	1477	CA	ILE	118	-13.329	74.210	10.808	1.00	0.00	C
ATOM	1478	C	ILE	118	-13.170	73.057	11.775	1.00	0.00	C
ATOM	1479	O	ILE	118	-14.147	72.652	12.395	1.00	0.00	O
ATOM	1480	CB	ILE	118	-13.406	73.742	9.316	1.00	0.00	C
ATOM	1481	CG1	ILE	118	-14.066	74.840	8.443	1.00	0.00	C
ATOM	1482	CG2	ILE	118	-14.154	72.403	9.097	1.00	0.00	C
ATOM	1483	CD1	ILE	118	-13.792	74.711	6.936	1.00	0.00	C
ATOM	1484	H	ILE	118	-11.690	75.407	10.175	1.00	0.00	H
ATOM	1485	HA	ILE	118	-14.291	74.665	11.056	1.00	0.00	H
ATOM	1486	HB	ILE	118	-12.384	73.591	8.970	1.00	0.00	H
ATOM	1487	HG12	ILE	118	-15.143	74.846	8.619	1.00	0.00	H
ATOM	1488	HG13	ILE	118	-13.726	75.825	8.761	1.00	0.00	H
ATOM	1489	HG21	ILE	118	-14.257	72.155	8.043	1.00	0.00	H
ATOM	1490	HG22	ILE	118	-13.633	71.561	9.556	1.00	0.00	H

ATOM	1491	HG23	ILE	118	-15.158	72.437	9.524	1.00	0.00	H
ATOM	1492	HD11	ILE	118	-14.239	75.546	6.397	1.00	0.00	H
ATOM	1493	HD12	ILE	118	-12.723	74.725	6.728	1.00	0.00	H
ATOM	1494	HD13	ILE	118	-14.207	73.795	6.518	1.00	0.00	H
ATOM	1495	N	VAL	119	-11.951	72.541	11.916	1.00	0.00	N
ATOM	1496	CA	VAL	119	-11.684	71.466	12.850	1.00	0.00	C
ATOM	1497	C	VAL	119	-11.982	71.868	14.292	1.00	0.00	C
ATOM	1498	O	VAL	119	-12.666	71.158	15.024	1.00	0.00	O
ATOM	1499	CB	VAL	119	-10.221	70.961	12.646	1.00	0.00	C
ATOM	1500	CG1	VAL	119	-9.652	70.101	13.795	1.00	0.00	C
ATOM	1501	CG2	VAL	119	-10.096	70.164	11.336	1.00	0.00	C
ATOM	1502	H	VAL	119	-11.188	72.917	11.371	1.00	0.00	H
ATOM	1503	HA	VAL	119	-12.364	70.640	12.622	1.00	0.00	H
ATOM	1504	HB	VAL	119	-9.577	71.837	12.563	1.00	0.00	H
ATOM	1505	HG11	VAL	119	-8.645	69.756	13.567	1.00	0.00	H
ATOM	1506	HG12	VAL	119	-9.582	70.650	14.735	1.00	0.00	H
ATOM	1507	HG13	VAL	119	-10.268	69.220	13.973	1.00	0.00	H
ATOM	1508	HG21	VAL	119	-10.510	70.705	10.487	1.00	0.00	H
ATOM	1509	HG22	VAL	119	-9.053	69.941	11.111	1.00	0.00	H
ATOM	1510	HG23	VAL	119	-10.629	69.214	11.399	1.00	0.00	H
ATOM	1511	N	PHE	120	-11.547	73.068	14.654	1.00	0.00	N
ATOM	1512	CA	PHE	120	-11.826	73.658	15.945	1.00	0.00	C
ATOM	1513	C	PHE	120	-13.292	73.979	16.184	1.00	0.00	C
ATOM	1514	O	PHE	120	-13.737	74.046	17.323	1.00	0.00	O
ATOM	1515	CB	PHE	120	-10.998	74.968	16.016	1.00	0.00	C
ATOM	1516	CG	PHE	120	-10.988	75.711	17.343	1.00	0.00	C
ATOM	1517	CD1	PHE	120	-10.069	75.353	18.351	1.00	0.00	C
ATOM	1518	CD2	PHE	120	-11.887	76.776	17.570	1.00	0.00	C
ATOM	1519	CE1	PHE	120	-10.049	76.052	19.573	1.00	0.00	C
ATOM	1520	CE2	PHE	120	-11.868	77.473	18.793	1.00	0.00	C
ATOM	1521	CZ	PHE	120	-10.952	77.109	19.797	1.00	0.00	C
ATOM	1522	H	PHE	120	-11.018	73.620	13.986	1.00	0.00	H
ATOM	1523	HA	PHE	120	-11.492	72.977	16.731	1.00	0.00	H
ATOM	1524	HB2	PHE	120	-9.959	74.759	15.763	1.00	0.00	H
ATOM	1525	HB3	PHE	120	-11.343	75.660	15.246	1.00	0.00	H
ATOM	1526	HD1	PHE	120	-9.379	74.538	18.197	1.00	0.00	H
ATOM	1527	HD2	PHE	120	-12.607	77.055	16.813	1.00	0.00	H
ATOM	1528	HE1	PHE	120	-9.348	75.776	20.345	1.00	0.00	H
ATOM	1529	HE2	PHE	120	-12.565	78.280	18.969	1.00	0.00	H
ATOM	1530	HZ	PHE	120	-10.943	77.637	20.741	1.00	0.00	H
ATOM	1531	N	GLY	121	-14.055	74.094	15.106	1.00	0.00	N
ATOM	1532	CA	GLY	121	-15.456	74.403	15.170	1.00	0.00	C
ATOM	1533	C	GLY	121	-16.342	73.168	15.105	1.00	0.00	C
ATOM	1534	O	GLY	121	-17.562	73.288	15.146	1.00	0.00	O
ATOM	1535	H	GLY	121	-13.636	74.034	14.189	1.00	0.00	H
ATOM	1536	HA2	GLY	121	-15.713	74.982	16.060	1.00	0.00	H
ATOM	1537	HA3	GLY	121	-15.700	75.036	14.317	1.00	0.00	H
ATOM	1538	N	LEU	122	-15.720	71.991	15.031	1.00	0.00	N
ATOM	1539	CA	LEU	122	-16.360	70.704	15.207	1.00	0.00	C
ATOM	1540	C	LEU	122	-15.924	70.053	16.531	1.00	0.00	C
ATOM	1541	O	LEU	122	-16.689	69.295	17.114	1.00	0.00	O
ATOM	1542	CB	LEU	122	-15.931	69.775	14.046	1.00	0.00	C
ATOM	1543	CG	LEU	122	-16.464	70.189	12.655	1.00	0.00	C
ATOM	1544	CD1	LEU	122	-15.662	69.508	11.535	1.00	0.00	C
ATOM	1545	CD2	LEU	122	-17.965	69.896	12.503	1.00	0.00	C
ATOM	1546	H	LEU	122	-14.717	72.010	14.923	1.00	0.00	H
ATOM	1547	HA	LEU	122	-17.448	70.793	15.234	1.00	0.00	H
ATOM	1548	HB2	LEU	122	-14.841	69.735	14.019	1.00	0.00	H
ATOM	1549	HB3	LEU	122	-16.251	68.753	14.257	1.00	0.00	H
ATOM	1550	HG	LEU	122	-16.329	71.264	12.534	1.00	0.00	H
ATOM	1551	HD11	LEU	122	-16.019	69.818	10.553	1.00	0.00	H
ATOM	1552	HD12	LEU	122	-14.606	69.773	11.596	1.00	0.00	H
ATOM	1553	HD13	LEU	122	-15.740	68.422	11.595	1.00	0.00	H

ATOM	1554	HD21	LEU	122	-18.326	70.189	11.517	1.00	0.00	H
ATOM	1555	HD22	LEU	122	-18.182	68.836	12.636	1.00	0.00	H
ATOM	1556	HD23	LEU	122	-18.555	70.449	13.235	1.00	0.00	H
ATOM	1557	N	TYR	123	-14.700	70.332	17.001	1.00	0.00	N
ATOM	1558	CA	TYR	123	-14.149	69.813	18.255	1.00	0.00	C
ATOM	1559	C	TYR	123	-14.251	70.837	19.404	1.00	0.00	C
ATOM	1560	O	TYR	123	-13.689	70.571	20.464	1.00	0.00	O
ATOM	1561	CB	TYR	123	-12.655	69.460	18.037	1.00	0.00	C
ATOM	1562	CG	TYR	123	-12.397	68.103	17.402	1.00	0.00	C
ATOM	1563	CD1	TYR	123	-12.109	67.990	16.027	1.00	0.00	C
ATOM	1564	CD2	TYR	123	-12.422	66.942	18.203	1.00	0.00	C
ATOM	1565	CE1	TYR	123	-11.828	66.734	15.460	1.00	0.00	C
ATOM	1566	CE2	TYR	123	-12.152	65.682	17.635	1.00	0.00	C
ATOM	1567	CZ	TYR	123	-11.845	65.581	16.266	1.00	0.00	C
ATOM	1568	OH	TYR	123	-11.546	64.370	15.722	1.00	0.00	O
ATOM	1569	H	TYR	123	-14.088	70.897	16.428	1.00	0.00	H
ATOM	1570	HA	TYR	123	-14.684	68.919	18.585	1.00	0.00	H
ATOM	1571	HB2	TYR	123	-12.156	70.242	17.464	1.00	0.00	H
ATOM	1572	HB3	TYR	123	-12.118	69.437	18.986	1.00	0.00	H
ATOM	1573	HD1	TYR	123	-12.101	68.864	15.400	1.00	0.00	H
ATOM	1574	HD2	TYR	123	-12.647	67.012	19.259	1.00	0.00	H
ATOM	1575	HE1	TYR	123	-11.609	66.658	14.406	1.00	0.00	H
ATOM	1576	HE2	TYR	123	-12.171	64.797	18.253	1.00	0.00	H
ATOM	1577	HH	TYR	123	-10.755	64.405	15.189	1.00	0.00	H
ATOM	1578	N	TYR	124	-14.933	71.981	19.204	1.00	0.00	N
ATOM	1579	CA	TYR	124	-15.050	73.150	20.099	1.00	0.00	C
ATOM	1580	C	TYR	124	-15.201	72.786	21.578	1.00	0.00	C
ATOM	1581	O	TYR	124	-14.397	73.179	22.422	1.00	0.00	O
ATOM	1582	CB	TYR	124	-16.164	74.095	19.598	1.00	0.00	C
ATOM	1583	CG	TYR	124	-16.205	75.426	20.326	1.00	0.00	C
ATOM	1584	CD1	TYR	124	-15.238	76.412	20.043	1.00	0.00	C
ATOM	1585	CD2	TYR	124	-17.167	75.652	21.332	1.00	0.00	C
ATOM	1586	CE1	TYR	124	-15.218	77.610	20.782	1.00	0.00	C
ATOM	1587	CE2	TYR	124	-17.145	76.849	22.072	1.00	0.00	C
ATOM	1588	CZ	TYR	124	-16.162	77.823	21.804	1.00	0.00	C
ATOM	1589	OH	TYR	124	-16.090	78.956	22.558	1.00	0.00	O
ATOM	1590	H	TYR	124	-15.391	72.067	18.311	1.00	0.00	H
ATOM	1591	HA	TYR	124	-14.101	73.682	20.014	1.00	0.00	H
ATOM	1592	HB2	TYR	124	-16.035	74.302	18.536	1.00	0.00	H
ATOM	1593	HB3	TYR	124	-17.136	73.606	19.689	1.00	0.00	H
ATOM	1594	HD1	TYR	124	-14.490	76.234	19.284	1.00	0.00	H
ATOM	1595	HD2	TYR	124	-17.903	74.892	21.565	1.00	0.00	H
ATOM	1596	HE1	TYR	124	-14.460	78.353	20.584	1.00	0.00	H
ATOM	1597	HE2	TYR	124	-17.869	76.989	22.861	1.00	0.00	H
ATOM	1598	HH	TYR	124	-16.730	78.966	23.257	1.00	0.00	H
ATOM	1599	N	ASP	125	-16.200	71.955	21.861	1.00	0.00	N
ATOM	1600	CA	ASP	125	-16.570	71.492	23.185	1.00	0.00	C
ATOM	1601	C	ASP	125	-15.494	70.713	23.900	1.00	0.00	C
ATOM	1602	O	ASP	125	-15.189	70.957	25.066	1.00	0.00	O
ATOM	1603	CB	ASP	125	-17.860	70.643	23.117	1.00	0.00	C
ATOM	1604	CG	ASP	125	-19.039	71.391	22.509	1.00	0.00	C
ATOM	1605	OD1	ASP	125	-20.013	71.620	23.254	1.00	0.00	O
ATOM	1606	OD2	ASP	125	-18.915	71.746	21.314	1.00	0.00	O1-
ATOM	1607	H	ASP	125	-16.932	71.809	21.165	1.00	0.00	H
ATOM	1608	HA	ASP	125	-16.786	72.377	23.788	1.00	0.00	H
ATOM	1609	HB2	ASP	125	-17.702	69.741	22.526	1.00	0.00	H
ATOM	1610	HB3	ASP	125	-18.142	70.309	24.114	1.00	0.00	H
ATOM	1611	N	ALA	126	-14.868	69.810	23.153	1.00	0.00	N
ATOM	1612	CA	ALA	126	-13.852	68.924	23.668	1.00	0.00	C
ATOM	1613	C	ALA	126	-12.539	69.628	23.959	1.00	0.00	C
ATOM	1614	O	ALA	126	-11.719	69.096	24.699	1.00	0.00	O
ATOM	1615	CB	ALA	126	-13.608	67.816	22.629	1.00	0.00	C
ATOM	1616	H	ALA	126	-15.100	69.789	22.171	1.00	0.00	H

ATOM	1617	HA	ALA	126	-14.212	68.473	24.595	1.00	0.00	H
ATOM	1618	HB1	ALA	126	-12.939	67.054	23.027	1.00	0.00	H
ATOM	1619	HB2	ALA	126	-14.540	67.315	22.366	1.00	0.00	H
ATOM	1620	HB3	ALA	126	-13.165	68.207	21.713	1.00	0.00	H
ATOM	1621	N	ILE	127	-12.353	70.832	23.421	1.00	0.00	N
ATOM	1622	CA	ILE	127	-11.259	71.692	23.802	1.00	0.00	C
ATOM	1623	C	ILE	127	-11.484	72.241	25.203	1.00	0.00	C
ATOM	1624	O	ILE	127	-10.630	72.097	26.080	1.00	0.00	O
ATOM	1625	CB	ILE	127	-11.128	72.901	22.800	1.00	0.00	C
ATOM	1626	CG1	ILE	127	-10.999	72.525	21.298	1.00	0.00	C
ATOM	1627	CG2	ILE	127	-10.071	73.955	23.198	1.00	0.00	C
ATOM	1628	CD1	ILE	127	-9.571	72.470	20.728	1.00	0.00	C
ATOM	1629	H	ILE	127	-13.093	71.212	22.843	1.00	0.00	H
ATOM	1630	HA	ILE	127	-10.322	71.148	23.797	1.00	0.00	H
ATOM	1631	HB	ILE	127	-12.064	73.450	22.862	1.00	0.00	H
ATOM	1632	HG12	ILE	127	-11.497	71.581	21.100	1.00	0.00	H
ATOM	1633	HG13	ILE	127	-11.557	73.259	20.715	1.00	0.00	H
ATOM	1634	HG21	ILE	127	-10.072	74.769	22.476	1.00	0.00	H
ATOM	1635	HG22	ILE	127	-10.281	74.415	24.165	1.00	0.00	H
ATOM	1636	HG23	ILE	127	-9.068	73.531	23.247	1.00	0.00	H
ATOM	1637	HD11	ILE	127	-9.103	73.453	20.710	1.00	0.00	H
ATOM	1638	HD12	ILE	127	-8.933	71.816	21.315	1.00	0.00	H
ATOM	1639	HD13	ILE	127	-9.582	72.101	19.703	1.00	0.00	H
ATOM	1640	N	TRP	128	-12.641	72.882	25.374	1.00	0.00	N
ATOM	1641	CA	TRP	128	-13.017	73.564	26.590	1.00	0.00	C
ATOM	1642	C	TRP	128	-13.103	72.601	27.758	1.00	0.00	C
ATOM	1643	O	TRP	128	-12.366	72.736	28.733	1.00	0.00	O
ATOM	1644	CB	TRP	128	-14.354	74.307	26.396	1.00	0.00	C
ATOM	1645	CG	TRP	128	-14.215	75.679	25.816	1.00	0.00	C
ATOM	1646	CD1	TRP	128	-14.587	76.064	24.575	1.00	0.00	C
ATOM	1647	CD2	TRP	128	-13.658	76.866	26.457	1.00	0.00	C
ATOM	1648	NE1	TRP	128	-14.301	77.405	24.406	1.00	0.00	N
ATOM	1649	CE2	TRP	128	-13.715	77.950	25.530	1.00	0.00	C
ATOM	1650	CE3	TRP	128	-13.111	77.136	27.734	1.00	0.00	C
ATOM	1651	CZ2	TRP	128	-13.241	79.235	25.849	1.00	0.00	C
ATOM	1652	CZ3	TRP	128	-12.633	78.420	28.066	1.00	0.00	C
ATOM	1653	CH2	TRP	128	-12.694	79.468	27.125	1.00	0.00	C
ATOM	1654	H	TRP	128	-13.287	72.923	24.596	1.00	0.00	H
ATOM	1655	HA	TRP	128	-12.241	74.292	26.840	1.00	0.00	H
ATOM	1656	HB2	TRP	128	-15.045	73.718	25.792	1.00	0.00	H
ATOM	1657	HB3	TRP	128	-14.847	74.445	27.361	1.00	0.00	H
ATOM	1658	HD1	TRP	128	-15.041	75.409	23.843	1.00	0.00	H
ATOM	1659	HE1	TRP	128	-14.549	77.911	23.559	1.00	0.00	H
ATOM	1660	HE3	TRP	128	-13.066	76.346	28.474	1.00	0.00	H
ATOM	1661	HZ2	TRP	128	-13.294	80.033	25.124	1.00	0.00	H
ATOM	1662	HZ3	TRP	128	-12.224	78.592	29.052	1.00	0.00	H
ATOM	1663	HH2	TRP	128	-12.325	80.448	27.386	1.00	0.00	H
ATOM	1664	N	HIE	129	-13.888	71.537	27.566	1.00	0.00	N
ATOM	1665	CA	HIE	129	-14.153	70.535	28.584	1.00	0.00	C
ATOM	1666	C	HIE	129	-12.900	69.803	29.039	1.00	0.00	C
ATOM	1667	O	HIE	129	-12.823	69.358	30.184	1.00	0.00	O
ATOM	1668	CB	HIE	129	-15.175	69.494	28.071	1.00	0.00	C
ATOM	1669	CG	HIE	129	-16.535	70.003	27.665	1.00	0.00	C
ATOM	1670	ND1	HIE	129	-17.489	69.148	27.101	1.00	0.00	N
ATOM	1671	CD2	HIE	129	-17.065	71.276	27.727	1.00	0.00	C
ATOM	1672	CE1	HIE	129	-18.515	69.932	26.794	1.00	0.00	C
ATOM	1673	NE2	HIE	129	-18.316	71.204	27.135	1.00	0.00	N
ATOM	1674	H	HIE	129	-14.388	71.469	26.682	1.00	0.00	H
ATOM	1675	HA	HIE	129	-14.571	71.050	29.453	1.00	0.00	H
ATOM	1676	HB2	HIE	129	-14.760	68.969	27.210	1.00	0.00	H
ATOM	1677	HB3	HIE	129	-15.341	68.736	28.838	1.00	0.00	H
ATOM	1678	HD2	HIE	129	-16.638	72.200	28.097	1.00	0.00	H
ATOM	1679	HE1	HIE	129	-19.409	69.584	26.295	1.00	0.00	H

```
ATOM   1680  HE2 HIE   129      -18.945  71.971  26.933  1.00  0.00           H
ATOM   1681  N   PHE   130      -11.918  69.705  28.140  1.00  0.00           N
ATOM   1682  CA  PHE   130      -10.606  69.172  28.423  1.00  0.00           C
ATOM   1683  C   PHE   130       -9.759  70.118  29.252  1.00  0.00           C
ATOM   1684  O   PHE   130       -9.153  69.693  30.235  1.00  0.00           O
ATOM   1685  CB  PHE   130       -9.887  68.829  27.097  1.00  0.00           C
ATOM   1686  CG  PHE   130       -8.472  68.284  27.207  1.00  0.00           C
ATOM   1687  CD1 PHE   130       -8.258  66.930  27.537  1.00  0.00           C
ATOM   1688  CD2 PHE   130       -7.367  69.127  26.958  1.00  0.00           C
ATOM   1689  CE1 PHE   130       -6.947  66.423  27.624  1.00  0.00           C
ATOM   1690  CE2 PHE   130       -6.058  68.616  27.039  1.00  0.00           C
ATOM   1691  CZ  PHE   130       -5.848  67.266  27.374  1.00  0.00           C
ATOM   1692  H   PHE   130      -12.077  70.143  27.244  1.00  0.00           H
ATOM   1693  HA  PHE   130      -10.723  68.248  28.991  1.00  0.00           H
ATOM   1694  HB2 PHE   130      -10.469  68.068  26.577  1.00  0.00           H
ATOM   1695  HB3 PHE   130       -9.867  69.695  26.438  1.00  0.00           H
ATOM   1696  HD1 PHE   130       -9.104  66.278  27.701  1.00  0.00           H
ATOM   1697  HD2 PHE   130       -7.526  70.165  26.702  1.00  0.00           H
ATOM   1698  HE1 PHE   130       -6.779  65.381  27.850  1.00  0.00           H
ATOM   1699  HE2 PHE   130       -5.212  69.258  26.844  1.00  0.00           H
ATOM   1700  HZ  PHE   130       -4.844  66.869  27.420  1.00  0.00           H
ATOM   1701  N   ALA   131       -9.738  71.395  28.869  1.00  0.00           N
ATOM   1702  CA  ALA   131       -8.984  72.422  29.553  1.00  0.00           C
ATOM   1703  C   ALA   131       -9.468  72.658  30.971  1.00  0.00           C
ATOM   1704  O   ALA   131       -8.647  72.835  31.871  1.00  0.00           O
ATOM   1705  CB  ALA   131       -9.059  73.728  28.745  1.00  0.00           C
ATOM   1706  H   ALA   131      -10.329  71.682  28.094  1.00  0.00           H
ATOM   1707  HA  ALA   131       -7.955  72.079  29.608  1.00  0.00           H
ATOM   1708  HB1 ALA   131       -8.466  74.517  29.207  1.00  0.00           H
ATOM   1709  HB2 ALA   131       -8.688  73.588  27.730  1.00  0.00           H
ATOM   1710  HB3 ALA   131      -10.084  74.098  28.673  1.00  0.00           H
ATOM   1711  N   ASP   132      -10.781  72.576  31.165  1.00  0.00           N
ATOM   1712  CA  ASP   132      -11.429  72.729  32.453  1.00  0.00           C
ATOM   1713  C   ASP   132      -11.085  71.598  33.413  1.00  0.00           C
ATOM   1714  O   ASP   132      -10.687  71.813  34.556  1.00  0.00           O
ATOM   1715  CB  ASP   132      -12.975  72.738  32.304  1.00  0.00           C
ATOM   1716  CG  ASP   132      -13.562  73.857  31.451  1.00  0.00           C
ATOM   1717  OD1 ASP   132      -12.957  74.949  31.431  1.00  0.00           O
ATOM   1718  OD2 ASP   132      -14.618  73.587  30.837  1.00  0.00           O1-
ATOM   1719  H   ASP   132      -11.385  72.575  30.343  1.00  0.00           H
ATOM   1720  HA  ASP   132      -11.114  73.676  32.896  1.00  0.00           H
ATOM   1721  HB2 ASP   132      -13.325  71.794  31.884  1.00  0.00           H
ATOM   1722  HB3 ASP   132      -13.441  72.829  33.285  1.00  0.00           H
ATOM   1723  N   ASN   133      -11.166  70.371  32.899  1.00  0.00           N
ATOM   1724  CA  ASN   133      -10.891  69.126  33.604  1.00  0.00           C
ATOM   1725  C   ASN   133       -9.376  68.885  33.837  1.00  0.00           C
ATOM   1726  O   ASN   133       -9.013  67.817  34.343  1.00  0.00           O
ATOM   1727  CB  ASN   133      -11.380  67.961  32.702  1.00  0.00           C
ATOM   1728  CG  ASN   133      -12.764  67.438  33.052  1.00  0.00           C
ATOM   1729  OD1 ASN   133      -12.870  66.480  33.816  1.00  0.00           O
ATOM   1730  ND2 ASN   133      -13.808  67.985  32.446  1.00  0.00           N
ATOM   1731  H   ASN   133      -11.458  70.325  31.930  1.00  0.00           H
ATOM   1732  HA  ASN   133      -11.390  69.118  34.576  1.00  0.00           H
ATOM   1733  HB2 ASN   133      -11.303  68.216  31.648  1.00  0.00           H
ATOM   1734  HB3 ASN   133      -10.735  67.094  32.775  1.00  0.00           H
ATOM   1735 HD22 ASN   133      -14.745  67.688  32.664  1.00  0.00           H
ATOM   1736 HD21 ASN   133      -13.648  68.697  31.731  1.00  0.00           H
ATOM   1737  N   GLN   134       -8.496  69.805  33.408  1.00  0.00           N
ATOM   1738  CA  GLN   134       -7.035  69.689  33.444  1.00  0.00           C
ATOM   1739  C   GLN   134       -6.297  70.939  33.944  1.00  0.00           C
ATOM   1740  O   GLN   134       -5.126  70.816  34.295  1.00  0.00           O
ATOM   1741  CB  GLN   134       -6.484  69.258  32.065  1.00  0.00           C
ATOM   1742  CG  GLN   134       -6.785  67.802  31.655  1.00  0.00           C
```

ATOM	1743	CD	GLN	134	-6.042	66.737	32.465	1.00	0.00	C
ATOM	1744	OE1	GLN	134	-6.408	65.566	32.409	1.00	0.00	O
ATOM	1745	NE2	GLN	134	-5.034	67.096	33.251	1.00	0.00	N
ATOM	1746	H	GLN	134	-8.896	70.621	32.964	1.00	0.00	H
ATOM	1747	HA	GLN	134	-6.776	68.933	34.185	1.00	0.00	H
ATOM	1748	HB2	GLN	134	-6.888	69.929	31.309	1.00	0.00	H
ATOM	1749	HB3	GLN	134	-5.406	69.422	32.020	1.00	0.00	H
ATOM	1750	HG2	GLN	134	-7.857	67.613	31.700	1.00	0.00	H
ATOM	1751	HG3	GLN	134	-6.510	67.668	30.608	1.00	0.00	H
ATOM	1752	HE22	GLN	134	-4.558	66.395	33.797	1.00	0.00	H
ATOM	1753	HE21	GLN	134	-4.754	68.067	33.318	1.00	0.00	H
ATOM	1754	N	LEU	135	-6.940	72.113	33.942	1.00	0.00	N
ATOM	1755	CA	LEU	135	-6.420	73.423	34.354	1.00	0.00	C
ATOM	1756	C	LEU	135	-5.634	74.156	33.245	1.00	0.00	C
ATOM	1757	O	LEU	135	-4.864	75.074	33.529	1.00	0.00	O
ATOM	1758	CB	LEU	135	-5.750	73.438	35.755	1.00	0.00	C
ATOM	1759	CG	LEU	135	-6.582	72.828	36.908	1.00	0.00	C
ATOM	1760	CD1	LEU	135	-5.759	72.801	38.205	1.00	0.00	C
ATOM	1761	CD2	LEU	135	-7.914	73.564	37.133	1.00	0.00	C
ATOM	1762	H	LEU	135	-7.885	72.131	33.579	1.00	0.00	H
ATOM	1763	HA	LEU	135	-7.316	74.037	34.449	1.00	0.00	H
ATOM	1764	HB2	LEU	135	-4.788	72.929	35.693	1.00	0.00	H
ATOM	1765	HB3	LEU	135	-5.505	74.468	36.016	1.00	0.00	H
ATOM	1766	HG	LEU	135	-6.815	71.792	36.662	1.00	0.00	H
ATOM	1767	HD11	LEU	135	-6.316	72.331	39.017	1.00	0.00	H
ATOM	1768	HD12	LEU	135	-4.835	72.237	38.074	1.00	0.00	H
ATOM	1769	HD13	LEU	135	-5.491	73.808	38.526	1.00	0.00	H
ATOM	1770	HD21	LEU	135	-8.448	73.157	37.993	1.00	0.00	H
ATOM	1771	HD22	LEU	135	-7.758	74.628	37.314	1.00	0.00	H
ATOM	1772	HD23	LEU	135	-8.581	73.463	36.276	1.00	0.00	H
ATOM	1773	N	PHE	136	-5.852	73.808	31.966	1.00	0.00	N
ATOM	1774	CA	PHE	136	-5.211	74.477	30.822	1.00	0.00	C
ATOM	1775	C	PHE	136	-5.885	75.821	30.477	1.00	0.00	C
ATOM	1776	O	PHE	136	-5.382	76.537	29.617	1.00	0.00	O
ATOM	1777	CB	PHE	136	-5.230	73.594	29.553	1.00	0.00	C
ATOM	1778	CG	PHE	136	-4.355	72.353	29.593	1.00	0.00	C
ATOM	1779	CD1	PHE	136	-4.924	71.065	29.504	1.00	0.00	C
ATOM	1780	CD2	PHE	136	-2.953	72.488	29.677	1.00	0.00	C
ATOM	1781	CE1	PHE	136	-4.098	69.924	29.525	1.00	0.00	C
ATOM	1782	CE2	PHE	136	-2.128	71.347	29.692	1.00	0.00	C
ATOM	1783	CZ	PHE	136	-2.701	70.063	29.620	1.00	0.00	C
ATOM	1784	H	PHE	136	-6.547	73.093	31.794	1.00	0.00	H
ATOM	1785	HA	PHE	136	-4.172	74.697	31.074	1.00	0.00	H
ATOM	1786	HB2	PHE	136	-6.248	73.332	29.285	1.00	0.00	H
ATOM	1787	HB3	PHE	136	-4.863	74.177	28.705	1.00	0.00	H
ATOM	1788	HD1	PHE	136	-5.993	70.938	29.413	1.00	0.00	H
ATOM	1789	HD2	PHE	136	-2.501	73.469	29.726	1.00	0.00	H
ATOM	1790	HE1	PHE	136	-4.540	68.940	29.465	1.00	0.00	H
ATOM	1791	HE2	PHE	136	-1.056	71.461	29.762	1.00	0.00	H
ATOM	1792	HZ	PHE	136	-2.069	69.188	29.636	1.00	0.00	H
ATOM	1793	N	VAL	137	-6.973	76.212	31.158	1.00	0.00	N
ATOM	1794	CA	VAL	137	-7.554	77.558	31.061	1.00	0.00	C
ATOM	1795	C	VAL	137	-6.600	78.570	31.739	1.00	0.00	C
ATOM	1796	O	VAL	137	-6.337	79.643	31.205	1.00	0.00	O
ATOM	1797	CB	VAL	137	-8.955	77.579	31.739	1.00	0.00	C
ATOM	1798	CG1	VAL	137	-9.681	78.926	31.553	1.00	0.00	C
ATOM	1799	CG2	VAL	137	-9.871	76.446	31.237	1.00	0.00	C
ATOM	1800	H	VAL	137	-7.380	75.571	31.821	1.00	0.00	H
ATOM	1801	HA	VAL	137	-7.657	77.827	30.008	1.00	0.00	H
ATOM	1802	HB	VAL	137	-8.827	77.429	32.812	1.00	0.00	H
ATOM	1803	HG11	VAL	137	-10.663	78.911	32.026	1.00	0.00	H
ATOM	1804	HG12	VAL	137	-9.127	79.756	31.992	1.00	0.00	H
ATOM	1805	HG13	VAL	137	-9.830	79.151	30.496	1.00	0.00	H

ATOM	1806	HG21	VAL	137	-10.864	76.514	31.684	1.00	0.00	H
ATOM	1807	HG22	VAL	137	-10.006	76.481	30.156	1.00	0.00	H
ATOM	1808	HG23	VAL	137	-9.490	75.462	31.498	1.00	0.00	H
ATOM	1809	N	SER	138	-6.035	78.196	32.894	1.00	0.00	N
ATOM	1810	CA	SER	138	-5.039	78.959	33.640	1.00	0.00	C
ATOM	1811	C	SER	138	-3.598	78.691	33.153	1.00	0.00	C
ATOM	1812	O	SER	138	-2.685	79.417	33.538	1.00	0.00	O
ATOM	1813	CB	SER	138	-5.191	78.557	35.127	1.00	0.00	C
ATOM	1814	OG	SER	138	-5.464	77.170	35.304	1.00	0.00	O
ATOM	1815	H	SER	138	-6.306	77.321	33.321	1.00	0.00	H
ATOM	1816	HA	SER	138	-5.223	80.030	33.537	1.00	0.00	H
ATOM	1817	HB2	SER	138	-4.304	78.834	35.701	1.00	0.00	H
ATOM	1818	HB3	SER	138	-6.018	79.115	35.567	1.00	0.00	H
ATOM	1819	HG	SER	138	-4.785	76.644	34.890	1.00	0.00	H
ATOM	1820	N	GLY	139	-3.384	77.677	32.307	1.00	0.00	N
ATOM	1821	CA	GLY	139	-2.116	77.387	31.642	1.00	0.00	C
ATOM	1822	C	GLY	139	-2.318	77.204	30.134	1.00	0.00	C
ATOM	1823	O	GLY	139	-2.278	76.057	29.681	1.00	0.00	O
ATOM	1824	H	GLY	139	-4.191	77.145	32.014	1.00	0.00	H
ATOM	1825	HA2	GLY	139	-1.364	78.158	31.817	1.00	0.00	H
ATOM	1826	HA3	GLY	139	-1.712	76.465	32.063	1.00	0.00	H
ATOM	1827	N	PRO	140	-2.558	78.278	29.348	1.00	0.00	N
ATOM	1828	CA	PRO	140	-2.908	78.180	27.922	1.00	0.00	C
ATOM	1829	C	PRO	140	-1.695	77.853	27.026	1.00	0.00	C
ATOM	1830	O	PRO	140	-1.162	78.708	26.317	1.00	0.00	O
ATOM	1831	CB	PRO	140	-3.545	79.543	27.597	1.00	0.00	C
ATOM	1832	CG	PRO	140	-2.867	80.515	28.550	1.00	0.00	C
ATOM	1833	CD	PRO	140	-2.601	79.673	29.800	1.00	0.00	C
ATOM	1834	HA	PRO	140	-3.656	77.401	27.769	1.00	0.00	H
ATOM	1835	HB2	PRO	140	-3.457	79.840	26.551	1.00	0.00	H
ATOM	1836	HB3	PRO	140	-4.612	79.500	27.825	1.00	0.00	H
ATOM	1837	HG2	PRO	140	-1.923	80.848	28.118	1.00	0.00	H
ATOM	1838	HG3	PRO	140	-3.472	81.399	28.756	1.00	0.00	H
ATOM	1839	HD3	PRO	140	-3.429	79.799	30.499	1.00	0.00	H
ATOM	1840	HD2	PRO	140	-1.681	79.978	30.301	1.00	0.00	H
ATOM	1841	N	ASN	141	-1.262	76.589	27.037	1.00	0.00	N
ATOM	1842	CA	ASN	141	-0.362	75.998	26.051	1.00	0.00	C
ATOM	1843	C	ASN	141	-0.565	74.477	26.016	1.00	0.00	C
ATOM	1844	O	ASN	141	-1.426	73.938	26.717	1.00	0.00	O
ATOM	1845	CB	ASN	141	1.112	76.445	26.238	1.00	0.00	C
ATOM	1846	CG	ASN	141	1.797	76.684	24.888	1.00	0.00	C
ATOM	1847	OD1	ASN	141	1.661	75.878	23.970	1.00	0.00	O
ATOM	1848	ND2	ASN	141	2.475	77.812	24.720	1.00	0.00	N
ATOM	1849	H	ASN	141	-1.700	75.947	27.693	1.00	0.00	H
ATOM	1850	HA	ASN	141	-0.724	76.356	25.085	1.00	0.00	H
ATOM	1851	HB2	ASN	141	1.160	77.365	26.821	1.00	0.00	H
ATOM	1852	HB3	ASN	141	1.679	75.704	26.803	1.00	0.00	H
ATOM	1853	HD22	ASN	141	2.867	78.055	23.814	1.00	0.00	H
ATOM	1854	HD21	ASN	141	2.554	78.474	25.475	1.00	0.00	H
ATOM	1855	N	GLY	142	0.151	73.778	25.128	1.00	0.00	N
ATOM	1856	CA	GLY	142	0.154	72.319	25.001	1.00	0.00	C
ATOM	1857	C	GLY	142	-0.948	71.910	24.028	1.00	0.00	C
ATOM	1858	O	GLY	142	-0.696	71.428	22.927	1.00	0.00	O
ATOM	1859	H	GLY	142	0.806	74.303	24.552	1.00	0.00	H
ATOM	1860	HA2	GLY	142	1.120	71.993	24.615	1.00	0.00	H
ATOM	1861	HA3	GLY	142	0.011	71.829	25.966	1.00	0.00	H
ATOM	1862	N	THR	143	-2.176	72.243	24.434	1.00	0.00	N
ATOM	1863	CA	THR	143	-3.449	72.269	23.714	1.00	0.00	C
ATOM	1864	C	THR	143	-3.377	72.846	22.279	1.00	0.00	C
ATOM	1865	O	THR	143	-4.060	72.368	21.374	1.00	0.00	O
ATOM	1866	CB	THR	143	-4.467	72.996	24.635	1.00	0.00	C
ATOM	1867	OG1	THR	143	-3.924	74.213	25.148	1.00	0.00	O
ATOM	1868	CG2	THR	143	-4.923	72.146	25.830	1.00	0.00	C

ATOM	1869	H	THR	143	-2.200	72.668	25.353	1.00	0.00	H
ATOM	1870	HA	THR	143	-3.772	71.233	23.593	1.00	0.00	H
ATOM	1871	HB	THR	143	-5.351	73.243	24.044	1.00	0.00	H
ATOM	1872	HG1	THR	143	-3.406	74.023	25.926	1.00	0.00	H
ATOM	1873	HG21	THR	143	-5.657	72.678	26.437	1.00	0.00	H
ATOM	1874	HG22	THR	143	-5.397	71.226	25.488	1.00	0.00	H
ATOM	1875	HG23	THR	143	-4.094	71.868	26.483	1.00	0.00	H
ATOM	1876	N	ALA	144	-2.484	73.818	22.050	1.00	0.00	N
ATOM	1877	CA	ALA	144	-2.195	74.440	20.759	1.00	0.00	C
ATOM	1878	C	ALA	144	-1.479	73.509	19.756	1.00	0.00	C
ATOM	1879	O	ALA	144	-1.442	73.810	18.560	1.00	0.00	O
ATOM	1880	CB	ALA	144	-1.289	75.649	21.023	1.00	0.00	C
ATOM	1881	H	ALA	144	-1.987	74.163	22.855	1.00	0.00	H
ATOM	1882	HA	ALA	144	-3.132	74.771	20.308	1.00	0.00	H
ATOM	1883	HB1	ALA	144	-0.913	76.073	20.094	1.00	0.00	H
ATOM	1884	HB2	ALA	144	-1.830	76.439	21.540	1.00	0.00	H
ATOM	1885	HB3	ALA	144	-0.425	75.378	21.628	1.00	0.00	H
ATOM	1886	N	GLY	145	-0.969	72.357	20.213	1.00	0.00	N
ATOM	1887	CA	GLY	145	-0.098	71.457	19.455	1.00	0.00	C
ATOM	1888	C	GLY	145	-0.865	70.505	18.528	1.00	0.00	C
ATOM	1889	O	GLY	145	-0.269	69.682	17.840	1.00	0.00	O
ATOM	1890	H	GLY	145	-1.074	72.144	21.202	1.00	0.00	H
ATOM	1891	HA2	GLY	145	0.630	72.029	18.885	1.00	0.00	H
ATOM	1892	HA3	GLY	145	0.473	70.855	20.162	1.00	0.00	H
ATOM	1893	N	ILE	146	-2.193	70.658	18.473	1.00	0.00	N
ATOM	1894	CA	ILE	146	-3.081	70.103	17.456	1.00	0.00	C
ATOM	1895	C	ILE	146	-2.824	70.820	16.110	1.00	0.00	C
ATOM	1896	O	ILE	146	-2.798	70.206	15.036	1.00	0.00	O
ATOM	1897	CB	ILE	146	-4.550	70.328	17.940	1.00	0.00	C
ATOM	1898	CG1	ILE	146	-4.849	69.585	19.266	1.00	0.00	C
ATOM	1899	CG2	ILE	146	-5.604	69.963	16.876	1.00	0.00	C
ATOM	1900	CD1	ILE	146	-6.145	70.038	19.961	1.00	0.00	C
ATOM	1901	H	ILE	146	-2.588	71.317	19.130	1.00	0.00	H
ATOM	1902	HA	ILE	146	-2.885	69.043	17.333	1.00	0.00	H
ATOM	1903	HB	ILE	146	-4.665	71.393	18.143	1.00	0.00	H
ATOM	1904	HG12	ILE	146	-4.876	68.509	19.091	1.00	0.00	H
ATOM	1905	HG13	ILE	146	-4.042	69.745	19.980	1.00	0.00	H
ATOM	1906	HG21	ILE	146	-6.615	70.157	17.230	1.00	0.00	H
ATOM	1907	HG22	ILE	146	-5.498	70.544	15.961	1.00	0.00	H
ATOM	1908	HG23	ILE	146	-5.534	68.911	16.613	1.00	0.00	H
ATOM	1909	HD11	ILE	146	-6.246	69.556	20.933	1.00	0.00	H
ATOM	1910	HD12	ILE	146	-6.146	71.114	20.135	1.00	0.00	H
ATOM	1911	HD13	ILE	146	-7.038	69.792	19.389	1.00	0.00	H
ATOM	1912	N	PHE	147	-2.669	72.145	16.188	1.00	0.00	N
ATOM	1913	CA	PHE	147	-2.770	73.059	15.066	1.00	0.00	C
ATOM	1914	C	PHE	147	-1.392	73.601	14.685	1.00	0.00	C
ATOM	1915	O	PHE	147	-1.022	73.502	13.518	1.00	0.00	O
ATOM	1916	CB	PHE	147	-3.705	74.208	15.474	1.00	0.00	C
ATOM	1917	CG	PHE	147	-5.144	73.782	15.719	1.00	0.00	C
ATOM	1918	CD1	PHE	147	-5.652	73.739	17.034	1.00	0.00	C
ATOM	1919	CD2	PHE	147	-5.973	73.411	14.639	1.00	0.00	C
ATOM	1920	CE1	PHE	147	-6.968	73.297	17.271	1.00	0.00	C
ATOM	1921	CE2	PHE	147	-7.293	72.980	14.876	1.00	0.00	C
ATOM	1922	CZ	PHE	147	-7.787	72.914	16.193	1.00	0.00	C
ATOM	1923	H	PHE	147	-2.586	72.547	17.112	1.00	0.00	H
ATOM	1924	HA	PHE	147	-3.192	72.560	14.193	1.00	0.00	H
ATOM	1925	HB2	PHE	147	-3.318	74.671	16.382	1.00	0.00	H
ATOM	1926	HB3	PHE	147	-3.708	74.987	14.711	1.00	0.00	H
ATOM	1927	HD1	PHE	147	-5.034	74.032	17.872	1.00	0.00	H
ATOM	1928	HD2	PHE	147	-5.597	73.451	13.629	1.00	0.00	H
ATOM	1929	HE1	PHE	147	-7.344	73.252	18.284	1.00	0.00	H
ATOM	1930	HE2	PHE	147	-7.932	72.701	14.052	1.00	0.00	H
ATOM	1931	HZ	PHE	147	-8.798	72.578	16.379	1.00	0.00	H

ATOM	1932	N	ALA	148	-0.624	74.140	15.637	1.00	0.00	N
ATOM	1933	CA	ALA	148	0.730	74.647	15.419	1.00	0.00	C
ATOM	1934	C	ALA	148	1.730	73.542	15.784	1.00	0.00	C
ATOM	1935	O	ALA	148	1.491	72.796	16.731	1.00	0.00	O
ATOM	1936	CB	ALA	148	0.947	75.812	16.395	1.00	0.00	C
ATOM	1937	H	ALA	148	-0.917	74.049	16.606	1.00	0.00	H
ATOM	1938	HA	ALA	148	0.880	74.982	14.390	1.00	0.00	H
ATOM	1939	HB1	ALA	148	1.936	76.247	16.268	1.00	0.00	H
ATOM	1940	HB2	ALA	148	0.218	76.603	16.223	1.00	0.00	H
ATOM	1941	HB3	ALA	148	0.856	75.494	17.436	1.00	0.00	H
ATOM	1942	N	THR	149	2.850	73.430	15.062	1.00	0.00	N
ATOM	1943	CA	THR	149	3.895	72.450	15.370	1.00	0.00	C
ATOM	1944	C	THR	149	4.846	73.024	16.447	1.00	0.00	C
ATOM	1945	O	THR	149	4.994	74.245	16.568	1.00	0.00	O
ATOM	1946	CB	THR	149	4.638	72.096	14.057	1.00	0.00	C
ATOM	1947	OG1	THR	149	5.330	73.194	13.491	1.00	0.00	O
ATOM	1948	CG2	THR	149	3.698	71.482	13.005	1.00	0.00	C
ATOM	1949	H	THR	149	3.038	74.087	14.304	1.00	0.00	H
ATOM	1950	HA	THR	149	3.444	71.537	15.765	1.00	0.00	H
ATOM	1951	HB	THR	149	5.395	71.347	14.296	1.00	0.00	H
ATOM	1952	HG1	THR	149	4.706	73.894	13.249	1.00	0.00	H
ATOM	1953	HG21	THR	149	4.255	71.066	12.169	1.00	0.00	H
ATOM	1954	HG22	THR	149	3.118	70.663	13.431	1.00	0.00	H
ATOM	1955	HG23	THR	149	2.996	72.215	12.606	1.00	0.00	H
ATOM	1956	N	TYR	150	5.484	72.163	17.243	1.00	0.00	N
ATOM	1957	CA	TYR	150	6.368	72.521	18.354	1.00	0.00	C
ATOM	1958	C	TYR	150	7.610	71.619	18.344	1.00	0.00	C
ATOM	1959	O	TYR	150	7.518	70.510	17.811	1.00	0.00	O
ATOM	1960	CB	TYR	150	5.605	72.426	19.697	1.00	0.00	C
ATOM	1961	CG	TYR	150	4.579	73.505	20.024	1.00	0.00	C
ATOM	1962	CD1	TYR	150	3.432	73.158	20.770	1.00	0.00	C
ATOM	1963	CD2	TYR	150	4.788	74.857	19.667	1.00	0.00	C
ATOM	1964	CE1	TYR	150	2.532	74.152	21.197	1.00	0.00	C
ATOM	1965	CE2	TYR	150	3.870	75.845	20.068	1.00	0.00	C
ATOM	1966	CZ	TYR	150	2.766	75.499	20.864	1.00	0.00	C
ATOM	1967	OH	TYR	150	1.960	76.478	21.358	1.00	0.00	O
ATOM	1968	H	TYR	150	5.434	71.172	17.050	1.00	0.00	H
ATOM	1969	HA	TYR	150	6.734	73.533	18.201	1.00	0.00	H
ATOM	1970	HB2	TYR	150	5.122	71.449	19.753	1.00	0.00	H
ATOM	1971	HB3	TYR	150	6.317	72.428	20.523	1.00	0.00	H
ATOM	1972	HD1	TYR	150	3.258	72.130	21.053	1.00	0.00	H
ATOM	1973	HD2	TYR	150	5.662	75.160	19.112	1.00	0.00	H
ATOM	1974	HE1	TYR	150	1.679	73.875	21.798	1.00	0.00	H
ATOM	1975	HE2	TYR	150	4.048	76.879	19.820	1.00	0.00	H
ATOM	1976	HH	TYR	150	1.728	76.284	22.267	1.00	0.00	H
ATOM	1977	N	PRO	151	8.744	72.059	18.936	1.00	0.00	N
ATOM	1978	CA	PRO	151	9.940	71.228	19.076	1.00	0.00	C
ATOM	1979	C	PRO	151	9.796	70.269	20.271	1.00	0.00	C
ATOM	1980	O	PRO	151	9.019	70.518	21.198	1.00	0.00	O
ATOM	1981	CB	PRO	151	11.071	72.233	19.344	1.00	0.00	C
ATOM	1982	CG	PRO	151	10.393	73.378	20.081	1.00	0.00	C
ATOM	1983	CD	PRO	151	8.970	73.390	19.516	1.00	0.00	C
ATOM	1984	HA	PRO	151	10.139	70.672	18.161	1.00	0.00	H
ATOM	1985	HB2	PRO	151	11.906	71.812	19.907	1.00	0.00	H
ATOM	1986	HB3	PRO	151	11.475	72.588	18.396	1.00	0.00	H
ATOM	1987	HG2	PRO	151	10.367	73.156	21.149	1.00	0.00	H
ATOM	1988	HG3	PRO	151	10.912	74.328	19.950	1.00	0.00	H
ATOM	1989	HD3	PRO	151	8.883	74.152	18.747	1.00	0.00	H
ATOM	1990	HD2	PRO	151	8.252	73.626	20.301	1.00	0.00	H
ATOM	1991	N	SER	152	10.596	69.202	20.276	1.00	0.00	N
ATOM	1992	CA	SER	152	10.833	68.355	21.436	1.00	0.00	C
ATOM	1993	C	SER	152	11.763	69.097	22.419	1.00	0.00	C
ATOM	1994	O	SER	152	12.534	69.981	22.038	1.00	0.00	O

ATOM	1995	CB	SER	152	11.480	67.039	20.961	1.00	0.00	C
ATOM	1996	OG	SER	152	11.494	66.066	21.993	1.00	0.00	O
ATOM	1997	H	SER	152	11.128	69.015	19.432	1.00	0.00	H
ATOM	1998	HA	SER	152	9.877	68.142	21.918	1.00	0.00	H
ATOM	1999	HB2	SER	152	10.909	66.635	20.129	1.00	0.00	H
ATOM	2000	HB3	SER	152	12.494	67.201	20.591	1.00	0.00	H
ATOM	2001	HG	SER	152	11.476	65.209	21.582	1.00	0.00	H
ATOM	2002	N	GLY	153	11.746	68.708	23.697	1.00	0.00	N
ATOM	2003	CA	GLY	153	12.309	69.489	24.800	1.00	0.00	C
ATOM	2004	C	GLY	153	13.767	69.134	25.097	1.00	0.00	C
ATOM	2005	O	GLY	153	14.205	69.219	26.242	1.00	0.00	O
ATOM	2006	H	GLY	153	11.231	67.867	23.911	1.00	0.00	H
ATOM	2007	HA2	GLY	153	12.244	70.559	24.595	1.00	0.00	H
ATOM	2008	HA3	GLY	153	11.715	69.307	25.695	1.00	0.00	H
ATOM	2009	N	HIE	154	14.500	68.743	24.050	1.00	0.00	N
ATOM	2010	CA	HIE	154	15.940	68.505	24.003	1.00	0.00	C
ATOM	2011	C	HIE	154	16.592	69.261	22.832	1.00	0.00	C
ATOM	2012	O	HIE	154	17.808	69.186	22.676	1.00	0.00	O
ATOM	2013	CB	HIE	154	16.236	66.985	23.957	1.00	0.00	C
ATOM	2014	CG	HIE	154	16.161	66.238	25.262	1.00	0.00	C
ATOM	2015	ND1	HIE	154	16.150	64.838	25.316	1.00	0.00	N
ATOM	2016	CD2	HIE	154	16.129	66.716	26.557	1.00	0.00	C
ATOM	2017	CE1	HIE	154	16.099	64.534	26.604	1.00	0.00	C
ATOM	2018	NE2	HIE	154	16.081	65.610	27.389	1.00	0.00	N
ATOM	2019	H	HIE	154	14.012	68.730	23.164	1.00	0.00	H
ATOM	2020	HA	HIE	154	16.411	68.933	24.890	1.00	0.00	H
ATOM	2021	HB2	HIE	154	15.570	66.501	23.241	1.00	0.00	H
ATOM	2022	HB3	HIE	154	17.245	66.813	23.579	1.00	0.00	H
ATOM	2023	HD2	HIE	154	16.129	67.731	26.929	1.00	0.00	H
ATOM	2024	HE1	HIE	154	16.077	63.518	26.973	1.00	0.00	H
ATOM	2025	HE2	HIE	154	16.043	65.618	28.397	1.00	0.00	H
ATOM	2026	N	LEU	155	15.805	69.975	22.014	1.00	0.00	N
ATOM	2027	CA	LEU	155	16.274	70.767	20.879	1.00	0.00	C
ATOM	2028	C	LEU	155	16.319	72.250	21.276	1.00	0.00	C
ATOM	2029	O	LEU	155	15.671	72.665	22.241	1.00	0.00	O
ATOM	2030	CB	LEU	155	15.253	70.650	19.721	1.00	0.00	C
ATOM	2031	CG	LEU	155	14.894	69.218	19.273	1.00	0.00	C
ATOM	2032	CD1	LEU	155	13.955	69.274	18.063	1.00	0.00	C
ATOM	2033	CD2	LEU	155	16.124	68.360	18.943	1.00	0.00	C
ATOM	2034	H	LEU	155	14.839	70.122	22.268	1.00	0.00	H
ATOM	2035	HA	LEU	155	17.264	70.446	20.549	1.00	0.00	H
ATOM	2036	HB2	LEU	155	14.328	71.160	20.001	1.00	0.00	H
ATOM	2037	HB3	LEU	155	15.633	71.202	18.860	1.00	0.00	H
ATOM	2038	HG	LEU	155	14.356	68.731	20.086	1.00	0.00	H
ATOM	2039	HD11	LEU	155	13.635	68.275	17.772	1.00	0.00	H
ATOM	2040	HD12	LEU	155	13.064	69.863	18.276	1.00	0.00	H
ATOM	2041	HD13	LEU	155	14.443	69.717	17.193	1.00	0.00	H
ATOM	2042	HD21	LEU	155	15.831	67.368	18.598	1.00	0.00	H
ATOM	2043	HD22	LEU	155	16.713	68.818	18.151	1.00	0.00	H
ATOM	2044	HD23	LEU	155	16.773	68.222	19.807	1.00	0.00	H
ATOM	2045	N	ASP	156	17.037	73.051	20.484	1.00	0.00	N
ATOM	2046	CA	ASP	156	16.998	74.511	20.493	1.00	0.00	C
ATOM	2047	C	ASP	156	16.191	74.948	19.237	1.00	0.00	C
ATOM	2048	O	ASP	156	15.351	74.200	18.728	1.00	0.00	O
ATOM	2049	CB	ASP	156	18.466	75.042	20.554	1.00	0.00	C
ATOM	2050	CG	ASP	156	19.160	75.236	19.212	1.00	0.00	C
ATOM	2051	OD1	ASP	156	19.355	74.228	18.507	1.00	0.00	O
ATOM	2052	OD2	ASP	156	19.185	76.411	18.786	1.00	0.00	O1-
ATOM	2053	H	ASP	156	17.534	72.645	19.703	1.00	0.00	H
ATOM	2054	HA	ASP	156	16.463	74.870	21.374	1.00	0.00	H
ATOM	2055	HB2	ASP	156	18.468	76.005	21.063	1.00	0.00	H
ATOM	2056	HB3	ASP	156	19.087	74.389	21.168	1.00	0.00	H
ATOM	2057	N	MET	157	16.418	76.168	18.737	1.00	0.00	N

ATOM	2058	CA	MET	157	15.851	76.675	17.495	1.00	0.00	C
ATOM	2059	C	MET	157	16.616	76.186	16.271	1.00	0.00	C
ATOM	2060	O	MET	157	15.999	75.901	15.248	1.00	0.00	O
ATOM	2061	CB	MET	157	15.922	78.216	17.512	1.00	0.00	C
ATOM	2062	CG	MET	157	14.966	78.848	18.536	1.00	0.00	C
ATOM	2063	SD	MET	157	14.974	80.663	18.571	1.00	0.00	S
ATOM	2064	CE	MET	157	14.124	81.025	17.007	1.00	0.00	C
ATOM	2065	H	MET	157	17.308	76.577	19.028	1.00	0.00	H
ATOM	2066	HA	MET	157	14.812	76.354	17.396	1.00	0.00	H
ATOM	2067	HB2	MET	157	16.945	78.544	17.708	1.00	0.00	H
ATOM	2068	HB3	MET	157	15.671	78.592	16.519	1.00	0.00	H
ATOM	2069	HG2	MET	157	13.946	78.505	18.360	1.00	0.00	H
ATOM	2070	HG3	MET	157	15.236	78.503	19.535	1.00	0.00	H
ATOM	2071	HE1	MET	157	14.002	82.101	16.887	1.00	0.00	H
ATOM	2072	HE2	MET	157	14.694	80.652	16.156	1.00	0.00	H
ATOM	2073	HE3	MET	157	13.136	80.565	16.995	1.00	0.00	H
ATOM	2074	N	ILE	158	17.942	76.101	16.365	1.00	0.00	N
ATOM	2075	CA	ILE	158	18.826	75.757	15.270	1.00	0.00	C
ATOM	2076	C	ILE	158	18.753	74.265	14.980	1.00	0.00	C
ATOM	2077	O	ILE	158	18.622	73.875	13.819	1.00	0.00	O
ATOM	2078	CB	ILE	158	20.280	76.210	15.619	1.00	0.00	C
ATOM	2079	CG1	ILE	158	20.355	77.737	15.888	1.00	0.00	C
ATOM	2080	CG2	ILE	158	21.304	75.815	14.533	1.00	0.00	C
ATOM	2081	CD1	ILE	158	21.671	78.201	16.530	1.00	0.00	C
ATOM	2082	H	ILE	158	18.401	76.242	17.277	1.00	0.00	H
ATOM	2083	HA	ILE	158	18.503	76.291	14.374	1.00	0.00	H
ATOM	2084	HB	ILE	158	20.572	75.698	16.538	1.00	0.00	H
ATOM	2085	HG12	ILE	158	20.175	78.287	14.964	1.00	0.00	H
ATOM	2086	HG13	ILE	158	19.559	78.035	16.571	1.00	0.00	H
ATOM	2087	HG21	ILE	158	22.315	76.124	14.791	1.00	0.00	H
ATOM	2088	HG22	ILE	158	21.348	74.735	14.403	1.00	0.00	H
ATOM	2089	HG23	ILE	158	21.053	76.254	13.567	1.00	0.00	H
ATOM	2090	HD11	ILE	158	21.616	79.257	16.795	1.00	0.00	H
ATOM	2091	HD12	ILE	158	21.867	77.643	17.447	1.00	0.00	H
ATOM	2092	HD13	ILE	158	22.523	78.074	15.865	1.00	0.00	H
ATOM	2093	N	ASN	159	18.731	73.432	16.024	1.00	0.00	N
ATOM	2094	CA	ASN	159	18.558	71.999	15.862	1.00	0.00	C
ATOM	2095	C	ASN	159	17.194	71.641	15.278	1.00	0.00	C
ATOM	2096	O	ASN	159	17.077	70.669	14.533	1.00	0.00	O
ATOM	2097	CB	ASN	159	18.772	71.241	17.195	1.00	0.00	C
ATOM	2098	CG	ASN	159	19.257	69.791	17.014	1.00	0.00	C
ATOM	2099	OD1	ASN	159	20.245	69.390	17.618	1.00	0.00	O
ATOM	2100	ND2	ASN	159	18.578	68.962	16.226	1.00	0.00	N
ATOM	2101	H	ASN	159	18.936	73.803	16.969	1.00	0.00	H
ATOM	2102	HA	ASN	159	19.323	71.671	15.154	1.00	0.00	H
ATOM	2103	HB2	ASN	159	19.539	71.751	17.780	1.00	0.00	H
ATOM	2104	HB3	ASN	159	17.873	71.251	17.811	1.00	0.00	H
ATOM	2105	HD22	ASN	159	18.881	68.006	16.146	1.00	0.00	H
ATOM	2106	HD21	ASN	159	17.785	69.283	15.669	1.00	0.00	H
ATOM	2107	N	GLY	160	16.196	72.486	15.529	1.00	0.00	N
ATOM	2108	CA	GLY	160	14.870	72.281	14.996	1.00	0.00	C
ATOM	2109	C	GLY	160	14.713	72.807	13.576	1.00	0.00	C
ATOM	2110	O	GLY	160	13.862	72.341	12.817	1.00	0.00	O
ATOM	2111	H	GLY	160	16.372	73.276	16.134	1.00	0.00	H
ATOM	2112	HA2	GLY	160	14.594	71.224	15.023	1.00	0.00	H
ATOM	2113	HA3	GLY	160	14.158	72.796	15.637	1.00	0.00	H
ATOM	2114	N	PHE	161	15.588	73.737	13.198	1.00	0.00	N
ATOM	2115	CA	PHE	161	15.728	74.243	11.850	1.00	0.00	C
ATOM	2116	C	PHE	161	16.347	73.190	10.947	1.00	0.00	C
ATOM	2117	O	PHE	161	15.872	72.963	9.835	1.00	0.00	O
ATOM	2118	CB	PHE	161	16.646	75.501	11.891	1.00	0.00	C
ATOM	2119	CG	PHE	161	16.572	76.469	10.719	1.00	0.00	C
ATOM	2120	CD1	PHE	161	16.019	77.755	10.907	1.00	0.00	C

ATOM	2121	CD2	PHE	161	17.066	76.105	9.447	1.00	0.00	C
ATOM	2122	CE1	PHE	161	15.923	78.653	9.828	1.00	0.00	C
ATOM	2123	CE2	PHE	161	16.974	77.005	8.369	1.00	0.00	C
ATOM	2124	CZ	PHE	161	16.398	78.277	8.558	1.00	0.00	C
ATOM	2125	H	PHE	161	16.246	74.065	13.893	1.00	0.00	H
ATOM	2126	HA	PHE	161	14.752	74.516	11.463	1.00	0.00	H
ATOM	2127	HB2	PHE	161	16.422	76.075	12.788	1.00	0.00	H
ATOM	2128	HB3	PHE	161	17.693	75.219	11.989	1.00	0.00	H
ATOM	2129	HD1	PHE	161	15.654	78.056	11.878	1.00	0.00	H
ATOM	2130	HD2	PHE	161	17.516	75.136	9.287	1.00	0.00	H
ATOM	2131	HE1	PHE	161	15.485	79.629	9.975	1.00	0.00	H
ATOM	2132	HE2	PHE	161	17.354	76.712	7.401	1.00	0.00	H
ATOM	2133	HZ	PHE	161	16.328	78.975	7.738	1.00	0.00	H
ATOM	2134	N	PHE	162	17.392	72.530	11.448	1.00	0.00	N
ATOM	2135	CA	PHE	162	18.102	71.490	10.734	1.00	0.00	C
ATOM	2136	C	PHE	162	17.231	70.281	10.422	1.00	0.00	C
ATOM	2137	O	PHE	162	17.337	69.734	9.326	1.00	0.00	O
ATOM	2138	CB	PHE	162	19.323	71.020	11.558	1.00	0.00	C
ATOM	2139	CG	PHE	162	20.482	71.991	11.759	1.00	0.00	C
ATOM	2140	CD1	PHE	162	20.701	73.097	10.906	1.00	0.00	C
ATOM	2141	CD2	PHE	162	21.387	71.746	12.814	1.00	0.00	C
ATOM	2142	CE1	PHE	162	21.812	73.940	11.107	1.00	0.00	C
ATOM	2143	CE2	PHE	162	22.500	72.585	13.011	1.00	0.00	C
ATOM	2144	CZ	PHE	162	22.715	73.681	12.154	1.00	0.00	C
ATOM	2145	H	PHE	162	17.748	72.805	12.360	1.00	0.00	H
ATOM	2146	HA	PHE	162	18.437	71.883	9.774	1.00	0.00	H
ATOM	2147	HB2	PHE	162	18.984	70.683	12.539	1.00	0.00	H
ATOM	2148	HB3	PHE	162	19.753	70.136	11.082	1.00	0.00	H
ATOM	2149	HD1	PHE	162	20.032	73.319	10.090	1.00	0.00	H
ATOM	2150	HD2	PHE	162	21.232	70.907	13.478	1.00	0.00	H
ATOM	2151	HE1	PHE	162	21.970	74.785	10.454	1.00	0.00	H
ATOM	2152	HE2	PHE	162	23.182	72.389	13.825	1.00	0.00	H
ATOM	2153	HZ	PHE	162	23.564	74.329	12.311	1.00	0.00	H
ATOM	2154	N	ASP	163	16.309	69.938	11.328	1.00	0.00	N
ATOM	2155	CA	ASP	163	15.346	68.876	11.097	1.00	0.00	C
ATOM	2156	C	ASP	163	14.459	69.157	9.881	1.00	0.00	C
ATOM	2157	O	ASP	163	14.390	68.381	8.925	1.00	0.00	O
ATOM	2158	CB	ASP	163	14.287	68.746	12.239	1.00	0.00	C
ATOM	2159	CG	ASP	163	14.631	68.272	13.632	1.00	0.00	C
ATOM	2160	OD1	ASP	163	15.420	67.316	13.779	1.00	0.00	O
ATOM	2161	OD2	ASP	163	13.871	68.731	14.514	1.00	0.00	O1-
ATOM	2162	H	ASP	163	16.311	70.392	12.233	1.00	0.00	H
ATOM	2163	HA	ASP	163	15.866	67.925	10.967	1.00	0.00	H
ATOM	2164	HB2	ASP	163	13.792	69.705	12.392	1.00	0.00	H
ATOM	2165	HB3	ASP	163	13.503	68.054	11.924	1.00	0.00	H
ATOM	2166	N	GLN	164	13.746	70.290	9.962	1.00	0.00	N
ATOM	2167	CA	GLN	164	12.702	70.671	9.027	1.00	0.00	C
ATOM	2168	C	GLN	164	13.272	71.067	7.674	1.00	0.00	C
ATOM	2169	O	GLN	164	12.588	70.983	6.659	1.00	0.00	O
ATOM	2170	CB	GLN	164	11.860	71.824	9.621	1.00	0.00	C
ATOM	2171	CG	GLN	164	10.971	71.405	10.813	1.00	0.00	C
ATOM	2172	CD	GLN	164	9.993	70.286	10.450	1.00	0.00	C
ATOM	2173	OE1	GLN	164	9.267	70.366	9.461	1.00	0.00	O
ATOM	2174	NE2	GLN	164	9.990	69.204	11.212	1.00	0.00	N
ATOM	2175	H	GLN	164	13.879	70.854	10.793	1.00	0.00	H
ATOM	2176	HA	GLN	164	12.075	69.799	8.835	1.00	0.00	H
ATOM	2177	HB2	GLN	164	12.517	72.633	9.939	1.00	0.00	H
ATOM	2178	HB3	GLN	164	11.215	72.237	8.844	1.00	0.00	H
ATOM	2179	HG2	GLN	164	11.599	71.101	11.652	1.00	0.00	H
ATOM	2180	HG3	GLN	164	10.395	72.263	11.160	1.00	0.00	H
ATOM	2181	HE22	GLN	164	9.487	68.391	10.917	1.00	0.00	H
ATOM	2182	HE21	GLN	164	10.596	69.158	12.047	1.00	0.00	H
ATOM	2183	N	PHE	165	14.551	71.417	7.661	1.00	0.00	N

ATOM	2184	CA	PHE	165	15.365	71.508	6.481	1.00	0.00	C
ATOM	2185	C	PHE	165	15.587	70.158	5.821	1.00	0.00	C
ATOM	2186	O	PHE	165	15.143	69.938	4.691	1.00	0.00	O
ATOM	2187	CB	PHE	165	16.687	72.209	6.891	1.00	0.00	C
ATOM	2188	CG	PHE	165	17.776	72.373	5.844	1.00	0.00	C
ATOM	2189	CD1	PHE	165	17.589	73.238	4.749	1.00	0.00	C
ATOM	2190	CD2	PHE	165	19.003	71.691	5.989	1.00	0.00	C
ATOM	2191	CE1	PHE	165	18.611	73.397	3.792	1.00	0.00	C
ATOM	2192	CE2	PHE	165	20.020	71.843	5.029	1.00	0.00	C
ATOM	2193	CZ	PHE	165	19.822	72.693	3.925	1.00	0.00	C
ATOM	2194	H	PHE	165	15.018	71.485	8.559	1.00	0.00	H
ATOM	2195	HA	PHE	165	14.858	72.141	5.752	1.00	0.00	H
ATOM	2196	HB2	PHE	165	16.450	73.212	7.248	1.00	0.00	H
ATOM	2197	HB3	PHE	165	17.123	71.699	7.746	1.00	0.00	H
ATOM	2198	HD1	PHE	165	16.661	73.781	4.645	1.00	0.00	H
ATOM	2199	HD2	PHE	165	19.177	71.057	6.844	1.00	0.00	H
ATOM	2200	HE1	PHE	165	18.472	74.056	2.949	1.00	0.00	H
ATOM	2201	HE2	PHE	165	20.954	71.311	5.143	1.00	0.00	H
ATOM	2202	HZ	PHE	165	20.601	72.812	3.186	1.00	0.00	H
ATOM	2203	N	ILE	166	16.233	69.242	6.542	1.00	0.00	N
ATOM	2204	CA	ILE	166	16.646	67.960	6.008	1.00	0.00	C
ATOM	2205	C	ILE	166	15.480	67.088	5.527	1.00	0.00	C
ATOM	2206	O	ILE	166	15.564	66.416	4.492	1.00	0.00	O
ATOM	2207	CB	ILE	166	17.544	67.218	7.054	1.00	0.00	C
ATOM	2208	CG1	ILE	166	18.900	67.944	7.231	1.00	0.00	C
ATOM	2209	CG2	ILE	166	17.804	65.725	6.732	1.00	0.00	C
ATOM	2210	CD1	ILE	166	19.695	67.523	8.477	1.00	0.00	C
ATOM	2211	H	ILE	166	16.510	69.455	7.496	1.00	0.00	H
ATOM	2212	HA	ILE	166	17.256	68.156	5.123	1.00	0.00	H
ATOM	2213	HB	ILE	166	17.019	67.246	8.012	1.00	0.00	H
ATOM	2214	HG12	ILE	166	19.514	67.812	6.339	1.00	0.00	H
ATOM	2215	HG13	ILE	166	18.728	69.012	7.310	1.00	0.00	H
ATOM	2216	HG21	ILE	166	18.454	65.258	7.470	1.00	0.00	H
ATOM	2217	HG22	ILE	166	16.888	65.135	6.737	1.00	0.00	H
ATOM	2218	HG23	ILE	166	18.274	65.605	5.755	1.00	0.00	H
ATOM	2219	HD11	ILE	166	20.565	68.165	8.612	1.00	0.00	H
ATOM	2220	HD12	ILE	166	19.087	67.602	9.379	1.00	0.00	H
ATOM	2221	HD13	ILE	166	20.059	66.498	8.407	1.00	0.00	H
ATOM	2222	N	GLY	167	14.359	67.152	6.238	1.00	0.00	N
ATOM	2223	CA	GLY	167	13.249	66.278	5.945	1.00	0.00	C
ATOM	2224	C	GLY	167	12.350	66.828	4.847	1.00	0.00	C
ATOM	2225	O	GLY	167	11.742	66.046	4.116	1.00	0.00	O
ATOM	2226	H	GLY	167	14.367	67.678	7.111	1.00	0.00	H
ATOM	2227	HA2	GLY	167	13.586	65.272	5.684	1.00	0.00	H
ATOM	2228	HA3	GLY	167	12.651	66.172	6.850	1.00	0.00	H
ATOM	2229	N	THR	168	12.338	68.151	4.649	1.00	0.00	N
ATOM	2230	CA	THR	168	11.646	68.739	3.510	1.00	0.00	C
ATOM	2231	C	THR	168	12.486	68.641	2.250	1.00	0.00	C
ATOM	2232	O	THR	168	11.934	68.525	1.162	1.00	0.00	O
ATOM	2233	CB	THR	168	11.290	70.224	3.775	1.00	0.00	C
ATOM	2234	OG1	THR	168	10.625	70.341	5.016	1.00	0.00	O
ATOM	2235	CG2	THR	168	10.300	70.793	2.745	1.00	0.00	C
ATOM	2236	H	THR	168	12.896	68.751	5.240	1.00	0.00	H
ATOM	2237	HA	THR	168	10.708	68.205	3.360	1.00	0.00	H
ATOM	2238	HB	THR	168	12.189	70.843	3.801	1.00	0.00	H
ATOM	2239	HG1	THR	168	11.299	70.473	5.687	1.00	0.00	H
ATOM	2240	HG21	THR	168	10.002	71.810	2.988	1.00	0.00	H
ATOM	2241	HG22	THR	168	10.739	70.818	1.751	1.00	0.00	H
ATOM	2242	HG23	THR	168	9.391	70.192	2.690	1.00	0.00	H
ATOM	2243	N	ALA	169	13.812	68.584	2.376	1.00	0.00	N
ATOM	2244	CA	ALA	169	14.645	68.248	1.244	1.00	0.00	C
ATOM	2245	C	ALA	169	14.371	66.841	0.751	1.00	0.00	C
ATOM	2246	O	ALA	169	14.193	66.634	-0.436	1.00	0.00	O

ATOM	2247	CB	ALA	169	16.128	68.391	1.633	1.00	0.00	C
ATOM	2248	H	ALA	169	14.247	68.728	3.281	1.00	0.00	H
ATOM	2249	HA	ALA	169	14.401	68.909	0.417	1.00	0.00	H
ATOM	2250	HB1	ALA	169	16.773	68.226	0.769	1.00	0.00	H
ATOM	2251	HB2	ALA	169	16.347	69.388	2.014	1.00	0.00	H
ATOM	2252	HB3	ALA	169	16.424	67.676	2.399	1.00	0.00	H
ATOM	2253	N	SER	170	14.197	65.896	1.664	1.00	0.00	N
ATOM	2254	CA	SER	170	13.873	64.538	1.284	1.00	0.00	C
ATOM	2255	C	SER	170	12.456	64.381	0.758	1.00	0.00	C
ATOM	2256	O	SER	170	12.248	63.628	-0.194	1.00	0.00	O
ATOM	2257	CB	SER	170	14.030	63.669	2.552	1.00	0.00	C
ATOM	2258	OG	SER	170	15.344	63.764	3.085	1.00	0.00	O
ATOM	2259	H	SER	170	14.365	66.116	2.636	1.00	0.00	H
ATOM	2260	HA	SER	170	14.578	64.188	0.526	1.00	0.00	H
ATOM	2261	HB2	SER	170	13.311	63.948	3.324	1.00	0.00	H
ATOM	2262	HB3	SER	170	13.836	62.622	2.310	1.00	0.00	H
ATOM	2263	HG	SER	170	15.487	64.624	3.469	1.00	0.00	H
ATOM	2264	N	LEU	171	11.512	65.158	1.311	1.00	0.00	N
ATOM	2265	CA	LEU	171	10.176	65.317	0.764	1.00	0.00	C
ATOM	2266	C	LEU	171	10.228	65.709	-0.709	1.00	0.00	C
ATOM	2267	O	LEU	171	9.705	64.995	-1.557	1.00	0.00	O
ATOM	2268	CB	LEU	171	9.315	66.273	1.618	1.00	0.00	C
ATOM	2269	CG	LEU	171	7.855	66.457	1.143	1.00	0.00	C
ATOM	2270	CD1	LEU	171	7.070	65.136	1.151	1.00	0.00	C
ATOM	2271	CD2	LEU	171	7.142	67.514	1.997	1.00	0.00	C
ATOM	2272	H	LEU	171	11.757	65.703	2.130	1.00	0.00	H
ATOM	2273	HA	LEU	171	9.723	64.326	0.813	1.00	0.00	H
ATOM	2274	HB2	LEU	171	9.317	65.928	2.653	1.00	0.00	H
ATOM	2275	HB3	LEU	171	9.786	67.251	1.627	1.00	0.00	H
ATOM	2276	HG	LEU	171	7.858	66.835	0.120	1.00	0.00	H
ATOM	2277	HD11	LEU	171	6.024	65.297	0.891	1.00	0.00	H
ATOM	2278	HD12	LEU	171	7.466	64.426	0.424	1.00	0.00	H
ATOM	2279	HD13	LEU	171	7.107	64.659	2.131	1.00	0.00	H
ATOM	2280	HD21	LEU	171	6.125	67.690	1.645	1.00	0.00	H
ATOM	2281	HD22	LEU	171	7.085	67.196	3.036	1.00	0.00	H
ATOM	2282	HD23	LEU	171	7.668	68.470	1.971	1.00	0.00	H
ATOM	2283	N	ILE	172	10.940	66.793	-1.014	1.00	0.00	N
ATOM	2284	CA	ILE	172	10.884	67.414	-2.325	1.00	0.00	C
ATOM	2285	C	ILE	172	12.041	66.940	-3.230	1.00	0.00	C
ATOM	2286	O	ILE	172	12.143	67.355	-4.379	1.00	0.00	O
ATOM	2287	CB	ILE	172	10.717	68.964	-2.196	1.00	0.00	C
ATOM	2288	CG1	ILE	172	9.933	69.453	-0.929	1.00	0.00	C
ATOM	2289	CG2	ILE	172	10.067	69.568	-3.465	1.00	0.00	C
ATOM	2290	CD1	ILE	172	8.444	69.734	-1.078	1.00	0.00	C
ATOM	2291	H	ILE	172	11.413	67.300	-0.272	1.00	0.00	H
ATOM	2292	HA	ILE	172	9.982	67.076	-2.832	1.00	0.00	H
ATOM	2293	HB	ILE	172	11.728	69.368	-2.116	1.00	0.00	H
ATOM	2294	HG12	ILE	172	9.989	68.775	-0.092	1.00	0.00	H
ATOM	2295	HG13	ILE	172	10.396	70.359	-0.564	1.00	0.00	H
ATOM	2296	HG21	ILE	172	10.105	70.657	-3.455	1.00	0.00	H
ATOM	2297	HG22	ILE	172	10.544	69.229	-4.379	1.00	0.00	H
ATOM	2298	HG23	ILE	172	9.026	69.265	-3.577	1.00	0.00	H
ATOM	2299	HD11	ILE	172	7.997	69.972	-0.113	1.00	0.00	H
ATOM	2300	HD12	ILE	172	8.293	70.594	-1.726	1.00	0.00	H
ATOM	2301	HD13	ILE	172	7.919	68.869	-1.486	1.00	0.00	H
ATOM	2302	N	VAL	173	12.834	65.969	-2.761	1.00	0.00	N
ATOM	2303	CA	VAL	173	13.558	65.042	-3.611	1.00	0.00	C
ATOM	2304	C	VAL	173	12.582	63.973	-4.092	1.00	0.00	C
ATOM	2305	O	VAL	173	12.408	63.769	-5.290	1.00	0.00	O
ATOM	2306	CB	VAL	173	14.716	64.297	-2.837	1.00	0.00	C
ATOM	2307	CG1	VAL	173	15.236	62.984	-3.478	1.00	0.00	C
ATOM	2308	CG2	VAL	173	15.948	65.172	-2.633	1.00	0.00	C
ATOM	2309	H	VAL	173	12.799	65.776	-1.770	1.00	0.00	H

ATOM	2310	HA	VAL	173	13.971	65.557	-4.481	1.00	0.00	H
ATOM	2311	HB	VAL	173	14.342	64.034	-1.848	1.00	0.00	H
ATOM	2312	HG11	VAL	173	16.102	62.598	-2.940	1.00	0.00	H
ATOM	2313	HG12	VAL	173	14.500	62.180	-3.468	1.00	0.00	H
ATOM	2314	HG13	VAL	173	15.540	63.144	-4.513	1.00	0.00	H
ATOM	2315	HG21	VAL	173	16.639	64.738	-1.911	1.00	0.00	H
ATOM	2316	HG22	VAL	173	16.490	65.321	-3.567	1.00	0.00	H
ATOM	2317	HG23	VAL	173	15.649	66.136	-2.263	1.00	0.00	H
ATOM	2318	N	CYS	174	11.981	63.244	-3.147	1.00	0.00	N
ATOM	2319	CA	CYS	174	11.291	62.001	-3.456	1.00	0.00	C
ATOM	2320	C	CYS	174	9.979	62.249	-4.171	1.00	0.00	C
ATOM	2321	O	CYS	174	9.555	61.420	-4.965	1.00	0.00	O
ATOM	2322	CB	CYS	174	10.983	61.234	-2.154	1.00	0.00	C
ATOM	2323	SG	CYS	174	12.519	60.693	-1.349	1.00	0.00	S
ATOM	2324	H	CYS	174	12.140	63.465	-2.169	1.00	0.00	H
ATOM	2325	HA	CYS	174	11.927	61.384	-4.092	1.00	0.00	H
ATOM	2326	HB2	CYS	174	10.404	61.843	-1.457	1.00	0.00	H
ATOM	2327	HB3	CYS	174	10.390	60.344	-2.369	1.00	0.00	H
ATOM	2328	HG	CYS	174	12.839	61.903	-0.870	1.00	0.00	H
ATOM	2329	N	VAL	175	9.388	63.421	-3.947	1.00	0.00	N
ATOM	2330	CA	VAL	175	8.269	63.912	-4.710	1.00	0.00	C
ATOM	2331	C	VAL	175	8.611	64.272	-6.151	1.00	0.00	C
ATOM	2332	O	VAL	175	7.706	64.265	-6.975	1.00	0.00	O
ATOM	2333	CB	VAL	175	7.619	65.111	-3.941	1.00	0.00	C
ATOM	2334	CG1	VAL	175	6.618	65.977	-4.738	1.00	0.00	C
ATOM	2335	CG2	VAL	175	6.907	64.628	-2.661	1.00	0.00	C
ATOM	2336	H	VAL	175	9.762	64.022	-3.219	1.00	0.00	H
ATOM	2337	HA	VAL	175	7.511	63.133	-4.745	1.00	0.00	H
ATOM	2338	HB	VAL	175	8.423	65.783	-3.651	1.00	0.00	H
ATOM	2339	HG11	VAL	175	6.158	66.735	-4.104	1.00	0.00	H
ATOM	2340	HG12	VAL	175	7.100	66.509	-5.559	1.00	0.00	H
ATOM	2341	HG13	VAL	175	5.818	65.369	-5.163	1.00	0.00	H
ATOM	2342	HG21	VAL	175	6.685	65.464	-1.997	1.00	0.00	H
ATOM	2343	HG22	VAL	175	5.963	64.143	-2.902	1.00	0.00	H
ATOM	2344	HG23	VAL	175	7.498	63.909	-2.096	1.00	0.00	H
ATOM	2345	N	LEU	176	9.881	64.531	-6.475	1.00	0.00	N
ATOM	2346	CA	LEU	176	10.289	64.557	-7.867	1.00	0.00	C
ATOM	2347	C	LEU	176	10.528	63.152	-8.365	1.00	0.00	C
ATOM	2348	O	LEU	176	10.026	62.791	-9.421	1.00	0.00	O
ATOM	2349	CB	LEU	176	11.578	65.381	-8.075	1.00	0.00	C
ATOM	2350	CG	LEU	176	11.444	66.896	-7.815	1.00	0.00	C
ATOM	2351	CD1	LEU	176	12.818	67.561	-7.963	1.00	0.00	C
ATOM	2352	CD2	LEU	176	10.423	67.579	-8.742	1.00	0.00	C
ATOM	2353	H	LEU	176	10.608	64.454	-5.777	1.00	0.00	H
ATOM	2354	HA	LEU	176	9.509	64.990	-8.493	1.00	0.00	H
ATOM	2355	HB2	LEU	176	12.387	64.979	-7.467	1.00	0.00	H
ATOM	2356	HB3	LEU	176	11.911	65.244	-9.106	1.00	0.00	H
ATOM	2357	HG	LEU	176	11.112	67.044	-6.787	1.00	0.00	H
ATOM	2358	HD11	LEU	176	12.750	68.640	-7.858	1.00	0.00	H
ATOM	2359	HD12	LEU	176	13.503	67.193	-7.201	1.00	0.00	H
ATOM	2360	HD13	LEU	176	13.256	67.359	-8.940	1.00	0.00	H
ATOM	2361	HD21	LEU	176	10.407	68.657	-8.585	1.00	0.00	H
ATOM	2362	HD22	LEU	176	10.656	67.398	-9.793	1.00	0.00	H
ATOM	2363	HD23	LEU	176	9.409	67.222	-8.560	1.00	0.00	H
ATOM	2364	N	ALA	177	11.232	62.346	-7.572	1.00	0.00	N
ATOM	2365	CA	ALA	177	11.586	60.993	-7.943	1.00	0.00	C
ATOM	2366	C	ALA	177	10.396	60.080	-8.227	1.00	0.00	C
ATOM	2367	O	ALA	177	10.454	59.244	-9.129	1.00	0.00	O
ATOM	2368	CB	ALA	177	12.449	60.369	-6.835	1.00	0.00	C
ATOM	2369	H	ALA	177	11.643	62.747	-6.738	1.00	0.00	H
ATOM	2370	HA	ALA	177	12.176	61.045	-8.860	1.00	0.00	H
ATOM	2371	HB1	ALA	177	12.908	59.445	-7.188	1.00	0.00	H
ATOM	2372	HB2	ALA	177	13.253	61.036	-6.524	1.00	0.00	H

ATOM	2373	HB3	ALA	177	11.856	60.119	-5.956	1.00	0.00	H
ATOM	2374	N	ILE	178	9.313	60.272	-7.471	1.00	0.00	N
ATOM	2375	CA	ILE	178	8.083	59.525	-7.627	1.00	0.00	C
ATOM	2376	C	ILE	178	7.179	60.068	-8.734	1.00	0.00	C
ATOM	2377	O	ILE	178	6.168	59.439	-9.049	1.00	0.00	O
ATOM	2378	CB	ILE	178	7.348	59.413	-6.260	1.00	0.00	C
ATOM	2379	CG1	ILE	178	6.282	58.295	-6.174	1.00	0.00	C
ATOM	2380	CG2	ILE	178	6.703	60.733	-5.824	1.00	0.00	C
ATOM	2381	CD1	ILE	178	6.750	56.892	-6.590	1.00	0.00	C
ATOM	2382	H	ILE	178	9.375	60.923	-6.693	1.00	0.00	H
ATOM	2383	HA	ILE	178	8.367	58.521	-7.940	1.00	0.00	H
ATOM	2384	HB	ILE	178	8.112	59.164	-5.522	1.00	0.00	H
ATOM	2385	HG12	ILE	178	5.939	58.254	-5.142	1.00	0.00	H
ATOM	2386	HG13	ILE	178	5.405	58.558	-6.765	1.00	0.00	H
ATOM	2387	HG21	ILE	178	6.627	60.785	-4.739	1.00	0.00	H
ATOM	2388	HG22	ILE	178	7.288	61.578	-6.173	1.00	0.00	H
ATOM	2389	HG23	ILE	178	5.705	60.850	-6.247	1.00	0.00	H
ATOM	2390	HD11	ILE	178	5.956	56.163	-6.432	1.00	0.00	H
ATOM	2391	HD12	ILE	178	6.996	56.843	-7.650	1.00	0.00	H
ATOM	2392	HD13	ILE	178	7.622	56.573	-6.018	1.00	0.00	H
ATOM	2393	N	VAL	179	7.542	61.204	-9.336	1.00	0.00	N
ATOM	2394	CA	VAL	179	6.748	61.829	-10.369	1.00	0.00	C
ATOM	2395	C	VAL	179	7.509	62.156	-11.672	1.00	0.00	C
ATOM	2396	O	VAL	179	6.929	62.748	-12.584	1.00	0.00	O
ATOM	2397	CB	VAL	179	5.846	62.996	-9.860	1.00	0.00	C
ATOM	2398	CG1	VAL	179	5.118	62.698	-8.538	1.00	0.00	C
ATOM	2399	CG2	VAL	179	6.562	64.359	-9.816	1.00	0.00	C
ATOM	2400	H	VAL	179	8.406	61.662	-9.068	1.00	0.00	H
ATOM	2401	HA	VAL	179	6.038	61.090	-10.745	1.00	0.00	H
ATOM	2402	HB	VAL	179	5.054	63.109	-10.603	1.00	0.00	H
ATOM	2403	HG11	VAL	179	4.363	63.454	-8.323	1.00	0.00	H
ATOM	2404	HG12	VAL	179	4.620	61.730	-8.575	1.00	0.00	H
ATOM	2405	HG13	VAL	179	5.813	62.686	-7.701	1.00	0.00	H
ATOM	2406	HG21	VAL	179	6.026	65.084	-9.204	1.00	0.00	H
ATOM	2407	HG22	VAL	179	7.569	64.255	-9.421	1.00	0.00	H
ATOM	2408	HG23	VAL	179	6.661	64.786	-10.814	1.00	0.00	H
ATOM	2409	N	ASP	180	8.789	61.794	-11.785	1.00	0.00	N
ATOM	2410	CA	ASP	180	9.640	62.144	-12.923	1.00	0.00	C
ATOM	2411	C	ASP	180	9.501	61.069	-14.026	1.00	0.00	C
ATOM	2412	O	ASP	180	9.759	59.899	-13.739	1.00	0.00	O
ATOM	2413	CB	ASP	180	11.106	62.221	-12.421	1.00	0.00	C
ATOM	2414	CG	ASP	180	12.179	62.404	-13.492	1.00	0.00	C
ATOM	2415	OD1	ASP	180	11.835	62.662	-14.664	1.00	0.00	O
ATOM	2416	OD2	ASP	180	13.361	62.281	-13.114	1.00	0.00	O1-
ATOM	2417	H	ASP	180	9.223	61.316	-11.009	1.00	0.00	H
ATOM	2418	HA	ASP	180	9.370	63.134	-13.286	1.00	0.00	H
ATOM	2419	HB2	ASP	180	11.211	63.049	-11.722	1.00	0.00	H
ATOM	2420	HB3	ASP	180	11.353	61.316	-11.865	1.00	0.00	H
ATOM	2421	N	PRO	181	9.152	61.430	-15.284	1.00	0.00	N
ATOM	2422	CA	PRO	181	9.090	60.478	-16.399	1.00	0.00	C
ATOM	2423	C	PRO	181	10.466	60.051	-16.952	1.00	0.00	C
ATOM	2424	O	PRO	181	10.519	59.060	-17.683	1.00	0.00	O
ATOM	2425	CB	PRO	181	8.271	61.200	-17.481	1.00	0.00	C
ATOM	2426	CG	PRO	181	8.540	62.677	-17.236	1.00	0.00	C
ATOM	2427	CD	PRO	181	8.764	62.773	-15.725	1.00	0.00	C
ATOM	2428	HA	PRO	181	8.566	59.576	-16.082	1.00	0.00	H
ATOM	2429	HB2	PRO	181	8.525	60.893	-18.497	1.00	0.00	H
ATOM	2430	HB3	PRO	181	7.211	60.992	-17.338	1.00	0.00	H
ATOM	2431	HG2	PRO	181	9.455	62.969	-17.754	1.00	0.00	H
ATOM	2432	HG3	PRO	181	7.737	63.324	-17.588	1.00	0.00	H
ATOM	2433	HD3	PRO	181	7.843	63.061	-15.215	1.00	0.00	H
ATOM	2434	HD2	PRO	181	9.529	63.517	-15.498	1.00	0.00	H
ATOM	2435	N	TYR	182	11.571	60.743	-16.625	1.00	0.00	N

ATOM	2436	CA	TYR	182	12.925	60.332	-17.015	1.00	0.00	C
ATOM	2437	C	TYR	182	13.433	59.213	-16.088	1.00	0.00	C
ATOM	2438	O	TYR	182	14.311	58.446	-16.478	1.00	0.00	O
ATOM	2439	CB	TYR	182	13.902	61.525	-16.921	1.00	0.00	C
ATOM	2440	CG	TYR	182	13.701	62.599	-17.981	1.00	0.00	C
ATOM	2441	CD1	TYR	182	12.823	63.677	-17.748	1.00	0.00	C
ATOM	2442	CD2	TYR	182	14.405	62.527	-19.202	1.00	0.00	C
ATOM	2443	CE1	TYR	182	12.647	64.675	-18.726	1.00	0.00	C
ATOM	2444	CE2	TYR	182	14.232	63.525	-20.180	1.00	0.00	C
ATOM	2445	CZ	TYR	182	13.353	64.602	-19.941	1.00	0.00	C
ATOM	2446	OH	TYR	182	13.188	65.576	-20.881	1.00	0.00	O
ATOM	2447	H	TYR	182	11.519	61.536	-15.971	1.00	0.00	H
ATOM	2448	HA	TYR	182	12.924	59.952	-18.038	1.00	0.00	H
ATOM	2449	HB2	TYR	182	13.875	61.979	-15.931	1.00	0.00	H
ATOM	2450	HB3	TYR	182	14.925	61.161	-17.026	1.00	0.00	H
ATOM	2451	HD1	TYR	182	12.283	63.741	-16.814	1.00	0.00	H
ATOM	2452	HD2	TYR	182	15.086	61.708	-19.387	1.00	0.00	H
ATOM	2453	HE1	TYR	182	11.971	65.494	-18.538	1.00	0.00	H
ATOM	2454	HE2	TYR	182	14.784	63.453	-21.105	1.00	0.00	H
ATOM	2455	HH	TYR	182	13.654	65.383	-21.679	1.00	0.00	H
ATOM	2456	N	ASN	183	12.835	59.062	-14.898	1.00	0.00	N
ATOM	2457	CA	ASN	183	13.109	58.014	-13.903	1.00	0.00	C
ATOM	2458	C	ASN	183	12.259	56.753	-14.200	1.00	0.00	C
ATOM	2459	O	ASN	183	12.077	55.889	-13.342	1.00	0.00	O
ATOM	2460	CB	ASN	183	12.757	58.597	-12.510	1.00	0.00	C
ATOM	2461	CG	ASN	183	13.364	57.834	-11.328	1.00	0.00	C
ATOM	2462	OD1	ASN	183	14.479	57.325	-11.397	1.00	0.00	O
ATOM	2463	ND2	ASN	183	12.636	57.713	-10.223	1.00	0.00	N
ATOM	2464	H	ASN	183	12.113	59.732	-14.664	1.00	0.00	H
ATOM	2465	HA	ASN	183	14.164	57.745	-13.942	1.00	0.00	H
ATOM	2466	HB2	ASN	183	13.142	59.613	-12.442	1.00	0.00	H
ATOM	2467	HB3	ASN	183	11.675	58.671	-12.388	1.00	0.00	H
ATOM	2468	HD22	ASN	183	13.022	57.183	-9.464	1.00	0.00	H
ATOM	2469	HD21	ASN	183	11.738	58.184	-10.129	1.00	0.00	H
ATOM	2470	N	ASN	184	11.776	56.641	-15.447	1.00	0.00	N
ATOM	2471	CA	ASN	184	10.788	55.733	-16.045	1.00	0.00	C
ATOM	2472	C	ASN	184	9.427	56.438	-16.133	1.00	0.00	C
ATOM	2473	O	ASN	184	9.076	57.127	-15.176	1.00	0.00	O
ATOM	2474	CB	ASN	184	10.676	54.313	-15.434	1.00	0.00	C
ATOM	2475	CG	ASN	184	11.909	53.442	-15.650	1.00	0.00	C
ATOM	2476	OD1	ASN	184	12.003	52.727	-16.640	1.00	0.00	O
ATOM	2477	ND2	ASN	184	12.851	53.476	-14.717	1.00	0.00	N
ATOM	2478	H	ASN	184	12.013	57.439	-16.020	1.00	0.00	H
ATOM	2479	HA	ASN	184	11.171	55.607	-17.056	1.00	0.00	H
ATOM	2480	HB2	ASN	184	10.415	54.355	-14.377	1.00	0.00	H
ATOM	2481	HB3	ASN	184	9.843	53.790	-15.904	1.00	0.00	H
ATOM	2482	HD22	ASN	184	13.692	52.936	-14.832	1.00	0.00	H
ATOM	2483	HD21	ASN	184	12.754	54.159	-13.965	1.00	0.00	H
ATOM	2484	N	PRO	185	8.639	56.278	-17.228	1.00	0.00	N
ATOM	2485	CA	PRO	185	7.279	56.834	-17.369	1.00	0.00	C
ATOM	2486	C	PRO	185	6.395	56.620	-16.126	1.00	0.00	C
ATOM	2487	O	PRO	185	6.233	55.478	-15.685	1.00	0.00	O
ATOM	2488	CB	PRO	185	6.688	56.137	-18.604	1.00	0.00	C
ATOM	2489	CG	PRO	185	7.895	55.786	-19.456	1.00	0.00	C
ATOM	2490	CD	PRO	185	9.030	55.572	-18.452	1.00	0.00	C
ATOM	2491	HA	PRO	185	7.394	57.900	-17.570	1.00	0.00	H
ATOM	2492	HB2	PRO	185	6.172	55.216	-18.325	1.00	0.00	H
ATOM	2493	HB3	PRO	185	5.972	56.766	-19.134	1.00	0.00	H
ATOM	2494	HG2	PRO	185	7.730	54.922	-20.099	1.00	0.00	H
ATOM	2495	HG3	PRO	185	8.140	56.634	-20.098	1.00	0.00	H
ATOM	2496	HD3	PRO	185	9.966	55.955	-18.862	1.00	0.00	H
ATOM	2497	HD2	PRO	185	9.158	54.510	-18.236	1.00	0.00	H
ATOM	2498	N	VAL	186	5.887	57.727	-15.565	1.00	0.00	N

ATOM	2499	CA	VAL	186	5.243	57.896	-14.254	1.00	0.00	C
ATOM	2500	C	VAL	186	4.293	56.747	-13.822	1.00	0.00	C
ATOM	2501	O	VAL	186	3.611	56.225	-14.707	1.00	0.00	O
ATOM	2502	CB	VAL	186	4.609	59.309	-14.174	1.00	0.00	C
ATOM	2503	CG1	VAL	186	5.655	60.416	-14.386	1.00	0.00	C
ATOM	2504	CG2	VAL	186	3.417	59.508	-15.130	1.00	0.00	C
ATOM	2505	H	VAL	186	6.111	58.597	-16.018	1.00	0.00	H
ATOM	2506	HA	VAL	186	6.098	57.899	-13.581	1.00	0.00	H
ATOM	2507	HB	VAL	186	4.228	59.434	-13.158	1.00	0.00	H
ATOM	2508	HG11	VAL	186	5.235	61.396	-14.156	1.00	0.00	H
ATOM	2509	HG12	VAL	186	6.516	60.270	-13.733	1.00	0.00	H
ATOM	2510	HG13	VAL	186	6.020	60.451	-15.410	1.00	0.00	H
ATOM	2511	HG21	VAL	186	3.002	60.511	-15.026	1.00	0.00	H
ATOM	2512	HG22	VAL	186	3.700	59.366	-16.172	1.00	0.00	H
ATOM	2513	HG23	VAL	186	2.609	58.811	-14.902	1.00	0.00	H
ATOM	2514	N	PRO	187	4.277	56.313	-12.534	1.00	0.00	N
ATOM	2515	CA	PRO	187	3.483	55.192	-11.978	1.00	0.00	C
ATOM	2516	C	PRO	187	2.033	54.916	-12.450	1.00	0.00	C
ATOM	2517	O	PRO	187	1.653	53.742	-12.469	1.00	0.00	O
ATOM	2518	CB	PRO	187	3.572	55.342	-10.458	1.00	0.00	C
ATOM	2519	CG	PRO	187	4.892	56.049	-10.214	1.00	0.00	C
ATOM	2520	CD	PRO	187	5.131	56.876	-11.478	1.00	0.00	C
ATOM	2521	HA	PRO	187	4.052	54.302	-12.243	1.00	0.00	H
ATOM	2522	HB2	PRO	187	2.756	55.964	-10.090	1.00	0.00	H
ATOM	2523	HB3	PRO	187	3.522	54.382	-9.946	1.00	0.00	H
ATOM	2524	HG2	PRO	187	4.887	56.661	-9.310	1.00	0.00	H
ATOM	2525	HG3	PRO	187	5.688	55.311	-10.104	1.00	0.00	H
ATOM	2526	HD3	PRO	187	6.191	56.827	-11.733	1.00	0.00	H
ATOM	2527	HD2	PRO	187	4.880	57.924	-11.321	1.00	0.00	H
ATOM	2528	N	ARG	188	1.261	55.925	-12.877	1.00	0.00	N
ATOM	2529	CA	ARG	188	-0.045	55.855	-13.556	1.00	0.00	C
ATOM	2530	C	ARG	188	-1.275	55.982	-12.634	1.00	0.00	C
ATOM	2531	O	ARG	188	-2.318	55.398	-12.924	1.00	0.00	O
ATOM	2532	CB	ARG	188	-0.196	54.726	-14.618	1.00	0.00	C
ATOM	2533	CG	ARG	188	0.886	54.641	-15.715	1.00	0.00	C
ATOM	2534	CD	ARG	188	1.763	53.370	-15.646	1.00	0.00	C
ATOM	2535	NE	ARG	188	3.165	53.667	-15.303	1.00	0.00	N
ATOM	2536	CZ	ARG	188	4.010	52.939	-14.564	1.00	0.00	C
ATOM	2537	NH1	ARG	188	3.576	51.876	-13.888	1.00	0.00	N
ATOM	2538	NH2	ARG	188	5.293	53.293	-14.499	1.00	0.00	N1+
ATOM	2539	H	ARG	188	1.608	56.876	-12.671	1.00	0.00	H
ATOM	2540	HA	ARG	188	-0.068	56.788	-14.121	1.00	0.00	H
ATOM	2541	HB2	ARG	188	-0.331	53.769	-14.114	1.00	0.00	H
ATOM	2542	HB3	ARG	188	-1.147	54.885	-15.128	1.00	0.00	H
ATOM	2543	HG2	ARG	188	0.378	54.634	-16.680	1.00	0.00	H
ATOM	2544	HG3	ARG	188	1.475	55.556	-15.732	1.00	0.00	H
ATOM	2545	HD2	ARG	188	1.339	52.637	-14.962	1.00	0.00	H
ATOM	2546	HD3	ARG	188	1.773	52.887	-16.623	1.00	0.00	H
ATOM	2547	HE	ARG	188	3.418	54.657	-15.440	1.00	0.00	H
ATOM	2548	HH12	ARG	188	4.176	51.283	-13.338	1.00	0.00	H
ATOM	2549	HH11	ARG	188	2.580	51.833	-13.698	1.00	0.00	H
ATOM	2550	HH22	ARG	188	5.992	52.804	-13.964	1.00	0.00	H
ATOM	2551	HH21	ARG	188	5.631	54.138	-14.999	1.00	0.00	H
ATOM	2552	N	GLY	189	-1.182	56.738	-11.540	1.00	0.00	N
ATOM	2553	CA	GLY	189	-2.242	56.961	-10.556	1.00	0.00	C
ATOM	2554	C	GLY	189	-1.843	56.500	-9.150	1.00	0.00	C
ATOM	2555	O	GLY	189	-2.629	56.626	-8.213	1.00	0.00	O
ATOM	2556	H	GLY	189	-0.285	57.202	-11.319	1.00	0.00	H
ATOM	2557	HA2	GLY	189	-2.456	58.029	-10.520	1.00	0.00	H
ATOM	2558	HA3	GLY	189	-3.172	56.462	-10.832	1.00	0.00	H
ATOM	2559	N	LEU	190	-0.621	55.979	-8.997	1.00	0.00	N
ATOM	2560	CA	LEU	190	-0.047	55.498	-7.744	1.00	0.00	C
ATOM	2561	C	LEU	190	0.800	56.551	-7.049	1.00	0.00	C

ATOM	2562	O	LEU	190	1.106	56.422	-5.860	1.00	0.00	O
ATOM	2563	CB	LEU	190	0.853	54.272	-8.031	1.00	0.00	C
ATOM	2564	CG	LEU	190	0.184	53.120	-8.817	1.00	0.00	C
ATOM	2565	CD1	LEU	190	1.216	52.043	-9.185	1.00	0.00	C
ATOM	2566	CD2	LEU	190	-0.994	52.497	-8.050	1.00	0.00	C
ATOM	2567	H	LEU	190	0.003	56.115	-9.786	1.00	0.00	H
ATOM	2568	HA	LEU	190	-0.842	55.206	-7.055	1.00	0.00	H
ATOM	2569	HB2	LEU	190	1.731	54.604	-8.577	1.00	0.00	H
ATOM	2570	HB3	LEU	190	1.234	53.881	-7.085	1.00	0.00	H
ATOM	2571	HG	LEU	190	-0.202	53.513	-9.759	1.00	0.00	H
ATOM	2572	HD11	LEU	190	0.756	51.239	-9.761	1.00	0.00	H
ATOM	2573	HD12	LEU	190	2.017	52.460	-9.796	1.00	0.00	H
ATOM	2574	HD13	LEU	190	1.667	51.602	-8.296	1.00	0.00	H
ATOM	2575	HD21	LEU	190	-1.433	51.670	-8.608	1.00	0.00	H
ATOM	2576	HD22	LEU	190	-0.680	52.113	-7.079	1.00	0.00	H
ATOM	2577	HD23	LEU	190	-1.789	53.223	-7.881	1.00	0.00	H
ATOM	2578	N	GLU	191	1.157	57.593	-7.795	1.00	0.00	N
ATOM	2579	CA	GLU	191	2.036	58.670	-7.441	1.00	0.00	C
ATOM	2580	C	GLU	191	1.636	59.332	-6.121	1.00	0.00	C
ATOM	2581	O	GLU	191	2.405	59.358	-5.163	1.00	0.00	O
ATOM	2582	CB	GLU	191	2.071	59.729	-8.580	1.00	0.00	C
ATOM	2583	CG	GLU	191	2.726	59.302	-9.918	1.00	0.00	C
ATOM	2584	CD	GLU	191	1.837	58.499	-10.863	1.00	0.00	C
ATOM	2585	OE1	GLU	191	0.876	57.873	-10.379	1.00	0.00	O
ATOM	2586	OE2	GLU	191	2.120	58.423	-12.073	1.00	0.00	O1-
ATOM	2587	H	GLU	191	0.889	57.592	-8.791	1.00	0.00	H
ATOM	2588	HA	GLU	191	3.038	58.257	-7.314	1.00	0.00	H
ATOM	2589	HB2	GLU	191	1.065	60.099	-8.784	1.00	0.00	H
ATOM	2590	HB3	GLU	191	2.624	60.596	-8.219	1.00	0.00	H
ATOM	2591	HG2	GLU	191	3.047	60.190	-10.462	1.00	0.00	H
ATOM	2592	HG3	GLU	191	3.629	58.735	-9.706	1.00	0.00	H
ATOM	2593	N	ALA	192	0.383	59.792	-6.066	1.00	0.00	N
ATOM	2594	CA	ALA	192	-0.179	60.541	-4.953	1.00	0.00	C
ATOM	2595	C	ALA	192	-0.256	59.752	-3.660	1.00	0.00	C
ATOM	2596	O	ALA	192	-0.005	60.291	-2.586	1.00	0.00	O
ATOM	2597	CB	ALA	192	-1.596	60.994	-5.342	1.00	0.00	C
ATOM	2598	H	ALA	192	-0.172	59.673	-6.901	1.00	0.00	H
ATOM	2599	HA	ALA	192	0.442	61.423	-4.779	1.00	0.00	H
ATOM	2600	HB1	ALA	192	-2.046	61.594	-4.550	1.00	0.00	H
ATOM	2601	HB2	ALA	192	-1.582	61.602	-6.246	1.00	0.00	H
ATOM	2602	HB3	ALA	192	-2.258	60.146	-5.523	1.00	0.00	H
ATOM	2603	N	PHE	193	-0.566	58.461	-3.791	1.00	0.00	N
ATOM	2604	CA	PHE	193	-0.606	57.532	-2.680	1.00	0.00	C
ATOM	2605	C	PHE	193	0.789	57.287	-2.116	1.00	0.00	C
ATOM	2606	O	PHE	193	0.976	57.232	-0.901	1.00	0.00	O
ATOM	2607	CB	PHE	193	-1.256	56.211	-3.153	1.00	0.00	C
ATOM	2608	CG	PHE	193	-1.380	55.096	-2.123	1.00	0.00	C
ATOM	2609	CD1	PHE	193	-1.902	55.355	-0.836	1.00	0.00	C
ATOM	2610	CD2	PHE	193	-0.975	53.785	-2.453	1.00	0.00	C
ATOM	2611	CE1	PHE	193	-2.014	54.317	0.108	1.00	0.00	C
ATOM	2612	CE2	PHE	193	-1.093	52.745	-1.510	1.00	0.00	C
ATOM	2613	CZ	PHE	193	-1.613	53.011	-0.229	1.00	0.00	C
ATOM	2614	H	PHE	193	-0.676	58.100	-4.726	1.00	0.00	H
ATOM	2615	HA	PHE	193	-1.193	57.961	-1.873	1.00	0.00	H
ATOM	2616	HB2	PHE	193	-2.263	56.420	-3.515	1.00	0.00	H
ATOM	2617	HB3	PHE	193	-0.707	55.824	-4.012	1.00	0.00	H
ATOM	2618	HD1	PHE	193	-2.206	56.353	-0.559	1.00	0.00	H
ATOM	2619	HD2	PHE	193	-0.567	53.567	-3.430	1.00	0.00	H
ATOM	2620	HE1	PHE	193	-2.405	54.524	1.094	1.00	0.00	H
ATOM	2621	HE2	PHE	193	-0.776	51.745	-1.767	1.00	0.00	H
ATOM	2622	HZ	PHE	193	-1.696	52.216	0.498	1.00	0.00	H
ATOM	2623	N	THR	194	1.783	57.216	-3.000	1.00	0.00	N
ATOM	2624	CA	THR	194	3.147	56.956	-2.599	1.00	0.00	C

ATOM	2625	C	THR	194	3.802	58.161	-1.963	1.00	0.00	C
ATOM	2626	O	THR	194	4.487	58.021	-0.952	1.00	0.00	O
ATOM	2627	CB	THR	194	3.948	56.454	-3.827	1.00	0.00	C
ATOM	2628	OG1	THR	194	3.272	55.363	-4.434	1.00	0.00	O
ATOM	2629	CG2	THR	194	5.341	55.920	-3.445	1.00	0.00	C
ATOM	2630	H	THR	194	1.580	57.312	-3.986	1.00	0.00	H
ATOM	2631	HA	THR	194	3.140	56.155	-1.856	1.00	0.00	H
ATOM	2632	HB	THR	194	4.037	57.244	-4.572	1.00	0.00	H
ATOM	2633	HG1	THR	194	2.480	55.688	-4.857	1.00	0.00	H
ATOM	2634	HG21	THR	194	5.838	55.448	-4.291	1.00	0.00	H
ATOM	2635	HG22	THR	194	5.995	56.716	-3.086	1.00	0.00	H
ATOM	2636	HG23	THR	194	5.272	55.170	-2.657	1.00	0.00	H
ATOM	2637	N	VAL	195	3.474	59.347	-2.473	1.00	0.00	N
ATOM	2638	CA	VAL	195	3.736	60.593	-1.795	1.00	0.00	C
ATOM	2639	C	VAL	195	3.172	60.638	-0.369	1.00	0.00	C
ATOM	2640	O	VAL	195	3.814	61.157	0.544	1.00	0.00	O
ATOM	2641	CB	VAL	195	3.250	61.790	-2.670	1.00	0.00	C
ATOM	2642	CG1	VAL	195	3.237	63.154	-1.946	1.00	0.00	C
ATOM	2643	CG2	VAL	195	4.085	61.926	-3.954	1.00	0.00	C
ATOM	2644	H	VAL	195	2.927	59.377	-3.329	1.00	0.00	H
ATOM	2645	HA	VAL	195	4.819	60.680	-1.692	1.00	0.00	H
ATOM	2646	HB	VAL	195	2.222	61.591	-2.973	1.00	0.00	H
ATOM	2647	HG11	VAL	195	3.018	63.968	-2.638	1.00	0.00	H
ATOM	2648	HG12	VAL	195	2.475	63.194	-1.167	1.00	0.00	H
ATOM	2649	HG13	VAL	195	4.200	63.367	-1.480	1.00	0.00	H
ATOM	2650	HG21	VAL	195	3.676	62.686	-4.620	1.00	0.00	H
ATOM	2651	HG22	VAL	195	5.112	62.207	-3.725	1.00	0.00	H
ATOM	2652	HG23	VAL	195	4.128	60.996	-4.515	1.00	0.00	H
ATOM	2653	N	GLY	196	2.015	60.011	-0.164	1.00	0.00	N
ATOM	2654	CA	GLY	196	1.386	59.944	1.127	1.00	0.00	C
ATOM	2655	C	GLY	196	2.129	59.077	2.128	1.00	0.00	C
ATOM	2656	O	GLY	196	2.355	59.480	3.266	1.00	0.00	O
ATOM	2657	H	GLY	196	1.521	59.616	-0.955	1.00	0.00	H
ATOM	2658	HA2	GLY	196	1.256	60.952	1.518	1.00	0.00	H
ATOM	2659	HA3	GLY	196	0.435	59.447	1.056	1.00	0.00	H
ATOM	2660	N	LEU	197	2.549	57.893	1.696	1.00	0.00	N
ATOM	2661	CA	LEU	197	3.268	56.954	2.541	1.00	0.00	C
ATOM	2662	C	LEU	197	4.708	57.400	2.798	1.00	0.00	C
ATOM	2663	O	LEU	197	5.283	57.082	3.836	1.00	0.00	O
ATOM	2664	CB	LEU	197	3.348	55.603	1.785	1.00	0.00	C
ATOM	2665	CG	LEU	197	1.995	54.922	1.460	1.00	0.00	C
ATOM	2666	CD1	LEU	197	2.199	53.783	0.450	1.00	0.00	C
ATOM	2667	CD2	LEU	197	1.265	54.404	2.710	1.00	0.00	C
ATOM	2668	H	LEU	197	2.312	57.622	0.747	1.00	0.00	H
ATOM	2669	HA	LEU	197	2.682	56.775	3.430	1.00	0.00	H
ATOM	2670	HB2	LEU	197	3.892	55.760	0.851	1.00	0.00	H
ATOM	2671	HB3	LEU	197	3.959	54.903	2.358	1.00	0.00	H
ATOM	2672	HG	LEU	197	1.335	55.645	0.985	1.00	0.00	H
ATOM	2673	HD11	LEU	197	1.250	53.316	0.187	1.00	0.00	H
ATOM	2674	HD12	LEU	197	2.640	54.150	-0.477	1.00	0.00	H
ATOM	2675	HD13	LEU	197	2.853	53.006	0.848	1.00	0.00	H
ATOM	2676	HD21	LEU	197	0.336	53.904	2.436	1.00	0.00	H
ATOM	2677	HD22	LEU	197	1.875	53.689	3.263	1.00	0.00	H
ATOM	2678	HD23	LEU	197	0.999	55.218	3.383	1.00	0.00	H
ATOM	2679	N	VAL	198	5.270	58.171	1.865	1.00	0.00	N
ATOM	2680	CA	VAL	198	6.511	58.888	2.053	1.00	0.00	C
ATOM	2681	C	VAL	198	6.418	59.898	3.185	1.00	0.00	C
ATOM	2682	O	VAL	198	7.233	59.886	4.108	1.00	0.00	O
ATOM	2683	CB	VAL	198	6.979	59.508	0.698	1.00	0.00	C
ATOM	2684	CG1	VAL	198	8.023	60.642	0.808	1.00	0.00	C
ATOM	2685	CG2	VAL	198	7.540	58.416	-0.234	1.00	0.00	C
ATOM	2686	H	VAL	198	4.774	58.308	0.989	1.00	0.00	H
ATOM	2687	HA	VAL	198	7.272	58.164	2.338	1.00	0.00	H

ATOM	2688	HB	VAL	198	6.109	59.942	0.209	1.00	0.00	H
ATOM	2689	HG11	VAL	198	8.351	60.970	-0.179	1.00	0.00	H
ATOM	2690	HG12	VAL	198	7.619	61.524	1.308	1.00	0.00	H
ATOM	2691	HG13	VAL	198	8.907	60.319	1.360	1.00	0.00	H
ATOM	2692	HG21	VAL	198	7.693	58.799	-1.244	1.00	0.00	H
ATOM	2693	HG22	VAL	198	8.499	58.043	0.128	1.00	0.00	H
ATOM	2694	HG23	VAL	198	6.874	57.557	-0.310	1.00	0.00	H
ATOM	2695	N	VAL	199	5.375	60.724	3.134	1.00	0.00	N
ATOM	2696	CA	VAL	199	5.053	61.644	4.198	1.00	0.00	C
ATOM	2697	C	VAL	199	4.824	60.959	5.544	1.00	0.00	C
ATOM	2698	O	VAL	199	5.342	61.395	6.575	1.00	0.00	O
ATOM	2699	CB	VAL	199	3.882	62.571	3.755	1.00	0.00	C
ATOM	2700	CG1	VAL	199	3.264	63.402	4.895	1.00	0.00	C
ATOM	2701	CG2	VAL	199	4.319	63.539	2.641	1.00	0.00	C
ATOM	2702	H	VAL	199	4.765	60.696	2.323	1.00	0.00	H
ATOM	2703	HA	VAL	199	5.929	62.280	4.344	1.00	0.00	H
ATOM	2704	HB	VAL	199	3.089	61.945	3.347	1.00	0.00	H
ATOM	2705	HG11	VAL	199	2.483	64.063	4.518	1.00	0.00	H
ATOM	2706	HG12	VAL	199	2.806	62.769	5.655	1.00	0.00	H
ATOM	2707	HG13	VAL	199	4.013	64.023	5.389	1.00	0.00	H
ATOM	2708	HG21	VAL	199	3.458	64.030	2.187	1.00	0.00	H
ATOM	2709	HG22	VAL	199	4.970	64.318	3.039	1.00	0.00	H
ATOM	2710	HG23	VAL	199	4.865	63.038	1.843	1.00	0.00	H
ATOM	2711	N	LEU	200	4.108	59.837	5.493	1.00	0.00	N
ATOM	2712	CA	LEU	200	3.972	58.883	6.569	1.00	0.00	C
ATOM	2713	C	LEU	200	5.288	58.508	7.258	1.00	0.00	C
ATOM	2714	O	LEU	200	5.412	58.633	8.481	1.00	0.00	O
ATOM	2715	CB	LEU	200	3.141	57.665	6.131	1.00	0.00	C
ATOM	2716	CG	LEU	200	3.479	56.243	6.671	1.00	0.00	C
ATOM	2717	CD1	LEU	200	3.192	56.048	8.162	1.00	0.00	C
ATOM	2718	CD2	LEU	200	2.779	55.153	5.852	1.00	0.00	C
ATOM	2719	H	LEU	200	3.681	59.612	4.599	1.00	0.00	H
ATOM	2720	HA	LEU	200	3.414	59.401	7.341	1.00	0.00	H
ATOM	2721	HB2	LEU	200	2.092	57.911	6.216	1.00	0.00	H
ATOM	2722	HB3	LEU	200	3.284	57.629	5.078	1.00	0.00	H
ATOM	2723	HG	LEU	200	4.540	56.047	6.526	1.00	0.00	H
ATOM	2724	HD11	LEU	200	2.131	56.016	8.352	1.00	0.00	H
ATOM	2725	HD12	LEU	200	3.605	55.100	8.501	1.00	0.00	H
ATOM	2726	HD13	LEU	200	3.611	56.830	8.790	1.00	0.00	H
ATOM	2727	HD21	LEU	200	1.718	55.360	5.742	1.00	0.00	H
ATOM	2728	HD22	LEU	200	3.214	55.082	4.856	1.00	0.00	H
ATOM	2729	HD23	LEU	200	2.888	54.171	6.312	1.00	0.00	H
ATOM	2730	N	VAL	201	6.258	58.001	6.494	1.00	0.00	N
ATOM	2731	CA	VAL	201	7.469	57.476	7.096	1.00	0.00	C
ATOM	2732	C	VAL	201	8.396	58.571	7.604	1.00	0.00	C
ATOM	2733	O	VAL	201	9.034	58.380	8.638	1.00	0.00	O
ATOM	2734	CB	VAL	201	8.227	56.491	6.162	1.00	0.00	C
ATOM	2735	CG1	VAL	201	7.425	55.197	5.934	1.00	0.00	C
ATOM	2736	CG2	VAL	201	8.687	57.078	4.821	1.00	0.00	C
ATOM	2737	H	VAL	201	6.105	57.884	5.496	1.00	0.00	H
ATOM	2738	HA	VAL	201	7.183	56.896	7.976	1.00	0.00	H
ATOM	2739	HB	VAL	201	9.134	56.194	6.693	1.00	0.00	H
ATOM	2740	HG11	VAL	201	8.014	54.463	5.384	1.00	0.00	H
ATOM	2741	HG12	VAL	201	7.133	54.738	6.879	1.00	0.00	H
ATOM	2742	HG13	VAL	201	6.516	55.380	5.362	1.00	0.00	H
ATOM	2743	HG21	VAL	201	9.294	56.363	4.265	1.00	0.00	H
ATOM	2744	HG22	VAL	201	7.828	57.319	4.206	1.00	0.00	H
ATOM	2745	HG23	VAL	201	9.283	57.983	4.940	1.00	0.00	H
ATOM	2746	N	ILE	202	8.390	59.736	6.956	1.00	0.00	N
ATOM	2747	CA	ILE	202	9.052	60.934	7.447	1.00	0.00	C
ATOM	2748	C	ILE	202	8.473	61.389	8.778	1.00	0.00	C
ATOM	2749	O	ILE	202	9.215	61.677	9.718	1.00	0.00	O
ATOM	2750	CB	ILE	202	8.996	62.050	6.350	1.00	0.00	C

ATOM	2751	CG1	ILE	202	9.816	61.650	5.096	1.00	0.00	C
ATOM	2752	CG2	ILE	202	9.466	63.438	6.853	1.00	0.00	C
ATOM	2753	CD1	ILE	202	9.536	62.508	3.850	1.00	0.00	C
ATOM	2754	H	ILE	202	7.842	59.812	6.103	1.00	0.00	H
ATOM	2755	HA	ILE	202	10.104	60.697	7.621	1.00	0.00	H
ATOM	2756	HB	ILE	202	7.953	62.159	6.046	1.00	0.00	H
ATOM	2757	HG12	ILE	202	10.882	61.681	5.328	1.00	0.00	H
ATOM	2758	HG13	ILE	202	9.613	60.613	4.829	1.00	0.00	H
ATOM	2759	HG21	ILE	202	9.434	64.190	6.066	1.00	0.00	H
ATOM	2760	HG22	ILE	202	8.836	63.823	7.655	1.00	0.00	H
ATOM	2761	HG23	ILE	202	10.490	63.397	7.227	1.00	0.00	H
ATOM	2762	HD11	ILE	202	10.063	62.107	2.983	1.00	0.00	H
ATOM	2763	HD12	ILE	202	8.473	62.520	3.610	1.00	0.00	H
ATOM	2764	HD13	ILE	202	9.867	63.539	3.974	1.00	0.00	H
ATOM	2765	N	GLY	203	7.147	61.327	8.889	1.00	0.00	N
ATOM	2766	CA	GLY	203	6.468	61.685	10.107	1.00	0.00	C
ATOM	2767	C	GLY	203	6.697	60.695	11.229	1.00	0.00	C
ATOM	2768	O	GLY	203	6.664	61.044	12.401	1.00	0.00	O
ATOM	2769	H	GLY	203	6.586	61.139	8.061	1.00	0.00	H
ATOM	2770	HA2	GLY	203	6.820	62.659	10.441	1.00	0.00	H
ATOM	2771	HA3	GLY	203	5.399	61.780	9.917	1.00	0.00	H
ATOM	2772	N	THR	204	6.937	59.435	10.900	1.00	0.00	N
ATOM	2773	CA	THR	204	7.164	58.437	11.922	1.00	0.00	C
ATOM	2774	C	THR	204	8.632	58.399	12.342	1.00	0.00	C
ATOM	2775	O	THR	204	8.911	58.138	13.511	1.00	0.00	O
ATOM	2776	CB	THR	204	6.785	57.057	11.332	1.00	0.00	C
ATOM	2777	OG1	THR	204	5.456	57.121	10.858	1.00	0.00	O
ATOM	2778	CG2	THR	204	6.819	55.905	12.352	1.00	0.00	C
ATOM	2779	H	THR	204	6.949	59.189	9.920	1.00	0.00	H
ATOM	2780	HA	THR	204	6.648	58.609	12.856	1.00	0.00	H
ATOM	2781	HB	THR	204	7.437	56.809	10.491	1.00	0.00	H
ATOM	2782	HG1	THR	204	5.439	57.681	10.079	1.00	0.00	H
ATOM	2783	HG21	THR	204	6.518	54.967	11.891	1.00	0.00	H
ATOM	2784	HG22	THR	204	7.821	55.747	12.751	1.00	0.00	H
ATOM	2785	HG23	THR	204	6.156	56.096	13.195	1.00	0.00	H
ATOM	2786	N	SER	205	9.560	58.640	11.416	1.00	0.00	N
ATOM	2787	CA	SER	205	10.973	58.576	11.722	1.00	0.00	C
ATOM	2788	C	SER	205	11.488	59.854	12.377	1.00	0.00	C
ATOM	2789	O	SER	205	12.330	59.777	13.267	1.00	0.00	O
ATOM	2790	CB	SER	205	11.773	58.269	10.435	1.00	0.00	C
ATOM	2791	OG	SER	205	11.514	59.169	9.372	1.00	0.00	O
ATOM	2792	H	SER	205	9.288	58.867	10.467	1.00	0.00	H
ATOM	2793	HA	SER	205	11.164	57.760	12.423	1.00	0.00	H
ATOM	2794	HB2	SER	205	12.843	58.295	10.654	1.00	0.00	H
ATOM	2795	HB3	SER	205	11.558	57.256	10.093	1.00	0.00	H
ATOM	2796	HG	SER	205	10.656	58.955	9.011	1.00	0.00	H
ATOM	2797	N	MET	206	10.974	61.009	11.950	1.00	0.00	N
ATOM	2798	CA	MET	206	11.482	62.316	12.322	1.00	0.00	C
ATOM	2799	C	MET	206	10.365	63.259	12.750	1.00	0.00	C
ATOM	2800	O	MET	206	10.436	64.473	12.560	1.00	0.00	O
ATOM	2801	CB	MET	206	12.398	62.886	11.210	1.00	0.00	C
ATOM	2802	CG	MET	206	13.748	62.170	11.010	1.00	0.00	C
ATOM	2803	SD	MET	206	15.108	62.686	12.102	1.00	0.00	S
ATOM	2804	CE	MET	206	14.958	61.535	13.497	1.00	0.00	C
ATOM	2805	H	MET	206	10.311	60.997	11.181	1.00	0.00	H
ATOM	2806	HA	MET	206	12.088	62.240	13.224	1.00	0.00	H
ATOM	2807	HB2	MET	206	11.844	62.863	10.270	1.00	0.00	H
ATOM	2808	HB3	MET	206	12.611	63.941	11.394	1.00	0.00	H
ATOM	2809	HG2	MET	206	13.644	61.086	11.026	1.00	0.00	H
ATOM	2810	HG3	MET	206	14.088	62.407	10.002	1.00	0.00	H
ATOM	2811	HE1	MET	206	15.804	61.660	14.172	1.00	0.00	H
ATOM	2812	HE2	MET	206	14.050	61.718	14.069	1.00	0.00	H
ATOM	2813	HE3	MET	206	14.950	60.504	13.146	1.00	0.00	H

ATOM	2814	N	GLY	207	9.315	62.695	13.347	1.00	0.00	N
ATOM	2815	CA	GLY	207	8.133	63.459	13.684	1.00	0.00	C
ATOM	2816	C	GLY	207	7.983	63.768	15.170	1.00	0.00	C
ATOM	2817	O	GLY	207	7.160	64.609	15.527	1.00	0.00	O
ATOM	2818	H	GLY	207	9.288	61.688	13.412	1.00	0.00	H
ATOM	2819	HA2	GLY	207	8.100	64.414	13.158	1.00	0.00	H
ATOM	2820	HA3	GLY	207	7.218	62.972	13.399	1.00	0.00	H
ATOM	2821	N	PHE	208	8.802	63.149	16.029	1.00	0.00	N
ATOM	2822	CA	PHE	208	8.913	63.484	17.454	1.00	0.00	C
ATOM	2823	C	PHE	208	9.645	64.807	17.638	1.00	0.00	C
ATOM	2824	O	PHE	208	9.349	65.548	18.569	1.00	0.00	O
ATOM	2825	CB	PHE	208	9.707	62.347	18.146	1.00	0.00	C
ATOM	2826	CG	PHE	208	10.050	62.543	19.618	1.00	0.00	C
ATOM	2827	CD1	PHE	208	9.030	62.750	20.571	1.00	0.00	C
ATOM	2828	CD2	PHE	208	11.399	62.533	20.035	1.00	0.00	C
ATOM	2829	CE1	PHE	208	9.356	62.942	21.927	1.00	0.00	C
ATOM	2830	CE2	PHE	208	11.724	62.723	21.391	1.00	0.00	C
ATOM	2831	CZ	PHE	208	10.703	62.925	22.339	1.00	0.00	C
ATOM	2832	H	PHE	208	9.491	62.527	15.635	1.00	0.00	H
ATOM	2833	HA	PHE	208	7.919	63.571	17.895	1.00	0.00	H
ATOM	2834	HB2	PHE	208	9.144	61.417	18.067	1.00	0.00	H
ATOM	2835	HB3	PHE	208	10.637	62.174	17.601	1.00	0.00	H
ATOM	2836	HD1	PHE	208	7.995	62.782	20.264	1.00	0.00	H
ATOM	2837	HD2	PHE	208	12.194	62.388	19.316	1.00	0.00	H
ATOM	2838	HE1	PHE	208	8.574	63.114	22.652	1.00	0.00	H
ATOM	2839	HE2	PHE	208	12.759	62.718	21.704	1.00	0.00	H
ATOM	2840	HZ	PHE	208	10.954	63.081	23.378	1.00	0.00	H
ATOM	2841	N	ASN	209	10.583	65.088	16.730	1.00	0.00	N
ATOM	2842	CA	ASN	209	11.536	66.172	16.829	1.00	0.00	C
ATOM	2843	C	ASN	209	10.823	67.523	16.741	1.00	0.00	C
ATOM	2844	O	ASN	209	10.969	68.360	17.626	1.00	0.00	O
ATOM	2845	CB	ASN	209	12.569	66.144	15.661	1.00	0.00	C
ATOM	2846	CG	ASN	209	13.230	64.829	15.249	1.00	0.00	C
ATOM	2847	OD1	ASN	209	12.676	63.744	15.434	1.00	0.00	O
ATOM	2848	ND2	ASN	209	14.403	64.926	14.632	1.00	0.00	N
ATOM	2849	H	ASN	209	10.805	64.385	16.037	1.00	0.00	H
ATOM	2850	HA	ASN	209	12.063	66.093	17.782	1.00	0.00	H
ATOM	2851	HB2	ASN	209	12.155	66.549	14.737	1.00	0.00	H
ATOM	2852	HB3	ASN	209	13.376	66.819	15.945	1.00	0.00	H
ATOM	2853	HD22	ASN	209	14.842	64.117	14.226	1.00	0.00	H
ATOM	2854	HD21	ASN	209	14.800	65.861	14.405	1.00	0.00	H
ATOM	2855	N	SER	210	10.054	67.720	15.664	1.00	0.00	N
ATOM	2856	CA	SER	210	9.537	69.022	15.260	1.00	0.00	C
ATOM	2857	C	SER	210	8.134	69.011	14.664	1.00	0.00	C
ATOM	2858	O	SER	210	7.696	69.993	14.067	1.00	0.00	O
ATOM	2859	CB	SER	210	10.648	69.834	14.550	1.00	0.00	C
ATOM	2860	OG	SER	210	11.343	69.053	13.589	1.00	0.00	O
ATOM	2861	H	SER	210	10.100	67.009	14.952	1.00	0.00	H
ATOM	2862	HA	SER	210	9.344	69.581	16.176	1.00	0.00	H
ATOM	2863	HB2	SER	210	10.239	70.724	14.070	1.00	0.00	H
ATOM	2864	HB3	SER	210	11.379	70.188	15.280	1.00	0.00	H
ATOM	2865	HG	SER	210	12.237	68.896	13.952	1.00	0.00	H
ATOM	2866	N	GLY	211	7.413	67.893	14.811	1.00	0.00	N
ATOM	2867	CA	GLY	211	6.006	67.810	14.435	1.00	0.00	C
ATOM	2868	C	GLY	211	5.824	67.528	12.943	1.00	0.00	C
ATOM	2869	O	GLY	211	4.702	67.617	12.454	1.00	0.00	O
ATOM	2870	H	GLY	211	7.801	67.141	15.367	1.00	0.00	H
ATOM	2871	HA2	GLY	211	5.541	66.999	14.996	1.00	0.00	H
ATOM	2872	HA3	GLY	211	5.472	68.724	14.700	1.00	0.00	H
ATOM	2873	N	TYR	212	6.937	67.246	12.237	1.00	0.00	N
ATOM	2874	CA	TYR	212	7.097	66.975	10.802	1.00	0.00	C
ATOM	2875	C	TYR	212	6.315	67.961	9.916	1.00	0.00	C
ATOM	2876	O	TYR	212	5.635	67.556	8.977	1.00	0.00	O

ATOM	2877	CB	TYR	212	6.841	65.485	10.461	1.00	0.00	C
ATOM	2878	CG	TYR	212	5.456	64.912	10.719	1.00	0.00	C
ATOM	2879	CD1	TYR	212	4.549	64.741	9.653	1.00	0.00	C
ATOM	2880	CD2	TYR	212	5.080	64.518	12.019	1.00	0.00	C
ATOM	2881	CE1	TYR	212	3.275	64.198	9.893	1.00	0.00	C
ATOM	2882	CE2	TYR	212	3.799	64.002	12.261	1.00	0.00	C
ATOM	2883	CZ	TYR	212	2.898	63.835	11.196	1.00	0.00	C
ATOM	2884	OH	TYR	212	1.678	63.281	11.427	1.00	0.00	O
ATOM	2885	H	TYR	212	7.779	67.255	12.790	1.00	0.00	H
ATOM	2886	HA	TYR	212	8.145	67.160	10.574	1.00	0.00	H
ATOM	2887	HB2	TYR	212	7.076	65.327	9.407	1.00	0.00	H
ATOM	2888	HB3	TYR	212	7.567	64.871	10.991	1.00	0.00	H
ATOM	2889	HD1	TYR	212	4.822	65.035	8.649	1.00	0.00	H
ATOM	2890	HD2	TYR	212	5.755	64.645	12.849	1.00	0.00	H
ATOM	2891	HE1	TYR	212	2.592	64.058	9.075	1.00	0.00	H
ATOM	2892	HE2	TYR	212	3.523	63.721	13.267	1.00	0.00	H
ATOM	2893	HH	TYR	212	1.747	62.701	12.175	1.00	0.00	H
ATOM	2894	N	ALA	213	6.423	69.261	10.241	1.00	0.00	N
ATOM	2895	CA	ALA	213	5.694	70.370	9.632	1.00	0.00	C
ATOM	2896	C	ALA	213	5.751	70.375	8.100	1.00	0.00	C
ATOM	2897	O	ALA	213	4.699	70.380	7.460	1.00	0.00	O
ATOM	2898	CB	ALA	213	6.194	71.708	10.209	1.00	0.00	C
ATOM	2899	H	ALA	213	7.006	69.486	11.032	1.00	0.00	H
ATOM	2900	HA	ALA	213	4.649	70.242	9.917	1.00	0.00	H
ATOM	2901	HB1	ALA	213	5.535	72.524	9.911	1.00	0.00	H
ATOM	2902	HB2	ALA	213	6.231	71.696	11.297	1.00	0.00	H
ATOM	2903	HB3	ALA	213	7.197	71.957	9.861	1.00	0.00	H
ATOM	2904	N	VAL	214	6.978	70.392	7.548	1.00	0.00	N
ATOM	2905	CA	VAL	214	7.427	70.285	6.143	1.00	0.00	C
ATOM	2906	C	VAL	214	6.666	71.160	5.112	1.00	0.00	C
ATOM	2907	O	VAL	214	6.780	70.976	3.898	1.00	0.00	O
ATOM	2908	CB	VAL	214	7.519	68.793	5.697	1.00	0.00	C
ATOM	2909	CG1	VAL	214	8.476	67.982	6.592	1.00	0.00	C
ATOM	2910	CG2	VAL	214	6.164	68.068	5.554	1.00	0.00	C
ATOM	2911	H	VAL	214	7.739	70.371	8.223	1.00	0.00	H
ATOM	2912	HA	VAL	214	8.435	70.694	6.150	1.00	0.00	H
ATOM	2913	HB	VAL	214	7.979	68.795	4.707	1.00	0.00	H
ATOM	2914	HG11	VAL	214	8.642	66.984	6.186	1.00	0.00	H
ATOM	2915	HG12	VAL	214	9.450	68.468	6.667	1.00	0.00	H
ATOM	2916	HG13	VAL	214	8.086	67.868	7.601	1.00	0.00	H
ATOM	2917	HG21	VAL	214	6.303	67.037	5.229	1.00	0.00	H
ATOM	2918	HG22	VAL	214	5.619	68.029	6.494	1.00	0.00	H
ATOM	2919	HG23	VAL	214	5.521	68.557	4.823	1.00	0.00	H
ATOM	2920	N	ASN	215	5.856	72.099	5.599	1.00	0.00	N
ATOM	2921	CA	ASN	215	4.828	72.822	4.878	1.00	0.00	C
ATOM	2922	C	ASN	215	4.549	74.101	5.677	1.00	0.00	C
ATOM	2923	O	ASN	215	3.855	74.010	6.695	1.00	0.00	O
ATOM	2924	CB	ASN	215	3.576	71.917	4.723	1.00	0.00	C
ATOM	2925	CG	ASN	215	2.477	72.515	3.842	1.00	0.00	C
ATOM	2926	OD1	ASN	215	2.287	73.727	3.794	1.00	0.00	O
ATOM	2927	ND2	ASN	215	1.762	71.679	3.100	1.00	0.00	N
ATOM	2928	H	ASN	215	5.785	72.120	6.607	1.00	0.00	H
ATOM	2929	HA	ASN	215	5.201	73.060	3.889	1.00	0.00	H
ATOM	2930	HB2	ASN	215	3.879	70.963	4.291	1.00	0.00	H
ATOM	2931	HB3	ASN	215	3.154	71.677	5.697	1.00	0.00	H
ATOM	2932	HD22	ASN	215	1.087	72.051	2.425	1.00	0.00	H
ATOM	2933	HD21	ASN	215	1.920	70.685	3.140	1.00	0.00	H
ATOM	2934	N	PRO	216	5.039	75.283	5.238	1.00	0.00	N
ATOM	2935	CA	PRO	216	4.752	76.576	5.873	1.00	0.00	C
ATOM	2936	C	PRO	216	3.256	76.880	6.046	1.00	0.00	C
ATOM	2937	O	PRO	216	2.884	77.504	7.037	1.00	0.00	O
ATOM	2938	CB	PRO	216	5.411	77.633	4.971	1.00	0.00	C
ATOM	2939	CG	PRO	216	6.496	76.881	4.223	1.00	0.00	C

ATOM	2940	CD	PRO	216	5.995	75.443	4.143	1.00	0.00	C
ATOM	2941	HA	PRO	216	5.215	76.579	6.858	1.00	0.00	H
ATOM	2942	HB2	PRO	216	4.702	78.047	4.255	1.00	0.00	H
ATOM	2943	HB3	PRO	216	5.814	78.468	5.546	1.00	0.00	H
ATOM	2944	HG2	PRO	216	6.747	77.312	3.254	1.00	0.00	H
ATOM	2945	HG3	PRO	216	7.405	76.892	4.825	1.00	0.00	H
ATOM	2946	HD3	PRO	216	6.811	74.724	4.196	1.00	0.00	H
ATOM	2947	HD2	PRO	216	5.473	75.298	3.199	1.00	0.00	H
ATOM	2948	N	ALA	217	2.383	76.423	5.132	1.00	0.00	N
ATOM	2949	CA	ALA	217	0.944	76.643	5.228	1.00	0.00	C
ATOM	2950	C	ALA	217	0.297	75.757	6.299	1.00	0.00	C
ATOM	2951	O	ALA	217	-0.623	76.232	6.957	1.00	0.00	O
ATOM	2952	CB	ALA	217	0.264	76.374	3.876	1.00	0.00	C
ATOM	2953	H	ALA	217	2.714	75.807	4.400	1.00	0.00	H
ATOM	2954	HA	ALA	217	0.762	77.681	5.508	1.00	0.00	H
ATOM	2955	HB1	ALA	217	-0.816	76.502	3.959	1.00	0.00	H
ATOM	2956	HB2	ALA	217	0.612	77.073	3.116	1.00	0.00	H
ATOM	2957	HB3	ALA	217	0.431	75.362	3.511	1.00	0.00	H
ATOM	2958	N	ARG	218	0.788	74.525	6.527	1.00	0.00	N
ATOM	2959	CA	ARG	218	0.261	73.620	7.561	1.00	0.00	C
ATOM	2960	C	ARG	218	0.701	74.045	8.972	1.00	0.00	C
ATOM	2961	O	ARG	218	0.155	73.519	9.939	1.00	0.00	O
ATOM	2962	CB	ARG	218	0.713	72.154	7.318	1.00	0.00	C
ATOM	2963	CG	ARG	218	-0.019	71.093	8.193	1.00	0.00	C
ATOM	2964	CD	ARG	218	0.805	70.491	9.360	1.00	0.00	C
ATOM	2965	NE	ARG	218	-0.052	69.782	10.343	1.00	0.00	N
ATOM	2966	CZ	ARG	218	-0.623	70.308	11.443	1.00	0.00	C
ATOM	2967	NH1	ARG	218	-0.485	71.606	11.699	1.00	0.00	N
ATOM	2968	NH2	ARG	218	-1.337	69.544	12.278	1.00	0.00	N1+
ATOM	2969	H	ARG	218	1.595	74.230	5.991	1.00	0.00	H
ATOM	2970	HA	ARG	218	-0.830	73.663	7.525	1.00	0.00	H
ATOM	2971	HB2	ARG	218	0.524	71.911	6.272	1.00	0.00	H
ATOM	2972	HB3	ARG	218	1.792	72.063	7.455	1.00	0.00	H
ATOM	2973	HG2	ARG	218	-0.949	71.507	8.582	1.00	0.00	H
ATOM	2974	HG3	ARG	218	-0.334	70.282	7.539	1.00	0.00	H
ATOM	2975	HD2	ARG	218	1.540	69.788	8.964	1.00	0.00	H
ATOM	2976	HD3	ARG	218	1.391	71.252	9.875	1.00	0.00	H
ATOM	2977	HE	ARG	218	-0.072	68.775	10.221	1.00	0.00	H
ATOM	2978	HH12	ARG	218	-0.821	72.091	12.538	1.00	0.00	H
ATOM	2979	HH11	ARG	218	-0.100	72.263	11.010	1.00	0.00	H
ATOM	2980	HH22	ARG	218	-1.753	69.914	13.134	1.00	0.00	H
ATOM	2981	HH21	ARG	218	-1.634	68.592	12.056	1.00	0.00	H
ATOM	2982	N	ASP	219	1.652	74.973	9.116	1.00	0.00	N
ATOM	2983	CA	ASP	219	1.965	75.577	10.408	1.00	0.00	C
ATOM	2984	C	ASP	219	1.373	76.989	10.473	1.00	0.00	C
ATOM	2985	O	ASP	219	0.490	77.233	11.290	1.00	0.00	O
ATOM	2986	CB	ASP	219	3.482	75.598	10.670	1.00	0.00	C
ATOM	2987	CG	ASP	219	3.759	76.073	12.091	1.00	0.00	C
ATOM	2988	OD1	ASP	219	3.750	75.224	13.011	1.00	0.00	O
ATOM	2989	OD2	ASP	219	3.918	77.289	12.315	1.00	0.00	O1-
ATOM	2990	H	ASP	219	2.093	75.344	8.287	1.00	0.00	H
ATOM	2991	HA	ASP	219	1.515	75.010	11.227	1.00	0.00	H
ATOM	2992	HB2	ASP	219	3.899	74.597	10.558	1.00	0.00	H
ATOM	2993	HB3	ASP	219	4.008	76.238	9.962	1.00	0.00	H
ATOM	2994	N	PHE	220	1.825	77.908	9.606	1.00	0.00	N
ATOM	2995	CA	PHE	220	1.613	79.353	9.738	1.00	0.00	C
ATOM	2996	C	PHE	220	0.155	79.774	9.736	1.00	0.00	C
ATOM	2997	O	PHE	220	-0.256	80.619	10.531	1.00	0.00	O
ATOM	2998	CB	PHE	220	2.385	80.117	8.641	1.00	0.00	C
ATOM	2999	CG	PHE	220	2.319	81.640	8.714	1.00	0.00	C
ATOM	3000	CD1	PHE	220	2.613	82.318	9.919	1.00	0.00	C
ATOM	3001	CD2	PHE	220	1.953	82.386	7.572	1.00	0.00	C
ATOM	3002	CE1	PHE	220	2.537	83.723	9.982	1.00	0.00	C

ATOM	3003	CE2	PHE	220	1.878	83.791	7.636	1.00	0.00	C
ATOM	3004	CZ	PHE	220	2.169	84.460	8.841	1.00	0.00	C
ATOM	3005	H	PHE	220	2.486	77.613	8.895	1.00	0.00	H
ATOM	3006	HA	PHE	220	2.029	79.629	10.708	1.00	0.00	H
ATOM	3007	HB2	PHE	220	3.432	79.820	8.675	1.00	0.00	H
ATOM	3008	HB3	PHE	220	2.019	79.809	7.662	1.00	0.00	H
ATOM	3009	HD1	PHE	220	2.899	81.768	10.804	1.00	0.00	H
ATOM	3010	HD2	PHE	220	1.725	81.887	6.642	1.00	0.00	H
ATOM	3011	HE1	PHE	220	2.762	84.235	10.906	1.00	0.00	H
ATOM	3012	HE2	PHE	220	1.593	84.358	6.762	1.00	0.00	H
ATOM	3013	HZ	PHE	220	2.109	85.537	8.891	1.00	0.00	H
ATOM	3014	N	GLY	221	-0.634	79.121	8.887	1.00	0.00	N
ATOM	3015	CA	GLY	221	-2.052	79.374	8.804	1.00	0.00	C
ATOM	3016	C	GLY	221	-2.776	78.928	10.071	1.00	0.00	C
ATOM	3017	O	GLY	221	-3.337	79.766	10.775	1.00	0.00	O
ATOM	3018	H	GLY	221	-0.241	78.351	8.363	1.00	0.00	H
ATOM	3019	HA2	GLY	221	-2.230	80.439	8.643	1.00	0.00	H
ATOM	3020	HA3	GLY	221	-2.469	78.859	7.938	1.00	0.00	H
ATOM	3021	N	PRO	222	-2.727	77.632	10.414	1.00	0.00	N
ATOM	3022	CA	PRO	222	-3.168	77.130	11.703	1.00	0.00	C
ATOM	3023	C	PRO	222	-2.668	77.820	12.968	1.00	0.00	C
ATOM	3024	O	PRO	222	-3.435	77.997	13.914	1.00	0.00	O
ATOM	3025	CB	PRO	222	-2.759	75.650	11.709	1.00	0.00	C
ATOM	3026	CG	PRO	222	-2.792	75.255	10.248	1.00	0.00	C
ATOM	3027	CD	PRO	222	-2.373	76.531	9.523	1.00	0.00	C
ATOM	3028	HA	PRO	222	-4.259	77.192	11.702	1.00	0.00	H
ATOM	3029	HB2	PRO	222	-1.739	75.535	12.080	1.00	0.00	H
ATOM	3030	HB3	PRO	222	-3.412	75.032	12.325	1.00	0.00	H
ATOM	3031	HG2	PRO	222	-2.171	74.393	10.017	1.00	0.00	H
ATOM	3032	HG3	PRO	222	-3.813	74.997	9.964	1.00	0.00	H
ATOM	3033	HD3	PRO	222	-2.861	76.624	8.557	1.00	0.00	H
ATOM	3034	HD2	PRO	222	-1.296	76.524	9.373	1.00	0.00	H
ATOM	3035	N	ARG	223	-1.404	78.234	12.984	1.00	0.00	N
ATOM	3036	CA	ARG	223	-0.803	78.961	14.083	1.00	0.00	C
ATOM	3037	C	ARG	223	-1.446	80.322	14.287	1.00	0.00	C
ATOM	3038	O	ARG	223	-1.746	80.701	15.418	1.00	0.00	O
ATOM	3039	CB	ARG	223	0.712	79.096	13.815	1.00	0.00	C
ATOM	3040	CG	ARG	223	1.511	79.710	14.976	1.00	0.00	C
ATOM	3041	CD	ARG	223	3.016	79.794	14.708	1.00	0.00	C
ATOM	3042	NE	ARG	223	3.606	78.453	14.594	1.00	0.00	N
ATOM	3043	CZ	ARG	223	4.022	77.630	15.557	1.00	0.00	C
ATOM	3044	NH1	ARG	223	3.982	77.956	16.848	1.00	0.00	N
ATOM	3045	NH2	ARG	223	4.464	76.435	15.205	1.00	0.00	N1+
ATOM	3046	H	ARG	223	-0.785	77.951	12.223	1.00	0.00	H
ATOM	3047	HA	ARG	223	-0.947	78.377	14.995	1.00	0.00	H
ATOM	3048	HB2	ARG	223	1.121	78.104	13.618	1.00	0.00	H
ATOM	3049	HB3	ARG	223	0.884	79.680	12.910	1.00	0.00	H
ATOM	3050	HG2	ARG	223	1.146	80.706	15.216	1.00	0.00	H
ATOM	3051	HG3	ARG	223	1.344	79.110	15.865	1.00	0.00	H
ATOM	3052	HD2	ARG	223	3.199	80.341	13.783	1.00	0.00	H
ATOM	3053	HD3	ARG	223	3.513	80.354	15.499	1.00	0.00	H
ATOM	3054	HE	ARG	223	3.706	78.080	13.609	1.00	0.00	H
ATOM	3055	HH12	ARG	223	4.288	77.319	17.567	1.00	0.00	H
ATOM	3056	HH11	ARG	223	3.534	78.829	17.165	1.00	0.00	H
ATOM	3057	HH22	ARG	223	4.715	75.675	15.848	1.00	0.00	H
ATOM	3058	HH21	ARG	223	4.306	76.107	14.223	1.00	0.00	H
ATOM	3059	N	LEU	224	-1.731	81.006	13.178	1.00	0.00	N
ATOM	3060	CA	LEU	224	-2.494	82.234	13.176	1.00	0.00	C
ATOM	3061	C	LEU	224	-3.921	82.044	13.653	1.00	0.00	C
ATOM	3062	O	LEU	224	-4.460	82.912	14.337	1.00	0.00	O
ATOM	3063	CB	LEU	224	-2.443	82.869	11.764	1.00	0.00	C
ATOM	3064	CG	LEU	224	-3.004	84.306	11.643	1.00	0.00	C
ATOM	3065	CD1	LEU	224	-2.164	85.330	12.425	1.00	0.00	C

ATOM	3066	CD2	LEU	224	-3.109	84.715	10.166	1.00	0.00	C
ATOM	3067	H	LEU	224	-1.438	80.632	12.281	1.00	0.00	H
ATOM	3068	HA	LEU	224	-2.006	82.912	13.876	1.00	0.00	H
ATOM	3069	HB2	LEU	224	-1.410	82.876	11.413	1.00	0.00	H
ATOM	3070	HB3	LEU	224	-2.981	82.228	11.068	1.00	0.00	H
ATOM	3071	HG	LEU	224	-4.016	84.332	12.048	1.00	0.00	H
ATOM	3072	HD11	LEU	224	-2.546	86.341	12.282	1.00	0.00	H
ATOM	3073	HD12	LEU	224	-2.186	85.136	13.498	1.00	0.00	H
ATOM	3074	HD13	LEU	224	-1.121	85.321	12.105	1.00	0.00	H
ATOM	3075	HD21	LEU	224	-3.534	85.715	10.064	1.00	0.00	H
ATOM	3076	HD22	LEU	224	-2.132	84.717	9.683	1.00	0.00	H
ATOM	3077	HD23	LEU	224	-3.754	84.032	9.613	1.00	0.00	H
ATOM	3078	N	PHE	225	-4.507	80.887	13.348	1.00	0.00	N
ATOM	3079	CA	PHE	225	-5.808	80.557	13.878	1.00	0.00	C
ATOM	3080	C	PHE	225	-5.802	80.337	15.389	1.00	0.00	C
ATOM	3081	O	PHE	225	-6.613	80.915	16.106	1.00	0.00	O
ATOM	3082	CB	PHE	225	-6.456	79.353	13.152	1.00	0.00	C
ATOM	3083	CG	PHE	225	-7.929	79.228	13.516	1.00	0.00	C
ATOM	3084	CD1	PHE	225	-8.884	80.033	12.858	1.00	0.00	C
ATOM	3085	CD2	PHE	225	-8.323	78.430	14.612	1.00	0.00	C
ATOM	3086	CE1	PHE	225	-10.208	80.090	13.337	1.00	0.00	C
ATOM	3087	CE2	PHE	225	-9.641	78.505	15.100	1.00	0.00	C
ATOM	3088	CZ	PHE	225	-10.581	79.343	14.471	1.00	0.00	C
ATOM	3089	H	PHE	225	-4.020	80.233	12.749	1.00	0.00	H
ATOM	3090	HA	PHE	225	-6.456	81.417	13.689	1.00	0.00	H
ATOM	3091	HB2	PHE	225	-6.381	79.479	12.071	1.00	0.00	H
ATOM	3092	HB3	PHE	225	-5.947	78.419	13.390	1.00	0.00	H
ATOM	3093	HD1	PHE	225	-8.593	80.650	12.021	1.00	0.00	H
ATOM	3094	HD2	PHE	225	-7.605	77.817	15.138	1.00	0.00	H
ATOM	3095	HE1	PHE	225	-10.931	80.731	12.854	1.00	0.00	H
ATOM	3096	HE2	PHE	225	-9.923	77.934	15.974	1.00	0.00	H
ATOM	3097	HZ	PHE	225	-11.589	79.404	14.858	1.00	0.00	H
ATOM	3098	N	THR	226	-4.848	79.547	15.876	1.00	0.00	N
ATOM	3099	CA	THR	226	-4.724	79.203	17.289	1.00	0.00	C
ATOM	3100	C	THR	226	-4.358	80.444	18.114	1.00	0.00	C
ATOM	3101	O	THR	226	-4.764	80.576	19.268	1.00	0.00	O
ATOM	3102	CB	THR	226	-3.619	78.125	17.417	1.00	0.00	C
ATOM	3103	OG1	THR	226	-3.832	77.097	16.469	1.00	0.00	O
ATOM	3104	CG2	THR	226	-3.595	77.419	18.779	1.00	0.00	C
ATOM	3105	H	THR	226	-4.206	79.127	15.217	1.00	0.00	H
ATOM	3106	HA	THR	226	-5.676	78.799	17.638	1.00	0.00	H
ATOM	3107	HB	THR	226	-2.639	78.564	17.220	1.00	0.00	H
ATOM	3108	HG1	THR	226	-3.742	77.447	15.587	1.00	0.00	H
ATOM	3109	HG21	THR	226	-2.860	76.616	18.780	1.00	0.00	H
ATOM	3110	HG22	THR	226	-3.333	78.102	19.585	1.00	0.00	H
ATOM	3111	HG23	THR	226	-4.564	76.980	19.021	1.00	0.00	H
ATOM	3112	N	ALA	227	-3.644	81.387	17.485	1.00	0.00	N
ATOM	3113	CA	ALA	227	-3.385	82.700	18.022	1.00	0.00	C
ATOM	3114	C	ALA	227	-4.668	83.490	18.311	1.00	0.00	C
ATOM	3115	O	ALA	227	-4.854	83.987	19.422	1.00	0.00	O
ATOM	3116	CB	ALA	227	-2.430	83.487	17.122	1.00	0.00	C
ATOM	3117	H	ALA	227	-3.289	81.182	16.557	1.00	0.00	H
ATOM	3118	HA	ALA	227	-2.879	82.554	18.977	1.00	0.00	H
ATOM	3119	HB1	ALA	227	-2.213	84.469	17.545	1.00	0.00	H
ATOM	3120	HB2	ALA	227	-1.484	82.957	17.044	1.00	0.00	H
ATOM	3121	HB3	ALA	227	-2.828	83.640	16.121	1.00	0.00	H
ATOM	3122	N	LEU	228	-5.577	83.542	17.336	1.00	0.00	N
ATOM	3123	CA	LEU	228	-6.856	84.232	17.457	1.00	0.00	C
ATOM	3124	C	LEU	228	-7.866	83.474	18.333	1.00	0.00	C
ATOM	3125	O	LEU	228	-8.743	84.097	18.924	1.00	0.00	O
ATOM	3126	CB	LEU	228	-7.465	84.395	16.045	1.00	0.00	C
ATOM	3127	CG	LEU	228	-6.673	85.328	15.099	1.00	0.00	C
ATOM	3128	CD1	LEU	228	-7.082	85.093	13.638	1.00	0.00	C

ATOM	3129	CD2	LEU	228	-6.838	86.809	15.475	1.00	0.00	C
ATOM	3130	H	LEU	228	-5.376	83.073	16.461	1.00	0.00	H
ATOM	3131	HA	LEU	228	-6.702	85.212	17.911	1.00	0.00	H
ATOM	3132	HB2	LEU	228	-7.553	83.406	15.590	1.00	0.00	H
ATOM	3133	HB3	LEU	228	-8.490	84.763	16.125	1.00	0.00	H
ATOM	3134	HG	LEU	228	-5.613	85.087	15.173	1.00	0.00	H
ATOM	3135	HD11	LEU	228	-6.494	85.711	12.959	1.00	0.00	H
ATOM	3136	HD12	LEU	228	-6.920	84.053	13.355	1.00	0.00	H
ATOM	3137	HD13	LEU	228	-8.136	85.323	13.477	1.00	0.00	H
ATOM	3138	HD21	LEU	228	-6.278	87.452	14.795	1.00	0.00	H
ATOM	3139	HD22	LEU	228	-7.884	87.117	15.436	1.00	0.00	H
ATOM	3140	HD23	LEU	228	-6.471	87.012	16.481	1.00	0.00	H
ATOM	3141	N	ALA	229	-7.723	82.149	18.447	1.00	0.00	N
ATOM	3142	CA	ALA	229	-8.557	81.282	19.276	1.00	0.00	C
ATOM	3143	C	ALA	229	-8.196	81.357	20.776	1.00	0.00	C
ATOM	3144	O	ALA	229	-8.831	80.679	21.578	1.00	0.00	O
ATOM	3145	CB	ALA	229	-8.383	79.836	18.788	1.00	0.00	C
ATOM	3146	H	ALA	229	-7.042	81.706	17.841	1.00	0.00	H
ATOM	3147	HA	ALA	229	-9.603	81.573	19.165	1.00	0.00	H
ATOM	3148	HB1	ALA	229	-9.091	79.175	19.287	1.00	0.00	H
ATOM	3149	HB2	ALA	229	-8.572	79.752	17.717	1.00	0.00	H
ATOM	3150	HB3	ALA	229	-7.382	79.460	18.989	1.00	0.00	H
ATOM	3151	N	GLY	230	-7.216	82.187	21.162	1.00	0.00	N
ATOM	3152	CA	GLY	230	-6.941	82.563	22.547	1.00	0.00	C
ATOM	3153	C	GLY	230	-5.497	82.336	22.998	1.00	0.00	C
ATOM	3154	O	GLY	230	-5.079	82.939	23.982	1.00	0.00	O
ATOM	3155	H	GLY	230	-6.752	82.712	20.433	1.00	0.00	H
ATOM	3156	HA2	GLY	230	-7.175	83.622	22.661	1.00	0.00	H
ATOM	3157	HA3	GLY	230	-7.589	82.034	23.249	1.00	0.00	H
ATOM	3158	N	TRP	231	-4.699	81.531	22.281	1.00	0.00	N
ATOM	3159	CA	TRP	231	-3.320	81.204	22.674	1.00	0.00	C
ATOM	3160	C	TRP	231	-2.303	82.279	22.219	1.00	0.00	C
ATOM	3161	O	TRP	231	-1.103	82.106	22.435	1.00	0.00	O
ATOM	3162	CB	TRP	231	-2.914	79.835	22.081	1.00	0.00	C
ATOM	3163	CG	TRP	231	-3.586	78.622	22.665	1.00	0.00	C
ATOM	3164	CD1	TRP	231	-3.015	77.775	23.554	1.00	0.00	C
ATOM	3165	CD2	TRP	231	-4.930	78.094	22.426	1.00	0.00	C
ATOM	3166	NE1	TRP	231	-3.897	76.757	23.860	1.00	0.00	N
ATOM	3167	CE2	TRP	231	-5.102	76.922	23.224	1.00	0.00	C
ATOM	3168	CE3	TRP	231	-6.038	78.486	21.638	1.00	0.00	C
ATOM	3169	CZ2	TRP	231	-6.305	76.199	23.262	1.00	0.00	C
ATOM	3170	CZ3	TRP	231	-7.252	77.770	21.667	1.00	0.00	C
ATOM	3171	CH2	TRP	231	-7.389	76.633	22.482	1.00	0.00	C
ATOM	3172	H	TRP	231	-5.079	81.094	21.450	1.00	0.00	H
ATOM	3173	HA	TRP	231	-3.251	81.136	23.762	1.00	0.00	H
ATOM	3174	HB2	TRP	231	-3.048	79.834	21.003	1.00	0.00	H
ATOM	3175	HB3	TRP	231	-1.845	79.683	22.238	1.00	0.00	H
ATOM	3176	HD1	TRP	231	-2.015	77.898	23.943	1.00	0.00	H
ATOM	3177	HE1	TRP	231	-3.727	75.980	24.504	1.00	0.00	H
ATOM	3178	HE3	TRP	231	-5.972	79.368	21.026	1.00	0.00	H
ATOM	3179	HZ2	TRP	231	-6.407	75.337	23.903	1.00	0.00	H
ATOM	3180	HZ3	TRP	231	-8.096	78.113	21.088	1.00	0.00	H
ATOM	3181	HH2	TRP	231	-8.330	76.111	22.526	1.00	0.00	H
ATOM	3182	N	GLY	232	-2.786	83.388	21.632	1.00	0.00	N
ATOM	3183	CA	GLY	232	-2.122	84.576	21.079	1.00	0.00	C
ATOM	3184	C	GLY	232	-0.606	84.505	20.895	1.00	0.00	C
ATOM	3185	O	GLY	232	-0.122	83.908	19.934	1.00	0.00	O
ATOM	3186	H	GLY	232	-3.792	83.381	21.524	1.00	0.00	H
ATOM	3187	HA2	GLY	232	-2.554	84.805	20.107	1.00	0.00	H
ATOM	3188	HA3	GLY	232	-2.372	85.425	21.716	1.00	0.00	H
ATOM	3189	N	SER	233	0.145	85.115	21.815	1.00	0.00	N
ATOM	3190	CA	SER	233	1.599	85.199	21.755	1.00	0.00	C
ATOM	3191	C	SER	233	2.261	83.851	22.097	1.00	0.00	C

ATOM	3192	O	SER	233	3.330	83.556	21.574	1.00	0.00	O
ATOM	3193	CB	SER	233	2.057	86.239	22.797	1.00	0.00	C
ATOM	3194	OG	SER	233	1.304	87.437	22.667	1.00	0.00	O
ATOM	3195	H	SER	233	-0.296	85.681	22.525	1.00	0.00	H
ATOM	3196	HA	SER	233	1.907	85.513	20.755	1.00	0.00	H
ATOM	3197	HB2	SER	233	1.946	85.862	23.816	1.00	0.00	H
ATOM	3198	HB3	SER	233	3.118	86.459	22.656	1.00	0.00	H
ATOM	3199	HG	SER	233	1.729	88.118	23.171	1.00	0.00	H
ATOM	3200	N	ALA	234	1.637	83.007	22.932	1.00	0.00	N
ATOM	3201	CA	ALA	234	2.231	81.784	23.482	1.00	0.00	C
ATOM	3202	C	ALA	234	2.393	80.666	22.434	1.00	0.00	C
ATOM	3203	O	ALA	234	3.204	79.757	22.626	1.00	0.00	O
ATOM	3204	CB	ALA	234	1.334	81.277	24.623	1.00	0.00	C
ATOM	3205	H	ALA	234	0.666	83.193	23.155	1.00	0.00	H
ATOM	3206	HA	ALA	234	3.221	82.022	23.879	1.00	0.00	H
ATOM	3207	HB1	ALA	234	1.798	80.441	25.144	1.00	0.00	H
ATOM	3208	HB2	ALA	234	1.158	82.055	25.366	1.00	0.00	H
ATOM	3209	HB3	ALA	234	0.362	80.934	24.265	1.00	0.00	H
ATOM	3210	N	VAL	235	1.661	80.757	21.319	1.00	0.00	N
ATOM	3211	CA	VAL	235	1.766	79.872	20.160	1.00	0.00	C
ATOM	3212	C	VAL	235	2.596	80.542	19.035	1.00	0.00	C
ATOM	3213	O	VAL	235	2.693	79.994	17.939	1.00	0.00	O
ATOM	3214	CB	VAL	235	0.324	79.463	19.730	1.00	0.00	C
ATOM	3215	CG1	VAL	235	-0.531	80.612	19.169	1.00	0.00	C
ATOM	3216	CG2	VAL	235	0.279	78.254	18.784	1.00	0.00	C
ATOM	3217	H	VAL	235	0.983	81.508	21.272	1.00	0.00	H
ATOM	3218	HA	VAL	235	2.302	78.964	20.439	1.00	0.00	H
ATOM	3219	HB	VAL	235	-0.172	79.122	20.640	1.00	0.00	H
ATOM	3220	HG11	VAL	235	-1.546	80.273	18.974	1.00	0.00	H
ATOM	3221	HG12	VAL	235	-0.603	81.435	19.875	1.00	0.00	H
ATOM	3222	HG13	VAL	235	-0.132	81.006	18.233	1.00	0.00	H
ATOM	3223	HG21	VAL	235	-0.739	77.891	18.661	1.00	0.00	H
ATOM	3224	HG22	VAL	235	0.648	78.499	17.795	1.00	0.00	H
ATOM	3225	HG23	VAL	235	0.879	77.427	19.163	1.00	0.00	H
ATOM	3226	N	PHE	236	3.245	81.685	19.292	1.00	0.00	N
ATOM	3227	CA	PHE	236	4.177	82.355	18.382	1.00	0.00	C
ATOM	3228	C	PHE	236	5.549	82.464	19.053	1.00	0.00	C
ATOM	3229	O	PHE	236	6.503	81.828	18.609	1.00	0.00	O
ATOM	3230	CB	PHE	236	3.615	83.715	17.910	1.00	0.00	C
ATOM	3231	CG	PHE	236	2.871	83.648	16.586	1.00	0.00	C
ATOM	3232	CD1	PHE	236	1.479	83.425	16.546	1.00	0.00	C
ATOM	3233	CD2	PHE	236	3.583	83.807	15.380	1.00	0.00	C
ATOM	3234	CE1	PHE	236	0.814	83.363	15.304	1.00	0.00	C
ATOM	3235	CE2	PHE	236	2.920	83.732	14.141	1.00	0.00	C
ATOM	3236	CZ	PHE	236	1.532	83.508	14.101	1.00	0.00	C
ATOM	3237	H	PHE	236	3.161	82.071	20.226	1.00	0.00	H
ATOM	3238	HA	PHE	236	4.351	81.747	17.492	1.00	0.00	H
ATOM	3239	HB2	PHE	236	2.966	84.151	18.672	1.00	0.00	H
ATOM	3240	HB3	PHE	236	4.428	84.431	17.781	1.00	0.00	H
ATOM	3241	HD1	PHE	236	0.922	83.303	17.465	1.00	0.00	H
ATOM	3242	HD2	PHE	236	4.647	83.993	15.400	1.00	0.00	H
ATOM	3243	HE1	PHE	236	-0.248	83.191	15.267	1.00	0.00	H
ATOM	3244	HE2	PHE	236	3.480	83.856	13.225	1.00	0.00	H
ATOM	3245	HZ	PHE	236	1.017	83.452	13.152	1.00	0.00	H
ATOM	3246	N	THR	237	5.646	83.206	20.159	1.00	0.00	N
ATOM	3247	CA	THR	237	6.793	83.246	21.053	1.00	0.00	C
ATOM	3248	C	THR	237	6.555	82.179	22.134	1.00	0.00	C
ATOM	3249	O	THR	237	6.178	82.482	23.267	1.00	0.00	O
ATOM	3250	CB	THR	237	6.926	84.682	21.631	1.00	0.00	C
ATOM	3251	OG1	THR	237	5.731	85.121	22.254	1.00	0.00	O
ATOM	3252	CG2	THR	237	7.316	85.715	20.563	1.00	0.00	C
ATOM	3253	H	THR	237	4.805	83.624	20.552	1.00	0.00	H
ATOM	3254	HA	THR	237	7.716	82.985	20.544	1.00	0.00	H

ATOM	3255	HB	THR	237	7.709	84.670	22.393	1.00	0.00	H
ATOM	3256	HG1	THR	237	5.524	84.459	22.909	1.00	0.00	H
ATOM	3257	HG21	THR	237	7.457	86.701	21.007	1.00	0.00	H
ATOM	3258	HG22	THR	237	8.250	85.439	20.071	1.00	0.00	H
ATOM	3259	HG23	THR	237	6.551	85.805	19.792	1.00	0.00	H
ATOM	3260	N	THR	238	6.731	80.916	21.738	1.00	0.00	N
ATOM	3261	CA	THR	238	6.716	79.753	22.619	1.00	0.00	C
ATOM	3262	C	THR	238	8.113	79.615	23.271	1.00	0.00	C
ATOM	3263	O	THR	238	8.217	79.230	24.433	1.00	0.00	O
ATOM	3264	CB	THR	238	6.395	78.516	21.743	1.00	0.00	C
ATOM	3265	OG1	THR	238	5.242	78.792	20.959	1.00	0.00	O
ATOM	3266	CG2	THR	238	6.111	77.244	22.557	1.00	0.00	C
ATOM	3267	H	THR	238	6.928	80.742	20.760	1.00	0.00	H
ATOM	3268	HA	THR	238	5.962	79.877	23.399	1.00	0.00	H
ATOM	3269	HB	THR	238	7.222	78.317	21.058	1.00	0.00	H
ATOM	3270	HG1	THR	238	4.546	79.073	21.553	1.00	0.00	H
ATOM	3271	HG21	THR	238	5.909	76.395	21.905	1.00	0.00	H
ATOM	3272	HG22	THR	238	6.964	76.969	23.178	1.00	0.00	H
ATOM	3273	HG23	THR	238	5.251	77.370	23.216	1.00	0.00	H
ATOM	3274	N	GLY	239	9.169	80.004	22.544	1.00	0.00	N
ATOM	3275	CA	GLY	239	10.483	80.350	23.063	1.00	0.00	C
ATOM	3276	C	GLY	239	10.782	81.778	22.598	1.00	0.00	C
ATOM	3277	O	GLY	239	9.881	82.622	22.548	1.00	0.00	O
ATOM	3278	H	GLY	239	8.993	80.315	21.593	1.00	0.00	H
ATOM	3279	HA2	GLY	239	10.521	80.311	24.153	1.00	0.00	H
ATOM	3280	HA3	GLY	239	11.220	79.647	22.676	1.00	0.00	H
ATOM	3281	N	GLN	240	12.021	82.054	22.167	1.00	0.00	N
ATOM	3282	CA	GLN	240	12.394	83.299	21.486	1.00	0.00	C
ATOM	3283	C	GLN	240	11.911	83.185	20.025	1.00	0.00	C
ATOM	3284	O	GLN	240	12.701	82.976	19.114	1.00	0.00	O
ATOM	3285	CB	GLN	240	13.931	83.491	21.593	1.00	0.00	C
ATOM	3286	CG	GLN	240	14.425	84.922	21.248	1.00	0.00	C
ATOM	3287	CD	GLN	240	15.009	85.180	19.851	1.00	0.00	C
ATOM	3288	OE1	GLN	240	15.205	86.333	19.486	1.00	0.00	O
ATOM	3289	NE2	GLN	240	15.290	84.167	19.042	1.00	0.00	N
ATOM	3290	H	GLN	240	12.700	81.308	22.156	1.00	0.00	H
ATOM	3291	HA	GLN	240	11.893	84.142	21.966	1.00	0.00	H
ATOM	3292	HB2	GLN	240	14.211	83.312	22.631	1.00	0.00	H
ATOM	3293	HB3	GLN	240	14.468	82.723	21.034	1.00	0.00	H
ATOM	3294	HG2	GLN	240	13.641	85.655	21.438	1.00	0.00	H
ATOM	3295	HG3	GLN	240	15.227	85.178	21.941	1.00	0.00	H
ATOM	3296	HE22	GLN	240	15.713	84.364	18.151	1.00	0.00	H
ATOM	3297	HE21	GLN	240	15.007	83.220	19.267	1.00	0.00	H
ATOM	3298	N	HIE	241	10.587	83.220	19.832	1.00	0.00	N
ATOM	3299	CA	HIE	241	9.855	82.812	18.632	1.00	0.00	C
ATOM	3300	C	HIE	241	9.683	81.284	18.672	1.00	0.00	C
ATOM	3301	O	HIE	241	9.280	80.730	19.697	1.00	0.00	O
ATOM	3302	CB	HIE	241	10.388	83.401	17.295	1.00	0.00	C
ATOM	3303	CG	HIE	241	10.468	84.899	17.243	1.00	0.00	C
ATOM	3304	ND1	HIE	241	9.375	85.698	16.880	1.00	0.00	N
ATOM	3305	CD2	HIE	241	11.545	85.717	17.501	1.00	0.00	C
ATOM	3306	CE1	HIE	241	9.820	86.947	16.963	1.00	0.00	C
ATOM	3307	NE2	HIE	241	11.104	87.016	17.317	1.00	0.00	N
ATOM	3308	H	HIE	241	10.054	83.232	20.697	1.00	0.00	H
ATOM	3309	HA	HIE	241	8.852	83.220	18.734	1.00	0.00	H
ATOM	3310	HB2	HIE	241	11.377	83.008	17.064	1.00	0.00	H
ATOM	3311	HB3	HIE	241	9.757	83.092	16.464	1.00	0.00	H
ATOM	3312	HD2	HIE	241	12.557	85.473	17.805	1.00	0.00	H
ATOM	3313	HE1	HIE	241	9.207	87.815	16.759	1.00	0.00	H
ATOM	3314	HE2	HIE	241	11.650	87.858	17.453	1.00	0.00	H
ATOM	3315	N	TRP	242	10.008	80.649	17.544	1.00	0.00	N
ATOM	3316	CA	TRP	242	10.022	79.240	17.150	1.00	0.00	C
ATOM	3317	C	TRP	242	9.558	79.250	15.689	1.00	0.00	C

ATOM	3318	O	TRP	242	10.285	78.800	14.811	1.00	0.00	O
ATOM	3319	CB	TRP	242	9.083	78.311	17.969	1.00	0.00	C
ATOM	3320	CG	TRP	242	8.798	76.974	17.333	1.00	0.00	C
ATOM	3321	CD1	TRP	242	7.572	76.533	16.972	1.00	0.00	C
ATOM	3322	CD2	TRP	242	9.738	75.947	16.882	1.00	0.00	C
ATOM	3323	NE1	TRP	242	7.689	75.323	16.320	1.00	0.00	N
ATOM	3324	CE2	TRP	242	9.002	74.919	16.215	1.00	0.00	C
ATOM	3325	CE3	TRP	242	11.142	75.781	16.954	1.00	0.00	C
ATOM	3326	CZ2	TRP	242	9.621	73.794	15.644	1.00	0.00	C
ATOM	3327	CZ3	TRP	242	11.776	74.654	16.392	1.00	0.00	C
ATOM	3328	CH2	TRP	242	11.019	73.663	15.735	1.00	0.00	C
ATOM	3329	H	TRP	242	10.341	81.272	16.823	1.00	0.00	H
ATOM	3330	HA	TRP	242	11.046	78.866	17.191	1.00	0.00	H
ATOM	3331	HB2	TRP	242	9.519	78.124	18.950	1.00	0.00	H
ATOM	3332	HB3	TRP	242	8.127	78.804	18.151	1.00	0.00	H
ATOM	3333	HD1	TRP	242	6.656	77.080	17.136	1.00	0.00	H
ATOM	3334	HE1	TRP	242	6.891	74.808	15.953	1.00	0.00	H
ATOM	3335	HE3	TRP	242	11.745	76.526	17.449	1.00	0.00	H
ATOM	3336	HZ2	TRP	242	9.034	73.037	15.143	1.00	0.00	H
ATOM	3337	HZ3	TRP	242	12.850	74.554	16.465	1.00	0.00	H
ATOM	3338	HH2	TRP	242	11.514	72.807	15.299	1.00	0.00	H
ATOM	3339	N	TRP	243	8.363	79.813	15.456	1.00	0.00	N
ATOM	3340	CA	TRP	243	7.557	79.798	14.229	1.00	0.00	C
ATOM	3341	C	TRP	243	8.301	80.055	12.907	1.00	0.00	C
ATOM	3342	O	TRP	243	7.925	79.500	11.880	1.00	0.00	O
ATOM	3343	CB	TRP	243	6.360	80.747	14.402	1.00	0.00	C
ATOM	3344	CG	TRP	243	6.681	82.211	14.424	1.00	0.00	C
ATOM	3345	CD1	TRP	243	7.220	82.886	15.463	1.00	0.00	C
ATOM	3346	CD2	TRP	243	6.510	83.190	13.357	1.00	0.00	C
ATOM	3347	NE1	TRP	243	7.420	84.203	15.108	1.00	0.00	N
ATOM	3348	CE2	TRP	243	6.974	84.456	13.826	1.00	0.00	C
ATOM	3349	CE3	TRP	243	6.009	83.136	12.035	1.00	0.00	C
ATOM	3350	CZ2	TRP	243	6.923	85.612	13.030	1.00	0.00	C
ATOM	3351	CZ3	TRP	243	5.961	84.287	11.223	1.00	0.00	C
ATOM	3352	CH2	TRP	243	6.413	85.524	11.721	1.00	0.00	C
ATOM	3353	H	TRP	243	7.897	80.164	16.279	1.00	0.00	H
ATOM	3354	HA	TRP	243	7.158	78.784	14.154	1.00	0.00	H
ATOM	3355	HB2	TRP	243	5.649	80.573	13.593	1.00	0.00	H
ATOM	3356	HB3	TRP	243	5.831	80.499	15.322	1.00	0.00	H
ATOM	3357	HD1	TRP	243	7.481	82.439	16.410	1.00	0.00	H
ATOM	3358	HE1	TRP	243	7.912	84.866	15.710	1.00	0.00	H
ATOM	3359	HE3	TRP	243	5.661	82.192	11.640	1.00	0.00	H
ATOM	3360	HZ2	TRP	243	7.284	86.554	13.412	1.00	0.00	H
ATOM	3361	HZ3	TRP	243	5.580	84.215	10.214	1.00	0.00	H
ATOM	3362	HH2	TRP	243	6.383	86.403	11.094	1.00	0.00	H
ATOM	3363	N	TRP	244	9.387	80.842	12.939	1.00	0.00	N
ATOM	3364	CA	TRP	244	10.314	81.062	11.827	1.00	0.00	C
ATOM	3365	C	TRP	244	10.904	79.765	11.245	1.00	0.00	C
ATOM	3366	O	TRP	244	11.237	79.736	10.062	1.00	0.00	O
ATOM	3367	CB	TRP	244	11.462	81.991	12.260	1.00	0.00	C
ATOM	3368	CG	TRP	244	11.085	83.384	12.654	1.00	0.00	C
ATOM	3369	CD1	TRP	244	11.211	83.907	13.893	1.00	0.00	C
ATOM	3370	CD2	TRP	244	10.559	84.459	11.817	1.00	0.00	C
ATOM	3371	NE1	TRP	244	10.786	85.220	13.888	1.00	0.00	N
ATOM	3372	CE2	TRP	244	10.403	85.626	12.627	1.00	0.00	C
ATOM	3373	CE3	TRP	244	10.210	84.574	10.451	1.00	0.00	C
ATOM	3374	CZ2	TRP	244	9.945	86.847	12.103	1.00	0.00	C
ATOM	3375	CZ3	TRP	244	9.741	85.791	9.915	1.00	0.00	C
ATOM	3376	CH2	TRP	244	9.613	86.928	10.737	1.00	0.00	C
ATOM	3377	H	TRP	244	9.610	81.266	13.823	1.00	0.00	H
ATOM	3378	HA	TRP	244	9.752	81.553	11.031	1.00	0.00	H
ATOM	3379	HB2	TRP	244	12.016	81.529	13.079	1.00	0.00	H
ATOM	3380	HB3	TRP	244	12.177	82.085	11.441	1.00	0.00	H

ATOM	3381	HD1	TRP	244	11.597	83.363	14.742	1.00	0.00	H
ATOM	3382	HE1	TRP	244	10.751	85.786	14.726	1.00	0.00	H
ATOM	3383	HE3	TRP	244	10.312	83.713	9.807	1.00	0.00	H
ATOM	3384	HZ2	TRP	244	9.843	87.713	12.739	1.00	0.00	H
ATOM	3385	HZ3	TRP	244	9.484	85.850	8.867	1.00	0.00	H
ATOM	3386	HH2	TRP	244	9.259	87.859	10.320	1.00	0.00	H
ATOM	3387	N	VAL	245	11.025	78.707	12.059	1.00	0.00	N
ATOM	3388	CA	VAL	245	11.450	77.372	11.647	1.00	0.00	C
ATOM	3389	C	VAL	245	10.521	76.738	10.631	1.00	0.00	C
ATOM	3390	O	VAL	245	10.898	76.705	9.463	1.00	0.00	O
ATOM	3391	CB	VAL	245	11.832	76.513	12.893	1.00	0.00	C
ATOM	3392	CG1	VAL	245	12.017	75.014	12.586	1.00	0.00	C
ATOM	3393	CG2	VAL	245	13.107	77.051	13.569	1.00	0.00	C
ATOM	3394	H	VAL	245	10.718	78.804	13.023	1.00	0.00	H
ATOM	3395	HA	VAL	245	12.385	77.526	11.107	1.00	0.00	H
ATOM	3396	HB	VAL	245	11.060	76.604	13.653	1.00	0.00	H
ATOM	3397	HG11	VAL	245	12.430	74.492	13.446	1.00	0.00	H
ATOM	3398	HG12	VAL	245	11.094	74.500	12.328	1.00	0.00	H
ATOM	3399	HG13	VAL	245	12.690	74.866	11.746	1.00	0.00	H
ATOM	3400	HG21	VAL	245	13.323	76.509	14.490	1.00	0.00	H
ATOM	3401	HG22	VAL	245	13.971	76.933	12.917	1.00	0.00	H
ATOM	3402	HG23	VAL	245	13.018	78.106	13.826	1.00	0.00	H
ATOM	3403	N	PRO	246	9.296	76.312	10.987	1.00	0.00	N
ATOM	3404	CA	PRO	246	8.374	75.838	9.968	1.00	0.00	C
ATOM	3405	C	PRO	246	8.015	76.812	8.844	1.00	0.00	C
ATOM	3406	O	PRO	246	7.453	76.385	7.841	1.00	0.00	O
ATOM	3407	CB	PRO	246	7.059	75.556	10.724	1.00	0.00	C
ATOM	3408	CG	PRO	246	7.422	75.417	12.186	1.00	0.00	C
ATOM	3409	CD	PRO	246	8.740	76.161	12.339	1.00	0.00	C
ATOM	3410	HA	PRO	246	8.765	74.914	9.540	1.00	0.00	H
ATOM	3411	HB2	PRO	246	6.364	76.394	10.639	1.00	0.00	H
ATOM	3412	HB3	PRO	246	6.544	74.677	10.338	1.00	0.00	H
ATOM	3413	HG2	PRO	246	6.648	75.793	12.857	1.00	0.00	H
ATOM	3414	HG3	PRO	246	7.571	74.362	12.418	1.00	0.00	H
ATOM	3415	HD3	PRO	246	9.354	75.575	13.014	1.00	0.00	H
ATOM	3416	HD2	PRO	246	8.586	77.138	12.792	1.00	0.00	H
ATOM	3417	N	ILE	247	8.315	78.103	9.002	1.00	0.00	N
ATOM	3418	CA	ILE	247	8.312	79.018	7.890	1.00	0.00	C
ATOM	3419	C	ILE	247	9.474	78.730	6.923	1.00	0.00	C
ATOM	3420	O	ILE	247	9.259	78.289	5.796	1.00	0.00	O
ATOM	3421	CB	ILE	247	8.306	80.510	8.358	1.00	0.00	C
ATOM	3422	CG1	ILE	247	7.055	80.873	9.189	1.00	0.00	C
ATOM	3423	CG2	ILE	247	8.506	81.546	7.224	1.00	0.00	C
ATOM	3424	CD1	ILE	247	5.830	81.313	8.381	1.00	0.00	C
ATOM	3425	H	ILE	247	8.681	78.420	9.887	1.00	0.00	H
ATOM	3426	HA	ILE	247	7.393	78.864	7.321	1.00	0.00	H
ATOM	3427	HB	ILE	247	9.153	80.632	9.025	1.00	0.00	H
ATOM	3428	HG12	ILE	247	6.752	80.034	9.812	1.00	0.00	H
ATOM	3429	HG13	ILE	247	7.317	81.674	9.881	1.00	0.00	H
ATOM	3430	HG21	ILE	247	8.447	82.564	7.609	1.00	0.00	H
ATOM	3431	HG22	ILE	247	9.472	81.456	6.734	1.00	0.00	H
ATOM	3432	HG23	ILE	247	7.752	81.436	6.443	1.00	0.00	H
ATOM	3433	HD11	ILE	247	5.008	81.500	9.064	1.00	0.00	H
ATOM	3434	HD12	ILE	247	5.997	82.241	7.836	1.00	0.00	H
ATOM	3435	HD13	ILE	247	5.518	80.546	7.672	1.00	0.00	H
ATOM	3436	N	VAL	248	10.700	79.048	7.337	1.00	0.00	N
ATOM	3437	CA	VAL	248	11.823	79.180	6.422	1.00	0.00	C
ATOM	3438	C	VAL	248	12.527	77.852	6.214	1.00	0.00	C
ATOM	3439	O	VAL	248	13.045	77.599	5.126	1.00	0.00	O
ATOM	3440	CB	VAL	248	12.838	80.200	7.041	1.00	0.00	C
ATOM	3441	CG1	VAL	248	14.071	80.461	6.150	1.00	0.00	C
ATOM	3442	CG2	VAL	248	12.194	81.561	7.379	1.00	0.00	C
ATOM	3443	H	VAL	248	10.845	79.309	8.309	1.00	0.00	H

ATOM	3444	HA	VAL	248	11.483	79.554	5.454	1.00	0.00	H
ATOM	3445	HB	VAL	248	13.207	79.782	7.980	1.00	0.00	H
ATOM	3446	HG11	VAL	248	14.734	81.203	6.595	1.00	0.00	H
ATOM	3447	HG12	VAL	248	14.666	79.561	6.008	1.00	0.00	H
ATOM	3448	HG13	VAL	248	13.780	80.822	5.163	1.00	0.00	H
ATOM	3449	HG21	VAL	248	12.928	82.245	7.807	1.00	0.00	H
ATOM	3450	HG22	VAL	248	11.782	82.034	6.489	1.00	0.00	H
ATOM	3451	HG23	VAL	248	11.396	81.470	8.114	1.00	0.00	H
ATOM	3452	N	SER	249	12.571	77.019	7.253	1.00	0.00	N
ATOM	3453	CA	SER	249	13.287	75.768	7.223	1.00	0.00	C
ATOM	3454	C	SER	249	12.809	74.809	6.145	1.00	0.00	C
ATOM	3455	O	SER	249	13.649	74.362	5.359	1.00	0.00	O
ATOM	3456	CB	SER	249	13.366	75.116	8.613	1.00	0.00	C
ATOM	3457	OG	SER	249	13.822	76.054	9.566	1.00	0.00	O
ATOM	3458	H	SER	249	12.074	77.235	8.115	1.00	0.00	H
ATOM	3459	HA	SER	249	14.315	76.025	6.954	1.00	0.00	H
ATOM	3460	HB2	SER	249	12.417	74.731	8.965	1.00	0.00	H
ATOM	3461	HB3	SER	249	14.050	74.267	8.586	1.00	0.00	H
ATOM	3462	HG	SER	249	14.722	76.299	9.374	1.00	0.00	H
ATOM	3463	N	PRO	250	11.487	74.578	6.017	1.00	0.00	N
ATOM	3464	CA	PRO	250	10.930	74.003	4.818	1.00	0.00	C
ATOM	3465	C	PRO	250	11.382	74.543	3.467	1.00	0.00	C
ATOM	3466	O	PRO	250	11.794	73.765	2.611	1.00	0.00	O
ATOM	3467	CB	PRO	250	9.410	74.108	4.949	1.00	0.00	C
ATOM	3468	CG	PRO	250	9.198	74.017	6.435	1.00	0.00	C
ATOM	3469	CD	PRO	250	10.416	74.744	7.010	1.00	0.00	C
ATOM	3470	HA	PRO	250	11.205	72.948	4.850	1.00	0.00	H
ATOM	3471	HB2	PRO	250	9.054	75.076	4.596	1.00	0.00	H
ATOM	3472	HB3	PRO	250	8.882	73.328	4.400	1.00	0.00	H
ATOM	3473	HG2	PRO	250	8.235	74.407	6.750	1.00	0.00	H
ATOM	3474	HG3	PRO	250	9.232	72.968	6.737	1.00	0.00	H
ATOM	3475	HD3	PRO	250	10.647	74.334	7.987	1.00	0.00	H
ATOM	3476	HD2	PRO	250	10.190	75.804	7.117	1.00	0.00	H
ATOM	3477	N	LEU	251	11.308	75.861	3.285	1.00	0.00	N
ATOM	3478	CA	LEU	251	11.615	76.522	2.028	1.00	0.00	C
ATOM	3479	C	LEU	251	13.072	76.398	1.600	1.00	0.00	C
ATOM	3480	O	LEU	251	13.357	76.315	0.411	1.00	0.00	O
ATOM	3481	CB	LEU	251	11.250	78.026	2.155	1.00	0.00	C
ATOM	3482	CG	LEU	251	9.798	78.356	1.754	1.00	0.00	C
ATOM	3483	CD1	LEU	251	9.285	79.617	2.465	1.00	0.00	C
ATOM	3484	CD2	LEU	251	9.682	78.534	0.232	1.00	0.00	C
ATOM	3485	H	LEU	251	11.006	76.440	4.055	1.00	0.00	H
ATOM	3486	HA	LEU	251	11.017	76.067	1.237	1.00	0.00	H
ATOM	3487	HB2	LEU	251	11.439	78.356	3.176	1.00	0.00	H
ATOM	3488	HB3	LEU	251	11.916	78.640	1.544	1.00	0.00	H
ATOM	3489	HG	LEU	251	9.158	77.527	2.063	1.00	0.00	H
ATOM	3490	HD11	LEU	251	8.250	79.827	2.195	1.00	0.00	H
ATOM	3491	HD12	LEU	251	9.320	79.506	3.547	1.00	0.00	H
ATOM	3492	HD13	LEU	251	9.882	80.492	2.204	1.00	0.00	H
ATOM	3493	HD21	LEU	251	10.203	79.431	-0.105	1.00	0.00	H
ATOM	3494	HD22	LEU	251	10.123	77.694	-0.304	1.00	0.00	H
ATOM	3495	HD23	LEU	251	8.640	78.623	-0.078	1.00	0.00	H
ATOM	3496	N	LEU	252	13.997	76.371	2.560	1.00	0.00	N
ATOM	3497	CA	LEU	252	15.401	76.174	2.243	1.00	0.00	C
ATOM	3498	C	LEU	252	15.743	74.715	1.963	1.00	0.00	C
ATOM	3499	O	LEU	252	16.678	74.430	1.214	1.00	0.00	O
ATOM	3500	CB	LEU	252	16.275	76.682	3.407	1.00	0.00	C
ATOM	3501	CG	LEU	252	16.252	78.216	3.603	1.00	0.00	C
ATOM	3502	CD1	LEU	252	17.066	78.595	4.844	1.00	0.00	C
ATOM	3503	CD2	LEU	252	16.771	78.994	2.381	1.00	0.00	C
ATOM	3504	H	LEU	252	13.710	76.503	3.523	1.00	0.00	H
ATOM	3505	HA	LEU	252	15.661	76.720	1.335	1.00	0.00	H
ATOM	3506	HB2	LEU	252	15.962	76.190	4.329	1.00	0.00	H

ATOM	3507	HB3	LEU	252	17.310	76.373	3.244	1.00	0.00	H
ATOM	3508	HG	LEU	252	15.221	78.525	3.780	1.00	0.00	H
ATOM	3509	HD11	LEU	252	17.016	79.665	5.046	1.00	0.00	H
ATOM	3510	HD12	LEU	252	16.676	78.075	5.714	1.00	0.00	H
ATOM	3511	HD13	LEU	252	18.116	78.322	4.735	1.00	0.00	H
ATOM	3512	HD21	LEU	252	16.826	80.062	2.591	1.00	0.00	H
ATOM	3513	HD22	LEU	252	17.768	78.661	2.090	1.00	0.00	H
ATOM	3514	HD23	LEU	252	16.114	78.881	1.518	1.00	0.00	H
ATOM	3515	N	GLY	253	14.954	73.795	2.509	1.00	0.00	N
ATOM	3516	CA	GLY	253	15.196	72.380	2.357	1.00	0.00	C
ATOM	3517	C	GLY	253	14.552	71.877	1.098	1.00	0.00	C
ATOM	3518	O	GLY	253	15.191	71.123	0.376	1.00	0.00	O
ATOM	3519	H	GLY	253	14.170	74.097	3.075	1.00	0.00	H
ATOM	3520	HA2	GLY	253	16.259	72.134	2.378	1.00	0.00	H
ATOM	3521	HA3	GLY	253	14.731	71.871	3.197	1.00	0.00	H
ATOM	3522	N	SER	254	13.354	72.367	0.767	1.00	0.00	N
ATOM	3523	CA	SER	254	12.709	72.095	-0.500	1.00	0.00	C
ATOM	3524	C	SER	254	13.596	72.408	-1.707	1.00	0.00	C
ATOM	3525	O	SER	254	13.724	71.586	-2.609	1.00	0.00	O
ATOM	3526	CB	SER	254	11.341	72.795	-0.576	1.00	0.00	C
ATOM	3527	OG	SER	254	11.431	74.203	-0.626	1.00	0.00	O
ATOM	3528	H	SER	254	12.890	73.035	1.380	1.00	0.00	H
ATOM	3529	HA	SER	254	12.539	71.018	-0.520	1.00	0.00	H
ATOM	3530	HB2	SER	254	10.829	72.465	-1.482	1.00	0.00	H
ATOM	3531	HB3	SER	254	10.704	72.511	0.260	1.00	0.00	H
ATOM	3532	HG	SER	254	10.691	74.511	-1.147	1.00	0.00	H
ATOM	3533	N	ILE	255	14.281	73.552	-1.655	1.00	0.00	N
ATOM	3534	CA	ILE	255	15.266	73.958	-2.636	1.00	0.00	C
ATOM	3535	C	ILE	255	16.473	73.026	-2.700	1.00	0.00	C
ATOM	3536	O	ILE	255	16.869	72.611	-3.790	1.00	0.00	O
ATOM	3537	CB	ILE	255	15.677	75.445	-2.386	1.00	0.00	C
ATOM	3538	CG1	ILE	255	14.476	76.402	-2.594	1.00	0.00	C
ATOM	3539	CG2	ILE	255	16.865	75.919	-3.258	1.00	0.00	C
ATOM	3540	CD1	ILE	255	14.714	77.819	-2.048	1.00	0.00	C
ATOM	3541	H	ILE	255	14.060	74.190	-0.901	1.00	0.00	H
ATOM	3542	HA	ILE	255	14.788	73.907	-3.617	1.00	0.00	H
ATOM	3543	HB	ILE	255	15.992	75.524	-1.344	1.00	0.00	H
ATOM	3544	HG12	ILE	255	14.216	76.455	-3.652	1.00	0.00	H
ATOM	3545	HG13	ILE	255	13.589	76.004	-2.102	1.00	0.00	H
ATOM	3546	HG21	ILE	255	17.115	76.963	-3.076	1.00	0.00	H
ATOM	3547	HG22	ILE	255	17.778	75.358	-3.055	1.00	0.00	H
ATOM	3548	HG23	ILE	255	16.641	75.814	-4.319	1.00	0.00	H
ATOM	3549	HD11	ILE	255	13.780	78.377	-2.001	1.00	0.00	H
ATOM	3550	HD12	ILE	255	15.116	77.790	-1.035	1.00	0.00	H
ATOM	3551	HD13	ILE	255	15.405	78.387	-2.670	1.00	0.00	H
ATOM	3552	N	ALA	256	17.032	72.683	-1.536	1.00	0.00	N
ATOM	3553	CA	ALA	256	18.150	71.762	-1.426	1.00	0.00	C
ATOM	3554	C	ALA	256	17.819	70.379	-1.952	1.00	0.00	C
ATOM	3555	O	ALA	256	18.626	69.774	-2.648	1.00	0.00	O
ATOM	3556	CB	ALA	256	18.602	71.681	0.042	1.00	0.00	C
ATOM	3557	H	ALA	256	16.645	73.069	-0.685	1.00	0.00	H
ATOM	3558	HA	ALA	256	18.975	72.158	-2.021	1.00	0.00	H
ATOM	3559	HB1	ALA	256	19.483	71.045	0.143	1.00	0.00	H
ATOM	3560	HB2	ALA	256	18.864	72.665	0.430	1.00	0.00	H
ATOM	3561	HB3	ALA	256	17.827	71.266	0.685	1.00	0.00	H
ATOM	3562	N	GLY	257	16.589	69.940	-1.704	1.00	0.00	N
ATOM	3563	CA	GLY	257	16.101	68.661	-2.142	1.00	0.00	C
ATOM	3564	C	GLY	257	15.865	68.627	-3.643	1.00	0.00	C
ATOM	3565	O	GLY	257	16.355	67.732	-4.332	1.00	0.00	O
ATOM	3566	H	GLY	257	15.993	70.509	-1.106	1.00	0.00	H
ATOM	3567	HA2	GLY	257	16.800	67.883	-1.832	1.00	0.00	H
ATOM	3568	HA3	GLY	257	15.142	68.483	-1.670	1.00	0.00	H
ATOM	3569	N	VAL	258	15.177	69.649	-4.159	1.00	0.00	N

ATOM	3570	CA	VAL	258	14.952	69.820	-5.581	1.00	0.00	C
ATOM	3571	C	VAL	258	16.239	69.901	-6.406	1.00	0.00	C
ATOM	3572	O	VAL	258	16.344	69.296	-7.476	1.00	0.00	O
ATOM	3573	CB	VAL	258	13.950	70.979	-5.834	1.00	0.00	C
ATOM	3574	CG1	VAL	258	13.908	71.494	-7.277	1.00	0.00	C
ATOM	3575	CG2	VAL	258	12.541	70.527	-5.437	1.00	0.00	C
ATOM	3576	H	VAL	258	14.787	70.349	-3.535	1.00	0.00	H
ATOM	3577	HA	VAL	258	14.477	68.898	-5.921	1.00	0.00	H
ATOM	3578	HB	VAL	258	14.234	71.829	-5.212	1.00	0.00	H
ATOM	3579	HG11	VAL	258	13.140	72.257	-7.403	1.00	0.00	H
ATOM	3580	HG12	VAL	258	14.862	71.944	-7.540	1.00	0.00	H
ATOM	3581	HG13	VAL	258	13.707	70.697	-7.992	1.00	0.00	H
ATOM	3582	HG21	VAL	258	11.769	71.236	-5.725	1.00	0.00	H
ATOM	3583	HG22	VAL	258	12.276	69.584	-5.912	1.00	0.00	H
ATOM	3584	HG23	VAL	258	12.481	70.365	-4.365	1.00	0.00	H
ATOM	3585	N	PHE	259	17.231	70.605	-5.867	1.00	0.00	N
ATOM	3586	CA	PHE	259	18.555	70.682	-6.440	1.00	0.00	C
ATOM	3587	C	PHE	259	19.269	69.340	-6.436	1.00	0.00	C
ATOM	3588	O	PHE	259	19.770	68.900	-7.473	1.00	0.00	O
ATOM	3589	CB	PHE	259	19.366	71.747	-5.665	1.00	0.00	C
ATOM	3590	CG	PHE	259	20.785	71.979	-6.159	1.00	0.00	C
ATOM	3591	CD1	PHE	259	21.015	72.730	-7.330	1.00	0.00	C
ATOM	3592	CD2	PHE	259	21.878	71.427	-5.456	1.00	0.00	C
ATOM	3593	CE1	PHE	259	22.329	72.924	-7.799	1.00	0.00	C
ATOM	3594	CE2	PHE	259	23.191	71.622	-5.926	1.00	0.00	C
ATOM	3595	CZ	PHE	259	23.417	72.369	-7.098	1.00	0.00	C
ATOM	3596	H	PHE	259	17.056	71.092	-4.992	1.00	0.00	H
ATOM	3597	HA	PHE	259	18.467	71.004	-7.478	1.00	0.00	H
ATOM	3598	HB2	PHE	259	18.843	72.703	-5.714	1.00	0.00	H
ATOM	3599	HB3	PHE	259	19.404	71.492	-4.605	1.00	0.00	H
ATOM	3600	HD1	PHE	259	20.186	73.153	-7.879	1.00	0.00	H
ATOM	3601	HD2	PHE	259	21.714	70.842	-4.563	1.00	0.00	H
ATOM	3602	HE1	PHE	259	22.503	73.493	-8.700	1.00	0.00	H
ATOM	3603	HE2	PHE	259	24.025	71.192	-5.390	1.00	0.00	H
ATOM	3604	HZ	PHE	259	24.424	72.513	-7.460	1.00	0.00	H
ATOM	3605	N	VAL	260	19.270	68.682	-5.275	1.00	0.00	N
ATOM	3606	CA	VAL	260	19.872	67.384	-5.081	1.00	0.00	C
ATOM	3607	C	VAL	260	19.392	66.311	-6.048	1.00	0.00	C
ATOM	3608	O	VAL	260	20.209	65.552	-6.569	1.00	0.00	O
ATOM	3609	CB	VAL	260	19.808	66.966	-3.578	1.00	0.00	C
ATOM	3610	CG1	VAL	260	19.934	65.454	-3.289	1.00	0.00	C
ATOM	3611	CG2	VAL	260	20.893	67.708	-2.771	1.00	0.00	C
ATOM	3612	H	VAL	260	18.833	69.110	-4.464	1.00	0.00	H
ATOM	3613	HA	VAL	260	20.928	67.510	-5.329	1.00	0.00	H
ATOM	3614	HB	VAL	260	18.832	67.259	-3.192	1.00	0.00	H
ATOM	3615	HG11	VAL	260	19.939	65.259	-2.216	1.00	0.00	H
ATOM	3616	HG12	VAL	260	19.098	64.889	-3.703	1.00	0.00	H
ATOM	3617	HG13	VAL	260	20.856	65.043	-3.703	1.00	0.00	H
ATOM	3618	HG21	VAL	260	20.738	67.586	-1.699	1.00	0.00	H
ATOM	3619	HG22	VAL	260	21.888	67.330	-3.009	1.00	0.00	H
ATOM	3620	HG23	VAL	260	20.902	68.777	-2.981	1.00	0.00	H
ATOM	3621	N	TYR	261	18.089	66.283	-6.318	1.00	0.00	N
ATOM	3622	CA	TYR	261	17.528	65.339	-7.260	1.00	0.00	C
ATOM	3623	C	TYR	261	18.032	65.557	-8.679	1.00	0.00	C
ATOM	3624	O	TYR	261	18.528	64.625	-9.312	1.00	0.00	O
ATOM	3625	CB	TYR	261	15.985	65.398	-7.224	1.00	0.00	C
ATOM	3626	CG	TYR	261	15.341	64.366	-8.135	1.00	0.00	C
ATOM	3627	CD1	TYR	261	15.341	63.007	-7.759	1.00	0.00	C
ATOM	3628	CD2	TYR	261	14.848	64.739	-9.405	1.00	0.00	C
ATOM	3629	CE1	TYR	261	14.891	62.028	-8.662	1.00	0.00	C
ATOM	3630	CE2	TYR	261	14.392	63.757	-10.304	1.00	0.00	C
ATOM	3631	CZ	TYR	261	14.430	62.400	-9.936	1.00	0.00	C
ATOM	3632	OH	TYR	261	14.032	61.432	-10.803	1.00	0.00	O

ATOM	3633	H	TYR	261	17.476	66.928	-5.826	1.00	0.00	H
ATOM	3634	HA	TYR	261	17.835	64.336	-6.951	1.00	0.00	H
ATOM	3635	HB2	TYR	261	15.629	65.225	-6.208	1.00	0.00	H
ATOM	3636	HB3	TYR	261	15.639	66.394	-7.499	1.00	0.00	H
ATOM	3637	HD1	TYR	261	15.726	62.699	-6.799	1.00	0.00	H
ATOM	3638	HD2	TYR	261	14.854	65.773	-9.717	1.00	0.00	H
ATOM	3639	HE1	TYR	261	14.917	60.983	-8.393	1.00	0.00	H
ATOM	3640	HE2	TYR	261	14.035	64.037	-11.286	1.00	0.00	H
ATOM	3641	HH	TYR	261	13.813	61.761	-11.700	1.00	0.00	H
ATOM	3642	N	GLN	262	17.942	66.802	-9.149	1.00	0.00	N
ATOM	3643	CA	GLN	262	18.321	67.177	-10.498	1.00	0.00	C
ATOM	3644	C	GLN	262	19.817	67.014	-10.773	1.00	0.00	C
ATOM	3645	O	GLN	262	20.217	66.843	-11.922	1.00	0.00	O
ATOM	3646	CB	GLN	262	17.854	68.639	-10.738	1.00	0.00	C
ATOM	3647	CG	GLN	262	18.183	69.263	-12.116	1.00	0.00	C
ATOM	3648	CD	GLN	262	17.546	68.511	-13.285	1.00	0.00	C
ATOM	3649	OE1	GLN	262	16.416	68.798	-13.673	1.00	0.00	O
ATOM	3650	NE2	GLN	262	18.271	67.563	-13.859	1.00	0.00	N
ATOM	3651	H	GLN	262	17.589	67.525	-8.534	1.00	0.00	H
ATOM	3652	HA	GLN	262	17.791	66.515	-11.186	1.00	0.00	H
ATOM	3653	HB2	GLN	262	16.775	68.692	-10.585	1.00	0.00	H
ATOM	3654	HB3	GLN	262	18.289	69.280	-9.968	1.00	0.00	H
ATOM	3655	HG2	GLN	262	17.816	70.289	-12.138	1.00	0.00	H
ATOM	3656	HG3	GLN	262	19.260	69.341	-12.268	1.00	0.00	H
ATOM	3657	HE22	GLN	262	17.909	66.980	-14.605	1.00	0.00	H
ATOM	3658	HE21	GLN	262	19.177	67.312	-13.463	1.00	0.00	H
ATOM	3659	N	LEU	263	20.622	67.057	-9.718	1.00	0.00	N
ATOM	3660	CA	LEU	263	22.041	66.832	-9.792	1.00	0.00	C
ATOM	3661	C	LEU	263	22.374	65.345	-9.705	1.00	0.00	C
ATOM	3662	O	LEU	263	22.963	64.792	-10.629	1.00	0.00	O
ATOM	3663	CB	LEU	263	22.689	67.598	-8.604	1.00	0.00	C
ATOM	3664	CG	LEU	263	24.226	67.485	-8.457	1.00	0.00	C
ATOM	3665	CD1	LEU	263	24.974	68.047	-9.676	1.00	0.00	C
ATOM	3666	CD2	LEU	263	24.691	68.183	-7.170	1.00	0.00	C
ATOM	3667	H	LEU	263	20.215	67.279	-8.818	1.00	0.00	H
ATOM	3668	HA	LEU	263	22.436	67.227	-10.730	1.00	0.00	H
ATOM	3669	HB2	LEU	263	22.429	68.654	-8.691	1.00	0.00	H
ATOM	3670	HB3	LEU	263	22.229	67.271	-7.670	1.00	0.00	H
ATOM	3671	HG	LEU	263	24.501	66.434	-8.354	1.00	0.00	H
ATOM	3672	HD11	LEU	263	26.054	67.992	-9.532	1.00	0.00	H
ATOM	3673	HD12	LEU	263	24.749	67.482	-10.581	1.00	0.00	H
ATOM	3674	HD13	LEU	263	24.717	69.091	-9.859	1.00	0.00	H
ATOM	3675	HD21	LEU	263	25.767	68.071	-7.030	1.00	0.00	H
ATOM	3676	HD22	LEU	263	24.466	69.249	-7.192	1.00	0.00	H
ATOM	3677	HD23	LEU	263	24.206	67.755	-6.292	1.00	0.00	H
ATOM	3678	N	MET	264	22.036	64.705	-8.582	1.00	0.00	N
ATOM	3679	CA	MET	264	22.609	63.415	-8.232	1.00	0.00	C
ATOM	3680	C	MET	264	21.916	62.274	-8.996	1.00	0.00	C
ATOM	3681	O	MET	264	22.568	61.286	-9.332	1.00	0.00	O
ATOM	3682	CB	MET	264	22.427	63.156	-6.720	1.00	0.00	C
ATOM	3683	CG	MET	264	23.151	64.167	-5.811	1.00	0.00	C
ATOM	3684	SD	MET	264	24.956	64.252	-5.985	1.00	0.00	S
ATOM	3685	CE	MET	264	25.446	62.611	-5.389	1.00	0.00	C
ATOM	3686	H	MET	264	21.467	65.182	-7.889	1.00	0.00	H
ATOM	3687	HA	MET	264	23.673	63.398	-8.481	1.00	0.00	H
ATOM	3688	HB2	MET	264	21.364	63.155	-6.472	1.00	0.00	H
ATOM	3689	HB3	MET	264	22.777	62.151	-6.481	1.00	0.00	H
ATOM	3690	HG2	MET	264	22.762	65.170	-5.988	1.00	0.00	H
ATOM	3691	HG3	MET	264	22.924	63.941	-4.769	1.00	0.00	H
ATOM	3692	HE1	MET	264	26.533	62.535	-5.360	1.00	0.00	H
ATOM	3693	HE2	MET	264	25.062	62.436	-4.383	1.00	0.00	H
ATOM	3694	HE3	MET	264	25.071	61.830	-6.050	1.00	0.00	H
ATOM	3695	N	ILE	265	20.616	62.412	-9.288	1.00	0.00	N

ATOM	3696	CA	ILE	265	19.833	61.420	-10.022	1.00	0.00	C
ATOM	3697	C	ILE	265	19.617	61.952	-11.448	1.00	0.00	C
ATOM	3698	O	ILE	265	20.018	61.318	-12.420	1.00	0.00	O
ATOM	3699	CB	ILE	265	18.471	61.165	-9.304	1.00	0.00	C
ATOM	3700	CG1	ILE	265	18.594	60.937	-7.777	1.00	0.00	C
ATOM	3701	CG2	ILE	265	17.679	60.014	-9.960	1.00	0.00	C
ATOM	3702	CD1	ILE	265	19.457	59.732	-7.368	1.00	0.00	C
ATOM	3703	H	ILE	265	20.163	63.285	-9.052	1.00	0.00	H
ATOM	3704	HA	ILE	265	20.369	60.471	-10.096	1.00	0.00	H
ATOM	3705	HB	ILE	265	17.851	62.056	-9.418	1.00	0.00	H
ATOM	3706	HG12	ILE	265	18.989	61.835	-7.300	1.00	0.00	H
ATOM	3707	HG13	ILE	265	17.596	60.809	-7.358	1.00	0.00	H
ATOM	3708	HG21	ILE	265	16.737	59.826	-9.447	1.00	0.00	H
ATOM	3709	HG22	ILE	265	17.427	60.239	-10.996	1.00	0.00	H
ATOM	3710	HG23	ILE	265	18.244	59.082	-9.965	1.00	0.00	H
ATOM	3711	HD11	ILE	265	19.498	59.638	-6.283	1.00	0.00	H
ATOM	3712	HD12	ILE	265	19.053	58.798	-7.760	1.00	0.00	H
ATOM	3713	HD13	ILE	265	20.482	59.828	-7.725	1.00	0.00	H
ATOM	3714	N	GLY	266	19.055	63.158	-11.592	1.00	0.00	N
ATOM	3715	CA	GLY	266	18.377	63.589	-12.813	1.00	0.00	C
ATOM	3716	C	GLY	266	19.288	64.282	-13.826	1.00	0.00	C
ATOM	3717	O	GLY	266	18.802	64.805	-14.826	1.00	0.00	O
ATOM	3718	H	GLY	266	18.824	63.666	-10.740	1.00	0.00	H
ATOM	3719	HA2	GLY	266	17.903	62.742	-13.305	1.00	0.00	H
ATOM	3720	HA3	GLY	266	17.573	64.273	-12.541	1.00	0.00	H
ATOM	3721	N	CYS	267	20.601	64.258	-13.587	1.00	0.00	N
ATOM	3722	CA	CYS	267	21.639	64.550	-14.573	1.00	0.00	C
ATOM	3723	C	CYS	267	22.079	63.232	-15.257	1.00	0.00	C
ATOM	3724	O	CYS	267	22.742	63.263	-16.289	1.00	0.00	O
ATOM	3725	CB	CYS	267	22.845	65.150	-13.812	1.00	0.00	C
ATOM	3726	SG	CYS	267	23.929	66.124	-14.897	1.00	0.00	S
ATOM	3727	H	CYS	267	20.902	63.861	-12.708	1.00	0.00	H
ATOM	3728	HA	CYS	267	21.274	65.254	-15.323	1.00	0.00	H
ATOM	3729	HB2	CYS	267	22.505	65.824	-13.027	1.00	0.00	H
ATOM	3730	HB3	CYS	267	23.433	64.372	-13.321	1.00	0.00	H
ATOM	3731	HG	CYS	267	24.988	66.085	-14.083	1.00	0.00	H
ATOM	3732	N	HIS	268	21.711	62.072	-14.688	1.00	0.00	N
ATOM	3733	CA	HIS	268	22.252	60.752	-15.012	1.00	0.00	C
ATOM	3734	C	HIS	268	21.162	59.788	-15.510	1.00	0.00	C
ATOM	3735	O	HIS	268	21.431	58.602	-15.692	1.00	0.00	O
ATOM	3736	CB	HIS	268	22.981	60.191	-13.767	1.00	0.00	C
ATOM	3737	CG	HIS	268	23.981	61.142	-13.144	1.00	0.00	C
ATOM	3738	ND1	HIS	268	23.968	61.504	-11.805	1.00	0.00	N
ATOM	3739	CD2	HIS	268	25.029	61.845	-13.699	1.00	0.00	C
ATOM	3740	CE1	HIS	268	24.947	62.392	-11.620	1.00	0.00	C
ATOM	3741	NE2	HIS	268	25.636	62.641	-12.726	1.00	0.00	N
ATOM	3742	H	HIS	268	21.126	62.102	-13.861	1.00	0.00	H
ATOM	3743	HA	HIS	268	22.983	60.833	-15.819	1.00	0.00	H
ATOM	3744	HB2	HIS	268	22.250	59.910	-13.007	1.00	0.00	H
ATOM	3745	HB3	HIS	268	23.506	59.272	-14.030	1.00	0.00	H
ATOM	3746	HD1	HIS	268	23.340	61.193	-11.062	1.00	0.00	H
ATOM	3747	HD2	HIS	268	25.372	61.861	-14.725	1.00	0.00	H
ATOM	3748	HE1	HIS	268	25.154	62.865	-10.670	1.00	0.00	H
ATOM	3749	N	LEU	269	19.939	60.281	-15.739	1.00	0.00	N
ATOM	3750	CA	LEU	269	18.819	59.517	-16.284	1.00	0.00	C
ATOM	3751	C	LEU	269	18.817	59.669	-17.810	1.00	0.00	C
ATOM	3752	O	LEU	269	18.632	60.738	-18.394	1.00	0.00	O
ATOM	3753	CB	LEU	269	17.490	60.095	-15.741	1.00	0.00	C
ATOM	3754	CG	LEU	269	17.277	59.934	-14.220	1.00	0.00	C
ATOM	3755	CD1	LEU	269	16.030	60.700	-13.758	1.00	0.00	C
ATOM	3756	CD2	LEU	269	17.190	58.462	-13.785	1.00	0.00	C
ATOM	3757	H	LEU	269	19.818	61.276	-15.636	1.00	0.00	H
ATOM	3758	HA	LEU	269	18.901	58.458	-16.035	1.00	0.00	H

```
ATOM   3759  HXT LEU   269      19.013  58.731 -18.331  1.00  0.00           H
ATOM   3760  HB2 LEU   269      17.423  61.153 -16.001  1.00  0.00           H
ATOM   3761  HB3 LEU   269      16.658  59.615 -16.257  1.00  0.00           H
ATOM   3762  HG  LEU   269      18.128  60.375 -13.710  1.00  0.00           H
ATOM   3763 HD11 LEU   269      15.943  60.715 -12.673  1.00  0.00           H
ATOM   3764 HD12 LEU   269      16.025  61.735 -14.099  1.00  0.00           H
ATOM   3765 HD13 LEU   269      15.120  60.244 -14.135  1.00  0.00           H
ATOM   3766 HD21 LEU   269      16.920  58.377 -12.731  1.00  0.00           H
ATOM   3767 HD22 LEU   269      16.437  57.921 -14.359  1.00  0.00           H
ATOM   3768 HD23 LEU   269      18.142  57.946 -13.912  1.00  0.00           H
ENDMDL
END



REMARK 888
REMARK 888 WRITTEN BY MAESTRO (A PRODUCT OF SCHRODINGER, LLC)
TITLE     hmod3
HELIX    1   1 LEU     21  VAL     47  1
HELIX    2   2 ILE     59  SER     78  1
HELIX    3   3 VAL     86  ALA     94  1
HELIX    4   4 LEU    101  ASN    133  1
HELIX    5   5 MET    157  VAL    179  1
HELIX    6   6 GLU    191  SER    210  1
HELIX    7   7 PHE    220  ALA    229  1
HELIX    8   8 ALA    234  THR    237  1
HELIX    9   9 VAL    245  HIE    268  1
TURN     1   1 ARG    20  ARG    20
TURN     2   2 LEU    48  THR    58
TURN     3   3 GLY    79  ALA    85
TURN     4   4 ARG    95  LYS   100
TURN     5   5 GLN   134  ASP   156
TURN     6   6 ASP   180  LEU   190
TURN     7   7 GLY   211  ASP   219
TURN     8   8 GLY   230  SER   233
TURN     9   9 THR   238  TRP   244
TURN    10  10 LEU   269  LEU   269
SEQRES   1    250  ARG LEU LEU ARG GLN ALA LEU ALA GLU CYS LEU GLY THR
SEQRES   2    250  LEU ILE LEU VAL MET PHE GLY CYS GLY SER VAL ALA GLN
SEQRES   3    250  VAL VAL LEU SER ARG GLY THR HIS GLY GLY PHE LEU THR
SEQRES   4    250  ILE ASN LEU ALA PHE GLY PHE ALA VAL THR LEU GLY ILE
SEQRES   5    250  LEU ILE ALA GLY GLN VAL SER GLY ALA HIS LEU ASN PRO
SEQRES   6    250  ALA VAL THR PHE ALA MET CYS PHE LEU ALA ARG GLU PRO
SEQRES   7    250  TRP ILE LYS LEU PRO ILE TYR THR LEU ALA GLN THR LEU
SEQRES   8    250  GLY ALA PHE LEU GLY ALA GLY ILE VAL PHE GLY LEU TYR
SEQRES   9    250  TYR ASP ALA ILE TRP HIS PHE ALA ASP ASN GLN LEU PHE
SEQRES  10    250  VAL SER GLY PRO ASN GLY THR ALA GLY ILE PHE ALA THR
SEQRES  11    250  TYR PRO SER GLY HIS LEU ASP MET ILE ASN GLY PHE PHE
SEQRES  12    250  ASP GLN PHE ILE GLY THR ALA SER LEU ILE VAL CYS VAL
SEQRES  13    250  LEU ALA ILE VAL ASP PRO TYR ASN ASN PRO VAL PRO ARG
SEQRES  14    250  GLY LEU GLU ALA PHE THR VAL GLY LEU VAL VAL LEU VAL
SEQRES  15    250  ILE GLY THR SER MET GLY PHE ASN SER GLY TYR ALA VAL
SEQRES  16    250  ASN PRO ALA ARG ASP PHE GLY PRO ARG LEU PHE THR ALA
SEQRES  17    250  LEU ALA GLY TRP GLY SER ALA VAL PHE THR THR GLY GLN
SEQRES  18    250  HIS TRP TRP TRP VAL PRO ILE VAL SER PRO LEU LEU GLY
SEQRES  19    250  SER ILE ALA GLY VAL PHE VAL TYR GLN LEU MET ILE GLY
SEQRES  20    250  CYS HIS LEU
MODEL        1
ATOM      1  N   ARG    20      -5.708  76.858 -14.736  1.00114.19       1SG N1+
ATOM      2  CA  ARG    20      -6.011  78.255 -15.110  1.00114.19       1SG C
ATOM      3  C   ARG    20      -6.340  79.005 -13.802  1.00114.19       1SG C
ATOM      4  O   ARG    20      -7.197  78.524 -13.074  1.00114.19       1SG O
```

```
ATOM      5  CB  ARG    20      -7.155  78.335 -16.157  1.00114.19          1SG C
ATOM      6  CG  ARG    20      -6.816  77.676 -17.519  1.00114.19          1SG C
ATOM      7  CD  ARG    20      -7.921  77.821 -18.590  1.00114.19          1SG C
ATOM      8  NE  ARG    20      -7.567  77.113 -19.840  1.00114.19          1SG N
ATOM      9  CZ  ARG    20      -8.402  76.722 -20.822  1.00114.19          1SG C
ATOM     10  NH1 ARG    20      -9.712  76.971 -20.753  1.00114.19          1SG N
ATOM     11  NH2 ARG    20      -7.914  76.075 -21.884  1.00114.19          1SG N1+
ATOM     12  H1  ARG    20      -4.842  76.925 -14.190  1.00  0.00              H
ATOM     13  H2  ARG    20      -6.416  76.509 -14.104  1.00  0.00              H
ATOM     14  H3  ARG    20      -5.543  76.231 -15.504  1.00  0.00              H
ATOM     15  HA  ARG    20      -5.105  78.684 -15.541  1.00  0.00              H
ATOM     16  HB2 ARG    20      -8.065  77.890 -15.748  1.00  0.00              H
ATOM     17  HB3 ARG    20      -7.396  79.386 -16.330  1.00  0.00              H
ATOM     18  HG2 ARG    20      -5.895  78.117 -17.904  1.00  0.00              H
ATOM     19  HG3 ARG    20      -6.610  76.614 -17.374  1.00  0.00              H
ATOM     20  HD2 ARG    20      -8.855  77.410 -18.203  1.00  0.00              H
ATOM     21  HD3 ARG    20      -8.103  78.873 -18.818  1.00  0.00              H
ATOM     22  HE  ARG    20      -6.572  76.999 -19.994  1.00  0.00              H
ATOM     23 HH12 ARG    20     -10.359  76.690 -21.482  1.00  0.00              H
ATOM     24 HH11 ARG    20     -10.118  77.496 -19.992  1.00  0.00              H
ATOM     25 HH22 ARG    20      -8.504  75.776 -22.653  1.00  0.00              H
ATOM     26 HH21 ARG    20      -6.934  75.846 -21.995  1.00  0.00              H
ATOM     27  N   LEU    21      -5.625  80.101 -13.497  1.00 38.86          1SG N
ATOM     28  CA  LEU    21      -5.388  80.778 -12.203  1.00 38.86          1SG C
ATOM     29  C   LEU    21      -6.343  80.613 -11.060  1.00 38.86          1SG C
ATOM     30  O   LEU    21      -6.040  79.913 -10.101  1.00 38.86          1SG O
ATOM     31  CB  LEU    21      -4.901  82.223 -12.464  1.00 38.86          1SG C
ATOM     32  CG  LEU    21      -4.321  82.990 -11.253  1.00 38.86          1SG C
ATOM     33  CD2 LEU    21      -4.010  84.438 -11.658  1.00 38.86          1SG C
ATOM     34  CD1 LEU    21      -3.065  82.315 -10.670  1.00 38.86          1SG C
ATOM     35  H   LEU    21      -4.960  80.401 -14.194  1.00  0.00              H
ATOM     36  HA  LEU    21      -4.499  80.251 -11.853  1.00  0.00              H
ATOM     37  HB2 LEU    21      -4.134  82.208 -13.240  1.00  0.00              H
ATOM     38  HB3 LEU    21      -5.727  82.801 -12.881  1.00  0.00              H
ATOM     39  HG  LEU    21      -5.070  83.038 -10.461  1.00  0.00              H
ATOM     40 HD21 LEU    21      -3.236  84.487 -12.425  1.00  0.00              H
ATOM     41 HD22 LEU    21      -4.895  84.948 -12.042  1.00  0.00              H
ATOM     42 HD23 LEU    21      -3.658  85.019 -10.807  1.00  0.00              H
ATOM     43 HD11 LEU    21      -2.649  82.905  -9.854  1.00  0.00              H
ATOM     44 HD12 LEU    21      -3.282  81.331 -10.253  1.00  0.00              H
ATOM     45 HD13 LEU    21      -2.280  82.202 -11.418  1.00  0.00              H
ATOM     46  N   LEU    22      -7.500  81.253 -11.175  1.00115.44          1SG N
ATOM     47  CA  LEU    22      -8.491  81.234 -10.120  1.00115.44          1SG C
ATOM     48  C   LEU    22      -8.973  79.822  -9.806  1.00115.44          1SG C
ATOM     49  O   LEU    22      -9.176  79.452  -8.652  1.00115.44          1SG O
ATOM     50  CB  LEU    22      -9.653  82.174 -10.536  1.00115.44          1SG C
ATOM     51  CG  LEU    22     -10.760  82.484  -9.494  1.00115.44          1SG C
ATOM     52  CD2 LEU    22     -10.216  82.830  -8.098  1.00115.44          1SG C
ATOM     53  CD1 LEU    22     -11.877  81.428  -9.425  1.00115.44          1SG C
ATOM     54  H   LEU    22      -7.647  81.868 -11.958  1.00  0.00              H
ATOM     55  HA  LEU    22      -8.019  81.631  -9.218  1.00  0.00              H
ATOM     56  HB2 LEU    22      -9.211  83.136 -10.803  1.00  0.00              H
ATOM     57  HB3 LEU    22     -10.121  81.811 -11.452  1.00  0.00              H
ATOM     58  HG  LEU    22     -11.252  83.390  -9.855  1.00  0.00              H
ATOM     59 HD21 LEU    22     -11.012  83.199  -7.450  1.00  0.00              H
ATOM     60 HD22 LEU    22      -9.779  81.963  -7.601  1.00  0.00              H
ATOM     61 HD23 LEU    22      -9.454  83.609  -8.146  1.00  0.00              H
ATOM     62 HD11 LEU    22     -12.718  81.801  -8.838  1.00  0.00              H
ATOM     63 HD12 LEU    22     -12.262  81.183 -10.415  1.00  0.00              H
ATOM     64 HD13 LEU    22     -11.553  80.505  -8.947  1.00  0.00              H
ATOM     65  N   ARG    23      -9.033  78.996 -10.851  1.00121.02          1SG N
ATOM     66  CA  ARG    23      -9.346  77.603 -10.705  1.00121.02          1SG C
ATOM     67  C   ARG    23      -8.214  76.732 -10.190  1.00121.02          1SG C
```

```
ATOM     68  O   ARG    23      -8.477  75.617  -9.737  1.00121.02        1SG O
ATOM     69  CB  ARG    23      -9.980  77.061 -12.015  1.00121.02        1SG C
ATOM     70  CG  ARG    23     -10.531  75.610 -11.999  1.00121.02        1SG C
ATOM     71  CD  ARG    23     -11.609  75.302 -10.932  1.00121.02        1SG C
ATOM     72  NE  ARG    23     -11.018  74.764  -9.693  1.00121.02        1SG N
ATOM     73  CZ  ARG    23     -11.607  74.035  -8.733  1.00121.02        1SG C
ATOM     74  NH1 ARG    23     -12.880  73.645  -8.843  1.00121.02        1SG N
ATOM     75  NH2 ARG    23     -10.907  73.701  -7.649  1.00121.02        1SG N1+
ATOM     76  H   ARG    23      -8.694  79.330 -11.740  1.00  0.00            H
ATOM     77  HA  ARG    23     -10.128  77.530  -9.945  1.00  0.00            H
ATOM     78  HB2 ARG    23     -10.803  77.722 -12.292  1.00  0.00            H
ATOM     79  HB3 ARG    23      -9.261  77.133 -12.827  1.00  0.00            H
ATOM     80  HG2 ARG    23     -10.965  75.407 -12.979  1.00  0.00            H
ATOM     81  HG3 ARG    23      -9.708  74.903 -11.919  1.00  0.00            H
ATOM     82  HD2 ARG    23     -12.221  76.178 -10.711  1.00  0.00            H
ATOM     83  HD3 ARG    23     -12.280  74.542 -11.334  1.00  0.00            H
ATOM     84  HE  ARG    23     -10.017  74.996  -9.591  1.00  0.00            H
ATOM     85 HH12 ARG    23     -13.335  73.085  -8.132  1.00  0.00            H
ATOM     86 HH11 ARG    23     -13.437  73.884  -9.649  1.00  0.00            H
ATOM     87 HH22 ARG    23     -11.292  73.161  -6.883  1.00  0.00            H
ATOM     88 HH21 ARG    23      -9.954  74.021  -7.504  1.00  0.00            H
ATOM     89  N   GLN    24      -6.993  77.252 -10.188  1.00 54.53        1SG N
ATOM     90  CA  GLN    24      -5.913  76.612  -9.482  1.00 54.53        1SG C
ATOM     91  C   GLN    24      -5.946  76.985  -8.022  1.00 54.53        1SG C
ATOM     92  O   GLN    24      -6.016  76.113  -7.168  1.00 54.53        1SG O
ATOM     93  CB  GLN    24      -4.532  77.037 -10.058  1.00 54.53        1SG C
ATOM     94  CG  GLN    24      -4.323  76.613 -11.527  1.00 54.53        1SG C
ATOM     95  CD  GLN    24      -3.320  77.455 -12.313  1.00 54.53        1SG C
ATOM     96  OE1 GLN    24      -3.522  77.681 -13.505  1.00 54.53        1SG O
ATOM     97  NE2 GLN    24      -2.275  77.970 -11.697  1.00 54.53        1SG N
ATOM     98  H   GLN    24      -6.878  78.233 -10.412  1.00  0.00            H
ATOM     99  HA  GLN    24      -5.980  75.522  -9.556  1.00  0.00            H
ATOM    100  HB2 GLN    24      -4.375  78.108  -9.945  1.00  0.00            H
ATOM    101  HB3 GLN    24      -3.743  76.577  -9.456  1.00  0.00            H
ATOM    102  HG2 GLN    24      -3.965  75.587 -11.557  1.00  0.00            H
ATOM    103  HG3 GLN    24      -5.275  76.617 -12.050  1.00  0.00            H
ATOM    104 HE22 GLN    24      -1.577  78.475 -12.209  1.00  0.00            H
ATOM    105 HE21 GLN    24      -1.975  77.517 -10.821  1.00  0.00            H
ATOM    106  N   ALA    25      -6.021  78.287  -7.749  1.00 31.81        1SG N
ATOM    107  CA  ALA    25      -6.068  78.822  -6.406  1.00 31.81        1SG C
ATOM    108  C   ALA    25      -7.135  78.195  -5.519  1.00 31.81        1SG C
ATOM    109  O   ALA    25      -6.906  77.867  -4.360  1.00 31.81        1SG O
ATOM    110  CB  ALA    25      -6.231  80.348  -6.476  1.00 31.81        1SG C
ATOM    111  H   ALA    25      -5.971  78.945  -8.518  1.00  0.00            H
ATOM    112  HA  ALA    25      -5.103  78.607  -5.938  1.00  0.00            H
ATOM    113  HB1 ALA    25      -6.198  80.786  -5.476  1.00  0.00            H
ATOM    114  HB2 ALA    25      -5.426  80.803  -7.052  1.00  0.00            H
ATOM    115  HB3 ALA    25      -7.180  80.634  -6.930  1.00  0.00            H
ATOM    116  N   LEU    26      -8.283  77.930  -6.133  1.00120.86        1SG N
ATOM    117  CA  LEU    26      -9.393  77.299  -5.471  1.00120.86        1SG C
ATOM    118  C   LEU    26      -9.248  75.804  -5.241  1.00120.86        1SG C
ATOM    119  O   LEU    26      -9.880  75.246  -4.348  1.00120.86        1SG O
ATOM    120  CB  LEU    26     -10.652  77.611  -6.332  1.00120.86        1SG C
ATOM    121  CG  LEU    26     -12.062  77.434  -5.704  1.00120.86        1SG C
ATOM    122  CD2 LEU    26     -12.154  77.858  -4.228  1.00120.86        1SG C
ATOM    123  CD1 LEU    26     -12.687  76.051  -5.942  1.00120.86        1SG C
ATOM    124  H   LEU    26      -8.408  78.286  -7.071  1.00  0.00            H
ATOM    125  HA  LEU    26      -9.505  77.773  -4.494  1.00  0.00            H
ATOM    126  HB2 LEU    26     -10.597  78.669  -6.593  1.00  0.00            H
ATOM    127  HB3 LEU    26     -10.599  77.085  -7.286  1.00  0.00            H
ATOM    128  HG  LEU    26     -12.707  78.127  -6.247  1.00  0.00            H
ATOM    129 HD21 LEU    26     -13.190  77.879  -3.889  1.00  0.00            H
ATOM    130 HD22 LEU    26     -11.620  77.171  -3.570  1.00  0.00            H
```

```
ATOM    131 HD23 LEU    26     -11.744  78.856  -4.072  1.00  0.00           H
ATOM    132 HD11 LEU    26     -13.722  76.035  -5.597  1.00  0.00           H
ATOM    133 HD12 LEU    26     -12.696  75.803  -7.001  1.00  0.00           H
ATOM    134 HD13 LEU    26     -12.164  75.262  -5.405  1.00  0.00           H
ATOM    135  N   ALA    27      -8.400  75.146  -6.024  1.00 29.74       1SG N
ATOM    136  CA  ALA    27      -8.039  73.778  -5.741  1.00 29.74       1SG C
ATOM    137  C   ALA    27      -6.949  73.674  -4.713  1.00 29.74       1SG C
ATOM    138  O   ALA    27      -7.030  72.786  -3.869  1.00 29.74       1SG O
ATOM    139  CB  ALA    27      -7.539  73.122  -7.040  1.00 29.74       1SG C
ATOM    140  H   ALA    27      -7.735  75.694  -6.566  1.00  0.00           H
ATOM    141  HA  ALA    27      -8.896  73.205  -5.380  1.00  0.00           H
ATOM    142  HB1 ALA    27      -7.109  72.137  -6.845  1.00  0.00           H
ATOM    143  HB2 ALA    27      -8.330  72.979  -7.771  1.00  0.00           H
ATOM    144  HB3 ALA    27      -6.769  73.725  -7.514  1.00  0.00           H
ATOM    145  N   GLU    28      -6.005  74.616  -4.730  1.00 95.46       1SG N
ATOM    146  CA  GLU    28      -5.041  74.706  -3.665  1.00 95.46       1SG C
ATOM    147  C   GLU    28      -5.657  74.961  -2.305  1.00 95.46       1SG C
ATOM    148  O   GLU    28      -5.304  74.313  -1.321  1.00 95.46       1SG O
ATOM    149  CB  GLU    28      -3.934  75.752  -3.924  1.00 95.46       1SG C
ATOM    150  CG  GLU    28      -2.887  75.455  -5.023  1.00 95.46       1SG C
ATOM    151  CD  GLU    28      -2.184  74.088  -4.969  1.00 95.46       1SG C
ATOM    152  OE1 GLU    28      -1.756  73.664  -3.877  1.00 95.46       1SG O
ATOM    153  OE2 GLU    28      -2.075  73.451  -6.043  1.00 95.46       1SG O1-
ATOM    154  H   GLU    28      -5.896  75.221  -5.538  1.00  0.00           H
ATOM    155  HA  GLU    28      -4.554  73.731  -3.585  1.00  0.00           H
ATOM    156  HB2 GLU    28      -4.375  76.732  -4.102  1.00  0.00           H
ATOM    157  HB3 GLU    28      -3.371  75.850  -3.000  1.00  0.00           H
ATOM    158  HG2 GLU    28      -3.322  75.627  -6.002  1.00  0.00           H
ATOM    159  HG3 GLU    28      -2.102  76.202  -4.925  1.00  0.00           H
ATOM    160  N   CYS    29      -6.640  75.853  -2.292  1.00 37.73       1SG N
ATOM    161  CA  CYS    29      -7.398  76.166  -1.110  1.00 37.73       1SG C
ATOM    162  C   CYS    29      -8.134  74.981  -0.525  1.00 37.73       1SG C
ATOM    163  O   CYS    29      -8.093  74.761   0.680  1.00 37.73       1SG O
ATOM    164  CB  CYS    29      -8.374  77.318  -1.437  1.00 37.73       1SG C
ATOM    165  SG  CYS    29      -9.111  78.050   0.053  1.00 37.73       1SG S
ATOM    166  H   CYS    29      -6.795  76.401  -3.131  1.00  0.00           H
ATOM    167  HA  CYS    29      -6.690  76.519  -0.357  1.00  0.00           H
ATOM    168  HB2 CYS    29      -7.854  78.125  -1.952  1.00  0.00           H
ATOM    169  HB3 CYS    29      -9.174  76.979  -2.097  1.00  0.00           H
ATOM    170  HG  CYS    29      -9.438  76.897   0.650  1.00  0.00           H
ATOM    171  N   LEU    30      -8.760  74.198  -1.398  1.00133.90       1SG N
ATOM    172  CA  LEU    30      -9.447  72.997  -0.996  1.00133.90       1SG C
ATOM    173  C   LEU    30      -8.485  71.917  -0.577  1.00133.90       1SG C
ATOM    174  O   LEU    30      -8.694  71.285   0.446  1.00133.90       1SG O
ATOM    175  CB  LEU    30     -10.306  72.515  -2.195  1.00133.90       1SG C
ATOM    176  CG  LEU    30     -11.177  71.257  -1.955  1.00133.90       1SG C
ATOM    177  CD2 LEU    30     -11.813  70.779  -3.270  1.00133.90       1SG C
ATOM    178  CD1 LEU    30     -12.260  71.488  -0.888  1.00133.90       1SG C
ATOM    179  H   LEU    30      -8.717  74.427  -2.379  1.00  0.00           H
ATOM    180  HA  LEU    30     -10.099  73.232  -0.152  1.00  0.00           H
ATOM    181  HB2 LEU    30     -10.958  73.331  -2.510  1.00  0.00           H
ATOM    182  HB3 LEU    30      -9.646  72.322  -3.042  1.00  0.00           H
ATOM    183  HG  LEU    30     -10.541  70.442  -1.606  1.00  0.00           H
ATOM    184 HD21 LEU    30     -12.387  69.864  -3.115  1.00  0.00           H
ATOM    185 HD22 LEU    30     -12.488  71.530  -3.682  1.00  0.00           H
ATOM    186 HD23 LEU    30     -11.051  70.558  -4.018  1.00  0.00           H
ATOM    187 HD11 LEU    30     -12.866  70.593  -0.744  1.00  0.00           H
ATOM    188 HD12 LEU    30     -11.825  71.728   0.083  1.00  0.00           H
ATOM    189 HD13 LEU    30     -12.931  72.303  -1.162  1.00  0.00           H
ATOM    190  N   GLY    31      -7.407  71.753  -1.330  1.00 33.20       1SG N
ATOM    191  CA  GLY    31      -6.493  70.667  -1.124  1.00 33.20       1SG C
ATOM    192  C   GLY    31      -5.682  70.800   0.140  1.00 33.20       1SG C
ATOM    193  O   GLY    31      -5.495  69.823   0.863  1.00 33.20       1SG O
```

```
ATOM    194  H   GLY    31      -7.282  72.313  -2.170  1.00  0.00           H
ATOM    195  HA2 GLY    31      -7.004  69.707  -1.172  1.00  0.00           H
ATOM    196  HA3 GLY    31      -5.800  70.657  -1.965  1.00  0.00           H
ATOM    197  N   THR    32      -5.285  72.035   0.441  1.00 38.14       1SG N
ATOM    198  CA  THR    32      -4.541  72.309   1.645  1.00 38.14       1SG C
ATOM    199  C   THR    32      -5.429  72.331   2.874  1.00 38.14       1SG C
ATOM    200  O   THR    32      -4.997  71.962   3.966  1.00 38.14       1SG O
ATOM    201  CB  THR    32      -3.816  73.679   1.545  1.00 38.14       1SG C
ATOM    202  OG1 THR    32      -3.183  73.828   0.290  1.00 38.14       1SG O
ATOM    203  CG2 THR    32      -2.693  73.813   2.586  1.00 38.14       1SG C
ATOM    204  H   THR    32      -5.474  72.795  -0.202  1.00  0.00           H
ATOM    205  HA  THR    32      -3.785  71.531   1.773  1.00  0.00           H
ATOM    206  HB  THR    32      -4.522  74.505   1.659  1.00  0.00           H
ATOM    207  HG1 THR    32      -3.859  73.912  -0.380  1.00  0.00           H
ATOM    208 HG21 THR    32      -2.091  74.704   2.420  1.00  0.00           H
ATOM    209 HG22 THR    32      -3.092  73.867   3.598  1.00  0.00           H
ATOM    210 HG23 THR    32      -2.019  72.958   2.551  1.00  0.00           H
ATOM    211  N   LEU    33      -6.689  72.712   2.674  1.00 91.34       1SG N
ATOM    212  CA  LEU    33      -7.685  72.595   3.699  1.00 91.34       1SG C
ATOM    213  C   LEU    33      -7.975  71.155   4.016  1.00 91.34       1SG C
ATOM    214  O   LEU    33      -7.936  70.769   5.182  1.00 91.34       1SG O
ATOM    215  CB  LEU    33      -8.981  73.325   3.252  1.00 91.34       1SG C
ATOM    216  CG  LEU    33     -10.171  73.324   4.247  1.00 91.34       1SG C
ATOM    217  CD2 LEU    33     -11.152  72.154   4.039  1.00 91.34       1SG C
ATOM    218  CD1 LEU    33     -10.942  74.649   4.175  1.00 91.34       1SG C
ATOM    219  H   LEU    33      -6.990  72.976   1.748  1.00  0.00           H
ATOM    220  HA  LEU    33      -7.315  73.070   4.610  1.00  0.00           H
ATOM    221  HB2 LEU    33      -8.700  74.362   3.080  1.00  0.00           H
ATOM    222  HB3 LEU    33      -9.325  72.958   2.287  1.00  0.00           H
ATOM    223  HG  LEU    33      -9.768  73.250   5.255  1.00  0.00           H
ATOM    224 HD21 LEU    33     -12.003  72.233   4.717  1.00  0.00           H
ATOM    225 HD22 LEU    33     -11.543  72.132   3.022  1.00  0.00           H
ATOM    226 HD23 LEU    33     -10.697  71.186   4.241  1.00  0.00           H
ATOM    227 HD11 LEU    33     -11.736  74.684   4.922  1.00  0.00           H
ATOM    228 HD12 LEU    33     -10.281  75.492   4.369  1.00  0.00           H
ATOM    229 HD13 LEU    33     -11.395  74.798   3.195  1.00  0.00           H
ATOM    230  N   ILE    34      -8.250  70.361   2.981  1.00 98.20       1SG N
ATOM    231  CA  ILE    34      -8.630  68.999   3.222  1.00 98.20       1SG C
ATOM    232  C   ILE    34      -7.529  68.108   3.783  1.00 98.20       1SG C
ATOM    233  O   ILE    34      -7.786  67.211   4.582  1.00 98.20       1SG O
ATOM    234  CB  ILE    34      -9.405  68.315   2.060  1.00 98.20       1SG C
ATOM    235  CG2 ILE    34     -10.737  69.045   1.775  1.00 98.20       1SG C
ATOM    236  CG1 ILE    34      -8.567  68.007   0.802  1.00 98.20       1SG C
ATOM    237  CD1 ILE    34      -9.356  67.366  -0.352  1.00 98.20       1SG C
ATOM    238  H   ILE    34      -8.319  70.733   2.036  1.00  0.00           H
ATOM    239  HA  ILE    34      -9.362  69.030   4.034  1.00  0.00           H
ATOM    240  HB  ILE    34      -9.705  67.337   2.446  1.00  0.00           H
ATOM    241 HG21 ILE    34     -11.383  68.468   1.114  1.00  0.00           H
ATOM    242 HG22 ILE    34     -11.300  69.195   2.697  1.00  0.00           H
ATOM    243 HG23 ILE    34     -10.591  70.023   1.327  1.00  0.00           H
ATOM    244 HG12 ILE    34      -8.092  68.915   0.461  1.00  0.00           H
ATOM    245 HG13 ILE    34      -7.751  67.335   1.071  1.00  0.00           H
ATOM    246 HD11 ILE    34      -8.703  67.109  -1.185  1.00  0.00           H
ATOM    247 HD12 ILE    34      -9.851  66.449  -0.030  1.00  0.00           H
ATOM    248 HD13 ILE    34     -10.114  68.042  -0.747  1.00  0.00           H
ATOM    249  N   LEU    35      -6.295  68.456   3.451  1.00103.34       1SG N
ATOM    250  CA  LEU    35      -5.110  67.971   4.103  1.00103.34       1SG C
ATOM    251  C   LEU    35      -5.091  68.246   5.601  1.00103.34       1SG C
ATOM    252  O   LEU    35      -4.884  67.332   6.402  1.00103.34       1SG O
ATOM    253  CB  LEU    35      -3.916  68.630   3.355  1.00103.34       1SG C
ATOM    254  CG  LEU    35      -2.465  68.444   3.860  1.00103.34       1SG C
ATOM    255  CD2 LEU    35      -2.077  69.356   5.042  1.00103.34       1SG C
ATOM    256  CD1 LEU    35      -1.498  68.730   2.697  1.00103.34       1SG C
```

```
ATOM    257  H   LEU    35      -6.186  69.173   2.744  1.00  0.00           H
ATOM    258  HA  LEU    35      -5.057  66.889   3.961  1.00  0.00           H
ATOM    259  HB2 LEU    35      -3.959  68.244   2.335  1.00  0.00           H
ATOM    260  HB3 LEU    35      -4.094  69.698   3.255  1.00  0.00           H
ATOM    261  HG  LEU    35      -2.341  67.406   4.167  1.00  0.00           H
ATOM    262 HD21 LEU    35      -1.003  69.326   5.224  1.00  0.00           H
ATOM    263 HD22 LEU    35      -2.350  70.395   4.853  1.00  0.00           H
ATOM    264 HD23 LEU    35      -2.545  69.049   5.975  1.00  0.00           H
ATOM    265 HD11 LEU    35      -0.455  68.646   3.002  1.00  0.00           H
ATOM    266 HD12 LEU    35      -1.651  68.033   1.872  1.00  0.00           H
ATOM    267 HD13 LEU    35      -1.640  69.739   2.309  1.00  0.00           H
ATOM    268  N   VAL    36      -5.303  69.514   5.974  1.00102.65       1SG N
ATOM    269  CA  VAL    36      -5.263  69.933   7.363  1.00102.65       1SG C
ATOM    270  C   VAL    36      -6.379  69.345   8.173  1.00102.65       1SG C
ATOM    271  O   VAL    36      -6.142  68.918   9.297  1.00102.65       1SG O
ATOM    272  CB  VAL    36      -5.121  71.488   7.475  1.00102.65       1SG C
ATOM    273  CG1 VAL    36      -6.399  72.264   7.838  1.00102.65       1SG C
ATOM    274  CG2 VAL    36      -4.000  71.877   8.455  1.00102.65       1SG C
ATOM    275  H   VAL    36      -5.496  70.212   5.266  1.00  0.00           H
ATOM    276  HA  VAL    36      -4.346  69.498   7.766  1.00  0.00           H
ATOM    277  HB  VAL    36      -4.798  71.867   6.506  1.00  0.00           H
ATOM    278 HG11 VAL    36      -6.230  73.337   7.834  1.00  0.00           H
ATOM    279 HG12 VAL    36      -7.196  72.061   7.131  1.00  0.00           H
ATOM    280 HG13 VAL    36      -6.761  72.013   8.836  1.00  0.00           H
ATOM    281 HG21 VAL    36      -3.853  72.957   8.471  1.00  0.00           H
ATOM    282 HG22 VAL    36      -4.235  71.560   9.473  1.00  0.00           H
ATOM    283 HG23 VAL    36      -3.048  71.427   8.174  1.00  0.00           H
ATOM    284  N   MET    37      -7.549  69.256   7.556  1.00 91.63       1SG N
ATOM    285  CA  MET    37      -8.745  68.723   8.146  1.00 91.63       1SG C
ATOM    286  C   MET    37      -8.579  67.345   8.752  1.00 91.63       1SG C
ATOM    287  O   MET    37      -9.062  67.076   9.849  1.00 91.63       1SG O
ATOM    288  CB  MET    37      -9.884  68.768   7.105  1.00 91.63       1SG C
ATOM    289  CG  MET    37     -11.287  68.592   7.701  1.00 91.63       1SG C
ATOM    290  SD  MET    37     -12.614  68.946   6.516  1.00 91.63       1SG S
ATOM    291  CE  MET    37     -14.048  68.645   7.586  1.00 91.63       1SG C
ATOM    292  H   MET    37      -7.620  69.658   6.624  1.00  0.00           H
ATOM    293  HA  MET    37      -9.002  69.408   8.953  1.00  0.00           H
ATOM    294  HB2 MET    37      -9.868  69.738   6.608  1.00  0.00           H
ATOM    295  HB3 MET    37      -9.722  68.022   6.325  1.00  0.00           H
ATOM    296  HG2 MET    37     -11.414  67.576   8.078  1.00  0.00           H
ATOM    297  HG3 MET    37     -11.415  69.261   8.552  1.00  0.00           H
ATOM    298  HE1 MET    37     -14.974  68.823   7.039  1.00  0.00           H
ATOM    299  HE2 MET    37     -14.052  67.613   7.940  1.00  0.00           H
ATOM    300  HE3 MET    37     -14.029  69.306   8.453  1.00  0.00           H
ATOM    301  N   PHE    38      -7.812  66.516   8.057  1.00100.98       1SG N
ATOM    302  CA  PHE    38      -7.549  65.179   8.509  1.00100.98       1SG C
ATOM    303  C   PHE    38      -6.303  65.008   9.353  1.00100.98       1SG C
ATOM    304  O   PHE    38      -6.156  64.004  10.054  1.00100.98       1SG O
ATOM    305  CB  PHE    38      -7.497  64.249   7.284  1.00100.98       1SG C
ATOM    306  CG  PHE    38      -8.836  64.006   6.602  1.00100.98       1SG C
ATOM    307  CD1 PHE    38      -9.915  63.444   7.320  1.00100.98       1SG C
ATOM    308  CD2 PHE    38      -9.007  64.330   5.239  1.00100.98       1SG C
ATOM    309  CE1 PHE    38     -11.156  63.235   6.688  1.00100.98       1SG C
ATOM    310  CE2 PHE    38     -10.249  64.125   4.608  1.00100.98       1SG C
ATOM    311  CZ  PHE    38     -11.325  63.579   5.333  1.00100.98       1SG C
ATOM    312  H   PHE    38      -7.475  66.801   7.145  1.00  0.00           H
ATOM    313  HA  PHE    38      -8.364  64.826   9.143  1.00  0.00           H
ATOM    314  HB2 PHE    38      -6.822  64.687   6.557  1.00  0.00           H
ATOM    315  HB3 PHE    38      -7.088  63.273   7.552  1.00  0.00           H
ATOM    316  HD1 PHE    38      -9.799  63.170   8.358  1.00  0.00           H
ATOM    317  HD2 PHE    38      -8.187  64.741   4.667  1.00  0.00           H
ATOM    318  HE1 PHE    38     -11.978  62.807   7.243  1.00  0.00           H
ATOM    319  HE2 PHE    38     -10.372  64.386   3.567  1.00  0.00           H
```

```
ATOM    320  HZ  PHE    38     -12.277  63.418   4.849  1.00  0.00         H
ATOM    321  N   GLY    39      -5.465  66.033   9.390  1.00 44.23     1SG N
ATOM    322  CA  GLY    39      -4.256  65.990  10.164  1.00 44.23     1SG C
ATOM    323  C   GLY    39      -4.467  66.639  11.513  1.00 44.23     1SG C
ATOM    324  O   GLY    39      -4.188  66.035  12.546  1.00 44.23     1SG O
ATOM    325  H   GLY    39      -5.662  66.843   8.815  1.00  0.00         H
ATOM    326  HA2 GLY    39      -3.876  64.975  10.290  1.00  0.00         H
ATOM    327  HA3 GLY    39      -3.478  66.537   9.631  1.00  0.00         H
ATOM    328  N   CYS    40      -5.081  67.824  11.530  1.00 93.03     1SG N
ATOM    329  CA  CYS    40      -5.503  68.419  12.784  1.00 93.03     1SG C
ATOM    330  C   CYS    40      -6.720  67.775  13.406  1.00 93.03     1SG C
ATOM    331  O   CYS    40      -6.986  67.974  14.585  1.00 93.03     1SG O
ATOM    332  CB  CYS    40      -5.635  69.962  12.705  1.00 93.03     1SG C
ATOM    333  SG  CYS    40      -6.908  70.623  11.595  1.00 93.03     1SG S
ATOM    334  H   CYS    40      -5.411  68.240  10.660  1.00  0.00         H
ATOM    335  HA  CYS    40      -4.690  68.277  13.491  1.00  0.00         H
ATOM    336  HB2 CYS    40      -5.855  70.353  13.700  1.00  0.00         H
ATOM    337  HB3 CYS    40      -4.676  70.395  12.424  1.00  0.00         H
ATOM    338  HG  CYS    40      -6.571  69.878  10.527  1.00  0.00         H
ATOM    339  N   GLY    41      -7.410  66.936  12.643  1.00 32.38     1SG N
ATOM    340  CA  GLY    41      -8.628  66.342  13.110  1.00 32.38     1SG C
ATOM    341  C   GLY    41      -8.445  64.953  13.687  1.00 32.38     1SG C
ATOM    342  O   GLY    41      -9.269  64.518  14.490  1.00 32.38     1SG O
ATOM    343  H   GLY    41      -7.175  66.851  11.666  1.00  0.00         H
ATOM    344  HA2 GLY    41      -9.137  66.968  13.844  1.00  0.00         H
ATOM    345  HA3 GLY    41      -9.318  66.260  12.272  1.00  0.00         H
ATOM    346  N   SER    42      -7.333  64.295  13.357  1.00 94.53     1SG N
ATOM    347  CA  SER    42      -6.872  63.122  14.074  1.00 94.53     1SG C
ATOM    348  C   SER    42      -6.165  63.512  15.355  1.00 94.53     1SG C
ATOM    349  O   SER    42      -6.401  62.928  16.412  1.00 94.53     1SG O
ATOM    350  CB  SER    42      -5.960  62.291  13.141  1.00 94.53     1SG C
ATOM    351  OG  SER    42      -4.992  63.072  12.457  1.00 94.53     1SG O
ATOM    352  H   SER    42      -6.678  64.717  12.715  1.00  0.00         H
ATOM    353  HA  SER    42      -7.720  62.491  14.353  1.00  0.00         H
ATOM    354  HB2 SER    42      -5.449  61.515  13.714  1.00  0.00         H
ATOM    355  HB3 SER    42      -6.564  61.768  12.398  1.00  0.00         H
ATOM    356  HG  SER    42      -5.356  63.336  11.610  1.00  0.00         H
ATOM    357  N   VAL    43      -5.365  64.572  15.283  1.00115.26     1SG N
ATOM    358  CA  VAL    43      -4.588  64.995  16.418  1.00115.26     1SG C
ATOM    359  C   VAL    43      -5.342  65.925  17.371  1.00115.26     1SG C
ATOM    360  O   VAL    43      -4.824  66.292  18.419  1.00115.26     1SG O
ATOM    361  CB  VAL    43      -3.245  65.594  15.883  1.00115.26     1SG C
ATOM    362  CG1 VAL    43      -3.249  67.104  15.600  1.00115.26     1SG C
ATOM    363  CG2 VAL    43      -2.046  65.235  16.778  1.00115.26     1SG C
ATOM    364  H   VAL    43      -5.154  64.972  14.375  1.00  0.00         H
ATOM    365  HA  VAL    43      -4.320  64.104  16.983  1.00  0.00         H
ATOM    366  HB  VAL    43      -3.035  65.110  14.927  1.00  0.00         H
ATOM    367 HG11 VAL    43      -2.595  67.353  14.765  1.00  0.00         H
ATOM    368 HG12 VAL    43      -4.247  67.467  15.372  1.00  0.00         H
ATOM    369 HG13 VAL    43      -2.892  67.662  16.463  1.00  0.00         H
ATOM    370 HG21 VAL    43      -1.118  65.640  16.372  1.00  0.00         H
ATOM    371 HG22 VAL    43      -2.168  65.634  17.786  1.00  0.00         H
ATOM    372 HG23 VAL    43      -1.919  64.155  16.857  1.00  0.00         H
ATOM    373  N   ALA    44      -6.598  66.222  17.050  1.00 50.64     1SG N
ATOM    374  CA  ALA    44      -7.576  66.679  18.009  1.00 50.64     1SG C
ATOM    375  C   ALA    44      -8.110  65.517  18.791  1.00 50.64     1SG C
ATOM    376  O   ALA    44      -8.184  65.571  20.007  1.00 50.64     1SG O
ATOM    377  CB  ALA    44      -8.769  67.266  17.229  1.00 50.64     1SG C
ATOM    378  H   ALA    44      -6.908  65.985  16.121  1.00  0.00         H
ATOM    379  HA  ALA    44      -7.154  67.435  18.672  1.00  0.00         H
ATOM    380  HB1 ALA    44      -9.542  67.635  17.905  1.00  0.00         H
ATOM    381  HB2 ALA    44      -8.467  68.100  16.603  1.00  0.00         H
ATOM    382  HB3 ALA    44      -9.237  66.535  16.570  1.00  0.00         H
```

```
ATOM    383  N   GLN    45      -8.454  64.444  18.090  1.00 88.18        1SG N
ATOM    384  CA  GLN    45      -9.072  63.301  18.702  1.00 88.18        1SG C
ATOM    385  C   GLN    45      -8.166  62.572  19.661  1.00 88.18        1SG C
ATOM    386  O   GLN    45      -8.607  62.113  20.715  1.00 88.18        1SG O
ATOM    387  CB  GLN    45      -9.601  62.376  17.584  1.00 88.18        1SG C
ATOM    388  CG  GLN    45     -10.481  61.208  18.086  1.00 88.18        1SG C
ATOM    389  CD  GLN    45     -11.452  60.612  17.063  1.00 88.18        1SG C
ATOM    390  OE1 GLN    45     -11.782  59.437  17.152  1.00 88.18        1SG O
ATOM    391  NE2 GLN    45     -11.930  61.367  16.080  1.00 88.18        1SG N
ATOM    392  H   GLN    45      -8.321  64.438  17.091  1.00  0.00            H
ATOM    393  HA  GLN    45      -9.932  63.660  19.271  1.00  0.00            H
ATOM    394  HB2 GLN    45     -10.164  63.000  16.897  1.00  0.00            H
ATOM    395  HB3 GLN    45      -8.777  61.963  17.001  1.00  0.00            H
ATOM    396  HG2 GLN    45      -9.862  60.411  18.497  1.00  0.00            H
ATOM    397  HG3 GLN    45     -11.107  61.557  18.906  1.00  0.00            H
ATOM    398 HE22 GLN    45     -12.584  60.938  15.448  1.00  0.00            H
ATOM    399 HE21 GLN    45     -11.769  62.370  16.036  1.00  0.00            H
ATOM    400  N   VAL    46      -6.876  62.576  19.336  1.00 98.75        1SG N
ATOM    401  CA  VAL    46      -5.894  62.120  20.270  1.00 98.75        1SG C
ATOM    402  C   VAL    46      -5.821  62.933  21.555  1.00 98.75        1SG C
ATOM    403  O   VAL    46      -5.710  62.363  22.641  1.00 98.75        1SG O
ATOM    404  CB  VAL    46      -4.499  61.920  19.597  1.00 98.75        1SG C
ATOM    405  CG1 VAL    46      -3.638  63.186  19.477  1.00 98.75        1SG C
ATOM    406  CG2 VAL    46      -3.665  60.844  20.319  1.00 98.75        1SG C
ATOM    407  H   VAL    46      -6.600  62.882  18.409  1.00  0.00            H
ATOM    408  HA  VAL    46      -6.226  61.123  20.570  1.00  0.00            H
ATOM    409  HB  VAL    46      -4.683  61.547  18.588  1.00  0.00            H
ATOM    410 HG11 VAL    46      -2.830  63.046  18.760  1.00  0.00            H
ATOM    411 HG12 VAL    46      -4.234  64.032  19.163  1.00  0.00            H
ATOM    412 HG13 VAL    46      -3.183  63.458  20.430  1.00  0.00            H
ATOM    413 HG21 VAL    46      -2.723  60.661  19.802  1.00  0.00            H
ATOM    414 HG22 VAL    46      -3.429  61.140  21.342  1.00  0.00            H
ATOM    415 HG23 VAL    46      -4.200  59.899  20.374  1.00  0.00            H
ATOM    416  N   VAL    47      -5.906  64.256  21.416  1.00131.72        1SG N
ATOM    417  CA  VAL    47      -5.542  65.142  22.489  1.00131.72        1SG C
ATOM    418  C   VAL    47      -6.754  65.719  23.232  1.00131.72        1SG C
ATOM    419  O   VAL    47      -6.595  66.213  24.340  1.00131.72        1SG O
ATOM    420  CB  VAL    47      -4.476  66.176  22.008  1.00131.72        1SG C
ATOM    421  CG1 VAL    47      -5.024  67.393  21.255  1.00131.72        1SG C
ATOM    422  CG2 VAL    47      -3.555  66.649  23.148  1.00131.72        1SG C
ATOM    423  H   VAL    47      -6.072  64.651  20.503  1.00  0.00            H
ATOM    424  HA  VAL    47      -5.028  64.567  23.263  1.00  0.00            H
ATOM    425  HB  VAL    47      -3.825  65.652  21.306  1.00  0.00            H
ATOM    426 HG11 VAL    47      -5.424  68.136  21.941  1.00  0.00            H
ATOM    427 HG12 VAL    47      -4.239  67.880  20.677  1.00  0.00            H
ATOM    428 HG13 VAL    47      -5.808  67.099  20.563  1.00  0.00            H
ATOM    429 HG21 VAL    47      -2.774  67.312  22.774  1.00  0.00            H
ATOM    430 HG22 VAL    47      -4.111  67.193  23.913  1.00  0.00            H
ATOM    431 HG23 VAL    47      -3.061  65.808  23.635  1.00  0.00            H
ATOM    432  N   LEU    48      -7.958  65.622  22.663  1.00149.95        1SG N
ATOM    433  CA  LEU    48      -9.194  66.100  23.259  1.00149.95        1SG C
ATOM    434  C   LEU    48     -10.090  64.938  23.694  1.00149.95        1SG C
ATOM    435  O   LEU    48     -10.526  64.875  24.846  1.00149.95        1SG O
ATOM    436  CB  LEU    48     -10.014  66.930  22.236  1.00149.95        1SG C
ATOM    437  CG  LEU    48      -9.598  68.397  21.985  1.00149.95        1SG C
ATOM    438  CD2 LEU    48     -10.420  68.951  20.819  1.00149.95        1SG C
ATOM    439  CD1 LEU    48      -8.113  68.622  21.718  1.00149.95        1SG C
ATOM    440  H   LEU    48      -7.987  65.331  21.689  1.00  0.00            H
ATOM    441  HA  LEU    48      -9.006  66.721  24.139  1.00  0.00            H
ATOM    442  HB2 LEU    48     -10.088  66.392  21.293  1.00  0.00            H
ATOM    443  HB3 LEU    48     -11.044  66.987  22.587  1.00  0.00            H
ATOM    444  HG  LEU    48      -9.840  68.972  22.879  1.00  0.00            H
ATOM    445 HD21 LEU    48     -10.035  69.902  20.462  1.00  0.00            H
```

```
ATOM    446 HD22 LEU    48     -10.415  68.272  19.967  1.00  0.00           H
ATOM    447 HD23 LEU    48     -11.452  69.105  21.128  1.00  0.00           H
ATOM    448 HD11 LEU    48      -7.894  69.662  21.476  1.00  0.00           H
ATOM    449 HD12 LEU    48      -7.528  68.379  22.604  1.00  0.00           H
ATOM    450 HD13 LEU    48      -7.777  68.014  20.883  1.00  0.00           H
ATOM    451  N   SER    49     -10.393  64.009  22.775  1.00100.09       1SG N
ATOM    452  CA  SER    49     -11.240  62.842  23.043  1.00100.09       1SG C
ATOM    453  C   SER    49     -10.454  61.798  23.863  1.00100.09       1SG C
ATOM    454  O   SER    49     -11.051  60.910  24.463  1.00100.09       1SG O
ATOM    455  CB  SER    49     -11.705  62.229  21.707  1.00100.09       1SG C
ATOM    456  OG  SER    49     -12.125  63.247  20.805  1.00100.09       1SG O
ATOM    457  H   SER    49     -10.069  64.120  21.823  1.00  0.00           H
ATOM    458  HA  SER    49     -12.114  63.154  23.619  1.00  0.00           H
ATOM    459  HB2 SER    49     -10.923  61.631  21.244  1.00  0.00           H
ATOM    460  HB3 SER    49     -12.533  61.540  21.883  1.00  0.00           H
ATOM    461  HG  SER    49     -12.918  63.646  21.138  1.00  0.00           H
ATOM    462  N   ARG    50      -9.127  61.959  23.947  1.00202.09       1SG N
ATOM    463  CA  ARG    50      -8.237  61.514  25.014  1.00202.09       1SG C
ATOM    464  C   ARG    50      -7.897  60.016  24.931  1.00202.09       1SG C
ATOM    465  O   ARG    50      -7.610  59.375  25.941  1.00202.09       1SG O
ATOM    466  CB  ARG    50      -8.577  62.141  26.404  1.00202.09       1SG C
ATOM    467  CG  ARG    50      -9.620  61.476  27.343  1.00202.09       1SG C
ATOM    468  CD  ARG    50     -11.065  62.034  27.410  1.00202.09       1SG C
ATOM    469  NE  ARG    50     -11.218  63.475  27.114  1.00202.09       1SG N
ATOM    470  CZ  ARG    50     -11.815  64.421  27.857  1.00202.09       1SG C
ATOM    471  NH1 ARG    50     -12.127  64.209  29.135  1.00202.09       1SG N
ATOM    472  NH2 ARG    50     -12.098  65.595  27.299  1.00202.09       1SG N1+
ATOM    473  H   ARG    50      -8.732  62.637  23.308  1.00  0.00           H
ATOM    474  HA  ARG    50      -7.292  61.984  24.740  1.00  0.00           H
ATOM    475  HB2 ARG    50      -7.637  62.106  26.956  1.00  0.00           H
ATOM    476  HB3 ARG    50      -8.763  63.209  26.296  1.00  0.00           H
ATOM    477  HG2 ARG    50      -9.676  60.408  27.130  1.00  0.00           H
ATOM    478  HG3 ARG    50      -9.218  61.525  28.356  1.00  0.00           H
ATOM    479  HD2 ARG    50     -11.707  61.496  26.716  1.00  0.00           H
ATOM    480  HD3 ARG    50     -11.485  61.800  28.387  1.00  0.00           H
ATOM    481  HE  ARG    50     -10.891  63.763  26.184  1.00  0.00           H
ATOM    482 HH12 ARG    50     -12.396  64.987  29.761  1.00  0.00           H
ATOM    483 HH11 ARG    50     -11.933  63.333  29.587  1.00  0.00           H
ATOM    484 HH22 ARG    50     -12.601  66.349  27.789  1.00  0.00           H
ATOM    485 HH21 ARG    50     -11.815  65.793  26.343  1.00  0.00           H
ATOM    486  N   GLY    51      -7.916  59.468  23.711  1.00 27.43       1SG N
ATOM    487  CA  GLY    51      -7.613  58.081  23.394  1.00 27.43       1SG C
ATOM    488  C   GLY    51      -6.342  58.043  22.551  1.00 27.43       1SG C
ATOM    489  O   GLY    51      -6.288  58.652  21.481  1.00 27.43       1SG O
ATOM    490  H   GLY    51      -8.093  60.085  22.930  1.00  0.00           H
ATOM    491  HA2 GLY    51      -7.516  57.463  24.289  1.00  0.00           H
ATOM    492  HA3 GLY    51      -8.435  57.666  22.809  1.00  0.00           H
ATOM    493  N   THR    52      -5.312  57.352  23.052  1.00109.04       1SG N
ATOM    494  CA  THR    52      -3.990  57.226  22.442  1.00109.04       1SG C
ATOM    495  C   THR    52      -4.061  56.625  21.019  1.00109.04       1SG C
ATOM    496  O   THR    52      -4.804  55.678  20.765  1.00109.04       1SG O
ATOM    497  CB  THR    52      -3.056  56.456  23.416  1.00109.04       1SG C
ATOM    498  OG1 THR    52      -1.718  56.515  22.964  1.00109.04       1SG O
ATOM    499  CG2 THR    52      -3.424  54.980  23.645  1.00109.04       1SG C
ATOM    500  H   THR    52      -5.456  56.865  23.921  1.00  0.00           H
ATOM    501  HA  THR    52      -3.606  58.244  22.354  1.00  0.00           H
ATOM    502  HB  THR    52      -3.077  56.968  24.380  1.00  0.00           H
ATOM    503  HG1 THR    52      -1.151  56.107  23.604  1.00  0.00           H
ATOM    504 HG21 THR    52      -2.778  54.520  24.394  1.00  0.00           H
ATOM    505 HG22 THR    52      -4.451  54.874  23.996  1.00  0.00           H
ATOM    506 HG23 THR    52      -3.332  54.394  22.730  1.00  0.00           H
ATOM    507  N   HIS    53      -3.313  57.225  20.091  1.00 74.02       1SG N
ATOM    508  CA  HIS    53      -3.163  56.823  18.699  1.00 74.02       1SG C
```

```
ATOM    509  C   HIS    53      -1.697  56.388  18.549  1.00 74.02         1SG C
ATOM    510  O   HIS    53      -0.803  57.042  19.090  1.00 74.02         1SG O
ATOM    511  ND1 HIS    53      -5.787  58.635  18.614  1.00 74.02         1SG N
ATOM    512  CG  HIS    53      -4.843  58.511  17.603  1.00 74.02         1SG C
ATOM    513  CB  HIS    53      -3.413  58.050  17.800  1.00 74.02         1SG C
ATOM    514  NE2 HIS    53      -6.787  59.346  16.768  1.00 74.02         1SG N
ATOM    515  CD2 HIS    53      -5.483  58.955  16.466  1.00 74.02         1SG C
ATOM    516  CE1 HIS    53      -6.905  59.118  18.069  1.00 74.02         1SG C
ATOM    517  H   HIS    53      -2.625  57.885  20.425  1.00  0.00             H
ATOM    518  HA  HIS    53      -3.832  56.003  18.429  1.00  0.00             H
ATOM    519  HD1 HIS    53      -5.704  58.414  19.607  1.00  0.00             H
ATOM    520  HB2 HIS    53      -2.834  58.895  18.172  1.00  0.00             H
ATOM    521  HB3 HIS    53      -3.026  57.845  16.803  1.00  0.00             H
ATOM    522  HD2 HIS    53      -5.105  59.045  15.458  1.00  0.00             H
ATOM    523  HE1 HIS    53      -7.806  59.311  18.634  1.00  0.00             H
ATOM    524  N   GLY    54      -1.407  55.329  17.787  1.00 73.63         1SG N
ATOM    525  CA  GLY    54      -0.123  54.637  17.854  1.00 73.63         1SG C
ATOM    526  C   GLY    54       0.838  55.122  16.774  1.00 73.63         1SG C
ATOM    527  O   GLY    54       1.151  54.373  15.856  1.00 73.63         1SG O
ATOM    528  H   GLY    54      -2.138  54.871  17.255  1.00  0.00             H
ATOM    529  HA2 GLY    54       0.343  54.732  18.837  1.00  0.00             H
ATOM    530  HA3 GLY    54      -0.305  53.571  17.707  1.00  0.00             H
ATOM    531  N   GLY    55       1.312  56.370  16.927  1.00207.74         1SG N
ATOM    532  CA  GLY    55       2.222  57.174  16.094  1.00207.74         1SG C
ATOM    533  C   GLY    55       2.499  56.670  14.670  1.00207.74         1SG C
ATOM    534  O   GLY    55       1.807  57.085  13.737  1.00207.74         1SG O
ATOM    535  H   GLY    55       0.939  56.840  17.746  1.00  0.00             H
ATOM    536  HA2 GLY    55       1.819  58.184  16.030  1.00  0.00             H
ATOM    537  HA3 GLY    55       3.172  57.267  16.622  1.00  0.00             H
ATOM    538  N   PHE    56       3.476  55.760  14.528  1.00233.10         1SG N
ATOM    539  CA  PHE    56       3.907  54.979  13.346  1.00233.10         1SG C
ATOM    540  C   PHE    56       2.776  54.495  12.482  1.00233.10         1SG C
ATOM    541  O   PHE    56       2.784  54.599  11.258  1.00233.10         1SG O
ATOM    542  CB  PHE    56       4.809  53.787  13.803  1.00233.10         1SG C
ATOM    543  CG  PHE    56       5.098  53.699  15.298  1.00233.10         1SG C
ATOM    544  CD1 PHE    56       4.192  53.036  16.154  1.00233.10         1SG C
ATOM    545  CD2 PHE    56       6.249  54.309  15.841  1.00233.10         1SG C
ATOM    546  CE1 PHE    56       4.429  52.994  17.541  1.00233.10         1SG C
ATOM    547  CE2 PHE    56       6.486  54.265  17.228  1.00233.10         1SG C
ATOM    548  CZ  PHE    56       5.575  53.609  18.079  1.00233.10         1SG C
ATOM    549  H   PHE    56       3.941  55.464  15.378  1.00  0.00             H
ATOM    550  HA  PHE    56       4.457  55.664  12.712  1.00  0.00             H
ATOM    551  HB2 PHE    56       4.359  52.834  13.519  1.00  0.00             H
ATOM    552  HB3 PHE    56       5.749  53.783  13.254  1.00  0.00             H
ATOM    553  HD1 PHE    56       3.304  52.567  15.752  1.00  0.00             H
ATOM    554  HD2 PHE    56       6.955  54.816  15.200  1.00  0.00             H
ATOM    555  HE1 PHE    56       3.731  52.487  18.192  1.00  0.00             H
ATOM    556  HE2 PHE    56       7.371  54.731  17.638  1.00  0.00             H
ATOM    557  HZ  PHE    56       5.762  53.574  19.143  1.00  0.00             H
ATOM    558  N   LEU    57       1.797  53.961  13.189  1.00141.63         1SG N
ATOM    559  CA  LEU    57       0.714  53.233  12.621  1.00141.63         1SG C
ATOM    560  C   LEU    57      -0.490  54.065  12.323  1.00141.63         1SG C
ATOM    561  O   LEU    57      -1.496  53.559  11.829  1.00141.63         1SG O
ATOM    562  CB  LEU    57       0.350  52.122  13.651  1.00141.63         1SG C
ATOM    563  CG  LEU    57      -0.410  50.885  13.113  1.00141.63         1SG C
ATOM    564  CD2 LEU    57      -0.910  50.017  14.278  1.00141.63         1SG C
ATOM    565  CD1 LEU    57       0.447  50.041  12.154  1.00141.63         1SG C
ATOM    566  H   LEU    57       1.824  54.031  14.205  1.00  0.00             H
ATOM    567  HA  LEU    57       1.035  52.772  11.686  1.00  0.00             H
ATOM    568  HB2 LEU    57       1.262  51.755  14.124  1.00  0.00             H
ATOM    569  HB3 LEU    57      -0.226  52.566  14.464  1.00  0.00             H
ATOM    570  HG  LEU    57      -1.296  51.208  12.566  1.00  0.00             H
ATOM    571 HD21 LEU    57      -1.478  49.159  13.916  1.00  0.00             H
```

```
ATOM    572 HD22 LEU    57      -0.082  49.638  14.878  1.00  0.00           H
ATOM    573 HD23 LEU    57      -1.567  50.584  14.939  1.00  0.00           H
ATOM    574 HD11 LEU    57      -0.093  49.153  11.825  1.00  0.00           H
ATOM    575 HD12 LEU    57       0.716  50.594  11.255  1.00  0.00           H
ATOM    576 HD13 LEU    57       1.370  49.707  12.630  1.00  0.00           H
ATOM    577  N   THR    58      -0.424  55.318  12.739  1.00135.69       1SG N
ATOM    578  CA  THR    58      -1.633  56.008  12.982  1.00135.69       1SG C
ATOM    579  C   THR    58      -1.573  57.451  12.642  1.00135.69       1SG C
ATOM    580  O   THR    58      -2.099  57.851  11.613  1.00135.69       1SG O
ATOM    581  CB  THR    58      -2.213  55.729  14.412  1.00135.69       1SG C
ATOM    582  OG1 THR    58      -2.222  54.358  14.767  1.00135.69       1SG O
ATOM    583  CG2 THR    58      -3.667  56.201  14.582  1.00135.69       1SG C
ATOM    584  H   THR    58       0.445  55.659  13.129  1.00  0.00           H
ATOM    585  HA  THR    58      -2.405  55.656  12.292  1.00  0.00           H
ATOM    586  HB  THR    58      -1.595  56.231  15.157  1.00  0.00           H
ATOM    587  HG1 THR    58      -2.497  53.858  14.003  1.00  0.00           H
ATOM    588 HG21 THR    58      -4.048  55.977  15.579  1.00  0.00           H
ATOM    589 HG22 THR    58      -3.763  57.277  14.435  1.00  0.00           H
ATOM    590 HG23 THR    58      -4.330  55.716  13.865  1.00  0.00           H
ATOM    591  N   ILE    59      -0.967  58.235  13.528  1.00108.86       1SG N
ATOM    592  CA  ILE    59      -0.953  59.667  13.378  1.00108.86       1SG C
ATOM    593  C   ILE    59      -0.268  60.064  12.080  1.00108.86       1SG C
ATOM    594  O   ILE    59      -0.785  60.835  11.279  1.00108.86       1SG O
ATOM    595  CB  ILE    59      -0.283  60.340  14.619  1.00108.86       1SG C
ATOM    596  CG2 ILE    59       0.065  61.836  14.430  1.00108.86       1SG C
ATOM    597  CG1 ILE    59      -1.100  60.151  15.918  1.00108.86       1SG C
ATOM    598  CD1 ILE    59      -2.449  60.896  15.941  1.00108.86       1SG C
ATOM    599  H   ILE    59      -0.459  57.824  14.294  1.00  0.00           H
ATOM    600  HA  ILE    59      -1.983  60.020  13.295  1.00  0.00           H
ATOM    601  HB  ILE    59       0.675  59.843  14.775  1.00  0.00           H
ATOM    602 HG21 ILE    59       0.415  62.286  15.359  1.00  0.00           H
ATOM    603 HG22 ILE    59       0.861  61.983  13.698  1.00  0.00           H
ATOM    604 HG23 ILE    59      -0.795  62.412  14.086  1.00  0.00           H
ATOM    605 HG12 ILE    59      -1.260  59.090  16.100  1.00  0.00           H
ATOM    606 HG13 ILE    59      -0.503  60.499  16.762  1.00  0.00           H
ATOM    607 HD11 ILE    59      -2.937  60.802  16.910  1.00  0.00           H
ATOM    608 HD12 ILE    59      -2.331  61.965  15.763  1.00  0.00           H
ATOM    609 HD13 ILE    59      -3.142  60.514  15.192  1.00  0.00           H
ATOM    610  N   ASN    60       0.871  59.425  11.853  1.00 55.50       1SG N
ATOM    611  CA  ASN    60       1.735  59.771  10.752  1.00 55.50       1SG C
ATOM    612  C   ASN    60       1.211  59.209   9.443  1.00 55.50       1SG C
ATOM    613  O   ASN    60       1.232  59.866   8.405  1.00 55.50       1SG O
ATOM    614  CB  ASN    60       3.159  59.295  11.072  1.00 55.50       1SG C
ATOM    615  CG  ASN    60       3.633  59.946  12.374  1.00 55.50       1SG C
ATOM    616  OD1 ASN    60       3.487  61.148  12.557  1.00 55.50       1SG O
ATOM    617  ND2 ASN    60       4.137  59.179  13.329  1.00 55.50       1SG N
ATOM    618  H   ASN    60       1.201  58.766  12.546  1.00  0.00           H
ATOM    619  HA  ASN    60       1.736  60.853  10.644  1.00  0.00           H
ATOM    620  HB2 ASN    60       3.213  58.207  11.134  1.00  0.00           H
ATOM    621  HB3 ASN    60       3.833  59.604  10.273  1.00  0.00           H
ATOM    622 HD22 ASN    60       4.569  59.665  14.103  1.00  0.00           H
ATOM    623 HD21 ASN    60       4.281  58.193  13.182  1.00  0.00           H
ATOM    624  N   LEU    61       0.597  58.035   9.543  1.00 92.11       1SG N
ATOM    625  CA  LEU    61      -0.171  57.421   8.488  1.00 92.11       1SG C
ATOM    626  C   LEU    61      -1.371  58.236   8.059  1.00 92.11       1SG C
ATOM    627  O   LEU    61      -1.619  58.427   6.868  1.00 92.11       1SG O
ATOM    628  CB  LEU    61      -0.574  56.001   8.987  1.00 92.11       1SG C
ATOM    629  CG  LEU    61      -1.431  55.113   8.047  1.00 92.11       1SG C
ATOM    630  CD2 LEU    61      -2.947  55.329   8.223  1.00 92.11       1SG C
ATOM    631  CD1 LEU    61      -1.130  53.619   8.264  1.00 92.11       1SG C
ATOM    632  H   LEU    61       0.569  57.609  10.454  1.00  0.00           H
ATOM    633  HA  LEU    61       0.452  57.341   7.602  1.00  0.00           H
ATOM    634  HB2 LEU    61       0.357  55.471   9.198  1.00  0.00           H
```

ATOM	635	HB3	LEU	61	-1.078	56.067	9.950	1.00	0.00		H
ATOM	636	HG	LEU	61	-1.159	55.351	7.019	1.00	0.00		H
ATOM	637	HD21	LEU	61	-3.514	54.629	7.608	1.00	0.00		H
ATOM	638	HD22	LEU	61	-3.252	55.172	9.259	1.00	0.00		H
ATOM	639	HD23	LEU	61	-3.270	56.326	7.930	1.00	0.00		H
ATOM	640	HD11	LEU	61	-1.651	53.004	7.529	1.00	0.00		H
ATOM	641	HD12	LEU	61	-0.070	53.391	8.178	1.00	0.00		H
ATOM	642	HD13	LEU	61	-1.452	53.288	9.251	1.00	0.00		H
ATOM	643	N	ALA	62	-2.098	58.735	9.050	1.00	31.83	1SG	N
ATOM	644	CA	ALA	62	-3.293	59.496	8.835	1.00	31.83	1SG	C
ATOM	645	C	ALA	62	-3.038	60.864	8.247	1.00	31.83	1SG	C
ATOM	646	O	ALA	62	-3.850	61.371	7.478	1.00	31.83	1SG	O
ATOM	647	CB	ALA	62	-4.044	59.683	10.165	1.00	31.83	1SG	C
ATOM	648	H	ALA	62	-1.869	58.490	10.010	1.00	0.00		H
ATOM	649	HA	ALA	62	-3.940	58.951	8.145	1.00	0.00		H
ATOM	650	HB1	ALA	62	-4.949	60.277	10.024	1.00	0.00		H
ATOM	651	HB2	ALA	62	-4.354	58.725	10.580	1.00	0.00		H
ATOM	652	HB3	ALA	62	-3.434	60.195	10.910	1.00	0.00		H
ATOM	653	N	PHE	63	-1.877	61.424	8.564	1.00	54.13	1SG	N
ATOM	654	CA	PHE	63	-1.397	62.612	7.911	1.00	54.13	1SG	C
ATOM	655	C	PHE	63	-0.999	62.353	6.477	1.00	54.13	1SG	C
ATOM	656	O	PHE	63	-1.328	63.124	5.577	1.00	54.13	1SG	O
ATOM	657	CB	PHE	63	-0.165	63.178	8.665	1.00	54.13	1SG	C
ATOM	658	CG	PHE	63	-0.385	63.983	9.946	1.00	54.13	1SG	C
ATOM	659	CD1	PHE	63	-1.461	63.725	10.823	1.00	54.13	1SG	C
ATOM	660	CD2	PHE	63	0.556	64.977	10.297	1.00	54.13	1SG	C
ATOM	661	CE1	PHE	63	-1.573	64.423	12.040	1.00	54.13	1SG	C
ATOM	662	CE2	PHE	63	0.440	65.683	11.510	1.00	54.13	1SG	C
ATOM	663	CZ	PHE	63	-0.627	65.405	12.385	1.00	54.13	1SG	C
ATOM	664	H	PHE	63	-1.303	60.991	9.280	1.00	0.00		H
ATOM	665	HA	PHE	63	-2.182	63.372	7.906	1.00	0.00		H
ATOM	666	HB2	PHE	63	0.523	62.367	8.898	1.00	0.00		H
ATOM	667	HB3	PHE	63	0.393	63.832	7.992	1.00	0.00		H
ATOM	668	HD1	PHE	63	-2.197	62.967	10.602	1.00	0.00		H
ATOM	669	HD2	PHE	63	1.402	65.177	9.654	1.00	0.00		H
ATOM	670	HE1	PHE	63	-2.389	64.193	12.710	1.00	0.00		H
ATOM	671	HE2	PHE	63	1.188	66.417	11.779	1.00	0.00		H
ATOM	672	HZ	PHE	63	-0.715	65.931	13.325	1.00	0.00		H
ATOM	673	N	GLY	64	-0.346	61.222	6.252	1.00	33.42	1SG	N
ATOM	674	CA	GLY	64	0.149	60.873	4.953	1.00	33.42	1SG	C
ATOM	675	C	GLY	64	-0.952	60.582	3.962	1.00	33.42	1SG	C
ATOM	676	O	GLY	64	-0.976	61.110	2.848	1.00	33.42	1SG	O
ATOM	677	H	GLY	64	-0.075	60.640	7.039	1.00	0.00		H
ATOM	678	HA2	GLY	64	0.766	61.685	4.587	1.00	0.00		H
ATOM	679	HA3	GLY	64	0.763	59.991	5.107	1.00	0.00		H
ATOM	680	N	PHE	65	-1.945	59.837	4.423	1.00	61.32	1SG	N
ATOM	681	CA	PHE	65	-3.102	59.527	3.627	1.00	61.32	1SG	C
ATOM	682	C	PHE	65	-4.254	60.508	3.805	1.00	61.32	1SG	C
ATOM	683	O	PHE	65	-5.354	60.276	3.306	1.00	61.32	1SG	O
ATOM	684	CB	PHE	65	-3.566	58.094	3.976	1.00	61.32	1SG	C
ATOM	685	CG	PHE	65	-2.567	56.970	3.700	1.00	61.32	1SG	C
ATOM	686	CD1	PHE	65	-1.675	57.014	2.602	1.00	61.32	1SG	C
ATOM	687	CD2	PHE	65	-2.551	55.844	4.549	1.00	61.32	1SG	C
ATOM	688	CE1	PHE	65	-0.775	55.956	2.371	1.00	61.32	1SG	C
ATOM	689	CE2	PHE	65	-1.659	54.780	4.312	1.00	61.32	1SG	C
ATOM	690	CZ	PHE	65	-0.770	54.837	3.223	1.00	61.32	1SG	C
ATOM	691	H	PHE	65	-1.857	59.413	5.347	1.00	0.00		H
ATOM	692	HA	PHE	65	-2.821	59.545	2.584	1.00	0.00		H
ATOM	693	HB2	PHE	65	-3.860	58.058	5.027	1.00	0.00		H
ATOM	694	HB3	PHE	65	-4.466	57.849	3.408	1.00	0.00		H
ATOM	695	HD1	PHE	65	-1.662	57.846	1.917	1.00	0.00		H
ATOM	696	HD2	PHE	65	-3.229	55.790	5.388	1.00	0.00		H
ATOM	697	HE1	PHE	65	-0.103	55.995	1.524	1.00	0.00		H

```
ATOM    698  HE2 PHE    65      -1.657  53.921   4.966  1.00  0.00          H
ATOM    699  HZ  PHE    65      -0.095  54.016   3.032  1.00  0.00          H
ATOM    700  N   ALA    66      -3.974  61.658   4.407  1.00 32.21      1SG N
ATOM    701  CA  ALA    66      -4.777  62.837   4.198  1.00 32.21      1SG C
ATOM    702  C   ALA    66      -4.278  63.621   3.005  1.00 32.21      1SG C
ATOM    703  O   ALA    66      -5.047  64.054   2.146  1.00 32.21      1SG O
ATOM    704  CB  ALA    66      -4.623  63.770   5.410  1.00 32.21      1SG C
ATOM    705  H   ALA    66      -3.083  61.753   4.875  1.00  0.00          H
ATOM    706  HA  ALA    66      -5.835  62.598   4.064  1.00  0.00          H
ATOM    707  HB1 ALA    66      -5.149  64.710   5.240  1.00  0.00          H
ATOM    708  HB2 ALA    66      -5.026  63.308   6.308  1.00  0.00          H
ATOM    709  HB3 ALA    66      -3.593  64.039   5.624  1.00  0.00          H
ATOM    710  N   VAL    67      -2.958  63.769   2.956  1.00 35.38      1SG N
ATOM    711  CA  VAL    67      -2.262  64.474   1.913  1.00 35.38      1SG C
ATOM    712  C   VAL    67      -2.394  63.795   0.581  1.00 35.38      1SG C
ATOM    713  O   VAL    67      -2.596  64.476  -0.424  1.00 35.38      1SG O
ATOM    714  CB  VAL    67      -0.750  64.568   2.319  1.00 35.38      1SG C
ATOM    715  CG1 VAL    67       0.189  65.126   1.228  1.00 35.38      1SG C
ATOM    716  CG2 VAL    67      -0.510  65.358   3.618  1.00 35.38      1SG C
ATOM    717  H   VAL    67      -2.404  63.409   3.725  1.00  0.00          H
ATOM    718  HA  VAL    67      -2.675  65.482   1.828  1.00  0.00          H
ATOM    719  HB  VAL    67      -0.405  63.553   2.518  1.00  0.00          H
ATOM    720 HG11 VAL    67       1.206  65.241   1.605  1.00  0.00          H
ATOM    721 HG12 VAL    67       0.255  64.461   0.367  1.00  0.00          H
ATOM    722 HG13 VAL    67      -0.145  66.101   0.870  1.00  0.00          H
ATOM    723 HG21 VAL    67       0.397  65.014   4.116  1.00  0.00          H
ATOM    724 HG22 VAL    67      -0.372  66.416   3.418  1.00  0.00          H
ATOM    725 HG23 VAL    67      -1.325  65.270   4.334  1.00  0.00          H
ATOM    726  N   THR    68      -2.355  62.459   0.609  1.00103.90      1SG N
ATOM    727  CA  THR    68      -2.621  61.666  -0.559  1.00103.90      1SG C
ATOM    728  C   THR    68      -3.940  62.074  -1.244  1.00103.90      1SG C
ATOM    729  O   THR    68      -4.013  62.321  -2.448  1.00103.90      1SG O
ATOM    730  CB  THR    68      -2.635  60.134  -0.278  1.00103.90      1SG C
ATOM    731  OG1 THR    68      -2.614  59.473  -1.507  1.00103.90      1SG O
ATOM    732  CG2 THR    68      -3.910  59.518   0.330  1.00103.90      1SG C
ATOM    733  H   THR    68      -2.090  61.998   1.473  1.00  0.00          H
ATOM    734  HA  THR    68      -1.811  61.883  -1.260  1.00  0.00          H
ATOM    735  HB  THR    68      -1.756  59.848   0.298  1.00  0.00          H
ATOM    736  HG1 THR    68      -1.790  59.707  -1.939  1.00  0.00          H
ATOM    737 HG21 THR    68      -3.729  58.513   0.711  1.00  0.00          H
ATOM    738 HG22 THR    68      -4.299  60.136   1.125  1.00  0.00          H
ATOM    739 HG23 THR    68      -4.722  59.432  -0.395  1.00  0.00          H
ATOM    740  N   LEU    69      -4.975  62.190  -0.410  1.00 87.41      1SG N
ATOM    741  CA  LEU    69      -6.333  62.373  -0.831  1.00 87.41      1SG C
ATOM    742  C   LEU    69      -6.549  63.792  -1.342  1.00 87.41      1SG C
ATOM    743  O   LEU    69      -7.140  63.997  -2.403  1.00 87.41      1SG O
ATOM    744  CB  LEU    69      -7.269  62.063   0.365  1.00 87.41      1SG C
ATOM    745  CG  LEU    69      -8.788  62.180   0.099  1.00 87.41      1SG C
ATOM    746  CD2 LEU    69      -9.575  61.924   1.393  1.00 87.41      1SG C
ATOM    747  CD1 LEU    69      -9.269  61.239  -1.019  1.00 87.41      1SG C
ATOM    748  H   LEU    69      -4.802  62.076   0.577  1.00  0.00          H
ATOM    749  HA  LEU    69      -6.537  61.675  -1.645  1.00  0.00          H
ATOM    750  HB2 LEU    69      -7.056  61.053   0.722  1.00  0.00          H
ATOM    751  HB3 LEU    69      -7.020  62.719   1.199  1.00  0.00          H
ATOM    752  HG  LEU    69      -9.014  63.203  -0.209  1.00  0.00          H
ATOM    753 HD21 LEU    69     -10.646  62.063   1.242  1.00  0.00          H
ATOM    754 HD22 LEU    69      -9.420  60.909   1.760  1.00  0.00          H
ATOM    755 HD23 LEU    69      -9.266  62.608   2.184  1.00  0.00          H
ATOM    756 HD11 LEU    69     -10.349  61.303  -1.148  1.00  0.00          H
ATOM    757 HD12 LEU    69      -8.820  61.493  -1.980  1.00  0.00          H
ATOM    758 HD13 LEU    69      -9.023  60.200  -0.798  1.00  0.00          H
ATOM    759  N   GLY    70      -5.988  64.752  -0.606  1.00 34.08      1SG N
ATOM    760  CA  GLY    70      -5.655  66.089  -1.049  1.00 34.08      1SG C
```

```
ATOM    761  C   GLY    70      -5.170  66.218  -2.480  1.00 34.08       1SG C
ATOM    762  O   GLY    70      -5.734  66.960  -3.290  1.00 34.08       1SG O
ATOM    763  H   GLY    70      -5.561  64.450   0.265  1.00  0.00           H
ATOM    764  HA2 GLY    70      -6.520  66.732  -0.915  1.00  0.00           H
ATOM    765  HA3 GLY    70      -4.882  66.484  -0.389  1.00  0.00           H
ATOM    766  N   ILE    71      -4.114  65.462  -2.767  1.00105.54       1SG N
ATOM    767  CA  ILE    71      -3.451  65.459  -4.045  1.00105.54       1SG C
ATOM    768  C   ILE    71      -4.289  64.900  -5.171  1.00105.54       1SG C
ATOM    769  O   ILE    71      -4.318  65.496  -6.249  1.00105.54       1SG O
ATOM    770  CB  ILE    71      -2.054  64.764  -3.929  1.00105.54       1SG C
ATOM    771  CG2 ILE    71      -1.376  64.457  -5.288  1.00105.54       1SG C
ATOM    772  CG1 ILE    71      -1.093  65.615  -3.063  1.00105.54       1SG C
ATOM    773  CD1 ILE    71       0.143  64.852  -2.568  1.00105.54       1SG C
ATOM    774  H   ILE    71      -3.701  64.900  -2.027  1.00  0.00           H
ATOM    775  HA  ILE    71      -3.269  66.501  -4.306  1.00  0.00           H
ATOM    776  HB  ILE    71      -2.198  63.807  -3.427  1.00  0.00           H
ATOM    777 HG21 ILE    71      -0.406  63.978  -5.160  1.00  0.00           H
ATOM    778 HG22 ILE    71      -1.962  63.773  -5.901  1.00  0.00           H
ATOM    779 HG23 ILE    71      -1.216  65.368  -5.865  1.00  0.00           H
ATOM    780 HG12 ILE    71      -0.774  66.500  -3.617  1.00  0.00           H
ATOM    781 HG13 ILE    71      -1.610  66.000  -2.187  1.00  0.00           H
ATOM    782 HD11 ILE    71       0.730  65.472  -1.891  1.00  0.00           H
ATOM    783 HD12 ILE    71      -0.139  63.945  -2.032  1.00  0.00           H
ATOM    784 HD13 ILE    71       0.799  64.569  -3.390  1.00  0.00           H
ATOM    785  N   LEU    72      -5.008  63.809  -4.915  1.00104.05       1SG N
ATOM    786  CA  LEU    72      -5.904  63.228  -5.896  1.00104.05       1SG C
ATOM    787  C   LEU    72      -6.952  64.197  -6.417  1.00104.05       1SG C
ATOM    788  O   LEU    72      -7.246  64.264  -7.615  1.00104.05       1SG O
ATOM    789  CB  LEU    72      -6.639  61.982  -5.313  1.00104.05       1SG C
ATOM    790  CG  LEU    72      -5.999  60.609  -5.611  1.00104.05       1SG C
ATOM    791  CD2 LEU    72      -7.049  59.487  -5.580  1.00104.05       1SG C
ATOM    792  CD1 LEU    72      -4.870  60.283  -4.632  1.00104.05       1SG C
ATOM    793  H   LEU    72      -4.895  63.358  -4.011  1.00  0.00           H
ATOM    794  HA  LEU    72      -5.306  62.923  -6.758  1.00  0.00           H
ATOM    795  HB2 LEU    72      -6.805  62.098  -4.241  1.00  0.00           H
ATOM    796  HB3 LEU    72      -7.647  61.943  -5.733  1.00  0.00           H
ATOM    797  HG  LEU    72      -5.582  60.629  -6.619  1.00  0.00           H
ATOM    798 HD21 LEU    72      -6.595  58.519  -5.801  1.00  0.00           H
ATOM    799 HD22 LEU    72      -7.524  59.413  -4.601  1.00  0.00           H
ATOM    800 HD23 LEU    72      -7.831  59.652  -6.321  1.00  0.00           H
ATOM    801 HD11 LEU    72      -4.381  59.341  -4.883  1.00  0.00           H
ATOM    802 HD12 LEU    72      -4.119  61.066  -4.654  1.00  0.00           H
ATOM    803 HD13 LEU    72      -5.233  60.201  -3.607  1.00  0.00           H
ATOM    804  N   ILE    73      -7.492  64.946  -5.464  1.00108.97       1SG N
ATOM    805  CA  ILE    73      -8.516  65.918  -5.698  1.00108.97       1SG C
ATOM    806  C   ILE    73      -8.000  67.148  -6.457  1.00108.97       1SG C
ATOM    807  O   ILE    73      -8.699  67.683  -7.321  1.00108.97       1SG O
ATOM    808  CB  ILE    73      -9.153  66.297  -4.320  1.00108.97       1SG C
ATOM    809  CG2 ILE    73     -10.032  67.568  -4.318  1.00108.97       1SG C
ATOM    810  CG1 ILE    73      -9.923  65.111  -3.683  1.00108.97       1SG C
ATOM    811  CD1 ILE    73     -11.266  64.762  -4.347  1.00108.97       1SG C
ATOM    812  H   ILE    73      -7.178  64.793  -4.511  1.00  0.00           H
ATOM    813  HA  ILE    73      -9.284  65.469  -6.330  1.00  0.00           H
ATOM    814  HB  ILE    73      -8.327  66.516  -3.641  1.00  0.00           H
ATOM    815 HG21 ILE    73     -10.520  67.705  -3.352  1.00  0.00           H
ATOM    816 HG22 ILE    73      -9.440  68.466  -4.499  1.00  0.00           H
ATOM    817 HG23 ILE    73     -10.808  67.520  -5.082  1.00  0.00           H
ATOM    818 HG12 ILE    73      -9.304  64.214  -3.683  1.00  0.00           H
ATOM    819 HG13 ILE    73     -10.102  65.329  -2.629  1.00  0.00           H
ATOM    820 HD11 ILE    73     -11.721  63.905  -3.851  1.00  0.00           H
ATOM    821 HD12 ILE    73     -11.976  65.585  -4.284  1.00  0.00           H
ATOM    822 HD13 ILE    73     -11.142  64.498  -5.397  1.00  0.00           H
ATOM    823  N   ALA    74      -6.799  67.618  -6.117  1.00 79.35       1SG N
```

```
ATOM    824  CA  ALA    74      -6.291  68.883  -6.620  1.00 79.35        1SG C
ATOM    825  C   ALA    74      -5.447  68.788  -7.859  1.00 79.35        1SG C
ATOM    826  O   ALA    74      -5.455  69.695  -8.700  1.00 79.35        1SG O
ATOM    827  CB  ALA    74      -5.455  69.530  -5.499  1.00 79.35        1SG C
ATOM    828  H   ALA    74      -6.296  67.144  -5.372  1.00  0.00            H
ATOM    829  HA  ALA    74      -7.117  69.561  -6.843  1.00  0.00            H
ATOM    830  HB1 ALA    74      -4.938  70.429  -5.837  1.00  0.00            H
ATOM    831  HB2 ALA    74      -6.091  69.822  -4.663  1.00  0.00            H
ATOM    832  HB3 ALA    74      -4.708  68.845  -5.098  1.00  0.00            H
ATOM    833  N   GLY    75      -4.699  67.695  -7.899  1.00 44.65        1SG N
ATOM    834  CA  GLY    75      -3.683  67.315  -8.835  1.00 44.65        1SG C
ATOM    835  C   GLY    75      -3.807  67.876 -10.227  1.00 44.65        1SG C
ATOM    836  O   GLY    75      -3.035  68.714 -10.694  1.00 44.65        1SG O
ATOM    837  H   GLY    75      -4.811  67.033  -7.133  1.00  0.00            H
ATOM    838  HA2 GLY    75      -2.709  67.607  -8.443  1.00  0.00            H
ATOM    839  HA3 GLY    75      -3.654  66.225  -8.890  1.00  0.00            H
ATOM    840  N   GLN    76      -4.915  67.446 -10.812  1.00 75.99        1SG N
ATOM    841  CA  GLN    76      -5.321  67.597 -12.184  1.00 75.99        1SG C
ATOM    842  C   GLN    76      -5.493  69.036 -12.658  1.00 75.99        1SG C
ATOM    843  O   GLN    76      -5.598  69.300 -13.855  1.00 75.99        1SG O
ATOM    844  CB  GLN    76      -6.675  66.858 -12.326  1.00 75.99        1SG C
ATOM    845  CG  GLN    76      -6.718  65.399 -11.800  1.00 75.99        1SG C
ATOM    846  CD  GLN    76      -8.155  64.958 -11.518  1.00 75.99        1SG C
ATOM    847  OE1 GLN    76      -9.034  65.138 -12.353  1.00 75.99        1SG O
ATOM    848  NE2 GLN    76      -8.434  64.415 -10.338  1.00 75.99        1SG N
ATOM    849  H   GLN    76      -5.491  66.847 -10.240  1.00  0.00            H
ATOM    850  HA  GLN    76      -4.572  67.118 -12.819  1.00  0.00            H
ATOM    851  HB2 GLN    76      -7.442  67.435 -11.806  1.00  0.00            H
ATOM    852  HB3 GLN    76      -6.983  66.859 -13.373  1.00  0.00            H
ATOM    853  HG2 GLN    76      -6.280  64.725 -12.536  1.00  0.00            H
ATOM    854  HG3 GLN    76      -6.141  65.263 -10.886  1.00  0.00            H
ATOM    855 HE22 GLN    76      -9.383  64.126 -10.169  1.00  0.00            H
ATOM    856 HE21 GLN    76      -7.749  64.314  -9.592  1.00  0.00            H
ATOM    857  N   VAL    77      -5.609  69.947 -11.698  1.00 74.34        1SG N
ATOM    858  CA  VAL    77      -6.130  71.268 -11.914  1.00 74.34        1SG C
ATOM    859  C   VAL    77      -5.129  72.370 -11.539  1.00 74.34        1SG C
ATOM    860  O   VAL    77      -5.224  73.492 -12.051  1.00 74.34        1SG O
ATOM    861  CB  VAL    77      -7.438  71.419 -11.065  1.00 74.34        1SG C
ATOM    862  CG1 VAL    77      -8.257  72.667 -11.438  1.00 74.34        1SG C
ATOM    863  CG2 VAL    77      -8.365  70.185 -11.105  1.00 74.34        1SG C
ATOM    864  H   VAL    77      -5.501  69.620 -10.742  1.00  0.00            H
ATOM    865  HA  VAL    77      -6.385  71.418 -12.964  1.00  0.00            H
ATOM    866  HB  VAL    77      -7.144  71.536 -10.019  1.00  0.00            H
ATOM    867 HG11 VAL    77      -9.150  72.743 -10.817  1.00  0.00            H
ATOM    868 HG12 VAL    77      -7.687  73.583 -11.286  1.00  0.00            H
ATOM    869 HG13 VAL    77      -8.573  72.630 -12.480  1.00  0.00            H
ATOM    870 HG21 VAL    77      -9.309  70.372 -10.594  1.00  0.00            H
ATOM    871 HG22 VAL    77      -8.592  69.884 -12.129  1.00  0.00            H
ATOM    872 HG23 VAL    77      -7.915  69.333 -10.594  1.00  0.00            H
ATOM    873  N   SER    78      -4.245  72.056 -10.595  1.00111.39        1SG N
ATOM    874  CA  SER    78      -3.555  72.995  -9.720  1.00111.39        1SG C
ATOM    875  C   SER    78      -2.048  72.731  -9.787  1.00111.39        1SG C
ATOM    876  O   SER    78      -1.255  73.668  -9.833  1.00111.39        1SG O
ATOM    877  CB  SER    78      -4.163  72.760  -8.329  1.00111.39        1SG C
ATOM    878  OG  SER    78      -4.111  73.930  -7.571  1.00111.39        1SG O
ATOM    879  H   SER    78      -4.230  71.086 -10.306  1.00  0.00            H
ATOM    880  HA  SER    78      -3.745  74.020 -10.031  1.00  0.00            H
ATOM    881  HB2 SER    78      -5.206  72.460  -8.407  1.00  0.00            H
ATOM    882  HB3 SER    78      -3.661  71.955  -7.789  1.00  0.00            H
ATOM    883  HG  SER    78      -3.323  73.844  -6.998  1.00  0.00            H
ATOM    884  N   GLY    79      -1.666  71.452  -9.825  1.00 37.05        1SG N
ATOM    885  CA  GLY    79      -0.344  70.963  -9.478  1.00 37.05        1SG C
ATOM    886  C   GLY    79      -0.542  70.062  -8.263  1.00 37.05        1SG C
```

ATOM	887	O	GLY	79	-0.175	68.894	-8.306	1.00	37.05	1SG	O
ATOM	888	H	GLY	79	-2.359	70.719	-9.892	1.00	0.00		H
ATOM	889	HA2	GLY	79	0.072	70.388	-10.307	1.00	0.00		H
ATOM	890	HA3	GLY	79	0.362	71.759	-9.234	1.00	0.00		H
ATOM	891	N	ALA	80	-1.196	70.606	-7.221	1.00	76.23	1SG	N
ATOM	892	CA	ALA	80	-1.401	70.027	-5.895	1.00	76.23	1SG	C
ATOM	893	C	ALA	80	-0.122	70.172	-5.094	1.00	76.23	1SG	C
ATOM	894	O	ALA	80	0.439	69.189	-4.611	1.00	76.23	1SG	O
ATOM	895	CB	ALA	80	-1.965	68.602	-5.872	1.00	76.23	1SG	C
ATOM	896	H	ALA	80	-1.381	71.603	-7.260	1.00	0.00		H
ATOM	897	HA	ALA	80	-2.134	70.667	-5.402	1.00	0.00		H
ATOM	898	HB1	ALA	80	-2.146	68.304	-4.840	1.00	0.00		H
ATOM	899	HB2	ALA	80	-2.904	68.558	-6.398	1.00	0.00		H
ATOM	900	HB3	ALA	80	-1.283	67.868	-6.301	1.00	0.00		H
ATOM	901	N	HIS	81	0.352	71.412	-4.986	1.00	86.78	1SG	N
ATOM	902	CA	HIS	81	1.553	71.707	-4.229	1.00	86.78	1SG	C
ATOM	903	C	HIS	81	1.215	71.621	-2.739	1.00	86.78	1SG	C
ATOM	904	O	HIS	81	1.981	71.040	-1.971	1.00	86.78	1SG	O
ATOM	905	ND1	HIS	81	2.648	74.690	-6.426	1.00	86.78	1SG	N
ATOM	906	CG	HIS	81	2.386	73.408	-5.992	1.00	86.78	1SG	C
ATOM	907	CB	HIS	81	2.031	73.130	-4.551	1.00	86.78	1SG	C
ATOM	908	NE2	HIS	81	2.867	73.396	-8.208	1.00	86.78	1SG	N
ATOM	909	CD2	HIS	81	2.538	72.605	-7.102	1.00	86.78	1SG	C
ATOM	910	CE1	HIS	81	2.908	74.635	-7.734	1.00	86.78	1SG	C
ATOM	911	H	HIS	81	-0.233	72.184	-5.297	1.00	0.00		H
ATOM	912	HA	HIS	81	2.338	70.988	-4.469	1.00	0.00		H
ATOM	913	HD1	HIS	81	2.627	75.508	-5.820	1.00	0.00		H
ATOM	914	HB2	HIS	81	1.262	73.848	-4.264	1.00	0.00		H
ATOM	915	HB3	HIS	81	2.909	73.355	-3.944	1.00	0.00		H
ATOM	916	HD2	HIS	81	2.427	71.533	-7.190	1.00	0.00		H
ATOM	917	HE1	HIS	81	3.132	75.500	-8.343	1.00	0.00		H
ATOM	918	N	LEU	82	0.076	72.212	-2.337	1.00	80.56	1SG	N
ATOM	919	CA	LEU	82	-0.534	72.198	-1.004	1.00	80.56	1SG	C
ATOM	920	C	LEU	82	0.387	72.814	0.074	1.00	80.56	1SG	C
ATOM	921	O	LEU	82	0.215	72.591	1.273	1.00	80.56	1SG	O
ATOM	922	CB	LEU	82	-1.014	70.781	-0.588	1.00	80.56	1SG	C
ATOM	923	CG	LEU	82	-1.788	69.949	-1.640	1.00	80.56	1SG	C
ATOM	924	CD2	LEU	82	-2.915	70.733	-2.330	1.00	80.56	1SG	C
ATOM	925	CD1	LEU	82	-2.339	68.659	-1.013	1.00	80.56	1SG	C
ATOM	926	H	LEU	82	-0.498	72.694	-3.043	1.00	0.00		H
ATOM	927	HA	LEU	82	-1.402	72.844	-1.096	1.00	0.00		H
ATOM	928	HB2	LEU	82	-0.155	70.194	-0.258	1.00	0.00		H
ATOM	929	HB3	LEU	82	-1.648	70.899	0.292	1.00	0.00		H
ATOM	930	HG	LEU	82	-1.082	69.644	-2.411	1.00	0.00		H
ATOM	931	HD21	LEU	82	-3.562	70.079	-2.913	1.00	0.00		H
ATOM	932	HD22	LEU	82	-3.520	71.274	-1.607	1.00	0.00		H
ATOM	933	HD23	LEU	82	-2.510	71.469	-3.020	1.00	0.00		H
ATOM	934	HD11	LEU	82	-2.846	68.048	-1.759	1.00	0.00		H
ATOM	935	HD12	LEU	82	-1.535	68.054	-0.594	1.00	0.00		H
ATOM	936	HD13	LEU	82	-3.051	68.871	-0.215	1.00	0.00		H
ATOM	937	N	ASN	83	1.442	73.509	-0.364	1.00	71.33	1SG	N
ATOM	938	CA	ASN	83	2.684	73.713	0.366	1.00	71.33	1SG	C
ATOM	939	C	ASN	83	3.448	74.849	-0.334	1.00	71.33	1SG	C
ATOM	940	O	ASN	83	3.869	74.677	-1.487	1.00	71.33	1SG	O
ATOM	941	CB	ASN	83	3.501	72.395	0.379	1.00	71.33	1SG	C
ATOM	942	CG	ASN	83	4.731	72.416	1.287	1.00	71.33	1SG	C
ATOM	943	OD1	ASN	83	5.273	73.477	1.579	1.00	71.33	1SG	O
ATOM	944	ND2	ASN	83	5.166	71.257	1.772	1.00	71.33	1SG	N
ATOM	945	H	ASN	83	1.475	73.623	-1.368	1.00	0.00		H
ATOM	946	HA	ASN	83	2.435	73.963	1.394	1.00	0.00		H
ATOM	947	HB2	ASN	83	2.865	71.566	0.687	1.00	0.00		H
ATOM	948	HB3	ASN	83	3.820	72.161	-0.630	1.00	0.00		H
ATOM	949	HD22	ASN	83	5.939	71.216	2.434	1.00	0.00		H

```
ATOM    950 HD21 ASN    83       4.739  70.392   1.474  1.00  0.00           H
ATOM    951  N   PRO    84       3.641  76.017   0.320  1.00 77.27       1SG N
ATOM    952  CA  PRO    84       4.485  77.113  -0.182  1.00 77.27       1SG C
ATOM    953  C   PRO    84       5.936  76.703  -0.504  1.00 77.27       1SG C
ATOM    954  O   PRO    84       6.505  77.224  -1.460  1.00 77.27       1SG O
ATOM    955  CD  PRO    84       2.924  76.412   1.532  1.00 77.27       1SG C
ATOM    956  CB  PRO    84       4.472  78.166   0.936  1.00 77.27       1SG C
ATOM    957  CG  PRO    84       3.187  77.904   1.707  1.00 77.27       1SG C
ATOM    958  HA  PRO    84       4.023  77.524  -1.081  1.00  0.00           H
ATOM    959  HD3 PRO    84       1.856  76.202   1.456  1.00  0.00           H
ATOM    960  HD2 PRO    84       3.322  75.855   2.381  1.00  0.00           H
ATOM    961  HB2 PRO    84       5.323  78.041   1.604  1.00  0.00           H
ATOM    962  HB3 PRO    84       4.533  79.179   0.537  1.00  0.00           H
ATOM    963  HG3 PRO    84       2.368  78.454   1.245  1.00  0.00           H
ATOM    964  HG2 PRO    84       3.231  78.215   2.750  1.00  0.00           H
ATOM    965  N   ALA    85       6.517  75.754   0.250  1.00 28.62       1SG N
ATOM    966  CA  ALA    85       7.886  75.264   0.081  1.00 28.62       1SG C
ATOM    967  C   ALA    85       8.029  74.522  -1.257  1.00 28.62       1SG C
ATOM    968  O   ALA    85       8.907  74.847  -2.055  1.00 28.62       1SG O
ATOM    969  CB  ALA    85       8.224  74.334   1.262  1.00 28.62       1SG C
ATOM    970  H   ALA    85       5.964  75.282   0.956  1.00  0.00           H
ATOM    971  HA  ALA    85       8.568  76.115   0.082  1.00  0.00           H
ATOM    972  HB1 ALA    85       9.297  74.200   1.366  1.00  0.00           H
ATOM    973  HB2 ALA    85       7.877  74.745   2.207  1.00  0.00           H
ATOM    974  HB3 ALA    85       7.793  73.339   1.154  1.00  0.00           H
ATOM    975  N   VAL    86       7.087  73.608  -1.547  1.00 38.79       1SG N
ATOM    976  CA  VAL    86       6.974  72.882  -2.816  1.00 38.79       1SG C
ATOM    977  C   VAL    86       6.836  73.838  -3.987  1.00 38.79       1SG C
ATOM    978  O   VAL    86       7.582  73.750  -4.957  1.00 38.79       1SG O
ATOM    979  CB  VAL    86       5.767  71.888  -2.772  1.00 38.79       1SG C
ATOM    980  CG1 VAL    86       5.456  71.191  -4.116  1.00 38.79       1SG C
ATOM    981  CG2 VAL    86       5.893  70.808  -1.680  1.00 38.79       1SG C
ATOM    982  H   VAL    86       6.392  73.425  -0.841  1.00  0.00           H
ATOM    983  HA  VAL    86       7.902  72.330  -2.960  1.00  0.00           H
ATOM    984  HB  VAL    86       4.881  72.475  -2.538  1.00  0.00           H
ATOM    985 HG11 VAL    86       4.658  70.456  -4.000  1.00  0.00           H
ATOM    986 HG12 VAL    86       5.120  71.890  -4.883  1.00  0.00           H
ATOM    987 HG13 VAL    86       6.324  70.663  -4.511  1.00  0.00           H
ATOM    988 HG21 VAL    86       4.916  70.385  -1.443  1.00  0.00           H
ATOM    989 HG22 VAL    86       6.488  69.965  -2.015  1.00  0.00           H
ATOM    990 HG23 VAL    86       6.337  71.182  -0.760  1.00  0.00           H
ATOM    991  N   THR    87       5.919  74.793  -3.848  1.00 40.93       1SG N
ATOM    992  CA  THR    87       5.613  75.760  -4.881  1.00 40.93       1SG C
ATOM    993  C   THR    87       6.827  76.591  -5.283  1.00 40.93       1SG C
ATOM    994  O   THR    87       7.066  76.858  -6.462  1.00 40.93       1SG O
ATOM    995  CB  THR    87       4.512  76.727  -4.342  1.00 40.93       1SG C
ATOM    996  OG1 THR    87       3.347  76.033  -3.943  1.00 40.93       1SG O
ATOM    997  CG2 THR    87       4.026  77.786  -5.350  1.00 40.93       1SG C
ATOM    998  H   THR    87       5.387  74.807  -2.990  1.00  0.00           H
ATOM    999  HA  THR    87       5.254  75.228  -5.764  1.00  0.00           H
ATOM   1000  HB  THR    87       4.888  77.255  -3.466  1.00  0.00           H
ATOM   1001  HG1 THR    87       3.554  75.430  -3.232  1.00  0.00           H
ATOM   1002 HG21 THR    87       3.249  78.409  -4.909  1.00  0.00           H
ATOM   1003 HG22 THR    87       4.829  78.457  -5.655  1.00  0.00           H
ATOM   1004 HG23 THR    87       3.620  77.334  -6.255  1.00  0.00           H
ATOM   1005  N   PHE    88       7.599  76.972  -4.270  1.00132.23       1SG N
ATOM   1006  CA  PHE    88       8.799  77.742  -4.437  1.00132.23       1SG C
ATOM   1007  C   PHE    88       9.895  76.943  -5.124  1.00132.23       1SG C
ATOM   1008  O   PHE    88      10.558  77.422  -6.045  1.00132.23       1SG O
ATOM   1009  CB  PHE    88       9.271  78.224  -3.043  1.00132.23       1SG C
ATOM   1010  CG  PHE    88      10.257  79.380  -3.044  1.00132.23       1SG C
ATOM   1011  CD1 PHE    88      11.641  79.141  -3.176  1.00132.23       1SG C
ATOM   1012  CD2 PHE    88       9.790  80.703  -2.892  1.00132.23       1SG C
```

ATOM	1013	CE1	PHE	88	12.549	80.216	-3.157	1.00132.23	1SG	C
ATOM	1014	CE2	PHE	88	10.700	81.778	-2.870	1.00132.23	1SG	C
ATOM	1015	CZ	PHE	88	12.080	81.535	-3.001	1.00132.23	1SG	C
ATOM	1016	H	PHE	88	7.343	76.703	-3.325	1.00 0.00		H
ATOM	1017	HA	PHE	88	8.568	78.615	-5.051	1.00 0.00		H
ATOM	1018	HB2	PHE	88	8.411	78.556	-2.464	1.00 0.00		H
ATOM	1019	HB3	PHE	88	9.699	77.399	-2.469	1.00 0.00		H
ATOM	1020	HD1	PHE	88	12.015	78.134	-3.299	1.00 0.00		H
ATOM	1021	HD2	PHE	88	8.733	80.899	-2.786	1.00 0.00		H
ATOM	1022	HE1	PHE	88	13.607	80.028	-3.274	1.00 0.00		H
ATOM	1023	HE2	PHE	88	10.339	82.789	-2.751	1.00 0.00		H
ATOM	1024	HZ	PHE	88	12.781	82.356	-2.989	1.00 0.00		H
ATOM	1025	N	ALA	89	10.019	75.684	-4.715	1.00 30.73	1SG	N
ATOM	1026	CA	ALA	89	10.965	74.758	-5.279	1.00 30.73	1SG	C
ATOM	1027	C	ALA	89	10.682	74.410	-6.726	1.00 30.73	1SG	C
ATOM	1028	O	ALA	89	11.593	74.361	-7.552	1.00 30.73	1SG	O
ATOM	1029	CB	ALA	89	10.961	73.493	-4.421	1.00 30.73	1SG	C
ATOM	1030	H	ALA	89	9.429	75.354	-3.958	1.00 0.00		H
ATOM	1031	HA	ALA	89	11.958	75.210	-5.223	1.00 0.00		H
ATOM	1032	HB1	ALA	89	11.836	72.901	-4.669	1.00 0.00		H
ATOM	1033	HB2	ALA	89	11.032	73.729	-3.360	1.00 0.00		H
ATOM	1034	HB3	ALA	89	10.075	72.876	-4.570	1.00 0.00		H
ATOM	1035	N	MET	90	9.398	74.258	-7.037	1.00 77.53	1SG	N
ATOM	1036	CA	MET	90	8.950	74.042	-8.389	1.00 77.53	1SG	C
ATOM	1037	C	MET	90	9.130	75.235	-9.275	1.00 77.53	1SG	C
ATOM	1038	O	MET	90	9.459	75.080	-10.454	1.00 77.53	1SG	O
ATOM	1039	CB	MET	90	7.450	73.646	-8.421	1.00 77.53	1SG	C
ATOM	1040	CG	MET	90	7.082	72.268	-7.833	1.00 77.53	1SG	C
ATOM	1041	SD	MET	90	7.984	70.833	-8.475	1.00 77.53	1SG	S
ATOM	1042	CE	MET	90	9.146	70.537	-7.113	1.00 77.53	1SG	C
ATOM	1043	H	MET	90	8.713	74.249	-6.283	1.00 0.00		H
ATOM	1044	HA	MET	90	9.534	73.227	-8.821	1.00 0.00		H
ATOM	1045	HB2	MET	90	6.850	74.410	-7.924	1.00 0.00		H
ATOM	1046	HB3	MET	90	7.108	73.641	-9.458	1.00 0.00		H
ATOM	1047	HG2	MET	90	7.167	72.253	-6.753	1.00 0.00		H
ATOM	1048	HG3	MET	90	6.024	72.091	-8.032	1.00 0.00		H
ATOM	1049	HE1	MET	90	9.763	69.663	-7.320	1.00 0.00		H
ATOM	1050	HE2	MET	90	9.803	71.394	-6.973	1.00 0.00		H
ATOM	1051	HE3	MET	90	8.608	70.352	-6.183	1.00 0.00		H
ATOM	1052	N	CYS	91	8.971	76.422	-8.698	1.00 35.19	1SG	N
ATOM	1053	CA	CYS	91	9.292	77.621	-9.411	1.00 35.19	1SG	C
ATOM	1054	C	CYS	91	10.728	77.640	-9.905	1.00 35.19	1SG	C
ATOM	1055	O	CYS	91	10.983	77.774	-11.102	1.00 35.19	1SG	O
ATOM	1056	CB	CYS	91	9.010	78.908	-8.621	1.00 35.19	1SG	C
ATOM	1057	SG	CYS	91	9.286	80.348	-9.718	1.00 35.19	1SG	S
ATOM	1058	H	CYS	91	8.651	76.479	-7.738	1.00 0.00		H
ATOM	1059	HA	CYS	91	8.650	77.641	-10.292	1.00 0.00		H
ATOM	1060	HB2	CYS	91	7.991	78.896	-8.254	1.00 0.00		H
ATOM	1061	HB3	CYS	91	9.653	79.011	-7.749	1.00 0.00		H
ATOM	1062	HG	CYS	91	8.609	79.849	-10.768	1.00 0.00		H
ATOM	1063	N	PHE	92	11.637	77.444	-8.957	1.00114.42	1SG	N
ATOM	1064	CA	PHE	92	13.047	77.467	-9.231	1.00114.42	1SG	C
ATOM	1065	C	PHE	92	13.483	76.323	-10.140	1.00114.42	1SG	C
ATOM	1066	O	PHE	92	14.301	76.539	-11.034	1.00114.42	1SG	O
ATOM	1067	CB	PHE	92	13.823	77.431	-7.894	1.00114.42	1SG	C
ATOM	1068	CG	PHE	92	14.085	78.797	-7.276	1.00114.42	1SG	C
ATOM	1069	CD1	PHE	92	13.016	79.629	-6.880	1.00114.42	1SG	C
ATOM	1070	CD2	PHE	92	15.412	79.251	-7.107	1.00114.42	1SG	C
ATOM	1071	CE1	PHE	92	13.268	80.902	-6.336	1.00114.42	1SG	C
ATOM	1072	CE2	PHE	92	15.665	80.520	-6.552	1.00114.42	1SG	C
ATOM	1073	CZ	PHE	92	14.593	81.348	-6.168	1.00114.42	1SG	C
ATOM	1074	H	PHE	92	11.331	77.329	-7.997	1.00 0.00		H
ATOM	1075	HA	PHE	92	13.292	78.395	-9.753	1.00 0.00		H

```
ATOM   1076  HB2 PHE    92      13.312  76.802  -7.163  1.00  0.00          H
ATOM   1077  HB3 PHE    92      14.792  76.956  -8.041  1.00  0.00          H
ATOM   1078  HD1 PHE    92      11.994  79.304  -6.994  1.00  0.00          H
ATOM   1079  HD2 PHE    92      16.245  78.631  -7.406  1.00  0.00          H
ATOM   1080  HE1 PHE    92      12.442  81.529  -6.035  1.00  0.00          H
ATOM   1081  HE2 PHE    92      16.682  80.861  -6.427  1.00  0.00          H
ATOM   1082  HZ  PHE    92      14.785  82.323  -5.744  1.00  0.00          H
ATOM   1083  N   LEU    93      12.939  75.122  -9.914  1.00139.08      1SG N
ATOM   1084  CA  LEU    93      13.543  73.916 -10.433  1.00139.08      1SG C
ATOM   1085  C   LEU    93      12.649  72.846 -11.068  1.00139.08      1SG C
ATOM   1086  O   LEU    93      13.187  71.866 -11.585  1.00139.08      1SG O
ATOM   1087  CB  LEU    93      14.643  73.365  -9.483  1.00139.08      1SG C
ATOM   1088  CG  LEU    93      16.137  73.716  -9.728  1.00139.08      1SG C
ATOM   1089  CD2 LEU    93      16.619  74.967  -8.979  1.00139.08      1SG C
ATOM   1090  CD1 LEU    93      16.567  73.751 -11.204  1.00139.08      1SG C
ATOM   1091  H   LEU    93      12.306  75.020  -9.125  1.00  0.00          H
ATOM   1092  HA  LEU    93      14.053  74.197 -11.353  1.00  0.00          H
ATOM   1093  HB2 LEU    93      14.380  73.624  -8.458  1.00  0.00          H
ATOM   1094  HB3 LEU    93      14.596  72.277  -9.518  1.00  0.00          H
ATOM   1095  HG  LEU    93      16.692  72.890  -9.279  1.00  0.00          H
ATOM   1096 HD21 LEU    93      17.708  75.025  -8.991  1.00  0.00          H
ATOM   1097 HD22 LEU    93      16.251  75.881  -9.441  1.00  0.00          H
ATOM   1098 HD23 LEU    93      16.307  74.956  -7.934  1.00  0.00          H
ATOM   1099 HD11 LEU    93      17.653  73.714 -11.296  1.00  0.00          H
ATOM   1100 HD12 LEU    93      16.164  72.903 -11.754  1.00  0.00          H
ATOM   1101 HD13 LEU    93      16.233  74.662 -11.702  1.00  0.00          H
ATOM   1102  N   ALA    94      11.329  73.045 -11.152  1.00 81.59      1SG N
ATOM   1103  CA  ALA    94      10.551  72.451 -12.247  1.00 81.59      1SG C
ATOM   1104  C   ALA    94      10.557  73.442 -13.424  1.00 81.59      1SG C
ATOM   1105  O   ALA    94      10.576  73.015 -14.578  1.00 81.59      1SG O
ATOM   1106  CB  ALA    94       9.094  72.208 -11.826  1.00 81.59      1SG C
ATOM   1107  H   ALA    94      10.936  73.853 -10.686  1.00  0.00          H
ATOM   1108  HA  ALA    94      10.974  71.499 -12.575  1.00  0.00          H
ATOM   1109  HB1 ALA    94       8.554  71.682 -12.613  1.00  0.00          H
ATOM   1110  HB2 ALA    94       9.055  71.575 -10.943  1.00  0.00          H
ATOM   1111  HB3 ALA    94       8.539  73.120 -11.619  1.00  0.00          H
ATOM   1112  N   ARG    95      10.626  74.748 -13.111  1.00184.48      1SG N
ATOM   1113  CA  ARG    95      10.655  75.931 -13.976  1.00184.48      1SG C
ATOM   1114  C   ARG    95       9.257  76.545 -14.152  1.00184.48      1SG C
ATOM   1115  O   ARG    95       8.962  77.145 -15.184  1.00184.48      1SG O
ATOM   1116  CB  ARG    95      11.527  75.784 -15.247  1.00184.48      1SG C
ATOM   1117  CG  ARG    95      13.007  75.457 -14.942  1.00184.48      1SG C
ATOM   1118  CD  ARG    95      13.611  74.409 -15.889  1.00184.48      1SG C
ATOM   1119  NE  ARG    95      13.282  73.043 -15.441  1.00184.48      1SG N
ATOM   1120  CZ  ARG    95      14.127  72.092 -15.010  1.00184.48      1SG C
ATOM   1121  NH1 ARG    95      15.430  72.328 -14.885  1.00184.48      1SG N
ATOM   1122  NH2 ARG    95      13.670  70.885 -14.683  1.00184.48      1SG N1+
ATOM   1123  H   ARG    95      10.580  74.957 -12.121  1.00  0.00          H
ATOM   1124  HA  ARG    95      11.172  76.681 -13.378  1.00  0.00          H
ATOM   1125  HB2 ARG    95      11.087  75.040 -15.910  1.00  0.00          H
ATOM   1126  HB3 ARG    95      11.492  76.712 -15.819  1.00  0.00          H
ATOM   1127  HG2 ARG    95      13.591  76.378 -14.992  1.00  0.00          H
ATOM   1128  HG3 ARG    95      13.128  75.114 -13.913  1.00  0.00          H
ATOM   1129  HD2 ARG    95      13.254  74.555 -16.910  1.00  0.00          H
ATOM   1130  HD3 ARG    95      14.691  74.542 -15.931  1.00  0.00          H
ATOM   1131  HE  ARG    95      12.272  72.871 -15.396  1.00  0.00          H
ATOM   1132 HH12 ARG    95      16.012  71.648 -14.366  1.00  0.00          H
ATOM   1133 HH11 ARG    95      15.873  73.181 -15.176  1.00  0.00          H
ATOM   1134 HH22 ARG    95      14.319  70.156 -14.393  1.00  0.00          H
ATOM   1135 HH21 ARG    95      12.705  70.612 -14.788  1.00  0.00          H
ATOM   1136  N   GLU    96       8.391  76.374 -13.140  1.00 90.97      1SG N
ATOM   1137  CA  GLU    96       7.045  76.942 -13.018  1.00 90.97      1SG C
ATOM   1138  C   GLU    96       7.090  78.497 -13.101  1.00 90.97      1SG C
```

```
ATOM   1139  O   GLU    96       7.707  79.108 -12.224  1.00 90.97       1SG O
ATOM   1140  CB  GLU    96       6.397  76.330 -11.732  1.00 90.97       1SG C
ATOM   1141  CG  GLU    96       5.513  77.199 -10.809  1.00 90.97       1SG C
ATOM   1142  CD  GLU    96       4.285  77.769 -11.492  1.00 90.97       1SG C
ATOM   1143  OE1 GLU    96       4.020  77.423 -12.666  1.00 90.97       1SG O
ATOM   1144  OE2 GLU    96       3.652  78.690 -10.941  1.00 90.97       1SG O1-
ATOM   1145  H   GLU    96       8.754  75.904 -12.322  1.00  0.00           H
ATOM   1146  HA  GLU    96       6.470  76.544 -13.851  1.00  0.00           H
ATOM   1147  HB2 GLU    96       5.810  75.462 -12.036  1.00  0.00           H
ATOM   1148  HB3 GLU    96       7.166  75.914 -11.093  1.00  0.00           H
ATOM   1149  HG2 GLU    96       5.171  76.600  -9.965  1.00  0.00           H
ATOM   1150  HG3 GLU    96       6.094  78.015 -10.384  1.00  0.00           H
ATOM   1151  N   PRO    97       6.499  79.151 -14.140  1.00150.91       1SG N
ATOM   1152  CA  PRO    97       6.517  80.615 -14.327  1.00150.91       1SG C
ATOM   1153  C   PRO    97       6.055  81.430 -13.103  1.00150.91       1SG C
ATOM   1154  O   PRO    97       4.899  81.320 -12.683  1.00150.91       1SG O
ATOM   1155  CD  PRO    97       5.879  78.498 -15.299  1.00150.91       1SG C
ATOM   1156  CB  PRO    97       5.594  80.891 -15.529  1.00150.91       1SG C
ATOM   1157  CG  PRO    97       5.586  79.597 -16.322  1.00150.91       1SG C
ATOM   1158  HA  PRO    97       7.543  80.882 -14.589  1.00  0.00           H
ATOM   1159  HD3 PRO    97       6.530  77.744 -15.743  1.00  0.00           H
ATOM   1160  HD2 PRO    97       4.960  78.000 -14.992  1.00  0.00           H
ATOM   1161  HB2 PRO    97       4.577  81.126 -15.217  1.00  0.00           H
ATOM   1162  HB3 PRO    97       5.946  81.734 -16.126  1.00  0.00           H
ATOM   1163  HG3 PRO    97       6.397  79.622 -17.052  1.00  0.00           H
ATOM   1164  HG2 PRO    97       4.660  79.431 -16.873  1.00  0.00           H
ATOM   1165  N   TRP    98       6.957  82.283 -12.598  1.00140.00       1SG N
ATOM   1166  CA  TRP    98       6.898  83.081 -11.367  1.00140.00       1SG C
ATOM   1167  C   TRP    98       5.664  83.986 -11.176  1.00140.00       1SG C
ATOM   1168  O   TRP    98       5.375  84.419 -10.064  1.00140.00       1SG O
ATOM   1169  CB  TRP    98       8.201  83.895 -11.224  1.00140.00       1SG C
ATOM   1170  CG  TRP    98       8.517  84.852 -12.336  1.00140.00       1SG C
ATOM   1171  CD2 TRP    98       8.062  86.229 -12.513  1.00140.00       1SG C
ATOM   1172  CD1 TRP    98       9.323  84.590 -13.390  1.00140.00       1SG C
ATOM   1173  NE1 TRP    98       9.373  85.694 -14.218  1.00140.00       1SG N
ATOM   1174  CE2 TRP    98       8.619  86.738 -13.725  1.00140.00       1SG C
ATOM   1175  CE3 TRP    98       7.241  87.108 -11.767  1.00140.00       1SG C
ATOM   1176  CZ2 TRP    98       8.367  88.045 -14.179  1.00140.00       1SG C
ATOM   1177  CZ3 TRP    98       6.985  88.423 -12.208  1.00140.00       1SG C
ATOM   1178  CH2 TRP    98       7.543  88.890 -13.413  1.00140.00       1SG C
ATOM   1179  H   TRP    98       7.867  82.264 -13.034  1.00  0.00           H
ATOM   1180  HA  TRP    98       6.878  82.363 -10.553  1.00  0.00           H
ATOM   1181  HB2 TRP    98       8.189  84.458 -10.289  1.00  0.00           H
ATOM   1182  HB3 TRP    98       9.039  83.203 -11.124  1.00  0.00           H
ATOM   1183  HD1 TRP    98       9.844  83.655 -13.544  1.00  0.00           H
ATOM   1184  HE1 TRP    98       9.951  85.734 -15.047  1.00  0.00           H
ATOM   1185  HE3 TRP    98       6.809  86.767 -10.837  1.00  0.00           H
ATOM   1186  HZ2 TRP    98       8.807  88.402 -15.098  1.00  0.00           H
ATOM   1187  HZ3 TRP    98       6.360  89.075 -11.614  1.00  0.00           H
ATOM   1188  HH2 TRP    98       7.348  89.900 -13.744  1.00  0.00           H
ATOM   1189  N   ILE    99       4.902  84.253 -12.235  1.00 90.92       1SG N
ATOM   1190  CA  ILE    99       3.695  85.081 -12.255  1.00 90.92       1SG C
ATOM   1191  C   ILE    99       2.456  84.372 -11.637  1.00 90.92       1SG C
ATOM   1192  O   ILE    99       1.324  84.715 -11.975  1.00 90.92       1SG O
ATOM   1193  CB  ILE    99       3.446  85.508 -13.736  1.00 90.92       1SG C
ATOM   1194  CG2 ILE    99       4.540  86.489 -14.204  1.00 90.92       1SG C
ATOM   1195  CG1 ILE    99       3.310  84.315 -14.720  1.00 90.92       1SG C
ATOM   1196  CD1 ILE    99       2.801  84.709 -16.114  1.00 90.92       1SG C
ATOM   1197  H   ILE    99       5.191  83.837 -13.105  1.00  0.00           H
ATOM   1198  HA  ILE    99       3.869  85.975 -11.652  1.00  0.00           H
ATOM   1199  HB  ILE    99       2.508  86.067 -13.760  1.00  0.00           H
ATOM   1200 HG21 ILE    99       4.311  86.921 -15.178  1.00  0.00           H
ATOM   1201 HG22 ILE    99       4.637  87.323 -13.509  1.00  0.00           H
```

```
ATOM   1202 HG23 ILE    99       5.518  86.011 -14.276  1.00  0.00           H
ATOM   1203 HG12 ILE    99       4.262  83.794 -14.822  1.00  0.00           H
ATOM   1204 HG13 ILE    99       2.610  83.587 -14.313  1.00  0.00           H
ATOM   1205 HD11 ILE    99       2.643  83.826 -16.733  1.00  0.00           H
ATOM   1206 HD12 ILE    99       1.854  85.245 -16.050  1.00  0.00           H
ATOM   1207 HD13 ILE    99       3.513  85.347 -16.638  1.00  0.00           H
ATOM   1208  N   LYS   100       2.647  83.361 -10.771  1.00119.31       1SG N
ATOM   1209  CA  LYS   100       1.585  82.562 -10.144  1.00119.31       1SG C
ATOM   1210  C   LYS   100       1.865  82.215  -8.672  1.00119.31       1SG C
ATOM   1211  O   LYS   100       0.926  82.223  -7.879  1.00119.31       1SG O
ATOM   1212  CB  LYS   100       1.235  81.309 -10.976  1.00119.31       1SG C
ATOM   1213  CG  LYS   100       0.911  81.574 -12.461  1.00119.31       1SG C
ATOM   1214  CD  LYS   100       0.470  80.340 -13.265  1.00119.31       1SG C
ATOM   1215  CE  LYS   100       1.428  79.142 -13.157  1.00119.31       1SG C
ATOM   1216  NZ  LYS   100       2.823  79.495 -13.456  1.00119.31       1SG N1+
ATOM   1217  H   LYS   100       3.605  83.200 -10.495  1.00  0.00           H
ATOM   1218  HA  LYS   100       0.685  83.180 -10.110  1.00  0.00           H
ATOM   1219  HB2 LYS   100       2.068  80.613 -10.893  1.00  0.00           H
ATOM   1220  HB3 LYS   100       0.387  80.811 -10.505  1.00  0.00           H
ATOM   1221  HG2 LYS   100       0.137  82.340 -12.529  1.00  0.00           H
ATOM   1222  HG3 LYS   100       1.791  81.996 -12.944  1.00  0.00           H
ATOM   1223  HD2 LYS   100      -0.519  80.032 -12.928  1.00  0.00           H
ATOM   1224  HD3 LYS   100       0.356  80.624 -14.312  1.00  0.00           H
ATOM   1225  HE2 LYS   100       1.389  78.718 -12.152  1.00  0.00           H
ATOM   1226  HE3 LYS   100       1.122  78.339 -13.827  1.00  0.00           H
ATOM   1227  HZ1 LYS   100       3.352  78.633 -13.182  1.00  0.00           H
ATOM   1228  HZ2 LYS   100       3.046  79.733 -14.401  1.00  0.00           H
ATOM   1229  HZ3 LYS   100       3.212  80.152 -12.785  1.00  0.00           H
ATOM   1230  N   LEU   101       3.132  81.962  -8.291  1.00172.71       1SG N
ATOM   1231  CA  LEU   101       3.616  81.568  -6.946  1.00172.71       1SG C
ATOM   1232  C   LEU   101       2.940  82.207  -5.744  1.00172.71       1SG C
ATOM   1233  O   LEU   101       2.532  81.476  -4.830  1.00172.71       1SG O
ATOM   1234  CB  LEU   101       5.141  81.646  -6.745  1.00172.71       1SG C
ATOM   1235  CG  LEU   101       5.991  81.692  -8.004  1.00172.71       1SG C
ATOM   1236  CD2 LEU   101       5.832  80.397  -8.824  1.00172.71       1SG C
ATOM   1237  CD1 LEU   101       7.433  82.037  -7.608  1.00172.71       1SG C
ATOM   1238  H   LEU   101       3.826  81.907  -9.021  1.00  0.00           H
ATOM   1239  HA  LEU   101       3.361  80.507  -6.900  1.00  0.00           H
ATOM   1240  HB2 LEU   101       5.387  82.530  -6.154  1.00  0.00           H
ATOM   1241  HB3 LEU   101       5.459  80.810  -6.120  1.00  0.00           H
ATOM   1242  HG  LEU   101       5.644  82.525  -8.602  1.00  0.00           H
ATOM   1243 HD21 LEU   101       6.528  80.335  -9.656  1.00  0.00           H
ATOM   1244 HD22 LEU   101       5.957  79.512  -8.201  1.00  0.00           H
ATOM   1245 HD23 LEU   101       4.844  80.304  -9.269  1.00  0.00           H
ATOM   1246 HD11 LEU   101       8.012  82.365  -8.468  1.00  0.00           H
ATOM   1247 HD12 LEU   101       7.473  82.870  -6.906  1.00  0.00           H
ATOM   1248 HD13 LEU   101       7.941  81.194  -7.139  1.00  0.00           H
ATOM   1249  N   PRO   102       2.817  83.553  -5.715  1.00107.98       1SG N
ATOM   1250  CA  PRO   102       2.184  84.184  -4.583  1.00107.98       1SG C
ATOM   1251  C   PRO   102       0.737  83.826  -4.342  1.00107.98       1SG C
ATOM   1252  O   PRO   102       0.296  83.694  -3.203  1.00107.98       1SG O
ATOM   1253  CD  PRO   102       3.298  84.565  -6.673  1.00107.98       1SG C
ATOM   1254  CB  PRO   102       2.423  85.695  -4.722  1.00107.98       1SG C
ATOM   1255  CG  PRO   102       2.770  85.913  -6.183  1.00107.98       1SG C
ATOM   1256  HA  PRO   102       2.718  83.862  -3.685  1.00  0.00           H
ATOM   1257  HD3 PRO   102       4.388  84.571  -6.673  1.00  0.00           H
ATOM   1258  HD2 PRO   102       2.976  84.422  -7.700  1.00  0.00           H
ATOM   1259  HB2 PRO   102       1.576  86.303  -4.399  1.00  0.00           H
ATOM   1260  HB3 PRO   102       3.273  85.983  -4.101  1.00  0.00           H
ATOM   1261  HG3 PRO   102       3.482  86.726  -6.333  1.00  0.00           H
ATOM   1262  HG2 PRO   102       1.862  86.170  -6.732  1.00  0.00           H
ATOM   1263  N   ILE   103       0.029  83.588  -5.433  1.00 54.03       1SG N
ATOM   1264  CA  ILE   103      -1.383  83.358  -5.399  1.00 54.03       1SG C
```

```
ATOM   1265  C   ILE   103      -1.737  81.931  -5.006  1.00 54.03        1SG C
ATOM   1266  O   ILE   103      -2.822  81.674  -4.490  1.00 54.03        1SG O
ATOM   1267  CB  ILE   103      -1.961  83.747  -6.803  1.00 54.03        1SG C
ATOM   1268  CG2 ILE   103      -3.493  83.561  -6.902  1.00 54.03        1SG C
ATOM   1269  CG1 ILE   103      -1.578  85.210  -7.174  1.00 54.03        1SG C
ATOM   1270  CD1 ILE   103      -1.948  85.648  -8.596  1.00 54.03        1SG C
ATOM   1271  H   ILE   103       0.482  83.583  -6.337  1.00  0.00            H
ATOM   1272  HA  ILE   103      -1.839  84.014  -4.654  1.00  0.00            H
ATOM   1273  HB  ILE   103      -1.514  83.085  -7.545  1.00  0.00            H
ATOM   1274 HG21 ILE   103      -3.881  83.838  -7.881  1.00  0.00            H
ATOM   1275 HG22 ILE   103      -3.788  82.524  -6.749  1.00  0.00            H
ATOM   1276 HG23 ILE   103      -4.011  84.163  -6.155  1.00  0.00            H
ATOM   1277 HG12 ILE   103      -2.028  85.897  -6.456  1.00  0.00            H
ATOM   1278 HG13 ILE   103      -0.503  85.361  -7.086  1.00  0.00            H
ATOM   1279 HD11 ILE   103      -1.534  86.634  -8.810  1.00  0.00            H
ATOM   1280 HD12 ILE   103      -1.551  84.959  -9.342  1.00  0.00            H
ATOM   1281 HD13 ILE   103      -3.026  85.725  -8.724  1.00  0.00            H
ATOM   1282  N   TYR   104      -0.780  81.026  -5.172  1.00163.41        1SG N
ATOM   1283  CA  TYR   104      -0.861  79.706  -4.605  1.00163.41        1SG C
ATOM   1284  C   TYR   104      -0.678  79.743  -3.105  1.00163.41        1SG C
ATOM   1285  O   TYR   104      -1.475  79.191  -2.362  1.00163.41        1SG O
ATOM   1286  CB  TYR   104       0.279  78.832  -5.183  1.00163.41        1SG C
ATOM   1287  CG  TYR   104      -0.015  78.158  -6.503  1.00163.41        1SG C
ATOM   1288  CD1 TYR   104      -0.237  78.919  -7.668  1.00163.41        1SG C
ATOM   1289  CD2 TYR   104      -0.051  76.751  -6.567  1.00163.41        1SG C
ATOM   1290  CE1 TYR   104      -0.514  78.272  -8.883  1.00163.41        1SG C
ATOM   1291  CE2 TYR   104      -0.345  76.104  -7.777  1.00163.41        1SG C
ATOM   1292  CZ  TYR   104      -0.581  76.866  -8.933  1.00163.41        1SG C
ATOM   1293  OH  TYR   104      -0.889  76.246 -10.103  1.00163.41        1SG O
ATOM   1294  H   TYR   104       0.087  81.321  -5.597  1.00  0.00            H
ATOM   1295  HA  TYR   104      -1.825  79.240  -4.823  1.00  0.00            H
ATOM   1296  HB2 TYR   104       1.192  79.409  -5.302  1.00  0.00            H
ATOM   1297  HB3 TYR   104       0.545  78.042  -4.478  1.00  0.00            H
ATOM   1298  HD1 TYR   104      -0.199  79.995  -7.632  1.00  0.00            H
ATOM   1299  HD2 TYR   104       0.126  76.146  -5.687  1.00  0.00            H
ATOM   1300  HE1 TYR   104      -0.672  78.864  -9.768  1.00  0.00            H
ATOM   1301  HE2 TYR   104      -0.384  75.022  -7.788  1.00  0.00            H
ATOM   1302  HH  TYR   104      -0.854  75.286  -9.979  1.00  0.00            H
ATOM   1303  N   THR   105       0.363  80.433  -2.660  1.00 31.97        1SG N
ATOM   1304  CA  THR   105       0.762  80.458  -1.272  1.00 31.97        1SG C
ATOM   1305  C   THR   105      -0.259  81.110  -0.375  1.00 31.97        1SG C
ATOM   1306  O   THR   105      -0.570  80.588   0.696  1.00 31.97        1SG O
ATOM   1307  CB  THR   105       2.120  81.218  -1.231  1.00 31.97        1SG C
ATOM   1308  OG1 THR   105       3.087  80.455  -1.938  1.00 31.97        1SG O
ATOM   1309  CG2 THR   105       2.690  81.497   0.169  1.00 31.97        1SG C
ATOM   1310  H   THR   105       0.950  80.907  -3.331  1.00  0.00            H
ATOM   1311  HA  THR   105       0.913  79.443  -0.912  1.00  0.00            H
ATOM   1312  HB  THR   105       2.023  82.181  -1.736  1.00  0.00            H
ATOM   1313  HG1 THR   105       2.955  80.572  -2.871  1.00  0.00            H
ATOM   1314 HG21 THR   105       3.699  81.907   0.109  1.00  0.00            H
ATOM   1315 HG22 THR   105       2.085  82.226   0.711  1.00  0.00            H
ATOM   1316 HG23 THR   105       2.732  80.599   0.778  1.00  0.00            H
ATOM   1317  N   LEU   106      -0.840  82.194  -0.877  1.00134.16        1SG N
ATOM   1318  CA  LEU   106      -1.954  82.835  -0.240  1.00134.16        1SG C
ATOM   1319  C   LEU   106      -3.191  81.962  -0.153  1.00134.16        1SG C
ATOM   1320  O   LEU   106      -3.923  82.031   0.826  1.00134.16        1SG O
ATOM   1321  CB  LEU   106      -2.299  84.140  -0.996  1.00134.16        1SG C
ATOM   1322  CG  LEU   106      -1.265  85.278  -0.829  1.00134.16        1SG C
ATOM   1323  CD2 LEU   106      -1.321  85.912   0.572  1.00134.16        1SG C
ATOM   1324  CD1 LEU   106      -1.462  86.355  -1.907  1.00134.16        1SG C
ATOM   1325  H   LEU   106      -0.513  82.579  -1.758  1.00  0.00            H
ATOM   1326  HA  LEU   106      -1.675  83.083   0.785  1.00  0.00            H
ATOM   1327  HB2 LEU   106      -2.423  83.906  -2.054  1.00  0.00            H
```

```
ATOM   1328  HB3 LEU   106      -3.272  84.511  -0.668  1.00  0.00           H
ATOM   1329  HG  LEU   106      -0.264  84.868  -0.962  1.00  0.00           H
ATOM   1330 HD21 LEU   106      -0.595  86.721   0.667  1.00  0.00           H
ATOM   1331 HD22 LEU   106      -2.306  86.329   0.784  1.00  0.00           H
ATOM   1332 HD23 LEU   106      -1.094  85.190   1.356  1.00  0.00           H
ATOM   1333 HD11 LEU   106      -0.714  87.143  -1.817  1.00  0.00           H
ATOM   1334 HD12 LEU   106      -1.370  85.934  -2.908  1.00  0.00           H
ATOM   1335 HD13 LEU   106      -2.446  86.819  -1.829  1.00  0.00           H
ATOM   1336  N   ALA   107      -3.389  81.104  -1.149  1.00 33.44       1SG N
ATOM   1337  CA  ALA   107      -4.486  80.176  -1.129  1.00 33.44       1SG C
ATOM   1338  C   ALA   107      -4.269  78.988  -0.215  1.00 33.44       1SG C
ATOM   1339  O   ALA   107      -5.190  78.537   0.462  1.00 33.44       1SG O
ATOM   1340  CB  ALA   107      -4.743  79.653  -2.550  1.00 33.44       1SG C
ATOM   1341  H   ALA   107      -2.721  81.045  -1.904  1.00  0.00           H
ATOM   1342  HA  ALA   107      -5.391  80.690  -0.796  1.00  0.00           H
ATOM   1343  HB1 ALA   107      -5.603  78.987  -2.544  1.00  0.00           H
ATOM   1344  HB2 ALA   107      -4.966  80.469  -3.237  1.00  0.00           H
ATOM   1345  HB3 ALA   107      -3.905  79.089  -2.957  1.00  0.00           H
ATOM   1346  N   GLN   108      -3.034  78.507  -0.197  1.00 55.78       1SG N
ATOM   1347  CA  GLN   108      -2.626  77.385   0.604  1.00 55.78       1SG C
ATOM   1348  C   GLN   108      -2.680  77.698   2.087  1.00 55.78       1SG C
ATOM   1349  O   GLN   108      -3.272  76.955   2.869  1.00 55.78       1SG O
ATOM   1350  CB  GLN   108      -1.192  76.962   0.195  1.00 55.78       1SG C
ATOM   1351  CG  GLN   108      -1.151  76.290  -1.199  1.00 55.78       1SG C
ATOM   1352  CD  GLN   108       0.169  76.362  -1.966  1.00 55.78       1SG C
ATOM   1353  OE1 GLN   108       0.261  75.920  -3.099  1.00 55.78       1SG O
ATOM   1354  NE2 GLN   108       1.222  76.949  -1.423  1.00 55.78       1SG N
ATOM   1355  H   GLN   108      -2.373  78.884  -0.872  1.00  0.00           H
ATOM   1356  HA  GLN   108      -3.307  76.551   0.419  1.00  0.00           H
ATOM   1357  HB2 GLN   108      -0.547  77.839   0.238  1.00  0.00           H
ATOM   1358  HB3 GLN   108      -0.793  76.250   0.920  1.00  0.00           H
ATOM   1359  HG2 GLN   108      -1.460  75.247  -1.134  1.00  0.00           H
ATOM   1360  HG3 GLN   108      -1.878  76.770  -1.846  1.00  0.00           H
ATOM   1361 HE22 GLN   108       2.052  76.998  -1.998  1.00  0.00           H
ATOM   1362 HE21 GLN   108       1.189  77.310  -0.486  1.00  0.00           H
ATOM   1363  N   THR   109      -2.114  78.847   2.453  1.00100.80       1SG N
ATOM   1364  CA  THR   109      -2.191  79.348   3.806  1.00100.80       1SG C
ATOM   1365  C   THR   109      -3.606  79.633   4.282  1.00100.80       1SG C
ATOM   1366  O   THR   109      -3.956  79.323   5.423  1.00100.80       1SG O
ATOM   1367  CB  THR   109      -1.252  80.571   3.987  1.00100.80       1SG C
ATOM   1368  OG1 THR   109      -1.498  81.583   3.031  1.00100.80       1SG O
ATOM   1369  CG2 THR   109       0.242  80.215   3.930  1.00100.80       1SG C
ATOM   1370  H   THR   109      -1.692  79.436   1.746  1.00  0.00           H
ATOM   1371  HA  THR   109      -1.820  78.560   4.466  1.00  0.00           H
ATOM   1372  HB  THR   109      -1.446  81.011   4.968  1.00  0.00           H
ATOM   1373  HG1 THR   109      -1.181  81.282   2.183  1.00  0.00           H
ATOM   1374 HG21 THR   109       0.860  81.107   4.038  1.00  0.00           H
ATOM   1375 HG22 THR   109       0.510  79.544   4.744  1.00  0.00           H
ATOM   1376 HG23 THR   109       0.516  79.728   2.994  1.00  0.00           H
ATOM   1377  N   LEU   110      -4.428  80.148   3.371  1.00 84.77       1SG N
ATOM   1378  CA  LEU   110      -5.808  80.424   3.654  1.00 84.77       1SG C
ATOM   1379  C   LEU   110      -6.663  79.188   3.876  1.00 84.77       1SG C
ATOM   1380  O   LEU   110      -7.556  79.186   4.726  1.00 84.77       1SG O
ATOM   1381  CB  LEU   110      -6.403  81.319   2.539  1.00 84.77       1SG C
ATOM   1382  CG  LEU   110      -7.828  81.880   2.792  1.00 84.77       1SG C
ATOM   1383  CD2 LEU   110      -8.929  81.031   2.134  1.00 84.77       1SG C
ATOM   1384  CD1 LEU   110      -7.942  83.334   2.304  1.00 84.77       1SG C
ATOM   1385  H   LEU   110      -4.053  80.446   2.480  1.00  0.00           H
ATOM   1386  HA  LEU   110      -5.841  81.001   4.582  1.00  0.00           H
ATOM   1387  HB2 LEU   110      -5.737  82.177   2.462  1.00  0.00           H
ATOM   1388  HB3 LEU   110      -6.357  80.817   1.572  1.00  0.00           H
ATOM   1389  HG  LEU   110      -8.010  81.896   3.868  1.00  0.00           H
ATOM   1390 HD21 LEU   110      -9.917  81.444   2.337  1.00  0.00           H
```

ATOM	1391	HD22	LEU	110	-8.804	80.986	1.052	1.00	0.00		H
ATOM	1392	HD23	LEU	110	-8.937	80.007	2.506	1.00	0.00		H
ATOM	1393	HD11	LEU	110	-8.935	83.739	2.498	1.00	0.00		H
ATOM	1394	HD12	LEU	110	-7.226	83.979	2.814	1.00	0.00		H
ATOM	1395	HD13	LEU	110	-7.752	83.409	1.232	1.00	0.00		H
ATOM	1396	N	GLY	111	-6.335	78.111	3.172	1.00	20.00	1SG	N
ATOM	1397	CA	GLY	111	-7.063	76.877	3.313	1.00	20.00	1SG	C
ATOM	1398	C	GLY	111	-6.731	76.151	4.595	1.00	20.00	1SG	C
ATOM	1399	O	GLY	111	-7.609	75.593	5.251	1.00	20.00	1SG	O
ATOM	1400	H	GLY	111	-5.622	78.188	2.455	1.00	0.00		H
ATOM	1401	HA2	GLY	111	-8.139	77.049	3.251	1.00	0.00		H
ATOM	1402	HA3	GLY	111	-6.803	76.226	2.478	1.00	0.00		H
ATOM	1403	N	ALA	112	-5.467	76.253	4.992	1.00	30.96	1SG	N
ATOM	1404	CA	ALA	112	-4.988	75.722	6.244	1.00	30.96	1SG	C
ATOM	1405	C	ALA	112	-5.537	76.436	7.458	1.00	30.96	1SG	C
ATOM	1406	O	ALA	112	-5.817	75.815	8.479	1.00	30.96	1SG	O
ATOM	1407	CB	ALA	112	-3.461	75.772	6.200	1.00	30.96	1SG	C
ATOM	1408	H	ALA	112	-4.811	76.737	4.392	1.00	0.00		H
ATOM	1409	HA	ALA	112	-5.287	74.674	6.292	1.00	0.00		H
ATOM	1410	HB1	ALA	112	-3.043	75.096	6.946	1.00	0.00		H
ATOM	1411	HB2	ALA	112	-3.072	75.434	5.241	1.00	0.00		H
ATOM	1412	HB3	ALA	112	-3.075	76.777	6.370	1.00	0.00		H
ATOM	1413	N	PHE	113	-5.748	77.737	7.305	1.00	46.07	1SG	N
ATOM	1414	CA	PHE	113	-6.415	78.540	8.294	1.00	46.07	1SG	C
ATOM	1415	C	PHE	113	-7.856	78.130	8.534	1.00	46.07	1SG	C
ATOM	1416	O	PHE	113	-8.260	77.849	9.661	1.00	46.07	1SG	O
ATOM	1417	CB	PHE	113	-6.299	80.031	7.888	1.00	46.07	1SG	C
ATOM	1418	CG	PHE	113	-6.945	81.041	8.824	1.00	46.07	1SG	C
ATOM	1419	CD1	PHE	113	-6.282	81.453	10.000	1.00	46.07	1SG	C
ATOM	1420	CD2	PHE	113	-8.218	81.572	8.522	1.00	46.07	1SG	C
ATOM	1421	CE1	PHE	113	-6.891	82.378	10.870	1.00	46.07	1SG	C
ATOM	1422	CE2	PHE	113	-8.824	82.500	9.390	1.00	46.07	1SG	C
ATOM	1423	CZ	PHE	113	-8.161	82.904	10.565	1.00	46.07	1SG	C
ATOM	1424	H	PHE	113	-5.460	78.173	6.438	1.00	0.00		H
ATOM	1425	HA	PHE	113	-5.889	78.415	9.243	1.00	0.00		H
ATOM	1426	HB2	PHE	113	-5.244	80.296	7.804	1.00	0.00		H
ATOM	1427	HB3	PHE	113	-6.713	80.186	6.893	1.00	0.00		H
ATOM	1428	HD1	PHE	113	-5.310	81.058	10.250	1.00	0.00		H
ATOM	1429	HD2	PHE	113	-8.741	81.262	7.628	1.00	0.00		H
ATOM	1430	HE1	PHE	113	-6.393	82.676	11.781	1.00	0.00		H
ATOM	1431	HE2	PHE	113	-9.802	82.899	9.162	1.00	0.00		H
ATOM	1432	HZ	PHE	113	-8.629	83.607	11.240	1.00	0.00		H
ATOM	1433	N	LEU	114	-8.612	78.047	7.444	1.00123.44		1SG	N
ATOM	1434	CA	LEU	114	-10.020	77.734	7.508	1.00123.44		1SG	C
ATOM	1435	C	LEU	114	-10.300	76.302	7.910	1.00123.44		1SG	C
ATOM	1436	O	LEU	114	-11.301	76.031	8.564	1.00123.44		1SG	O
ATOM	1437	CB	LEU	114	-10.617	78.056	6.115	1.00123.44		1SG	C
ATOM	1438	CG	LEU	114	-12.136	77.825	5.927	1.00123.44		1SG	C
ATOM	1439	CD2	LEU	114	-12.544	78.099	4.471	1.00123.44		1SG	C
ATOM	1440	CD1	LEU	114	-12.985	78.671	6.890	1.00123.44		1SG	C
ATOM	1441	H	LEU	114	-8.207	78.281	6.546	1.00	0.00		H
ATOM	1442	HA	LEU	114	-10.481	78.386	8.253	1.00	0.00		H
ATOM	1443	HB2	LEU	114	-10.402	79.100	5.882	1.00	0.00		H
ATOM	1444	HB3	LEU	114	-10.075	77.481	5.364	1.00	0.00		H
ATOM	1445	HG	LEU	114	-12.364	76.776	6.124	1.00	0.00		H
ATOM	1446	HD21	LEU	114	-13.602	77.891	4.314	1.00	0.00		H
ATOM	1447	HD22	LEU	114	-12.364	79.140	4.197	1.00	0.00		H
ATOM	1448	HD23	LEU	114	-11.982	77.472	3.779	1.00	0.00		H
ATOM	1449	HD11	LEU	114	-14.050	78.519	6.712	1.00	0.00		H
ATOM	1450	HD12	LEU	114	-12.801	78.401	7.930	1.00	0.00		H
ATOM	1451	HD13	LEU	114	-12.779	79.736	6.779	1.00	0.00		H
ATOM	1452	N	GLY	115	-9.376	75.401	7.603	1.00	21.45	1SG	N
ATOM	1453	CA	GLY	115	-9.566	74.020	7.945	1.00	21.45	1SG	C

ATOM	1454	C	GLY	115	-9.221	73.705	9.380	1.00	21.45	1SG	C
ATOM	1455	O	GLY	115	-9.850	72.838	9.980	1.00	21.45	1SG	O
ATOM	1456	H	GLY	115	-8.586	75.666	7.026	1.00	0.00		H
ATOM	1457	HA2	GLY	115	-10.585	73.692	7.728	1.00	0.00		H
ATOM	1458	HA3	GLY	115	-8.919	73.424	7.310	1.00	0.00		H
ATOM	1459	N	ALA	116	-8.304	74.486	9.953	1.00	31.80	1SG	N
ATOM	1460	CA	ALA	116	-8.083	74.496	11.380	1.00	31.80	1SG	C
ATOM	1461	C	ALA	116	-9.271	75.032	12.146	1.00	31.80	1SG	C
ATOM	1462	O	ALA	116	-9.690	74.451	13.143	1.00	31.80	1SG	O
ATOM	1463	CB	ALA	116	-6.848	75.348	11.702	1.00	31.80	1SG	C
ATOM	1464	H	ALA	116	-7.840	75.191	9.398	1.00	0.00		H
ATOM	1465	HA	ALA	116	-7.899	73.474	11.716	1.00	0.00		H
ATOM	1466	HB1	ALA	116	-6.680	75.406	12.779	1.00	0.00		H
ATOM	1467	HB2	ALA	116	-5.962	74.903	11.259	1.00	0.00		H
ATOM	1468	HB3	ALA	116	-6.938	76.368	11.332	1.00	0.00		H
ATOM	1469	N	GLY	117	-9.863	76.090	11.602	1.00	18.60	1SG	N
ATOM	1470	CA	GLY	117	-10.986	76.729	12.228	1.00	18.60	1SG	C
ATOM	1471	C	GLY	117	-12.265	75.921	12.183	1.00	18.60	1SG	C
ATOM	1472	O	GLY	117	-13.069	75.967	13.109	1.00	18.60	1SG	O
ATOM	1473	H	GLY	117	-9.427	76.545	10.809	1.00	0.00		H
ATOM	1474	HA2	GLY	117	-10.749	76.957	13.268	1.00	0.00		H
ATOM	1475	HA3	GLY	117	-11.172	77.674	11.722	1.00	0.00		H
ATOM	1476	N	ILE	118	-12.406	75.123	11.130	1.00	78.73	1SG	N
ATOM	1477	CA	ILE	118	-13.477	74.170	11.004	1.00	78.73	1SG	C
ATOM	1478	C	ILE	118	-13.337	72.988	11.922	1.00	78.73	1SG	C
ATOM	1479	O	ILE	118	-14.305	72.613	12.579	1.00	78.73	1SG	O
ATOM	1480	CB	ILE	118	-13.596	73.740	9.502	1.00	78.73	1SG	C
ATOM	1481	CG2	ILE	118	-14.322	72.401	9.228	1.00	78.73	1SG	C
ATOM	1482	CG1	ILE	118	-14.211	74.869	8.633	1.00	78.73	1SG	C
ATOM	1483	CD1	ILE	118	-15.736	75.034	8.750	1.00	78.73	1SG	C
ATOM	1484	H	ILE	118	-11.737	75.202	10.374	1.00	0.00		H
ATOM	1485	HA	ILE	118	-14.409	74.661	11.291	1.00	0.00		H
ATOM	1486	HB	ILE	118	-12.578	73.588	9.140	1.00	0.00		H
ATOM	1487	HG21	ILE	118	-14.448	72.230	8.159	1.00	0.00		H
ATOM	1488	HG22	ILE	118	-13.761	71.550	9.616	1.00	0.00		H
ATOM	1489	HG23	ILE	118	-15.310	72.377	9.690	1.00	0.00		H
ATOM	1490	HG12	ILE	118	-13.754	75.825	8.885	1.00	0.00		H
ATOM	1491	HG13	ILE	118	-13.958	74.691	7.587	1.00	0.00		H
ATOM	1492	HD11	ILE	118	-16.076	75.881	8.154	1.00	0.00		H
ATOM	1493	HD12	ILE	118	-16.270	74.154	8.393	1.00	0.00		H
ATOM	1494	HD13	ILE	118	-16.046	75.217	9.779	1.00	0.00		H
ATOM	1495	N	VAL	119	-12.134	72.428	11.989	1.00	43.20	1SG	N
ATOM	1496	CA	VAL	119	-11.868	71.355	12.911	1.00	43.20	1SG	C
ATOM	1497	C	VAL	119	-12.064	71.760	14.366	1.00	43.20	1SG	C
ATOM	1498	O	VAL	119	-12.647	71.023	15.158	1.00	43.20	1SG	O
ATOM	1499	CB	VAL	119	-10.465	70.751	12.630	1.00	43.20	1SG	C
ATOM	1500	CG1	VAL	119	-9.891	69.880	13.766	1.00	43.20	1SG	C
ATOM	1501	CG2	VAL	119	-10.505	69.912	11.342	1.00	43.20	1SG	C
ATOM	1502	H	VAL	119	-11.375	72.777	11.416	1.00	0.00		H
ATOM	1503	HA	VAL	119	-12.608	70.570	12.732	1.00	0.00		H
ATOM	1504	HB	VAL	119	-9.760	71.571	12.484	1.00	0.00		H
ATOM	1505	HG11	VAL	119	-8.933	69.457	13.477	1.00	0.00		H
ATOM	1506	HG12	VAL	119	-9.707	70.448	14.679	1.00	0.00		H
ATOM	1507	HG13	VAL	119	-10.557	69.053	14.011	1.00	0.00		H
ATOM	1508	HG21	VAL	119	-9.528	69.483	11.129	1.00	0.00		H
ATOM	1509	HG22	VAL	119	-11.206	69.082	11.430	1.00	0.00		H
ATOM	1510	HG23	VAL	119	-10.805	70.508	10.482	1.00	0.00		H
ATOM	1511	N	PHE	120	-11.661	72.987	14.673	1.00	57.94	1SG	N
ATOM	1512	CA	PHE	120	-11.885	73.583	15.964	1.00	57.94	1SG	C
ATOM	1513	C	PHE	120	-13.338	73.803	16.325	1.00	57.94	1SG	C
ATOM	1514	O	PHE	120	-13.697	73.797	17.496	1.00	57.94	1SG	O
ATOM	1515	CB	PHE	120	-11.165	74.956	15.996	1.00	57.94	1SG	C
ATOM	1516	CG	PHE	120	-11.086	75.669	17.340	1.00	57.94	1SG	C

```
ATOM   1517  CD1 PHE   120      -10.455  75.054  18.443  1.00 57.94        1SG C
ATOM   1518  CD2 PHE   120      -11.638  76.959  17.488  1.00 57.94        1SG C
ATOM   1519  CE1 PHE   120      -10.360  75.732  19.674  1.00 57.94        1SG C
ATOM   1520  CE2 PHE   120      -11.540  77.637  18.718  1.00 57.94        1SG C
ATOM   1521  CZ  PHE   120      -10.902  77.023  19.813  1.00 57.94        1SG C
ATOM   1522  H   PHE   120      -11.158  73.528  13.977  1.00  0.00            H
ATOM   1523  HA  PHE   120      -11.448  72.928  16.721  1.00  0.00            H
ATOM   1524  HB2 PHE   120      -10.140  74.861  15.647  1.00  0.00            H
ATOM   1525  HB3 PHE   120      -11.638  75.627  15.279  1.00  0.00            H
ATOM   1526  HD1 PHE   120      -10.051  74.056  18.358  1.00  0.00            H
ATOM   1527  HD2 PHE   120      -12.149  77.433  16.662  1.00  0.00            H
ATOM   1528  HE1 PHE   120       -9.882  75.257  20.519  1.00  0.00            H
ATOM   1529  HE2 PHE   120      -11.968  78.623  18.831  1.00  0.00            H
ATOM   1530  HZ  PHE   120      -10.842  77.534  20.763  1.00  0.00            H
ATOM   1531  N   GLY   121      -14.177  73.928  15.308  1.00 23.31        1SG N
ATOM   1532  CA  GLY   121      -15.563  74.241  15.494  1.00 23.31        1SG C
ATOM   1533  C   GLY   121      -16.458  73.028  15.553  1.00 23.31        1SG C
ATOM   1534  O   GLY   121      -17.662  73.155  15.755  1.00 23.31        1SG O
ATOM   1535  H   GLY   121      -13.826  73.876  14.361  1.00  0.00            H
ATOM   1536  HA2 GLY   121      -15.730  74.862  16.376  1.00  0.00            H
ATOM   1537  HA3 GLY   121      -15.882  74.843  14.643  1.00  0.00            H
ATOM   1538  N   LEU   122      -15.846  71.860  15.401  1.00 53.46        1SG N
ATOM   1539  CA  LEU   122      -16.498  70.581  15.459  1.00 53.46        1SG C
ATOM   1540  C   LEU   122      -15.932  69.679  16.567  1.00 53.46        1SG C
ATOM   1541  O   LEU   122      -16.430  68.575  16.760  1.00 53.46        1SG O
ATOM   1542  CB  LEU   122      -16.347  69.882  14.083  1.00 53.46        1SG C
ATOM   1543  CG  LEU   122      -17.025  70.598  12.891  1.00 53.46        1SG C
ATOM   1544  CD2 LEU   122      -18.559  70.552  12.982  1.00 53.46        1SG C
ATOM   1545  CD1 LEU   122      -16.544  70.005  11.557  1.00 53.46        1SG C
ATOM   1546  H   LEU   122      -14.857  71.871  15.189  1.00  0.00            H
ATOM   1547  HA  LEU   122      -17.559  70.684  15.689  1.00  0.00            H
ATOM   1548  HB2 LEU   122      -15.281  69.772  13.876  1.00  0.00            H
ATOM   1549  HB3 LEU   122      -16.736  68.864  14.146  1.00  0.00            H
ATOM   1550  HG  LEU   122      -16.728  71.646  12.894  1.00  0.00            H
ATOM   1551 HD21 LEU   122      -19.020  71.038  12.122  1.00  0.00            H
ATOM   1552 HD22 LEU   122      -18.927  69.527  13.025  1.00  0.00            H
ATOM   1553 HD23 LEU   122      -18.920  71.072  13.871  1.00  0.00            H
ATOM   1554 HD11 LEU   122      -16.995  70.524  10.711  1.00  0.00            H
ATOM   1555 HD12 LEU   122      -15.462  70.100  11.459  1.00  0.00            H
ATOM   1556 HD13 LEU   122      -16.798  68.948  11.479  1.00  0.00            H
ATOM   1557  N   TYR   123      -14.917  70.153  17.293  1.00106.63        1SG N
ATOM   1558  CA  TYR   123      -14.435  69.588  18.549  1.00106.63        1SG C
ATOM   1559  C   TYR   123      -14.396  70.716  19.600  1.00106.63        1SG C
ATOM   1560  O   TYR   123      -13.591  70.668  20.529  1.00106.63        1SG O
ATOM   1561  CB  TYR   123      -13.035  68.954  18.373  1.00106.63        1SG C
ATOM   1562  CG  TYR   123      -12.866  67.704  17.522  1.00106.63        1SG C
ATOM   1563  CD1 TYR   123      -12.210  67.782  16.277  1.00106.63        1SG C
ATOM   1564  CD2 TYR   123      -13.246  66.442  18.026  1.00106.63        1SG C
ATOM   1565  CE1 TYR   123      -11.890  66.610  15.566  1.00106.63        1SG C
ATOM   1566  CE2 TYR   123      -12.922  65.266  17.317  1.00106.63        1SG C
ATOM   1567  CZ  TYR   123      -12.218  65.349  16.100  1.00106.63        1SG C
ATOM   1568  OH  TYR   123      -11.820  64.213  15.456  1.00106.63        1SG O
ATOM   1569  H   TYR   123      -14.538  71.051  17.034  1.00  0.00            H
ATOM   1570  HA  TYR   123      -15.127  68.832  18.930  1.00  0.00            H
ATOM   1571  HB2 TYR   123      -12.328  69.715  18.041  1.00  0.00            H
ATOM   1572  HB3 TYR   123      -12.691  68.648  19.357  1.00  0.00            H
ATOM   1573  HD1 TYR   123      -11.918  68.744  15.887  1.00  0.00            H
ATOM   1574  HD2 TYR   123      -13.757  66.370  18.976  1.00  0.00            H
ATOM   1575  HE1 TYR   123      -11.370  66.687  14.625  1.00  0.00            H
ATOM   1576  HE2 TYR   123      -13.189  64.304  17.730  1.00  0.00            H
ATOM   1577  HH  TYR   123      -11.012  64.356  14.964  1.00  0.00            H
ATOM   1578  N   TYR   124      -15.209  71.775  19.433  1.00 94.97        1SG N
ATOM   1579  CA  TYR   124      -15.178  72.986  20.267  1.00 94.97        1SG C
```

ATOM	1580	C	TYR	124	-15.605	72.696	21.706	1.00	94.97	1SG	C
ATOM	1581	O	TYR	124	-15.105	73.285	22.663	1.00	94.97	1SG	O
ATOM	1582	CB	TYR	124	-16.106	74.069	19.668	1.00	94.97	1SG	C
ATOM	1583	CG	TYR	124	-15.943	75.430	20.325	1.00	94.97	1SG	C
ATOM	1584	CD1	TYR	124	-14.776	76.187	20.086	1.00	94.97	1SG	C
ATOM	1585	CD2	TYR	124	-16.908	75.902	21.241	1.00	94.97	1SG	C
ATOM	1586	CE1	TYR	124	-14.559	77.386	20.789	1.00	94.97	1SG	C
ATOM	1587	CE2	TYR	124	-16.688	77.102	21.944	1.00	94.97	1SG	C
ATOM	1588	CZ	TYR	124	-15.505	77.835	21.729	1.00	94.97	1SG	C
ATOM	1589	OH	TYR	124	-15.242	78.955	22.459	1.00	94.97	1SG	O
ATOM	1590	H	TYR	124	-15.889	71.718	18.692	1.00	0.00		H
ATOM	1591	HA	TYR	124	-14.151	73.357	20.282	1.00	0.00		H
ATOM	1592	HB2	TYR	124	-15.912	74.191	18.606	1.00	0.00		H
ATOM	1593	HB3	TYR	124	-17.149	73.754	19.738	1.00	0.00		H
ATOM	1594	HD1	TYR	124	-14.024	75.827	19.398	1.00	0.00		H
ATOM	1595	HD2	TYR	124	-17.796	75.322	21.445	1.00	0.00		H
ATOM	1596	HE1	TYR	124	-13.649	77.944	20.632	1.00	0.00		H
ATOM	1597	HE2	TYR	124	-17.418	77.428	22.670	1.00	0.00		H
ATOM	1598	HH	TYR	124	-15.922	79.166	23.084	1.00	0.00		H
ATOM	1599	N	ASP	125	-16.505	71.728	21.841	1.00	58.26	1SG	N
ATOM	1600	CA	ASP	125	-16.979	71.159	23.080	1.00	58.26	1SG	C
ATOM	1601	C	ASP	125	-15.842	70.470	23.838	1.00	58.26	1SG	C
ATOM	1602	O	ASP	125	-15.643	70.695	25.036	1.00	58.26	1SG	O
ATOM	1603	CB	ASP	125	-18.131	70.155	22.773	1.00	58.26	1SG	C
ATOM	1604	CG	ASP	125	-17.759	68.943	21.908	1.00	58.26	1SG	C
ATOM	1605	OD1	ASP	125	-16.898	69.131	21.015	1.00	58.26	1SG	O
ATOM	1606	OD2	ASP	125	-18.264	67.846	22.209	1.00	58.26	1SG	O1-
ATOM	1607	H	ASP	125	-16.693	71.142	21.030	1.00	0.00		H
ATOM	1608	HA	ASP	125	-17.368	71.965	23.703	1.00	0.00		H
ATOM	1609	HB2	ASP	125	-18.539	69.779	23.712	1.00	0.00		H
ATOM	1610	HB3	ASP	125	-18.952	70.670	22.277	1.00	0.00		H
ATOM	1611	N	ALA	126	-15.088	69.656	23.107	1.00	39.72	1SG	N
ATOM	1612	CA	ALA	126	-14.112	68.734	23.629	1.00	39.72	1SG	C
ATOM	1613	C	ALA	126	-12.763	69.353	23.919	1.00	39.72	1SG	C
ATOM	1614	O	ALA	126	-11.944	68.745	24.606	1.00	39.72	1SG	O
ATOM	1615	CB	ALA	126	-13.927	67.624	22.581	1.00	39.72	1SG	C
ATOM	1616	H	ALA	126	-15.458	69.478	22.168	1.00	0.00		H
ATOM	1617	HA	ALA	126	-14.493	68.292	24.552	1.00	0.00		H
ATOM	1618	HB1	ALA	126	-13.284	66.829	22.956	1.00	0.00		H
ATOM	1619	HB2	ALA	126	-14.887	67.168	22.330	1.00	0.00		H
ATOM	1620	HB3	ALA	126	-13.499	68.006	21.654	1.00	0.00		H
ATOM	1621	N	ILE	127	-12.564	70.576	23.437	1.00100.89		1SG	N
ATOM	1622	CA	ILE	127	-11.519	71.457	23.893	1.00100.89		1SG	C
ATOM	1623	C	ILE	127	-11.761	71.866	25.339	1.00100.89		1SG	C
ATOM	1624	O	ILE	127	-10.927	71.635	26.220	1.00100.89		1SG	O
ATOM	1625	CB	ILE	127	-11.467	72.733	22.968	1.00100.89		1SG	C
ATOM	1626	CG2	ILE	127	-10.776	73.988	23.562	1.00100.89		1SG	C
ATOM	1627	CG1	ILE	127	-10.895	72.446	21.565	1.00100.89		1SG	C
ATOM	1628	CD1	ILE	127	-9.360	72.346	21.503	1.00100.89		1SG	C
ATOM	1629	H	ILE	127	-13.276	70.937	22.815	1.00	0.00		H
ATOM	1630	HA	ILE	127	-10.559	70.942	23.856	1.00	0.00		H
ATOM	1631	HB	ILE	127	-12.501	73.038	22.801	1.00	0.00		H
ATOM	1632	HG21	ILE	127	-10.686	74.777	22.816	1.00	0.00		H
ATOM	1633	HG22	ILE	127	-11.342	74.424	24.386	1.00	0.00		H
ATOM	1634	HG23	ILE	127	-9.778	73.762	23.933	1.00	0.00		H
ATOM	1635	HG12	ILE	127	-11.341	71.537	21.175	1.00	0.00		H
ATOM	1636	HG13	ILE	127	-11.224	73.231	20.883	1.00	0.00		H
ATOM	1637	HD11	ILE	127	-9.031	72.060	20.505	1.00	0.00		H
ATOM	1638	HD12	ILE	127	-8.878	73.296	21.734	1.00	0.00		H
ATOM	1639	HD13	ILE	127	-8.981	71.604	22.204	1.00	0.00		H
ATOM	1640	N	TRP	128	-12.906	72.521	25.545	1.00	76.87	1SG	N
ATOM	1641	CA	TRP	128	-13.186	73.220	26.778	1.00	76.87	1SG	C
ATOM	1642	C	TRP	128	-13.361	72.272	27.949	1.00	76.87	1SG	C

ATOM	1643	O	TRP	128	-12.741	72.420	28.999	1.00	76.87	1SG	O
ATOM	1644	CB	TRP	128	-14.389	74.166	26.606	1.00	76.87	1SG	C
ATOM	1645	CG	TRP	128	-14.021	75.455	25.932	1.00	76.87	1SG	C
ATOM	1646	CD2	TRP	128	-13.079	76.474	26.394	1.00	76.87	1SG	C
ATOM	1647	CD1	TRP	128	-14.473	75.888	24.733	1.00	76.87	1SG	C
ATOM	1648	NE1	TRP	128	-13.870	77.091	24.418	1.00	76.87	1SG	N
ATOM	1649	CE2	TRP	128	-12.984	77.491	25.396	1.00	76.87	1SG	C
ATOM	1650	CE3	TRP	128	-12.285	76.640	27.556	1.00	76.87	1SG	C
ATOM	1651	CZ2	TRP	128	-12.129	78.599	25.530	1.00	76.87	1SG	C
ATOM	1652	CZ3	TRP	128	-11.429	77.749	27.705	1.00	76.87	1SG	C
ATOM	1653	CH2	TRP	128	-11.344	78.725	26.692	1.00	76.87	1SG	C
ATOM	1654	H	TRP	128	-13.530	72.662	24.762	1.00	0.00		H
ATOM	1655	HA	TRP	128	-12.312	73.825	27.026	1.00	0.00		H
ATOM	1656	HB2	TRP	128	-15.199	73.674	26.065	1.00	0.00		H
ATOM	1657	HB3	TRP	128	-14.794	74.435	27.583	1.00	0.00		H
ATOM	1658	HD1	TRP	128	-15.182	75.354	24.117	1.00	0.00		H
ATOM	1659	HE1	TRP	128	-14.077	77.601	23.562	1.00	0.00		H
ATOM	1660	HE3	TRP	128	-12.337	75.907	28.352	1.00	0.00		H
ATOM	1661	HZ2	TRP	128	-12.076	79.348	24.753	1.00	0.00		H
ATOM	1662	HZ3	TRP	128	-10.846	77.845	28.610	1.00	0.00		H
ATOM	1663	HH2	TRP	128	-10.689	79.575	26.809	1.00	0.00		H
ATOM	1664	N	HIS	129	-14.063	71.178	27.695	1.00	82.35	1SG	N
ATOM	1665	CA	HIS	129	-14.198	70.107	28.652	1.00	82.35	1SG	C
ATOM	1666	C	HIS	129	-13.125	69.038	28.439	1.00	82.35	1SG	C
ATOM	1667	O	HIS	129	-13.359	67.824	28.404	1.00	82.35	1SG	O
ATOM	1668	ND1	HIS	129	-17.186	71.033	27.289	1.00	82.35	1SG	N
ATOM	1669	CG	HIS	129	-16.780	70.371	28.435	1.00	82.35	1SG	C
ATOM	1670	CB	HIS	129	-15.588	69.442	28.459	1.00	82.35	1SG	C
ATOM	1671	NE2	HIS	129	-18.605	71.642	28.877	1.00	82.35	1SG	N
ATOM	1672	CD2	HIS	129	-17.662	70.763	29.417	1.00	82.35	1SG	C
ATOM	1673	CE1	HIS	129	-18.257	71.764	27.603	1.00	82.35	1SG	C
ATOM	1674	H	HIS	129	-14.487	71.074	26.781	1.00	0.00		H
ATOM	1675	HA	HIS	129	-14.137	70.478	29.678	1.00	0.00		H
ATOM	1676	HD1	HIS	129	-16.743	70.964	26.369	1.00	0.00		H
ATOM	1677	HB2	HIS	129	-15.615	68.865	27.533	1.00	0.00		H
ATOM	1678	HB3	HIS	129	-15.753	68.724	29.263	1.00	0.00		H
ATOM	1679	HD2	HIS	129	-17.696	70.485	30.461	1.00	0.00		H
ATOM	1680	HE1	HIS	129	-18.789	72.387	26.897	1.00	0.00		H
ATOM	1681	N	PHE	130	-11.910	69.530	28.320	1.00	54.37	1SG	N
ATOM	1682	CA	PHE	130	-10.691	68.791	28.479	1.00	54.37	1SG	C
ATOM	1683	C	PHE	130	-9.710	69.643	29.241	1.00	54.37	1SG	C
ATOM	1684	O	PHE	130	-9.056	69.165	30.169	1.00	54.37	1SG	O
ATOM	1685	CB	PHE	130	-10.101	68.323	27.127	1.00	54.37	1SG	C
ATOM	1686	CG	PHE	130	-8.767	67.592	27.230	1.00	54.37	1SG	C
ATOM	1687	CD1	PHE	130	-8.709	66.250	27.665	1.00	54.37	1SG	C
ATOM	1688	CD2	PHE	130	-7.568	68.273	26.924	1.00	54.37	1SG	C
ATOM	1689	CE1	PHE	130	-7.469	65.592	27.782	1.00	54.37	1SG	C
ATOM	1690	CE2	PHE	130	-6.327	67.621	27.059	1.00	54.37	1SG	C
ATOM	1691	CZ	PHE	130	-6.277	66.278	27.481	1.00	54.37	1SG	C
ATOM	1692	H	PHE	130	-11.843	70.541	28.366	1.00	0.00		H
ATOM	1693	HA	PHE	130	-10.877	67.908	29.095	1.00	0.00		H
ATOM	1694	HB2	PHE	130	-10.803	67.679	26.607	1.00	0.00		H
ATOM	1695	HB3	PHE	130	-9.969	69.181	26.467	1.00	0.00		H
ATOM	1696	HD1	PHE	130	-9.609	65.717	27.922	1.00	0.00		H
ATOM	1697	HD2	PHE	130	-7.591	69.301	26.592	1.00	0.00		H
ATOM	1698	HE1	PHE	130	-7.428	64.563	28.108	1.00	0.00		H
ATOM	1699	HE2	PHE	130	-5.414	68.144	26.816	1.00	0.00		H
ATOM	1700	HZ	PHE	130	-5.323	65.777	27.566	1.00	0.00		H
ATOM	1701	N	ALA	131	-9.671	70.926	28.871	1.00	37.09	1SG	N
ATOM	1702	CA	ALA	131	-9.057	71.987	29.629	1.00	37.09	1SG	C
ATOM	1703	C	ALA	131	-9.407	71.927	31.094	1.00	37.09	1SG	C
ATOM	1704	O	ALA	131	-8.596	71.530	31.930	1.00	37.09	1SG	O
ATOM	1705	CB	ALA	131	-9.405	73.343	28.997	1.00	37.09	1SG	C

```
ATOM   1706  H   ALA   131     -10.167  71.201  28.028  1.00  0.00           H
ATOM   1707  HA  ALA   131      -7.979  71.852  29.554  1.00  0.00           H
ATOM   1708  HB1 ALA   131      -9.066  74.152  29.640  1.00  0.00           H
ATOM   1709  HB2 ALA   131      -8.941  73.464  28.019  1.00  0.00           H
ATOM   1710  HB3 ALA   131     -10.477  73.487  28.876  1.00  0.00           H
ATOM   1711  N   ASP   132     -10.687  72.122  31.356  1.00 31.16        1SG N
ATOM   1712  CA  ASP   132     -11.257  72.079  32.680  1.00 31.16        1SG C
ATOM   1713  C   ASP   132     -11.705  70.667  33.040  1.00 31.16        1SG C
ATOM   1714  O   ASP   132     -12.791  70.402  33.555  1.00 31.16        1SG O
ATOM   1715  CB  ASP   132     -12.401  73.114  32.764  1.00 31.16        1SG C
ATOM   1716  CG  ASP   132     -11.860  74.524  32.527  1.00 31.16        1SG C
ATOM   1717  OD1 ASP   132     -11.328  75.093  33.503  1.00 31.16        1SG O
ATOM   1718  OD2 ASP   132     -11.856  74.942  31.348  1.00 31.16        1SG O1-
ATOM   1719  H   ASP   132     -11.277  72.523  30.630  1.00  0.00           H
ATOM   1720  HA  ASP   132     -10.510  72.376  33.420  1.00  0.00           H
ATOM   1721  HB2 ASP   132     -13.183  72.902  32.034  1.00  0.00           H
ATOM   1722  HB3 ASP   132     -12.870  73.094  33.748  1.00  0.00           H
ATOM   1723  N   ASN   133     -10.809  69.722  32.768  1.00 64.61        1SG N
ATOM   1724  CA  ASN   133     -10.705  68.449  33.460  1.00 64.61        1SG C
ATOM   1725  C   ASN   133      -9.344  68.360  34.181  1.00 64.61        1SG C
ATOM   1726  O   ASN   133      -9.147  67.426  34.953  1.00 64.61        1SG O
ATOM   1727  CB  ASN   133     -10.858  67.261  32.472  1.00 64.61        1SG C
ATOM   1728  CG  ASN   133     -12.226  67.073  31.798  1.00 64.61        1SG C
ATOM   1729  OD1 ASN   133     -12.387  66.160  30.988  1.00 64.61        1SG O
ATOM   1730  ND2 ASN   133     -13.233  67.891  32.088  1.00 64.61        1SG N
ATOM   1731  H   ASN   133     -10.013  70.022  32.218  1.00  0.00           H
ATOM   1732  HA  ASN   133     -11.465  68.363  34.240  1.00  0.00           H
ATOM   1733  HB2 ASN   133     -10.099  67.324  31.692  1.00  0.00           H
ATOM   1734  HB3 ASN   133     -10.649  66.331  33.003  1.00  0.00           H
ATOM   1735 HD22 ASN   133     -14.130  67.747  31.656  1.00  0.00           H
ATOM   1736 HD21 ASN   133     -13.098  68.690  32.711  1.00  0.00           H
ATOM   1737  N   GLN   134      -8.405  69.296  33.940  1.00222.19        1SG N
ATOM   1738  CA  GLN   134      -7.046  69.294  34.501  1.00222.19        1SG C
ATOM   1739  C   GLN   134      -6.391  70.690  34.627  1.00222.19        1SG C
ATOM   1740  O   GLN   134      -5.310  70.794  35.198  1.00222.19        1SG O
ATOM   1741  CB  GLN   134      -6.172  68.267  33.751  1.00222.19        1SG C
ATOM   1742  CG  GLN   134      -5.928  68.636  32.274  1.00222.19        1SG C
ATOM   1743  CD  GLN   134      -5.983  67.427  31.347  1.00222.19        1SG C
ATOM   1744  OE1 GLN   134      -5.209  66.486  31.476  1.00222.19        1SG O
ATOM   1745  NE2 GLN   134      -6.896  67.441  30.388  1.00222.19        1SG N
ATOM   1746  H   GLN   134      -8.620  70.028  33.279  1.00  0.00           H
ATOM   1747  HA  GLN   134      -7.127  68.949  35.534  1.00  0.00           H
ATOM   1748  HB2 GLN   134      -5.206  68.175  34.251  1.00  0.00           H
ATOM   1749  HB3 GLN   134      -6.626  67.278  33.835  1.00  0.00           H
ATOM   1750  HG2 GLN   134      -6.642  69.383  31.926  1.00  0.00           H
ATOM   1751  HG3 GLN   134      -4.947  69.097  32.185  1.00  0.00           H
ATOM   1752 HE22 GLN   134      -6.893  66.709  29.694  1.00  0.00           H
ATOM   1753 HE21 GLN   134      -7.577  68.196  30.316  1.00  0.00           H
ATOM   1754  N   LEU   135      -7.023  71.743  34.090  1.00125.41        1SG N
ATOM   1755  CA  LEU   135      -6.661  73.165  34.111  1.00125.41        1SG C
ATOM   1756  C   LEU   135      -5.785  73.609  32.920  1.00125.41        1SG C
ATOM   1757  O   LEU   135      -5.148  74.662  32.960  1.00125.41        1SG O
ATOM   1758  CB  LEU   135      -6.287  73.743  35.506  1.00125.41        1SG C
ATOM   1759  CG  LEU   135      -7.435  73.888  36.539  1.00125.41        1SG C
ATOM   1760  CD2 LEU   135      -8.000  72.552  37.048  1.00125.41        1SG C
ATOM   1761  CD1 LEU   135      -8.559  74.830  36.071  1.00125.41        1SG C
ATOM   1762  H   LEU   135      -7.854  71.558  33.542  1.00  0.00           H
ATOM   1763  HA  LEU   135      -7.600  73.650  33.846  1.00  0.00           H
ATOM   1764  HB2 LEU   135      -5.465  73.178  35.944  1.00  0.00           H
ATOM   1765  HB3 LEU   135      -5.874  74.743  35.367  1.00  0.00           H
ATOM   1766  HG  LEU   135      -6.981  74.369  37.407  1.00  0.00           H
ATOM   1767 HD21 LEU   135      -8.660  72.710  37.901  1.00  0.00           H
ATOM   1768 HD22 LEU   135      -8.583  72.042  36.282  1.00  0.00           H
```

```
ATOM   1769 HD23 LEU   135      -7.202  71.883  37.370  1.00  0.00           H
ATOM   1770 HD11 LEU   135      -9.241  75.064  36.889  1.00  0.00           H
ATOM   1771 HD12 LEU   135      -8.162  75.776  35.702  1.00  0.00           H
ATOM   1772 HD13 LEU   135      -9.164  74.396  35.274  1.00  0.00           H
ATOM   1773  N   PHE   136      -5.784  72.852  31.813  1.00 80.67       1SG N
ATOM   1774  CA  PHE   136      -5.073  73.182  30.568  1.00 80.67       1SG C
ATOM   1775  C   PHE   136      -5.929  74.142  29.709  1.00 80.67       1SG C
ATOM   1776  O   PHE   136      -6.317  73.826  28.581  1.00 80.67       1SG O
ATOM   1777  CB  PHE   136      -4.776  71.885  29.775  1.00 80.67       1SG C
ATOM   1778  CG  PHE   136      -3.782  70.891  30.361  1.00 80.67       1SG C
ATOM   1779  CD1 PHE   136      -3.034  71.160  31.532  1.00 80.67       1SG C
ATOM   1780  CD2 PHE   136      -3.606  69.655  29.702  1.00 80.67       1SG C
ATOM   1781  CE1 PHE   136      -2.139  70.199  32.039  1.00 80.67       1SG C
ATOM   1782  CE2 PHE   136      -2.710  68.696  30.209  1.00 80.67       1SG C
ATOM   1783  CZ  PHE   136      -1.977  68.966  31.380  1.00 80.67       1SG C
ATOM   1784  H   PHE   136      -6.362  72.024  31.830  1.00  0.00           H
ATOM   1785  HA  PHE   136      -4.133  73.691  30.785  1.00  0.00           H
ATOM   1786  HB2 PHE   136      -5.704  71.350  29.569  1.00  0.00           H
ATOM   1787  HB3 PHE   136      -4.366  72.155  28.801  1.00  0.00           H
ATOM   1788  HD1 PHE   136      -3.143  72.090  32.071  1.00  0.00           H
ATOM   1789  HD2 PHE   136      -4.175  69.428  28.811  1.00  0.00           H
ATOM   1790  HE1 PHE   136      -1.585  70.405  32.944  1.00  0.00           H
ATOM   1791  HE2 PHE   136      -2.600  67.742  29.713  1.00  0.00           H
ATOM   1792  HZ  PHE   136      -1.300  68.224  31.778  1.00  0.00           H
ATOM   1793  N   VAL   137      -6.252  75.308  30.276  1.00128.12       1SG N
ATOM   1794  CA  VAL   137      -7.080  76.378  29.717  1.00128.12       1SG C
ATOM   1795  C   VAL   137      -6.343  77.167  28.605  1.00128.12       1SG C
ATOM   1796  O   VAL   137      -5.367  76.689  28.020  1.00128.12       1SG O
ATOM   1797  CB  VAL   137      -7.526  77.282  30.907  1.00128.12       1SG C
ATOM   1798  CG1 VAL   137      -8.439  76.514  31.883  1.00128.12       1SG C
ATOM   1799  CG2 VAL   137      -6.360  77.945  31.673  1.00128.12       1SG C
ATOM   1800  H   VAL   137      -5.905  75.455  31.217  1.00  0.00           H
ATOM   1801  HA  VAL   137      -7.961  75.950  29.243  1.00  0.00           H
ATOM   1802  HB  VAL   137      -8.151  78.084  30.513  1.00  0.00           H
ATOM   1803 HG11 VAL   137      -8.848  77.171  32.652  1.00  0.00           H
ATOM   1804 HG12 VAL   137      -9.295  76.071  31.371  1.00  0.00           H
ATOM   1805 HG13 VAL   137      -7.918  75.708  32.398  1.00  0.00           H
ATOM   1806 HG21 VAL   137      -6.738  78.601  32.457  1.00  0.00           H
ATOM   1807 HG22 VAL   137      -5.716  77.210  32.155  1.00  0.00           H
ATOM   1808 HG23 VAL   137      -5.734  78.556  31.021  1.00  0.00           H
ATOM   1809  N   SER   138      -6.826  78.379  28.296  1.00 85.36       1SG N
ATOM   1810  CA  SER   138      -6.233  79.326  27.355  1.00 85.36       1SG C
ATOM   1811  C   SER   138      -4.890  79.821  27.921  1.00 85.36       1SG C
ATOM   1812  O   SER   138      -4.862  80.459  28.972  1.00 85.36       1SG O
ATOM   1813  CB  SER   138      -7.230  80.490  27.145  1.00 85.36       1SG C
ATOM   1814  OG  SER   138      -6.812  81.412  26.154  1.00 85.36       1SG O
ATOM   1815  H   SER   138      -7.600  78.714  28.846  1.00  0.00           H
ATOM   1816  HA  SER   138      -6.075  78.836  26.400  1.00  0.00           H
ATOM   1817  HB2 SER   138      -8.194  80.090  26.828  1.00  0.00           H
ATOM   1818  HB3 SER   138      -7.404  81.029  28.078  1.00  0.00           H
ATOM   1819  HG  SER   138      -6.059  81.896  26.468  1.00  0.00           H
ATOM   1820  N   GLY   139      -3.784  79.508  27.240  1.00 45.66       1SG N
ATOM   1821  CA  GLY   139      -2.424  79.778  27.700  1.00 45.66       1SG C
ATOM   1822  C   GLY   139      -1.565  78.510  27.652  1.00 45.66       1SG C
ATOM   1823  O   GLY   139      -0.737  78.426  26.747  1.00 45.66       1SG O
ATOM   1824  H   GLY   139      -3.892  78.904  26.432  1.00  0.00           H
ATOM   1825  HA2 GLY   139      -1.986  80.528  27.040  1.00  0.00           H
ATOM   1826  HA3 GLY   139      -2.392  80.206  28.702  1.00  0.00           H
ATOM   1827  N   PRO   140      -1.736  77.537  28.584  1.00161.76       1SG N
ATOM   1828  CA  PRO   140      -1.059  76.225  28.606  1.00161.76       1SG C
ATOM   1829  C   PRO   140      -0.837  75.536  27.243  1.00161.76       1SG C
ATOM   1830  O   PRO   140      -1.784  75.272  26.499  1.00161.76       1SG O
ATOM   1831  CD  PRO   140      -2.607  77.661  29.760  1.00161.76       1SG C
```

```
ATOM   1832  CB  PRO   140      -1.903  75.371  29.560  1.00161.76      1SG C
ATOM   1833  CG  PRO   140      -2.424  76.377  30.573  1.00161.76      1SG C
ATOM   1834  HA  PRO   140      -0.087  76.403  29.070  1.00  0.00          H
ATOM   1835  HD3 PRO   140      -2.358  78.537  30.360  1.00  0.00          H
ATOM   1836  HD2 PRO   140      -3.643  77.761  29.454  1.00  0.00          H
ATOM   1837  HB2 PRO   140      -2.747  74.926  29.028  1.00  0.00          H
ATOM   1838  HB3 PRO   140      -1.336  74.563  30.023  1.00  0.00          H
ATOM   1839  HG3 PRO   140      -1.675  76.536  31.351  1.00  0.00          H
ATOM   1840  HG2 PRO   140      -3.344  76.056  31.065  1.00  0.00          H
ATOM   1841  N   ASN   141       0.433  75.231  26.939  1.00120.02      1SG N
ATOM   1842  CA  ASN   141       0.973  74.772  25.650  1.00120.02      1SG C
ATOM   1843  C   ASN   141       0.640  73.281  25.382  1.00120.02      1SG C
ATOM   1844  O   ASN   141       1.502  72.493  24.997  1.00120.02      1SG O
ATOM   1845  CB  ASN   141       2.513  74.996  25.693  1.00120.02      1SG C
ATOM   1846  CG  ASN   141       3.178  75.131  24.317  1.00120.02      1SG C
ATOM   1847  OD1 ASN   141       3.652  76.201  23.952  1.00120.02      1SG O
ATOM   1848  ND2 ASN   141       3.292  74.049  23.564  1.00120.02      1SG N
ATOM   1849  H   ASN   141       1.122  75.450  27.641  1.00  0.00          H
ATOM   1850  HA  ASN   141       0.534  75.380  24.856  1.00  0.00          H
ATOM   1851  HB2 ASN   141       2.731  75.926  26.220  1.00  0.00          H
ATOM   1852  HB3 ASN   141       3.019  74.214  26.262  1.00  0.00          H
ATOM   1853 HD22 ASN   141       3.739  74.107  22.656  1.00  0.00          H
ATOM   1854 HD21 ASN   141       2.898  73.172  23.895  1.00  0.00          H
ATOM   1855  N   GLY   142      -0.617  72.884  25.585  1.00117.76      1SG N
ATOM   1856  CA  GLY   142      -1.137  71.549  25.306  1.00117.76      1SG C
ATOM   1857  C   GLY   142      -2.268  71.669  24.293  1.00117.76      1SG C
ATOM   1858  O   GLY   142      -2.150  71.230  23.151  1.00117.76      1SG O
ATOM   1859  H   GLY   142      -1.264  73.602  25.903  1.00  0.00          H
ATOM   1860  HA2 GLY   142      -0.376  70.869  24.919  1.00  0.00          H
ATOM   1861  HA3 GLY   142      -1.516  71.106  26.228  1.00  0.00          H
ATOM   1862  N   THR   143      -3.343  72.352  24.691  1.00315.28      1SG N
ATOM   1863  CA  THR   143      -4.619  72.515  23.994  1.00315.28      1SG C
ATOM   1864  C   THR   143      -4.514  73.332  22.675  1.00315.28      1SG C
ATOM   1865  O   THR   143      -5.486  73.426  21.930  1.00315.28      1SG O
ATOM   1866  CB  THR   143      -5.579  73.172  25.027  1.00315.28      1SG C
ATOM   1867  OG1 THR   143      -5.351  72.600  26.313  1.00315.28      1SG O
ATOM   1868  CG2 THR   143      -7.071  72.986  24.713  1.00315.28      1SG C
ATOM   1869  H   THR   143      -3.418  72.617  25.666  1.00  0.00          H
ATOM   1870  HA  THR   143      -4.987  71.518  23.742  1.00  0.00          H
ATOM   1871  HB  THR   143      -5.352  74.235  25.102  1.00  0.00          H
ATOM   1872  HG1 THR   143      -5.830  73.102  26.977  1.00  0.00          H
ATOM   1873 HG21 THR   143      -7.699  73.418  25.493  1.00  0.00          H
ATOM   1874 HG22 THR   143      -7.342  73.473  23.777  1.00  0.00          H
ATOM   1875 HG23 THR   143      -7.334  71.931  24.626  1.00  0.00          H
ATOM   1876  N   ALA   144      -3.329  73.871  22.346  1.00187.03      1SG N
ATOM   1877  CA  ALA   144      -3.003  74.515  21.071  1.00187.03      1SG C
ATOM   1878  C   ALA   144      -2.219  73.578  20.121  1.00187.03      1SG C
ATOM   1879  O   ALA   144      -1.941  73.951  18.977  1.00187.03      1SG O
ATOM   1880  CB  ALA   144      -2.107  75.726  21.374  1.00187.03      1SG C
ATOM   1881  H   ALA   144      -2.587  73.742  23.016  1.00  0.00          H
ATOM   1882  HA  ALA   144      -3.907  74.855  20.561  1.00  0.00          H
ATOM   1883  HB1 ALA   144      -1.897  76.291  20.465  1.00  0.00          H
ATOM   1884  HB2 ALA   144      -2.608  76.409  22.057  1.00  0.00          H
ATOM   1885  HB3 ALA   144      -1.160  75.437  21.831  1.00  0.00          H
ATOM   1886  N   GLY   145      -1.869  72.358  20.556  1.00 80.28      1SG N
ATOM   1887  CA  GLY   145      -0.970  71.436  19.845  1.00 80.28      1SG C
ATOM   1888  C   GLY   145      -1.720  70.477  18.916  1.00 80.28      1SG C
ATOM   1889  O   GLY   145      -1.116  69.769  18.117  1.00 80.28      1SG O
ATOM   1890  H   GLY   145      -2.134  72.078  21.500  1.00  0.00          H
ATOM   1891  HA2 GLY   145      -0.228  71.975  19.256  1.00  0.00          H
ATOM   1892  HA3 GLY   145      -0.419  70.846  20.577  1.00  0.00          H
ATOM   1893  N   ILE   146      -3.053  70.561  18.947  1.00131.45      1SG N
ATOM   1894  CA  ILE   146      -4.004  70.154  17.915  1.00131.45      1SG C
```

```
ATOM   1895  C   ILE   146      -3.705  70.842  16.553  1.00131.45      1SG C
ATOM   1896  O   ILE   146      -3.997  70.283  15.495  1.00131.45      1SG O
ATOM   1897  CB  ILE   146      -5.453  70.403  18.462  1.00131.45      1SG C
ATOM   1898  CG2 ILE   146      -5.568  71.690  19.313  1.00131.45      1SG C
ATOM   1899  CG1 ILE   146      -6.563  70.319  17.386  1.00131.45      1SG C
ATOM   1900  CD1 ILE   146      -7.999  70.450  17.924  1.00131.45      1SG C
ATOM   1901  H   ILE   146      -3.427  71.148  19.676  1.00  0.00          H
ATOM   1902  HA  ILE   146      -3.892  69.081  17.767  1.00  0.00          H
ATOM   1903  HB  ILE   146      -5.653  69.580  19.152  1.00  0.00          H
ATOM   1904 HG21 ILE   146      -6.576  71.864  19.684  1.00  0.00          H
ATOM   1905 HG22 ILE   146      -4.957  71.628  20.211  1.00  0.00          H
ATOM   1906 HG23 ILE   146      -5.261  72.574  18.758  1.00  0.00          H
ATOM   1907 HG12 ILE   146      -6.417  71.087  16.629  1.00  0.00          H
ATOM   1908 HG13 ILE   146      -6.466  69.367  16.863  1.00  0.00          H
ATOM   1909 HD11 ILE   146      -8.727  70.274  17.133  1.00  0.00          H
ATOM   1910 HD12 ILE   146      -8.194  69.739  18.727  1.00  0.00          H
ATOM   1911 HD13 ILE   146      -8.206  71.449  18.303  1.00  0.00          H
ATOM   1912  N   PHE   147      -3.111  72.049  16.572  1.00179.81      1SG N
ATOM   1913  CA  PHE   147      -2.925  72.901  15.400  1.00179.81      1SG C
ATOM   1914  C   PHE   147      -1.450  73.191  15.114  1.00179.81      1SG C
ATOM   1915  O   PHE   147      -0.960  72.842  14.038  1.00179.81      1SG O
ATOM   1916  CB  PHE   147      -3.737  74.204  15.569  1.00179.81      1SG C
ATOM   1917  CG  PHE   147      -5.234  73.971  15.649  1.00179.81      1SG C
ATOM   1918  CD1 PHE   147      -5.913  73.322  14.594  1.00179.81      1SG C
ATOM   1919  CD2 PHE   147      -5.938  74.333  16.816  1.00179.81      1SG C
ATOM   1920  CE1 PHE   147      -7.273  72.988  14.732  1.00179.81      1SG C
ATOM   1921  CE2 PHE   147      -7.298  74.005  16.948  1.00179.81      1SG C
ATOM   1922  CZ  PHE   147      -7.959  73.315  15.914  1.00179.81      1SG C
ATOM   1923  H   PHE   147      -2.848  72.417  17.475  1.00  0.00          H
ATOM   1924  HA  PHE   147      -3.297  72.396  14.507  1.00  0.00          H
ATOM   1925  HB2 PHE   147      -3.408  74.731  16.466  1.00  0.00          H
ATOM   1926  HB3 PHE   147      -3.544  74.885  14.742  1.00  0.00          H
ATOM   1927  HD1 PHE   147      -5.392  73.034  13.694  1.00  0.00          H
ATOM   1928  HD2 PHE   147      -5.432  74.816  17.640  1.00  0.00          H
ATOM   1929  HE1 PHE   147      -7.789  72.454  13.947  1.00  0.00          H
ATOM   1930  HE2 PHE   147      -7.820  74.248  17.862  1.00  0.00          H
ATOM   1931  HZ  PHE   147      -8.988  73.013  16.035  1.00  0.00          H
ATOM   1932  N   ALA   148      -0.743  73.855  16.033  1.00 70.57      1SG N
ATOM   1933  CA  ALA   148       0.623  74.335  15.825  1.00 70.57      1SG C
ATOM   1934  C   ALA   148       1.633  73.281  16.301  1.00 70.57      1SG C
ATOM   1935  O   ALA   148       1.407  72.638  17.325  1.00 70.57      1SG O
ATOM   1936  CB  ALA   148       0.806  75.588  16.693  1.00 70.57      1SG C
ATOM   1937  H   ALA   148      -1.135  73.972  16.961  1.00  0.00          H
ATOM   1938  HA  ALA   148       0.802  74.588  14.777  1.00  0.00          H
ATOM   1939  HB1 ALA   148       1.819  75.977  16.609  1.00  0.00          H
ATOM   1940  HB2 ALA   148       0.126  76.383  16.391  1.00  0.00          H
ATOM   1941  HB3 ALA   148       0.623  75.374  17.745  1.00  0.00          H
ATOM   1942  N   THR   149       2.747  73.120  15.577  1.00 72.38      1SG N
ATOM   1943  CA  THR   149       3.873  72.278  15.986  1.00 72.38      1SG C
ATOM   1944  C   THR   149       4.630  72.895  17.181  1.00 72.38      1SG C
ATOM   1945  O   THR   149       4.847  74.111  17.238  1.00 72.38      1SG O
ATOM   1946  CB  THR   149       4.837  72.065  14.788  1.00 72.38      1SG C
ATOM   1947  OG1 THR   149       5.211  73.266  14.136  1.00 72.38      1SG O
ATOM   1948  CG2 THR   149       4.308  71.075  13.747  1.00 72.38      1SG C
ATOM   1949  H   THR   149       2.866  73.654  14.717  1.00  0.00          H
ATOM   1950  HA  THR   149       3.484  71.307  16.301  1.00  0.00          H
ATOM   1951  HB  THR   149       5.760  71.654  15.183  1.00  0.00          H
ATOM   1952  HG1 THR   149       4.439  73.588  13.651  1.00  0.00          H
ATOM   1953 HG21 THR   149       5.088  70.797  13.040  1.00  0.00          H
ATOM   1954 HG22 THR   149       3.967  70.158  14.225  1.00  0.00          H
ATOM   1955 HG23 THR   149       3.472  71.476  13.182  1.00  0.00          H
ATOM   1956  N   TYR   150       5.051  72.042  18.116  1.00144.44      1SG N
ATOM   1957  CA  TYR   150       5.930  72.341  19.240  1.00144.44      1SG C
```

```
ATOM   1958  C   TYR   150       6.885  71.142  19.339  1.00144.44       1SG C
ATOM   1959  O   TYR   150       6.413  70.018  19.137  1.00144.44       1SG O
ATOM   1960  CB  TYR   150       5.122  72.555  20.536  1.00144.44       1SG C
ATOM   1961  CG  TYR   150       4.014  73.593  20.453  1.00144.44       1SG C
ATOM   1962  CD1 TYR   150       4.321  74.968  20.410  1.00144.44       1SG C
ATOM   1963  CD2 TYR   150       2.667  73.179  20.408  1.00144.44       1SG C
ATOM   1964  CE1 TYR   150       3.291  75.927  20.349  1.00144.44       1SG C
ATOM   1965  CE2 TYR   150       1.640  74.135  20.332  1.00144.44       1SG C
ATOM   1966  CZ  TYR   150       1.949  75.506  20.302  1.00144.44       1SG C
ATOM   1967  OH  TYR   150       0.944  76.419  20.208  1.00144.44       1SG O
ATOM   1968  H   TYR   150       4.909  71.049  17.975  1.00  0.00           H
ATOM   1969  HA  TYR   150       6.486  73.250  19.011  1.00  0.00           H
ATOM   1970  HB2 TYR   150       4.693  71.605  20.859  1.00  0.00           H
ATOM   1971  HB3 TYR   150       5.804  72.852  21.335  1.00  0.00           H
ATOM   1972  HD1 TYR   150       5.353  75.287  20.425  1.00  0.00           H
ATOM   1973  HD2 TYR   150       2.417  72.128  20.412  1.00  0.00           H
ATOM   1974  HE1 TYR   150       3.532  76.981  20.342  1.00  0.00           H
ATOM   1975  HE2 TYR   150       0.616  73.805  20.285  1.00  0.00           H
ATOM   1976  HH  TYR   150       0.097  76.015  20.093  1.00  0.00           H
ATOM   1977  N   PRO   151       8.190  71.339  19.610  1.00 85.62       1SG N
ATOM   1978  CA  PRO   151       9.175  70.261  19.523  1.00 85.62       1SG C
ATOM   1979  C   PRO   151       9.244  69.404  20.795  1.00 85.62       1SG C
ATOM   1980  O   PRO   151       8.732  69.781  21.854  1.00 85.62       1SG O
ATOM   1981  CD  PRO   151       8.831  72.620  19.919  1.00 85.62       1SG C
ATOM   1982  CB  PRO   151      10.509  71.010  19.349  1.00 85.62       1SG C
ATOM   1983  CG  PRO   151      10.313  72.303  20.129  1.00 85.62       1SG C
ATOM   1984  HA  PRO   151       8.955  69.628  18.663  1.00  0.00           H
ATOM   1985  HD3 PRO   151       8.717  73.302  19.078  1.00  0.00           H
ATOM   1986  HD2 PRO   151       8.396  73.083  20.805  1.00  0.00           H
ATOM   1987  HB2 PRO   151      11.387  70.452  19.677  1.00  0.00           H
ATOM   1988  HB3 PRO   151      10.657  71.224  18.291  1.00  0.00           H
ATOM   1989  HG3 PRO   151      10.978  73.103  19.805  1.00  0.00           H
ATOM   1990  HG2 PRO   151      10.502  72.127  21.188  1.00  0.00           H
ATOM   1991  N   SER   152      10.035  68.327  20.730  1.00 83.16       1SG N
ATOM   1992  CA  SER   152      10.683  67.744  21.902  1.00 83.16       1SG C
ATOM   1993  C   SER   152      11.810  68.710  22.320  1.00 83.16       1SG C
ATOM   1994  O   SER   152      12.960  68.576  21.900  1.00 83.16       1SG O
ATOM   1995  CB  SER   152      11.271  66.361  21.550  1.00 83.16       1SG C
ATOM   1996  OG  SER   152      10.244  65.390  21.498  1.00 83.16       1SG O
ATOM   1997  H   SER   152      10.417  68.101  19.815  1.00  0.00           H
ATOM   1998  HA  SER   152       9.975  67.639  22.727  1.00  0.00           H
ATOM   1999  HB2 SER   152      11.822  66.381  20.608  1.00  0.00           H
ATOM   2000  HB3 SER   152      11.976  66.046  22.321  1.00  0.00           H
ATOM   2001  HG  SER   152       9.822  65.466  20.639  1.00  0.00           H
ATOM   2002  N   GLY   153      11.477  69.719  23.135  1.00 30.39       1SG N
ATOM   2003  CA  GLY   153      12.238  70.965  23.272  1.00 30.39       1SG C
ATOM   2004  C   GLY   153      13.446  70.883  24.211  1.00 30.39       1SG C
ATOM   2005  O   GLY   153      13.818  71.871  24.839  1.00 30.39       1SG O
ATOM   2006  H   GLY   153      10.492  69.773  23.364  1.00  0.00           H
ATOM   2007  HA2 GLY   153      12.588  71.296  22.292  1.00  0.00           H
ATOM   2008  HA3 GLY   153      11.566  71.743  23.634  1.00  0.00           H
ATOM   2009  N   HIE   154      14.071  69.702  24.266  1.00 51.75       1SG N
ATOM   2010  CA  HIE   154      15.474  69.510  24.627  1.00 51.75       1SG C
ATOM   2011  C   HIE   154      16.351  69.666  23.368  1.00 51.75       1SG C
ATOM   2012  O   HIE   154      17.543  69.939  23.489  1.00 51.75       1SG O
ATOM   2013  ND1 HIE   154      15.129  66.604  27.185  1.00 51.75       1SG N
ATOM   2014  CG  HIE   154      15.075  67.850  26.552  1.00 51.75       1SG C
ATOM   2015  CB  HIE   154      15.667  68.085  25.188  1.00 51.75       1SG C
ATOM   2016  NE2 HIE   154      14.074  68.019  28.523  1.00 51.75       1SG N
ATOM   2017  CD2 HIE   154      14.417  68.732  27.386  1.00 51.75       1SG C
ATOM   2018  CE1 HIE   154      14.518  66.775  28.349  1.00 51.75       1SG C
ATOM   2019  H   HIE   154      13.664  68.997  23.662  1.00  0.00           H
ATOM   2020  HA  HIE   154      15.800  70.254  25.358  1.00  0.00           H
```

```
ATOM   2021  HB2 HIE   154      15.260  67.344  24.498  1.00  0.00           H
ATOM   2022  HB3 HIE   154      16.733  67.867  25.271  1.00  0.00           H
ATOM   2023  HD2 HIE   154      14.178  69.778  27.248  1.00  0.00           H
ATOM   2024  HE1 HIE   154      14.397  65.985  29.077  1.00  0.00           H
ATOM   2025  HE2 HIE   154      13.584  68.371  29.332  1.00  0.00           H
ATOM   2026  N   LEU   155      15.753  69.562  22.171  1.00 53.40       1SG N
ATOM   2027  CA  LEU   155      16.302  70.050  20.912  1.00 53.40       1SG C
ATOM   2028  C   LEU   155      16.064  71.568  20.883  1.00 53.40       1SG C
ATOM   2029  O   LEU   155      14.963  72.030  21.201  1.00 53.40       1SG O
ATOM   2030  CB  LEU   155      15.512  69.428  19.737  1.00 53.40       1SG C
ATOM   2031  CG  LEU   155      15.595  67.890  19.624  1.00 53.40       1SG C
ATOM   2032  CD2 LEU   155      17.010  67.404  19.268  1.00 53.40       1SG C
ATOM   2033  CD1 LEU   155      14.575  67.380  18.597  1.00 53.40       1SG C
ATOM   2034  H   LEU   155      14.766  69.340  22.148  1.00  0.00           H
ATOM   2035  HA  LEU   155      17.367  69.826  20.837  1.00  0.00           H
ATOM   2036  HB2 LEU   155      14.461  69.714  19.829  1.00  0.00           H
ATOM   2037  HB3 LEU   155      15.843  69.874  18.799  1.00  0.00           H
ATOM   2038  HG  LEU   155      15.322  67.455  20.586  1.00  0.00           H
ATOM   2039 HD21 LEU   155      17.042  66.317  19.186  1.00  0.00           H
ATOM   2040 HD22 LEU   155      17.347  67.812  18.314  1.00  0.00           H
ATOM   2041 HD23 LEU   155      17.739  67.688  20.026  1.00  0.00           H
ATOM   2042 HD11 LEU   155      14.582  66.292  18.537  1.00  0.00           H
ATOM   2043 HD12 LEU   155      13.564  67.690  18.865  1.00  0.00           H
ATOM   2044 HD13 LEU   155      14.786  67.773  17.601  1.00  0.00           H
ATOM   2045  N   ASP   156      17.098  72.336  20.533  1.00 59.44       1SG N
ATOM   2046  CA  ASP   156      17.073  73.796  20.455  1.00 59.44       1SG C
ATOM   2047  C   ASP   156      16.435  74.247  19.111  1.00 59.44       1SG C
ATOM   2048  O   ASP   156      15.895  73.446  18.343  1.00 59.44       1SG O
ATOM   2049  CB  ASP   156      18.530  74.319  20.658  1.00 59.44       1SG C
ATOM   2050  CG  ASP   156      19.318  74.610  19.388  1.00 59.44       1SG C
ATOM   2051  OD1 ASP   156      19.671  73.641  18.688  1.00 59.44       1SG O
ATOM   2052  OD2 ASP   156      19.326  75.798  19.014  1.00 59.44       1SG O1-
ATOM   2053  H   ASP   156      17.953  71.888  20.242  1.00  0.00           H
ATOM   2054  HA  ASP   156      16.459  74.179  21.270  1.00  0.00           H
ATOM   2055  HB2 ASP   156      18.487  75.250  21.224  1.00  0.00           H
ATOM   2056  HB3 ASP   156      19.112  73.637  21.277  1.00  0.00           H
ATOM   2057  N   MET   157      16.504  75.553  18.834  1.00 83.98       1SG N
ATOM   2058  CA  MET   157      15.941  76.194  17.653  1.00 83.98       1SG C
ATOM   2059  C   MET   157      16.771  76.016  16.410  1.00 83.98       1SG C
ATOM   2060  O   MET   157      16.217  76.082  15.316  1.00 83.98       1SG O
ATOM   2061  CB  MET   157      15.746  77.690  17.982  1.00 83.98       1SG C
ATOM   2062  CG  MET   157      14.736  78.393  17.063  1.00 83.98       1SG C
ATOM   2063  SD  MET   157      14.133  79.981  17.708  1.00 83.98       1SG S
ATOM   2064  CE  MET   157      13.383  80.633  16.194  1.00 83.98       1SG C
ATOM   2065  H   MET   157      17.223  76.063  19.337  1.00  0.00           H
ATOM   2066  HA  MET   157      14.963  75.743  17.470  1.00  0.00           H
ATOM   2067  HB2 MET   157      15.362  77.767  19.001  1.00  0.00           H
ATOM   2068  HB3 MET   157      16.700  78.221  17.978  1.00  0.00           H
ATOM   2069  HG2 MET   157      15.188  78.551  16.083  1.00  0.00           H
ATOM   2070  HG3 MET   157      13.867  77.754  16.905  1.00  0.00           H
ATOM   2071  HE1 MET   157      12.950  81.614  16.387  1.00  0.00           H
ATOM   2072  HE2 MET   157      14.132  80.736  15.408  1.00  0.00           H
ATOM   2073  HE3 MET   157      12.596  79.969  15.839  1.00  0.00           H
ATOM   2074  N   ILE   158      18.060  75.742  16.569  1.00117.58       1SG N
ATOM   2075  CA  ILE   158      18.887  75.314  15.478  1.00117.58       1SG C
ATOM   2076  C   ILE   158      18.661  73.859  15.154  1.00117.58       1SG C
ATOM   2077  O   ILE   158      18.497  73.498  13.986  1.00117.58       1SG O
ATOM   2078  CB  ILE   158      20.393  75.639  15.778  1.00117.58       1SG C
ATOM   2079  CG2 ILE   158      21.390  74.898  14.854  1.00117.58       1SG C
ATOM   2080  CG1 ILE   158      20.694  77.161  15.675  1.00117.58       1SG C
ATOM   2081  CD1 ILE   158      20.270  78.015  16.878  1.00117.58       1SG C
ATOM   2082  H   ILE   158      18.486  75.734  17.506  1.00  0.00           H
ATOM   2083  HA  ILE   158      18.615  75.871  14.579  1.00  0.00           H
```

ATOM	2084	HB	ILE	158	20.630	75.311	16.791	1.00	0.00		H
ATOM	2085	HG21	ILE	158	22.419	75.192	15.061	1.00	0.00		H
ATOM	2086	HG22	ILE	158	21.354	73.817	14.995	1.00	0.00		H
ATOM	2087	HG23	ILE	158	21.188	75.107	13.803	1.00	0.00		H
ATOM	2088	HG12	ILE	158	21.769	77.306	15.565	1.00	0.00		H
ATOM	2089	HG13	ILE	158	20.253	77.566	14.763	1.00	0.00		H
ATOM	2090	HD11	ILE	158	20.602	79.046	16.756	1.00	0.00		H
ATOM	2091	HD12	ILE	158	19.190	78.038	17.008	1.00	0.00		H
ATOM	2092	HD13	ILE	158	20.709	77.635	17.800	1.00	0.00		H
ATOM	2093	N	ASN	159	18.590	73.036	16.191	1.00	31.52	1SG	N
ATOM	2094	CA	ASN	159	18.272	71.639	16.034	1.00	31.52	1SG	C
ATOM	2095	C	ASN	159	16.939	71.349	15.364	1.00	31.52	1SG	C
ATOM	2096	O	ASN	159	16.843	70.449	14.529	1.00	31.52	1SG	O
ATOM	2097	CB	ASN	159	18.343	70.883	17.381	1.00	31.52	1SG	C
ATOM	2098	CG	ASN	159	19.744	70.389	17.736	1.00	31.52	1SG	C
ATOM	2099	OD1	ASN	159	20.088	69.239	17.488	1.00	31.52	1SG	O
ATOM	2100	ND2	ASN	159	20.563	71.228	18.353	1.00	31.52	1SG	N
ATOM	2101	H	ASN	159	18.851	73.389	17.118	1.00	0.00		H
ATOM	2102	HA	ASN	159	19.027	71.211	15.370	1.00	0.00		H
ATOM	2103	HB2	ASN	159	17.941	71.497	18.183	1.00	0.00		H
ATOM	2104	HB3	ASN	159	17.714	69.993	17.345	1.00	0.00		H
ATOM	2105	HD22	ASN	159	21.495	70.938	18.581	1.00	0.00		H
ATOM	2106	HD21	ASN	159	20.260	72.216	18.503	1.00	0.00		H
ATOM	2107	N	GLY	160	15.952	72.195	15.646	1.00	29.09	1SG	N
ATOM	2108	CA	GLY	160	14.638	72.055	15.062	1.00	29.09	1SG	C
ATOM	2109	C	GLY	160	14.497	72.728	13.711	1.00	29.09	1SG	C
ATOM	2110	O	GLY	160	13.538	72.495	12.983	1.00	29.09	1SG	O
ATOM	2111	H	GLY	160	16.084	72.863	16.400	1.00	0.00		H
ATOM	2112	HA2	GLY	160	14.357	71.006	14.990	1.00	0.00		H
ATOM	2113	HA3	GLY	160	13.921	72.514	15.739	1.00	0.00		H
ATOM	2114	N	PHE	161	15.492	73.532	13.345	1.00	44.01	1SG	N
ATOM	2115	CA	PHE	161	15.639	74.027	11.996	1.00	44.01	1SG	C
ATOM	2116	C	PHE	161	16.247	72.964	11.108	1.00	44.01	1SG	C
ATOM	2117	O	PHE	161	15.857	72.806	9.952	1.00	44.01	1SG	O
ATOM	2118	CB	PHE	161	16.583	75.259	12.031	1.00	44.01	1SG	C
ATOM	2119	CG	PHE	161	16.475	76.247	10.883	1.00	44.01	1SG	C
ATOM	2120	CD1	PHE	161	15.867	77.503	11.097	1.00	44.01	1SG	C
ATOM	2121	CD2	PHE	161	16.994	75.931	9.610	1.00	44.01	1SG	C
ATOM	2122	CE1	PHE	161	15.764	78.431	10.044	1.00	44.01	1SG	C
ATOM	2123	CE2	PHE	161	16.897	76.861	8.560	1.00	44.01	1SG	C
ATOM	2124	CZ	PHE	161	16.283	78.111	8.777	1.00	44.01	1SG	C
ATOM	2125	H	PHE	161	16.254	73.666	13.995	1.00	0.00		H
ATOM	2126	HA	PHE	161	14.665	74.319	11.609	1.00	0.00		H
ATOM	2127	HB2	PHE	161	16.388	75.828	12.934	1.00	0.00		H
ATOM	2128	HB3	PHE	161	17.628	74.958	12.108	1.00	0.00		H
ATOM	2129	HD1	PHE	161	15.471	77.757	12.069	1.00	0.00		H
ATOM	2130	HD2	PHE	161	17.472	74.978	9.432	1.00	0.00		H
ATOM	2131	HE1	PHE	161	15.292	79.388	10.210	1.00	0.00		H
ATOM	2132	HE2	PHE	161	17.307	76.609	7.592	1.00	0.00		H
ATOM	2133	HZ	PHE	161	16.218	78.834	7.979	1.00	0.00		H
ATOM	2134	N	PHE	162	17.211	72.239	11.672	1.00	91.11	1SG	N
ATOM	2135	CA	PHE	162	17.956	71.223	10.981	1.00	91.11	1SG	C
ATOM	2136	C	PHE	162	17.114	70.034	10.589	1.00	91.11	1SG	C
ATOM	2137	O	PHE	162	17.225	69.529	9.470	1.00	91.11	1SG	O
ATOM	2138	CB	PHE	162	19.150	70.780	11.857	1.00	91.11	1SG	C
ATOM	2139	CG	PHE	162	20.121	69.830	11.172	1.00	91.11	1SG	C
ATOM	2140	CD1	PHE	162	20.985	70.306	10.163	1.00	91.11	1SG	C
ATOM	2141	CD2	PHE	162	20.137	68.462	11.516	1.00	91.11	1SG	C
ATOM	2142	CE1	PHE	162	21.853	69.419	9.497	1.00	91.11	1SG	C
ATOM	2143	CE2	PHE	162	21.005	67.575	10.850	1.00	91.11	1SG	C
ATOM	2144	CZ	PHE	162	21.863	68.053	9.840	1.00	91.11	1SG	C
ATOM	2145	H	PHE	162	17.477	72.458	12.626	1.00	0.00		H
ATOM	2146	HA	PHE	162	18.350	71.666	10.064	1.00	0.00		H

```
ATOM   2147  HB2 PHE   162      19.718  71.657  12.170  1.00  0.00           H
ATOM   2148  HB3 PHE   162      18.794  70.319  12.779  1.00  0.00           H
ATOM   2149  HD1 PHE   162      20.973  71.351   9.887  1.00  0.00           H
ATOM   2150  HD2 PHE   162      19.473  68.085  12.281  1.00  0.00           H
ATOM   2151  HE1 PHE   162      22.505  69.786   8.719  1.00  0.00           H
ATOM   2152  HE2 PHE   162      21.007  66.527  11.111  1.00  0.00           H
ATOM   2153  HZ  PHE   162      22.523  67.371   9.326  1.00  0.00           H
ATOM   2154  N   ASP   163      16.235  69.631  11.501  1.00108.53       1SG N
ATOM   2155  CA  ASP   163      15.376  68.504  11.254  1.00108.53       1SG C
ATOM   2156  C   ASP   163      14.353  68.800  10.160  1.00108.53       1SG C
ATOM   2157  O   ASP   163      14.167  68.018   9.224  1.00108.53       1SG O
ATOM   2158  CB  ASP   163      14.697  68.029  12.576  1.00108.53       1SG C
ATOM   2159  CG  ASP   163      13.257  68.477  12.864  1.00108.53       1SG C
ATOM   2160  OD1 ASP   163      12.315  67.838  12.344  1.00108.53       1SG O
ATOM   2161  OD2 ASP   163      13.101  69.464  13.602  1.00108.53       1SG O1-
ATOM   2162  H   ASP   163      16.190  70.088  12.405  1.00  0.00           H
ATOM   2163  HA  ASP   163      16.009  67.682  10.919  1.00  0.00           H
ATOM   2164  HB2 ASP   163      14.680  66.944  12.546  1.00  0.00           H
ATOM   2165  HB3 ASP   163      15.313  68.282  13.439  1.00  0.00           H
ATOM   2166  N   GLN   164      13.740  69.978  10.257  1.00 62.36       1SG N
ATOM   2167  CA  GLN   164      12.692  70.343   9.344  1.00 62.36       1SG C
ATOM   2168  C   GLN   164      13.224  70.777   7.986  1.00 62.36       1SG C
ATOM   2169  O   GLN   164      12.508  70.703   6.990  1.00 62.36       1SG O
ATOM   2170  CB  GLN   164      11.842  71.466   9.960  1.00 62.36       1SG C
ATOM   2171  CG  GLN   164      11.039  70.963  11.178  1.00 62.36       1SG C
ATOM   2172  CD  GLN   164       9.614  71.492  11.210  1.00 62.36       1SG C
ATOM   2173  OE1 GLN   164       8.807  71.199  10.331  1.00 62.36       1SG O
ATOM   2174  NE2 GLN   164       9.273  72.255  12.241  1.00 62.36       1SG N
ATOM   2175  H   GLN   164      13.852  70.497  11.120  1.00  0.00           H
ATOM   2176  HA  GLN   164      12.045  69.480   9.167  1.00  0.00           H
ATOM   2177  HB2 GLN   164      12.470  72.305  10.260  1.00  0.00           H
ATOM   2178  HB3 GLN   164      11.163  71.852   9.196  1.00  0.00           H
ATOM   2179  HG2 GLN   164      10.954  69.875  11.152  1.00  0.00           H
ATOM   2180  HG3 GLN   164      11.568  71.178  12.105  1.00  0.00           H
ATOM   2181 HE22 GLN   164       8.333  72.603  12.329  1.00  0.00           H
ATOM   2182 HE21 GLN   164       9.976  72.456  12.940  1.00  0.00           H
ATOM   2183  N   PHE   165      14.501  71.145   7.931  1.00 97.21       1SG N
ATOM   2184  CA  PHE   165      15.269  71.211   6.711  1.00 97.21       1SG C
ATOM   2185  C   PHE   165      15.424  69.842   6.079  1.00 97.21       1SG C
ATOM   2186  O   PHE   165      14.987  69.630   4.945  1.00 97.21       1SG O
ATOM   2187  CB  PHE   165      16.630  71.886   7.020  1.00 97.21       1SG C
ATOM   2188  CG  PHE   165      17.677  71.888   5.918  1.00 97.21       1SG C
ATOM   2189  CD1 PHE   165      18.679  70.894   5.896  1.00 97.21       1SG C
ATOM   2190  CD2 PHE   165      17.661  72.885   4.924  1.00 97.21       1SG C
ATOM   2191  CE1 PHE   165      19.644  70.885   4.871  1.00 97.21       1SG C
ATOM   2192  CE2 PHE   165      18.631  72.880   3.902  1.00 97.21       1SG C
ATOM   2193  CZ  PHE   165      19.619  71.877   3.872  1.00 97.21       1SG C
ATOM   2194  H   PHE   165      15.000  71.216   8.810  1.00  0.00           H
ATOM   2195  HA  PHE   165      14.737  71.839   5.994  1.00  0.00           H
ATOM   2196  HB2 PHE   165      16.446  72.923   7.303  1.00  0.00           H
ATOM   2197  HB3 PHE   165      17.086  71.434   7.897  1.00  0.00           H
ATOM   2198  HD1 PHE   165      18.707  70.129   6.660  1.00  0.00           H
ATOM   2199  HD2 PHE   165      16.899  73.650   4.939  1.00  0.00           H
ATOM   2200  HE1 PHE   165      20.403  70.116   4.852  1.00  0.00           H
ATOM   2201  HE2 PHE   165      18.614  73.639   3.134  1.00  0.00           H
ATOM   2202  HZ  PHE   165      20.358  71.869   3.084  1.00  0.00           H
ATOM   2203  N   ILE   166      16.030  68.913   6.822  1.00 82.19       1SG N
ATOM   2204  CA  ILE   166      16.480  67.672   6.236  1.00 82.19       1SG C
ATOM   2205  C   ILE   166      15.356  66.739   5.808  1.00 82.19       1SG C
ATOM   2206  O   ILE   166      15.469  66.029   4.806  1.00 82.19       1SG O
ATOM   2207  CB  ILE   166      17.548  66.988   7.148  1.00 82.19       1SG C
ATOM   2208  CG2 ILE   166      16.972  66.222   8.353  1.00 82.19       1SG C
ATOM   2209  CG1 ILE   166      18.494  66.077   6.329  1.00 82.19       1SG C
```

```
ATOM   2210  CD1 ILE   166      19.760  65.650   7.087  1.00 82.19        1SG C
ATOM   2211  H   ILE   166      16.323  69.127   7.772  1.00  0.00            H
ATOM   2212  HA  ILE   166      16.992  67.954   5.313  1.00  0.00            H
ATOM   2213  HB  ILE   166      18.172  67.788   7.550  1.00  0.00            H
ATOM   2214 HG21 ILE   166      17.752  65.942   9.060  1.00  0.00            H
ATOM   2215 HG22 ILE   166      16.271  66.840   8.898  1.00  0.00            H
ATOM   2216 HG23 ILE   166      16.450  65.313   8.053  1.00  0.00            H
ATOM   2217 HG12 ILE   166      17.958  65.193   5.982  1.00  0.00            H
ATOM   2218 HG13 ILE   166      18.812  66.603   5.428  1.00  0.00            H
ATOM   2219 HD11 ILE   166      20.431  65.093   6.433  1.00  0.00            H
ATOM   2220 HD12 ILE   166      20.307  66.516   7.463  1.00  0.00            H
ATOM   2221 HD13 ILE   166      19.527  65.006   7.935  1.00  0.00            H
ATOM   2222  N   GLY   167      14.217  66.857   6.481  1.00 29.71        1SG N
ATOM   2223  CA  GLY   167      13.071  66.068   6.131  1.00 29.71        1SG C
ATOM   2224  C   GLY   167      12.286  66.658   4.990  1.00 29.71        1SG C
ATOM   2225  O   GLY   167      11.762  65.905   4.170  1.00 29.71        1SG O
ATOM   2226  H   GLY   167      14.203  67.417   7.330  1.00  0.00            H
ATOM   2227  HA2 GLY   167      13.352  65.035   5.915  1.00  0.00            H
ATOM   2228  HA3 GLY   167      12.416  66.020   7.000  1.00  0.00            H
ATOM   2229  N   THR   168      12.290  67.986   4.859  1.00 41.02        1SG N
ATOM   2230  CA  THR   168      11.629  68.602   3.728  1.00 41.02        1SG C
ATOM   2231  C   THR   168      12.410  68.475   2.435  1.00 41.02        1SG C
ATOM   2232  O   THR   168      11.824  68.420   1.352  1.00 41.02        1SG O
ATOM   2233  CB  THR   168      11.254  70.078   4.007  1.00 41.02        1SG C
ATOM   2234  OG1 THR   168      10.589  70.183   5.252  1.00 41.02        1SG O
ATOM   2235  CG2 THR   168      10.232  70.613   2.989  1.00 41.02        1SG C
ATOM   2236  H   THR   168      12.798  68.565   5.513  1.00  0.00            H
ATOM   2237  HA  THR   168      10.684  68.083   3.577  1.00  0.00            H
ATOM   2238  HB  THR   168      12.144  70.711   4.018  1.00  0.00            H
ATOM   2239  HG1 THR   168      11.262  70.262   5.933  1.00  0.00            H
ATOM   2240 HG21 THR   168       9.964  71.647   3.185  1.00  0.00            H
ATOM   2241 HG22 THR   168      10.622  70.576   1.975  1.00  0.00            H
ATOM   2242 HG23 THR   168       9.311  70.028   3.004  1.00  0.00            H
ATOM   2243  N   ALA   169      13.723  68.317   2.559  1.00 25.13        1SG N
ATOM   2244  CA  ALA   169      14.538  67.941   1.441  1.00 25.13        1SG C
ATOM   2245  C   ALA   169      14.239  66.549   0.945  1.00 25.13        1SG C
ATOM   2246  O   ALA   169      14.117  66.337  -0.252  1.00 25.13        1SG O
ATOM   2247  CB  ALA   169      16.018  68.043   1.848  1.00 25.13        1SG C
ATOM   2248  H   ALA   169      14.152  68.406   3.474  1.00  0.00            H
ATOM   2249  HA  ALA   169      14.336  68.636   0.634  1.00  0.00            H
ATOM   2250  HB1 ALA   169      16.674  67.799   1.011  1.00  0.00            H
ATOM   2251  HB2 ALA   169      16.270  69.053   2.171  1.00  0.00            H
ATOM   2252  HB3 ALA   169      16.267  67.365   2.664  1.00  0.00            H
ATOM   2253  N   SER   170      14.044  65.617   1.866  1.00 73.74        1SG N
ATOM   2254  CA  SER   170      13.743  64.265   1.474  1.00 73.74        1SG C
ATOM   2255  C   SER   170      12.306  64.046   1.039  1.00 73.74        1SG C
ATOM   2256  O   SER   170      12.026  63.119   0.284  1.00 73.74        1SG O
ATOM   2257  CB  SER   170      14.059  63.366   2.698  1.00 73.74        1SG C
ATOM   2258  OG  SER   170      13.118  63.439   3.759  1.00 73.74        1SG O
ATOM   2259  H   SER   170      14.212  65.825   2.842  1.00  0.00            H
ATOM   2260  HA  SER   170      14.440  63.971   0.699  1.00  0.00            H
ATOM   2261  HB2 SER   170      14.105  62.324   2.377  1.00  0.00            H
ATOM   2262  HB3 SER   170      15.050  63.598   3.092  1.00  0.00            H
ATOM   2263  HG  SER   170      12.812  64.332   3.874  1.00  0.00            H
ATOM   2264  N   LEU   171      11.413  64.949   1.447  1.00105.45        1SG N
ATOM   2265  CA  LEU   171      10.120  65.060   0.832  1.00105.45        1SG C
ATOM   2266  C   LEU   171      10.263  65.430  -0.617  1.00105.45        1SG C
ATOM   2267  O   LEU   171       9.936  64.640  -1.495  1.00105.45        1SG O
ATOM   2268  CB  LEU   171       9.221  66.092   1.538  1.00105.45        1SG C
ATOM   2269  CG  LEU   171       8.432  65.500   2.703  1.00105.45        1SG C
ATOM   2270  CD2 LEU   171       7.264  64.629   2.218  1.00105.45        1SG C
ATOM   2271  CD1 LEU   171       7.917  66.621   3.603  1.00105.45        1SG C
ATOM   2272  H   LEU   171      11.682  65.617   2.157  1.00  0.00            H
```

ATOM	2273	HA	LEU	171	9.647	64.077	0.859	1.00 0.00		H
ATOM	2274	HB2	LEU	171	9.826	66.927	1.878	1.00 0.00		H
ATOM	2275	HB3	LEU	171	8.503	66.527	0.838	1.00 0.00		H
ATOM	2276	HG	LEU	171	9.116	64.882	3.287	1.00 0.00		H
ATOM	2277	HD21	LEU	171	6.780	64.166	3.071	1.00 0.00		H
ATOM	2278	HD22	LEU	171	6.516	65.215	1.684	1.00 0.00		H
ATOM	2279	HD23	LEU	171	7.583	63.822	1.560	1.00 0.00		H
ATOM	2280	HD11	LEU	171	7.214	66.243	4.339	1.00 0.00		H
ATOM	2281	HD12	LEU	171	8.748	67.074	4.140	1.00 0.00		H
ATOM	2282	HD13	LEU	171	7.408	67.402	3.036	1.00 0.00		H
ATOM	2283	N	ILE	172	10.797	66.624	-0.845	1.00126.58	1SG	N
ATOM	2284	CA	ILE	172	10.823	67.209	-2.159	1.00126.58	1SG	C
ATOM	2285	C	ILE	172	11.696	66.452	-3.144	1.00126.58	1SG	C
ATOM	2286	O	ILE	172	11.428	66.480	-4.341	1.00126.58	1SG	O
ATOM	2287	CB	ILE	172	11.160	68.707	-2.007	1.00126.58	1SG	C
ATOM	2288	CG2	ILE	172	12.673	68.899	-2.034	1.00126.58	1SG	C
ATOM	2289	CG1	ILE	172	10.415	69.570	-3.041	1.00126.58	1SG	C
ATOM	2290	CD1	ILE	172	8.918	69.693	-2.786	1.00126.58	1SG	C
ATOM	2291	H	ILE	172	11.128	67.180	-0.065	1.00 0.00		H
ATOM	2292	HA	ILE	172	9.817	67.121	-2.558	1.00 0.00		H
ATOM	2293	HB	ILE	172	10.817	69.058	-1.033	1.00 0.00		H
ATOM	2294	HG21	ILE	172	13.000	69.912	-1.979	1.00 0.00		H
ATOM	2295	HG22	ILE	172	13.106	68.371	-1.197	1.00 0.00		H
ATOM	2296	HG23	ILE	172	13.112	68.529	-2.954	1.00 0.00		H
ATOM	2297	HG12	ILE	172	10.809	70.585	-3.024	1.00 0.00		H
ATOM	2298	HG13	ILE	172	10.564	69.165	-4.042	1.00 0.00		H
ATOM	2299	HD11	ILE	172	8.460	70.276	-3.582	1.00 0.00		H
ATOM	2300	HD12	ILE	172	8.408	68.731	-2.776	1.00 0.00		H
ATOM	2301	HD13	ILE	172	8.733	70.193	-1.835	1.00 0.00		H
ATOM	2302	N	VAL	173	12.729	65.787	-2.619	1.00 92.10	1SG	N
ATOM	2303	CA	VAL	173	13.557	64.961	-3.438	1.00 92.10	1SG	C
ATOM	2304	C	VAL	173	12.786	63.736	-3.936	1.00 92.10	1SG	C
ATOM	2305	O	VAL	173	12.809	63.392	-5.118	1.00 92.10	1SG	O
ATOM	2306	CB	VAL	173	14.900	64.526	-2.731	1.00 92.10	1SG	C
ATOM	2307	CG1	VAL	173	14.820	63.308	-1.796	1.00 92.10	1SG	C
ATOM	2308	CG2	VAL	173	16.040	64.209	-3.711	1.00 92.10	1SG	C
ATOM	2309	H	VAL	173	12.960	65.888	-1.637	1.00 0.00		H
ATOM	2310	HA	VAL	173	13.836	65.550	-4.315	1.00 0.00		H
ATOM	2311	HB	VAL	173	15.259	65.375	-2.155	1.00 0.00		H
ATOM	2312	HG11	VAL	173	15.680	63.262	-1.127	1.00 0.00		H
ATOM	2313	HG12	VAL	173	13.919	63.333	-1.205	1.00 0.00		H
ATOM	2314	HG13	VAL	173	14.795	62.371	-2.354	1.00 0.00		H
ATOM	2315	HG21	VAL	173	16.911	63.803	-3.197	1.00 0.00		H
ATOM	2316	HG22	VAL	173	15.737	63.479	-4.463	1.00 0.00		H
ATOM	2317	HG23	VAL	173	16.386	65.102	-4.215	1.00 0.00		H
ATOM	2318	N	CYS	174	12.072	63.103	-3.006	1.00 75.28	1SG	N
ATOM	2319	CA	CYS	174	11.377	61.867	-3.246	1.00 75.28	1SG	C
ATOM	2320	C	CYS	174	10.133	62.097	-4.071	1.00 75.28	1SG	C
ATOM	2321	O	CYS	174	9.820	61.312	-4.961	1.00 75.28	1SG	O
ATOM	2322	CB	CYS	174	11.021	61.215	-1.892	1.00 75.28	1SG	C
ATOM	2323	SG	CYS	174	10.461	59.496	-2.079	1.00 75.28	1SG	S
ATOM	2324	H	CYS	174	12.033	63.490	-2.073	1.00 0.00		H
ATOM	2325	HA	CYS	174	12.040	61.195	-3.795	1.00 0.00		H
ATOM	2326	HB2	CYS	174	11.909	61.172	-1.264	1.00 0.00		H
ATOM	2327	HB3	CYS	174	10.267	61.791	-1.354	1.00 0.00		H
ATOM	2328	HG	CYS	174	10.307	59.258	-0.773	1.00 0.00		H
ATOM	2329	N	VAL	175	9.489	63.237	-3.845	1.00 96.31	1SG	N
ATOM	2330	CA	VAL	175	8.325	63.609	-4.590	1.00 96.31	1SG	C
ATOM	2331	C	VAL	175	8.640	64.473	-5.826	1.00 96.31	1SG	C
ATOM	2332	O	VAL	175	7.748	65.121	-6.366	1.00 96.31	1SG	O
ATOM	2333	CB	VAL	175	7.216	64.199	-3.660	1.00 96.31	1SG	C
ATOM	2334	CG1	VAL	175	6.986	63.322	-2.409	1.00 96.31	1SG	C
ATOM	2335	CG2	VAL	175	7.327	65.683	-3.256	1.00 96.31	1SG	C

```
ATOM   2336  H   VAL   175       9.789  63.842  -3.085  1.00  0.00           H
ATOM   2337  HA  VAL   175       7.862  62.721  -5.019  1.00  0.00           H
ATOM   2338  HB  VAL   175       6.299  64.141  -4.251  1.00  0.00           H
ATOM   2339 HG11 VAL   175       7.805  63.390  -1.697  1.00  0.00           H
ATOM   2340 HG12 VAL   175       6.085  63.626  -1.882  1.00  0.00           H
ATOM   2341 HG13 VAL   175       6.889  62.268  -2.671  1.00  0.00           H
ATOM   2342 HG21 VAL   175       8.081  65.841  -2.498  1.00  0.00           H
ATOM   2343 HG22 VAL   175       7.551  66.339  -4.097  1.00  0.00           H
ATOM   2344 HG23 VAL   175       6.384  66.030  -2.833  1.00  0.00           H
ATOM   2345  N   LEU   176       9.882  64.413  -6.317  1.00 45.90       1SG N
ATOM   2346  CA  LEU   176      10.181  64.510  -7.743  1.00 45.90       1SG C
ATOM   2347  C   LEU   176      10.348  63.125  -8.308  1.00 45.90       1SG C
ATOM   2348  O   LEU   176       9.794  62.812  -9.354  1.00 45.90       1SG O
ATOM   2349  CB  LEU   176      11.513  65.270  -7.956  1.00 45.90       1SG C
ATOM   2350  CG  LEU   176      11.400  66.807  -7.876  1.00 45.90       1SG C
ATOM   2351  CD2 LEU   176      10.637  67.391  -9.080  1.00 45.90       1SG C
ATOM   2352  CD1 LEU   176      12.797  67.436  -7.789  1.00 45.90       1SG C
ATOM   2353  H   LEU   176      10.566  63.944  -5.742  1.00  0.00           H
ATOM   2354  HA  LEU   176       9.389  65.013  -8.294  1.00  0.00           H
ATOM   2355  HB2 LEU   176      12.252  64.916  -7.236  1.00  0.00           H
ATOM   2356  HB3 LEU   176      11.925  65.018  -8.936  1.00  0.00           H
ATOM   2357  HG  LEU   176      10.861  67.067  -6.964  1.00  0.00           H
ATOM   2358 HD21 LEU   176      10.664  68.480  -9.084  1.00  0.00           H
ATOM   2359 HD22 LEU   176      11.066  67.052 -10.024  1.00  0.00           H
ATOM   2360 HD23 LEU   176       9.587  67.100  -9.073  1.00  0.00           H
ATOM   2361 HD11 LEU   176      12.734  68.521  -7.752  1.00  0.00           H
ATOM   2362 HD12 LEU   176      13.323  67.096  -6.897  1.00  0.00           H
ATOM   2363 HD13 LEU   176      13.403  67.180  -8.657  1.00  0.00           H
ATOM   2364  N   ALA   177      11.080  62.291  -7.578  1.00 31.97       1SG N
ATOM   2365  CA  ALA   177      11.486  60.991  -8.040  1.00 31.97       1SG C
ATOM   2366  C   ALA   177      10.388  59.974  -8.231  1.00 31.97       1SG C
ATOM   2367  O   ALA   177      10.554  59.025  -8.994  1.00 31.97       1SG O
ATOM   2368  CB  ALA   177      12.471  60.416  -7.008  1.00 31.97       1SG C
ATOM   2369  H   ALA   177      11.541  62.644  -6.752  1.00  0.00           H
ATOM   2370  HA  ALA   177      12.000  61.113  -8.995  1.00  0.00           H
ATOM   2371  HB1 ALA   177      12.972  59.532  -7.405  1.00  0.00           H
ATOM   2372  HB2 ALA   177      13.244  61.136  -6.745  1.00  0.00           H
ATOM   2373  HB3 ALA   177      11.971  60.124  -6.084  1.00  0.00           H
ATOM   2374  N   ILE   178       9.275  60.204  -7.548  1.00128.24       1SG N
ATOM   2375  CA  ILE   178       8.077  59.433  -7.709  1.00128.24       1SG C
ATOM   2376  C   ILE   178       6.978  60.227  -8.414  1.00128.24       1SG C
ATOM   2377  O   ILE   178       5.815  59.833  -8.414  1.00128.24       1SG O
ATOM   2378  CB  ILE   178       7.684  58.760  -6.351  1.00128.24       1SG C
ATOM   2379  CG2 ILE   178       6.888  59.672  -5.398  1.00128.24       1SG C
ATOM   2380  CG1 ILE   178       6.927  57.420  -6.523  1.00128.24       1SG C
ATOM   2381  CD1 ILE   178       7.781  56.273  -7.089  1.00128.24       1SG C
ATOM   2382  H   ILE   178       9.301  60.923  -6.833  1.00  0.00           H
ATOM   2383  HA  ILE   178       8.267  58.624  -8.413  1.00  0.00           H
ATOM   2384  HB  ILE   178       8.612  58.518  -5.830  1.00  0.00           H
ATOM   2385 HG21 ILE   178       6.799  59.226  -4.407  1.00  0.00           H
ATOM   2386 HG22 ILE   178       7.376  60.633  -5.275  1.00  0.00           H
ATOM   2387 HG23 ILE   178       5.880  59.857  -5.768  1.00  0.00           H
ATOM   2388 HG12 ILE   178       6.560  57.095  -5.549  1.00  0.00           H
ATOM   2389 HG13 ILE   178       6.038  57.556  -7.139  1.00  0.00           H
ATOM   2390 HD11 ILE   178       7.218  55.339  -7.079  1.00  0.00           H
ATOM   2391 HD12 ILE   178       8.085  56.451  -8.120  1.00  0.00           H
ATOM   2392 HD13 ILE   178       8.682  56.120  -6.495  1.00  0.00           H
ATOM   2393  N   VAL   179       7.369  61.299  -9.102  1.00116.75       1SG N
ATOM   2394  CA  VAL   179       6.549  61.991 -10.068  1.00116.75       1SG C
ATOM   2395  C   VAL   179       7.330  62.259 -11.377  1.00116.75       1SG C
ATOM   2396  O   VAL   179       7.045  63.194 -12.126  1.00116.75       1SG O
ATOM   2397  CB  VAL   179       5.723  63.139  -9.420  1.00116.75       1SG C
ATOM   2398  CG1 VAL   179       6.552  64.402  -9.173  1.00116.75       1SG C
```

```
ATOM   2399  CG2 VAL   179       4.436  63.480 -10.189  1.00116.75       1SG C
ATOM   2400  H   VAL   179       8.347  61.579  -9.075  1.00  0.00           H
ATOM   2401  HA  VAL   179       5.806  61.271 -10.416  1.00  0.00           H
ATOM   2402  HB  VAL   179       5.395  62.783  -8.442  1.00  0.00           H
ATOM   2403 HG11 VAL   179       6.017  65.120  -8.551  1.00  0.00           H
ATOM   2404 HG12 VAL   179       7.464  64.129  -8.656  1.00  0.00           H
ATOM   2405 HG13 VAL   179       6.832  64.902 -10.100  1.00  0.00           H
ATOM   2406 HG21 VAL   179       3.868  64.256  -9.674  1.00  0.00           H
ATOM   2407 HG22 VAL   179       4.652  63.842 -11.194  1.00  0.00           H
ATOM   2408 HG23 VAL   179       3.788  62.608 -10.273  1.00  0.00           H
ATOM   2409  N   ASP   180       8.336  61.418 -11.636  1.00 69.60       1SG N
ATOM   2410  CA  ASP   180       9.381  61.583 -12.644  1.00 69.60       1SG C
ATOM   2411  C   ASP   180       8.963  60.954 -13.996  1.00 69.60       1SG C
ATOM   2412  O   ASP   180       8.262  59.935 -13.992  1.00 69.60       1SG O
ATOM   2413  CB  ASP   180      10.611  60.833 -12.088  1.00 69.60       1SG C
ATOM   2414  CG  ASP   180      11.890  61.079 -12.867  1.00 69.60       1SG C
ATOM   2415  OD1 ASP   180      12.017  60.497 -13.959  1.00 69.60       1SG O
ATOM   2416  OD2 ASP   180      12.759  61.804 -12.347  1.00 69.60       1SG O1-
ATOM   2417  H   ASP   180       8.451  60.633 -11.015  1.00  0.00           H
ATOM   2418  HA  ASP   180       9.611  62.644 -12.742  1.00  0.00           H
ATOM   2419  HB2 ASP   180      10.802  61.134 -11.060  1.00  0.00           H
ATOM   2420  HB3 ASP   180      10.423  59.759 -12.058  1.00  0.00           H
ATOM   2421  N   PRO   181       9.359  61.520 -15.161  1.00136.58       1SG N
ATOM   2422  CA  PRO   181       9.055  60.935 -16.475  1.00136.58       1SG C
ATOM   2423  C   PRO   181       9.947  59.742 -16.886  1.00136.58       1SG C
ATOM   2424  O   PRO   181       9.589  59.059 -17.846  1.00136.58       1SG O
ATOM   2425  CD  PRO   181      10.056  62.803 -15.309  1.00136.58       1SG C
ATOM   2426  CB  PRO   181       9.243  62.101 -17.460  1.00136.58       1SG C
ATOM   2427  CG  PRO   181      10.303  62.976 -16.809  1.00136.58       1SG C
ATOM   2428  HA  PRO   181       8.018  60.597 -16.507  1.00  0.00           H
ATOM   2429  HD3 PRO   181       9.415  63.602 -14.935  1.00  0.00           H
ATOM   2430  HD2 PRO   181      10.998  62.843 -14.762  1.00  0.00           H
ATOM   2431  HB2 PRO   181       9.530  61.784 -18.464  1.00  0.00           H
ATOM   2432  HB3 PRO   181       8.309  62.657 -17.547  1.00  0.00           H
ATOM   2433  HG3 PRO   181      10.265  64.016 -17.133  1.00  0.00           H
ATOM   2434  HG2 PRO   181      11.293  62.590 -17.060  1.00  0.00           H
ATOM   2435  N   TYR   182      11.070  59.478 -16.207  1.00 63.59       1SG N
ATOM   2436  CA  TYR   182      12.051  58.447 -16.538  1.00 63.59       1SG C
ATOM   2437  C   TYR   182      12.161  57.410 -15.406  1.00 63.59       1SG C
ATOM   2438  O   TYR   182      12.144  56.205 -15.664  1.00 63.59       1SG O
ATOM   2439  CB  TYR   182      13.432  59.109 -16.742  1.00 63.59       1SG C
ATOM   2440  CG  TYR   182      13.522  60.181 -17.819  1.00 63.59       1SG C
ATOM   2441  CD1 TYR   182      13.167  59.898 -19.156  1.00 63.59       1SG C
ATOM   2442  CD2 TYR   182      14.002  61.465 -17.486  1.00 63.59       1SG C
ATOM   2443  CE1 TYR   182      13.315  60.885 -20.151  1.00 63.59       1SG C
ATOM   2444  CE2 TYR   182      14.156  62.447 -18.483  1.00 63.59       1SG C
ATOM   2445  CZ  TYR   182      13.828  62.153 -19.820  1.00 63.59       1SG C
ATOM   2446  OH  TYR   182      14.031  63.081 -20.799  1.00 63.59       1SG O
ATOM   2447  H   TYR   182      11.327  60.053 -15.391  1.00  0.00           H
ATOM   2448  HA  TYR   182      11.770  57.918 -17.450  1.00  0.00           H
ATOM   2449  HB2 TYR   182      13.774  59.541 -15.799  1.00  0.00           H
ATOM   2450  HB3 TYR   182      14.166  58.341 -16.991  1.00  0.00           H
ATOM   2451  HD1 TYR   182      12.795  58.922 -19.426  1.00  0.00           H
ATOM   2452  HD2 TYR   182      14.274  61.702 -16.467  1.00  0.00           H
ATOM   2453  HE1 TYR   182      13.062  60.663 -21.177  1.00  0.00           H
ATOM   2454  HE2 TYR   182      14.553  63.419 -18.222  1.00  0.00           H
ATOM   2455  HH  TYR   182      14.814  63.640 -20.612  1.00  0.00           H
ATOM   2456  N   ASN   183      12.272  57.857 -14.148  1.00 73.70       1SG N
ATOM   2457  CA  ASN   183      12.449  57.035 -12.945  1.00 73.70       1SG C
ATOM   2458  C   ASN   183      11.068  56.509 -12.514  1.00 73.70       1SG C
ATOM   2459  O   ASN   183      10.473  56.970 -11.541  1.00 73.70       1SG O
ATOM   2460  CB  ASN   183      13.084  57.891 -11.818  1.00 73.70       1SG C
ATOM   2461  CG  ASN   183      13.451  57.075 -10.574  1.00 73.70       1SG C
```

```
ATOM   2462  OD1 ASN   183      14.345  56.236 -10.604  1.00 73.70       1SG O
ATOM   2463  ND2 ASN   183      12.739  57.267  -9.469  1.00 73.70       1SG N
ATOM   2464  H   ASN   183      12.226  58.882 -14.022  1.00  0.00           H
ATOM   2465  HA  ASN   183      13.107  56.194 -13.169  1.00  0.00           H
ATOM   2466  HB2 ASN   183      13.998  58.361 -12.183  1.00  0.00           H
ATOM   2467  HB3 ASN   183      12.430  58.709 -11.523  1.00  0.00           H
ATOM   2468 HD22 ASN   183      12.978  56.759  -8.638  1.00  0.00           H
ATOM   2469 HD21 ASN   183      11.948  57.917  -9.482  1.00  0.00           H
ATOM   2470  N   ASN   184      10.563  55.521 -13.261  1.00119.08       1SG N
ATOM   2471  CA  ASN   184       9.241  54.883 -13.138  1.00119.08       1SG C
ATOM   2472  C   ASN   184       8.195  55.814 -13.777  1.00119.08       1SG C
ATOM   2473  O   ASN   184       7.450  56.444 -13.025  1.00119.08       1SG O
ATOM   2474  CB  ASN   184       8.861  54.438 -11.696  1.00119.08       1SG C
ATOM   2475  CG  ASN   184       9.875  53.501 -11.047  1.00119.08       1SG C
ATOM   2476  OD1 ASN   184       9.851  52.296 -11.268  1.00119.08       1SG O
ATOM   2477  ND2 ASN   184      10.780  54.047 -10.242  1.00119.08       1SG N
ATOM   2478  H   ASN   184      11.109  55.314 -14.091  1.00  0.00           H
ATOM   2479  HA  ASN   184       9.283  53.968 -13.728  1.00  0.00           H
ATOM   2480  HB2 ASN   184       8.687  55.294 -11.043  1.00  0.00           H
ATOM   2481  HB3 ASN   184       7.906  53.913 -11.729  1.00  0.00           H
ATOM   2482 HD22 ASN   184      11.487  53.463  -9.828  1.00  0.00           H
ATOM   2483 HD21 ASN   184      10.808  55.057 -10.153  1.00  0.00           H
ATOM   2484  N   PRO   185       8.183  55.963 -15.128  1.00124.27       1SG N
ATOM   2485  CA  PRO   185       7.479  57.023 -15.877  1.00124.27       1SG C
ATOM   2486  C   PRO   185       6.062  57.352 -15.380  1.00124.27       1SG C
ATOM   2487  O   PRO   185       5.137  56.575 -15.634  1.00124.27       1SG O
ATOM   2488  CD  PRO   185       8.908  55.095 -16.065  1.00124.27       1SG C
ATOM   2489  CB  PRO   185       7.492  56.556 -17.342  1.00124.27       1SG C
ATOM   2490  CG  PRO   185       8.753  55.720 -17.450  1.00124.27       1SG C
ATOM   2491  HA  PRO   185       8.099  57.914 -15.785  1.00  0.00           H
ATOM   2492  HD3 PRO   185       9.961  54.972 -15.818  1.00  0.00           H
ATOM   2493  HD2 PRO   185       8.448  54.106 -16.049  1.00  0.00           H
ATOM   2494  HB2 PRO   185       6.632  55.925 -17.568  1.00  0.00           H
ATOM   2495  HB3 PRO   185       7.487  57.392 -18.043  1.00  0.00           H
ATOM   2496  HG3 PRO   185       9.605  56.373 -17.646  1.00  0.00           H
ATOM   2497  HG2 PRO   185       8.715  54.981 -18.251  1.00  0.00           H
ATOM   2498  N   VAL   186       5.936  58.493 -14.681  1.00100.89       1SG N
ATOM   2499  CA  VAL   186       4.881  58.943 -13.760  1.00100.89       1SG C
ATOM   2500  C   VAL   186       3.555  58.140 -13.809  1.00100.89       1SG C
ATOM   2501  O   VAL   186       2.857  58.266 -14.819  1.00100.89       1SG O
ATOM   2502  CB  VAL   186       4.750  60.491 -13.817  1.00100.89       1SG C
ATOM   2503  CG1 VAL   186       4.491  61.044 -15.233  1.00100.89       1SG C
ATOM   2504  CG2 VAL   186       3.715  61.043 -12.819  1.00100.89       1SG C
ATOM   2505  H   VAL   186       6.819  58.981 -14.528  1.00  0.00           H
ATOM   2506  HA  VAL   186       5.346  58.775 -12.792  1.00  0.00           H
ATOM   2507  HB  VAL   186       5.715  60.895 -13.506  1.00  0.00           H
ATOM   2508 HG11 VAL   186       4.467  62.134 -15.224  1.00  0.00           H
ATOM   2509 HG12 VAL   186       5.277  60.748 -15.928  1.00  0.00           H
ATOM   2510 HG13 VAL   186       3.540  60.692 -15.633  1.00  0.00           H
ATOM   2511 HG21 VAL   186       3.763  62.131 -12.775  1.00  0.00           H
ATOM   2512 HG22 VAL   186       2.696  60.770 -13.093  1.00  0.00           H
ATOM   2513 HG23 VAL   186       3.898  60.665 -11.813  1.00  0.00           H
ATOM   2514  N   PRO   187       3.230  57.283 -12.807  1.00 93.08       1SG N
ATOM   2515  CA  PRO   187       2.107  56.329 -12.819  1.00 93.08       1SG C
ATOM   2516  C   PRO   187       0.783  56.779 -13.463  1.00 93.08       1SG C
ATOM   2517  O   PRO   187       0.442  56.253 -14.523  1.00 93.08       1SG O
ATOM   2518  CD  PRO   187       3.999  57.111 -11.568  1.00 93.08       1SG C
ATOM   2519  CB  PRO   187       1.952  55.838 -11.377  1.00 93.08       1SG C
ATOM   2520  CG  PRO   187       3.351  55.961 -10.796  1.00 93.08       1SG C
ATOM   2521  HA  PRO   187       2.467  55.485 -13.411  1.00  0.00           H
ATOM   2522  HD3 PRO   187       5.046  56.872 -11.767  1.00  0.00           H
ATOM   2523  HD2 PRO   187       3.966  58.036 -10.993  1.00  0.00           H
ATOM   2524  HB2 PRO   187       1.265  56.466 -10.811  1.00  0.00           H
```

```
ATOM   2525  HB3 PRO   187        1.571  54.818 -11.336  1.00  0.00           H
ATOM   2526  HG3 PRO   187        3.904  55.040 -10.986  1.00  0.00           H
ATOM   2527  HG2 PRO   187        3.357  56.130  -9.719  1.00  0.00           H
ATOM   2528  N   ARG   188        0.093  57.772 -12.886  1.00257.57       1SG N
ATOM   2529  CA  ARG   188       -1.254  58.270 -13.188  1.00257.57       1SG C
ATOM   2530  C   ARG   188       -2.296  57.612 -12.267  1.00257.57       1SG C
ATOM   2531  O   ARG   188       -3.354  57.189 -12.732  1.00257.57       1SG O
ATOM   2532  CB  ARG   188       -1.637  58.332 -14.692  1.00257.57       1SG C
ATOM   2533  CG  ARG   188       -0.721  59.226 -15.554  1.00257.57       1SG C
ATOM   2534  CD  ARG   188       -0.404  58.617 -16.931  1.00257.57       1SG C
ATOM   2535  NE  ARG   188        0.758  57.729 -16.820  1.00257.57       1SG N
ATOM   2536  CZ  ARG   188        1.748  57.498 -17.686  1.00257.57       1SG C
ATOM   2537  NH1 ARG   188        1.672  57.928 -18.947  1.00257.57       1SG N
ATOM   2538  NH2 ARG   188        2.825  56.838 -17.272  1.00257.57       1SG N1+
ATOM   2539  H   ARG   188        0.442  58.127 -11.982  1.00  0.00           H
ATOM   2540  HA  ARG   188       -1.232  59.305 -12.845  1.00  0.00           H
ATOM   2541  HB2 ARG   188       -1.690  57.320 -15.092  1.00  0.00           H
ATOM   2542  HB3 ARG   188       -2.658  58.705 -14.783  1.00  0.00           H
ATOM   2543  HG2 ARG   188       -1.189  60.204 -15.677  1.00  0.00           H
ATOM   2544  HG3 ARG   188        0.213  59.428 -15.026  1.00  0.00           H
ATOM   2545  HD2 ARG   188       -1.260  58.086 -17.349  1.00  0.00           H
ATOM   2546  HD3 ARG   188       -0.153  59.426 -17.617  1.00  0.00           H
ATOM   2547  HE  ARG   188        0.776  57.229 -15.914  1.00  0.00           H
ATOM   2548 HH12 ARG   188        2.426  57.784 -19.601  1.00  0.00           H
ATOM   2549 HH11 ARG   188        0.844  58.404 -19.262  1.00  0.00           H
ATOM   2550 HH22 ARG   188        3.635  56.603 -17.822  1.00  0.00           H
ATOM   2551 HH21 ARG   188        3.015  56.812 -16.264  1.00  0.00           H
ATOM   2552  N   GLY   189       -1.986  57.533 -10.970  1.00212.06       1SG N
ATOM   2553  CA  GLY   189       -2.798  56.911  -9.931  1.00212.06       1SG C
ATOM   2554  C   GLY   189       -2.013  55.836  -9.180  1.00212.06       1SG C
ATOM   2555  O   GLY   189       -2.463  54.696  -9.088  1.00212.06       1SG O
ATOM   2556  H   GLY   189       -1.105  57.969 -10.667  1.00  0.00           H
ATOM   2557  HA2 GLY   189       -3.103  57.680  -9.221  1.00  0.00           H
ATOM   2558  HA3 GLY   189       -3.711  56.471 -10.335  1.00  0.00           H
ATOM   2559  N   LEU   190       -0.839  56.192  -8.645  1.00164.84       1SG N
ATOM   2560  CA  LEU   190       -0.025  55.348  -7.760  1.00164.84       1SG C
ATOM   2561  C   LEU   190        0.829  56.226  -6.875  1.00164.84       1SG C
ATOM   2562  O   LEU   190        0.817  56.135  -5.647  1.00164.84       1SG O
ATOM   2563  CB  LEU   190        0.754  54.259  -8.547  1.00164.84       1SG C
ATOM   2564  CG  LEU   190        0.962  52.901  -7.836  1.00164.84       1SG C
ATOM   2565  CD2 LEU   190       -0.353  52.126  -7.647  1.00164.84       1SG C
ATOM   2566  CD1 LEU   190        1.749  53.004  -6.521  1.00164.84       1SG C
ATOM   2567  H   LEU   190       -0.632  57.198  -8.671  1.00  0.00           H
ATOM   2568  HA  LEU   190       -0.728  54.852  -7.090  1.00  0.00           H
ATOM   2569  HB2 LEU   190        0.241  54.055  -9.487  1.00  0.00           H
ATOM   2570  HB3 LEU   190        1.729  54.648  -8.832  1.00  0.00           H
ATOM   2571  HG  LEU   190        1.573  52.303  -8.514  1.00  0.00           H
ATOM   2572 HD21 LEU   190       -0.164  51.114  -7.287  1.00  0.00           H
ATOM   2573 HD22 LEU   190       -1.013  52.608  -6.927  1.00  0.00           H
ATOM   2574 HD23 LEU   190       -0.901  52.043  -8.587  1.00  0.00           H
ATOM   2575 HD11 LEU   190        2.036  52.017  -6.158  1.00  0.00           H
ATOM   2576 HD12 LEU   190        2.664  53.585  -6.648  1.00  0.00           H
ATOM   2577 HD13 LEU   190        1.158  53.476  -5.737  1.00  0.00           H
ATOM   2578  N   GLU   191        1.503  57.137  -7.561  1.00105.58       1SG N
ATOM   2579  CA  GLU   191        2.268  58.246  -7.061  1.00105.58       1SG C
ATOM   2580  C   GLU   191        1.719  58.974  -5.852  1.00105.58       1SG C
ATOM   2581  O   GLU   191        2.411  59.130  -4.849  1.00105.58       1SG O
ATOM   2582  CB  GLU   191        2.626  59.200  -8.233  1.00105.58       1SG C
ATOM   2583  CG  GLU   191        1.516  60.108  -8.823  1.00105.58       1SG C
ATOM   2584  CD  GLU   191        0.326  59.310  -9.319  1.00105.58       1SG C
ATOM   2585  OE1 GLU   191       -0.566  59.020  -8.495  1.00105.58       1SG O
ATOM   2586  OE2 GLU   191        0.395  58.800 -10.452  1.00105.58       1SG O1-
ATOM   2587  H   GLU   191        1.335  57.148  -8.557  1.00  0.00           H
```

```
ATOM   2588  HA  GLU   191       3.219  57.812  -6.764  1.00  0.00           H
ATOM   2589  HB2 GLU   191       3.403  59.872  -7.871  1.00  0.00           H
ATOM   2590  HB3 GLU   191       3.092  58.630  -9.033  1.00  0.00           H
ATOM   2591  HG2 GLU   191       1.178  60.846  -8.097  1.00  0.00           H
ATOM   2592  HG3 GLU   191       1.920  60.677  -9.660  1.00  0.00           H
ATOM   2593  N   ALA   192       0.461  59.397  -5.939  1.00 44.10       1SG N
ATOM   2594  CA  ALA   192      -0.152  60.222  -4.923  1.00 44.10       1SG C
ATOM   2595  C   ALA   192      -0.347  59.494  -3.613  1.00 44.10       1SG C
ATOM   2596  O   ALA   192      -0.237  60.085  -2.537  1.00 44.10       1SG O
ATOM   2597  CB  ALA   192      -1.494  60.732  -5.451  1.00 44.10       1SG C
ATOM   2598  H   ALA   192      -0.021  59.252  -6.837  1.00  0.00           H
ATOM   2599  HA  ALA   192       0.494  61.083  -4.741  1.00  0.00           H
ATOM   2600  HB1 ALA   192      -1.950  61.411  -4.731  1.00  0.00           H
ATOM   2601  HB2 ALA   192      -1.363  61.285  -6.382  1.00  0.00           H
ATOM   2602  HB3 ALA   192      -2.185  59.913  -5.651  1.00  0.00           H
ATOM   2603  N   PHE   193      -0.581  58.190  -3.712  1.00 49.04       1SG N
ATOM   2604  CA  PHE   193      -0.610  57.300  -2.580  1.00 49.04       1SG C
ATOM   2605  C   PHE   193       0.778  57.115  -1.990  1.00 49.04       1SG C
ATOM   2606  O   PHE   193       0.951  57.232  -0.775  1.00 49.04       1SG O
ATOM   2607  CB  PHE   193      -1.211  55.940  -3.007  1.00 49.04       1SG C
ATOM   2608  CG  PHE   193      -1.444  54.957  -1.872  1.00 49.04       1SG C
ATOM   2609  CD1 PHE   193      -2.485  55.183  -0.947  1.00 49.04       1SG C
ATOM   2610  CD2 PHE   193      -0.615  53.823  -1.730  1.00 49.04       1SG C
ATOM   2611  CE1 PHE   193      -2.698  54.279   0.111  1.00 49.04       1SG C
ATOM   2612  CE2 PHE   193      -0.828  52.921  -0.669  1.00 49.04       1SG C
ATOM   2613  CZ  PHE   193      -1.869  53.149   0.251  1.00 49.04       1SG C
ATOM   2614  H   PHE   193      -0.543  57.786  -4.640  1.00  0.00           H
ATOM   2615  HA  PHE   193      -1.225  57.667  -1.773  1.00  0.00           H
ATOM   2616  HB2 PHE   193      -2.174  56.109  -3.491  1.00  0.00           H
ATOM   2617  HB3 PHE   193      -0.588  55.455  -3.756  1.00  0.00           H
ATOM   2618  HD1 PHE   193      -3.118  56.055  -1.041  1.00  0.00           H
ATOM   2619  HD2 PHE   193       0.189  53.644  -2.429  1.00  0.00           H
ATOM   2620  HE1 PHE   193      -3.486  54.464   0.827  1.00  0.00           H
ATOM   2621  HE2 PHE   193      -0.190  52.057  -0.560  1.00  0.00           H
ATOM   2622  HZ  PHE   193      -2.028  52.464   1.071  1.00  0.00           H
ATOM   2623  N   THR   194       1.780  56.894  -2.842  1.00106.82       1SG N
ATOM   2624  CA  THR   194       3.137  56.722  -2.377  1.00106.82       1SG C
ATOM   2625  C   THR   194       3.786  57.942  -1.778  1.00106.82       1SG C
ATOM   2626  O   THR   194       4.516  57.829  -0.798  1.00106.82       1SG O
ATOM   2627  CB  THR   194       4.011  56.052  -3.465  1.00106.82       1SG C
ATOM   2628  OG1 THR   194       3.941  56.718  -4.703  1.00106.82       1SG O
ATOM   2629  CG2 THR   194       3.615  54.591  -3.720  1.00106.82       1SG C
ATOM   2630  H   THR   194       1.611  56.803  -3.841  1.00  0.00           H
ATOM   2631  HA  THR   194       3.097  56.004  -1.554  1.00  0.00           H
ATOM   2632  HB  THR   194       5.055  56.067  -3.142  1.00  0.00           H
ATOM   2633  HG1 THR   194       4.025  57.655  -4.575  1.00  0.00           H
ATOM   2634 HG21 THR   194       4.256  54.137  -4.477  1.00  0.00           H
ATOM   2635 HG22 THR   194       3.704  53.994  -2.812  1.00  0.00           H
ATOM   2636 HG23 THR   194       2.585  54.514  -4.069  1.00  0.00           H
ATOM   2637  N   VAL   195       3.410  59.108  -2.277  1.00 34.94       1SG N
ATOM   2638  CA  VAL   195       3.723  60.367  -1.663  1.00 34.94       1SG C
ATOM   2639  C   VAL   195       3.200  60.500  -0.241  1.00 34.94       1SG C
ATOM   2640  O   VAL   195       3.889  61.005   0.644  1.00 34.94       1SG O
ATOM   2641  CB  VAL   195       3.183  61.506  -2.589  1.00 34.94       1SG C
ATOM   2642  CG1 VAL   195       3.063  62.897  -1.931  1.00 34.94       1SG C
ATOM   2643  CG2 VAL   195       3.999  61.634  -3.889  1.00 34.94       1SG C
ATOM   2644  H   VAL   195       2.839  59.119  -3.122  1.00  0.00           H
ATOM   2645  HA  VAL   195       4.810  60.458  -1.606  1.00  0.00           H
ATOM   2646  HB  VAL   195       2.169  61.232  -2.885  1.00  0.00           H
ATOM   2647 HG11 VAL   195       2.870  63.671  -2.674  1.00  0.00           H
ATOM   2648 HG12 VAL   195       2.242  62.935  -1.214  1.00  0.00           H
ATOM   2649 HG13 VAL   195       3.973  63.172  -1.401  1.00  0.00           H
ATOM   2650 HG21 VAL   195       3.451  62.198  -4.644  1.00  0.00           H
```

```
ATOM   2651 HG22 VAL   195         4.940  62.150  -3.725  1.00  0.00           H
ATOM   2652 HG23 VAL   195         4.248  60.669  -4.324  1.00  0.00           H
ATOM   2653  N   GLY   196         2.010  59.955  -0.018  1.00 29.15      1SG N
ATOM   2654  CA  GLY   196         1.404  59.955   1.282  1.00 29.15      1SG C
ATOM   2655  C   GLY   196         2.051  58.960   2.227  1.00 29.15      1SG C
ATOM   2656  O   GLY   196         2.164  59.190   3.425  1.00 29.15      1SG O
ATOM   2657  H   GLY   196         1.519  59.513  -0.785  1.00  0.00           H
ATOM   2658  HA2 GLY   196         1.404  60.958   1.707  1.00  0.00           H
ATOM   2659  HA3 GLY   196         0.363  59.661   1.162  1.00  0.00           H
ATOM   2660  N   LEU   197         2.571  57.875   1.680  1.00 94.00      1SG N
ATOM   2661  CA  LEU   197         3.286  56.894   2.460  1.00 94.00      1SG C
ATOM   2662  C   LEU   197         4.724  57.283   2.741  1.00 94.00      1SG C
ATOM   2663  O   LEU   197         5.325  56.828   3.711  1.00 94.00      1SG O
ATOM   2664  CB  LEU   197         3.277  55.575   1.633  1.00 94.00      1SG C
ATOM   2665  CG  LEU   197         3.008  54.285   2.441  1.00 94.00      1SG C
ATOM   2666  CD2 LEU   197         4.189  53.855   3.325  1.00 94.00      1SG C
ATOM   2667  CD1 LEU   197         2.603  53.138   1.501  1.00 94.00      1SG C
ATOM   2668  H   LEU   197         2.436  57.723   0.688  1.00  0.00           H
ATOM   2669  HA  LEU   197         2.758  56.726   3.399  1.00  0.00           H
ATOM   2670  HB2 LEU   197         2.483  55.637   0.887  1.00  0.00           H
ATOM   2671  HB3 LEU   197         4.190  55.466   1.044  1.00  0.00           H
ATOM   2672  HG  LEU   197         2.159  54.471   3.098  1.00  0.00           H
ATOM   2673 HD21 LEU   197         4.005  52.886   3.789  1.00  0.00           H
ATOM   2674 HD22 LEU   197         5.115  53.780   2.753  1.00  0.00           H
ATOM   2675 HD23 LEU   197         4.355  54.558   4.139  1.00  0.00           H
ATOM   2676 HD11 LEU   197         2.363  52.233   2.061  1.00  0.00           H
ATOM   2677 HD12 LEU   197         1.720  53.399   0.916  1.00  0.00           H
ATOM   2678 HD13 LEU   197         3.405  52.894   0.803  1.00  0.00           H
ATOM   2679  N   VAL   198         5.267  58.182   1.934  1.00 30.83      1SG N
ATOM   2680  CA  VAL   198         6.496  58.841   2.266  1.00 30.83      1SG C
ATOM   2681  C   VAL   198         6.337  59.732   3.467  1.00 30.83      1SG C
ATOM   2682  O   VAL   198         7.078  59.592   4.438  1.00 30.83      1SG O
ATOM   2683  CB  VAL   198         7.051  59.592   1.014  1.00 30.83      1SG C
ATOM   2684  CG1 VAL   198         8.166  60.623   1.302  1.00 30.83      1SG C
ATOM   2685  CG2 VAL   198         7.575  58.593  -0.035  1.00 30.83      1SG C
ATOM   2686  H   VAL   198         4.785  58.434   1.078  1.00  0.00           H
ATOM   2687  HA  VAL   198         7.239  58.085   2.512  1.00  0.00           H
ATOM   2688  HB  VAL   198         6.233  60.143   0.555  1.00  0.00           H
ATOM   2689 HG11 VAL   198         8.541  61.063   0.378  1.00  0.00           H
ATOM   2690 HG12 VAL   198         7.814  61.449   1.920  1.00  0.00           H
ATOM   2691 HG13 VAL   198         9.011  60.163   1.816  1.00  0.00           H
ATOM   2692 HG21 VAL   198         7.718  59.078  -1.000  1.00  0.00           H
ATOM   2693 HG22 VAL   198         8.529  58.163   0.270  1.00  0.00           H
ATOM   2694 HG23 VAL   198         6.897  57.757  -0.193  1.00  0.00           H
ATOM   2695  N   VAL   199         5.332  60.603   3.422  1.00114.18      1SG N
ATOM   2696  CA  VAL   199         5.098  61.466   4.547  1.00114.18      1SG C
ATOM   2697  C   VAL   199         4.629  60.768   5.827  1.00114.18      1SG C
ATOM   2698  O   VAL   199         4.887  61.248   6.932  1.00114.18      1SG O
ATOM   2699  CB  VAL   199         4.294  62.744   4.139  1.00114.18      1SG C
ATOM   2700  CG1 VAL   199         2.982  62.512   3.393  1.00114.18      1SG C
ATOM   2701  CG2 VAL   199         4.098  63.760   5.278  1.00114.18      1SG C
ATOM   2702  H   VAL   199         4.753  60.678   2.592  1.00  0.00           H
ATOM   2703  HA  VAL   199         6.076  61.867   4.814  1.00  0.00           H
ATOM   2704  HB  VAL   199         4.916  63.260   3.415  1.00  0.00           H
ATOM   2705 HG11 VAL   199         2.215  63.238   3.663  1.00  0.00           H
ATOM   2706 HG12 VAL   199         3.122  62.601   2.318  1.00  0.00           H
ATOM   2707 HG13 VAL   199         2.611  61.522   3.605  1.00  0.00           H
ATOM   2708 HG21 VAL   199         3.387  63.395   6.020  1.00  0.00           H
ATOM   2709 HG22 VAL   199         5.042  63.965   5.784  1.00  0.00           H
ATOM   2710 HG23 VAL   199         3.721  64.710   4.902  1.00  0.00           H
ATOM   2711  N   LEU   200         4.092  59.561   5.674  1.00143.22      1SG N
ATOM   2712  CA  LEU   200         3.966  58.609   6.750  1.00143.22      1SG C
ATOM   2713  C   LEU   200         5.293  58.258   7.409  1.00143.22      1SG C
```

```
ATOM   2714  O   LEU   200       5.448  58.410   8.625  1.00143.22       1SG O
ATOM   2715  CB  LEU   200       3.214  57.349   6.241  1.00143.22       1SG C
ATOM   2716  CG  LEU   200       2.886  56.168   7.196  1.00143.22       1SG C
ATOM   2717  CD2 LEU   200       4.078  55.306   7.652  1.00143.22       1SG C
ATOM   2718  CD1 LEU   200       1.894  55.233   6.480  1.00143.22       1SG C
ATOM   2719  H   LEU   200       3.859  59.265   4.736  1.00  0.00           H
ATOM   2720  HA  LEU   200       3.362  59.078   7.521  1.00  0.00           H
ATOM   2721  HB2 LEU   200       2.273  57.705   5.817  1.00  0.00           H
ATOM   2722  HB3 LEU   200       3.754  56.918   5.415  1.00  0.00           H
ATOM   2723  HG  LEU   200       2.427  56.563   8.099  1.00  0.00           H
ATOM   2724 HD21 LEU   200       3.733  54.426   8.197  1.00  0.00           H
ATOM   2725 HD22 LEU   200       4.658  54.955   6.802  1.00  0.00           H
ATOM   2726 HD23 LEU   200       4.743  55.827   8.338  1.00  0.00           H
ATOM   2727 HD11 LEU   200       1.573  54.428   7.136  1.00  0.00           H
ATOM   2728 HD12 LEU   200       1.004  55.755   6.129  1.00  0.00           H
ATOM   2729 HD13 LEU   200       2.352  54.771   5.605  1.00  0.00           H
ATOM   2730  N   VAL   201       6.245  57.754   6.619  1.00 43.51       1SG N
ATOM   2731  CA  VAL   201       7.480  57.250   7.183  1.00 43.51       1SG C
ATOM   2732  C   VAL   201       8.447  58.332   7.623  1.00 43.51       1SG C
ATOM   2733  O   VAL   201       9.287  58.075   8.491  1.00 43.51       1SG O
ATOM   2734  CB  VAL   201       8.204  56.251   6.232  1.00 43.51       1SG C
ATOM   2735  CG1 VAL   201       7.380  54.975   5.995  1.00 43.51       1SG C
ATOM   2736  CG2 VAL   201       8.670  56.833   4.890  1.00 43.51       1SG C
ATOM   2737  H   VAL   201       6.090  57.670   5.620  1.00  0.00           H
ATOM   2738  HA  VAL   201       7.210  56.673   8.065  1.00  0.00           H
ATOM   2739  HB  VAL   201       9.109  55.929   6.753  1.00  0.00           H
ATOM   2740 HG11 VAL   201       7.956  54.234   5.440  1.00  0.00           H
ATOM   2741 HG12 VAL   201       7.079  54.516   6.937  1.00  0.00           H
ATOM   2742 HG13 VAL   201       6.480  55.184   5.417  1.00  0.00           H
ATOM   2743 HG21 VAL   201       9.284  56.117   4.343  1.00  0.00           H
ATOM   2744 HG22 VAL   201       7.813  57.062   4.268  1.00  0.00           H
ATOM   2745 HG23 VAL   201       9.259  57.743   5.005  1.00  0.00           H
ATOM   2746  N   ILE   202       8.302  59.549   7.091  1.00 99.55       1SG N
ATOM   2747  CA  ILE   202       9.012  60.652   7.684  1.00 99.55       1SG C
ATOM   2748  C   ILE   202       8.493  60.987   9.065  1.00 99.55       1SG C
ATOM   2749  O   ILE   202       9.261  61.056  10.022  1.00 99.55       1SG O
ATOM   2750  CB  ILE   202       9.136  61.925   6.786  1.00 99.55       1SG C
ATOM   2751  CG2 ILE   202       9.546  61.606   5.334  1.00 99.55       1SG C
ATOM   2752  CG1 ILE   202       7.991  62.962   6.896  1.00 99.55       1SG C
ATOM   2753  CD1 ILE   202       8.152  64.176   5.990  1.00 99.55       1SG C
ATOM   2754  H   ILE   202       7.668  59.694   6.311  1.00  0.00           H
ATOM   2755  HA  ILE   202      10.048  60.327   7.821  1.00  0.00           H
ATOM   2756  HB  ILE   202      10.002  62.447   7.198  1.00  0.00           H
ATOM   2757 HG21 ILE   202      10.060  62.452   4.876  1.00  0.00           H
ATOM   2758 HG22 ILE   202      10.232  60.759   5.288  1.00  0.00           H
ATOM   2759 HG23 ILE   202       8.688  61.383   4.708  1.00  0.00           H
ATOM   2760 HG12 ILE   202       7.036  62.490   6.697  1.00  0.00           H
ATOM   2761 HG13 ILE   202       7.931  63.347   7.914  1.00  0.00           H
ATOM   2762 HD11 ILE   202       7.385  64.916   6.209  1.00  0.00           H
ATOM   2763 HD12 ILE   202       9.126  64.651   6.113  1.00  0.00           H
ATOM   2764 HD13 ILE   202       8.041  63.880   4.951  1.00  0.00           H
ATOM   2765  N   GLY   203       7.172  61.083   9.187  1.00 47.07       1SG N
ATOM   2766  CA  GLY   203       6.554  61.471  10.426  1.00 47.07       1SG C
ATOM   2767  C   GLY   203       6.730  60.440  11.504  1.00 47.07       1SG C
ATOM   2768  O   GLY   203       6.721  60.784  12.678  1.00 47.07       1SG O
ATOM   2769  H   GLY   203       6.577  60.980   8.372  1.00  0.00           H
ATOM   2770  HA2 GLY   203       6.928  62.440  10.759  1.00  0.00           H
ATOM   2771  HA3 GLY   203       5.487  61.574  10.247  1.00  0.00           H
ATOM   2772  N   THR   204       6.927  59.176  11.131  1.00122.93       1SG N
ATOM   2773  CA  THR   204       7.198  58.200  12.146  1.00122.93       1SG C
ATOM   2774  C   THR   204       8.605  58.241  12.725  1.00122.93       1SG C
ATOM   2775  O   THR   204       8.798  57.810  13.862  1.00122.93       1SG O
ATOM   2776  CB  THR   204       6.864  56.753  11.692  1.00122.93       1SG C
```

```
ATOM   2777  OG1 THR   204       6.808  55.958  12.855  1.00122.93      1SG O
ATOM   2778  CG2 THR   204       7.869  56.025  10.795  1.00122.93      1SG C
ATOM   2779  H   THR   204       6.851  58.919  10.154  1.00  0.00          H
ATOM   2780  HA  THR   204       6.541  58.393  12.994  1.00  0.00          H
ATOM   2781  HB  THR   204       5.886  56.745  11.208  1.00  0.00          H
ATOM   2782  HG1 THR   204       7.614  56.112  13.343  1.00  0.00          H
ATOM   2783 HG21 THR   204       7.457  55.089  10.418  1.00  0.00          H
ATOM   2784 HG22 THR   204       8.129  56.636   9.951  1.00  0.00          H
ATOM   2785 HG23 THR   204       8.802  55.788  11.310  1.00  0.00          H
ATOM   2786  N   SER   205       9.574  58.649  11.921  1.00113.94      1SG N
ATOM   2787  CA  SER   205      10.960  58.509  12.296  1.00113.94      1SG C
ATOM   2788  C   SER   205      11.508  59.861  12.748  1.00113.94      1SG C
ATOM   2789  O   SER   205      12.334  59.916  13.655  1.00113.94      1SG O
ATOM   2790  CB  SER   205      11.746  58.118  11.020  1.00113.94      1SG C
ATOM   2791  OG  SER   205      11.177  56.993  10.364  1.00113.94      1SG O
ATOM   2792  H   SER   205       9.349  59.018  11.008  1.00  0.00          H
ATOM   2793  HA  SER   205      11.127  57.759  13.072  1.00  0.00          H
ATOM   2794  HB2 SER   205      11.790  58.938  10.300  1.00  0.00          H
ATOM   2795  HB3 SER   205      12.778  57.882  11.285  1.00  0.00          H
ATOM   2796  HG  SER   205      10.492  57.307   9.777  1.00  0.00          H
ATOM   2797  N   MET   206      11.046  60.947  12.111  1.00145.53      1SG N
ATOM   2798  CA  MET   206      11.316  62.304  12.533  1.00145.53      1SG C
ATOM   2799  C   MET   206      10.408  62.609  13.699  1.00145.53      1SG C
ATOM   2800  O   MET   206      10.875  62.728  14.830  1.00145.53      1SG O
ATOM   2801  CB  MET   206      11.095  63.331  11.386  1.00145.53      1SG C
ATOM   2802  CG  MET   206      12.255  63.449  10.383  1.00145.53      1SG C
ATOM   2803  SD  MET   206      12.446  62.068   9.217  1.00145.53      1SG S
ATOM   2804  CE  MET   206      13.864  61.210   9.953  1.00145.53      1SG C
ATOM   2805  H   MET   206      10.391  60.834  11.340  1.00  0.00          H
ATOM   2806  HA  MET   206      12.346  62.390  12.885  1.00  0.00          H
ATOM   2807  HB2 MET   206      10.158  63.172  10.858  1.00  0.00          H
ATOM   2808  HB3 MET   206      10.985  64.321  11.833  1.00  0.00          H
ATOM   2809  HG2 MET   206      12.084  64.339   9.777  1.00  0.00          H
ATOM   2810  HG3 MET   206      13.197  63.627  10.902  1.00  0.00          H
ATOM   2811  HE1 MET   206      14.079  60.297   9.398  1.00  0.00          H
ATOM   2812  HE2 MET   206      14.749  61.845   9.923  1.00  0.00          H
ATOM   2813  HE3 MET   206      13.667  60.945  10.991  1.00  0.00          H
ATOM   2814  N   GLY   207       9.123  62.685  13.358  1.00 59.98      1SG N
ATOM   2815  CA  GLY   207       7.967  63.220  14.044  1.00 59.98      1SG C
ATOM   2816  C   GLY   207       8.106  63.808  15.428  1.00 59.98      1SG C
ATOM   2817  O   GLY   207       7.711  64.945  15.674  1.00 59.98      1SG O
ATOM   2818  H   GLY   207       8.946  62.431  12.399  1.00  0.00          H
ATOM   2819  HA2 GLY   207       7.545  63.986  13.396  1.00  0.00          H
ATOM   2820  HA3 GLY   207       7.199  62.466  14.161  1.00  0.00          H
ATOM   2821  N   PHE   208       8.654  62.989  16.317  1.00125.65      1SG N
ATOM   2822  CA  PHE   208       9.035  63.270  17.678  1.00125.65      1SG C
ATOM   2823  C   PHE   208       9.768  64.575  17.914  1.00125.65      1SG C
ATOM   2824  O   PHE   208       9.538  65.259  18.910  1.00125.65      1SG O
ATOM   2825  CB  PHE   208       9.828  62.074  18.244  1.00125.65      1SG C
ATOM   2826  CG  PHE   208       9.086  60.748  18.203  1.00125.65      1SG C
ATOM   2827  CD1 PHE   208       9.385  59.796  17.205  1.00125.65      1SG C
ATOM   2828  CD2 PHE   208       8.091  60.465  19.162  1.00125.65      1SG C
ATOM   2829  CE1 PHE   208       8.693  58.570  17.170  1.00125.65      1SG C
ATOM   2830  CE2 PHE   208       7.398  59.239  19.124  1.00125.65      1SG C
ATOM   2831  CZ  PHE   208       7.698  58.291  18.127  1.00125.65      1SG C
ATOM   2832  H   PHE   208       9.018  62.127  15.935  1.00  0.00          H
ATOM   2833  HA  PHE   208       8.103  63.349  18.241  1.00  0.00          H
ATOM   2834  HB2 PHE   208      10.773  61.971  17.707  1.00  0.00          H
ATOM   2835  HB3 PHE   208      10.102  62.276  19.281  1.00  0.00          H
ATOM   2836  HD1 PHE   208      10.147  59.998  16.463  1.00  0.00          H
ATOM   2837  HD2 PHE   208       7.857  61.189  19.931  1.00  0.00          H
ATOM   2838  HE1 PHE   208       8.932  57.844  16.406  1.00  0.00          H
ATOM   2839  HE2 PHE   208       6.639  59.027  19.863  1.00  0.00          H
```

```
ATOM   2840  HZ  PHE   208       7.172  57.348  18.097  1.00  0.00           H
ATOM   2841  N   ASN   209      10.663  64.887  16.988  1.00133.94       1SG N
ATOM   2842  CA  ASN   209      11.458  66.088  17.000  1.00133.94       1SG C
ATOM   2843  C   ASN   209      10.665  67.379  16.899  1.00133.94       1SG C
ATOM   2844  O   ASN   209      10.728  68.192  17.820  1.00133.94       1SG O
ATOM   2845  CB  ASN   209      12.600  66.028  15.948  1.00133.94       1SG C
ATOM   2846  CG  ASN   209      12.208  65.599  14.531  1.00133.94       1SG C
ATOM   2847  OD1 ASN   209      11.040  65.594  14.158  1.00133.94       1SG O
ATOM   2848  ND2 ASN   209      13.174  65.206  13.716  1.00133.94       1SG N
ATOM   2849  H   ASN   209      10.716  64.289  16.167  1.00  0.00           H
ATOM   2850  HA  ASN   209      11.947  66.119  17.975  1.00  0.00           H
ATOM   2851  HB2 ASN   209      13.086  67.004  15.875  1.00  0.00           H
ATOM   2852  HB3 ASN   209      13.363  65.338  16.307  1.00  0.00           H
ATOM   2853 HD22 ASN   209      12.921  65.230  12.740  1.00  0.00           H
ATOM   2854 HD21 ASN   209      14.135  65.274  14.005  1.00  0.00           H
ATOM   2855  N   SER   210       9.941  67.547  15.798  1.00115.78       1SG N
ATOM   2856  CA  SER   210       9.322  68.795  15.422  1.00115.78       1SG C
ATOM   2857  C   SER   210       7.809  68.819  15.602  1.00115.78       1SG C
ATOM   2858  O   SER   210       7.231  69.893  15.759  1.00115.78       1SG O
ATOM   2859  CB  SER   210       9.722  69.128  13.972  1.00115.78       1SG C
ATOM   2860  OG  SER   210       9.697  68.007  13.105  1.00115.78       1SG O
ATOM   2861  H   SER   210      10.051  66.859  15.053  1.00  0.00           H
ATOM   2862  HA  SER   210       9.701  69.606  16.048  1.00  0.00           H
ATOM   2863  HB2 SER   210       9.059  69.892  13.570  1.00  0.00           H
ATOM   2864  HB3 SER   210      10.717  69.562  13.959  1.00  0.00           H
ATOM   2865  HG  SER   210      10.628  67.827  12.871  1.00  0.00           H
ATOM   2866  N   GLY   211       7.153  67.665  15.482  1.00 61.09       1SG N
ATOM   2867  CA  GLY   211       5.736  67.595  15.153  1.00 61.09       1SG C
ATOM   2868  C   GLY   211       5.555  67.629  13.627  1.00 61.09       1SG C
ATOM   2869  O   GLY   211       4.428  67.789  13.167  1.00 61.09       1SG O
ATOM   2870  H   GLY   211       7.676  66.790  15.488  1.00  0.00           H
ATOM   2871  HA2 GLY   211       5.334  66.656  15.532  1.00  0.00           H
ATOM   2872  HA3 GLY   211       5.166  68.400  15.619  1.00  0.00           H
ATOM   2873  N   TYR   212       6.673  67.505  12.883  1.00112.69       1SG N
ATOM   2874  CA  TYR   212       6.956  67.695  11.458  1.00112.69       1SG C
ATOM   2875  C   TYR   212       5.930  68.551  10.696  1.00112.69       1SG C
ATOM   2876  O   TYR   212       4.926  68.069  10.174  1.00112.69       1SG O
ATOM   2877  CB  TYR   212       7.296  66.333  10.800  1.00112.69       1SG C
ATOM   2878  CG  TYR   212       6.151  65.363  10.560  1.00112.69       1SG C
ATOM   2879  CD1 TYR   212       5.427  64.830  11.646  1.00112.69       1SG C
ATOM   2880  CD2 TYR   212       5.781  65.022   9.241  1.00112.69       1SG C
ATOM   2881  CE1 TYR   212       4.311  64.012  11.421  1.00112.69       1SG C
ATOM   2882  CE2 TYR   212       4.682  64.175   9.017  1.00112.69       1SG C
ATOM   2883  CZ  TYR   212       3.942  63.685  10.106  1.00112.69       1SG C
ATOM   2884  OH  TYR   212       2.884  62.866   9.894  1.00112.69       1SG O
ATOM   2885  H   TYR   212       7.522  67.392  13.424  1.00  0.00           H
ATOM   2886  HA  TYR   212       7.882  68.267  11.423  1.00  0.00           H
ATOM   2887  HB2 TYR   212       7.782  66.523   9.844  1.00  0.00           H
ATOM   2888  HB3 TYR   212       8.052  65.824  11.399  1.00  0.00           H
ATOM   2889  HD1 TYR   212       5.692  65.070  12.661  1.00  0.00           H
ATOM   2890  HD2 TYR   212       6.323  65.423   8.398  1.00  0.00           H
ATOM   2891  HE1 TYR   212       3.751  63.641  12.266  1.00  0.00           H
ATOM   2892  HE2 TYR   212       4.398  63.903   8.014  1.00  0.00           H
ATOM   2893  HH  TYR   212       2.482  62.638  10.722  1.00  0.00           H
ATOM   2894  N   ALA   213       6.199  69.864  10.630  1.00 68.41       1SG N
ATOM   2895  CA  ALA   213       5.359  70.797   9.897  1.00 68.41       1SG C
ATOM   2896  C   ALA   213       5.565  70.597   8.397  1.00 68.41       1SG C
ATOM   2897  O   ALA   213       4.578  70.462   7.678  1.00 68.41       1SG O
ATOM   2898  CB  ALA   213       5.709  72.249  10.244  1.00 68.41       1SG C
ATOM   2899  H   ALA   213       7.089  70.192  10.983  1.00  0.00           H
ATOM   2900  HA  ALA   213       4.317  70.610  10.152  1.00  0.00           H
ATOM   2901  HB1 ALA   213       5.141  72.933   9.616  1.00  0.00           H
ATOM   2902  HB2 ALA   213       5.493  72.480  11.287  1.00  0.00           H
```

ATOM	2903	HB3	ALA	213	6.754	72.478	10.052	1.00 0.00		H
ATOM	2904	N	VAL	214	6.836	70.594	7.947	1.00154.17	1SG	N
ATOM	2905	CA	VAL	214	7.362	70.263	6.605	1.00154.17	1SG	C
ATOM	2906	C	VAL	214	6.665	70.997	5.428	1.00154.17	1SG	C
ATOM	2907	O	VAL	214	6.747	70.624	4.256	1.00154.17	1SG	O
ATOM	2908	CB	VAL	214	7.436	68.723	6.413	1.00154.17	1SG	C
ATOM	2909	CG1	VAL	214	8.468	68.090	7.363	1.00154.17	1SG	C
ATOM	2910	CG2	VAL	214	6.088	67.975	6.500	1.00154.17	1SG	C
ATOM	2911	H	VAL	214	7.543	70.718	8.664	1.00 0.00		H
ATOM	2912	HA	VAL	214	8.384	70.641	6.589	1.00 0.00		H
ATOM	2913	HB	VAL	214	7.819	68.575	5.407	1.00 0.00		H
ATOM	2914	HG11	VAL	214	8.553	67.016	7.198	1.00 0.00		H
ATOM	2915	HG12	VAL	214	9.459	68.519	7.209	1.00 0.00		H
ATOM	2916	HG13	VAL	214	8.201	68.251	8.406	1.00 0.00		H
ATOM	2917	HG21	VAL	214	6.192	66.932	6.209	1.00 0.00		H
ATOM	2918	HG22	VAL	214	5.688	67.968	7.513	1.00 0.00		H
ATOM	2919	HG23	VAL	214	5.339	68.420	5.846	1.00 0.00		H
ATOM	2920	N	ASN	215	5.935	72.054	5.770	1.00 85.16	1SG	N
ATOM	2921	CA	ASN	215	4.926	72.730	4.986	1.00 85.16	1SG	C
ATOM	2922	C	ASN	215	4.594	73.978	5.810	1.00 85.16	1SG	C
ATOM	2923	O	ASN	215	3.949	73.838	6.855	1.00 85.16	1SG	O
ATOM	2924	CB	ASN	215	3.694	71.800	4.800	1.00 85.16	1SG	C
ATOM	2925	CG	ASN	215	2.559	72.400	3.967	1.00 85.16	1SG	C
ATOM	2926	OD1	ASN	215	2.381	73.614	3.909	1.00 85.16	1SG	O
ATOM	2927	ND2	ASN	215	1.787	71.565	3.286	1.00 85.16	1SG	N
ATOM	2928	H	ASN	215	5.897	72.232	6.764	1.00 0.00		H
ATOM	2929	HA	ASN	215	5.346	72.975	4.018	1.00 0.00		H
ATOM	2930	HB2	ASN	215	4.013	70.874	4.321	1.00 0.00		H
ATOM	2931	HB3	ASN	215	3.292	71.499	5.762	1.00 0.00		H
ATOM	2932	HD22	ASN	215	1.075	71.947	2.654	1.00 0.00		H
ATOM	2933	HD21	ASN	215	1.917	70.567	3.341	1.00 0.00		H
ATOM	2934	N	PRO	216	5.007	75.189	5.375	1.00 79.12	1SG	N
ATOM	2935	CA	PRO	216	4.657	76.443	6.040	1.00 79.12	1SG	C
ATOM	2936	C	PRO	216	3.145	76.652	6.179	1.00 79.12	1SG	C
ATOM	2937	O	PRO	216	2.717	77.133	7.218	1.00 79.12	1SG	O
ATOM	2938	CD	PRO	216	5.930	75.420	4.269	1.00 79.12	1SG	C
ATOM	2939	CB	PRO	216	5.273	77.565	5.192	1.00 79.12	1SG	C
ATOM	2940	CG	PRO	216	6.338	76.885	4.353	1.00 79.12	1SG	C
ATOM	2941	HA	PRO	216	5.099	76.438	7.035	1.00 0.00		H
ATOM	2942	HD3	PRO	216	6.793	74.758	4.312	1.00 0.00		H
ATOM	2943	HD2	PRO	216	5.408	75.243	3.332	1.00 0.00		H
ATOM	2944	HB2	PRO	216	4.538	78.029	4.537	1.00 0.00		H
ATOM	2945	HB3	PRO	216	5.692	78.358	5.812	1.00 0.00		H
ATOM	2946	HG3	PRO	216	7.294	76.947	4.873	1.00 0.00		H
ATOM	2947	HG2	PRO	216	6.468	77.344	3.378	1.00 0.00		H
ATOM	2948	N	ALA	217	2.322	76.265	5.188	1.00 37.68	1SG	N
ATOM	2949	CA	ALA	217	0.870	76.423	5.255	1.00 37.68	1SG	C
ATOM	2950	C	ALA	217	0.242	75.508	6.312	1.00 37.68	1SG	C
ATOM	2951	O	ALA	217	-0.605	75.991	7.058	1.00 37.68	1SG	O
ATOM	2952	CB	ALA	217	0.219	76.165	3.888	1.00 37.68	1SG	C
ATOM	2953	H	ALA	217	2.700	75.702	4.438	1.00 0.00		H
ATOM	2954	HA	ALA	217	0.650	77.448	5.552	1.00 0.00		H
ATOM	2955	HB1	ALA	217	-0.867	76.210	3.973	1.00 0.00		H
ATOM	2956	HB2	ALA	217	0.512	76.927	3.168	1.00 0.00		H
ATOM	2957	HB3	ALA	217	0.463	75.185	3.480	1.00 0.00		H
ATOM	2958	N	ARG	218	0.707	74.250	6.435	1.00159.09	1SG	N
ATOM	2959	CA	ARG	218	0.249	73.278	7.443	1.00159.09	1SG	C
ATOM	2960	C	ARG	218	0.424	73.796	8.871	1.00159.09	1SG	C
ATOM	2961	O	ARG	218	-0.351	73.415	9.739	1.00159.09	1SG	O
ATOM	2962	CB	ARG	218	0.987	71.919	7.311	1.00159.09	1SG	C
ATOM	2963	CG	ARG	218	0.486	70.779	8.248	1.00159.09	1SG	C
ATOM	2964	CD	ARG	218	1.471	70.301	9.348	1.00159.09	1SG	C
ATOM	2965	NE	ARG	218	1.638	71.270	10.453	1.00159.09	1SG	N

```
ATOM   2966  CZ  ARG   218       0.883  71.392  11.557  1.00159.09      1SG C
ATOM   2967  NH1 ARG   218      -0.139  70.566  11.787  1.00159.09      1SG N
ATOM   2968  NH2 ARG   218       1.129  72.340  12.459  1.00159.09      1SG N1+
ATOM   2969  H   ARG   218       1.429  73.963   5.785  1.00  0.00          H
ATOM   2970  HA  ARG   218      -0.819  73.119   7.278  1.00  0.00          H
ATOM   2971  HB2 ARG   218       0.887  71.574   6.283  1.00  0.00          H
ATOM   2972  HB3 ARG   218       2.052  72.069   7.481  1.00  0.00          H
ATOM   2973  HG2 ARG   218      -0.477  71.034   8.692  1.00  0.00          H
ATOM   2974  HG3 ARG   218       0.269  69.913   7.622  1.00  0.00          H
ATOM   2975  HD2 ARG   218       1.135  69.350   9.763  1.00  0.00          H
ATOM   2976  HD3 ARG   218       2.440  70.082   8.905  1.00  0.00          H
ATOM   2977  HE  ARG   218       2.383  71.939  10.313  1.00  0.00          H
ATOM   2978 HH12 ARG   218      -0.708  70.671  12.620  1.00  0.00          H
ATOM   2979 HH11 ARG   218      -0.323  69.780  11.186  1.00  0.00          H
ATOM   2980 HH22 ARG   218       0.414  72.548  13.169  1.00  0.00          H
ATOM   2981 HH21 ARG   218       1.947  72.981  12.492  1.00  0.00          H
ATOM   2982  N   ASP   219       1.444  74.626   9.119  1.00116.06      1SG N
ATOM   2983  CA  ASP   219       1.701  75.166  10.444  1.00116.06      1SG C
ATOM   2984  C   ASP   219       1.231  76.608  10.572  1.00116.06      1SG C
ATOM   2985  O   ASP   219       0.387  76.884  11.413  1.00116.06      1SG O
ATOM   2986  CB  ASP   219       3.191  75.060  10.784  1.00116.06      1SG C
ATOM   2987  CG  ASP   219       3.374  75.143  12.284  1.00116.06      1SG C
ATOM   2988  OD1 ASP   219       3.811  76.191  12.795  1.00116.06      1SG O
ATOM   2989  OD2 ASP   219       3.047  74.135  12.950  1.00116.06      1SG O1-
ATOM   2990  H   ASP   219       2.033  74.906   8.346  1.00  0.00          H
ATOM   2991  HA  ASP   219       1.136  74.604  11.189  1.00  0.00          H
ATOM   2992  HB2 ASP   219       3.564  74.091  10.469  1.00  0.00          H
ATOM   2993  HB3 ASP   219       3.799  75.813  10.280  1.00  0.00          H
ATOM   2994  N   PHE   220       1.731  77.521   9.731  1.00 87.98      1SG N
ATOM   2995  CA  PHE   220       1.546  78.971   9.827  1.00 87.98      1SG C
ATOM   2996  C   PHE   220       0.103  79.411   9.839  1.00 87.98      1SG C
ATOM   2997  O   PHE   220      -0.280  80.324  10.569  1.00 87.98      1SG O
ATOM   2998  CB  PHE   220       2.293  79.674   8.666  1.00 87.98      1SG C
ATOM   2999  CG  PHE   220       2.305  81.195   8.670  1.00 87.98      1SG C
ATOM   3000  CD1 PHE   220       3.322  81.887   9.354  1.00 87.98      1SG C
ATOM   3001  CD2 PHE   220       1.316  81.923   7.973  1.00 87.98      1SG C
ATOM   3002  CE1 PHE   220       3.362  83.295   9.333  1.00 87.98      1SG C
ATOM   3003  CE2 PHE   220       1.344  83.331   7.967  1.00 87.98      1SG C
ATOM   3004  CZ  PHE   220       2.369  84.018   8.645  1.00 87.98      1SG C
ATOM   3005  H   PHE   220       2.320  77.196   8.967  1.00  0.00          H
ATOM   3006  HA  PHE   220       1.997  79.290  10.768  1.00  0.00          H
ATOM   3007  HB2 PHE   220       3.329  79.333   8.641  1.00  0.00          H
ATOM   3008  HB3 PHE   220       1.854  79.373   7.714  1.00  0.00          H
ATOM   3009  HD1 PHE   220       4.079  81.329   9.884  1.00  0.00          H
ATOM   3010  HD2 PHE   220       0.525  81.407   7.449  1.00  0.00          H
ATOM   3011  HE1 PHE   220       4.154  83.824   9.845  1.00  0.00          H
ATOM   3012  HE2 PHE   220       0.579  83.884   7.441  1.00  0.00          H
ATOM   3013  HZ  PHE   220       2.395  85.098   8.635  1.00  0.00          H
ATOM   3014  N   GLY   221      -0.696  78.706   9.048  1.00 50.12      1SG N
ATOM   3015  CA  GLY   221      -2.088  79.006   8.918  1.00 50.12      1SG C
ATOM   3016  C   GLY   221      -2.867  78.657  10.149  1.00 50.12      1SG C
ATOM   3017  O   GLY   221      -3.416  79.549  10.797  1.00 50.12      1SG O
ATOM   3018  H   GLY   221      -0.320  77.897   8.570  1.00  0.00          H
ATOM   3019  HA2 GLY   221      -2.221  80.067   8.701  1.00  0.00          H
ATOM   3020  HA3 GLY   221      -2.498  78.476   8.059  1.00  0.00          H
ATOM   3021  N   PRO   222      -2.862  77.375  10.519  1.00 74.17      1SG N
ATOM   3022  CA  PRO   222      -3.368  76.969  11.793  1.00 74.17      1SG C
ATOM   3023  C   PRO   222      -2.826  77.631  13.029  1.00 74.17      1SG C
ATOM   3024  O   PRO   222      -3.577  77.871  13.972  1.00 74.17      1SG O
ATOM   3025  CD  PRO   222      -2.536  76.218   9.689  1.00 74.17      1SG C
ATOM   3026  CB  PRO   222      -3.093  75.459  11.883  1.00 74.17      1SG C
ATOM   3027  CG  PRO   222      -3.080  75.008  10.444  1.00 74.17      1SG C
ATOM   3028  HA  PRO   222      -4.448  77.128  11.772  1.00  0.00          H
```

ATOM	3029	HD3	PRO	222	-2.983	76.307	8.710	1.00 0.00		H
ATOM	3030	HD2	PRO	222	-1.458	76.131	9.573	1.00 0.00		H
ATOM	3031	HB2	PRO	222	-2.117	75.249	12.327	1.00 0.00		H
ATOM	3032	HB3	PRO	222	-3.845	74.931	12.470	1.00 0.00		H
ATOM	3033	HG3	PRO	222	-4.099	74.815	10.114	1.00 0.00		H
ATOM	3034	HG2	PRO	222	-2.537	74.083	10.292	1.00 0.00		H
ATOM	3035	N	ARG	223	-1.536	77.945	12.996	1.00103.65	1SG	N
ATOM	3036	CA	ARG	223	-0.883	78.668	14.043	1.00103.65	1SG	C
ATOM	3037	C	ARG	223	-1.503	80.029	14.307	1.00103.65	1SG	C
ATOM	3038	O	ARG	223	-1.731	80.408	15.453	1.00103.65	1SG	O
ATOM	3039	CB	ARG	223	0.644	78.771	13.799	1.00103.65	1SG	C
ATOM	3040	CG	ARG	223	1.447	79.317	15.001	1.00103.65	1SG	C
ATOM	3041	CD	ARG	223	2.968	79.324	14.787	1.00103.65	1SG	C
ATOM	3042	NE	ARG	223	3.508	77.960	14.741	1.00103.65	1SG	N
ATOM	3043	CZ	ARG	223	3.958	77.196	15.738	1.00103.65	1SG	C
ATOM	3044	NH1	ARG	223	3.952	77.576	17.014	1.00103.65	1SG	N
ATOM	3045	NH2	ARG	223	4.403	75.996	15.404	1.00103.65	1SG	N1+
ATOM	3046	H	ARG	223	-0.952	77.605	12.232	1.00 0.00		H
ATOM	3047	HA	ARG	223	-0.980	78.065	14.941	1.00 0.00		H
ATOM	3048	HB2	ARG	223	1.032	77.774	13.596	1.00 0.00		H
ATOM	3049	HB3	ARG	223	0.846	79.374	12.915	1.00 0.00		H
ATOM	3050	HG2	ARG	223	1.125	80.328	15.249	1.00 0.00		H
ATOM	3051	HG3	ARG	223	1.234	78.705	15.876	1.00 0.00		H
ATOM	3052	HD2	ARG	223	3.202	79.827	13.850	1.00 0.00		H
ATOM	3053	HD3	ARG	223	3.472	79.888	15.572	1.00 0.00		H
ATOM	3054	HE	ARG	223	3.550	77.491	13.811	1.00 0.00		H
ATOM	3055	HH12	ARG	223	4.307	76.974	17.742	1.00 0.00		H
ATOM	3056	HH11	ARG	223	3.691	78.530	17.299	1.00 0.00		H
ATOM	3057	HH22	ARG	223	4.681	75.263	16.072	1.00 0.00		H
ATOM	3058	HH21	ARG	223	4.363	75.755	14.400	1.00 0.00		H
ATOM	3059	N	LEU	224	-1.859	80.714	13.226	1.00 98.78	1SG	N
ATOM	3060	CA	LEU	224	-2.558	81.969	13.306	1.00 98.78	1SG	C
ATOM	3061	C	LEU	224	-3.980	81.833	13.800	1.00 98.78	1SG	C
ATOM	3062	O	LEU	224	-4.466	82.696	14.528	1.00 98.78	1SG	O
ATOM	3063	CB	LEU	224	-2.491	82.652	11.912	1.00 98.78	1SG	C
ATOM	3064	CG	LEU	224	-2.734	84.184	11.829	1.00 98.78	1SG	C
ATOM	3065	CD2	LEU	224	-2.046	84.990	12.942	1.00 98.78	1SG	C
ATOM	3066	CD1	LEU	224	-4.212	84.576	11.678	1.00 98.78	1SG	C
ATOM	3067	H	LEU	224	-1.645	80.339	12.309	1.00 0.00		H
ATOM	3068	HA	LEU	224	-2.017	82.582	14.028	1.00 0.00		H
ATOM	3069	HB2	LEU	224	-1.485	82.493	11.522	1.00 0.00		H
ATOM	3070	HB3	LEU	224	-3.146	82.139	11.206	1.00 0.00		H
ATOM	3071	HG	LEU	224	-2.264	84.499	10.896	1.00 0.00		H
ATOM	3072	HD21	LEU	224	-2.097	86.060	12.737	1.00 0.00		H
ATOM	3073	HD22	LEU	224	-2.519	84.829	13.911	1.00 0.00		H
ATOM	3074	HD23	LEU	224	-0.991	84.726	13.029	1.00 0.00		H
ATOM	3075	HD11	LEU	224	-4.316	85.649	11.519	1.00 0.00		H
ATOM	3076	HD12	LEU	224	-4.662	84.080	10.819	1.00 0.00		H
ATOM	3077	HD13	LEU	224	-4.800	84.327	12.560	1.00 0.00		H
ATOM	3078	N	PHE	225	-4.618	80.711	13.479	1.00 54.06	1SG	N
ATOM	3079	CA	PHE	225	-5.926	80.433	14.016	1.00 54.06	1SG	C
ATOM	3080	C	PHE	225	-5.926	80.213	15.505	1.00 54.06	1SG	C
ATOM	3081	O	PHE	225	-6.732	80.795	16.226	1.00 54.06	1SG	O
ATOM	3082	CB	PHE	225	-6.612	79.247	13.288	1.00 54.06	1SG	C
ATOM	3083	CG	PHE	225	-8.131	79.320	13.388	1.00 54.06	1SG	C
ATOM	3084	CD1	PHE	225	-8.881	79.884	12.335	1.00 54.06	1SG	C
ATOM	3085	CD2	PHE	225	-8.794	78.928	14.573	1.00 54.06	1SG	C
ATOM	3086	CE1	PHE	225	-10.260	80.123	12.488	1.00 54.06	1SG	C
ATOM	3087	CE2	PHE	225	-10.171	79.173	14.730	1.00 54.06	1SG	C
ATOM	3088	CZ	PHE	225	-10.904	79.780	13.692	1.00 54.06	1SG	C
ATOM	3089	H	PHE	225	-4.154	80.029	12.896	1.00 0.00		H
ATOM	3090	HA	PHE	225	-6.541	81.316	13.824	1.00 0.00		H
ATOM	3091	HB2	PHE	225	-6.346	79.254	12.230	1.00 0.00		H

ATOM	3092	HB3	PHE	225	-6.272	78.285	13.674	1.00	0.00		H
ATOM	3093	HD1	PHE	225	-8.399	80.168	11.412	1.00	0.00		H
ATOM	3094	HD2	PHE	225	-8.247	78.489	15.395	1.00	0.00		H
ATOM	3095	HE1	PHE	225	-10.820	80.580	11.686	1.00	0.00		H
ATOM	3096	HE2	PHE	225	-10.660	78.909	15.656	1.00	0.00		H
ATOM	3097	HZ	PHE	225	-11.960	79.971	13.821	1.00	0.00		H
ATOM	3098	N	THR	226	-4.981	79.405	15.967	1.00134.65		1SG	N
ATOM	3099	CA	THR	226	-4.921	79.115	17.370	1.00134.65		1SG	C
ATOM	3100	C	THR	226	-4.395	80.254	18.236	1.00134.65		1SG	C
ATOM	3101	O	THR	226	-4.650	80.302	19.442	1.00134.65		1SG	O
ATOM	3102	CB	THR	226	-4.205	77.748	17.602	1.00134.65		1SG	C
ATOM	3103	OG1	THR	226	-4.321	77.260	18.926	1.00134.65		1SG	O
ATOM	3104	CG2	THR	226	-2.712	77.735	17.288	1.00134.65		1SG	C
ATOM	3105	H	THR	226	-4.345	78.942	15.325	1.00	0.00		H
ATOM	3106	HA	THR	226	-5.946	78.947	17.706	1.00	0.00		H
ATOM	3107	HB	THR	226	-4.690	77.010	16.962	1.00	0.00		H
ATOM	3108	HG1	THR	226	-5.232	77.260	19.191	1.00	0.00		H
ATOM	3109	HG21	THR	226	-2.270	76.752	17.454	1.00	0.00		H
ATOM	3110	HG22	THR	226	-2.569	77.995	16.251	1.00	0.00		H
ATOM	3111	HG23	THR	226	-2.165	78.459	17.887	1.00	0.00		H
ATOM	3112	N	ALA	227	-3.782	81.242	17.588	1.00	39.39	1SG	N
ATOM	3113	CA	ALA	227	-3.526	82.509	18.206	1.00	39.39	1SG	C
ATOM	3114	C	ALA	227	-4.793	83.283	18.530	1.00	39.39	1SG	C
ATOM	3115	O	ALA	227	-4.970	83.746	19.657	1.00	39.39	1SG	O
ATOM	3116	CB	ALA	227	-2.612	83.359	17.310	1.00	39.39	1SG	C
ATOM	3117	H	ALA	227	-3.563	81.121	16.607	1.00	0.00		H
ATOM	3118	HA	ALA	227	-3.002	82.314	19.140	1.00	0.00		H
ATOM	3119	HB1	ALA	227	-2.320	84.275	17.825	1.00	0.00		H
ATOM	3120	HB2	ALA	227	-1.699	82.824	17.054	1.00	0.00		H
ATOM	3121	HB3	ALA	227	-3.095	83.657	16.381	1.00	0.00		H
ATOM	3122	N	LEU	228	-5.689	83.345	17.547	1.00	98.69	1SG	N
ATOM	3123	CA	LEU	228	-6.989	83.975	17.658	1.00	98.69	1SG	C
ATOM	3124	C	LEU	228	-7.969	83.200	18.560	1.00	98.69	1SG	C
ATOM	3125	O	LEU	228	-8.943	83.780	19.031	1.00	98.69	1SG	O
ATOM	3126	CB	LEU	228	-7.586	84.136	16.236	1.00	98.69	1SG	C
ATOM	3127	CG	LEU	228	-7.284	85.490	15.551	1.00	98.69	1SG	C
ATOM	3128	CD2	LEU	228	-7.949	85.550	14.167	1.00	98.69	1SG	C
ATOM	3129	CD1	LEU	228	-5.784	85.813	15.433	1.00	98.69	1SG	C
ATOM	3130	H	LEU	228	-5.465	82.902	16.666	1.00	0.00		H
ATOM	3131	HA	LEU	228	-6.872	84.960	18.115	1.00	0.00		H
ATOM	3132	HB2	LEU	228	-7.270	83.313	15.594	1.00	0.00		H
ATOM	3133	HB3	LEU	228	-8.674	84.044	16.287	1.00	0.00		H
ATOM	3134	HG	LEU	228	-7.736	86.272	16.164	1.00	0.00		H
ATOM	3135	HD21	LEU	228	-7.799	86.523	13.698	1.00	0.00		H
ATOM	3136	HD22	LEU	228	-7.539	84.791	13.498	1.00	0.00		H
ATOM	3137	HD23	LEU	228	-9.024	85.385	14.238	1.00	0.00		H
ATOM	3138	HD11	LEU	228	-5.629	86.780	14.955	1.00	0.00		H
ATOM	3139	HD12	LEU	228	-5.301	85.861	16.409	1.00	0.00		H
ATOM	3140	HD13	LEU	228	-5.260	85.068	14.838	1.00	0.00		H
ATOM	3141	N	ALA	229	-7.693	81.920	18.840	1.00	91.25	1SG	N
ATOM	3142	CA	ALA	229	-8.436	81.098	19.792	1.00	91.25	1SG	C
ATOM	3143	C	ALA	229	-8.023	81.375	21.254	1.00	91.25	1SG	C
ATOM	3144	O	ALA	229	-8.667	80.855	22.159	1.00	91.25	1SG	O
ATOM	3145	CB	ALA	229	-8.161	79.618	19.482	1.00	91.25	1SG	C
ATOM	3146	H	ALA	229	-6.924	81.501	18.338	1.00	0.00		H
ATOM	3147	HA	ALA	229	-9.506	81.295	19.694	1.00	0.00		H
ATOM	3148	HB1	ALA	229	-8.774	78.974	20.112	1.00	0.00		H
ATOM	3149	HB2	ALA	229	-8.404	79.379	18.446	1.00	0.00		H
ATOM	3150	HB3	ALA	229	-7.121	79.352	19.665	1.00	0.00		H
ATOM	3151	N	GLY	230	-6.976	82.180	21.493	1.00	50.80	1SG	N
ATOM	3152	CA	GLY	230	-6.617	82.712	22.809	1.00	50.80	1SG	C
ATOM	3153	C	GLY	230	-5.222	82.307	23.292	1.00	50.80	1SG	C
ATOM	3154	O	GLY	230	-4.664	82.966	24.168	1.00	50.80	1SG	O

ATOM	3155	H	GLY	230	-6.506	82.584	20.692	1.00	0.00		H
ATOM	3156	HA2	GLY	230	-6.661	83.800	22.756	1.00	0.00		H
ATOM	3157	HA3	GLY	230	-7.336	82.419	23.576	1.00	0.00		H
ATOM	3158	N	TRP	231	-4.620	81.257	22.719	1.00	132.06	1SG	N
ATOM	3159	CA	TRP	231	-3.345	80.694	23.186	1.00	132.06	1SG	C
ATOM	3160	C	TRP	231	-2.107	81.477	22.689	1.00	132.06	1SG	C
ATOM	3161	O	TRP	231	-0.986	81.145	23.077	1.00	132.06	1SG	O
ATOM	3162	CB	TRP	231	-3.251	79.214	22.751	1.00	132.06	1SG	C
ATOM	3163	CG	TRP	231	-4.022	78.261	23.612	1.00	132.06	1SG	C
ATOM	3164	CD2	TRP	231	-5.438	77.904	23.566	1.00	132.06	1SG	C
ATOM	3165	CD1	TRP	231	-3.496	77.554	24.638	1.00	132.06	1SG	C
ATOM	3166	NE1	TRP	231	-4.493	76.839	25.264	1.00	132.06	1SG	N
ATOM	3167	CE2	TRP	231	-5.710	77.000	24.637	1.00	132.06	1SG	C
ATOM	3168	CE3	TRP	231	-6.532	78.265	22.746	1.00	132.06	1SG	C
ATOM	3169	CZ2	TRP	231	-6.999	76.505	24.899	1.00	132.06	1SG	C
ATOM	3170	CZ3	TRP	231	-7.830	77.769	22.994	1.00	132.06	1SG	C
ATOM	3171	CH2	TRP	231	-8.066	76.893	24.071	1.00	132.06	1SG	C
ATOM	3172	H	TRP	231	-5.120	80.776	21.984	1.00	0.00		H
ATOM	3173	HA	TRP	231	-3.312	80.732	24.277	1.00	0.00		H
ATOM	3174	HB2	TRP	231	-3.542	79.080	21.709	1.00	0.00		H
ATOM	3175	HB3	TRP	231	-2.217	78.878	22.821	1.00	0.00		H
ATOM	3176	HD1	TRP	231	-2.457	77.589	24.937	1.00	0.00		H
ATOM	3177	HE1	TRP	231	-4.358	76.329	26.140	1.00	0.00		H
ATOM	3178	HE3	TRP	231	-6.377	78.953	21.931	1.00	0.00		H
ATOM	3179	HZ2	TRP	231	-7.174	75.851	25.741	1.00	0.00		H
ATOM	3180	HZ3	TRP	231	-8.655	78.086	22.372	1.00	0.00		H
ATOM	3181	HH2	TRP	231	-9.066	76.534	24.271	1.00	0.00		H
ATOM	3182	N	GLY	232	-2.317	82.512	21.860	1.00	38.97	1SG	N
ATOM	3183	CA	GLY	232	-1.411	83.522	21.289	1.00	38.97	1SG	C
ATOM	3184	C	GLY	232	0.029	83.618	21.815	1.00	38.97	1SG	C
ATOM	3185	O	GLY	232	0.975	83.524	21.037	1.00	38.97	1SG	O
ATOM	3186	H	GLY	232	-3.298	82.652	21.654	1.00	0.00		H
ATOM	3187	HA2	GLY	232	-1.351	83.359	20.214	1.00	0.00		H
ATOM	3188	HA3	GLY	232	-1.879	84.499	21.413	1.00	0.00		H
ATOM	3189	N	SER	233	0.195	83.813	23.128	1.00	47.60	1SG	N
ATOM	3190	CA	SER	233	1.486	83.974	23.799	1.00	47.60	1SG	C
ATOM	3191	C	SER	233	2.375	82.716	23.672	1.00	47.60	1SG	C
ATOM	3192	O	SER	233	3.552	82.828	23.330	1.00	47.60	1SG	O
ATOM	3193	CB	SER	233	1.212	84.353	25.275	1.00	47.60	1SG	C
ATOM	3194	OG	SER	233	2.382	84.639	26.017	1.00	47.60	1SG	O
ATOM	3195	H	SER	233	-0.636	83.797	23.701	1.00	0.00		H
ATOM	3196	HA	SER	233	2.011	84.799	23.314	1.00	0.00		H
ATOM	3197	HB2	SER	233	0.565	85.230	25.323	1.00	0.00		H
ATOM	3198	HB3	SER	233	0.682	83.545	25.782	1.00	0.00		H
ATOM	3199	HG	SER	233	2.843	85.368	25.627	1.00	0.00		H
ATOM	3200	N	ALA	234	1.805	81.519	23.873	1.00	58.16	1SG	N
ATOM	3201	CA	ALA	234	2.516	80.242	23.785	1.00	58.16	1SG	C
ATOM	3202	C	ALA	234	2.735	79.851	22.315	1.00	58.16	1SG	C
ATOM	3203	O	ALA	234	3.805	79.376	21.940	1.00	58.16	1SG	O
ATOM	3204	CB	ALA	234	1.647	79.166	24.457	1.00	58.16	1SG	C
ATOM	3205	H	ALA	234	0.802	81.483	24.007	1.00	0.00		H
ATOM	3206	HA	ALA	234	3.480	80.304	24.294	1.00	0.00		H
ATOM	3207	HB1	ALA	234	2.126	78.190	24.406	1.00	0.00		H
ATOM	3208	HB2	ALA	234	1.494	79.391	25.513	1.00	0.00		H
ATOM	3209	HB3	ALA	234	0.665	79.073	23.992	1.00	0.00		H
ATOM	3210	N	VAL	235	1.725	80.130	21.482	1.00	84.56	1SG	N
ATOM	3211	CA	VAL	235	1.628	79.860	20.047	1.00	84.56	1SG	C
ATOM	3212	C	VAL	235	2.785	80.437	19.203	1.00	84.56	1SG	C
ATOM	3213	O	VAL	235	3.091	79.884	18.145	1.00	84.56	1SG	O
ATOM	3214	CB	VAL	235	0.211	80.312	19.595	1.00	84.56	1SG	C
ATOM	3215	CG1	VAL	235	-0.014	80.413	18.083	1.00	84.56	1SG	C
ATOM	3216	CG2	VAL	235	-0.862	79.386	20.189	1.00	84.56	1SG	C
ATOM	3217	H	VAL	235	0.913	80.549	21.912	1.00	0.00		H

ATOM	3218	HA	VAL	235	1.705	78.782	19.920	1.00 0.00		H
ATOM	3219	HB	VAL	235	0.043	81.313	19.987	1.00 0.00		H
ATOM	3220	HG11	VAL	235	-1.033	80.731	17.879	1.00 0.00		H
ATOM	3221	HG12	VAL	235	0.644	81.142	17.608	1.00 0.00		H
ATOM	3222	HG13	VAL	235	0.129	79.447	17.607	1.00 0.00		H
ATOM	3223	HG21	VAL	235	-1.864	79.784	20.032	1.00 0.00		H
ATOM	3224	HG22	VAL	235	-0.821	78.397	19.735	1.00 0.00		H
ATOM	3225	HG23	VAL	235	-0.721	79.241	21.256	1.00 0.00		H
ATOM	3226	N	PHE	236	3.446	81.496	19.680	1.00153.22	1SG	N
ATOM	3227	CA	PHE	236	4.581	82.143	19.020	1.00153.22	1SG	C
ATOM	3228	C	PHE	236	5.841	82.131	19.905	1.00153.22	1SG	C
ATOM	3229	O	PHE	236	6.790	82.866	19.627	1.00153.22	1SG	O
ATOM	3230	CB	PHE	236	4.181	83.569	18.583	1.00153.22	1SG	C
ATOM	3231	CG	PHE	236	2.995	83.652	17.632	1.00153.22	1SG	C
ATOM	3232	CD1	PHE	236	3.030	82.991	16.386	1.00153.22	1SG	C
ATOM	3233	CD2	PHE	236	1.854	84.402	17.985	1.00153.22	1SG	C
ATOM	3234	CE1	PHE	236	1.938	83.082	15.502	1.00153.22	1SG	C
ATOM	3235	CE2	PHE	236	0.763	84.494	17.100	1.00153.22	1SG	C
ATOM	3236	CZ	PHE	236	0.803	83.832	15.858	1.00153.22	1SG	C
ATOM	3237	H	PHE	236	3.108	81.903	20.542	1.00 0.00		H
ATOM	3238	HA	PHE	236	4.869	81.586	18.127	1.00 0.00		H
ATOM	3239	HB2	PHE	236	3.978	84.175	19.468	1.00 0.00		H
ATOM	3240	HB3	PHE	236	5.024	84.049	18.083	1.00 0.00		H
ATOM	3241	HD1	PHE	236	3.899	82.418	16.099	1.00 0.00		H
ATOM	3242	HD2	PHE	236	1.811	84.912	18.937	1.00 0.00		H
ATOM	3243	HE1	PHE	236	1.982	82.586	14.543	1.00 0.00		H
ATOM	3244	HE2	PHE	236	-0.102	85.079	17.375	1.00 0.00		H
ATOM	3245	HZ	PHE	236	-0.033	83.905	15.178	1.00 0.00		H
ATOM	3246	N	THR	237	5.880	81.300	20.956	1.00 79.28	1SG	N
ATOM	3247	CA	THR	237	7.036	81.113	21.826	1.00 79.28	1SG	C
ATOM	3248	C	THR	237	7.325	79.615	21.979	1.00 79.28	1SG	C
ATOM	3249	O	THR	237	7.957	79.028	21.102	1.00 79.28	1SG	O
ATOM	3250	CB	THR	237	6.958	81.956	23.128	1.00 79.28	1SG	C
ATOM	3251	OG1	THR	237	5.870	81.564	23.947	1.00 79.28	1SG	O
ATOM	3252	CG2	THR	237	6.897	83.474	22.905	1.00 79.28	1SG	C
ATOM	3253	H	THR	237	5.098	80.670	21.112	1.00 0.00		H
ATOM	3254	HA	THR	237	7.917	81.488	21.313	1.00 0.00		H
ATOM	3255	HB	THR	237	7.867	81.751	23.698	1.00 0.00		H
ATOM	3256	HG1	THR	237	5.082	82.025	23.645	1.00 0.00		H
ATOM	3257	HG21	THR	237	6.935	84.011	23.853	1.00 0.00		H
ATOM	3258	HG22	THR	237	7.742	83.811	22.304	1.00 0.00		H
ATOM	3259	HG23	THR	237	5.985	83.775	22.390	1.00 0.00		H
ATOM	3260	N	THR	238	6.865	79.032	23.091	1.00211.51	1SG	N
ATOM	3261	CA	THR	238	7.006	77.660	23.588	1.00211.51	1SG	C
ATOM	3262	C	THR	238	8.153	77.517	24.616	1.00211.51	1SG	C
ATOM	3263	O	THR	238	8.336	76.467	25.225	1.00211.51	1SG	O
ATOM	3264	CB	THR	238	6.864	76.569	22.477	1.00211.51	1SG	C
ATOM	3265	OG1	THR	238	6.160	75.441	22.954	1.00211.51	1SG	O
ATOM	3266	CG2	THR	238	8.181	76.027	21.885	1.00211.51	1SG	C
ATOM	3267	H	THR	238	6.331	79.663	23.685	1.00 0.00		H
ATOM	3268	HA	THR	238	6.112	77.563	24.205	1.00 0.00		H
ATOM	3269	HB	THR	238	6.260	76.980	21.668	1.00 0.00		H
ATOM	3270	HG1	THR	238	5.315	75.742	23.289	1.00 0.00		H
ATOM	3271	HG21	THR	238	7.984	75.405	21.013	1.00 0.00		H
ATOM	3272	HG22	THR	238	8.860	76.814	21.566	1.00 0.00		H
ATOM	3273	HG23	THR	238	8.720	75.414	22.608	1.00 0.00		H
ATOM	3274	N	GLY	239	8.927	78.590	24.819	1.00 45.46	1SG	N
ATOM	3275	CA	GLY	239	10.145	78.646	25.629	1.00 45.46	1SG	C
ATOM	3276	C	GLY	239	11.322	79.044	24.736	1.00 45.46	1SG	C
ATOM	3277	O	GLY	239	12.199	79.796	25.150	1.00 45.46	1SG	O
ATOM	3278	H	GLY	239	8.731	79.406	24.257	1.00 0.00		H
ATOM	3279	HA2	GLY	239	10.020	79.387	26.419	1.00 0.00		H
ATOM	3280	HA3	GLY	239	10.370	77.694	26.111	1.00 0.00		H

```
ATOM   3281  N   GLN   240      11.275  78.589  23.480  1.00 99.41      1SG N
ATOM   3282  CA  GLN   240      12.041  79.088  22.344  1.00 99.41      1SG C
ATOM   3283  C   GLN   240      11.144  80.131  21.627  1.00 99.41      1SG C
ATOM   3284  O   GLN   240      10.300  80.768  22.267  1.00 99.41      1SG O
ATOM   3285  CB  GLN   240      12.332  77.867  21.438  1.00 99.41      1SG C
ATOM   3286  CG  GLN   240      13.267  76.829  22.098  1.00 99.41      1SG C
ATOM   3287  CD  GLN   240      13.459  75.569  21.252  1.00 99.41      1SG C
ATOM   3288  OE1 GLN   240      13.303  75.592  20.035  1.00 99.41      1SG O
ATOM   3289  NE2 GLN   240      13.794  74.457  21.893  1.00 99.41      1SG N
ATOM   3290  H   GLN   240      10.484  78.005  23.263  1.00  0.00          H
ATOM   3291  HA  GLN   240      12.968  79.568  22.662  1.00  0.00          H
ATOM   3292  HB2 GLN   240      11.394  77.379  21.175  1.00  0.00          H
ATOM   3293  HB3 GLN   240      12.796  78.183  20.502  1.00  0.00          H
ATOM   3294  HG2 GLN   240      14.246  77.274  22.276  1.00  0.00          H
ATOM   3295  HG3 GLN   240      12.876  76.533  23.071  1.00  0.00          H
ATOM   3296 HE22 GLN   240      14.027  73.613  21.365  1.00  0.00          H
ATOM   3297 HE21 GLN   240      13.929  74.440  22.891  1.00  0.00          H
ATOM   3298  N   HIS   241      11.290  80.316  20.309  1.00237.33      1SG N
ATOM   3299  CA  HIS   241      10.502  81.246  19.499  1.00237.33      1SG C
ATOM   3300  C   HIS   241       9.998  80.521  18.249  1.00237.33      1SG C
ATOM   3301  O   HIS   241      10.371  80.846  17.121  1.00237.33      1SG O
ATOM   3302  ND1 HIS   241       9.170  83.948  18.703  1.00237.33      1SG N
ATOM   3303  CG  HIS   241      10.534  83.700  18.598  1.00237.33      1SG C
ATOM   3304  CB  HIS   241      11.267  82.570  19.293  1.00237.33      1SG C
ATOM   3305  NE2 HIS   241       9.977  85.566  17.420  1.00237.33      1SG N
ATOM   3306  CD2 HIS   241      11.020  84.722  17.812  1.00237.33      1SG C
ATOM   3307  CE1 HIS   241       8.901  85.044  17.995  1.00237.33      1SG C
ATOM   3308  H   HIS   241      11.992  79.777  19.818  1.00  0.00          H
ATOM   3309  HA  HIS   241       9.596  81.513  20.034  1.00  0.00          H
ATOM   3310  HD1 HIS   241       8.445  83.412  19.189  1.00  0.00          H
ATOM   3311  HB2 HIS   241      11.571  82.954  20.267  1.00  0.00          H
ATOM   3312  HB3 HIS   241      12.190  82.380  18.744  1.00  0.00          H
ATOM   3313  HD2 HIS   241      12.039  84.914  17.504  1.00  0.00          H
ATOM   3314  HE1 HIS   241       7.907  85.459  17.892  1.00  0.00          H
ATOM   3315  N   TRP   242       9.178  79.481  18.464  1.00231.13      1SG N
ATOM   3316  CA  TRP   242       8.639  78.639  17.403  1.00231.13      1SG C
ATOM   3317  C   TRP   242       7.549  79.375  16.623  1.00231.13      1SG C
ATOM   3318  O   TRP   242       6.383  79.433  17.009  1.00231.13      1SG O
ATOM   3319  CB  TRP   242       8.129  77.292  17.953  1.00231.13      1SG C
ATOM   3320  CG  TRP   242       9.195  76.246  17.883  1.00231.13      1SG C
ATOM   3321  CD2 TRP   242       9.265  75.040  17.062  1.00231.13      1SG C
ATOM   3322  CD1 TRP   242      10.379  76.330  18.522  1.00231.13      1SG C
ATOM   3323  NE1 TRP   242      11.180  75.273  18.161  1.00231.13      1SG N
ATOM   3324  CE2 TRP   242      10.559  74.457  17.238  1.00231.13      1SG C
ATOM   3325  CE3 TRP   242       8.375  74.374  16.184  1.00231.13      1SG C
ATOM   3326  CZ2 TRP   242      10.955  73.293  16.560  1.00231.13      1SG C
ATOM   3327  CZ3 TRP   242       8.756  73.192  15.516  1.00231.13      1SG C
ATOM   3328  CH2 TRP   242      10.048  72.658  15.694  1.00231.13      1SG C
ATOM   3329  H   TRP   242       8.872  79.287  19.414  1.00  0.00          H
ATOM   3330  HA  TRP   242       9.447  78.416  16.700  1.00  0.00          H
ATOM   3331  HB2 TRP   242       7.760  77.383  18.975  1.00  0.00          H
ATOM   3332  HB3 TRP   242       7.290  76.921  17.363  1.00  0.00          H
ATOM   3333  HD1 TRP   242      10.657  77.139  19.176  1.00  0.00          H
ATOM   3334  HE1 TRP   242      12.108  75.141  18.577  1.00  0.00          H
ATOM   3335  HE3 TRP   242       7.380  74.766  16.030  1.00  0.00          H
ATOM   3336  HZ2 TRP   242      11.936  72.872  16.723  1.00  0.00          H
ATOM   3337  HZ3 TRP   242       8.046  72.689  14.873  1.00  0.00          H
ATOM   3338  HH2 TRP   242      10.347  71.753  15.184  1.00  0.00          H
ATOM   3339  N   TRP   243       7.993  79.932  15.501  1.00189.98      1SG N
ATOM   3340  CA  TRP   243       7.209  80.470  14.409  1.00189.98      1SG C
ATOM   3341  C   TRP   243       8.067  80.318  13.151  1.00189.98      1SG C
ATOM   3342  O   TRP   243       7.654  79.699  12.178  1.00189.98      1SG O
ATOM   3343  CB  TRP   243       6.774  81.919  14.720  1.00189.98      1SG C
```

```
ATOM   3344  CG  TRP   243       5.684  82.527  13.885  1.00189.98       1SG C
ATOM   3345  CD2 TRP   243       5.243  83.918  13.885  1.00189.98       1SG C
ATOM   3346  CD1 TRP   243       4.893  81.891  12.988  1.00189.98       1SG C
ATOM   3347  NE1 TRP   243       3.980  82.783  12.462  1.00189.98       1SG N
ATOM   3348  CE2 TRP   243       4.156  84.052  12.970  1.00189.98       1SG C
ATOM   3349  CE3 TRP   243       5.652  85.086  14.570  1.00189.98       1SG C
ATOM   3350  CZ2 TRP   243       3.508  85.279  12.745  1.00189.98       1SG C
ATOM   3351  CZ3 TRP   243       5.009  86.323  14.357  1.00189.98       1SG C
ATOM   3352  CH2 TRP   243       3.938  86.420  13.446  1.00189.98       1SG C
ATOM   3353  H   TRP   243       8.994  79.906  15.372  1.00  0.00           H
ATOM   3354  HA  TRP   243       6.326  79.838  14.300  1.00  0.00           H
ATOM   3355  HB2 TRP   243       6.425  81.979  15.751  1.00  0.00           H
ATOM   3356  HB3 TRP   243       7.637  82.584  14.665  1.00  0.00           H
ATOM   3357  HD1 TRP   243       4.972  80.845  12.729  1.00  0.00           H
ATOM   3358  HE1 TRP   243       3.306  82.538  11.746  1.00  0.00           H
ATOM   3359  HE3 TRP   243       6.476  85.024  15.265  1.00  0.00           H
ATOM   3360  HZ2 TRP   243       2.686  85.346  12.048  1.00  0.00           H
ATOM   3361  HZ3 TRP   243       5.342  87.199  14.894  1.00  0.00           H
ATOM   3362  HH2 TRP   243       3.448  87.369  13.289  1.00  0.00           H
ATOM   3363  N   TRP   244       9.304  80.829  13.209  1.00133.74       1SG N
ATOM   3364  CA  TRP   244      10.203  80.995  12.065  1.00133.74       1SG C
ATOM   3365  C   TRP   244      10.801  79.686  11.526  1.00133.74       1SG C
ATOM   3366  O   TRP   244      11.087  79.617  10.336  1.00133.74       1SG O
ATOM   3367  CB  TRP   244      11.329  81.963  12.472  1.00133.74       1SG C
ATOM   3368  CG  TRP   244      10.818  83.213  13.124  1.00133.74       1SG C
ATOM   3369  CD2 TRP   244       9.956  84.248  12.557  1.00133.74       1SG C
ATOM   3370  CD1 TRP   244      10.979  83.536  14.426  1.00133.74       1SG C
ATOM   3371  NE1 TRP   244      10.274  84.686  14.702  1.00133.74       1SG N
ATOM   3372  CE2 TRP   244       9.617  85.169  13.593  1.00133.74       1SG C
ATOM   3373  CE3 TRP   244       9.404  84.492  11.277  1.00133.74       1SG C
ATOM   3374  CZ2 TRP   244       8.772  86.271  13.375  1.00133.74       1SG C
ATOM   3375  CZ3 TRP   244       8.548  85.589  11.046  1.00133.74       1SG C
ATOM   3376  CH2 TRP   244       8.231  86.477  12.093  1.00133.74       1SG C
ATOM   3377  H   TRP   244       9.533  81.368  14.033  1.00  0.00           H
ATOM   3378  HA  TRP   244       9.641  81.454  11.254  1.00  0.00           H
ATOM   3379  HB2 TRP   244      12.021  81.470  13.158  1.00  0.00           H
ATOM   3380  HB3 TRP   244      11.917  82.244  11.597  1.00  0.00           H
ATOM   3381  HD1 TRP   244      11.535  82.950  15.144  1.00  0.00           H
ATOM   3382  HE1 TRP   244      10.232  85.087  15.648  1.00  0.00           H
ATOM   3383  HE3 TRP   244       9.635  83.821  10.464  1.00  0.00           H
ATOM   3384  HZ2 TRP   244       8.535  86.945  14.185  1.00  0.00           H
ATOM   3385  HZ3 TRP   244       8.132  85.748  10.062  1.00  0.00           H
ATOM   3386  HH2 TRP   244       7.572  87.314  11.914  1.00  0.00           H
ATOM   3387  N   VAL   245      10.955  78.653  12.364  1.00 54.44       1SG N
ATOM   3388  CA  VAL   245      11.373  77.293  11.986  1.00 54.44       1SG C
ATOM   3389  C   VAL   245      10.458  76.674  10.963  1.00 54.44       1SG C
ATOM   3390  O   VAL   245      10.834  76.606   9.798  1.00 54.44       1SG O
ATOM   3391  CB  VAL   245      11.643  76.470  13.285  1.00 54.44       1SG C
ATOM   3392  CG1 VAL   245      11.757  74.957  13.052  1.00 54.44       1SG C
ATOM   3393  CG2 VAL   245      12.908  76.966  14.009  1.00 54.44       1SG C
ATOM   3394  H   VAL   245      10.688  78.797  13.322  1.00  0.00           H
ATOM   3395  HA  VAL   245      12.336  77.397  11.480  1.00  0.00           H
ATOM   3396  HB  VAL   245      10.826  76.623  13.988  1.00  0.00           H
ATOM   3397 HG11 VAL   245      12.031  74.439  13.971  1.00  0.00           H
ATOM   3398 HG12 VAL   245      10.830  74.508  12.705  1.00  0.00           H
ATOM   3399 HG13 VAL   245      12.518  74.742  12.302  1.00  0.00           H
ATOM   3400 HG21 VAL   245      13.049  76.442  14.955  1.00  0.00           H
ATOM   3401 HG22 VAL   245      13.800  76.781  13.410  1.00  0.00           H
ATOM   3402 HG23 VAL   245      12.869  78.032  14.228  1.00  0.00           H
ATOM   3403  N   PRO   246       9.211  76.342  11.311  1.00 81.34       1SG N
ATOM   3404  CA  PRO   246       8.362  75.762  10.303  1.00 81.34       1SG C
ATOM   3405  C   PRO   246       7.860  76.712   9.223  1.00 81.34       1SG C
ATOM   3406  O   PRO   246       7.201  76.257   8.292  1.00 81.34       1SG O
```

ATOM	3407	CD	PRO	246	8.533	76.454	12.608	1.00	81.34	1SG	C
ATOM	3408	CB	PRO	246	7.181	75.190	11.107	1.00	81.34	1SG	C
ATOM	3409	CG	PRO	246	7.083	76.065	12.343	1.00	81.34	1SG	C
ATOM	3410	HA	PRO	246	8.867	74.934	9.802	1.00	0.00		H
ATOM	3411	HD3	PRO	246	8.950	75.728	13.305	1.00	0.00		H
ATOM	3412	HD2	PRO	246	8.611	77.442	13.055	1.00	0.00		H
ATOM	3413	HB2	PRO	246	6.243	75.148	10.551	1.00	0.00		H
ATOM	3414	HB3	PRO	246	7.419	74.175	11.419	1.00	0.00		H
ATOM	3415	HG3	PRO	246	6.611	75.563	13.189	1.00	0.00		H
ATOM	3416	HG2	PRO	246	6.493	76.956	12.116	1.00	0.00		H
ATOM	3417	N	ILE	247	8.206	77.998	9.313	1.00	81.56	1SG	N
ATOM	3418	CA	ILE	247	8.204	78.848	8.156	1.00	81.56	1SG	C
ATOM	3419	C	ILE	247	9.380	78.540	7.224	1.00	81.56	1SG	C
ATOM	3420	O	ILE	247	9.188	78.073	6.103	1.00	81.56	1SG	O
ATOM	3421	CB	ILE	247	8.147	80.368	8.533	1.00	81.56	1SG	C
ATOM	3422	CG2	ILE	247	8.322	81.314	7.315	1.00	81.56	1SG	C
ATOM	3423	CG1	ILE	247	6.822	80.731	9.241	1.00	81.56	1SG	C
ATOM	3424	CD1	ILE	247	6.848	82.108	9.930	1.00	81.56	1SG	C
ATOM	3425	H	ILE	247	8.707	78.313	10.128	1.00	0.00		H
ATOM	3426	HA	ILE	247	7.301	78.646	7.576	1.00	0.00		H
ATOM	3427	HB	ILE	247	8.963	80.572	9.222	1.00	0.00		H
ATOM	3428	HG21	ILE	247	8.300	82.363	7.605	1.00	0.00		H
ATOM	3429	HG22	ILE	247	9.272	81.177	6.801	1.00	0.00		H
ATOM	3430	HG23	ILE	247	7.532	81.158	6.579	1.00	0.00		H
ATOM	3431	HG12	ILE	247	6.002	80.697	8.523	1.00	0.00		H
ATOM	3432	HG13	ILE	247	6.573	79.976	9.984	1.00	0.00		H
ATOM	3433	HD11	ILE	247	5.956	82.269	10.526	1.00	0.00		H
ATOM	3434	HD12	ILE	247	7.688	82.201	10.613	1.00	0.00		H
ATOM	3435	HD13	ILE	247	6.900	82.930	9.217	1.00	0.00		H
ATOM	3436	N	VAL	248	10.589	78.886	7.658	1.00	67.50	1SG	N
ATOM	3437	CA	VAL	248	11.726	79.005	6.764	1.00	67.50	1SG	C
ATOM	3438	C	VAL	248	12.388	77.675	6.513	1.00	67.50	1SG	C
ATOM	3439	O	VAL	248	12.938	77.440	5.435	1.00	67.50	1SG	O
ATOM	3440	CB	VAL	248	12.743	80.013	7.391	1.00	67.50	1SG	C
ATOM	3441	CG1	VAL	248	13.989	80.260	6.515	1.00	67.50	1SG	C
ATOM	3442	CG2	VAL	248	12.099	81.383	7.698	1.00	67.50	1SG	C
ATOM	3443	H	VAL	248	10.715	79.179	8.623	1.00	0.00		H
ATOM	3444	HA	VAL	248	11.395	79.401	5.800	1.00	0.00		H
ATOM	3445	HB	VAL	248	13.093	79.596	8.339	1.00	0.00		H
ATOM	3446	HG11	VAL	248	14.664	80.981	6.976	1.00	0.00		H
ATOM	3447	HG12	VAL	248	14.563	79.349	6.359	1.00	0.00		H
ATOM	3448	HG13	VAL	248	13.713	80.643	5.531	1.00	0.00		H
ATOM	3449	HG21	VAL	248	12.822	82.064	8.147	1.00	0.00		H
ATOM	3450	HG22	VAL	248	11.724	81.852	6.788	1.00	0.00		H
ATOM	3451	HG23	VAL	248	11.266	81.309	8.395	1.00	0.00		H
ATOM	3452	N	SER	249	12.346	76.825	7.527	1.00	44.82	1SG	N
ATOM	3453	CA	SER	249	13.008	75.566	7.497	1.00	44.82	1SG	C
ATOM	3454	C	SER	249	12.643	74.615	6.395	1.00	44.82	1SG	C
ATOM	3455	O	SER	249	13.544	74.161	5.680	1.00	44.82	1SG	O
ATOM	3456	CB	SER	249	13.069	74.882	8.878	1.00	44.82	1SG	C
ATOM	3457	OG	SER	249	13.620	75.774	9.830	1.00	44.82	1SG	O
ATOM	3458	H	SER	249	11.841	77.050	8.382	1.00	0.00		H
ATOM	3459	HA	SER	249	14.049	75.821	7.282	1.00	0.00		H
ATOM	3460	HB2	SER	249	12.105	74.538	9.238	1.00	0.00		H
ATOM	3461	HB3	SER	249	13.704	73.996	8.826	1.00	0.00		H
ATOM	3462	HG	SER	249	14.523	75.967	9.601	1.00	0.00		H
ATOM	3463	N	PRO	250	11.340	74.396	6.189	1.00	73.41	1SG	N
ATOM	3464	CA	PRO	250	10.900	73.770	4.983	1.00	73.41	1SG	C
ATOM	3465	C	PRO	250	11.397	74.311	3.667	1.00	73.41	1SG	C
ATOM	3466	O	PRO	250	11.796	73.524	2.819	1.00	73.41	1SG	O
ATOM	3467	CD	PRO	250	10.218	74.625	7.111	1.00	73.41	1SG	C
ATOM	3468	CB	PRO	250	9.364	73.880	5.010	1.00	73.41	1SG	C
ATOM	3469	CG	PRO	250	9.002	73.963	6.474	1.00	73.41	1SG	C

```
ATOM   3470  HA  PRO   250      11.188  72.723   5.059  1.00  0.00           H
ATOM   3471  HD3 PRO   250      10.383  74.193   8.094  1.00  0.00           H
ATOM   3472  HD2 PRO   250      10.030  75.690   7.226  1.00  0.00           H
ATOM   3473  HB2 PRO   250       9.032  74.798   4.523  1.00  0.00           H
ATOM   3474  HB3 PRO   250       8.866  73.048   4.512  1.00  0.00           H
ATOM   3475  HG3 PRO   250       8.885  72.957   6.878  1.00  0.00           H
ATOM   3476  HG2 PRO   250       8.072  74.503   6.650  1.00  0.00           H
ATOM   3477  N   LEU   251      11.377  75.632   3.509  1.00 88.51       1SG N
ATOM   3478  CA  LEU   251      11.716  76.265   2.256  1.00 88.51       1SG C
ATOM   3479  C   LEU   251      13.140  76.029   1.819  1.00 88.51       1SG C
ATOM   3480  O   LEU   251      13.418  75.775   0.650  1.00 88.51       1SG O
ATOM   3481  CB  LEU   251      11.465  77.800   2.339  1.00 88.51       1SG C
ATOM   3482  CG  LEU   251      10.197  78.277   1.604  1.00 88.51       1SG C
ATOM   3483  CD2 LEU   251      10.241  79.787   1.328  1.00 88.51       1SG C
ATOM   3484  CD1 LEU   251       8.927  77.913   2.379  1.00 88.51       1SG C
ATOM   3485  H   LEU   251      11.082  76.219   4.275  1.00  0.00           H
ATOM   3486  HA  LEU   251      11.069  75.850   1.485  1.00  0.00           H
ATOM   3487  HB2 LEU   251      11.449  78.143   3.374  1.00  0.00           H
ATOM   3488  HB3 LEU   251      12.306  78.335   1.891  1.00  0.00           H
ATOM   3489  HG  LEU   251      10.163  77.784   0.630  1.00  0.00           H
ATOM   3490 HD21 LEU   251       9.352  80.114   0.787  1.00  0.00           H
ATOM   3491 HD22 LEU   251      10.302  80.362   2.252  1.00  0.00           H
ATOM   3492 HD23 LEU   251      11.103  80.051   0.712  1.00  0.00           H
ATOM   3493 HD11 LEU   251       8.036  78.180   1.809  1.00  0.00           H
ATOM   3494 HD12 LEU   251       8.880  76.846   2.586  1.00  0.00           H
ATOM   3495 HD13 LEU   251       8.883  78.434   3.336  1.00  0.00           H
ATOM   3496  N   LEU   252      14.041  76.093   2.789  1.00135.68       1SG N
ATOM   3497  CA  LEU   252      15.426  75.843   2.512  1.00135.68       1SG C
ATOM   3498  C   LEU   252      15.736  74.380   2.253  1.00135.68       1SG C
ATOM   3499  O   LEU   252      16.622  74.058   1.461  1.00135.68       1SG O
ATOM   3500  CB  LEU   252      16.283  76.363   3.683  1.00135.68       1SG C
ATOM   3501  CG  LEU   252      16.295  77.904   3.814  1.00135.68       1SG C
ATOM   3502  CD2 LEU   252      17.005  78.601   2.640  1.00135.68       1SG C
ATOM   3503  CD1 LEU   252      16.943  78.315   5.137  1.00135.68       1SG C
ATOM   3504  H   LEU   252      13.738  76.316   3.730  1.00  0.00           H
ATOM   3505  HA  LEU   252      15.716  76.371   1.602  1.00  0.00           H
ATOM   3506  HB2 LEU   252      15.919  75.916   4.610  1.00  0.00           H
ATOM   3507  HB3 LEU   252      17.312  76.013   3.572  1.00  0.00           H
ATOM   3508  HG  LEU   252      15.263  78.260   3.841  1.00  0.00           H
ATOM   3509 HD21 LEU   252      17.056  79.679   2.798  1.00  0.00           H
ATOM   3510 HD22 LEU   252      18.026  78.237   2.519  1.00  0.00           H
ATOM   3511 HD23 LEU   252      16.482  78.449   1.696  1.00  0.00           H
ATOM   3512 HD11 LEU   252      16.928  79.396   5.274  1.00  0.00           H
ATOM   3513 HD12 LEU   252      16.392  77.870   5.960  1.00  0.00           H
ATOM   3514 HD13 LEU   252      17.979  77.979   5.202  1.00  0.00           H
ATOM   3515  N   GLY   253      14.930  73.504   2.842  1.00 21.33       1SG N
ATOM   3516  CA  GLY   253      15.045  72.090   2.619  1.00 21.33       1SG C
ATOM   3517  C   GLY   253      14.445  71.693   1.303  1.00 21.33       1SG C
ATOM   3518  O   GLY   253      15.054  70.938   0.552  1.00 21.33       1SG O
ATOM   3519  H   GLY   253      14.186  73.842   3.440  1.00  0.00           H
ATOM   3520  HA2 GLY   253      16.081  71.754   2.686  1.00  0.00           H
ATOM   3521  HA3 GLY   253      14.495  71.579   3.407  1.00  0.00           H
ATOM   3522  N   SER   254      13.305  72.286   0.965  1.00 34.21       1SG N
ATOM   3523  CA  SER   254      12.639  72.021  -0.275  1.00 34.21       1SG C
ATOM   3524  C   SER   254      13.369  72.531  -1.508  1.00 34.21       1SG C
ATOM   3525  O   SER   254      13.063  72.127  -2.620  1.00 34.21       1SG O
ATOM   3526  CB  SER   254      11.215  72.619  -0.255  1.00 34.21       1SG C
ATOM   3527  OG  SER   254      11.200  74.032  -0.205  1.00 34.21       1SG O
ATOM   3528  H   SER   254      12.834  72.917   1.615  1.00  0.00           H
ATOM   3529  HA  SER   254      12.512  70.944  -0.311  1.00  0.00           H
ATOM   3530  HB2 SER   254      10.669  72.304  -1.147  1.00  0.00           H
ATOM   3531  HB3 SER   254      10.645  72.243   0.590  1.00  0.00           H
ATOM   3532  HG  SER   254      11.660  74.381  -0.957  1.00  0.00           H
```

ATOM	3533	N	ILE	255	14.329	73.426	-1.329	1.00	40.62	1SG	N
ATOM	3534	CA	ILE	255	15.190	73.801	-2.417	1.00	40.62	1SG	C
ATOM	3535	C	ILE	255	16.367	72.869	-2.514	1.00	40.62	1SG	C
ATOM	3536	O	ILE	255	16.687	72.389	-3.599	1.00	40.62	1SG	O
ATOM	3537	CB	ILE	255	15.671	75.275	-2.194	1.00	40.62	1SG	C
ATOM	3538	CG2	ILE	255	16.797	75.724	-3.158	1.00	40.62	1SG	C
ATOM	3539	CG1	ILE	255	14.491	76.273	-2.308	1.00	40.62	1SG	C
ATOM	3540	CD1	ILE	255	14.770	77.627	-1.636	1.00	40.62	1SG	C
ATOM	3541	H	ILE	255	14.484	73.807	-0.405	1.00	0.00		H
ATOM	3542	HA	ILE	255	14.667	73.777	-3.377	1.00	0.00		H
ATOM	3543	HB	ILE	255	16.070	75.341	-1.180	1.00	0.00		H
ATOM	3544	HG21	ILE	255	17.068	76.768	-3.005	1.00	0.00		H
ATOM	3545	HG22	ILE	255	17.716	75.154	-3.018	1.00	0.00		H
ATOM	3546	HG23	ILE	255	16.495	75.613	-4.200	1.00	0.00		H
ATOM	3547	HG12	ILE	255	14.227	76.427	-3.355	1.00	0.00		H
ATOM	3548	HG13	ILE	255	13.595	75.856	-1.852	1.00	0.00		H
ATOM	3549	HD11	ILE	255	13.859	78.219	-1.572	1.00	0.00		H
ATOM	3550	HD12	ILE	255	15.135	77.496	-0.617	1.00	0.00		H
ATOM	3551	HD13	ILE	255	15.507	78.213	-2.185	1.00	0.00		H
ATOM	3552	N	ALA	256	16.981	72.600	-1.366	1.00	27.32	1SG	N
ATOM	3553	CA	ALA	256	18.124	71.733	-1.287	1.00	27.32	1SG	C
ATOM	3554	C	ALA	256	17.837	70.320	-1.737	1.00	27.32	1SG	C
ATOM	3555	O	ALA	256	18.626	69.723	-2.458	1.00	27.32	1SG	O
ATOM	3556	CB	ALA	256	18.641	71.723	0.161	1.00	27.32	1SG	C
ATOM	3557	H	ALA	256	16.655	73.035	-0.511	1.00	0.00		H
ATOM	3558	HA	ALA	256	18.907	72.138	-1.932	1.00	0.00		H
ATOM	3559	HB1	ALA	256	19.521	71.085	0.258	1.00	0.00		H
ATOM	3560	HB2	ALA	256	18.928	72.723	0.484	1.00	0.00		H
ATOM	3561	HB3	ALA	256	17.889	71.353	0.859	1.00	0.00		H
ATOM	3562	N	GLY	257	16.641	69.857	-1.403	1.00	22.24	1SG	N
ATOM	3563	CA	GLY	257	16.151	68.581	-1.819	1.00	22.24	1SG	C
ATOM	3564	C	GLY	257	15.835	68.505	-3.288	1.00	22.24	1SG	C
ATOM	3565	O	GLY	257	16.141	67.507	-3.933	1.00	22.24	1SG	O
ATOM	3566	H	GLY	257	16.063	70.411	-0.772	1.00	0.00		H
ATOM	3567	HA2	GLY	257	16.811	67.777	-1.501	1.00	0.00		H
ATOM	3568	HA3	GLY	257	15.229	68.395	-1.295	1.00	0.00		H
ATOM	3569	N	VAL	258	15.263	69.583	-3.829	1.00	41.72	1SG	N
ATOM	3570	CA	VAL	258	14.980	69.637	-5.241	1.00	41.72	1SG	C
ATOM	3571	C	VAL	258	16.254	69.663	-6.075	1.00	41.72	1SG	C
ATOM	3572	O	VAL	258	16.368	68.966	-7.087	1.00	41.72	1SG	O
ATOM	3573	CB	VAL	258	14.021	70.804	-5.609	1.00	41.72	1SG	C
ATOM	3574	CG1	VAL	258	14.078	71.291	-7.063	1.00	41.72	1SG	C
ATOM	3575	CG2	VAL	258	12.591	70.369	-5.324	1.00	41.72	1SG	C
ATOM	3576	H	VAL	258	15.079	70.397	-3.263	1.00	0.00		H
ATOM	3577	HA	VAL	258	14.490	68.700	-5.518	1.00	0.00		H
ATOM	3578	HB	VAL	258	14.257	71.669	-4.987	1.00	0.00		H
ATOM	3579	HG11	VAL	258	13.298	72.028	-7.257	1.00	0.00		H
ATOM	3580	HG12	VAL	258	15.036	71.766	-7.273	1.00	0.00		H
ATOM	3581	HG13	VAL	258	13.943	70.476	-7.774	1.00	0.00		H
ATOM	3582	HG21	VAL	258	12.359	69.379	-5.715	1.00	0.00		H
ATOM	3583	HG22	VAL	258	12.491	70.328	-4.255	1.00	0.00		H
ATOM	3584	HG23	VAL	258	11.843	71.050	-5.722	1.00	0.00		H
ATOM	3585	N	PHE	259	17.211	70.451	-5.596	1.00	49.41	1SG	N
ATOM	3586	CA	PHE	259	18.537	70.542	-6.142	1.00	49.41	1SG	C
ATOM	3587	C	PHE	259	19.229	69.193	-6.187	1.00	49.41	1SG	C
ATOM	3588	O	PHE	259	19.686	68.765	-7.248	1.00	49.41	1SG	O
ATOM	3589	CB	PHE	259	19.335	71.600	-5.350	1.00	49.41	1SG	C
ATOM	3590	CG	PHE	259	20.695	71.967	-5.916	1.00	49.41	1SG	C
ATOM	3591	CD1	PHE	259	20.789	72.844	-7.018	1.00	49.41	1SG	C
ATOM	3592	CD2	PHE	259	21.870	71.445	-5.334	1.00	49.41	1SG	C
ATOM	3593	CE1	PHE	259	22.051	73.200	-7.531	1.00	49.41	1SG	C
ATOM	3594	CE2	PHE	259	23.131	71.805	-5.846	1.00	49.41	1SG	C
ATOM	3595	CZ	PHE	259	23.222	72.684	-6.942	1.00	49.41	1SG	C

ATOM	3596	H	PHE	259	17.004	71.001	-4.767	1.00	0.00		H
ATOM	3597	HA	PHE	259	18.439	70.891	-7.172	1.00	0.00		H
ATOM	3598	HB2	PHE	259	18.756	72.522	-5.299	1.00	0.00		H
ATOM	3599	HB3	PHE	259	19.464	71.281	-4.317	1.00	0.00		H
ATOM	3600	HD1	PHE	259	19.896	73.243	-7.475	1.00	0.00		H
ATOM	3601	HD2	PHE	259	21.810	70.766	-4.495	1.00	0.00		H
ATOM	3602	HE1	PHE	259	22.123	73.866	-8.378	1.00	0.00		H
ATOM	3603	HE2	PHE	259	24.029	71.402	-5.401	1.00	0.00		H
ATOM	3604	HZ	PHE	259	24.190	72.953	-7.339	1.00	0.00		H
ATOM	3605	N	VAL	260	19.229	68.489	-5.053	1.00	95.13	1SG	N
ATOM	3606	CA	VAL	260	19.931	67.235	-4.995	1.00	95.13	1SG	C
ATOM	3607	C	VAL	260	19.285	66.059	-5.693	1.00	95.13	1SG	C
ATOM	3608	O	VAL	260	19.983	65.084	-5.959	1.00	95.13	1SG	O
ATOM	3609	CB	VAL	260	20.378	66.838	-3.560	1.00	95.13	1SG	C
ATOM	3610	CG1	VAL	260	21.345	67.869	-2.946	1.00	95.13	1SG	C
ATOM	3611	CG2	VAL	260	19.230	66.472	-2.612	1.00	95.13	1SG	C
ATOM	3612	H	VAL	260	18.838	68.870	-4.197	1.00	0.00		H
ATOM	3613	HA	VAL	260	20.871	67.373	-5.538	1.00	0.00		H
ATOM	3614	HB	VAL	260	20.971	65.926	-3.655	1.00	0.00		H
ATOM	3615	HG11	VAL	260	21.690	67.547	-1.963	1.00	0.00		H
ATOM	3616	HG12	VAL	260	22.228	67.991	-3.574	1.00	0.00		H
ATOM	3617	HG13	VAL	260	20.894	68.851	-2.833	1.00	0.00		H
ATOM	3618	HG21	VAL	260	19.586	66.278	-1.600	1.00	0.00		H
ATOM	3619	HG22	VAL	260	18.516	67.278	-2.574	1.00	0.00		H
ATOM	3620	HG23	VAL	260	18.698	65.582	-2.948	1.00	0.00		H
ATOM	3621	N	TYR	261	18.012	66.159	-6.072	1.00	58.41	1SG	N
ATOM	3622	CA	TYR	261	17.486	65.172	-6.986	1.00	58.41	1SG	C
ATOM	3623	C	TYR	261	18.047	65.379	-8.377	1.00	58.41	1SG	C
ATOM	3624	O	TYR	261	18.579	64.466	-9.004	1.00	58.41	1SG	O
ATOM	3625	CB	TYR	261	15.937	65.226	-7.092	1.00	58.41	1SG	C
ATOM	3626	CG	TYR	261	15.382	64.097	-7.946	1.00	58.41	1SG	C
ATOM	3627	CD1	TYR	261	15.631	62.755	-7.582	1.00	58.41	1SG	C
ATOM	3628	CD2	TYR	261	14.706	64.375	-9.151	1.00	58.41	1SG	C
ATOM	3629	CE1	TYR	261	15.253	61.707	-8.441	1.00	58.41	1SG	C
ATOM	3630	CE2	TYR	261	14.293	63.323	-9.989	1.00	58.41	1SG	C
ATOM	3631	CZ	TYR	261	14.587	61.990	-9.644	1.00	58.41	1SG	C
ATOM	3632	OH	TYR	261	14.228	60.967	-10.463	1.00	58.41	1SG	O
ATOM	3633	H	TYR	261	17.462	66.965	-5.804	1.00	0.00		H
ATOM	3634	HA	TYR	261	17.770	64.175	-6.641	1.00	0.00		H
ATOM	3635	HB2	TYR	261	15.453	65.158	-6.125	1.00	0.00		H
ATOM	3636	HB3	TYR	261	15.610	66.184	-7.498	1.00	0.00		H
ATOM	3637	HD1	TYR	261	16.148	62.514	-6.666	1.00	0.00		H
ATOM	3638	HD2	TYR	261	14.517	65.390	-9.464	1.00	0.00		H
ATOM	3639	HE1	TYR	261	15.469	60.681	-8.183	1.00	0.00		H
ATOM	3640	HE2	TYR	261	13.778	63.541	-10.914	1.00	0.00		H
ATOM	3641	HH	TYR	261	13.706	61.278	-11.235	1.00	0.00		H
ATOM	3642	N	GLN	262	17.923	66.621	-8.829	1.00	91.53	1SG	N
ATOM	3643	CA	GLN	262	18.124	66.999	-10.202	1.00	91.53	1SG	C
ATOM	3644	C	GLN	262	19.556	66.877	-10.665	1.00	91.53	1SG	C
ATOM	3645	O	GLN	262	19.813	66.684	-11.847	1.00	91.53	1SG	O
ATOM	3646	CB	GLN	262	17.575	68.436	-10.329	1.00	91.53	1SG	C
ATOM	3647	CG	GLN	262	17.501	68.967	-11.776	1.00	91.53	1SG	C
ATOM	3648	CD	GLN	262	16.395	69.984	-12.066	1.00	91.53	1SG	C
ATOM	3649	OE1	GLN	262	16.432	70.660	-13.088	1.00	91.53	1SG	O
ATOM	3650	NE2	GLN	262	15.395	70.138	-11.204	1.00	91.53	1SG	N
ATOM	3651	H	GLN	262	17.559	67.322	-8.198	1.00	0.00		H
ATOM	3652	HA	GLN	262	17.517	66.338	-10.823	1.00	0.00		H
ATOM	3653	HB2	GLN	262	16.585	68.420	-9.876	1.00	0.00		H
ATOM	3654	HB3	GLN	262	18.168	69.124	-9.724	1.00	0.00		H
ATOM	3655	HG2	GLN	262	18.466	69.369	-12.089	1.00	0.00		H
ATOM	3656	HG3	GLN	262	17.297	68.127	-12.437	1.00	0.00		H
ATOM	3657	HE22	GLN	262	14.632	70.779	-11.419	1.00	0.00		H
ATOM	3658	HE21	GLN	262	15.346	69.545	-10.392	1.00	0.00		H

ATOM	3659	N	LEU	263	20.472	66.954	-9.709	1.00	98.25	1SG	N
ATOM	3660	CA	LEU	263	21.863	66.821	-10.013	1.00	98.25	1SG	C
ATOM	3661	C	LEU	263	22.448	65.454	-9.804	1.00	98.25	1SG	C
ATOM	3662	O	LEU	263	23.410	65.095	-10.481	1.00	98.25	1SG	O
ATOM	3663	CB	LEU	263	22.665	67.814	-9.124	1.00	98.25	1SG	C
ATOM	3664	CG	LEU	263	22.822	69.233	-9.723	1.00	98.25	1SG	C
ATOM	3665	CD2	LEU	263	24.020	69.957	-9.091	1.00	98.25	1SG	C
ATOM	3666	CD1	LEU	263	21.557	70.096	-9.591	1.00	98.25	1SG	C
ATOM	3667	H	LEU	263	20.180	67.205	-8.775	1.00	0.00		H
ATOM	3668	HA	LEU	263	22.071	67.070	-11.057	1.00	0.00		H
ATOM	3669	HB2	LEU	263	22.249	67.865	-8.116	1.00	0.00		H
ATOM	3670	HB3	LEU	263	23.678	67.427	-8.986	1.00	0.00		H
ATOM	3671	HG	LEU	263	23.044	69.130	-10.786	1.00	0.00		H
ATOM	3672	HD21	LEU	263	24.137	70.962	-9.498	1.00	0.00		H
ATOM	3673	HD22	LEU	263	23.901	70.045	-8.011	1.00	0.00		H
ATOM	3674	HD23	LEU	263	24.951	69.421	-9.279	1.00	0.00		H
ATOM	3675	HD11	LEU	263	21.696	71.068	-10.065	1.00	0.00		H
ATOM	3676	HD12	LEU	263	20.695	69.629	-10.065	1.00	0.00		H
ATOM	3677	HD13	LEU	263	21.312	70.278	-8.545	1.00	0.00		H
ATOM	3678	N	MET	264	21.916	64.716	-8.839	1.00	96.83	1SG	N
ATOM	3679	CA	MET	264	22.444	63.402	-8.596	1.00	96.83	1SG	C
ATOM	3680	C	MET	264	21.771	62.394	-9.564	1.00	96.83	1SG	C
ATOM	3681	O	MET	264	22.423	61.437	-9.980	1.00	96.83	1SG	O
ATOM	3682	CB	MET	264	22.131	62.966	-7.151	1.00	96.83	1SG	C
ATOM	3683	CG	MET	264	22.800	63.828	-6.064	1.00	96.83	1SG	C
ATOM	3684	SD	MET	264	24.602	63.660	-5.897	1.00	96.83	1SG	S
ATOM	3685	CE	MET	264	25.185	65.041	-6.923	1.00	96.83	1SG	C
ATOM	3686	H	MET	264	21.098	65.032	-8.340	1.00	0.00		H
ATOM	3687	HA	MET	264	23.523	63.342	-8.751	1.00	0.00		H
ATOM	3688	HB2	MET	264	21.051	62.968	-6.997	1.00	0.00		H
ATOM	3689	HB3	MET	264	22.442	61.929	-7.009	1.00	0.00		H
ATOM	3690	HG2	MET	264	22.552	64.882	-6.188	1.00	0.00		H
ATOM	3691	HG3	MET	264	22.373	63.539	-5.104	1.00	0.00		H
ATOM	3692	HE1	MET	264	26.272	65.106	-6.872	1.00	0.00		H
ATOM	3693	HE2	MET	264	24.908	64.910	-7.968	1.00	0.00		H
ATOM	3694	HE3	MET	264	24.769	65.984	-6.571	1.00	0.00		H
ATOM	3695	N	ILE	265	20.496	62.605	-9.945	1.00106.66		1SG	N
ATOM	3696	CA	ILE	265	19.665	61.681	-10.729	1.00106.66		1SG	C
ATOM	3697	C	ILE	265	18.940	62.392	-11.895	1.00106.66		1SG	C
ATOM	3698	O	ILE	265	18.969	61.917	-13.028	1.00106.66		1SG	O
ATOM	3699	CB	ILE	265	18.684	60.894	-9.793	1.00106.66		1SG	C
ATOM	3700	CG2	ILE	265	17.767	59.923	-10.575	1.00106.66		1SG	C
ATOM	3701	CG1	ILE	265	19.409	60.143	-8.644	1.00106.66		1SG	C
ATOM	3702	CD1	ILE	265	18.475	59.493	-7.610	1.00106.66		1SG	C
ATOM	3703	H	ILE	265	20.023	63.430	-9.593	1.00	0.00		H
ATOM	3704	HA	ILE	265	20.312	60.942	-11.205	1.00	0.00		H
ATOM	3705	HB	ILE	265	18.030	61.634	-9.329	1.00	0.00		H
ATOM	3706	HG21	ILE	265	17.038	59.434	-9.932	1.00	0.00		H
ATOM	3707	HG22	ILE	265	17.173	60.432	-11.335	1.00	0.00		H
ATOM	3708	HG23	ILE	265	18.344	59.146	-11.077	1.00	0.00		H
ATOM	3709	HG12	ILE	265	20.079	59.390	-9.061	1.00	0.00		H
ATOM	3710	HG13	ILE	265	20.042	60.832	-8.089	1.00	0.00		H
ATOM	3711	HD11	ILE	265	19.049	59.096	-6.773	1.00	0.00		H
ATOM	3712	HD12	ILE	265	17.764	60.216	-7.211	1.00	0.00		H
ATOM	3713	HD13	ILE	265	17.913	58.661	-8.034	1.00	0.00		H
ATOM	3714	N	GLY	266	18.239	63.498	-11.632	1.00	33.54	1SG	N
ATOM	3715	CA	GLY	266	17.040	63.854	-12.398	1.00	33.54	1SG	C
ATOM	3716	C	GLY	266	17.198	65.021	-13.373	1.00	33.54	1SG	C
ATOM	3717	O	GLY	266	16.313	65.874	-13.436	1.00	33.54	1SG	O
ATOM	3718	H	GLY	266	18.296	63.855	-10.685	1.00	0.00		H
ATOM	3719	HA2	GLY	266	16.639	62.998	-12.946	1.00	0.00		H
ATOM	3720	HA3	GLY	266	16.266	64.122	-11.681	1.00	0.00		H
ATOM	3721	N	CYS	267	18.318	65.075	-14.100	1.00	35.97	1SG	N

```
ATOM   3722  CA  CYS   267      18.544  65.891 -15.302  1.00 35.97       1SG C
ATOM   3723  C   CYS   267      19.734  65.346 -16.112  1.00 35.97       1SG C
ATOM   3724  O   CYS   267      20.506  66.113 -16.687  1.00 35.97       1SG O
ATOM   3725  CB  CYS   267      18.595  67.419 -15.014  1.00 35.97       1SG C
ATOM   3726  SG  CYS   267      16.991  68.217 -15.355  1.00 35.97       1SG S
ATOM   3727  H   CYS   267      19.024  64.380 -13.895  1.00  0.00           H
ATOM   3728  HA  CYS   267      17.697  65.708 -15.967  1.00  0.00           H
ATOM   3729  HB2 CYS   267      18.905  67.612 -13.992  1.00  0.00           H
ATOM   3730  HB3 CYS   267      19.320  67.934 -15.643  1.00  0.00           H
ATOM   3731  HG  CYS   267      16.300  67.251 -14.723  1.00  0.00           H
ATOM   3732  N   HIE   268      19.913  64.014 -16.127  1.00 59.23       1SG N
ATOM   3733  CA  HIE   268      20.903  63.339 -16.979  1.00 59.23       1SG C
ATOM   3734  C   HIE   268      20.277  62.962 -18.342  1.00 59.23       1SG C
ATOM   3735  O   HIE   268      20.983  62.496 -19.235  1.00 59.23       1SG O
ATOM   3736  ND1 HIE   268      23.078  63.115 -14.711  1.00 59.23       1SG N
ATOM   3737  CG  HIE   268      22.063  62.184 -14.965  1.00 59.23       1SG C
ATOM   3738  CB  HIE   268      21.389  62.033 -16.304  1.00 59.23       1SG C
ATOM   3739  NE2 HIE   268      22.698  61.974 -12.849  1.00 59.23       1SG N
ATOM   3740  CD2 HIE   268      21.855  61.451 -13.816  1.00 59.23       1SG C
ATOM   3741  CE1 HIE   268      23.387  62.956 -13.430  1.00 59.23       1SG C
ATOM   3742  H   HIE   268      19.202  63.436 -15.705  1.00  0.00           H
ATOM   3743  HA  HIE   268      21.765  63.984 -17.164  1.00  0.00           H
ATOM   3744  HB2 HIE   268      20.556  61.338 -16.191  1.00  0.00           H
ATOM   3745  HB3 HIE   268      22.106  61.538 -16.960  1.00  0.00           H
ATOM   3746  HD2 HIE   268      21.167  60.643 -13.620  1.00  0.00           H
ATOM   3747  HE1 HIE   268      24.120  63.559 -12.911  1.00  0.00           H
ATOM   3748  HE2 HIE   268      22.757  61.694 -11.871  1.00  0.00           H
ATOM   3749  N   LEU   269      18.963  63.162 -18.483  1.00 36.57       1SG N
ATOM   3750  CA  LEU   269      18.100  63.079 -19.647  1.00 36.57       1SG C
ATOM   3751  C   LEU   269      17.043  64.176 -19.426  1.00 36.57       1SG C
ATOM   3752  O   LEU   269      16.206  64.380 -20.334  1.00 36.57       1SG O
ATOM   3753  CB  LEU   269      17.461  61.677 -19.799  1.00 36.57       1SG C
ATOM   3754  CG  LEU   269      18.413  60.497 -20.089  1.00 36.57       1SG C
ATOM   3755  CD2 LEU   269      19.106  60.631 -21.455  1.00 36.57       1SG C
ATOM   3756  CD1 LEU   269      17.653  59.165 -20.001  1.00 36.57       1SG C
ATOM   3757  OXT LEU   269      17.027  64.741 -18.303  1.00 36.57       1SG O1-
ATOM   3758  H   LEU   269      18.478  63.730 -17.793  1.00  0.00           H
ATOM   3759  HA  LEU   269      18.661  63.347 -20.542  1.00  0.00           H
ATOM   3760  HB2 LEU   269      16.899  61.466 -18.888  1.00  0.00           H
ATOM   3761  HB3 LEU   269      16.711  61.723 -20.591  1.00  0.00           H
ATOM   3762  HG  LEU   269      19.183  60.476 -19.318  1.00  0.00           H
ATOM   3763 HD21 LEU   269      19.757  59.779 -21.651  1.00  0.00           H
ATOM   3764 HD22 LEU   269      18.379  60.696 -22.264  1.00  0.00           H
ATOM   3765 HD23 LEU   269      19.730  61.524 -21.494  1.00  0.00           H
ATOM   3766 HD11 LEU   269      18.318  58.317 -20.165  1.00  0.00           H
ATOM   3767 HD12 LEU   269      17.197  59.040 -19.018  1.00  0.00           H
ATOM   3768 HD13 LEU   269      16.854  59.117 -20.742  1.00  0.00           H
ENDMDL
END
```
